# Supplementary material for: Safety of topical corticosteroids in atopic eczema: an umbrella review
Source: BMJ Open. 2021 Jul 7;11(7):e046476. doi: 10.1136/bmjopen-2020-046476 (PMC8264889; doi:10.1136/bmjopen-2020-046476)
Supplement: Supplementary data [file bmjopen-2020-046476supp001.pdf]

Appendix 1: Search strategies

Search facets for searches:
Topical steroids
Eczema
Systematic reviews

PubMed search

Uses PubMed Clinical Queries systematic review filter (command systematic[sb]):
https://www.nlm.nih.gov/bsd/pubmed_subsets/sysreviews_strategy.html
(steroid* OR corticosteroid* OR glucocorticosteroid* OR glucocorticoid* OR glucocorticoids[MeSH Terms] OR alclometasone OR alclomethasone OR amcinonide OR beclometasone OR beclomethasone OR beclomethasone[MeSH Terms] OR betametasone OR betamethasone OR betamethasone[MeSH Terms] OR clobetasol OR clobetasol[MeSH Terms] OwR clobetasone OR desonide OR desonide[MeSH Terms] OR desoximetasone OR desoximetasone[MeSH Terms] OR diflorasone OR diflucortolone OR diflucortolone[MeSH Terms] OR fludroxycortide OR flumetasone OR flumethasone OR flumethasone[MeSH Terms] OR fluocinolone OR fluocinolone acetonide[MeSH Terms] OR fluocinonide OR fluocinonide[MeSH Terms] OR fluocortolone OR fluocortolone[MeSH Terms] OR flurandrenolide OR flurandrenolone OR flurandrenolone[MeSH Terms] OR fluticasone OR halcinonide OR halcinonide[MeSH Terms] OR halobetasol OR halometasone OR hydrocortisone OR hydrocortisone[MeSH Terms] OR methylprednisolone OR methylprednisolone[MeSH Terms] OR mometasone OR triamcinolone OR triamcinolone[MeSH Terms]) AND ("dermatitis, atopic"[MeSH Terms] OR "eczema"[MeSH Terms] OR "neurodermatitis"[MeSH Terms] OR eczema OR "atopic dermatitis" OR neurodermatitis) AND (systematic[sb] OR "systematic review")

Ovid MEDLINE search

Uses SIGN MEDLINE systematic review filter:
http://www.sign.ac.uk/search-filters.html
Ovid MEDLINE(R) Epub Ahead of Print, In-Process & Other Non-Indexed Citations, Ovid MEDLINE(R) Daily, Ovid MEDLINE and Versions(R)
MEDLINE eczema steroid systematic reviews
1. Meta-Analysis as Topic/
2. meta analy$.tw.
3. metaanaly$.tw.
4. Meta-Analysis/
5. (systematic adj (review$1 or overview$1)).tw.
6. exp Review Literature as Topic/
7. or/1-6
8. cochrane.ab.
9. embase.ab.
10. (psychlit or psyclit).ab.
11. (psychinfo or psycinfo).ab.
12. (cinahl or cinhal).ab.
13. science citation index.ab.
14. bids.ab.
15. cancerlit.ab.
16. or/8-15
17. reference list$.ab.
18. bibliograph$.ab.

19. hand-search$.ab.
20. relevant journals.ab.
21. manual search$.ab.
22. or/17-21
23. selection criteria.ab.
24. data extraction.ab.
25. 23 or 24
26. Review/
27. 25 and 26
28. Comment/
29. Letter/
30. Editorial/
31. animal/
32. human/
33. 31 not (31 and 32)
34. or/28-30,33
35. 7 or 16 or 22 or 27
36. 35 not 34
37. steroid$.mp.
38. corticosteroid$.mp.
39. glucocorticosteroid$.mp.
40. glucocorticoid$.mp.
41. exp Glucocorticoids/
42. alclometasone.mp.
43. alclomethasone.mp.
44. amcinonide.mp.
45. beclometasone.mp.
46. beclomethasone.mp.
47. exp Beclomethasone/
48. betametasone.mp.
49. betamethasone.mp.
50. exp Betamethasone/
51. clobetasol.mp.
52. exp Clobetasol/
53. clobetasone.mp.
54. desonide.mp.
55. exp Desonide/
56. desoximetasone.mp.
57. exp Desoximetasone/
58. diflorasone.mp.
59. diflucortolone.mp.
60. exp Diflucortolone/
61. fludroxycortide.mp.
62. flumetasone.mp.
63. flumethasone.mp.
64. exp Flumethasone/
65. fluocinolone.mp.
66. exp Fluocinolone Acetonide/
67. fluocinonide.mp.

68. exp Fluocinonide/
69. fluocortolone.mp.
70. exp Fluocortolone/
71. flurandrenolide.mp.
72. flurandrenolone.mp.
73. exp Flurandrenolone/
74. fluticasone.mp.
75. halcinonide.mp.
76. exp Halcinonide/
77. halobetasol.mp.
78. halometasone.mp.
79. hydrocortisone.mp.
80. exp Hydrocortisone/
81. methylprednisolone.mp.
82. exp methylprednisolone/
83. mometasone.mp.
84. triamcinolone.mp.
85. exp Triamcinolone/
86. or/37-85
87. exp dermatitis, atopic/
88. exp eczema/
89. exp neurodermatitis/
90. eczema.mp.
91. atopic dermatitis.mp.
92. neurodermatitis.mp.
93. or/87-92
94. 36 and 86 and 93

Ovid Embase search

Uses SIGN Embase systematic review filter:
http://www.sign.ac.uk/search-filters.html
Embase 1974 to 2017 October 23
Embase eczema steroid systematic reviews

1. exp Meta Analysis/
2. ((meta adj analy$) or metaanalys$).tw.
3. (systematic adj (review$1 or overview$1)).tw.
4. or/1-3
5. cancerlit.ab.
6. cochrane.ab.
7. embase.ab.
8. (psychlit or psyclit).ab.
9. (psychinfo or psycinfo).ab.
10. (cinahl or cinhal).ab.
11. science citation index.ab.
12. bids.ab.
13. or/5-12
14. reference lists.ab.
15. bibliograph$.ab.
16. hand-search$.ab.

17. manual search$.ab.
18. relevant journals.ab.
19. or/14-18
20. data extraction.ab.
21. selection criteria.ab.
22. 20 or 21
23. review.pt.
24. 22 and 23
25. letter.pt.
26. editorial.pt.
27. animal/
28. human/
29. 27 not (27 and 28)
30. or/25-26,29
31. 4 or 13 or 19 or 24
32. 31 not 30
33. steroid$.mp.
34. corticosteroid$.mp.
35. exp corticosteroid/
36. glucocorticosteroid$.mp.
37. glucocorticoid$.mp.
38. exp glucocorticoid/
39. alclometasone.mp.
40. alclomethasone.mp.
41. amcinonide.mp.
42. beclometasone.mp.
43. beclomethasone.mp.
44. betametasone.mp.
45. betamethasone.mp.
46. clobetasol.mp.
47. clobetasone.mp.
48. desonide.mp.
49. desoximetasone.mp.
50. diflorasone.mp.
51. diflucortolone.mp.
52. fludroxycortide.mp.
53. flumetasone.mp.
54. flumethasone.mp.
55. fluocinolone.mp.
56. fluocinonide.mp.
57. fluocortolone.mp.
58. flurandrenolide.mp.
59. flurandrenolone.mp.
60. fluticasone.mp.
61. halcinonide.mp.
62. halobetasol.mp.
63. halometasone.mp.
64. hydrocortisone.mp.
65. methylprednisolone.mp.

66. mometasone.mp.
67. triamcinolone.mp.
68. or/33-67
69. exp atopic dermatitis/
70. exp eczema/
71. exp neurodermatitis/
72. eczema.mp.
73. atopic dermatitis.mp.
74. neurodermatitis.mp.
75. or/69-74
76. 32 and 68 and 75

Epistemonikos

(steroid* OR corticosteroid* OR glucocorticosteroid* OR glucocorticoid* OR alclometasone OR alclomethasone OR amcinonide OR beclometasone OR beclomethasone OR betametasone OR betamethasone OR clobetasol OR clobetasone OR desonide OR desoximetasone OR diflorasone OR diflucortolone OR fludroxycortide OR flumetasone OR flumethasone OR fluocinolone OR fluocinonide OR fluocortolone OR flurandrenolide OR flurandrenolone OR fluticasone OR halcinonide OR halobetasol OR halometasone OR hydrocortisone OR methylprednisolone OR mometasone OR triamcinolone) AND (eczema OR "atopic dermatitis" OR neurodermatitis)
Enter search into advanced search and choose “**systematic review**” from drop-down box for “Publication type”:
https://www.epistemonikos.org/advanced_search?q=(steroid*%20OR%20corticosteroid*%20OR%20glucocorticosteroid*%20OR%20glucocorticoid*%20OR%20alclometasone%20OR%20alclomethasone%20OR%20amcinonide%20OR%20beclometasone%20OR%20beclomethasone%20OR%20betametasone%20OR%20betamethasone%20OR%20clobetasol%20OR%20clobetasone%20OR%20desonide%20OR%20desoximetasone%20OR%20diflorasone%20OR%20diflucortolone%20OR%20fludroxycortide%20OR%20flumetasone%20OR%20flumethasone%20OR%20fluocinolone%20OR%20fluocinonide%20OR%20fluocortolone%20OR%20flurandrenolide%20OR%20flurandrenolone%20OR%20fluticasone%20OR%20halcinonide%20OR%20halobetasol%20OR%20halometasone%20OR%20hydrocortisone%20OR%20methylprednisolone%20OR%20mometasone%20OR%20triamcinolone)%20AND%20(eczema%20OR%20%22atopic%20dermatitis%22%20OR%20neurodermatitis)&protocol=no&classification=systematic-review

Cochrane Library

(steroid* OR corticosteroid* OR glucocorticosteroid* OR glucocorticoid* OR [mh "glucocorticoids"] OR alclometasone OR alclomethasone OR amcinonide OR beclometasone OR beclomethasone OR betametasone OR betamethasone OR clobetasol OR clobetasone OR desonide OR desoximetasone OR diflorasone OR diflucortolone OR fludroxycortide OR flumetasone OR flumethasone OR fluocinolone OR fluocinonide OR fluocortolone OR flurandrenolide OR flurandrenolone OR fluticasone OR halcinonide OR halobetasol OR halometasone OR hydrocortisone OR methylprednisolone OR mometasone OR triamcinolone) AND ([mh "eczema"] OR [mh "dermatitis, atopic"] OR [mh "neurodermatitis"] OR eczema OR "atopic dermatitis" OR neurodermatitis)
“Search all text” option chosen.
Cochrane Reviews, **Other Reviews** (i.e. DARE), and **Technology Assessments** (i.e. HTA) chosen.
