# Supplementary material for: Safety of topical corticosteroids in atopic eczema: an umbrella review
Source: BMJ Open. 2021 Jul 7;11(7):e046476. doi: 10.1136/bmjopen-2020-046476 (PMC8264889; doi:10.1136/bmjopen-2020-046476)
Supplement: Supplementary data [file bmjopen-2020-046476supp002.pdf]

Appendix 2 - list of excluded studies with reasons

Excluded study	Reason for exclusion
Abramovits 2005 (1)	Not a systematic review
Abramovits 2006 (2)	Not a systematic review
Anonymous 1995 (3)	Not a systematic review
Anonymous 1999 (4)	Not a systematic review
Anonymous 2004 (5)	Not a systematic review
Anonymous 2005 (6)	Abstract
Anonymous 2007 (7)	Not a systematic review
Anonymous 2015 (8)	Abstract
Anonymous 2015 (9)	Abstract
Aslam 2014 (10)	Not a systematic review
Barfield 2017 (11)	Wrong intervention (not topical corticosteroids)
Batchelor 2010 (12)	Not a systematic review
Bath-Hextall 2010 (13)	Updated version of a Cochrane review (non-Cochrane) but no additional safety data
Bigby 2001 (14)	Commentary paper
Bonchak 2017 (15)	Wrong intervention (not topical corticosteroids)
Birnie 2008 (16)	Wrong intervention (not topical corticosteroids)
Boucher 2001 (17)	Not a systematic review
Broersen 2015 (18)	Unable to extract separate data for atopic eczema patients
Cameron 2000 (19)	Commentary paper
Carbone 2010 (20)	Not a systematic review
Chavigny 2005 (21)	Not a systematic review
Chi 2009 (22)	Unable to extract separate data for atopic eczema patients
Chi 2015 (23)	Unable to extract separate data for atopic eczema patients
Chia 2015 (24)	Not a systematic review
Chu 1995 (25)	Not a systematic review
Conroy 2004 (26)	Not a systematic review
Das 2017 (27)	Not a systematic review
El-Batawy 2009 (28)	No safety outcome
Fleischer Jr 2010 (29)	Wrong intervention (not topical corticosteroids)
Frohna 2005 (30)	Commentary paper
Froschl 2007 (31)	Duplicate record of an included systematic review
Furue 2006 (32)	Not a systematic review
Furue 2006 (33)	Not a systematic review
Garside 2005 (34)	No safety outcome
Ghajar 2019 (35)	'Subgroup analysis' of an included review (Wood Heickman 2018) – no additional safety data
Goustas 2003 (36)	Not a systematic review
Green 2005 (37)	Duplicate record of an included systematic review
Green 2004 (38)	Duplicate record of an included systematic review
Halling-Overgaard 2017 (39)	Skin atrophy is not assessed clinically in this review
Health Technology Assessment Database 2004 (40)	Not a systematic review
Health Technology Assessment Database 2004 (41)	Abstract – unable to find the full publication
Health Technology Assessment Database 2001 (42)	Abstract – unable to find the full publication
Health Technology Assessment Database 2004 (43)	Abstract – unable to find the full publication
Hannuksela 2000 (44)	Wrong intervention (not topical corticosteroids)
Hebert 2006 (45)	Wrong intervention (not topical corticosteroids)

Hoare 2000 [46]	Duplicate record of an included systematic review
Hon 2011 [47]	Wrong intervention (not topical corticosteroids)
Hulshof 2017 [48]	Wrong intervention (not topical corticosteroids)
Hussain 2016 [49]	Not a systematic review
Kaufman 2016 [50]	Abstract
Legendre 2015 [51]	Abstract
Li 2017 [52]	Abstract
Li 2017 [53]	No safety outcome
Meffert 1999 [54]	Not a systematic review
Mooney 2015 [55]	Not a systematic review
Murashkin 2016 [56]	Not a systematic review
Nankervis 2013 [57]	Abstract
Nankervis 2016 [58]	Duplicate record of an included systematic review
Nankervis 2017 [59]	Duplicate record of an included systematic review
Nowak 2017 [60]	No safety outcome
Orlow 2007 [61]	Not a systematic review
Pan 2013 [62]	No safety outcome
Park-Wyllie 2000 [63]	Unable to extract separate data for atopic eczema patients
Payne 2019 [64]	Wrong intervention (not topical corticosteroids)
Phipatanakul 2006 [65]	Commentary paper
Radovic 2017 [66]	Wrong intervention (not topical corticosteroids)
Ricci 2007 [67]	Not a systematic review
Ruzicka 1999 [68]	Wrong intervention (not topical corticosteroids)
Sanchez 2014 [69]	Not a systematic review
Schiffner 2003 [70]	No safety outcome
Schmitt 2011 [71]	Not a systematic review
Schmitt 2011 [72]	Duplicate record of an included systematic review
Sher 2012 [73]	Abstract
Sher 2012 [74]	No safety outcome
Siegfried 2013 [75]	Not a systematic review
Siegfried 2018 [76]	Not a systematic review
Silverberg 2014 [77]	Not a systematic review
Simpson 2010 [78]	Not a systematic review
Spada 2018 [79]	Not a systematic review
Torii 2003 [80]	No safety outcome
Torley 2013 [81]	Not a systematic review
Uppal 2020 [82]	Wrong intervention (not topical corticosteroids)
Van Zuuren 2017 [83]	No safety outcome (abridged Cochrane review)
Wat 2014 [84]	Wrong patient population (not atopic eczema)
Wellington 2004 [85]	Not a systematic review
Williams 2007 [86]	Not a systematic review
Williams 2008 [87]	Not a systematic review
Williams 2010 [88]	Not a systematic review
Wollenberg 2018 [89]	Not a systematic review

1. Abramovits W, Boguniewicz M, Paller A, Whitaker-Worth D, Prendergast M, Tokar M, et al. The economics of topical immunomodulators for the treatment of atopic dermatitis. *Pharmacoeconomics*. 2005;**23**(6):543-66.
2. Abramovits W, Hung P, Tong K. Efficacy and economics of topical calcineurin inhibitors for the treatment of atopic dermatitis. *Am J Clin Dermatol*. 2006;**7**(4):213-22.
3. Anonymous. Once-a-day topical corticosteroids. *Drug Ther Bull*. 1995;**33**(3):21-2.
4. Anonymous. Using topical corticosteroids in general practice. *MeReC Bulletin*. 1999;**10**(6):1-5.
5. Anonymous. Pimecrolimus: Me-too: Too many risks, not beneficial enough in atopic dermatitis. *Prescrire International*. 2004;**13**(74):209+12.
6. Anonymous. Once-daily topical steroid dosing effective for atopic eczema. *J Fam Pract*. 2005;**54**(6):499-500.
7. Anonymous. Atopic dermatitis in the infant and child. [French, English]. *Nouvelles Dermatologiques*. 2007;**26**(SUPPL. 1):3-9.
8. Anonymous. Abstracts of the Australasian College of Dermatologists 48th Annual Scientific Meeting. *Australasian Journal of Dermatology Conference: 48th Annual Scientific Meeting of the Australasian College of Dermatologists Adelaide, SA Australia Conference Publication:*. 2015;**56**(no pagination).
9. Anonymous. 14th International Kidney Cancer Symposium. *BJU International Conference: 14th International Kidney Cancer Symposium Miami, FL United States Conference Publication:*. 2015;**116**(no pagination).
10. Aslam I, Sandoval L, Feldman S. What's new in the topical treatment of allergic skin diseases. *Curr Opin Allergy Clin Immunol*. 2014;**14**(5):436-50.
11. Barfield A, Brown H, Pernell P, Woodard J. Effectiveness of emollient therapy in pediatric patients with atopic dermatitis. *J Dermatol Nurses Assoc*. 2017;**9**(3):123-8.
12. Batchelor J, Grindlay D, Williams H. What's new in atopic eczema? An analysis of systematic reviews published in 2008 and 2009. *Clin Exp Dermatol*. 2010;**35**(8):823-7; quiz 7.
13. Bath-Hextall F, Birnie A, Ravenscroft J, Williams H. Interventions to reduce Staphylococcus aureus in the management of atopic eczema: An updated Cochrane review. *Br J Dermatol*. 2010;**163**(1):12-26.
14. Bigby M. A thorough systematic review of treatments for atopic eczema. *Arch Dermatol*. 2001;**137**(12):1635-6.
15. Bonchak J, Thareja S, Chen S, Quave C. Botanical Complementary and Alternative Medicine for Pruritus: a Systematic Review. *Curr Dermatol Rep*. 2017;**6**(4):248-55.
16. AJ B, FJ BH, JC R, HC W. Interventions to reduce Staphylococcus aureus in the management of atopic eczema. *Cochrane Database of Systematic Reviews*. 2008(3):CD003871.
17. Boucher M. Tacrolimus ointment for the treatment of atopic dermatitis (Structured abstract)2001; (4):[4 p.]. Available from: http://onlinelibrary.wiley.com/o/cochrane/clhta/articles/HTA-32001000957/frame.html.
18. Broersen L, Pereira A, Jorgensen J, Dekkers O. Adrenal insufficiency in corticosteroids use: Systematic review and meta-analysis. *J Clin Endocrinol Metab* 2015;**100**(6):2171-80.
19. Cameron F. 'Down to skin and bone'. *Australas J Dermatol*. 2000;**41**(3):146-8.
20. Carbone A, Siu A, Patel R. Pediatric atopic dermatitis: A review of the medical management. *Ann Pharmacother*. 2010;**44**(9):1448-58.
21. Chavigny J. The place of therapeutic education in the treatment of atopic dermatitis in children. [French]. *Ann Dermatol Venereol*. 2005;**132**(SPEC. ISS. 1):1S116-1S20.
22. Chi C, Lee C, Wojnarowska F, Kirtschig G. Safety of topical corticosteroids in pregnancy. *Cochrane Database of Systematic Reviews*. 2009;**(3) (no pagination)**(CD007346).

23. Chi C-C, Wang S-H, Wojnarowska F, Kirtschig G, Davies E, Bennett C. Safety of topical corticosteroids in pregnancy2015; (10). Available from: http://onlinelibrary.wiley.com/doi/10.1002/14651858.CD007346.pub3/abstract.
24. Chia B, Tey H. Systematic review on the efficacy, safety, and cost-effectiveness of topical calcineurin inhibitors in atopic dermatitis. *Dermatitis*. 2015;**26**(3):122-32.
25. Chu A, Munn S. Fluticasone propionate in the treatment of inflammatory dermatoses. *Br J Clin Pract*. 1995;**49**(3):131-3.
26. Conroy S. New products for eczema. *Arch Dis Child Educ Pract Ed*. 2004;**89**(1):ep23-ep6.
27. Das A, Panda S. Use of Topical Corticosteroids in Dermatology: An Evidence-based Approach. *Indian J Dermatol*. 2017;**62**(3):237-50.
28. El-Batawy M, Bosseila M, Mashaly H, Hafez V. Topical calcineurin inhibitors in atopic dermatitis: a systematic review and meta-analysis. *J Dermatol Sci*. 2009;**54**(2):76-87.
29. Fleischer Jr A, Boguniewicz M. An approach to pruritus in atopic dermatitis: A critical systematic review of the tacrolimus ointment literature. *J Drugs Dermatol*. 2010;**9**(5):488-98.
30. Frohna J. Efficacy and tolerability of topical pimecrolimus and tacrolimus in the treatment of atopic dermatitis: Meta-analysis of randomised controlled trials. *J Pediatr*. 2005;**147**(1):126.
31. Froschl B, Arts D, Leopold C. Corticosteroid therapy in the treatment of pediatric patients with atopic dermatitis. *GMS Health Technol Assess*. 2007;**3**:Doc09.
32. Furue M. [Topical steroids and tacrolimus for the treatment of atopic dermatitis]. *Fukuoka Igaku Zasshi*. 2006;**97**(10):285-92.
33. Furue M, Uchi H, Moroi Y, Ogawa S, Nakahara T, Urabe K. Topical tacrolimus in the management of atopic dermatitis in Japan. *Dermatol Ther*. 2006;**19**(2):118-26.
34. Garside R, Stein K, Castelnuovo E, Pitt M, Ashcroft D, Dimmock P, et al. The effectiveness and cost-effectiveness of pimecrolimus and tacrolimus for atopic eczema: A systematic review and economic evaluation. *Health Technol Assess*. 2005;**9**(29):iii-122.
35. Ghajar DL, Wood Heickman LK, Conaway M, Rogol AD. Low Risk of Adrenal Insufficiency After Use of Low- to Moderate-Potency Topical Corticosteroids for Children With Atopic Dermatitis. *Clin Pediatr (Phila)*. 2019;**58**(4):406-12.
36. Goustas P, Cork M, Higson D. EumovateTM (clobetasone butyrate 0.05%) cream: A review of clinical efficacy and safety. *J Dermatolog Treat*. 2003;**14**(2):71-85.
37. Green C, Colquitt J, Kirby J, Davidson P. Topical corticosteroids for atopic eczema: Clinical and cost effectiveness of once-daily vs. more frequent use. *Br J Dermatol*. 2005;**152**(1):130-41.
38. Green C, Colquitt J, Kirby J, Davidson P, Payne E. Clinical and cost-effectiveness of once-daily versus more frequent use of same potency topical corticosteroids for atopic eczema: a systematic review and economic evaluation (Structured abstract)2004; (4):[1 p.]. Available from: http://onlinelibrary.wiley.com/o/cochrane/clhta/articles/HTA-32004000840/frame.html.
39. Halling-Overgaard A, Kezic S, Jakasa I, Engebretsen K, Maibach H, Thyssen J. Skin absorption through atopic dermatitis skin: a systematic review. *Br J Dermatol*. 2017;**177**(1):84-106.
40. Tacrolimus and pimecrolimus for atopic eczema (Structured abstract)2004; (4):[45 p.]. Available from: http://onlinelibrary.wiley.com/o/cochrane/clhta/articles/HTA-32004000795/frame.html.
41. Pimecrolimus (Elidel (R)) for atopic dermatitis (Structured abstract)2004; (4). Available from: http://onlinelibrary.wiley.com/o/cochrane/clhta/articles/HTA-32004000175/frame.html.
42. New products for atopic dermatitis - horizon scanning review (Structured abstract)2001; (4):[5 p.]. Available from: http://onlinelibrary.wiley.com/o/cochrane/clhta/articles/HTA-32002000841/frame.html.
43. Frequency of application of topical corticosteroids for atopic eczema (Structured abstract)2004; (4):[34 p.]. Available from: http://onlinelibrary.wiley.com/o/cochrane/clhta/articles/HTA-32004000794/frame.html.

44. Hannuksela M. [What is the best treatment for atopic dermatitis?]. *Duodecim*. 2000;**116**(21):2321-2.
45. Hebert A. Review of pimecrolimus cream 1% for the treatment of mild to moderate atopic dermatitis. *Clin Ther*. 2006;**28**(12):1972-82.
46. Hoare C, Li WPA, Williams H. Systematic review of treatments for atopic eczema (Structured abstract)2000; 4(37):[1-191 pp.]. Available from: http://onlinelibrary.wiley.com/o/cochrane/cldare/articles/DARE-12001008089/frame.html.
47. Hon KL, Chan BC, Leung PC. Chinese herbal medicine research in eczema treatment. *Chin Med*. 2011;**6 (no pagination)**(17).
48. Hulshof L, van't Land B, Sprikkelman A, Garssen J. Role of microbial modulation in management of atopic dermatitis in children. *Nutrients*. 2017;**9** (8):pii: E854.
49. Hussain Z, Sahudin S, Thu H, Shuid A, Bukhari S, Kumolosasi E. Recent Advances in Pharmacotherapeutic Paradigm of Mild to Recalcitrant Atopic Dermatitis. *Crit Rev Ther Drug Carrier Syst*. 2016;**33**(3):213-63.
50. Kaufman B, Alexis A. Safety and efficacy of topical calcineurin inhibitors and topical steroids in atopic dermatitis in skin of color: A systematic review. *Exp Dermatol* 2016;**25**:50.
51. Legendre L, Barnetche T, Juliette M, Meyer N, Paul C. Risk of lymphoma in atopic dermatitis: A systematic review and metaanalysis of epidemiologic studies. *J Am Acad Dermatol*. 2015;**1**:AB75.
52. Li A, Yin E, Antaya R. Topical corticosteroid phobia and fear in atopic dermatitis: A systematic review. *J Invest Dermatol*. 2017;**137 (5 Supplement 1)**:S26.
53. Li A, Yin E, Antaya R. Topical corticosteroid phobia in atopic dermatitis: A systematic review. *JAMA Dermatol*. 2017;**153**(10):1036-42.
54. Meffert H, Schuppler J. Methylprednisolone aceponate lotion for the treatment of acute eczema. [German]. *H+G Zeitschrift fur Hautkrankheiten*. 1999;**74**(2):89-94.
55. Mooney E, Rademaker M, Dailey R, Daniel B, Drummond C, Fischer G, et al. Adverse effects of topical corticosteroids in paediatric eczema: Australasian consensus statement. *Australas J Dermatol*. 2015;**56**(4):241-51.
56. Murashkin N, Materikin A, Ambarchyan E, Epishev R. Current views on the pathogenesis and principles of external treatment of atopic dermatitis in children. [Russian]. *Voprosy Sovremennoi Pediatrii - Current Pediatrics*. 2016;**15**(6):584-9.
57. Nankervis H, Delamere F, Williams H. Systematic review of treatments for eczema informs clinical practice and highlights research gaps. *J Invest Dermatol*. 2013;**133**:S176.
58. Nankervis H, Thomas K, Delamere F, Barbarot S, Rogers N, Williams H. Programme Grants for Applied Research. 2016.
59. Nankervis H, Thomas K, Delamere F, Barbarot S, Smith S, Rogers N, et al. What is the evidence base for atopic eczema treatments? A summary of published randomized controlled trials. *Br J Dermatol*. 2017;**176**(4):910-27.
60. Novak N. Allergen specific immunotherapy for atopic dermatitis. *Curr Opin Allergy Clin Immunol*. 2007;**7**(6):542-6.
61. Orlow S. Topical calcineurin inhibitors in pediatric atopic dermatitis: a critical analysis of current issues. *Paediatr Drugs*. 2007;**9**(5):289-99.
62. Pan M, Heinecke G, Bernardo S, Tsui C, Levitt J. Urea: A comprehensive review of the clinical literature. *Dermatol Online J*. 2013;**19**(11).
63. Park-Wyllie L, Mazzotta P, Pastuszak A, Moretti M, Beique L, Hunnisett L, et al. Birth defects after maternal exposure to corticosteroids: prospective cohort study and meta-analysis of epidemiological studies (Structured abstract)2000; 62(6):[385-92 pp.]. Available from: http://onlinelibrary.wiley.com/o/cochrane/cldare/articles/DARE-12001003037/frame.html.
64. Payne J, Habet KA, Pona A, Feldman SR. A Review of Topical Corticosteroid Foams. *J Drugs Dermatol*. 2019;**18**(8):756-70.
65. Phipatanakul W. Efficacy and tolerability of pimecrolimus and tacrolimus in the treatment of atopic dermatitis: Meta-analysis of randomised controlled trials. Commentary. *Pediatrics*. 2006;**118**(SUPPL. 1):S16-S7.

66. Radovic T, Kostovic K, Ceovic R, Mokos Z. Topical calcineurin inhibitors and malignancy risk. *Int J Cancer Manag*. 2017;**10 (4) (no pagination)**(e6173).
67. Ricci G, Dondi A, Patrizi A. Role of topical calcineurin inhibitors on atopic dermatitis of children. *Curr Med Chem*. 2007;**14**(14):1579-91.
68. Ruzicka T, Assmann T, Homey B. Tacrolimus: The drug for the turn of the millennium? *Arch Dermatol*. 1999;**135**(5):574-80.
69. Sanchez J, Paez B, Macias A, Olmos C, De Falco A. Atopic dermatitis guideline. Position paper from the Latin American Society of allergy, asthma and immunology. *Revista Alergia Mexico*. 2014;**61**(3):178-211.
70. Schiffner R, Schiffner-Rohe J, Landthaler M, Stolz W. Treatment of atopic dermatitis and impact on quality of life: a review with emphasis on topical non-corticosteroids. *Pharmacoeconomics*. 2003;**21**(3):159-79.
71. Schmitt J, Apfelbacher C, Flohr C. Eczema. *BMJ Clin Evid*. 2011(pagination).
72. Schmitt J, Kobyletzki L, Svensson A, Apfelbacher C. Efficacy and tolerability of proactive treatment with topical corticosteroids and calcineurin inhibitors for atopic eczema: systematic review and meta-analysis of randomized controlled trials (Provisional abstract)2011; 164(2):[415-28 pp.]. Available from: http://onlinelibrary.wiley.com/o/cochrane/cldare/articles/DARE-12011001466/frame.html.
73. Sher L, Chang J, Patel I, Balkrishnan R, Fleischer A. Relieving the pruritus of atopic dermatitis: A meta-analysis. *Value in Health*. 2012;**15 (4)**:A249-A50.
74. Sher LG, Chang J, Patel I, Balkrishnan R, Fleischer Jr A. Relieving the pruritus of atopic dermatitis: A meta-analysis. *Acta Derm Venereol*. 2012;**92**(5):455-61.
75. Siegfried E, Jaworski J, Hebert A. Topical calcineurin inhibitors and lymphoma risk: evidence update with implications for daily practice. *Am J Clin Dermatol*. 2013;**14**(3):163-78.
76. Siegfried E, Jaworski J, Mina-Osorio P. A Systematic Scoping Literature Review of Publications Supporting Treatment Guidelines for Pediatric Atopic Dermatitis in Contrast to Clinical Practice Patterns. *Dermatol Ther (Heidelb)*. 2018;**8**(3):349-77.
77. Silverberg J. Atopic dermatitis: An evidence-based treatment update. *Am J Clin Dermatol*. 2014;**15**(3):149-64.
78. Simpson E. Atopic dermatitis: A review of topical treatment options. *Curr Med Res Opin*. 2010;**26**(3):633-40.
79. Spada F, Barnes T, Greive K. Comparative safety and efficacy of topical mometasone furoate with other topical corticosteroids. *Australas J Dermatol*. 2018;**59**(3):e168-e74.
80. Torii H. The Evaluation of Tacrolimus on Evidence-based Medicine (EBM). [Japanese]. *Skin Research*. 2003;**2**(SUPPL. 3):27-30.
81. Torley D, Futamura M, Williams H, Thomas K. What's new in atopic eczema? An analysis of systematic reviews published in 2010-11. *Clin Exp Dermatol*. 2013;**38**(5):449-56.
82. Uppal SK, Chat VS, Kearns DG, Wu JJ. Topical Agents Currently in Phase II or Phase III Trials for Atopic Dermatitis. *J Drugs Dermatol*. 2020;**19**(10):956-9.
83. van Zuuren E, Fedorowicz Z, Arents B. Emollients and moisturisers for eczema: abridged Cochrane systematic review including GRADE assessments. *Br J Dermatol*. 2017.
84. Wat H, Dytoc M. Off-label uses of topical vitamin D in dermatology: A systematic review. *J Cutan Med Surg*. 2014;**18**(2):91-108.
85. Wellington K, Noble S. Pimecrolimus: A review of its use in atopic dermatitis. *Am J Clin Dermatol*. 2004;**5**(6):479-95.
86. Williams H. Established corticosteroid creams should be applied only once daily in patients with atopic eczema. *BMJ*. 2007;**334**(7606):1272.
87. Williams H, Grindlay D. What's new in atopic eczema? An analysis of the clinical significance of systematic reviews on atopic eczema published in 2006 and 2007. *Clin Exp Dermatol*. 2008;**33**(6):685-8.

88. Williams H, Grindlay D. What's new in atopic eczema? An analysis of systematic reviews published in 2007 and 2008. Part 2. Disease prevention and treatment. *Clin Exp Dermatol*. 2010;**35**(3):223-7.
89. Wollenberg A, Barbarot S, Bieber T, Christen-Zaech S, Deleuran M, Fink-Wagner A, et al. Consensus-based European guidelines for treatment of atopic eczema (atopic dermatitis) in adults and children: part I. *J Eur Acad Dermatol Venereol*. 2018;**32**(5):657-82.
