# Supplementary material for: Safety of topical corticosteroids in atopic eczema: an umbrella review
Source: BMJ Open. 2021 Jul 7;11(7):e046476. doi: 10.1136/bmjopen-2020-046476 (PMC8264889; doi:10.1136/bmjopen-2020-046476)
Supplement: Supplementary data [file bmjopen-2020-046476supp003.pdf]

Appendix 3: Prospero search

Prospero – searched up to 23rd March 2021 – search results from 'Eczema OR dermatitis'

Potentially relevant ongoing systematic reviews:

Prospero ID	Date registered	Title	Status	Anticipated completion date
CRD42015016525 (1)	11 February 2015	Effects of emollients in the management of atopic dermatitis in pediatric patients a systemic review and meta-analysis	Review ongoing	25 February 2016
CRD42015027873 (2)	04 November 2015	Interventions to improve quality of life in paediatric atopic dermatitis: a systematic review	Review ongoing	01 January 2016
CRD42020190452 (3)	14 July 2020	The association between topical calcineurin inhibitor use and risk of cancer: a systematic review and meta-analysis	Review ongoing	31 August 2020
CRD42020161558 (4)	28 April 2020	Efficacy of Non-Steroidal Topical Therapies for Atopic Dermatitis: A Systematic Review & Meta-Analysis	Review ongoing	31 May 2020
CRD42021230047 (5)	31 October 2021	A network meta-analysis of five categories of external therapy of traditional Chinese for common diseases of dermatology	Review ongoing	31 October 2021

1. Tan Q, Tan C, Peng W, Shi Y, Xia L. Effects of emollients in the management of atopic dermatitis in pediatric patients a systemic review and meta-analysis [CRD42015016525] 2015 (accessed: 27/03/21). Available from: http://www.crd.york.ac.uk/PROSPERO/display_record.php?ID=CRD42015016525.
2. Yu A, Hong J, Lee M, Hong B. Interventions to improve quality of life in paediatric atopic dermatitis: a systematic review [CRD42015027873] 2015 (accessed: 27/03/21). Available from: http://www.crd.york.ac.uk/PROSPERO/display_record.php?ID=CRD42015027873.
3. Lam M, Zhu J, Tadrous M, Drucker A. The association between topical calcineurin inhibitor use and risk of cancer: a systematic review and meta-analysis [CRD42020190452]2020 (accessed: 27/03/2021). Available from: www.crd.york.ac.uk/prospero/display_record.php?RecordID=190452.
4. Lee K. Efficacy of Non-Steroidal Topical Therapies for Atopic Dermatitis: A Systematic Review & Meta-Analysis [CRD42020161558] 2020 (accessed: 27/03/2021). Available from: www.crd.york.ac.uk/prospero/display_record.php?RecordID=161558.
5. Ruirui L, Jing G, Dingxi B, Qian Y, Lin Z, Zhi Y, et al. A network meta-analysis of five categories of external therapy of traditional Chinese for common diseases of dermatology [CRD42021230047]2021 (assessed: 27/03/2021). Available from: www.crd.york.ac.uk/prospero/display_record.php?RecordID=230047.
