# Supplementary material for: Safety of topical corticosteroids in atopic eczema: an umbrella review
Source: BMJ Open. 2021 Jul 7;11(7):e046476. doi: 10.1136/bmjopen-2020-046476 (PMC8264889; doi:10.1136/bmjopen-2020-046476)
Supplement: Supplementary data [file bmjopen-2020-046476supp006.pdf]

Appendix 6 – Characteristics and safety data from the included studies

Study ID *(Systematic review*)*	Study design and study duration *(Quality assessment)*	Intervention and comparator	Participants	Cutaneous adverse events	Systemic adverse events	Unspecified adverse events
**How safe are topical corticosteroids compared to emollient or vehicle?**						
**Very potent topical corticosteroids**						
Breneman 2003 [(1)]<br>(unpublished)<br>***(Feldman 2005 [(2)] Nankervis [(3)])***	RCT<br>2 weeks treatment, then followed up for additional 2 weeks<br>*Cochrane risk of bias tool: randomisation described, allocation concealment unclear, intention-to-treat unclear.*	**Intervention:** Clobetasol propionate 0.05% lotion (twice a day) **(n=96)**<br>**Intervention:** Clobetasol propionate 0.05% emollient cream (twice a day) (n=100)<br>**Comparator:** Vehicle (n=33)	**Severity:** moderate to severe<br>**Age:** ≥ 12 years<br>**Sample size:** 229 participants	**<u>Local application site skin reactions</u>**<br>No clinically significant telangiectasia or skin thinning		**<u>Unspecified adverse events</u>**<br>Incidence comparable between groups.<br>**<u>Treatment-related adverse events</u>**<br>**Clobetasol lotion** = 4/96 patients (4.2%); **Clobetasol cream** = 1/100 patients (1%)<br>**Vehicle** = 6/33 patients (18.2%)<br>*(Difference between groups: p= 0.0006[a])*
Kimball 2008 [(4)] (trial a)<br>***(Frangos 2008 [(5)])***	RCT<br>Duration not specified in review<br>*Risk of bias not assessed in any of the included systematic reviews.*	**Intervention:** Clobetasol propionate emulsion formulation foam 0.05%<br>**Comparator:** Vehicle	**Severity:** not specified in the review<br>**Age:** not specified in the review<br>**Sample size:** not specified in the review			**<u>Incidence of adverse events or treatment related adverse events</u>**<br>**Clobetasol foam** = 8%<br>**Vehicle foam** = 10%<br>*(no significant differences between groups)*
Rosso 2009 [(6)]<br>***(Barnes 2009 [(7)])***	RCT<br>2 weeks treatment<br>*Risk of bias not assessed in any of the included systematic reviews.*	**Intervention:** Fluocinonide 0.1% cream (n=109)<br>**Comparator:** Vehicle (n=50)	**Severity:** not specified in the review<br>**Age:** not specified in the review<br>**Sample size:** 159 participants	**<u>Skin thinning</u>**<br>**Fluocinonide:** 6/109 participants (5.6%)<br>**Vehicle:** 2/50 participants (4.3%)<br>*(Difference between groups: p=0.69[a])*		
www.olux-e.com (online data) [(8)]<br>***(Frangos 2008 [(5)])***	Single arm study (observational)<br>2 weeks treatment	**Intervention:** Clobetasol propionate emollient foam (twice daily) (n=37)<br>**Comparator:** No comparator	**Severity:** ≥30% BSA<br>**Age:** ≥12 years old<br>**Sample size:** 37 participants		**<u>HPA axis suppression</u>**<br>6/37 patients (16%)<br>(not specified in the review *how it was measured)*	

	Risk of bias not assessed in any of the included systematic reviews.					
Kimball 2008 [(4)] (trial b)<br><br>***(Frangos 2008 [(5)]; Wood Heickman 2018 [(9)])***	Open label Phase II safety study<br><br>2 weeks treatment<br><br>*Risk of bias not assessed in any of the included systematic reviews.*	**Intervention:** Clobetasol propionate emollient foam 0.05% (twice daily) (n=52)<br><br>**Comparator:** No comparator	**Severity:** mild to severe<br><br>**Age:** children (from 6 years old) and adults<br><br>**Sample size:** 52 participants		**HPA axis suppression**<br>7/30 (23.3%) had adrenal insufficiency (ACTH stimulation testing, measuring serum cortisol levels).<br>• 47% of children (aged 6-11)<br>• 0% of adolescents (aged 12-17)<br>• 27% of adults (≥18 years)<br>Was reported as transient and reversible. After TCS discontinuation, children with biochemical adrenal insufficiency had complete resolution at retesting.	
Herz 1991 [(10)]<br><br>***(Barnes 2015 [(7)])***	Single arm study (observational)<br><br>*(2 weeks treatment)*<br><br>*Risk of bias not assessed in any of the included systematic reviews.*	**Intervention:** Clobetasol propionate (n=59)<br><br>**Comparator:** No comparator	**Severity:** not specified in the review<br><br>**Age:** not specified in the review<br><br>**Sample size:** 59 participants	**Skin thinning** 1 case of skin thinning reported (not clear if in a psoriasis or eczema patient – but assume its eczema as this is the topic of the systematic review).		
**Potent topical corticosteroids**						
Sugarman 2009 [(11)]<br><br>***(Van Zuuren 2017 [(12)])***	RCT<br><br>*(4 weeks treatment)*<br><br>*(Cochrane risk of bias tool: low risk of selection, attrition and other biases. Unclear risk of reporting and performance bias, High risk of detection bias. [(12)])*<br><br>*(Cochrane risk of bias tool: unclear risk of selection bias, high risk from no blinding. [(3)])*	**Intervention:** Fluticasone 0.05% cream twice daily (hydrocortisone 2.5% for the face and body folds) (n=62)<br><br>**Comparator:** Ceramide-dominant barrier repair formulation (EpiCeram) twice daily (emollient) (n=59)	**Severity:** moderate to severe<br><br>**Age:** children 6 months to 18 years (mean age 7.1 years)<br><br>**Sample size:** 121 participants			**Serious adverse events**<br>The participants did not report any in either group.<br><br>No further details regarding other possible treatment related adverse events were reported.

Griffiths 2002 [13]<br><br>***(Nankervis 2017 [3])***	RCT<br><br>*(up to 14 days treatment)*<br><br>*(Cochrane risk of bias tool: low risk of selection bias from sequence generation, unclear risk of selection bias from allocation concealment, low risk from blinding. [3])*	**Intervention:** Hydrocortisone 17-butyrate cream (0.1%) maximum application of 2g (four fingertip units) per day (n=49)<br><br>**Comparator:** Cipamfylline cream (1.5 mg of cipamfylline per gram of cream) used up to a maximum of 2 g (four fingertip units) of cream per day (emollient) (n=54)	**Severity:** not specified<br><br>**Age:** adults ≥18 years old<br><br>**Sample size:** 103 participants	No difference in cutaneous adverse events which were possibly or probably related to treatment in either group (p = 0.13)<br><br>The adverse events were mostly application site reactions, including itching, stinging or burning, and drug reactions.		**<u>Unspecified adverse events</u>**<br>**Hydrocortisone group:** 20/49 (40.8%) participants reported 41 adverse events in total.<br>**Emollient:** 29/52 (55.8%) participants reported 63 adverse events in total.<br>*(Difference between groups: p= 0.14 [a])*
Eichenfield 2006 [14]<br><br>***(Nankervis 2017 [3])***	RCT<br><br>*(4 weeks treatment)*<br><br>*Risk of bias not assessed*	**Intervention:** Fluticasone propionate four times daily (n=221)<br><br>**Comparator:** Vehicle four times daily (n=217)	**Severity:** moderate to severe<br><br>**Age:** children from 3 months old to 16 years old<br><br>**Sample size:** 438 children			**<u>Withdrawal due to adverse events</u>**<br>**Topical corticosteroids:** 4 participants in total from this study and from Hebert 2007<br><br>**<u>The number of participants reporting at least 1 adverse event</u>**<br>**Fluticasone:** 77/221 (34.8%) participants<br>**Vehicle:** 82/217 (37.8%) participants<br>*(Difference between groups: p=0.52[a])*
Wu 2013 [15]<br><br>***(Nankervis 2017 [3], Fishbein 2019 [16])***	RCT<br><br>*(10 days treatment)*<br><br>*(Cochrane risk of bias tool: low risk of selection bias from sequence generation. Unclear risk of selection bias from allocation concealment, unclear risk from blinding and other biases: Two out of 60 participants were excluded from the analyses as they used concomitant medication [3])*	**Intervention:** Mometasone furoate 0.1% cream, twice a day (n=20)<br><br>**Comparator:** placebo of distilled water in 1% dimethyl sulfoxide mixed with the identical cream base as used for the 15(R/S)-methyl-lipoxin A4 (n=20)<br><br>**Comparator:** 15(R/S)-methyl-lipoxin A4 0.1% cream (n=20)	**Severity:** all severities<br><br>**Age:** children from 1 month to 1 year old<br><br>**Sample size:** 60 participants		None of the safety tests (e.g. full blood count, kidney and liver function test, and electrocardiogram) showed any significant differences compared with baseline for all three treatment groups.	No clinical adverse events were reported.

Pellanda 2005 [17]<br>***(Nankervis 2017 [3])***	RCT<br>*(Duration not specified in the review)*<br>*Risk of bias not assessed*	**Intervention:** Triamcinolone acetonide<br>**Comparator:** Vehicle	**Severity:** mild to moderate<br>**Age:** not specified in the review<br>**Sample size:** not specified in the review	**Skin changes**<br>One report by a participant using placebo (no further details)		
Lebwohl 1996 [18]<br>***(Hoare 2000 [19])***	RCT<br>*(29 days treatment)*<br>*(Moher 1995 quality checklist: method and concealment of randomisation unclear, double blinded, large number of withdrawals and dropouts, no ITT analysis [19])*	**Intervention:** Fluticasone propionate ointment 0.005%<br>**Comparator:** Vehicle	**Severity:** not specified in the review<br>**Age:** not specified in the review<br>**Sample size:** 203 participants			The review authors only reported that *"Drug related adverse effects were rare"*
Lebwohl 1999 [20]<br>***(Hoare 2000 [19])***	RCT<br>*(29 days treatment)*<br>*(Moher 1995 quality checklist: method and concealment of randomisation unclear, double blinded, large number of withdrawals and dropouts, no ITT analysis [19])*	**Intervention:** Fluticasone propionate ointment 0.005%<br>**Comparator:** Vehicle	**Severity:** not specified in the review<br>**Age:** not specified in the review<br>**Sample size:** 169 participants			The review authors only reported that *"Drug related adverse effects were rare"*
Abramovitis 2010 [21]<br>***(Wood Heickman 2018 [9], Fishbein 2019 [16])***	RCT<br>(21 to 29 days treatment)<br>*Risk of bias not assessed in any of the included systematic reviews.*	**Intervention:** Hydrocortisone butyrate 0.1% cream, twice daily (n=131)<br>**Comparator:** Lipocream vehicle, twice daily (n=133)	**Severity:** Mild to moderate<br>**Age:** children 3 months to 18 years (mean 7.2 years)<br>**Sample size**: 264 children		**HPA axis suppression (no data for vehicle group)**<br>5/63 (7.9%) children in the hydrocortisone group (measured using ACTH stimulation testing, measuring serum cortisol levels)<br>After TCS discontinuation, children with biochemical adrenal insufficiency had complete resolution at retesting.	**The number of participants reporting at least 1 adverse event**<br>**Hydrocortisone:** 29/131 (22.1%) participants<br>**Vehicle:** 28/133 (21.1%) participants<br>*(Difference between groups: p=0.83 [a])*
Matheson 2008 [22]	RCT<br>(28 days treatment)	**Intervention:** Hydrocortisone butyrate 0.1% lotion, twice daily (n=139)	**Severity:** Mild to moderate			**The number of participants reporting at least 1 adverse event**<br>**Hydrocortisone:** 48/139 (34.5%)

(Fishbein 2019 [16])	*Risk of bias not assessed in any of the included systematic reviews.*	**Comparator:** Vehicle, twice daily (n=145)	**Age:** children 3 months to 18 years<br>**Sample size**: 284 children			participants<br>**Vehicle:** 56/145 (38.6%) participants<br>*(Difference between groups: p=0.48 [a])*
Friedlander 2002 [23]<br>***(Callen 2007 [24]; Wood Heickman 2018 [9])***	Single arm study (observational)<br>*(3 to 4 weeks)*<br>*Risk of bias not assessed in any of the included systematic reviews.*	**Intervention:** Fluticasone propionate cream 0.05% (n=43)<br>**Comparator:** No comparator	**Severity:** not specified in the review<br>**Age:** children 3 months to 6 years<br>**Sample size:** 43 participants		**HPA axis suppression**<br>2/43 (4.7%) children (measured using ACTH stimulation testing, measuring serum cortisol levels)<br>After TCS discontinuation, children with biochemical adrenal insufficiency had complete resolution at retesting.	
Eichenfield 2007 [25]<br>***(Wood Heickman 2018 [9])***	Single arm study (observational)<br>(4 weeks treatment)<br>*Risk of bias not assessed in any of the included systematic reviews.*	**Intervention:** Hydrocortisone butyrate 0.1% (n=20)<br>**Comparator:** No comparator	**Severity:** not specified in the review<br>**Age:** children (median or mean = 9 years)<br>**Sample size**: 20 children		**HPA axis suppression**<br>0/20 (0%) children (measured using ACTH stimulation testing, measuring serum cortisol levels)	
Hebert 2006 [26]<br>***(Wood Heickman 2018 [9])***	Single arm study (observational)<br>(3 to 4 weeks treatment)<br>*Risk of bias not assessed in any of the included systematic reviews.*	**Intervention:** Fluticasone propionate 0.05% lotion (n=42)<br>**Comparator:** No comparator	**Severity:** not specified in the review<br>**Age:** children (median or mean 2.6 years)<br>**Sample size**: 42 children		**HPA axis suppression**<br>0/42 (0%) children (measured using ACTH stimulation testing, measuring serum cortisol levels)	
**Moderate potency topical corticosteroids**						
De Belilovsky 2011 [27]<br>***(Van Zuuren 2017 [12])***	RCT<br>*(3 weeks treatment)*<br>*(Cochrane risk of bias tool: low risk of selection, attrition, reporting and other biases. Unclear risk of performance bias.*	**Intervention:** Hydrocortisone butyric propionate 0.1% twice daily (n=40)<br>**Comparator:** Stelatopia (2% sunflower oil, fatty acids, ceramides) twice daily (n=40)	**Severity:** mild to moderate<br>**Age:** children 4 months to 4 years (mean age 2.3 years)<br>**Sample size:** 80 participants			No participants reported adverse events

	High risk of detection bias. [12])<br>*(Cochrane risk of bias tool: unclear risk of selection bias and risk from blinding. [3])*					
Rosenthal 1980 [28]<br>***(Singh 2012 [29])***	RCT<br>(*14 days treatment)*<br>*(Delphi list: method of randomisation not described, allocation not concealed, blinded, no ITT analysis [29])*	**Intervention:** Clocortolone pivalate 0.1% cream (applied thrice daily)<br>**Comparator:** Vehicle (applied thrice daily)	**Severity:** not specified in the review<br>**Age:** not specified in the review<br>**Sample size:** 100 participants			No adverse events
Binder 1977 [30]<br>***(Singh 2012 [29])***	RCT<br>*(14 days treatment)*<br>*(Delphi list: method of randomisation not described, allocation not concealed, blinded, no ITT analysis [29])*	**Intervention:** Clocortolone pivalate (applied thrice daily) (n=17)<br>**Comparator:** Vehicle (n=12)	**Severity:** not specified in the review<br>**Age:** mean age 30 years<br>**Sample size:** 29 participants	**<u>Irritation and dryness</u>**<br>Clinically significant in one patient in each group – did not result in discontinuation.		
Rauschkol 1981 [31]<br>***(Fishbein 2019 [16])***	Within-participant RCT<br>*(14 days treatment)*<br>*Risk of bias not assessed in any of the included systematic reviews.*	**Intervention:** Halcinonide 0.025% cream, twice daily, on one arm<br>**Comparator:** Placebo cream unspecified, twice daily on the other arm at the same time	**Severity:** not reported<br>**Age:** children 7 months to 15 years (mean age 8 years)<br>**Sample size:** 86 children			**<u>The number of participants reporting at least 1 adverse event</u>**<br>**Halcinonide:** 4/86 (4.7%) participants<br>**Placebo:** 5/86 (5.8%) participants
Nolting 1991 [32]<br>***(De Tiedra 1997 [33])***	RCT (but safety data only presented for one arm)<br>*(21 days treatment)*<br>*Risk of bias not assessed in any of the included systematic reviews.*	**Intervention:** Prednicarbate cream 0.25% (2 applications per day) (n=34)<br>**Comparator:** mometasone cream 0.1% twice daily (no safety data given)	**Severity:** Disease duration = mean 4.1 years ± 2.7<br>**Age:** children 2-12 years (mean 6.6 ± 3.6).<br>**Sample size:** 34 participants (with safety data)			**<u>Adverse reactions</u>**<br>2/34 patients (5.9%)
Rampini 1992 [34]	RCT (but safety data only presented for one arm)	Intervention: Prednicarbate cream/unguent 0.25% (2 applications per day) (n=93)	**Severity:** not specified in the review			**<u>Adverse reactions</u>**<br>3/93 patients (3.2%)

(De Tiedra 1997 [33])	*(21 days treatment)*<br>*(Moher 1995 quality checklist: method and concealment of randomisation unclear, double blinded, two dropouts, no ITT analysis, [19])*	Comparator:methylprednisolone aceponate 0.1% once daily (no safety data given)	**Age:** children 0.3 to 14 years (mean 6.6).<br>**Sample size:** 93 participants (with safety data)			
Camacho 1996 [35]<br>***(De Tiedra 1997 [33])***	RCT (but safety data only presented for one arm)<br>*(21 days treatment)*<br>*(Moher 1995 quality checklist: method and concealment of randomisation unclear, double blinded, no ITT analysis, 14/49 dropouts, [19])*	**Intervention:** Prednicarbate cream 0.25% (2 applications per day) (n=49)<br>**Comparator:** fluocortolone pivalate cream 0.2% (no safety data given)	**Severity:** Disease duration= mean 6.2 years ± 8.2 (range 0.25 to 39 years).<br>**Age:** adults 19 to 65 years (mean 34.1 ± 12).<br>**Sample size:** 49 participants (with safety data)			**Adverse reactions**<br>4/49 patients (8.1%)
Gimenez Camarasa 1994 [36]<br>***(De Tiedra 1997 [33])***	RCT (but safety data only presented for one arm)<br>*(21 days treatment)*<br>*Risk of bias not assessed in any of the included systematic reviews.*	**Intervention:** Prednicarbate cream 0.25% (2 applications per day) (n=41)<br>**Comparator**: fluocinolone cream 0.025% twice daily (no safety data given)	**Severity:** Disease duration = mean 6.4 years ± 8.6 (range 0-40).<br>**Age:** adults 18 to 77 years (mean 37.6 ± 15.9).<br>**Sample size:** 41 participants (with safety data)			**Adverse reactions**<br>0/41 patients (0%)
Moshang 2001 [37]<br>***(Callen 2007 [24]; Wood Heickman 2018 [9])***	Single arm study (observational)<br>*(3 weeks treatment)*<br>*Risk of bias not assessed in any of the included systematic reviews.*	**Intervention:** Prednicarbate emollient cream 0.1%, twice daily (n=55)<br>**Comparator:** No comparator	**Severity:** not specified in the review<br>**Age:** children 4 months to 12 years<br>**Sample size:** 55 participants		**HPA axis suppression**<br>All normal (measured using ACTH stimulation testing, measuring serum cortisol levels)	
Conde 2008 [38]<br>***(Singh 2012 [29])***	Single arm study (observational)<br>*(4 weeks treatment)*	**Intervention:** Clocortolone pivalate cream 0.1% twice daily (n=10)<br>**Comparator:** No comparator	**Severity:** mild to moderate<br>**Age:** children, mean age 7.9 years			No adverse events reported

	Risk of bias not assessed in any of the included systematic reviews.		**Sample size:** 10 participants			
Crespi 1986 [39]<br>***(Callen 2007 [24])***	Single arm study (observational)<br>*(4 weeks treatment)*<br>*Risk of bias not assessed in any of the included systematic reviews.*	**Intervention:** Alclometasone cream, twice daily (n=39)<br>**Comparator:** No comparator	**Severity:** not specified in the review<br>**Age:** children<br>**Sample size:** 39 participants		**HPA axis suppression**<br>All normal (measured via morning cortisol)	
**Mild potency topical corticosteroids**						
Udompataikul 2011 [40]<br>***(Van Zuuren 2017 [12])***	Within-participant RCT<br>*(6 weeks treatment)*<br>*(Cochrane risk of bias tool: unclear risk of selection, performance and attrition bias. Low risk of reporting and other biases. High risk of detection bias. [12])*<br>*(Cochrane risk of bias tool: unclear risk of selection bias from sequence generation and risk from blinding. Low risk of selection bias from allocation concealment, [3])*	**Intervention:** Hydrocortisone acetate 1% cream twice daily, was applied one side of the body for 4 weeks followed by the cream base for 2 weeks.<br>**Comparator:** Licochalcone (containing *Glycyrrhiza inflata* root extract, decanediol, menthoxypropanediol and 6-fatty acids) applied twice daily on one side of the body for 6 weeks	**Severity:** mild to moderate<br>**Age:** children 2 months to 10 years (mean age 5.8 years)<br>**Sample size:** 30 participants			No adverse events on either side during the study.
Hebert 2007 [41]<br>***(Nankervis 2017 [3], Fishbein 2019 [16])***	RCT<br>*(28 days)*<br>*Risk of bias not assessed in any of the included systematic reviews.*	**Intervention:** Desonide 0.05% gel twice daily (n=425)<br>**Comparator:** Hydrogel vehicle twice daily (n=157)	**Severity:** mild to moderate<br>**Age:** children 3 months to 18 years<br>**Sample size:** 582 children			**Serious adverse events**<br>One event reported in TCS group but not thought to be related to treatment<br>**Withdrawal due to adverse events**<br>TCS group: 4 in total from this study and from Eichenfield 2006<br>**The number of participants reporting at least 1 adverse event**<br>**Desonide:** 85/425 (20 %) participants<br>**Vehicle:** 46/157 (29.3%)

						participants<br>*(Difference between groups: p=0.02 [a])*
Udompataikul 2012 [42]<br><br>***(Fishbein 2019 [16])***	Within-participant RCT<br>*(4 weeks treatment)*<br>*Risk of bias not assessed in any of the included systematic reviews.*	**Intervention:** Hydrocortisone 1% ointment twice daily, applied to one arm.<br>**Comparator:** 5% dexapanthenol ointment twice daily, applied to the other arm at the same time.	**Severity:** mild to moderate<br>**Age:** children 2 years to 15 years (mean age 7.2 years)<br>**Sample size:** 30 participants			No adverse events on either side during the study.
Wananukul 2013 [43]<br>***(Van Zuuren 2017 [12])***	Within-participant RCT<br>*(4 weeks treatment)*<br>*(Cochrane risk of bias tool: low risk of selection bias from sequence generation, performance, detection, attrition, reporting and other biases. Unclear risk of selection bias from allocation concealment. [12])*	**Intervention:** Hydrocortisone acetate 1% cream twice daily on one side of the body<br>**Comparator:** Licochalcone (containing *Glycyrrhiza inflata* root extract, decanediol, menthoxypropanediol and 6-fatty acids) twice daily on one side of the body	**Severity:** mild to moderate<br>**Age:** children, mean age 3.1 years<br>**Sample size:** 55 participants			No adverse events on either side during the study
Jirabundansuk 2014 [44]<br>***(Van Zuuren 2017 [12])***	Within-participant RCT<br>*(4 weeks treatment)*<br>*(Cochrane risk of bias tool: Unclear risk of selection and performance bias. High risk of detection bias. Low risk of attrition, reporting and other biases. [12])*	**Intervention:** Hydrocortisone acetate 1% cream twice daily on one side of the body<br>**Comparator:** Moisturiser containing spent grain, Vitellaria paradoxa (formerly Butyrospermum parkii) extract plus Argania spinosa kernel oil twice daily on one side of the body	**Severity:** Mild or moderate<br>**Age:** children 2-15 years (mean age 4.3 years)<br>**Sample size:** 31 participants			The investigators stated that "no specific adverse events were reported".
Dolle 2010 [45]<br>***(Nankervis 2017 [3])***	Within-participant RCT<br>*(3 weeks treatment)*<br>*(Cochrane risk of bias tool: unclear risk of selection bias and risk from blinding. [3])*	**Intervention:** 1% hydrocortisone solution once daily for 1st week then twice daily up to 3 weeks<br>**Comparator:** 6% miltefosine solution once daily for 1st week then twice daily up to 3 weeks	**Severity:** moderate to severe<br>**Age:** adults (≥18 years old)<br>**Sample size:** 16 participants	**Local topical adverse events related to the treatment**<br>**Hydrocortisone:** 7/16 participants (44%)<br>**Emollient:** 10/16 participants (63%)<br>These adverse events included pruritus, burning, tingling and dry	No systemic adverse events	No withdrawals because of adverse events

				skin. Dry skin was seen only with emollient treatment.		
Patzelt-Wenczler 2000 [46]<br><br>***(Nankervis 2017*** [3]***)***	Within-participant RCT<br><br>*(2 weeks treatment)*<br><br>*(Cochrane risk of bias tool: unclear risk of selection bias and high risk from no blinding.* [3]*)*	**Intervention:** Hydrocortisone 0.5% twice daily<br><br>**Comparator:** Kamillosan® cream, containing 2% ethanolic extract of chamomile flowers, twice daily (emollient)<br><br>Comparator: Vehicle cream applied twice daily	**Severity:** at least moderate<br><br>**Age:** not specified in the review<br><br>**Sample size:** 72 participants			Three participants in the emollient group withdrew early because of intolerability.
Paller 2003 [47]<br><br>***(Nankervis 2017*** [3]***)***	RCT<br><br>*(2 weeks treatment)*<br><br>*Risk of bias not assessed in any of the included systematic reviews.*	**Intervention:** Fluocinolone acetonide 0.01% twice daily (n=45)<br><br>**Comparator:** Vehicle twice daily (n=49)	**Severity:** not specified in the review<br><br>**Age:** children from 2 to 12 years old<br><br>**Sample size:** 94 participants	**Mild hypopigmentation**<br>Two participants out of 45 reported this event with fluocinolone (4.4%)		
Patel 1995 [48]<br><br>***(Callen 2007*** [24]***)***	Single arm study (observational)<br><br>*(3-10 years follow up)*<br><br>*Risk of bias not assessed in any of the included systematic reviews.*	**Intervention:** 1% Hydrocortisone ointment (n=14; 9/14 intermittently used moderate to high potency)<br><br>**Comparator:** No comparator	**Severity:** not specified in the review<br><br>**Age:** children 3.1 to 10.7 years<br><br>**Sample size:** 14 participants		**HPA axis suppression**<br>Plasma cortisol levels - no change in basal/peak levels but peaked earlier	
Dohil 2009 [49]<br><br>(Wood Heickman 2018 [9])	Single arm study (observational)<br><br>(4 weeks duration)<br><br>*Risk of bias not assessed in any of the included systematic reviews.*	**Intervention:** fluocinolone acetonide 0.01%<br><br>**Comparator:** No comparator	**Severity:** not specified in the review<br><br>**Age:** children (median or mean age 1.1 years)<br><br>**Sample size:** 24 participants		**HPA axis suppression**<br>No cases of adrenal insufficiency *(measured using ACTH stimulation testing, measuring serum cortisol levels)*	
Eichenfield 2007 [50]<br><br>(Wood Heickman 2018 [9])	Single arm study (observational)<br><br>(4 weeks duration)<br><br>*Risk of bias not assessed in any of the included systematic reviews.*	**Intervention:** Desonide hydrogel 0.05%<br><br>**Comparator:** No comparator	**Severity:** not specified in the review<br><br>**Age:** children (median or mean age 3.3 years)		**HPA axis suppression**<br>No cases of adrenal insufficiency *(measured using ACTH stimulation testing, measuring serum cortisol levels)*	

			**Sample size:** 34 participants			
Hebert 2008 [51]<br><br>(Wood Heickman 2018 [9])	Single arm study (observational)<br><br>(4 weeks duration)<br><br>*Risk of bias not assessed in any of the included systematic reviews.*	**Intervention:** Desonide 0.05% foam<br><br>**Comparator:** No comparator	**Severity:** not specified in the review<br><br>**Age:** children (median or mean age 6.7 years)<br><br>**Sample size:** 75 participants		<u>**HPA axis suppression**</u><br>Three out of 75 participants had adrenal insufficiency<br>*(measured using ACTH stimulation testing, measuring serum cortisol levels)*	
**How safe are topical corticosteroids compared to topical calcineurin inhibitors?**						
**Study ID *(Systematic review*)***	**Study design and study duration *(Quality assessment)***	**Intervention and comparator**	**Participants**	**Cutaneous adverse events**	**Systemic adverse events**	**Unspecified adverse events**
**Potent topical corticosteroids**						
Bieber 2007 [52]<br><br>***(Broeders 2016 [53])***	RCT<br><br>*(up to 3 weeks treatment)*<br><br>*(Jadad score 4/5 – risk from sequence generation and allocation concealment [53])*<br><br>*(Cochrane risk of bias tool: unclear risk of selection bias and from blinding. [3])*<br><br>*(Cochrane risk of bias tool: unclear risk of selection bias. Low risk of performance, detection, attrition, reporting and other biases. [54])*	**Intervention:** Methyl-prednisolone 0.1% (n=129) once daily in the evening to all affected body surface areas for a minimum of 2 weeks and a maximum of 3 weeks and cleared areas treated for an additional 7 days post clearance. Also applied a vehicle ointment in the morning to maintain blinding.<br><br>**Comparator:** Tacrolimus 0.03% (n=136), applied twice daily, morning and evening, to all affected body surface areas for a minimum of 2 weeks and a maximum of 3 weeks and cleared areas treated for an additional 7 days post clearance.	**Severity:** severe to very severe<br><br>**Age:** children 2 to 15 years old<br><br>**Sample size:** 265 participants	<u>**Adverse events related to treatment**</u><br>**Methyl-prednisolone:** 0/129 participants (0%)<br>**Tacrolimus:** 6/136 participants (4.4%)<br>*(Difference between groups: p=0.09 [a, b])*		<u>**Severe adverse events**</u><br>**Methyl-prednisolone:** 0/129 participants (0%)<br>**Tacrolimus:** 6/136 participants (4.4%)<br>*(Difference between groups: p=0.09 [a, c])*<br><br><u>**Adverse events requiring discontinuation**</u><br>**Methyl-prednisolone:** 0/129 (0%)<br>**Tacrolimus:** 4/136 (3%)<br>*(Difference between groups: p=0.15 [a])*
Doss 2010 [55]<br><br>***(Broeders 2016 [53])***	RCT<br><br>*(3 weeks treatment twice daily, plus 3 weeks follow up with once daily treatment)*	**Intervention:** Fluticasone 0.005% ointment applied twice daily to all affected areas except eyelids until clearance, up to 3 weeks. All participants who responded to treatment could apply treatment once a day to	**Severity:** moderate to severe<br><br>**Age:** children 2 to 15 years old	<u>**Adverse events related to treatment**</u><br>**Fluticasone:** 45/239 participants (19%)<br>**Tacrolimus:** 55/239 participants (23%)		<u>**Severe adverse events**</u><br>**Fluticasone:** 2/239 participants (0.8%)<br>**Tacrolimus:** 1/239 participants (0.4%)<br>*(Difference between groups: p=0.57 [a])*

	(Jadad score 5/5 – risk from allocation concealment [53])<br><br>*(Cochrane risk of bias tool: unclear risk of selection bias and from blinding. [3])*<br><br>*(Cochrane risk of bias tool: Low risk of selection, performance, detection, attrition, reporting and other biases. [54])*<br><br>*Cochrane risk of bias tool: low risk of selection, performance, attrition, reporting and other biases. Unclear risk of performance bias [56]).*	the remaining lesions for another 3 weeks (n=239)<br><br>**Comparator:** Tacrolimus 0.03% ointment applied twice daily to all affected areas except eyelids until clearance, up to 3 weeks. All participants who responded to treatment could apply treatment once a day to the remaining lesions for another 3 weeks (n=239)	**Sample size:** 478 participants	*(Difference between groups: p=0.26 [a])*<br><br>**Skin burning**<br>**Fluticasone:** 6/239 (2.5%)<br>**Tacrolimus:** 18/237 (7.6%)<br>*(Difference between groups: p=0.02 [a])*<br><br>**Pruritus**<br>**Fluticasone:** 8/239 participants (3.3%)<br>**Tacrolimus:** 10/237 participants (4.2%)<br>*(Difference between groups: p=0.62 [a])*<br><br>**Skin infection**<br><br>**Fluticasone:** 49/239 participants (21%)<br>**Tacrolimus:** 44/239 participants (18%)<br>*(Difference between groups: p=0.56 [a])*		**Adverse events requiring discontinuation**<br>**Fluticasone:** 6/239 participants (2.5%)<br>**Tacrolimus:** 4/239 participants (1.7%)<br>*(Difference between groups: p=0.53 [a])*
Doss 2009 [57]<br><br>***(Broeders 2016 [53])***	RCT<br><br>*(3 weeks of treatment – then for a further 3 weeks either stop treatment, once daily treatment or switch to other treatment twice daily)*<br><br>*(Jadad score 5/5 – risk from allocation concealment [53])*<br><br>*(Cochrane risk of bias tool: unclear risk of selection bias and low risk from blinding. [3])*	**Intervention:** Fluticasone 0.005% ointment twice daily on facial eczema lesions for 3 weeks or until clearance (n=279)<br><br>**Comparator:** Tacrolimus 0.1% twice daily on facial eczema lesions for 3 weeks or until clearance (n=287)<br><br>For 21 days after the initial 3 weeks, the participants could stop treatments if the facial lesions had cleared; stay on the same treatment once a day; or swap treatment using it twice daily (still blinded)	**Severity:** moderate to severe<br><br>**Age:** adults<br><br>**Sample size:** 566 participants	**Adverse events related to treatment**<br>**Fluticasone:** 42/279 participants (15%)<br>**Tacrolimus:** 75/287 participants (26%)<br>*(Difference between groups: p=0.001 [a])*<br><br>**Skin burning**<br>**Fluticasone:** 9/279 participants (3.2%)<br>**Tacrolimus:** 47/287 participants (16.4%)<br>*(Difference between groups: p<0.00001 [a])*<br><br>**Pruritus**<br>**Fluticasone:** 9/279 participants (3.2%)<br>**Tacrolimus:** 12/287 participants (4.2%)<br>*(Difference between groups: p=0.55 [a])*		**Severe adverse events**<br>**Fluticasone:** 0/279 participants (0%)<br>**Tacrolimus:** 1/287 participants (0.3%)<br>*(Difference between groups: p=0.51 [a]).*<br><br>**Adverse events requiring discontinuation**<br>**Fluticasone:** 8/279 participants (2.9%)<br>**Tacrolimus:** 7/287 participants (2.4%)<br>*(Difference between groups: p=0.75 [a])*

Luger 2001 [58]<br><br>***(Broeders 2016 [53])***	RCT<br><br>*(up to 3 weeks treatment)*<br><br>*(Jadad score 3/5 – risk from sequence generation and allocation concealment [53])*<br><br>*(Cochrane risk of bias tool: unclear risk of selection bias and unclear risk from blinding. [3])*<br><br>*(Jadad scale: 3/5 [59])*<br><br>*(Cochrane risk of bias tool: unclear risk of selection bias, adequate blinding, inadequate loss to follow up. [60])*	**Intervention:** Betamethasone valerate 0.1% applied twice daily on all affected areas except for the head and neck for up to 3 weeks or until complete clearance if this was sooner (n=42)<br><br>**Comparator:** Pimecrolimus 1% applied twice daily on all affected areas except for the head and neck for up to 3 weeks or until complete clearance if this was sooner (n=45)	**Severity:** moderate<br><br>**Age:** adults ≥ 18 years old<br><br>**Sample size:** 87 participants	**<u>Pruritus</u>**<br>**Betamethasone:** 5/42 participants (12%)<br>**Pimecrolimus:** 14/45 participants (31%)<br>*(Difference between groups: p=0.04 [a])*<br><br>**<u>Skin burning</u>**<br>**Betamethasone:** 4/42 participants (9.5%)<br>**Pimecrolimus:** 22/45 participants (49%)<br>*(Difference between groups: p=0.001 [a])*		**<u>Adverse events requiring discontinuation</u>**<br>**Betamethasone:** 1/42 participants (2.4%)<br>**Pimecrolimus:** 3/45 participants (6.7%)<br>*(Difference between groups: p=0.36 [a])*
Luger 2004 [61]<br><br>***(Broeders 2016 [53])***	*RCT*<br><br>*(52 weeks. Twice daily until clearance, restarted with flares)*<br><br>*(Jadad score 3/5 – risk from sequence generation and allocation concealment [53])*<br><br>*(Cochrane risk of bias tool: unclear risk of selection bias, low risk from blinding. [3])*<br><br>*(Jadad scale: 3/5 [59])*<br><br>*(Cochrane risk of bias tool: adequate allocation generation, unclear allocation concealment, adequate blinding, inadequate loss to follow up. [60])*	**Intervention:** Triamcinolone 0.1% (potent) and Hydrocortisone acetate 1% (face) (Mild potency) twice daily until complete clearance and itching had stopped, then treatment restarted if inflammation recurred (n=330)<br><br>**Comparator:** Pimecrolimus 1% twice daily until complete clearance and itching had stopped, then treatment restarted if inflammation recurred (n=328)	**Severity:** moderate to severe<br><br>**Age:** adults (age 18 to 79 years)<br><br>**Sample size:** 658 participants	**<u>Skin burning</u>**<br>**Triamcinolone + hydrocortisone:** 36/330 participants (11%)<br>**Pimecrolimus:** 85/328 participants (26%)<br>*(Difference between groups: p<0.00001 [a])*<br><br>**<u>Pruritus</u>**<br>**Triamcinolone + hydrocortisone:** 6/330 participants (1.8%)<br>**Pimecrolimus:** 18/328 participants (5.5%)<br>*(Difference between groups: p=0.02 [a])*<br><br>**<u>Skin thinning</u>**<br>**Triamcinolone + hydrocortisone:** 3/330 participants (0.9%)<br>**Pimecrolimus:** 0/328 participants (0%)<br>*(Difference between groups: p=20 [a])*<br><br>**<u>Skin infection</u>**<br>**Triamcinolone + hydrocortisone:** 80/330 participants (24%)		**<u>Severe adverse events</u>**<br>**Triamcinolone + hydrocortisone:** 21/330 participants (6.4%)<br>**Pimecrolimus:** 16/328 participants (4.9%)<br>*(Difference between groups: p=0.41 [a])*

				**Pimecrolimus:** 69/328 participants (21%)<br>*(Difference between groups: p=0.33 [a])*		
Mandelin 2010 [62]<br><br>***(Broeders 2016 [53])***	RCT<br><br>*(52 weeks, as prescribed until 7 days after clearance, then restarted with flares)*<br><br>*(Jadad score 3/5 – risk from sequence generation and allocation concealment, [53])*<br><br>*(Cochrane risk of bias tool: unclear risk of selection bias, risk from no blinding. [3])*	**Intervention:** Hydrocortisone butyrate 0.1% ointment (potent) and Hydrocortisone acetate 1% ointment (face) (Mild potency) twice daily, as prescribed, for a flare until 7 days after clearance, as many times as required in 1 year (n=40)<br><br>**Comparator:** Tacrolimus 0.1% ointment twice daily, as prescribed, for a flare until 7 days after clearance, as many times as required in 1 year (n=40)	**Severity:** moderate to severe<br><br>**Age:** adults<br><br>**Sample size:** 80 participants	**<u>Skin thinning</u>**<br>**Hydrocortisone:** 2/40 participants (5%)<br>**Tacrolimus:** 0/40 participants (0%)<br>*(Difference between groups: p=0.29 [a])*<br><br>**<u>Skin infection</u>**<br>**Hydrocortisone:** 17/40 participants (43%)<br>**Tacrolimus:** 26/40 participants (65%)<br>*(Difference between groups: p=0.05 [a])*		**Severe adverse events**<br>None in either group

Reitamo 2002 (I) [63]<br><br>***(Broeders 2016 [53]; Iskedjian 2004 [64])***	RCT<br><br>*(3 weeks treatment)*<br><br>*(Jadad score 4/5 – risk from allocation concealment [53])*<br><br>*(Cochrane risk of bias tool: unclear risk of selection bias, unclear risk from blinding. [3])*<br><br>*(Jadad scale: 5/5, [59])*<br><br>*(Cochrane risk of bias tool: Low risk of selection, performance, detection, attrition, reporting and other biases. [54])*<br><br>*(Cochrane risk of bias tool: adequate randomisation and allocation concealment, blinding and ITT analysis done. [65])*	**Intervention:** Hydrocortisone butyrate 0.1% twice daily for 3 weeks (n=186)<br><br>**Comparator:** Tacrolimus 0.1% twice daily for 3 weeks (n=191)<br><br>**Comparator:** Tacrolimus 0.03% twice daily for 3 weeks (arm not included in Broeders 2016 review) (n=193)	**Severity:** moderate to severe<br><br>**Age:** adults (age 16 to 70 years)<br><br>**Sample size: 571** participants	**<u>Skin burning</u>**<br>**Hydrocortisone:** 24/186 participants (13%)<br>**Tacrolimus 0.1%:** 113/191 participants (59%)<br>*(Difference between groups: p<0.00001 [a])*<br><br>**<u>Pruritus</u>**<br>**Hydrocortisone:** 18/186 participants (9.7%)<br>**Tacrolimus 0.1%:** 29/191 participants (15%)<br>*(Difference between groups: p=0.11 [a])*<br><br>**<u>Erythema at application site</u>**<br>**Hydrocortisone:** 1/186 participants (0.5%)<br>**Tacrolimus 0.1%:** 7/191 participants (3.7%)<br>**Tacrolimus 0.03%:** 4/193 participants (2.1%)<br>*(Difference between groups: tacrolimus 0.1% versus hydrocortisone: p=0.07 [a])*<br>(*Difference between groups: tacrolimus 0.03% versus hydrocortisone: p=0.23 [a])*		**<u>Severe adverse events</u>**<br>**Hydrocortisone:** 0/186 participants (0%)<br>**Tacrolimus 0.1%:** 1/191 participants (0.5%)<br>*(Difference between groups: p=0.51 [a])*<br><br>**<u>Adverse events requiring discontinuation</u>**<br>**Hydrocortisone:** 3/186 participants (1.6%)<br>**Tacrolimus 0.1%:** 8/191 participants (4.2%)<br>*(Difference between groups: p=0.15 [a])*

Reitamo 2005 [66]<br><br>***(Broeders 2016*** [53]***)***	RCT<br><br>*(26 weeks)* twice daily treatment until 7 days after clearance, then whenever a flare occurs)<br><br>*(Jadad score 5/5;* [53]*)*<br><br>*(Cochrane risk of bias tool: unclear risk of selection bias,, low risk from blinding.* [3]*)*<br><br>*(Jadad scale: 5/5,* [59]*)*<br><br>*(Cochrane risk of bias tool: High risk of attrition bias. Low risk of selection, performance, detection, reporting and other biases.* [54]*)*<br><br>*(Cochrane risk of bias tool: unclear risk of selection bias, unclear if blinded, and unclear if ITT analysis used.* [65]*)*	**Intervention:** Hydrocortisone butyrate 0.1%(potent) **and** Hydrocortisone acetate 1% (face) (Mild potency) twice daily until 7 days after clearance of eczema each time a flare of eczema occurred for 6 months (n=485)<br><br>**Comparator:** Tacrolimus 0.1% twice daily until 7 days after clearance of eczema each time a flare of eczema occurred for 6 months (n=487)	**Severity:** moderate to severe<br><br>**Age:** adults (≥18 years old)<br><br>**Sample size:** 972 participants	**Adverse events related to treatment**<br>**Hydrocortisone:** 11/485 participants (2.3%)<br>**Tacrolimus:** 7/487 participants (1.4%)<br>*(Difference between groups: p=0.34* [a]*)*<br><br>**Skin burning**<br>**Hydrocortisone:** 67/485 participants (14%)<br>**Tacrolimus:** 255/487 participants (52%)<br>*(Difference between groups: p<0.00001* [a]*)*<br><br>**Pruritus**<br>**Hydrocortisone:** 65/485 participants (13%)<br>**Tacrolimus:** 88/487 participants (18%)<br>*(Difference between groups: p=0.05* [a]*)*<br><br>**Adverse events requiring discontinuation**<br>**Hydrocortisone:** 16/485 participants (3.3%)<br>**Tacrolimus:** 10/487 participants (2%)<br>*(Difference between groups: p=0.23* [a]*)*<br><br>**Skin thinning**<br>**Hydrocortisone:** 2/485 participants (0.4%)<br>**Tacrolimus:** 0/487 participants (0%)<br>*(Difference between groups: p=0.30* [a]*)*<br><br>**Skin infection**<br>**Hydrocortisone:** 9/485 participants (1.9%)<br>**Tacrolimus:** 13/487 participants (2.7%)<br>*(Difference between groups: p=0.40* [a]*)*		**Severe adverse events**<br>**Hydrocortisone:** 9/485 participants (1.9%)<br>**Tacrolimus:** 5/487 participants (1%)<br>*(Difference between groups: p=0.29* [a]*)*<br><br>**Adverse events requiring discontinuation**<br>**Hydrocortisone:** 16/485 participants (3.3%)<br>**Tacrolimus:** 10/487 participants (2.1%)<br>*(Difference between groups: p=0.23* [a]*)*

Gradman 2007 [67]<br><br>***(Svensson 201 [68] 1)***	Crossover RCT<br><br>*(2 weeks treatment)*<br><br>*(Cochrane risk of bias tool: low risk of selection bias from sequence generation, but unclear for allocation concealment. Low risk from blinding.* [3]*)*	**Intervention:** Mometasone furoate 0.1% once daily<br><br>**Comparator:** Tacrolimus 0.1% twice daily	**Severity:** mild to moderate<br><br>**Age:** children 5 to 12 years<br><br>**Sample size:** 20 participants			**Withdrawal from study**<br>**Mometasone:** 1 patient<br>**Tacrolimus**: 1 patient
Kawashima 1997 [69]<br><br>(Ashcroft 2005 [59])	RCT<br><br>*(3 weeks treatment)*<br><br>*(Jadad scale: 5/5,* [59]*)*	**Intervention:** Betamethasone valerate 0.12% twice daily for three weeks (n=89)<br><br>**Comparator:** tacrolimus 0.1% twice daily for three weeks (n=92)	**Severity:** mild to moderate<br><br>**Age:** adults<br><br>**Sample size:** 181 participants	**Skin infections**<br>**Betamethasone:** 5/89 participants<br>**Tacrolimus:** 6/92 participants<br>(*Difference between groups: p=0.80* [a]*)*<br><br>**Skin burning**<br>**Betamethasone:** 3/89 participants<br>**Tacrolimus:** 25/92 participants<br>(*Difference between groups: p=0.0004* [a]*)*		
**Potent or mild potency topical corticosteroids**						
Hofman 2006 [70]<br><br>***(Broeders 2016*** [53]***; Siegfried 2016*** [71]***)***	RCT<br><br>*(2 weeks treatment, 28 weeks follow up)*<br><br>*(Jadad score 5/5 – risk from sequence generation and allocation concealment* [53]*)*	**Intervention:** Hydrocortisone ointment 1% (mild potency) twice daily for head/neck and hydrocortisone butyrate ointment 0.1% (potent) for trunk and limbs for 2 weeks then hydrocortisone 1% (mild potency) twice daily for flares. (n=124)<br><br>**Comparator:** Tacrolimus 0.03% twice daily for 3 weeks then tacrolimus once daily and vehicle once daily for flares (n=133)	**Severity:** moderate to severe<br><br>**Age:** children 2 to 11 years old (mean 6 years old)<br><br>**Sample size:** 257 participants	**Adverse events related to treatment**<br>**Hydrocortisone:** 2/124 participants (1.6%)<br>**Tacrolimus:** 10/133 participants (7.5%)<br>*(Difference between groups: p=0.04* [a]*)*<br><br>**Skin burning**<br>**Hydrocortisone:** 0/124 participants (0%)<br>**Tacrolimus:** 2/133 participants (1.5%)<br>*(Difference between groups: p=0.32* [a]*)*<br><br>**Pruritus**<br>**Hydrocortisone:** 4/124 participants (3%)<br>**Tacrolimus:** 8/133 participants (6%)		**Severe adverse events**<br>**Hydrocortisone:** 0/124 participants (0%)<br>**Tacrolimus:** 2/133 participants (1.5%)<br>(*Difference between groups: p=0.32* [a]*)*

				(Difference between groups: p=0.30 [a])<br>**Skin infection**<br>**Hydrocortisone:** 4/124 participants (3.2%)<br>**Tacrolimus:** 2/133 participants (1.5%)<br>*(Difference between groups: p=0.37 [a])*<br>**Bacterial infection**<br>**Hydrocortisone:** 3/124 participants (2%)<br>**Tacrolimus:** 33/133 participants (2%)<br>*(Difference between groups: p<0.0001)*<br>**Viral infection**<br>**Hydrocortisone:** incidence not reported<br>**Tacrolimus:** 1/133 participants (0.8%)		
**Moderate potency topical corticosteroids**						
Sikder 2005 [72]<br>***(Broeders 2016 [53])***	RCT<br>*(4 weeks treatment)*<br>*(Jadad score 2/5 – risk from sequence generation and allocation concealment, no blinding of observer or patients, [53])*<br>*(Cochrane risk of bias tool: Unclear risk of selection and detection bias. Low risk of performance, attrition, reporting and other biases.[54])*<br>*(Cochrane risk of bias tool: low risk of selection bias, from blinding of participants and missing data. Unclear risk from*	**Intervention:** Clobetasone 0.05% twice daily (n=15)<br>Tacrolimus 0.03% twice daily (n=15)	**Severity:** moderate to severe<br>**Age:** children 7 to 15 years old<br>**Sample size:** 30 participants	**Skin burning**<br>**Clobetasone:** 1/15 participants (6.7%)<br>**Tacrolimus**: 7/15 participants (47%)<br>*(Difference between groups: p=0.05 [a, d])*<br>**Pruritus**<br>**Clobetasone:** 2/15 participants (13%)<br>**Tacrolimus**: 3/15 participants (20%)<br>*(Difference between groups: p=0.63 [a])*		

	blinding outcome assessors, reporting and other biases [56]).					
Torok 2003 [73]<br><br>***(Svensson 2011 [68])***	RCT<br><br>*(3 weeks treatment)*<br><br>*(Delphi list: method of randomisation not described, allocation not concealed, blinded assessors but not participants, ITT analysis, [29])*	**Intervention:** Clocortolone pivalate 0.1% twice daily (n=19)<br><br>**Intervention:** Clocortolone 0.1% + Tacrolimus 0.1% twice daily (n=19)<br><br>**Comparator:** Tacrolimus 0.1% twice daily (n=19)	**Severity:** not specified in the review<br><br>**Age:** adults 16 to 65 years<br><br>**Sample size:** 57 participants	**Skin irritation**<br>Most commonly reported adverse event<br><br>**Skin burning**<br>More frequent in those treated with Tacrolimus 0.1%.<br><br>**Pruritus**<br>Commonly reported in both arms.<br>*(No numerical data provided in the review)*		
**Moderate or mild potency topical corticosteroids**						
Sigurgeirsson 2015 [74]<br><br>***(Broeders 2016 [53]; Siegfried 2016 [71])***	RCT<br><br>*(260 weeks used until clearance or according to country's label. Medication reinstated when a flare occurred))*<br><br>*(Jadad score 3/5 – risk from allocation concealment, no blinding of observer or patients, [53])*	**Intervention:** A moderate potency or mild potency TCS used according to the country's label with potency selected by the investigator (n=1213)<br><br>**Comparator:** Pimecrolimus 1% twice daily (n=1205)	**Severity:** mild to moderate<br><br>**Age:** children age 3 to 12 months old (mean 7 months)<br><br>**Sample size:** 2418 participants	**Skin thinning*(from online correspondence)***<br>**Topical corticosteroid:** 1/1213 participants (0.08%)<br>**Pimecrolimus:** 0/1205 (0%)<br>*(Difference between groups: p=0.50 [a])*<br><br>**Skin infection**<br>**Topical corticosteroid:** 150/1213 participants (12%)<br>**Pimecrolimus:** 157/1205 participants (13%)<br>*(Difference between groups: p=0.62 [a])*<br><br>**Cutaneous bacterial infection**<br>**Topical corticosteroid:** 121/1213 participants (10%)<br>**Pimecrolimus:** 145/1205 participants (12%)<br>*(Difference between groups: p=0.11 [a])*<br><br>**Cutaneous viral infection**<br>**Topical corticosteroid:** 279/1213 participants (23%)<br>**Pimecrolimus:** 301/12 05 participants (25%)	**Systemic bacterial infection**<br>**Topical corticosteroid:** 206/1213 participants (17%)<br>**Pimecrolimus:** 205/1205 participants (17%)<br>*(Difference between groups: p=0.98 [a])*<br><br>**Systemic viral infection**<br>**Topical corticosteroid:** 206/1213 participants (17%)<br>**Pimecrolimus:** 205/1205 participants (17%)<br>*(Difference between groups: p=0.98 [a])*<br><br>**Systemic RTI**<br>**Topical corticosteroid:** 388/1213 participants (32%)<br>**Pimecrolimus:** 422/1205 participants (35%)<br>*(Difference between groups: p=0.11 [a])*<br><br>**Systemic GI**<br>**Topical corticosteroid:** 376/1213 participants (31%)<br>**Pimecrolimus:** 386/1205 participants (32%)	**Severe adverse events**<br>**Topical corticosteroid:** 210/1213 participants (17%)<br>**Pimecrolimus:** 247/1205 participants (20%)<br>*(Difference between groups: p=0.05 [a])*<br><br>**Adverse events requiring discontinuation**<br>**Topical corticosteroid:** 12/1213 participants (1.0%)<br>**Pimecrolimus:** 7/1205 participants (0.6%)<br>*(Difference between groups: p=0.26 [a])*

				(Difference between groups: p=0.25 [a])	*(Difference between groups: p=0.58[a])*<br>**Lymphoma**<br>Zero cases in either group<br>**Growth rate and immune system**<br>No difference between groups	
**Mild potency topical corticosteroids**						
Reitamo 2002 (II) [75] ***(Broeders 2016 [53]; Iskedjian 2004 [64])***	RCT<br>*(3 weeks treatment)*<br>*(Jadad score 5/5 – risk from allocation concealment [53])*<br>*(Cochrane risk of bias tool: unclear risk of selection bias, unclear risk of blinding [3])*<br>*(Jadad scale: 5/5, [59])*<br>*(Cochrane risk of bias tool: Low risk of selection, performance, detection, attrition, reporting and other biases. [54])*<br>*Cochrane risk of bias tool: low risk of selection, performance, attrition, reporting and other bias. Unclear risk from blinding outcome assessors [56]).*<br>*(Cochrane risk of bias tool: adequate method of randomisation and allocation concealment, blinding done, ITT used. [65])*	**Intervention:** Hydrocortisone acetate 1% twice daily (n=185)<br>**Comparator:** Tacrolimus 0.1% ointment twice daily (n=186)<br>**Comparator:** Tacrolimus 0.03% ointment twice daily (arm not included in Broeders 2016 review) (n=189)	**Severity:** moderate to severe<br>**Age:** children 2 to 15 years old<br>**Sample size:** 560participants	**Skin burning**<br>**Hydrocortisone:** 13/185 participants (7%)<br>**Tacrolimus 0.1%:** 38/186 participants (20%)<br>*(Difference between groups: p=0.004 [a])*<br>**Pruritus**<br>**Hydrocortisone:** 14/185 participants (7.6%)<br>**Tacrolimus 0.1%:** 21/186 participants (11%)<br>*(Difference between groups: p=0.22 [a])*<br>**Skin infection**<br>**Hydrocortisone:** 4/185 participants (2.2%)<br>**Tacrolimus 0.1%:** 4/186 participants (2.2%)<br>*(Difference between groups: p=0.99 [a])*<br>**Erythema at application site**<br>**Hydrocortisone:** 3/185 participants (1.6%)<br>**Tacrolimus 0.1%:** 1/186 participants (0.5%)<br>**Tacrolimus 0.03%:** 4/189 participants (2.1%)<br>*(Difference between groups, hydrocortisone vs tacrolimus 0.1%: P=0.34 [a])*<br>*(Difference between groups, hydrocortisone vs tacrolimus 0.03%: P=0.72 [a])*		**Severe adverse events**<br>**Hydrocortisone:** 2/185 participants (1.1%)<br>**Tacrolimus 0.1%:** 1/186 participants (0.5%)<br>*(Difference between groups: (p=0.57 [a])*<br>**Adverse events requiring discontinuation**<br>**Hydrocortisone:** 4/185 participants (2.2%)<br>**Tacrolimus 0.1%:** 3/186 participants (1.6%)<br>*(Difference between groups: p=0.70 [a])*

Reitamo 2004 [76]<br><br>***(Broeders 2016*** **[53])**	RCT<br><br>*(3 weeks treatment)*<br><br>*(Jadad score 3/5 – risk from sequence generation and allocation concealment [53])*<br><br>*(Cochrane risk of bias tool: unclear risk of selection bias. Unclear risk from blinding. [3])*<br><br>*(Jadad scale: 4/5, [59])*<br><br>*(Cochrane risk of bias tool: Unclear risk of selection bias (allocation concealment). Low risk of selection bias (random sequence generation), performance, detection, attrition, reporting and other biases. [54])*<br><br>*Cochrane risk of bias tool: low risk of selection, performance, attrition, reporting and other bias. Unclear risk from blinding outcome assessors [56]).*	**Intervention:** Hydrocortisone acetate 1% twice daily (n=207)<br><br>**Comparator:** Tacrolimus 0.03% twice daily (n=210)	**Severity:** moderate to severe<br><br>**Age:** children 2 to 15 years old<br><br>**Sample size:** 417 participants	**<u>Skin burning</u>**<br>**Hydrocortisone:** 30/207 participants (15%)<br>**Tacrolimus:** 50/210 participants (24%)<br>*(Difference between groups: p=0.02* [a]*)*<br><br>**<u>Pruritus</u>**<br>**Hydrocortisone:** 33/207 participants (16%)<br>**Tacrolimus:** 45/210 participants (21%)<br>*(Difference between groups: p=0.15* [a]*)*<br><br>**<u>Skin infection</u>**<br>**Hydrocortisone:** 6/207 participants (2.9%)<br>**Tacrolimus:** 6/210 participants (2.9%)<br>*(Difference between groups: p=0.98* [a]*)*		**<u>Severe adverse events</u>**<br>**Hydrocortisone:** 3/207 participants (1.4%)<br>**Tacrolimus:** 3/210 participants (1.4%)<br>*(Difference between groups: p=0.99* [a]*)*<br><br>**<u>Adverse events requiring discontinuation</u>**<br>**Hydrocortisone:** 6/207 participants (2.9%)<br>**Tacrolimus:** 8/210 participants (3.8%)<br>*(Difference between groups: p=0.61* [a]*)*
**Potency of topical corticosteroids unknown**						
Gutgesell 1998 [77]<br><br>(abstract only)<br><br>***(Penaloza Hidalgo 2004*** **[65])**	Within-participant RCT<br><br>*(3 weeks treatment)*<br><br>*(Cochrane risk of bias tool: randomisation and allocation concealment method inadequate, unclear if blinded ,ITT analysis used [65])*	**Intervention:** Topical corticosteroids on one side of the body, twice daily<br><br>**Comparator:** Tacrolimus 0.1% on one side of the body, twice daily	**Severity:** severe<br><br>**Age:** adults (22 to 36 years)<br><br>**Sample size:** 7 participants	**<u>Skin burning</u>**<br>**Topical corticosteroids:** 0/7 (0%)<br>**Tacrolimus:** 2/7 participants (29%)		
Arellano 2007 [78]<br><br>***(Ashcroft 2007*** **[60]*;*** Cury Martins 2015 [54]	Nested case-control<br><br>*(Duration not specified in the review)*	**Intervention:** Topical corticosteroids at different potencies<br><br>**Comparator:** pimecrolimus or tacrolimus	**Severity:** not specified in the review<br><br>**Age:** not specified in the review		**<u>Lymphoma</u>**<br>No increased risk of lymphoma with TCI or TCS when compared against controls.<br>***Super potent TCS:*** OR 1.2, 95% CI 0.8 to 1.8	

	Risk of bias not assessed in any of the included systematic reviews.	**Comparator:** controls (not specified in the review)	**Sample size:** 294 cases/293,000 controls		***Low potency TCS:*** *OR* 1.1, 95%CI 0.7 to 1.6<br>***Pimecrolimus:*** OR 0.8, 95%CI 0.4 to 1.6<br>***Tacrolimus*** OR 0.8, 95% CI 0.4 to 1.7	
Arellano 2009 (79)<br><br>(Cury Martins 2015 (54))	Cohort<br><br>*(followed up between 1992 to 2006)*<br><br>*Risk of bias not assessed in any of the included systematic reviews.*	**Intervention:** Topical corticosteroids at different potencies<br><br>**Comparator:** pimecrolimus or tacrolimus	**Severity:** not specified in the review<br><br>**Age:** not specified in the review<br><br>**Sample size:** > 3,000,000		**Lymphoma**<br>Increased risk with topical corticosteroids (related to potency) but no numerical data given. Insufficient data to assess TCI-related risks.	
Schneeweiss 2009 (80)<br><br>***(Cury Martins 2015 (54))***	Cohort<br><br>*(followed up between the years of 2002 to 2006)*<br><br>*Risk of bias not assessed in any of the included systematic reviews.*	**Intervention: mid to potent t**opical corticosteroids (n=1,043,025)<br><br>**Comparator:** pimecrolimus (n=118,863) or tacrolimus (n=38,757)<br><br>(also a comparison with untreated dermatitis (n=118,825) and general population (n=118,863) .)	**Severity:** not specified<br><br>**Age:** median 1.3 years<br><br>**Sample size:** 1,438,333 participants		**Lymphoma**<br>Very small non-significant increased risk in TCI and TCS patients when compared with the general population, but with similar risks between the treatment groups	
Reitamo 2000 (81)<br><br>***(Cury Martins 2015 (54))***	Open label, single group<br><br>*(6 to 12 months of treatment)*<br><br>*Risk of bias not assessed in any of the included systematic reviews.*	**Intervention:** No steroids (except prior to treatment)<br><br>**Comparator:** Tacrolimus 0.1%	**Severity:** not specified in the review<br><br>**Age:** adults<br><br>**Sample size:** 316 participants	**Skin thinning**<br>One participant had skin thinning when using TCS prior to treatment with tacrolimus – but this ameliorated after 6 months of treatment with tacrolimus.		
**Is there any difference in safety of topical corticosteroids of different potencies?**						
**Study ID *(Systematic review*)***	**Study design and study duration *(Quality assessment)***	**Intervention and comparator**	**Participants**	**Cutaneous adverse events**	**Systemic adverse events**	**Unspecified adverse events**
**Potent topical corticosteroid versus moderate potency topical corticosteroid**						
Ulrich 1991 (82)<br><br>***(Hoare 2000 (19))***	RCT<br><br>*(2 weeks treatment)*<br><br>*(Moher 1995 quality checklist: method and*	**Intervention:** 0.05% halomethasone cream, twice daily (Assume potent)	**Severity:** not specified in the review			No adverse events

	concealment of randomisation unclear, concerns over subgroup analysis [19])	**Comparator:** 0.25% prednicarbate cream, twice daily (moderate potency)	**Age:** not specified in the review<br>**Sample size:** 165 participants			
Smitt 1993 [83]<br>***(Callen 2007 [24])***	RCT<br>*(3 weeks treatment)*<br>*Risk of bias not assessed in any of the included systematic reviews.*	**Intervention:** Trimaconiolone acetonide 0.1%, twice daily (potent)<br>**Comparator:** Alclomethasone cream, twice daily (moderate)	**Severity:** not specified in the review<br>**Age:** children 1 to 15 years<br>**Sample size:** 40 participants		**<u>HPA axis suppression</u>**<br>There was suppression after 2 weeks, but no further after 3 (no further details).	
**Potent topical corticosteroid versus mild potency topical corticosteroid**						
Lebrun-Vignes 2000 [84]<br>***(Nankervis 2017 [3])***	RCT<br>*(15 days treatment, 30 days follow up)*<br>*(Cochrane risk of bias tool: unclear risk of selection bias and unclear risk from blinding. [3])*	**Intervention:** Micronized desonide cream 0.1% (mild potency) 1 to 5 days twice daily (in hospital), days 6 and 7 once daily, then alternate days until day 15 (n=15)<br>**Comparator:** Betamethasone dipropionate cream 0.05% (potent) 1 to 5 days twice daily (in hospital), days 6 and 7 once daily, then alternate days until day 15 (n=14)	**Severity:** severe<br>**Age:** children ≤ 8 years<br>**Sample size:** 29 participants			There were no adverse events in either group
Prado de Oliveira 2002 [85]<br>***(Nankervis 2017 [3])***	RCT<br>*(Up to 42 days treatment)*<br>*(Cochrane risk of bias tool: unclear risk of selection bias and unclear risk from blinding. [3])*	**Intervention:** Mometasone furoate 0.1% once daily after a bath (N=13) (potent)<br>**Comparator:** Desonide cream 0.05% once a day after a bath (N=12) (mild potency)	**Severity:** not specified in the review<br>**Age:** children 2 to 12 years<br>**Sample size:** 25 participants	**<u>Signs of mild thinning</u>**<br>**Mometasone furoate:** 4/13 participants (31%)<br>**Desonide:** 2/12 participants (17%)<br>*(Difference between groups: p=0.42 [a])*		
Hanifin 1996 [86]<br>***(Callen 2007 [24])***	Matched case control<br>*(3 weeks treatment)*<br>*Risk of bias not assessed in any of the included systematic reviews.*	**Intervention:** Mometasone cream (potent)<br>**Comparator:** Hydrocortisone cream (mild potency)	**Severity:** not specified in the review<br>**Age:** children 6 months to 2 years<br>**Sample size:** 62 participants		**<u>HPA axis suppression</u>**<br>**Mometasone:** 1 abnormal cotrosyn simulation test	

Kirkup 2003 [87] (trial a)<br>***(Tang 2014* [88]*; Siegfried 2016* [71]*)***<br>Most safety data presented was combined with Kirkup 2003 (trial b) (see same potency section below)	RCT<br>*(16 weeks: twice daily for 2-4 weeks until stabilised then 'as required' for 12 weeks)*<br>*(Cochrane risk of bias tool: unclear risk of selection bias, unclear risk from blinding.* [3]*)*	**Intervention:** Fluticasone propionate 0.005% ointment (potent), twice daily for 2-4 weeks until stabilised then 'as required' for 12 weeks (n=70)<br>**Comparator:** Hydrocortisone 1% cream (mild potency), twice daily for 2-4 weeks until stabilised then 'as required' for 12 weeks (n=67)	**Severity: moderate**<br>**Age:** children (age 2-14 years old)<br>**Sample size (maintenance phase):** 137 participants	**Ringworm and folliculitis**<br>1 participant but not clear which group<br>**Kirkup 2003a and b: Bacterial infection**<br>**Fluticasone:** 1/136 participants (0.7%)<br>**Hydrocortisone:** 3/129 participants (2%)<br>*(Difference between groups: p = 0.32* [a]*)*<br>**Kirkup 2003a and b: Fungal infection**<br>**Fluticasone:** 1/136 participants (0.7%)<br>**Hydrocortisone:** 0/129 (0%)<br>*(Difference between groups: p = 0.52* [a]*)*	**Kirkup 2003a and b viral infection**<br>**Fluticasone:** 5/136 participants (4%)<br>**Hydrocortisone:** 5/129 participants (4%)<br>*(Difference between groups: p = 0.93* [a]*)*<br>**Kirkup 2003a and b: Respiratory tract infection**<br>**Fluticasone:** 8/136 participants (6%)<br>**Hydrocortisone**: 5/129 participants (4%)<br>*(Difference between groups: p = 0.45* [a]*)*	**Kirkup 2003a and b: Discontinuation due to adverse events**<br>**Fluticasone:** 1/136 participants (0.7%)<br>**Hydrocortisone:** 1/129 participants (0.7%)<br>*(Difference between groups: p = 0.97* [a]*)*
**Moderate potency topical corticosteroid versus mild potency topical corticosteroid**						
Kuokkanen 1987 [89]<br>***(Hoare 2000* [19]*)***	RCT, within participant<br>*(3 weeks treatment)*<br>*(Moher 1995 quality checklist: method and concealment of randomisation unclear, double blinded study, three dropouts/ withdrawals, no ITT,* [19]*)*	**Intervention:** Alclometasone dipropionate 0.05% twice daily (moderate potency)<br>**Comparator:** Hydrocortisone 1% twice daily (mild potency)	**Severity:** not specified in the review<br>**Age:** children<br>**Sample size:** 37 participants	No evidence of skin thinning		
**Various potencies**						
Ellison 2000 [90]<br>***(Callen 2007* [24]*; Eichenfield 2014* [91]*)***	Observational study<br>*(Duration 0.7 to 18.7 years)*<br>*Risk of bias not assessed in any of the included systematic reviews.*	**Intervention:** Mild, moderate, potent topical corticosteroids	**Severity:** Disease severity score 5-8<br>**Age:** children and adolescents (0.7 to 18.7 years)<br>**Sample size:** 35 participants		**HPA axis suppression**<br>**Mild potency topical corticosteroids:** no change in plasma cortisol levels<br>**Potent topical corticosteroids:** suppression in 4/4 (100%) patients	

Kristmundsdottir 1987 [92]<br>***(Eichenfield 2014 [91])***	Observational study<br>*(Duration not specified in the review)*<br>*Risk of bias not assessed in any of the included systematic reviews.*	**Intervention:** Different potencies of topical corticosteroids	**Severity:** not specified in the review<br>**Age:** not specified in the review<br>**Sample size:** not specified in the review		Review authors reported "Also concerns for negative effects on linear growth, although reports have given mixed conclusions"	
Patel 1997 [93]<br>***(Eichenfield 2014 [91])***	Observational study<br>*(Duration not specified in the review)*<br>*Risk of bias not assessed in any of the included systematic reviews.*	**Intervention:** Four different potency topical corticosteroids	**Severity:** not specified in the review<br>**Age:** not specified in the review<br>**Sample size:** not specified in the review		Review authors reported "Also concerns for negative effects on linear growth, although reports have given mixed conclusions"	
Patel 1998 [94]<br>***(Eichenfield 2014 [91])***	Observational study<br>*(Duration not specified in the review)*<br>*Risk of bias not assessed in any of the included systematic reviews.*	**Intervention:** Different potencies of topical corticosteroids	**Severity:** not specified in the review<br>**Age:** not specified in the review<br>**Sample size:** not specified in the review		Review authors reported "Also concerns for negative effects on linear growth, although reports have given mixed conclusions"	
**Is there any difference in safety between topical corticosteroids of the same potency?**						
**Study ID *(Systematic review*)***	**Study design and study duration *(Quality assessment)***	**Intervention and comparator**	**Participants**	**Cutaneous adverse events**	**Systemic adverse events**	**Unspecified adverse events**
**Potent topical corticosteroid versus another potent topical corticosteroid**						
Kirkup 2003 [87] (trial b)<br>***(Tang 2014 [88]; Siegfried 2016 [71])***<br>Most safety data presented was combined with Kirkup 2003	RCT<br>*(16 weeks: twice daily for 2-4 weeks until stabilised then intermittently for 12 weeks)*<br>*(Cochrane risk of bias tool: unclear risk of selection bias, unclear risk from blinding. [3])*	**Intervention:** Fluticasone propionate 0.005% ointment, twice daily for 2-4 weeks until stabilised then intermittently for 12 weeks (n=66)<br>**Comparator:** Hydrocortisone butyrate 0.1% cream (potent), twice daily for 2-4 weeks until	**Severity:** moderate<br>**Age:** children (age 2-14 years old)<br>**Sample size:** n=128	**Ringworm and folliculitis**<br>None reported<br>**Kirkup 2003a and b: Bacterial infection**<br>**Fluticasone:** 1/136 participants (0.7%)<br>**Hydrocortisone:** 3/129 participants (2%)	**Kirkup 2003a and b viral infection**<br>**Fluticasone:** 5/136 participants (4%)<br>**Hydrocortisone:** 5/129 participants (4%)<br>*(Difference between groups: p = 0.93 [a])*<br>**Kirkup 2003a and b: Respiratory tract infection**	**Kirkup 2003a and b: Discontinuation due to adverse events**<br>**Fluticasone:** 1/136 participants (0.7%)<br>**Hydrocortisone:** 1/129 participants (0.7%)<br>*(Difference between groups: p = 0.97 [a])*

(trial a) (see different potency section above)		stabilised then intermittently for 12 weeks (n=62)		*(Difference between groups: p = 0.32 [a])*<br>**Kirkup 2003a and b: Fungal infection**<br>**Fluticasone:** 1/136 participants (0.7%)<br>**Hydrocortisone:** 0/129 (0%)<br>*(Difference between groups: p = 0.52 [a])*	**Fluticasone:** 8/136 participants (6%)<br>**Hydrocortisone**: 5/129 participants (4%)<br>*(Difference between groups: p = 0.45 [a])*	
**Moderate potency topical corticosteroid versus another moderate potency topical corticosteroid**						
Aliaga 1994 [95]<br>***(De Tiedra 1997 [33])***	RCT<br>*(21 days treatment)*<br>*Risk of bias not assessed in any of the included systematic reviews.*	**Intervention:** Prednicarbate ointment 0.25%, twice daily (moderate potency) (n=36)<br>**Comparator:** Flucortin ointment 0.75%, twice daily (assumed moderate potency) (n=31)	**Severity:** Disease duration – mean 7.7 years (range 0.1 to 31).<br>**Age:** adults 18-74 years (mean 33.6)<br>**Sample size:** 67 participants			**Adverse reactions**<br>**Prednicarbate:** 0/36 patients (0%)<br>**Flucortin:** 2/31 patients (6.5%)<br>*(Difference between groups: p = 0.16 [a])*
**Mild potency topical corticosteroid versus another mild potency topical corticosteroid**						
Lucky 1997 [96]<br>***(Callen 2007 [24]; Hoare 2000 [19]; Wood Heickman 2018 [9])***	RCT<br>*(4 weeks treatment)*<br>*(Moher 1995 quality checklist: method and concealment of randomisation unclear, open label, five dropouts, no ITT [19])*	**Intervention:** Desonide 0.05% ointment, twice per day (mild potency)<br>**Comparator:** Hydrocortisone 2.5% ointment, twice per day (mild potency)	**Severity:** not specified in the review<br>**Age:** children (mean or median is 4.7 years)<br>**Sample size:** 20 participants		**HPA axis suppression**<br>Normal in both groups (measured using ACTH stimulation testing, measuring serum cortisol levels)	
Jorizzo 1995 [97]<br>***(Siegfried 2016 [71]; Froeschl 2007 [98])***	RCT<br>*(25 weeks: 5 weeks of treatment, 20 weeks follow up)*<br>*(Moher 1995 quality checklist: method and concealment of randomisation unclear, investigator blind, two dropouts/withdrawals, no ITT [19])*	**Intervention:** 0.05% desonide twice daily (n=16) (mild potency)<br>**Comparator:** 1% hydrocortisone ointment twice daily (n=20) (mild potency)	**Severity:** mild to moderate<br>**Age:** children 5 years and under<br>**Sample size:** 36 participants	**Skin thinning**<br>No cases - measured by a magnifying lamp		

How safe are topical corticosteroids compared to Chinese herbal medicine?						
**Study ID *(Systematic review*)***	**Study design and study duration *(Quality assessment)***	**Intervention and comparator**	**Participants**	**Cutaneous adverse events**	**Systemic adverse events**	**Unspecified adverse events**
**Very potent topical corticosteroids**						
Huang 2010 [99]<br>***(Gu 2013 [100]; Gu 2014 [101])***	RCT<br>*(2 weeks treatment, followed up for 12 weeks after)*<br>*(Cochrane risk of bias tool: low risk of selection bias (random sequence generation), and other biases. Unclear risk of selection (allocation concealment), detection and attrition bias. High risk of performance and reporting bias. [100, 101]*	**Intervention:** Clobetasol propionate ointment, 3 times daily (n=97)<br>**Comparator:** Chushi Zhiyang ointment, 3 times daily (n=98)	**Severity:** not specified in the review<br>**Age:** children and adults, 3 months to 22 years<br>**Sample size:** 195 participants	**<u>Cutaneous adverse events</u>**<br>**Clobetasol:** 5/97 participants (5%)<br>**Chinese herbal medicine:** 0/98 participants (0%)<br>*(Difference between groups: p=0.10 [a, e])*<br>The five events were pigmentation (unclear if hyper- or hypo-)		
**Potent topical corticosteroids**						
Chen 2011 [102]<br>***(Gu 2013 [100]; Gu 2014 [101])***	RCT<br>*(2 weeks treatment)*<br>*(Cochrane risk of bias tool: unclear risk of selection, detection, attrition, reporting and other bias. High risk of performance bias [100, 101])*	**Intervention:** Mometasone furoate cream, once daily (n=50)<br>**Comparator:** Huanglian Qingdai ointment, 2 to 3 times daily (n=50)	**Severity:** not specified in the review<br>**Age:** children 58 days to 2 years<br>**Sample size:** 100 participants	**<u>Cutaneous adverse events</u>**<br>**Mometasone:** 6/50 participants (12%)<br>**Chinese herbal medicine:** 0/50 participants (0%)<br>*(Difference between groups: p=0.08 [a, f])*<br>Minor adverse events such as burning, dryness and scaling of the skin were reported in the TCS groups		
Dong 2012 [103]<br>***(Gu 2014 [101])***	RCT<br>*(2 weeks treatment)*<br>*(Cochrane risk of bias tool: unclear risk of selection, detection, attrition, and reporting bias. High risk of*	**Intervention:** Hydrocortisone butyrate cream, twice daily (n=47)<br>**Comparator:** Jingfang mixture solution, twice daily (n=48)	**Severity:** not specified in the review<br>**Age:** children 0.5 to 5.5 years<br>**Sample size:** 95 participants	Minor adverse events such as burning, dryness and scaling of the skin were reported in the TCS groups.<br>*(No numerical data provided in the review)*		

	performance bias. Low risk of other biases. [(101)]*)*					
Xu 2012 [(104)]<br><br>***(Gu 2014*** [(101)]***)***	RCT<br><br>*(2 weeks treatment)*<br><br>*(Cochrane risk of bias tool: unclear risk of selection, detection, attrition, and reporting bias. High risk of performance and other biases.* [(101)]*)*	**Intervention:** Triamcinolone acetonide acetate cream, twice daily (n=51)<br><br>**Comparator:** Kouqiang Xiaoyan powder, twice daily (n=53)	**Severity:** not specified in the review<br><br>**Age:** children 35 days to 2 years<br><br>**Sample size:** 104 participants			No adverse events in either group
**How safe is more frequent topical corticosteroid application compared with once daily application?**						
**Study ID *(Systematic review*)***	**Study design and study duration *(Quality assessment)***	**Intervention and comparator**	**Participants**	**Cutaneous adverse events**	**Systemic adverse events**	**Unspecified adverse events**
**Very potent topical corticosteroid**						
Schlessinger 2006 [(105)]<br><br>***(Nankervis 2017*** [(3)]***; Wood Heickman 2018*** [(9)]***)***	Open label RCT<br><br>*(2 weeks treatment)*<br><br>*(Cochrane risk of bias tool: unclear risk of selection bias, high risk from no blinding.* [(3)]*)*	**Intervention:** fluocinonide cream 0.1% applied once daily (n=63)<br><br>**Comparator:** fluocinonide cream 0.1% applied twice daily (n=63)	**Severity:** not specified in the review<br><br>**Age:** children, aged 12 to <18 years (cohort 1); 6 to <12 years (cohort 2); 2 to <6 years (cohort 3); and 3 months to <2 years (cohort 4).<br><br>**Sample size:** 126 participants		<u>**HPA axis suppression**</u><br>**Once daily**: 0/63 (0%)<br>**Twice daily**: 3/63 (4.8%)<br>*(Difference between groups: P=0.19*[a]*)*<br><br>(measured using ACTH stimulation testing, measuring serum cortisol levels)<br><br>After TCS discontinuation, children with biochemical adrenal insufficiency had complete resolution at retesting.	
**Potent topical corticosteroids**						
Bleehen 1995 [(106)]<br><br>***(Green 2004*** [(107)]***)***	RCT<br><br>*(4 weeks treatment))*<br><br>*(Quality using NHS CRD criteria: method for randomisation/allocation concealment unknown, adequate blinding, and ITT used.* [(107)]*)*<br><br>*(Moher 1995 quality checklist: method and*	**Intervention:** Fluticasone propionate 0.05% cream once daily (plus vehicle once daily for blinding) (n=137)<br><br>**Comparator:** Fluticasone propionate 0.05% cream twice daily (n=133)	**Severity:** at least moderate severity<br><br>**Age:** children and adults<br><br>**Sample size:** 270 participants			<u>**Number of events possibility, probably or almost certainly related to study medication**</u><br>**Once daily:** 26 events<br>**Twice daily**: 24 events<br>(most were skin disorders)

	concealment of randomisation unclear, Probably investigator blinded but unclear, ITT analysis, [(19)])					
GSK report 1995 [(108)]<br><br>***(Green 2004*** [(107)]***)***	RCT<br><br>*(4 weeks treatment)*<br><br>*(Quality using NHS CRD criteria: adequate method of randomisation /allocation concealment, adequate blinding, and ITT used.* [(107)]*)*	**Intervention:** Fluticasone propionate 0.005% ointment once daily and placebo only daily (n=123)<br><br>**Comparator:** Fluticasone propionate 0.005% ointment twice daily (n=122)	**Severity:** at least moderate severity<br><br>**Age:** children and adults<br><br>**Sample size:** 245 participants			**<u>Number of adverse events possibly related to medication</u>**<br>**Once daily:** 6 events<br>**Twice daily**: 8 events<br><br>**<u>Number of adverse events probably related to medication</u>**<br>**Once daily:** 9 events<br>**Twice daily:** 3 events<br><br>**<u>Number of adverse events almost certain related to medication</u>**<br>**Once daily:** 6 events<br>**Twice daily:** 3 events<br><br>(Mainly included skin related disorders including exacerbation of eczema, pruritus and redness of skin)
Koopmans 1995 [(109)]<br><br>***(Green 2004*** [(107)]***)***	RCT<br><br>*(4 weeks treatment))*<br><br>*(Quality using NHS CRD criteria: method for randomisation /allocation concealment unknown, partial blinding, and no ITT used.* [(107)]*)*<br><br>*(Moher 1995 quality checklist: method and concealment of randomisation unclear, double blinded, one dropout, no ITT analysis,* [(19)]*)*	**Intervention:** Locoid lipocream (0.1% hydrocortisone 17-butyrate) once daily and locobase once daily (n=75)<br><br>**Comparator:** Locoid lipocream twice daily (n=75)	**Severity:** not specified in the review<br><br>**Age:** children aged over 12 years and adults<br><br>**Sample size:** 150 participants	**<u>Folliculitis in all skin areas after 1 week of treatment – treatment stopped</u>**<br>**Once daily:** 1/75 participants (1.3%)<br>**Twice daily:** 0/75 participants (0%)<br>*(Difference between groups: p = 0.50* [a]*)*<br><br>**<u>Folliculitis - treatment continued</u>**<br>**Once daily:** 0/75 participants (0%)<br>**Twice daily:** 4/75 participants (5.3%)<br>*(Difference between groups: p = 0.14* [a]*)*<br><br>**<u>Burning, itching and stinging sensations – treatment continued</u>**<br>**Once daily:** 3/75 participants (4%)		

				**Twice daily:** 0/75 participants (0%)<br>*(Difference between groups: p = 0.20 [a])*		
Tharp 1996 [110]<br><br>***(Green 2004*** *[107]* ***)***	RCT<br><br>*(4 weeks treatment)*<br><br>*(Quality using NHS CRD criteria: method for randomisation /allocation concealment unknown, adequate blinding, and no ITT used.* *[107]* *)*	**Intervention:** Fluticasone propionate cream 0.05% once daily and vehicle once daily (n=77)<br><br>**Comparator:** Fluticasone propionate cream 0.05% twice daily (n=77)	**Severity:** moderate to severe<br><br>**Age:** children over 12 years and adults<br><br>**Sample size:** 154 participants	**Burning**<br>**Once daily**: 2/77 participants (3%)<br>**Twice daily:** 0/77 participants (0%)<br>*(Difference between groups: p = 0.30 [a])*<br><br>**Dryness**<br>**Once daily:** 2/77 participants (3%)<br>**Twice daily:** 0/77 participants (0%)<br>*(Difference between groups: p = 0.30 [a])*<br><br>**Pruritus**<br>**Once daily:** 0/77 participants (0%)<br>**Twice daily**: 1/77 participants (1%)<br>*(Difference between groups: p = 0.50 [a])*<br><br>**Erythema**<br>**Once daily:** 0/77 participants (0%)<br>**Twice daily:** 0/77 participants(0%)<br><br>**Stinging**<br>**Once daily:** 0/77 participants (0%)<br>**Twice daily:** 1/77 participants (1%)<br>*(Difference between groups: p = 0.50 [a])*<br><br>**Irritation**<br>**Once daily:** 0/77 participants (0%)<br>**Twice daily:** 1/77 participants (1%)		None of adverse events were serious or unexpected

				(Difference between groups: p = 0.50 [a]*)*		
Hoybye 1991 [111]<br><br>***(Green 2004*** [107]***)***	RCT<br><br>*((3 weeks treatment)*<br><br>*(Quality using NHS CRD criteria: method for randomisation /allocation concealment unknown, partial or inadequate blinding, and no ITT used.* [107]*)*<br><br>*(Moher 1995 quality checklist: method and concealment of randomisation unclear, single blind, ten dropouts/withdrawals, no ITT analysis,* [19]*)*	**Intervention:** Mometasone furoate in fatty cream base (Elocon) once daily (n=49)<br><br>**Comparator:** Hydrocortisone 17-butyrate in fatty cream base (Locoid) twice daily (n=45)	**Severity:** severity score at least 4.5/9<br><br>**Age:** adults (age 18 to 70)<br><br>**Sample size:** 94 participants	**Treatment related side effects**<br>Were only a few and similar in both groups. They included stinging, burning, itching, dryness, acne, folliculitis, and hair growth.<br><br>**Skin thinning**<br>No evidence		
Berth-Jones 2003 [112]<br><br>***(Green 2004*** [107]***)***<br><br>(This study is also included in the "Topical corticosteroids used proactively to prevent flares", as there is a second phase of the study when participants who have gained control of eczema are randomised to proactive treatment with topical corticosteroid or vehicle. This section only	RCT (four arms)<br><br>*(4 weeks treatment)*<br><br>*(Quality using NHS CRD criteria: adequate randomisation /allocation concealment, partial blinding, and ITT used.* [107]*)*	**Intervention:** Fluticasone propionate cream 0.05% once daily<br><br>N=95<br><br>**Intervention:** Fluticasone propionate ointment 0.005% once daily<br><br>N=100<br><br>**Comparator:** Fluticasone propionate cream 0.05% twice daily<br><br>N=91<br><br>**Comparator:** Fluticasone propionate ointment 0.005% twice daily<br><br>N=90	**Severity:** moderate to severe<br><br>**Age:** children and adults (12-65 years)<br><br>**Sample size:** 376 participants	**Telangiectasia**<br>**Once daily cream**: 0/95 participants (0%)<br>**Twice daily cream:** 1/91 participants (1%)<br>*(Difference between groups: p = 0.48* [a]*)*<br><br>**Once daily ointment:** 1/100 participants (1%)<br>**Twice daily ointment:** 0/90 participants (0%)<br>*(Difference between groups: p = 0.54* [a]*)*<br><br>**Striae**<br>**Once daily cream:** 0/95 participants (0%)<br>**Twice daily cream:** 0/91 participants (0%)<br>*(Difference between groups: n/a)*<br><br>**Once daily ointment:** 1/100 participants (1%)<br>**Twice daily ointment**: 0/90 participants (0%)		

includes safety data from the induction of remission phase).				*(Difference between groups: p = 0.54 [a])*<br>For the three events listed above: two of these patients had a previous history of skin changes, and therefore only one report was newly observed (group not specified in the review).		
Marchesi 1994 [113]<br>***(Green 2004*** [107]***)***	RCT<br>*(3 weeks treatment))*<br>*(Quality using NHS CRD criteria: method for randomisation /allocation concealment unknown, partial blinding, and no ITT used.* [107]*)*<br>*(Moher 1995 quality checklist: method and concealment of randomisation unclear, third-party blind evaluator, no dropouts/withdrawals* [19]*)*	**Intervention:** Mometasone furoate ointment 0.1% once daily (n=30)<br>**Comparator:** Betamethasone dipropionate ointment 0.05% twice daily (n=30)	**Severity:** at least moderate severity<br>**Age:** adults<br>**Sample size:** 60 participants	**Telangiectasia of mild severity in last 2 weeks**<br>**Once daily:** 4/30 participants (13.3%)<br>**Twice daily:** 5/30 participants (16.7%)<br>*(Difference between groups: p = 0.72 [a])*<br>**Possible skin thinning (*"Loss of skin marks and reduced elasticity"*)**<br>**Once daily:** 0/30 participants (0%)<br>**Twice daily:** 1/30 participants (3.3%)<br>*(Difference between groups: p = 0.50 [a])*<br>**Local application site reactions**<br>Did not occur	**Systemic reactions**<br>None – all patients checked for blood test and value varied within a very narrow range.	
**Moderate potency topical corticosteroids**						
Richelli 1990 [114]<br>***(Green 2004*** [107]***)***	RCT<br>(one week treatment<br>*(Quality using NHS CRD criteria: method for randomisation /allocation concealment unknown, inadequate blinding, and no ITT used.* [107]*)*<br>*(Moher 1995 quality checklist: method and concealment of*	**Intervention:** Clobetasone 17-butyrate 0.05% lotion once daily at 9pm (n=9)<br>**Comparator:** Clobetasone 17-butyrate 0.05% lotion twice daily at 8am and 3pm (n=13)<br>**Comparator:** Clobetasone 17-butyrate 0.05% lotion twice daily at 3pm and 8pm (n=8)	**Severity:** not specified in the review<br>**Age:** children<br>**Sample size:** 30 participants		**HPA axis suppression**<br>No significant difference in serum cortisol and ACTH levels before and after TCS administration in any of the three groups, or any differences between groups	Adverse effects not reported

Study ID *(Systematic review*)*	Study design and study duration *(Quality assessment)*	Intervention and comparator	Participants	Cutaneous adverse events	Systemic adverse events	Unspecified adverse events
	randomisation unclear, blinding unclear, ITT unclear [19])					
**How safe are topical corticosteroids when used proactively to prevent flares ("weekend therapy")?**						
**Study ID *(Systematic review*)***	**Study design and study duration *(Quality assessment)***	**Intervention and comparator**	**Participants**	**Cutaneous adverse events**	**Systemic adverse events**	**Unspecified adverse events**
**Potent topical corticosteroids versus vehicle**						
Berth-Jones 2003 [112]<br>***(Schmitt 2011 [115]; Tang 2014 [88])***<br>(This study is also included under the comparison "Topical corticosteroids applied once a day compared with more frequent application" – where the induction of remission part of the study is included).	RCT<br>*(16 weeks maintenance)*<br>*(Cochrane risk of bias tool: low risk of selection (sequence generation), attrition and other biases, Unclear risk of selection (allocation concealment), bias from blinding and reporting bias. [115])*	**Intervention:** Fluticasone propionate 0.005% ointment on two consecutive days per week, once daily (n=68)<br>**Intervention:** Fluticasone propionate 0.05% cream on two consecutive days per week, once daily (n=70)<br>**Comparator:** Vehicle cream or ointment (n=84)<br>**Comparator:** Vehicle ointment (n=73)	**Severity:** moderate to severe<br>**Age:** 12 to 65 years<br>**Sample size (maintenance phase):** 295 participants	**Skin thinning**<br>No new visual signs observed in either group during maintenance phase		
Glazenburg 2009 [116]<br>***(Schmitt 2011 [115]; Tang 2014 [88])***	RCT<br>*(16 weeks maintenance)*<br>*(Cochrane risk of bias tool: low risk of selection (sequence generation), and attrition bias. Unclear risk of selection (allocation concealment), bias from blinding, reporting and other biases. [115])*	**Intervention:** Fluticasone propionate 0.005% ointment (two consecutive days per week, once daily) (n=39)<br>**Comparator:** Vehicle (n=36)	**Severity:** moderate to severe<br>**Age:** children 4-10 years<br>**Sample size (maintenance phase):** 75 participants	**Skin thinning**<br>No evidence in either group<br>**Adverse events related to treatment (cutaneous)**<br>**Fluticasone:** 2 events (flexural hyperpigmentation, folliculitis, transient telangiectasia) (n=39)<br>**Vehicle:** 1 event (no further details reported) (n=36)<br>*(Difference between groups: p=0.61[a])*	**Adrenal suppression**<br>No evidence in either group (measured by assessment of urinary overnight cortisol/creatinine ratios)<br>**Cancer**<br>No cases in either group	

Hanifin 2002 [117]<br>***(Schmitt 2011*** [115]***)***<br>***(Fishbein 2019*** [16]***)***	RCT<br>*(20 weeks maintenance)*<br>*(Cochrane risk of bias tool: low risk of attrition bias. Unclear risk of selection, bias from blinding and reporting bias. High risk of other biases (noncompliance)* [115]*)*	**Intervention:** Fluticasone propionate 0.05% cream (once daily 4 days per week for 4 weeks, then once daily 2 days per week for 16 weeks) (n=229)<br>**Comparator:** Vehicle (n=119)	**Severity:** moderate to severe<br>**Age:** children and adults, 3 months to 65 years<br>**Sample size (maintenance phase):** 348 participants	**Adverse events related to treatment**<br>**Fluticasone:** 1/229 (one case of acne) (0.4%)<br>**Vehicle:** 0/119 (0%)<br>*(Difference between groups: p=0.78*[a]*)*<br>**Skin thinning**<br>No evidence (by visual skin assessment)	**Possible adrenal suppression**<br>**Fluticasone:** 2/44* children (4.5%)<br>**Vehicle:** no evidence of adrenal suppression<br>*(measured by cosynthropin stimulation test)*<br>*One participant received 345 days of treatment and had a cortisol stimulation level after treatment of 17 ug/dL (normal was >=18 ug/dL). The other participant was treated for 280 days and had a cortisol stimulation level of 9 ug/dL. No follow up testing.<br>**Cancer**<br>No cases	
Van der Meer 1999 [118]<br>***(Schmitt 2011*** [115]***)***	RCT<br>*(16 weeks maintenance)*<br>*(Cochrane risk of bias tool: low risk of attrition, and other biases. Unclear risk of selection, bias from blinding and reporting bias.* [115]*)*<br>*((Moher 1995 quality checklist: method and concealment of randomisation unclear, double blinded, 17 withdrawals/dropouts, no ITT, only data up to first relapse analysed,* [19]*)*	**Intervention:** Fluticasone propionate 0.005% ointment (2 consecutive days per week, once daily) (n=23)<br>**Comparator:** Vehicle (n=31)	**Severity:** moderate to severe<br>**Age:** children and adults, aged 15-50 years<br>**Sample size (maintenance phase):** 54 participants	**Skin thinning**<br>No evidence	**Adrenal suppression**<br>No change in geometric mean cortisol levels at baseline and end of maintenance<br>**Cancer**<br>No cases	
Peserico 2008 [119]<br>***(Schmitt 2011*** [115]***)***	RCT<br>*(16 weeks maintenance)*<br>*(Cochrane risk of bias tool: high risk of selection bias (sequence generation). Low risk of attrition bias and bias from blinding. Unclear*	**Intervention:** Prednisolone aceponate 0.1% cream (two consecutive days per week, once daily) (n=112)<br>**Comparator:** Vehicle (n=108)	**Severity:** IGA≥ moderate<br>**Age:** children ≥12 years and adults<br>**Sample size (maintenance**	**Skin thinning**<br>No evidence	**Cancer**<br>No cases.	**Adverse events related to treatment**<br>Zero in both groups

	risk of selection (allocation concealment) reporting and other biases. [(115)]*)*		**phase):** 221 participants			
**How safe are topical corticosteroids used under occlusion?**						
**Study ID *(Systematic review*)***	**Study design and study duration *(Quality assessment)***	**Intervention and comparator**	**Participants**	**Cutaneous adverse events**	**Systemic adverse events**	**Unspecified adverse events**
**Very potent topical corticosteroid**						
Volden 1992 [(120)]<br>***(Braham 2010*** [(121)]***)***	Prospective (observational)<br>*(8-18 days treatment)*<br>*Risk of bias not assessed in any of the included systematic reviews.*	**Intervention:** Dry occlusion with clobetasol propionate lotion under dry occlusion (weekly) (n=48)<br>**Comparator:** No comparator	**Severity:** therapy resistant atopic eczema<br>**Age:** adults<br>**Sample size:** 48 participants	**Mild folliculitis**<br>2/48 participants (4%)<br>**Skin thinning**<br>None		
**Potent topical corticosteroids**						
Janmohamed 2014 [(122)]<br>***(Van Zuuren 2017*** [(12)]***)***	RCT<br>*(4 weeks treatment)*<br>*(Cochrane risk of bias tool: low risk of selection (sequence generation), attrition, reporting and other biases. Unclear risk of selection (allocation concealment), performance and detection bias.* [(12)]*)*	**Intervention:** wet wrap therapy with diluted mometasone furoate 0.1% ointment (n=19)<br>**Comparator:** 20% petrolatum in cetomacrogol combined with wet wrap (n=20)	**Severity:** severe<br>**Age:** children 6 months to 10 years (mean age 3.4 years)<br>**Sample size:** 39 participants	**Folliculitis**<br>**Mometasone under wet wrap:** 9/19 (47%)<br>**Emollient under wet wrap:** 2/20 (10%)<br>*(Difference between groups: p = 0.03* [a]*)*<br>**Severe folliculitis**<br>**Mometasone under wet wrap:** 1/19 (5.2%)<br>**Emollient under wet wrap:** 0/20 (0%)<br>*(Difference between groups: p = 0.47*)*<br>**Secondary infected eczema**<br>**Mometasone under wet wrap:** 0/19 (0%)<br>**Emollient under wet wrap**: 2/20 (10%)<br>*(Difference between groups: p= 0.30* [a]*)*<br>**Beginning of decubitus**<br>**Mometasone under wet wrap:** 0/19 (%)		

				**Emollient under wet wrap:** 2/20 (10%)<br>*(Difference between groups: p = 0.30[a])*<br>__Decubitus__<br>**Mometasone under wet wrap:** 2/19 (11%)<br>**Emollient under wet wrap:** 1/20 (5%)<br>*(Difference between groups: p = 0.53 [a])*		
Schnopp 2002 [123]<br>***(Braham 2010 [121])***	RCT, within-participant<br>*(5 days treatment)*<br>*(Cochrane risk of bias tool: unclear risk of selection bias, unclear risk from blinding. [3])*	**Intervention:** wet wrap therapy with mometasone furoate 0.1%, twice daily<br>**Comparator:** wet wrap therapy with vehicle	**Severity:** exacerbated atopic eczema<br>**Age:** children aged 2 to 17 years (mean 7.2 years)<br>**Sample size:** 20 participants	__Clinical skin infections__<br>None in either group		
McGowan 2003 [124]<br>***(Devillers 2006 [125])***	Prospective (observational)<br>*(Up to 14 days treatment)*<br>*Risk of bias not assessed in any of the included systematic reviews.*	**Intervention:** wet wrap therapy with diluted beclomethasone dipropionate, once daily (n=8)<br>**Comparator:** No comparator	**Severity:** not specified in the review<br>**Age:** children age 3.3 to 8.8 years<br>**Sample size:** 8 participants		__Short term growth and bone turnover__<br>No significant differences found between outcomes before and during a median treatment period of 12 weeks (range 2-18).<br>*(assessed safety with knemometry and urinary deoxypyridinoline crosslink excretion and early morning serum cortisol).*	
Wolkerstorfer 2000 [126]<br>***(Braham 2010 [121])***<br>***(Fishbein 2019 [16])***	Prospective, side to side (observational)<br>*(1 week treatment)*<br>*Risk of bias not assessed in any of the included systematic reviews.*	**Intervention:** wet wrap therapy with 10-50% dilution fluticasone propionate 0.05% cream (daily)<br>**Comparator:** emollient (only 2 participants) or no comparator	**Severity:** severe<br>**Age:** children 5 months to 13 years<br>**Sample size:** 18 participants	__URI and/or folliculitis__<br>**Fluticasone:** one third of participants<br>__Furunculosis__<br>**Fluticasone:** one case<br>__Generalized folliculitis__<br>One case in both emollient controls<br>__Skin thinning__<br>No cases	__HPA axis suppression__<br>"Nearly all" had decreased cortisol, 3 children were HPA suppressed (from Braham 2010 review).<br>Two patients having a 9am serum cortisol < 0.2 umol/L (0.09 and 0.03) after treatment for 7 days. Those participants used 957 ug/m^2 and 1125 ug/m^2 of steroid cream. There was no follow up	

					testing (from Fishbein 2019 review).	
Tang 2000 [127]<br>***(Braham 2010 [121])***	Prospective (observational)<br>*("Few days" treatment)*<br>*Risk of bias not assessed in any of the included systematic reviews.*	**Intervention:** wet wrap therapy with 10% dilution mometasone furoate 0.1% (daily for 2 to 3 hours) (n=10)<br>**Comparator:** No comparator	**Severity:** review only reports 'facial eczema flare'<br>**Age:** children (mean 8.4 years)<br>**Sample size:** 10 participants	**Skin thinning**<br>None<br>**Infections**<br>None		
Goodyear 1991 [128]<br>***(Braham 2010 [121])***	Prospective (observational)<br>*(2 to 5 days treatment)*<br>*Risk of bias not assessed in any of the included systematic reviews.*	**Intervention:** wet wrap therapy with 25% dilution betamethasone or hydrocortisone 1%, twice daily (potent or mild potency) (n=30)<br>**Comparator:** No comparator	**Severity:** acute erythrodermic eczema<br>**Age:** children aged 9 months to 2 years (mean 5.5 years)<br>**Sample size:** 30 participants	**Bacterial infections**<br>Some during follow up at home	**HPA axis suppression**<br>Transient low morning cortisol. During the follow up at home some adrenal suppression.	
Mallon 1994 [129]<br>***(Braham 2010 [121])***	Prospective (observational)<br>*(up to 5 days treatment)*<br>*Risk of bias not assessed in any of the included systematic reviews.*	**Intervention:** wet wrap therapy with 10% dilution betamethasone 0.1% cream or hydrocortisone 0.5% cream(daily) (potent or mild potency) (n=21)<br>**Comparator:** No comparator	**Severity:** chronic severe eczema<br>**Age:** children aged 4 months to 10 years (5.1 years)<br>**Sample size:** 21 participants	No infections.		
Devillers 2002 [130]<br>***(Braham 2010 [121])***	Retrospective side to side (observational)<br>*(1 week treatment)*<br>*Risk of bias not assessed in any of the included systematic reviews.*	**Intervention:** wet wrap therapy with **d**iluted fluticasone propionate 0.05% (daily re-wet every 2 to3 hours) (n=26)<br>**Comparator:** No comparator	**Severity:** refractory atopic eczema<br>**Age:** children (mean 3 years), adults (mean 30 years)<br>**Sample size:** 26 participants (14 children, 12 adults)	**Infections**<br>38% (n=10) had localized folliculitis, impetigo, pseudomonas, cellulitis, or purulent conjunctitivitis<br>**Skin thinning**<br>One case of striae in a patient taking inhaled steroids.	**HPA axis suppression**<br>Transient low morning cortisol, 12.5% with HPA suppression	
**Moderate potency topical corticosteroids**						
Foelster-Holst 2006 [131]	Within-participant RCT<br>*(48 to 72 hours treatment)*	**Intervention:** wet wrap therapy with prednicarbate ointment	**Severity:** local SCORAD >10, severe	Zero adverse events in either group. Did not observe severe cutaneous events.	Did not observe systemic events such as growth retardation or HPA suppression – but these	

(Gonzalez-Lopez 2017 [132])	*(Cochrane risk of bias tool: unclear risk of selection and performance bias. High risk of performance bias. Unclear risk of attrition, reporting and other biases. [132])*<br>*(Cochrane risk of bias tool: unclear risk of selection bias, high risk from no blinding. [3])*	**Comparator:** Prednicarbate ointment	**Age:** children and adults, aged 6-63 years<br>**Sample size: 24** participants		events were not actively investigated.	
**Mild potency topical corticosteroid**						
Beattie 2004 [133]<br>***(Gonzalez-Lopez 2017 [132])***	RCT<br>*(2 weeks treatment)*<br>*(Cochrane risk of bias tool: low risk of selection, reporting and other biases. High risk of performance and attrition bias. Unclear risk of detection bias. Gonzalez-Lopez 2017)*<br>*(Cochrane risk of bias tool: low risk of selection bias (sequence generation), unclear risk of selection bias (allocation concealment), unclear risk from blinding. [3])*	**Intervention:** wet wrap therapy with hydrocortisone 1% twice daily then overnight the second week(n=10)<br>**Comparator:** Hydrocortisone 1% twice daily then daily (n=9)	**Severity:** moderate<br>**Age:** children < 5 years<br>**Sample size:** 19 participants	<u>**Cutaneous adverse events**</u><br>**Wet wrap therapy with hydrocortisone:** 2/10 participants (20%) (2 events were folliculitis, one child withdrew)<br>**Hydrocortisone only:** 0/9 participants (0%)<br>*(Difference between groups: (p=0.31 [a])*<br>Did not observe severe cutaneous events.	Did not observe systemic such as growth retardation or HPA suppression – but these events were not actively investigated.	
Hindley 2006 [134]<br>***(Gonzalez-Lopez 2017 [132])***	RCT<br>*(4 weeks – not clear if treatment given for whole 4 weeks)*<br>*(Cochrane risk of bias tool: low risk of selection (random sequence generation) and reporting bias. Unclear risk of selection (allocation concealment),*	**Intervention:** wet wrap therapy with hydrocortisone 1% for 24 hours – could be reduced to 12 hours per day after first week (n=28)<br>**Comparator:** Hydrocortisone 1% twice day (n=22)	**Severity:** SCORAD >15, moderate to severe<br>**Age:** children 3 months to 5 years<br>**Sample size:** 50 participants	<u>**Cutaneous adverse events**</u><br>**Wet wrap therapy with hydrocortisone:** 5/28 participants (18%) (five cases of infected eczema)<br>**Hydrocortisone only:** 0/22 participants (0%)<br>*(Difference between groups: p = 0.14 [a])*	Did not observe systemic events such as growth retardation or HPA suppression – but these events were not actively investigated.	

	detection and other biases. High risk of performance and attrition bias. [(132)])<br><br>*(Cochrane risk of bias tool: unclear risk of selection bias, low risk from blinding. [(3)])*			Did not observe severe cutaneous events.		

Footnotes:

*This column refers to the systematic review in which the safety data was extract from. The trial may have also been included in other systematic reviews, but no additional safety data was reported.

Abbreviations: RCT = randomised controlled trial; TCS = topical corticosteroids; TCI = topical calcineurin inhibitors; HPA = hypothalamic pituitary adrenal, WWT = wet wrap therapy; RR = risk ratio; OR: odds ratios; 95% CI = 95% confidence interval; CHM = Chinese herbal medicine; IPA = Investigator's Global Assessment; BSA = Body Surface Area

[a] P value calculated by review authors using RevMan software.

[b] The P value calculated from Fisher's Exact Test was significant: 0.0298 (but in the overview, this study is included in a meta-analysis)

[c] The P value calculated from Fisher's Exact Test was significant: 0.0298 (but in the overview, this study is included in a meta-analysis)

[d] The P value calculated from Fisher's Exact Test was significant: 0.0352 (but in the overview, this study is included in a meta-analysis)

[e] The P value calculated from Fisher's Exact Test was significant: 0.0289 (but in the overview, this study is included in a meta-analysis)

[f] The P value calculated from Fisher's Exact Test was significant: 0.0267 (but in the overview, this study is included in a meta-analysis)

Where studies include "diluted" topical corticosteroids and we aren't sure how this affects the potency, we have put the topical corticosteroids in the potency classification based on the undiluted version.

The terms skin atrophy and skin thinning were both used in the included reviews – for consistently we have used skin thinning throughout.

1. Breneman D, Fleischer A, Foley V ea, editors. Clobetasol propionate 0.05% is equivalent as lotion or emollient cream in atopic dermatitis. Presented at the World Congress of Dermatology, 2002; and the European Academy of Dermatology and Venereology, 2003; 2003.
2. Feldman S. Relative efficacy and interchangeability ofvarious clobetasol propionate vehicles in the management of steroid-responsive dermatoses. *Curr Ther Res Clin Exp*. 2005;**66**(3):154-71.
3. Nankervis H, Thomas K, Delamere F, Barbarot S, Rogers N, Williams H. Scoping systematic review of treatments for eczema2016 2016/05/None.
4. Kimball AB, Gold MH, Zib B, Davis MW, Phase CPEFF, Group ICS. Clobetasol propionate emulsion formulation foam 0.05%: review of phase II open-label and phase III randomized controlled trials in steroid-responsive dermatoses in adults and adolescents. *J Am Acad Dermatol*. 2008;**59**(3):448-54. e1.
5. Frangos J, Kimball A. Clobetasol propionate emollient formulation foam in the treatment of corticosteroid-responsive dermatoses. *Expert Opin Pharmacother*. 2008;**9**(11):2001-7.
6. Rosso J, Bhambri S. Daily application of fluocinonide 0.1% cream for the treatment of atopic dermatitis. *J Clin Aesthet Dermatol*. 2009;**2**(9):24-32.
7. Barnes L, Kaya G, Rollason V. Topical Corticosteroid-Induced Skin Atrophy: A Comprehensive Review. *Drug Saf*. 2015;**38**(5):493-509.
8. Olux-e (dobetasol propionate) foam pw. Steiffel Corporation, Palo Alto, CA; [Available from: www.olux-e.com.
9. Wood Heickman L, Davallow Ghajar L, Conaway M, Rogol A. Evaluation of Hypothalamic-Pituitary-Adrenal Axis Suppression following Cutaneous Use of Topical Corticosteroids in Children: A Meta-Analysis. *Horm Res Paediatr*. 2018;**89**(6):389-96.
10. Herz G, Yawalkar S, Weirich E. Evaluation of halometasone ointment in the treatment of paediatric patients with chronic eczematous dermatoses. *J Int Med Res*. 1983;**11 Suppl 1**:21-5.
11. Sugarman J, Parish L. Efficacy of a lipid-based barrier repair formulation in moderate-to-severe pediatric atopic dermatitis. *J Drugs Dermatol*. 2009;**8**(12):1106-11.

12. van Zuuren EJ, Fedorowicz Z, Christensen R, Lavrijsen AP, Arents BW. Emollients and moisturisers for eczema. Cochrane Database Syst Rev [Internet]. 2017; (2). Available from: http://onlinelibrary.wiley.com/doi/10.1002/14651858.CD012119.pub2/abstract.
13. Griffiths C, Van Leent E, Gilbert M, Traulsen J, Cipamyflline Study Group. Randomized comparison of the type 4 phosphodiesterase inhibitor cipamfylline cream, cream vehicle and hydrocortisone 17-butyrate cream for the treatment of atopic dermatitis. *Br J Dermatol*. 2002;**147**(2):299-307.
14. Eichenfield L, Miller B, Cutivate Lotion Study Group. Two randomized, double-blind, placebo-controlled studies of fluticasone propionate lotion 0.05% for the treatment of atopic dermatitis in subjects from 3 months of age. *J Am Acad Dermatol*. 2006;**54**(4):715-7.
15. Wu S, Chen X, Liu B, Wu H, Dong L. Efficacy and safety of 15(R/S)-methyl-lipoxin A4 in topical treatment of infantile eczema. *Br J Dermatol*. 2013;**168**(1):172-8.
16. Fishbein AB, Mueller K, Lor J, Smith P, Paller AS, Kaat A. Systematic Review and Meta-analysis Comparing Topical Corticosteroids With Vehicle/Moisturizer in Childhood Atopic Dermatitis. *J Pediatr Nurs*. 2019;**47**:36-43.
17. Pellanda C, Weber M, Bircher A, Surber C. Low-dose triamcinolone acetonide in the phytocosmetic lichtena reduces inflammation in mild to moderate atopic dermatitis. *Dermatology*. 2005;**211**(4):338-40.
18. Lebwohl M. Efficacy and safety of fluticasone propionate ointment, 0.005%, in the treatment of eczema. *Cutis*. 1996;**57**(Suppl 2):62-8.
19. Hoare C, Li Wan Po A, Williams H. Systematic review of treatments for atopic eczema. *Health Technol Assess*. 2000;**4**(37):1-191.
20. Lebwohl M. A comparison of once-daily application of mometasone furoate 0.1% cream compared with twice-daily hydrocortisone valerate 0.2% cream in pediatric atopic dermatitis patients who failed to respond to hydrocortisone. *Int J Dermatol*. 1999;**38**(8):604-6.
21. Abramovits W, Oquendo M. Hydrocortisone butyrate 0.1% cream (proprietary lipid rich cream vehicle) does not significantly suppress hypothalamic-pituitary-adrenal axis and is effective in pediatric patients 3 months and older with extensive atopic dermatitis. *Skinmed*. 2010;**8**(3):150-4.
22. Matheson R, Kempers S, Breneman D, Draelos Z, Johnson CE, Loss R, et al. Hydrocortisone butyrate 0.1% lotion in the treatment of atopic dermatitis in pediatric subjects. *J Drugs Dermatol*. 2008;**7**(3):266-71.
23. Friedlander S, Hebert A, Allen D. Safety of fluticasone propionate cream 0.05% for the treatment of severe and extensive atopic dermatitis in children as young as 3 months. *J Am Acad Dermatol*. 2002;**46**(3):387-93.
24. Callen J, Chamlin S, Eichenfield L, Ellis C, Girardi M, Goldfarb M, et al. A systematic review of the safety of topical therapies for atopic dermatitis. *Br J Dermatol*. 2007;**156**(2):203-21.
25. Eichenfield L, Ellis CN, Fivenson D, Hebert AA, Dromgoole S, Piacquadio D. Evaluation of adrenal suppression of a lipid enhanced, topical emollient cream formulation of hydrocortisone butyrate 0.1% in treating children with atopic dermatitis. *Pediatr Dermatol*. 2007;**24**(1):81-4.
26. Hebert AA, Friedlander SF, Allen DB. Topical fluticasone propionate lotion does not cause HPA axis suppression. *J Pediatr*. 2006;**149**(3):378-82.
27. De Belilovsky C, Roo-Rodriguez E, Baudouin C, Menu F, Chadoutaud B, Msika P. Natural peroxisome proliferator-activated receptor-alpha agonist cream demonstrates similar therapeutic response to topical steroids in atopic dermatitis. *J Dermatolog Treat*. 2011;**22**(6):359-65.
28. Rosenthal A. Clocortolone pivalate: a paired comparison clinical trial of a new topical steroid in eczema/atopic dermatitis. *Cutis*. 1980;**25**(1):96-8.
29. Singh S, Mann B. Clinical utility of clocortolone pivalate for the treatment of corticosteroid-responsive skin disorders: A systematic review. *Clin Cosmet Investig Dermatol*. 2012;**5**:61-8.
30. Binder R. Clinical study of clocortolone pivalate in the treatment of eczema/atopic dermatitis. *Curr Ther Res Clin Exp*. 1977;**21**(6):796-801.
31. Rauschkolb EW, Bender SH, Ebling JK, McCormick GE, Rosenthal AL. Low concentration halcinonide cream in the topical management of atopic dermatitis in pediatric patients. *Cutis*. 1981;**27**(1):105-7.
32. Nolting S GH, Griinder K. Therapie des atopischen ekzems im kindesalter: ergebnisse einer kontrollierten studie mit den topischen kortikosteroidzubereitungen mometason und prednicarbaL *Der Kinderarzt*. 1991;**22**(10):1690-3.
33. de Tiedra A, Mercadal J, Lozano R. Prednicarbate versus fluocortin for inflammatory dermatoses: A cost-effectiveness study. *PharmacoEconomics*. 1997;**12**(2 Pt 1):193-208.

34. Rampini E. Methylprenisolone aceponate (MPA)* - Use and clinical experience in children. *J Dermatolog Treat*. 1992;**3 (SUPPL. 2)**(Suppl 2 CNO - CN-00421335):27-9.
35. Camacho F, García B, Diaz J, Aguirre A, Arnau C, Garcia J, et al. Double-blind intraindividual comparative trial between prednicarbate versus fluocortolone for the treatment of atopic dermatitis [Ensayo comparativo intraindividual y doble ciego entre prednicarbato y f1uocortolona en el tratamiento de la dermatitis atapica]. Actas Dermo-Sifiliograficas [Internet]. 1996; 87:[59-63 pp.]. Available from: http://cochranelibrary-wiley.com/o/cochrane/clcentral/articles/811/CN-00662811/frame.html.
36. Gimenez Camarasa JM SEFC, editor Prednicarbato, un nuevo corticoide tapico, en comparacian con f1uocinolona en el tratamiento de la dermatitis atapica. XXIII Congreso Nacional de la Academia Espanola de Dermatologia y Venereologia; 1994 Jun 16-18; Madrid.
37. Moshang T. Prednicarbate emollient cream 0.1% in pediatric patients with atopic dermatitis. *Cutis*. 2001;**68**(1):63-9.
38. Conde J, Kaur M, Fleischer Jr A, Tusa M, Camacho F, Feldman S. Adherence to clocortolone pivalate cream 0.1% in a pediatric population with atopic dermatitis. *Cutis*. 2008;**81**(5):435-41.
39. Crespi H. Topical corticosteroid therapy for children: alclometasone dipropionate cream 0.05%. *Clin Ther*. 1986;**8**(2):203-10.
40. Udompataikul M, Srisatwaja W. Comparative trial of moisturizer containing licochalcone A vs. hydrocortisone lotion in the treatment of childhood atopic dermatitis: a pilot study. *J Eur Acad Dermatol Venereol*. 2011;**25**(6):660-5.
41. Hebert A, Cook-Bolden F, Basu S, Calvarese B, Trancik R, Desonide Hydrogel Study Group. Safety and efficacy of desonide hydrogel 0.05% in pediatric subjects with atopic dermatitis. *J Drugs Dermatol*. 2007;**6**(2):175-81.
42. Udompataikul M, Limpa-o-vart D. Comparative trial of 5% dexpanthenol in water-in-oil formulation with 1% hydrocortisone ointment in the treatment of childhood atopic dermatitis: a pilot study. *J Drugs Dermatol*. 2012;**11**(3):366-74.
43. Wananukul S, Chatproedprai S, Chunharas A, Limpongsanuruk W, Singalavanija S, Nitiyarom R, et al. Randomized, double-blind, split-side, comparison study of moisturizer containing licochalcone A and 1% hydrocortisone in the treatment of childhood atopic dermatitis. *J Med Assoc Thai [Chotmaihet thangphaet]*. 2013;**96**(9):1135-42.
44. Jirabundansuk P, Ophaswongse S, Udompataikul M. Comparative trial of moisturizer containing spent grain wax, Butyrospermum parkii extract, Argania spinosa kernel oil vs. 1% hydrocortisone cream in the treatment of childhood atopic dermatitis. *J Med Assoc Thai [Chotmaihet thangphaet]*. 2014;**97**(8):820-6.
45. Dolle S, Hoser D, Rasche C, Loddenkemper C, Maurer M, Zuberbier T, et al. Long-term reduction in local inflammation by a lipid raft molecule in atopic dermatitis. *Allergy*. 2010;**65**(9):1158-65.
46. Patzelt-Wenczler R, Ponce-Poschl E. Proof of efficacy of Kamillosan(R) cream in atopic eczema. *Eur J Med Res*. 2000;**5**(4):171-5.
47. Paller A, Nimmagadda S, Schachner L, Mallory S, Kahn T, Willis I, et al. Fluocinolone acetonide 0.01% in peanut oil: therapy for childhood atopic dermatitis, even in patients who are peanut sensitive. *J Am Acad Dermatol*. 2003;**48**(4):569-77.
48. Patel L, Clayton P, Addison G, Price D, David T. Adrenal function following topical steroid treatment in children with atopic dermatitis. *Br J Dermatol*. 1995;**132**(6):950-5.
49. Dohil MA, Alvarez-Connelly E, Eichenfield LF. Fluocinolone acetonide 0.01% in peanut oil: safety and efficacy data in the treatment of childhood atopic dermatitis in infants as young as 3 months of age. *Pediatr Dermatol*. 2009;**26**(3):262-8.
50. Eichenfield LF, Basu S, Calvarese B, Trancik RJ. Effect of desonide hydrogel 0.05% on the hypothalamic-pituitary-adrenal axis in pediatric subjects with moderate to severe atopic dermatitis. *Pediatr Dermatol*. 2007;**24**(3):289-95.
51. Hebert AA, Desonide Foam Phase III Clinical Study Group. Desonide foam 0.05%: safety in children as young as 3 months. *J Am Acad Dermatol*. 2008;**59**(2):334-40.
52. Bieber T, Vick K, Folster-Holst R, Belloni-Fortina A, Stadtler G, Worm M, et al. Efficacy and safety of methylprednisolone aceponate ointment 0.1% compared to tacrolimus 0.03% in children and adolescents with an acute flare of severe atopic dermatitis. *Allergy*. 2007;**62**(2):184-9.
53. Broeders J, Ahmed Ali U, Fischer G. Systematic review and meta-analysis of randomized clinical trials (RCTs) comparing topical calcineurin inhibitors with topical corticosteroids for atopic dermatitis: A 15-year experience. *J Am Acad Dermatol*. 2016;**75**(2):410-9.e3.

54. Cury Martins J, Martins C, Aoki V, Gois AF, Ishii HA, da SEM. Topical tacrolimus for atopic dermatitis. Cochrane Database Syst Rev [Internet]. 2015; (7). Available from: http://onlinelibrary.wiley.com/doi/10.1002/14651858.CD009864.pub2/abstract.

55. Doss N, Kamoun M, Dubertret L, Cambazard F, Remitz A, Lahfa M, et al. Efficacy of tacrolimus 0.03% ointment as second-line treatment for children with moderate-to-severe atopic dermatitis: evidence from a randomized, double-blind non-inferiority trial vs. fluticasone 0.005% ointment. *Pediatr Allergy Immunol*. 2010;**21**(2 Pt 1):321-9.

56. Dong Y, Zeng W, Li W, Ma H, Zheng W. Efficacy and safety of topical tacrolimus for childhood atopic dermatitis; a meta-analysis. [Chinese]. *J Clin Dermatol*. 2017;**46**(4):239-42.

57. Doss N, Reitamo S, Dubertret L, Fekete G, Kamoun M, Lahfa M, et al. Superiority of tacrolimus 0.1% ointment compared with fluticasone 0.005% in adults with moderate to severe atopic dermatitis of the face: results from a randomized, double-blind trial. *Br J Dermatol*. 2009;**161**(2):427-34.

58. Luger T, Van Leent E, Graeber M, Hedgecock S, Thurston M, Kandra A, et al. SDZ ASM 981: an emerging safe and effective treatment for atopic dermatitis. *Br J Dermatol*. 2001;**144**(4):788-94.

59. Ashcroft D, Dimmock P, Garside R, Stein K, Williams H. Efficacy and tolerability of topical pimecrolimus and tacrolimus in the treatment of atopic dermatitis: Meta-analysis of randomised controlled trials. *BMJ* 2005;**330**(7490):516-22.

60. Ashcroft DM, Chen L-C, Garside R, Stein K, Williams HC. Topical pimecrolimus for eczema Cochrane Database Syst Rev [Internet]. 2007; (4). Available from: http://onlinelibrary.wiley.com/doi/10.1002/14651858.CD005500.pub2/abstract.

61. Luger T, Lahfa M, Folster-Holst R, Gulliver W, Allen R, Molloy S, et al. Long-term safety and tolerability of pimecrolimus cream 1% and topical corticosteroids in adults with moderate to severe atopic dermatitis. *J Dermatolog Treat*. 2004;**15**(3):169-78.

62. Mandelin J, Remitz A, Virtanen H, Reitamo S. One-year treatment with 0.1% tacrolimus ointment versus a corticosteroid regimen in adults with moderate to severe atopic dermatitis: A randomized, double-blind, comparative trial. *Acta Derm Venereol*. 2010;**90**(2):170-4.

63. Reitamo S, Rustin M, Ruzicka T, Cambazard F, Kalimo K, Friedmann P, et al. Efficacy and safety of tacrolimus ointment compared with that of hydrocortisone butyrate ointment in adult patients with atopic dermatitis. *J Allergy Clin Immunol*. 2002;**109**(3):547-55.

64. Iskedjian M, Piwko C, Shear N, Langley R, Einarson T. Topical calcineurin inhibitors in the treatment of atopic dermatitis: A meta-analysis of current evidence. *Am J Clin Dermatol*. 2004;**5**(4):267-79.

65. Penaloza Hidalgo B, Knight T, Burls A. A systematic review of effectiveness and cost effectiveness of tacrolimus ointment for topical treatment of atopic dermatitis in adults and children Health Technol Assess [Internet]. 2004; (4):[81 p.].

66. Reitamo S, Ortonne J, Sand C, Cambazard F, Bieber T, Folster-Holst R, et al. A multicentre, randomized, double-blind, controlled study of long-term treatment with 0.1% tacrolimus ointment in adults with moderate to severe atopic dermatitis. *Br J Dermatol*. 2005;**152**(6):1282-9.

67. Gradman J, Wolthers O. Short-term growth in children with eczema during treatment with topical mometasone furoate and tacrolimus. *Acta paediatrica*. 2007;**96**(8):1233-7.

68. Svensson A, Chambers C, Gånemo A, Mitchell S. A systematic review of tacrolimus ointment compared with corticosteroids in the treatment of atopic dermatitis. *Curr Med Res Opin*. 2011;**27**(7):1395-406.

69. FK506 Ointment Study Group. Phase III comparative study of FK506 ointment vs betamethasone valerate ointment in atopic dermatitis (trunk/extremities) [in Japanese]. *Nishinihon J Dermatol*. 1997;**59**:870-9.

70. Hofman T, Cranswick N, Kuna P, Boznanski A, Latos T, Gold M, et al. Tacrolimus ointment does not affect the immediate response to vaccination, the generation of immune memory, or humoral and cell-mediated immunity in children. *Arch Dis Child*. 2006;**91**(11):905-10.

71. Siegfried E, Jaworski J, Kaiser J, Hebert A. Systematic review of published trials: Long-term safety of topical corticosteroids and topical calcineurin inhibitors in pediatric patients with atopic dermatitis. *BMC Pediatr*. 2016;**16** (75).

72. Sikder M, Al Mamun S, Khan R, Chowdhury A, Khan H, Hoque M. Topical 0.03% tacrolimus ointment, 0.05% clobetasone butyrate cream alone and their combination in older children with atopic dermatitis - An open randomized comparative study. *J Pak Assoc Dermatol*. 2005;**15**(4):304-12.

73. Torok H, Maas-Irslinger R, Slayton R. Clocortolone pivalate cream 0.1% used concomitantly with tacrolimus ointment 0.1% in atopic dermatitis. *Cutis*. 2003;**72**(2):161-6.
74. Sigurgeirsson B, Boznanski A, Todd G, Vertruyen A, Schuttelaar M, Zhu X, et al. Safety and efficacy of pimecrolimus in atopic dermatitis: a 5-year randomized trial. *Pediatrics* 2015;**135**(4):597-606.
75. Reitamo S, Van Leent E, Ho V, Harper J, Ruzicka T, Kalimo K, et al. Efficacy and safety of tacrolimus ointment compared with that of hydrocortisone acetate ointment in children with atopic dermatitis. *J Allergy Clin Immunol*. 2002;**109**(3):539-46.
76. Reitamo S, Harper J, Bos J, Cambazard F, Bruijnzeel-Koomen C, Valk P, et al. 0.03% Tacrolimus ointment applied once or twice daily is more efficacious than 1% hydrocortisone acetate in children with moderate to severe atopic dermatitis: results of a randomized double-blind controlled trial. *Br J Dermatol*. 2004;**150**(3):554-62.
77. Gutgesell C, Jung T, Reich K, Junghans V, Bohn M, Neumann C. Double-blind hydrocortisone-controlled tacrolimus ointment for atopic dermatitis.(Abstract 1255). *J Invest Dermatol*. 1998;**110**(4):681.
78. Arellano F, Wentworth C, Arana A, Fernandez C, Paul C. Risk of lymphoma following exposure to calcineurin inhibitors and topical steroids in patients with atopic dermatitis. *J Invest Dermatol*. 2007;**127**(4):808-16.
79. Arellano F, Arana A, Wentworth C, Fernandez-Vidaurre C, Schlienger R, Conde E. Lymphoma among patients with atopic dermatitis and/or treated with topical immunosuppressants in the United Kingdom. *J Allergy Clin Immunol*. 2009;**123**(5):1111–6.
80. Schneeweiss S, Doherty M, Zhu S, Funch D, Schlienger R, Fernandez-Vidaurre C, et al. Topical treatments with pimecrolimus, tacrolimus and medium- to high-potency corticosteroids, and risk of lymphoma. *Dermatology*. 2009;**219**(1):7-21.
81. Reitamo S WA, Schopf E, Perrot JL, Marks R, Ruzicka T, et al. Safety and efficacy of 1 year of tacrolimus ointment monotherapy in adults with atopic dermatitis.The European Tacrolimus Ointment Study Group. *Arch Dermatol*. 2000;**136**(8):999-1006.
82. Ulrich R, Andresen I. Double-blind comparative trial involving 0.5% halomethasone (Sicorten (R)) cream versus 0.25% prednicarbate cream in patients with acute episodes of atopic dermatitis [Behandlung akuter schube der atopischen dermatitis. Doppelblinde vergleichsstudie mit 0,05%-halometason-creme versus 0,25%-prednicarbat-creme]. *MMW Fortschr Med*. 1991;**109**(36):49-50+3-4.
83. Smitt J, Winterberg D, Oosting J. Treatment of atopic dermatitis with topical corticosteroids in children. Efficacy and systemic effects of triamcinolone acetonide and alclometasone dipropionate. *Eur J Dermatol*. 1993;**3**(7):549-52.
84. Lebrun-Vignes B, Legrain V, Amoric J, Taieb A. [Comparative study of efficacy and effect on plasma cortisol levels of micronised desonide cream 0.1 p. 100 versus betamethasone dipropionate cream 0.05 p. 100 In the treatment of childhood atopic dermatitis] (French). *Ann Dermatol Venereol*. 2000;**127**(6-7):590-5.
85. Prado de Oliveira Z, Cuce L, Arnone M. Comparative evaluation of efficacy, tolerability and safety of 0.1% topical momethasone furoate and 0.05% desonide in the treatment of childhood atopic dermatitis. *An Bras Dermatol*. 2002;**77**:25-33.
86. Hanifin J. The role of topical steroids in the treatment of allergic skin disease. Towards Evolution of Allergic Skin Disease Management. Hong Kong: Excerpta Medica; 1996. p. 1-2.
87. Kirkup M, Birchall N, Weinberg E, Helm K, Kennedy C. Acute and maintenance treatment of atopic dermatitis in children - two comparative studies with fluticasone propionate (0.05%) cream. *J Dermatolog Treat*. 2003;**14**(3):141-8.
88. Tang T, Bieber T, Williams H. Are the concepts of induction of remission and treatment of subclinical inflammation in atopic dermatitis clinically useful? *J Allergy Clin Immunol*. 2014;**133**(6):1615-25.e1.
89. Kuokkanen K, Sillantaka I. Alclometasone dipropionate 0.05% vs hydrocortisone 1.0%: potential to induce cutaneous atrophy in children. *Clin Ther*. 1987;**9**(2):223-31.
90. Ellison JA, Patel L, Ray DW, David TJ, Clayton PE. Hypothalamic-pituitary-adrenal function and glucocorticoid sensitivity in atopic dermatitis. *Pediatrics*. 2000;**105**(4 Pt 1):794-9.

91. Eichenfield L, Tom W, Berger T, Krol A, Paller A, Schwarzenberger K, et al. Guidelines of care for the management of atopic dermatitis: Section 2. Management and treatment of atopic dermatitis with topical therapies. *J Am Acad Dermatol*. 2014;**71**(1):116-32.
92. Kristmundsdottir F, David T. Growth impairment in children with atopic eczema. *Journal of the Royal Society of Medicine*. 1987;**80**(1):9-12.
93. Patel L, Clayton P, Jenney M, Ferguson J, David T. Adult height in patients with childhood onset atopic dermatitis. *Arch Dis Child*. 1997;**76**(6):505-7.
94. Patel L, Clayton P, Addison G, Price D, David T. Linear growth in prepubertal children with atopic dermatitis. *Arch Dis Child*. 1998;**79**(2):169-72.
95. Aliaga A, Rodriguez M, Armijo M, Bravo J, Avila AL, Mascaro JM, et al. Double-blind study of prednicarbate versus fluocortin butyl ester in atopic dermatitis. *Int J Dermatol*. 1996;**35**(2):131-2.
96. Lucky A, Grote G, Williams J, Tuley M, Czernielewski J, Dolak T, et al. Effect of desonide ointment, 0.05%, on the hypothalamic-pituitary-adrenal axis of children with atopic dermatitis. *Cutis*. 1997;**59**(3):151-3.
97. Jorizzo J, Levy M, Lucky A, Shavin J, Goldberg G, Dunlap F, et al. Multicenter trial for long-term safety and efficacy comparison of 0.05% desonide and 1% hydrocortisone ointments in the treatment of atopic dermatitis in pediatric patients. *J Am Acad Dermatol*. 1995;**33**(1):74-7.
98. Froeschl B, Arts D, Leopold C. Corticosteroid therapy in the treatment of pediatric patients with atopic dermatitis (Structured abstract). Health Technol Assess [Internet]. 2007; (4). Available from: http://onlinelibrary.wiley.com/o/cochrane/clhta/articles/HTA-32008100208/frame.html.
99. Huang Z, Chen P, Wei W. Observation of efficacy of Chushi Zhiyang cream for atopic dermatitis. *Mod J Integr Trad Chin West Med*. 2010;**19**:2647–8.
100. Gu S, Yang AW, Xue CC, Li CG, Pang C, Zhang W, et al. Chinese herbal medicine for atopic eczema. Cochrane Database Syst Rev [Internet]. 2013; (9). Available from: http://onlinelibrary.wiley.com/doi/10.1002/14651858.CD008642.pub2/abstract.
101. Gu S, Yang A, Li C, Lu C, Xue C. Topical application of Chinese herbal medicine for atopic eczema: A systematic review with a meta-analysis. *Dermatology*. 2014;**228**(4):294-302.
102. Chen H. Clinical observation of Huanglian Qingdai cream for infantile eczema

J Sichuan Trad Chin Med. 2011;**29**:88–9.
103. Dong M. Efficacy of external use of Jingfang mixture in the treatment of children with eczema. *Med J Qilu*. 2012;**27**:75–8.
104. Xu XC GY. Clinical observation of Kouqiang Xiaoyan powder for infantile eczema. *Strait Pharm J*. 2012;**24**:210.
105. Schlessinger J, Miller B, Gilbert R, Plott R, Vanos Study Group. An open-label adrenal suppression study of 0.1% fluocinonide cream in pediatric patients with atopic dermatitis. *Arch Dermatol*. 2006;**142**(12):1568-72.
106. Bleehen S, Chu A, Hamann I, Holden C, Hunter J, Marks R. Fluticasone propionate 0.05% cream in the treatment of atopic eczema: a multicentre study comparing once-daily treatment and once-daily vehicle cream application versus twice-daily treatment. *Br J Dermatol*. 1995;**133**(4):592-7.
107. Green C, Colquitt J, Kirby J, Davidson P, Payne E. Clinical and cost-effectiveness of once-daily versus more frequent use of same potency topical corticosteroids for atopic eczema: a systematic review and economic evaluation. *Health Technol Assess (Winchester, England)*. 2004;**8**(47):iii,iv, 1-120.
108. (GSK) G. A four week multicentre, double blind study to compare safety and efficacy with an OD and BD administration of fluticasone propionate 0.005% ointment in the treatment of atopic eczema. 1995. Contract No.: Report 135L,Protocol No. GL/FLT/002.
109. Koopmans B, Lasthein Andersen B, Mork N, Austad J, Suhonen R, Roders G. Multicentre randomized double-blind study of Locoid Lipocream fatty cream twice daily versus Locoid Lipocream once daily and Locobase once daily. *J Dermatolog Treat*. 1995;**6**(2):103-6.
110. Tharp M. A comparison of twice-daily and once-daily administration of fluticasone propionate cream, 0.05%, in the treatment of eczema. *Cutis*. 1996;**57**(Suppl 2):19-26.
111. Hoybye S, Balk Moller S, De Cunha Bang F, Ottevanger V, Veien N. Continuous and intermittent treatment of atopic dermatitis in adults with mometasone furoate versus hydrocortisone 17-butyrate. *Curr Ther Res Clin Exp*. 1991;**50**(1):67-72.
112. Berth-Jones J, Damstra R, Golsch S, Livden J, Van Hooteghem O, Allegra F, et al. Twice weekly fluticasone propionate added to emollient maintenance treatment to reduce risk of relapse in atopic dermatitis: randomised, double blind, parallel group study. *BMJ* 2003;**326**(7403):1367-9.

113. Marchesi E, Rozzoni M, Pini P, Cainelli T. Comparative study of mometasone furoate and betamethasone dipropionate in the treatment of atopic dermatitis. *G Ital Dermatol Venereol*. 1994;**129**(1-2):IX-XII.
114. Richelli C, Piacentini G, Sette L, Bonizzato M, Andreoli A, Boner A. Clinical efficacy and tolerability of clobetasone 17-butyrate 0.5% lotion in children with atopic dermatitis. *Curr Ther Res Clin Exp*. 1990;**47**(3):413-7.
115. Schmitt J, Von Kobyletzki L, Svensson A, Apfelbacher C. Efficacy and tolerability of proactive treatment with topical corticosteroids and calcineurin inhibitors for atopic eczema: Systematic review and meta-analysis of randomized controlled trials. *Br J Dermatol*. 2011;**164**(2):415-28.
116. Glazenburg E, Wolkerstorfer A, Gerretsen A, Mulder P, Oranje A. Efficacy and safety of fluticasone propionate 0.005% ointment in the long-term maintenance treatment of children with atopic dermatitis: differences between boys and girls? *Pediatr Allergy Immunol*. 2009;**20**(1):59-66.
117. Hanifin J, Gupta A, Rajagopalan R. Intermittent dosing of fluticasone propionate cream for reducing the risk of relapse in atopic dermatitis patients. *Br J Dermatol*. 2002;**147**(3):528-37.
118. Van Der Meer J, Glazenburg E, Mulder P, Eggink H, Coenraads P. The management of moderate to severe atopic dermatitis in adults with topical fluticasone propionate. The Netherlands Adult Atopic DermatitisStudy Group. *Br J Dermatol*. 1999;**140**(6):1114-21.
119. Peserico A, Stadtler G, Sebastian M, Fernandez R, Vick K, Bieber T. Reduction of relapses of atopic dermatitis with methylprednisolone aceponate cream twice weekly in addition to maintenance treatment with emollient: a multicentre, randomized, double-blind, controlled study. *Br J Dermatol*. 2008;**158**(4):801-7.
120. Volden G. Successful treatment of therapy-resistant atopic dermatitis with clobetasol propionate and a hydrocolloid occlusive dressing. *Acta Derm Venereol Suppl (Stockh)*. 1992;**176**:126-8.
121. Braham S, Pugashetti R, Koo J, Maibach H. Occlusive therapy in atopic dermatitis: overview. *J Dermatolog Treat*. 2010;**21**(2):62-72.
122. Janmohamed S, Oranje A, Devillers A, Rizopoulos D, van Praag M, Van Gysel D, et al. The proactive wet-wrap method with diluted corticosteroids versus emollients in children with atopic dermatitis: a prospective, randomized, double-blind, placebo-controlled trial. *J Am Acad Dermatol*. 2014;**70**(6):1076-82.
123. Schnopp C, Holtmann C, Stock S, Remling R, Folster-Holst R, Ring J, et al. Topical steroids under wet-wrap dressings in atopic dermatitis - A vehicle-controlled trial. *Dermatology*. 2002;**204**(1):56-9.
124. McGowan R, Tucker P, Joseph D, Wallace A, Hughes I, Burrows N, et al. Short-term growth and bone turnover in children undergoing occlusive steroid ('wet-wrap') dressings for treatment of atopic eczema. *J Dermatolog Treat*. 2003;**14**(3):149-52.
125. Devillers A, Oranje A. Efficacy and safety of 'wet-wrap' dressings as an intervention treatment in children with severe and/or refractory atopic dermatitis: a critical review of the literature. *Br J Dermatol*. 2006;**154**(4):579-85.
126. Wolkerstorfer A, Visser R, De Waard van der Spek F, Mulder P, Oranje A. Efficacy and safety of wet-wrap dressings in children with severe atopic dermatitis: influence of corticosteroid dilution. *Br J Dermatol*. 2000;**143**(5):999-1004.
127. Tang W. Diluted steroid facial wet wraps for childhood atopic eczema. *Dermatology*. 2000;**200**(4):338-9.
128. Goodyear H, Spowart K, Harper J. 'Wet-wrap'dressings for the treatment of atopic eczema in children. *Br J Dermatol*. 1991;**125**(6):604.
129. Mallon E, Powell S, Bridgman A. 'Wet-wrap'dressings for the treatment of atopic eczema in the community. *J Dermatolog Treat*. 1994;**5**(2):97-8.
130. Devillers A, de Waard-van der Spek F, Mulder P, Oranje A. Treatment of refractory atopic dermatitis using 'wet-wrap'dressings and diluted corticosteroids: results of standardized treatment in both children and adults. *Dermatology*. 2002;**204**(1):50-5.
131. Foelster-Holst R, Nagel F, Zoellner P, Spaeth D. Efficacy of crisis intervention treatment with topical corticosteroid prednicarbat with and without partial wet-wrap dressing in atopic dermatitis. *Dermatology*. 2006;**212**(1):66-9.
132. Gonzalez-Lopez G, Ceballos-Rodriguez R, Gonzalez-Lopez J, Feito Rodriguez M, Herranz-Pinto P. Efficacy and safety of wet wrap therapy for patients with atopic dermatitis: a systematic review and meta-analysis. *Br J Dermatol*. 2017;**177**(3):688-95.
133. Beattie P, Lewis-Jones M. A pilot study on the use of wet wraps in infants with moderate atopic eczema. *Clin Exp Dermatol*. 2004;**29**(4):348-53.

134. Hindley D, Galloway G, Murray J, Gardener L. A randomised study of "wet wraps" versus conventional treatment for atopic eczema. *Arch Dis Child*. 2006;**91**(2):164-8.
