# Supplementary material for: Safety of topical corticosteroids in atopic eczema: an umbrella review
Source: BMJ Open. 2021 Jul 7;11(7):e046476. doi: 10.1136/bmjopen-2020-046476 (PMC8264889; doi:10.1136/bmjopen-2020-046476)
Supplement: Supplementary data [file bmjopen-2020-046476supp004.pdf]

Appendix 4: AMSTAR 2 ratings [1]:

Review ID	1 PICO	2 Protocol*	3 Study designs	4 Search strategy*	5 Duplicate screening	6 Duplicate data extraction	7 Excluded studies*	8 Included studies	9 Risk of bias assessed*	10 Funding of studies	11 Appropriate meta-analysis*	12 Risk of bias in meta-analysis	13 Risk of bias in discussion *	14 Hetero-geneity	15 Publication bias in meta-analysis*	16 Reviewers' conflict of interest	Overall rating
Ashcroft 2005 [2]	Yes	No	No	Yes	Yes	Yes	No	Partial yes	Partial yes	No	Yes	No	No	No	No	Yes	Critically low
Ashcroft 2007 [3]	Yes	Partial yes	No	Yes	Yes	Yes	Yes	Partial yes	Yes	No	N/A	N/A	Yes	Yes	N/A	Yes	Moderate
Barnes 2015 [4]	No	No	No	No	No	No	No	No	No	No	N/A	N/A	No	No	N/A	Yes	Critically low
Braham 2010 [5]	No	No	Yes	No	No	No	No	Partial yes	No	No	N/A	N/A	No	No	N/A	Yes	Critically low
Broeders 2016 [6]	Yes	No	No	Partial yes	No	No	No	Partial yes	Partial yes	Yes	No	No	No	No	Yes	Yes	Critically low
Callen 2007 [7]	Yes	No	No	No	No	No	No	No	No	No	N/A	N/A	No	No	N/A	Yes	Critically low
Chen 2010 [8]	Yes	No	No	Partial yes	No	No	No	Partial yes	Yes	Yes	N/A	N/A	No	No	N/A	Yes	Critically low
Cury Martins 2015 [9]	Yes	Yes	No	Yes	Yes	Yes	Yes	Partial yes	Yes	No	Yes	Yes	Yes	No	Yes	Yes	Moderate
De Tiedra 1997 [10]	No	No	No	No	No	No	No	Partial yes	No	No	N/A	N/A	No	No	N/A	No	Critically low
Devillers 2006 [11]	No	No	Yes	No	No	No	No	Partial yes	No	No	N/A	N/A	No	No	N/A	Yes	Critically low
Dong 2017 [12]	Yes	No	No	No	Yes	Yes	No	No	Yes	No	No	No	No	No	No	No	Critically low
Eichenfield 2014 [13]	No	No	Yes	No	No	No	No	No	No	No	N/A	N/A	No	No	N/A	Yes	Critically low
Feldman 2005 [14]	No	No	Yes	No	No	No	No	Partial yes	No	No	N/A	N/A	No	No	N/A	No	Critically low
Fishbein 2019 [15]	Yes	Partial yes	Yes	Partial yes	Yes	Yes	No	Yes	No	Yes	No	No	No	No	No	Yes	Critically low
Frangos 2008 [16]	No	No	No	No	No	No	No	No	No	No	N/A	N/A	No	No	N/A	No	Critically low
Froeschl 2007 [17]	No	No	No	No	No	No	Yes	Partial yes	No	Yes	N/A	N/A	No	No	N/A	No	Critically low

Gonzalez-Lopez 2017 [18]	Yes	No	No	Yes	Yes	Yes	No	Yes	Yes	Yes	Yes	Yes	Yes	Yes	Yes	Yes	Critically low
Green 2004 [19]	Yes	Partial Yes	No	No	Partial yes	Yes	Partial yes	Yes	Partial yes	Yes	N/A	N/A	Yes	Yes	N/A	Yes	Low
Gu 2013 [20]	Yes	Yes	No	Yes	Yes	Yes	Yes	Yes	Yes	Yes	Yes	Yes	Yes	Yes	Yes	Yes	High
Gu 2014 [21]	Yes	No	No	No	No	No	No	No	Yes	No	Yes	No	Yes	Yes	No	Yes	Critically low
Hajar 2015 [22]	Yes	Yes	Yes	No	Yes	Yes	No	No	Partial yes	No	N/A	N/A	No	No	N/A	Yes	Critically low
Hoare 2000 [23]	Yes	No	Yes	Yes	No	No	Yes	Partial yes	Partial yes	No	N/A	N/A	Yes	No	N/A	Yes	Low
Iskedjian 2004 [24]	Yes	No	No	Partial yes	Yes	Yes	No	Partial yes	Partial yes	No	N/A	N/A	No	No	N/A	No	Critically low
Juhasz 2017 [25]	No	No	No	No	No	No	No	No	No	N/A	N/A	N/A	No	No	N/A	Yes	Critically low
Labedz 2019 [26]	Yes	No	Yes	Yes	No	No	No	Partial yes	Yes	No	Yes	No	No	No	No	Yes	Critically low
Legendre 2015 [27]	Yes	No	No	No	Yes	Yes	No	Partial yes	Yes	No	Yes	No	No	Yes	Yes	Yes	Critically low
Li 2007 [28]	Yes	No	No	Partial yes	No	Yes	No	No	Partial yes	No	N/A	N/A	No	No	N/A	No	Critically low
Nankervis 2016 [29]	Yes	Partial yes	Yes	Partial yes	No	Yes	No	Partial yes	Partial yes	Yes	N/A	N/A	Yes	No	N/A	Yes	Low
Penaloza Hidalgo 2004 [30]	Yes	Partial yes	No	Yes	No	No	Yes	Partial yes	Partial yes	Yes	N/A	N/A	No	Yes	N/A	Yes	Low
Schmitt 2011 [31]	Yes	Partial yes	No	Partial yes	Yes	Yes	No	Yes	Yes	No	N/A	N/A	No	Yes	N/A	Yes	Critically low
Sidbury 2011 [32]	No	No	Yes	No	No	No	No	No	No	No	N/A	N/A	No	No	N/A	Yes	Critically low
Siegfried 2016 [33]	No	No	No	No	No	No	No	No	No	No	N/A	N/A	No	No	N/A	Yes	Critically low
Singh 2012 [34]	No	No	No	No	No	No	Partial yes	No	Partial yes	No	N/A	N/A	Yes	No	N/A	Yes	Critically low
Svensson 2011 [35]	Yes	No	No	Partial yes	Yes	No	No	Partial yes	Partial yes	No	Yes	No	No	Yes	No	Yes	Critically low
Tang 2014 [36]	Yes	No	No	Partial yes	No	No	No	No	No	No	N/A	N/A	No	No	N/A	Yes	Critically low
van Zuuren 2017 [37]	Yes	Yes	No	Partial yes	Yes	Yes	Yes	Yes	Yes	Yes	Yes	Yes	Yes	Yes	Yes	Yes	Moderate
Wood Heickman 2017 [38]	Yes	No	Yes	No	Yes	No	No	No	No	No	Yes	No	No	Yes	No	Yes	Critically low

Yan 2008 (39)	Yes	No	No	Partial yes	No	No	No	No	Partial yes	Yes	Yes	No	No	No	No	No	Critically low

Footnotes: AMSTAR 2 domains

1 Did the research questions and inclusion criteria for the review include the components of PICO?

**2 Did the report of the review contain an explicit statement that the review methods were established prior to the conduct of the review and did the report justify any significant deviations from the protocol?*

3 Did the review authors explain their selection of the study designs for inclusion in the review?

**4 Did the review authors use a comprehensive literature search strategy?*

5 Did the review authors perform study selection in duplicate?

6 Did the review authors perform data extraction in duplicate?

**7 Did the review authors provide a list of excluded studies and justify the exclusions?*

8 Did the review authors describe the included studies in adequate detail?

**9 Did the review authors use a satisfactory technique for assessing the risk of bias (RoB) in individual studies that were included in the review?*

10 Did the review authors report on the sources of funding for the studies included in the review?

**11 If meta-analysis was performed, did the review authors use appropriate methods for statistical combination of results?*

12 If meta-analysis was performed, did the review authors assess the potential impact of RoB in individual studies on the results of the meta-analysis or other evidence synthesis?

**13 Did the review authors account for RoB in primary studies when interpreting/discussing the results of the review?*

14 Did the review authors provide a satisfactory explanation for, and discussion of, any heterogeneity observed in the results of the review?

**15 If they performed quantitative synthesis did the review authors carry out an adequate investigation of publication bias (small study bias) and discuss its likely impact on the results of the review?*

16 Did the review authors report any potential sources of conflict of interest, including any funding they received for conducting the review?

NB domains marked * in the table and footnotes are critical domains.

1. Shea BJ, Reeves BC, Wells G, Thuku M, Hamel C, Moran J, et al. AMSTAR 2: a critical appraisal tool for systematic reviews that include randomised or non-randomised studies of healthcare interventions, or both. *BMJ*. 2017;**358**:j4008.
2. Ashcroft D, Dimmock P, Garside R, Stein K, Williams H. Efficacy and tolerability of topical pimecrolimus and tacrolimus in the treatment of atopic dermatitis: Meta-analysis of randomised controlled trials. *BMJ* 2005;**330**(7490):516-22.
3. Ashcroft DM, Chen L-C, Garside R, Stein K, Williams HC. Topical pimecrolimus for eczema Cochrane Database Syst Rev [Internet]. 2007; (4). Available from: http://onlinelibrary.wiley.com/doi/10.1002/14651858.CD005500.pub2/abstract.
4. Barnes L, Kaya G, Rollason V. Topical Corticosteroid-Induced Skin Atrophy: A Comprehensive Review. *Drug Saf*. 2015;**38**(5):493-509.
5. Braham S, Pugashetti R, Koo J, Maibach H. Occlusive therapy in atopic dermatitis: overview. *J Dermatolog Treat*. 2010;**21**(2):62-72.
6. Broeders J, Ahmed Ali U, Fischer G. Systematic review and meta-analysis of randomized clinical trials (RCTs) comparing topical calcineurin inhibitors with topical corticosteroids for atopic dermatitis: A 15-year experience. *J Am Acad Dermatol*. 2016;**75**(2):410-9.e3.
7. Callen J, Chamlin S, Eichenfield L, Ellis C, Girardi M, Goldfarb M, et al. A systematic review of the safety of topical therapies for atopic dermatitis. *Br J Dermatol*. 2007;**156**(2):203-21.
8. Chen S, Yan J, Wang F. Two topical calcineurin inhibitors for the treatment of atopic dermatitis in pediatric patients: A meta-analysis of randomized clinical trials. *J Dermatolog Treat*. 2010;**21**(3):144-56.
9. Cury Martins J, Martins C, Aoki V, Gois AF, Ishii HA, da SEM. Topical tacrolimus for atopic dermatitis. Cochrane Database Syst Rev [Internet]. 2015; (7). Available from: http://onlinelibrary.wiley.com/doi/10.1002/14651858.CD009864.pub2/abstract.
10. de Tiedra A, Mercadal J, Lozano R. Prednicarbate versus fluocortin for inflammatory dermatoses: A cost-effectiveness study. *PharmacoEconomics*. 1997;**12**(2 Pt 1):193-208.
11. Devillers A, Oranje A. Efficacy and safety of 'wet-wrap' dressings as an intervention treatment in children with severe and/or refractory atopic dermatitis: a critical review of the literature. *Br J Dermatol*. 2006;**154**(4):579-85.
12. Dong Y, Zeng W, Li W, Ma H, Zheng W. Efficacy and safety of topical tacrolimus for childhood atopic dermatitis; a meta-analysis. [Chinese]. *J Clin Dermatol*. 2017;**46**(4):239-42.
13. Eichenfield L, Tom W, Berger T, Krol A, Paller A, Schwarzenberger K, et al. Guidelines of care for the management of atopic dermatitis: Section 2. Management and treatment of atopic dermatitis with topical therapies. *J Am Acad Dermatol*. 2014;**71**(1):116-32.
14. Feldman S. Relative efficacy and interchangeability ofvarious clobetasol propionate vehicles in the management of steroid-responsive dermatoses. *Curr Ther Res Clin Exp*. 2005;**66**(3):154-71.
15. Fishbein AB, Mueller K, Lor J, Smith P, Paller AS, Kaat A. Systematic Review and Meta-analysis Comparing Topical Corticosteroids With Vehicle/Moisturizer in Childhood Atopic Dermatitis. *J Pediatr Nurs*. 2019;**47**:36-43.

16. Frangos J, Kimball A. Clobetasol propionate emollient formulation foam in the treatment of corticosteroid-responsive dermatoses. *Expert Opin Pharmacother*. 2008;**9**(11):2001-7.
17. Froeschl B, Arts D, Leopold C. Corticosteroid therapy in the treatment of pediatric patients with atopic dermatitis (Structured abstract). Health Technol Assess [Internet]. 2007; (4). Available from: http://onlinelibrary.wiley.com/o/cochrane/clhta/articles/HTA-32008100208/frame.html.
18. Gonzalez-Lopez G, Ceballos-Rodriguez R, Gonzalez-Lopez J, Feito Rodriguez M, Herranz-Pinto P. Efficacy and safety of wet wrap therapy for patients with atopic dermatitis: a systematic review and meta-analysis. *Br J Dermatol*. 2017;**177**(3):688-95.
19. Green C, Colquitt J, Kirby J, Davidson P, Payne E. Clinical and cost-effectiveness of once-daily versus more frequent use of same potency topical corticosteroids for atopic eczema: a systematic review and economic evaluation. *Health Technol Assess (Winchester, England)*. 2004;**8**(47):iii,iv, 1-120.
20. Gu S, Yang AW, Xue CC, Li CG, Pang C, Zhang W, et al. Chinese herbal medicine for atopic eczema. Cochrane Database Syst Rev [Internet]. 2013; (9). Available from: http://onlinelibrary.wiley.com/doi/10.1002/14651858.CD008642.pub2/abstract.
21. Gu S, Yang A, Li C, Lu C, Xue C. Topical application of Chinese herbal medicine for atopic eczema: A systematic review with a meta-analysis. *Dermatology*. 2014;**228**(4):294-302.
22. Hajar T, Leshem Y, Hanifin J, Nedorost S, Lio P, Paller A, et al. A systematic review of topical corticosteroid withdrawal ("steroid addiction") in patients with atopic dermatitis and other dermatoses. *J Am Acad Dermatol*. 2015;**72**(3):541-9.e2.
23. Hoare C, Li Wan Po A, Williams H. Systematic review of treatments for atopic eczema. *Health Technol Assess*. 2000;**4**(37):1-191.
24. Iskedjian M, Piwko C, Shear N, Langley R, Einarson T. Topical calcineurin inhibitors in the treatment of atopic dermatitis: A meta-analysis of current evidence. *Am J Clin Dermatol*. 2004;**5**(4):267-79.
25. Juhász ML, Curley RA, Rasmussen A, Malakouti M, Silverberg N, Jacob SE. Systematic Review of the Topical Steroid Addiction and Topical Steroid Withdrawal Phenomenon in Children Diagnosed With Atopic Dermatitis and Treated With Topical Corticosteroids. *J Dermatol Nurses Assoc*. 2017;**9**(5):233-40.
26. Łabędź N, Pawliczak R. Efficacy and safety of topical calcineurin inhibitors for the treatment of atopic dermatitis: meta-analysis of randomized clinical trials. *Postepy Dermatol Alergol*. 2019;**36**(6):752-9.
27. Legendre L, Barnetche T, Mazereeuw-Hautier J, Meyer N, Murrell D, Paul C. Risk of lymphoma in patients with atopic dermatitis and the role of topical treatment: A systematic review and meta-analysis. *J Am Acad Dermatol*. 2015;**72**(6):992-1002.
28. Li R, Zhu H, Fan L, Ni S, Feng C, Wu Z. Efficacy and tolerability of topical tacrolimus in the treatment of atopic dermatitis: A systematic review of randomized controlled trials. [Chinese]. *J Clin Dermatol*. 2007;**36**(12):757-60.
29. Nankervis H, Thomas K, Delamere F, Barbarot S, Rogers N, Williams H. Scoping systematic review of treatments for eczema2016 2016/05/None.
30. Penaloza Hidalgo B, Knight T, Burls A. A systematic review of effectiveness and cost effectiveness of tacrolimus ointment for topical treatment of atopic dermatitis in adults and children Health Technol Assess [Internet]. 2004; (4):[81 p.].
31. Schmitt J, Von Kobyletzki L, Svensson A, Apfelbacher C. Efficacy and tolerability of proactive treatment with topical corticosteroids and calcineurin inhibitors for atopic eczema: Systematic review and meta-analysis of randomized controlled trials. *Br J Dermatol*. 2011;**164**(2):415-28.
32. Sidbury R, Tom W, Bergman J, Cooper K, Silverman R, Berger T, et al. Guidelines of care for the management of atopic dermatitis: Section 4. Prevention of disease flares and use of adjunctive therapies and approaches. *J Am Acad Dermatol*. 2014;**71**(6):1218-33.

33. Siegfried E, Jaworski J, Kaiser J, Hebert A. Systematic review of published trials: Long-term safety of topical corticosteroids and topical calcineurin inhibitors in pediatric patients with atopic dermatitis. *BMC Pediatr*. 2016;**16** (75).
34. Singh S, Mann B. Clinical utility of clocortolone pivalate for the treatment of corticosteroid-responsive skin disorders: A systematic review. *Clin Cosmet Investig Dermatol*. 2012;**5**:61-8.
35. Svensson A, Chambers C, Gånemo A, Mitchell S. A systematic review of tacrolimus ointment compared with corticosteroids in the treatment of atopic dermatitis. *Curr Med Res Opin*. 2011;**27**(7):1395-406.
36. Tang T, Bieber T, Williams H. Are the concepts of induction of remission and treatment of subclinical inflammation in atopic dermatitis clinically useful? *J Allergy Clin Immunol*. 2014;**133**(6):1615-25.e1.
37. van Zuuren EJ, Fedorowicz Z, Christensen R, Lavrijsen AP, Arents BW. Emollients and moisturisers for eczema. Cochrane Database Syst Rev [Internet]. 2017; (2). Available from: http://onlinelibrary.wiley.com/doi/10.1002/14651858.CD012119.pub2/abstract.
38. Wood Heickman L, Davallow Ghajar L, Conaway M, Rogol A. Evaluation of Hypothalamic-Pituitary-Adrenal Axis Suppression following Cutaneous Use of Topical Corticosteroids in Children: A Meta-Analysis. *Horm Res Paediatr*. 2018;**89**(6):389-96.
39. Yan J, Chen S, Wang X, Zhou W, Wang F. Meta-analysis of tacrolimus ointment for atopic dermatitis in pediatric patients. *Pediatr Dermatol*. 2008;**25**(1):117-20.
