# Supplementary material for: Safety of topical corticosteroids in atopic eczema: an umbrella review
Source: BMJ Open. 2021 Jul 7;11(7):e046476. doi: 10.1136/bmjopen-2020-046476 (PMC8264889; doi:10.1136/bmjopen-2020-046476)
Supplement: Supplementary data [file bmjopen-2020-046476supp005.pdf]

Appendix 5: Characteristics of the included systematic reviews

First author and year of publication	Type of review	Language of publication	Funding source	Conflicts of interest	RCT comparisons	Observational data included	AMSTAR-2 rating
Ashcroft 2005 (1)	Non-Cochrane	English	NHS health technology assessment programme.	None known	TCS versus TCI	No	Critically low
Ashcroft 2007 (2)	Cochrane	English	School of Pharmacy and Pharmaceutical Sciences, University of Manchester, UK.	None known	TCS versus TCI	Yes – TCI compared with TCS	Moderate
Barnes 2015 (3)	Non-Cochrane	English	No funding	None known	TCS versus vehicle<br>TCS versus TCI<br>TCS versus TCS	Yes (single arm TCS studies)	Critically low
Braham 2010 (4)	Non-Cochrane	English	No funding	One author was a speaker for a number of pharmaceutical companies	Occlusion therapy versus non-occlusion therapy	Yes (occlusive therapy, no comparison)	Critically low
Broeders 2016 (5)	Non-Cochrane	English	No funding	None known	TCS versus TCI	No	Critically low
Callen 2007 (6)	Non-Cochrane	English	Funding from EBMed. One author received funding from Novartis Corporation for the project. They declared that "Novartis Corporation played no role in the design and conduct of the study or in data collection, data management, data analysis, interpretation of the data, manuscript preparation, manuscript review or manuscript approval"	Most authors had consultancy fees and/or research support from pharmaceutical companies	TCS versus vehicle<br>TCS versus TCS	Yes (single arm TCS studies or comparing various TCS potencies)	Critically low
Chen 2010 (7)	Non-Cochrane	English	Not stated	None known	TCS versus TCI	No	Critically low
Cury Martins 2015 (8)	Cochrane	English	NIHR	None known	TCS versus TCI	Yes – TCI compared to TCS	Moderate
De Tiedra 1997 (9)	Non-Cochrane	English	Supported by Laboratorios Novag, S.A, Grupo Ferrer.	Not clear	TCS versus TCS	Yes – in most cases they only report data from one arm of an RCT	Critically low
Devillers 2006 (10)	Non-Cochrane	English	Not stated	None known	Occlusive therapy versus non-occlusive therapy	Yes – occlusive therapy (no comparison)	Critically low
Dong 2017 (11)	Non-Cochrane	Chinese	Not stated	Not clear	TCS versus TCI	No	Critically low
Eichenfield 2014 (12)	Non-Cochrane	English	No funding	Most authors served as consultants, speakers, members of the advisory	None	Yes (comparing different TCS potencies)	Critically low

				board and/or investigators for pharmaceutical companies.			
Feldman 2005 [13]	Non-Cochrane	English	Grant from Galderma Laboratories, LP, Fort Worth, Texas.	Not clear	TCS versus vehicle	No	Critically low
Fishbein 2019 [14]	Non-Cochrane	English	No funding	None known	TCS versus vehicle/moisturizer	No	Critically low
Frangos 2008 [15]	Non-Cochrane	English	Not stated	One author is an investigator for Steifel and was an investigator on two of the studies reviewed.	TCS versus vehicle	Yes (single arm studies)	Critically low
Froschl 2007 [16]	GMS HTA report	German (executive summary in English)	Not stated	Not stated	TCS versus placebo/vehicle<br>TCS versus TCS<br>TCS versus TCI	No	Critically low
Gonzalez-Lopez 2017 [17]	Non-Cochrane	English	No funding	None known	Occlusive therapy versus non-occlusive therapy	No	Critically low
Green 2004 [18]	HTA report	English	Funded by the HTA Programme on behalf of NICE	None known	Once daily versus twice daily TCS use	No	Low
Gu 2013 [19]	Cochrane	English	RMIT University<br>Nottingham University, UK.<br>NIHR	One author was a principal investigator on one included study (but this study was not relevant for this overview)	Chinese herbal medicine versus TCS	No	High
Gu 2014 [20]	Non-Cochrane	English	Not stated	None known	Chinese herbal medicine versus TCS	No	Critically low
Hajar 2015 [21]	Non-Cochrane	English	No funding	None known	No RCTs found	Yes (case series or case reports on steroid withdrawal)	Critically low
Hoare 2000 [22]	NIHR HTA report	English	HTA programme	One author received payment from Novartis for lectures on the epidemiology of atopic eczema in 1999.<br>Another author has acted as occasional lecturer or consultant for pharmaceutical companies.	TCS versus TCS<br>TCS versus vehicle	No	Low
Iskedjian 2004 [23]	Non-Cochrane	English	Funded by Fujisawa Canada Inc.	Not clear	TCS versus TCI<br>TCS versus placebo	No	Critically low
Juhasz 2017 [24]	Non-Cochrane	English	Not stated	One author had primary contact with the 2nd case and has a blog on the subject matter in this systematic review	No RCTs found	Yes (case reports on steroid withdrawal)	Critically low

Labedz 2019 [25]	Non-Cochrane	English	Not stated	None known	TCS versus TCI	No	Critically low
Legendre 2015 [26]	Non-Cochrane	English	No funding	One author is a consultant and investigator for two pharmaceutical companies. One author is a speaker and/or on the advisory board for five pharmaceutical companies.	Only searched for cohort or case control studies	Yes (comparing TCS and TCI)	Critically low
Li 2007 [27]	Non-Cochrane	Chinese	Not stated	Not stated	TCS versus TCI	No	Critically low
Nankervis 2016 [28]	NIHR HTA report	English	NIHR	One author reports grants and fees from a number of pharmaceutical companies.	TCS versus placebo/vehicle<br>Proactive treatment versus vehicle<br>TCS versus TCI<br>TCS versus TCS<br>Once a day versus twice a day use of TCS<br>Occlusive therapy versus non-occlusive therapy<br>TCS versus emollients	No	Low
Penzaloza Hidalgo 2004 [29]	West Midlands HTA report	English	Not stated	None known	TCS versus TCI	No	Low
Schmitt 2011 [30]	Non-Cochrane	English	No funding	One author has served as paid lecturer for a pharmaceutical company.	Proactive treatment versus vehicle	No	Critically low
Sidbury 2014 [31]	Non-Cochrane	English	Not stated	Some authors have served as investigators, consultants, speakers, and on advisory boards for pharmaceutical companies.	Proactive treatment versus vehicle	No	Critically low
Siegfried 2016 [32]	Non-Cochrane	English	Financial support for writing by Valent Pharmaceutical North America LLC. They declared that "Valeant Pharmaceuticals had no role in the design of the literature searches, or analysis and presentation of results."	Authors have either participated in paid contract research, received travel expenses for presentations, consulting fees, speakers, on advisory boards, or on data safety monitoring boards with pharmaceutical companies.	TCS versus TCS<br>TCS versus TCI<br>TCS versus vehicle	No	Critically low
Singh 2012 [33]	Non-Cochrane	English	Not stated	None known	TCS versus TCS<br>TCI versus TCS<br>TCS versus placebo/vehicle	Yes (single arm TCS study)	Critically low

Svensson 2011 [34]	Non-Cochrane	English	Funded by Astellas Pharma Europe Ltd.	One author was a paid employee of Astellas Pharma Europe Ltd and one author undertook paid consultancy work for Astellas Pharma Europe Ltd.	TCI versus TCS	No	Critically low
Tang 2014 [35]	Non-Cochrane	English	Not stated	One author has received lecture fees from Astellas.	Proactive treatment versus vehicle	No	Critically low
van Zuuren 2017 [36]	Cochrane	English	Oak Foundation, Denmark<br>NIHR	None known	TCS versus emollient	No	Moderate
Wood Heickman 2018 [37]	Non-Cochrane	English	No grants, honoraria or royalties were received supporting the writing of the paper.	One author was a consultant with Perrigo, Inc. with regard to topical corticosteroid treatment. All authors have no financial or other potential conflicts of interest.	Two RCTs included but analysed as observational data	Yes – single arm cohort studies	Critically low
Yan 2008 [38]	Non-Cochrane	English	Not stated	Not stated	TCI versus TCS	No	Critically low

Key: TCI=topical calcinueirin inhibitor; TCS=topical corticosteroids; RCT=Randomised Controlled Trial; NIHR= National Institute for Health Research

1. Ashcroft D, Dimmock P, Garside R, Stein K, Williams H. Efficacy and tolerability of topical pimecrolimus and tacrolimus in the treatment of atopic dermatitis: Meta-analysis of randomised controlled trials. *BMJ* 2005;**330**(7490):516-22.
2. Ashcroft DM, Chen L-C, Garside R, Stein K, Williams HC. Topical pimecrolimus for eczema Cochrane Database Syst Rev [Internet]. 2007; (4). Available from: http://onlinelibrary.wiley.com/doi/10.1002/14651858.CD005500.pub2/abstract.
3. Barnes L, Kaya G, Rollason V. Topical Corticosteroid-Induced Skin Atrophy: A Comprehensive Review. *Drug Saf*. 2015;**38**(5):493-509.
4. Braham S, Pugashetti R, Koo J, Maibach H. Occlusive therapy in atopic dermatitis: overview. *J Dermatolog Treat*. 2010;**21**(2):62-72.
5. Broeders J, Ahmed Ali U, Fischer G. Systematic review and meta-analysis of randomized clinical trials (RCTs) comparing topical calcineurin inhibitors with topical corticosteroids for atopic dermatitis: A 15-year experience. *J Am Acad Dermatol*. 2016;**75**(2):410-9.e3.
6. Callen J, Chamlin S, Eichenfield L, Ellis C, Girardi M, Goldfarb M, et al. A systematic review of the safety of topical therapies for atopic dermatitis. *Br J Dermatol*. 2007;**156**(2):203-21.
7. Chen S, Yan J, Wang F. Two topical calcineurin inhibitors for the treatment of atopic dermatitis in pediatric patients: A meta-analysis of randomized clinical trials. *J Dermatolog Treat*. 2010;**21**(3):144-56.

8. Cury Martins J, Martins C, Aoki V, Gois AF, Ishii HA, da SEM. Topical tacrolimus for atopic dermatitis. Cochrane Database Syst Rev [Internet]. 2015; (7). Available from: http://onlinelibrary.wiley.com/doi/10.1002/14651858.CD009864.pub2/abstract.

9. de Tiedra A, Mercadal J, Lozano R. Prednicarbate versus fluocortin for inflammatory dermatoses: A cost-effectiveness study. *PharmacoEconomics*. 1997;**12**(2 Pt 1):193-208.

10. Devillers A, Oranje A. Efficacy and safety of 'wet-wrap' dressings as an intervention treatment in children with severe and/or refractory atopic dermatitis: a critical review of the literature. *Br J Dermatol*. 2006;**154**(4):579-85.

11. Dong Y, Zeng W, Li W, Ma H, Zheng W. Efficacy and safety of topical tacrolimus for childhood atopic dermatitis; a meta-analysis. [Chinese]. *J Clin Dermatol*. 2017;**46**(4):239-42.

12. Eichenfield L, Tom W, Berger T, Krol A, Paller A, Schwarzenberger K, et al. Guidelines of care for the management of atopic dermatitis: Section 2. Management and treatment of atopic dermatitis with topical therapies. *J Am Acad Dermatol*. 2014;**71**(1):116-32.

13. Feldman S. Relative efficacy and interchangeability ofvarious clobetasol propionate vehicles in the management of steroid-responsive dermatoses. *Curr Ther Res Clin Exp*. 2005;**66**(3):154-71.

14. Fishbein AB, Mueller K, Lor J, Smith P, Paller AS, Kaat A. Systematic Review and Meta-analysis Comparing Topical Corticosteroids With Vehicle/Moisturizer in Childhood Atopic Dermatitis. *J Pediatr Nurs*. 2019;**47**:36-43.

15. Frangos J, Kimball A. Clobetasol propionate emollient formulation foam in the treatment of corticosteroid-responsive dermatoses. *Expert Opin Pharmacother*. 2008;**9**(11):2001-7.

16. Froeschl B, Arts D, Leopold C. Corticosteroid therapy in the treatment of pediatric patients with atopic dermatitis (Structured abstract). Health Technol Assess [Internet]. 2007; (4). Available from: http://onlinelibrary.wiley.com/o/cochrane/clhta/articles/HTA-32008100208/frame.html.

17. Gonzalez-Lopez G, Ceballos-Rodriguez R, Gonzalez-Lopez J, Feito Rodriguez M, Herranz-Pinto P. Efficacy and safety of wet wrap therapy for patients with atopic dermatitis: a systematic review and meta-analysis. *Br J Dermatol*. 2017;**177**(3):688-95.

18. Green C, Colquitt J, Kirby J, Davidson P, Payne E. Clinical and cost-effectiveness of once-daily versus more frequent use of same potency topical corticosteroids for atopic eczema: a systematic review and economic evaluation. *Health Technol Assess (Winchester, England)*. 2004;**8**(47):iii,iv, 1-120.

19. Gu S, Yang A, Li C, Lu C, Xue C. Topical application of Chinese herbal medicine for atopic eczema: A systematic review with a meta-analysis. *Dermatology*. 2014;**228**(4):294-302.

20. Gu S, Yang AW, Xue CC, Li CG, Pang C, Zhang W, et al. Chinese herbal medicine for atopic eczema. Cochrane Database Syst Rev [Internet]. 2013; (9). Available from: http://onlinelibrary.wiley.com/doi/10.1002/14651858.CD008642.pub2/abstract.

21. Hajar T, Leshem Y, Hanifin J, Nedorost S, Lio P, Paller A, et al. A systematic review of topical corticosteroid withdrawal ("steroid addiction") in patients with atopic dermatitis and other dermatoses. *J Am Acad Dermatol*. 2015;**72**(3):541-9.e2.

22. Hoare C, Li Wan Po A, Williams H. Systematic review of treatments for atopic eczema. *Health Technol Assess*. 2000;**4**(37):1-191.

23. Iskedjian M, Piwko C, Shear N, Langley R, Einarson T. Topical calcineurin inhibitors in the treatment of atopic dermatitis: A meta-analysis of current evidence. *Am J Clin Dermatol*. 2004;**5**(4):267-79.

24. Juhász ML, Curley RA, Rasmussen A, Malakouti M, Silverberg N, Jacob SE. Systematic Review of the Topical Steroid Addiction and Topical Steroid Withdrawal Phenomenon in Children Diagnosed With Atopic Dermatitis and Treated With Topical Corticosteroids. *J Dermatol Nurses Assoc*. 2017;**9**(5):233-40.

25. Łabędź N, Pawliczak R. Efficacy and safety of topical calcineurin inhibitors for the treatment of atopic dermatitis: meta-analysis of randomized clinical trials. *Postepy Dermatol Alergol*. 2019;**36**(6):752-9.

26. Legendre L, Barnetche T, Mazereeuw-Hautier J, Meyer N, Murrell D, Paul C. Risk of lymphoma in patients with atopic dermatitis and the role of topical treatment: A systematic review and meta-analysis. *J Am Acad Dermatol*. 2015;**72**(6):992-1002.

27. Li R, Zhu H, Fan L, Ni S, Feng C, Wu Z. Efficacy and tolerability of topical tacrolimus in the treatment of atopic dermatitis: A systematic review of randomized controlled trials. [Chinese]. *J Clin Dermatol*. 2007;**36**(12):757-60.

28. Nankervis H, Thomas K, Delamere F, Barbarot S, Rogers N, Williams H. Scoping systematic review of treatments for eczema2016 2016/05/None.

29. Penaloza Hidalgo B, Knight T, Burls A. A systematic review of effectiveness and cost effectiveness of tacrolimus ointment for topical treatment of atopic dermatitis in adults and children Health Technol Assess [Internet]. 2004; (4):[81 p.].

30. Schmitt J, Von Kobyletzki L, Svensson A, Apfelbacher C. Efficacy and tolerability of proactive treatment with topical corticosteroids and calcineurin inhibitors for atopic eczema: Systematic review and meta-analysis of randomized controlled trials. *Br J Dermatol*. 2011;**164**(2):415-28.

31. Sidbury R, Tom W, Bergman J, Cooper K, Silverman R, Berger T, et al. Guidelines of care for the management of atopic dermatitis: Section 4. Prevention of disease flares and use of adjunctive therapies and approaches. *J Am Acad Dermatol*. 2014;**71**(6):1218-33.

32. Siegfried E, Jaworski J, Kaiser J, Hebert A. Systematic review of published trials: Long-term safety of topical corticosteroids and topical calcineurin inhibitors in pediatric patients with atopic dermatitis. *BMC Pediatr*. 2016;**16** (75).

33. Singh S, Mann B. Clinical utility of clocortolone pivalate for the treatment of corticosteroid-responsive skin disorders: A systematic review. *Clin Cosmet Investig Dermatol*. 2012;**5**:61-8.

34. Svensson A, Chambers C, Gånemo A, Mitchell S. A systematic review of tacrolimus ointment compared with corticosteroids in the treatment of atopic dermatitis. *Curr Med Res Opin*. 2011;**27**(7):1395-406.

35. Tang T, Bieber T, Williams H. Are the concepts of induction of remission and treatment of subclinical inflammation in atopic dermatitis clinically useful? *J Allergy Clin Immunol*. 2014;**133**(6):1615-25.e1.

36. van Zuuren EJ, Fedorowicz Z, Christensen R, Lavrijsen AP, Arents BW. Emollients and moisturisers for eczema. Cochrane Database Syst Rev [Internet]. 2017; (2). Available from: http://onlinelibrary.wiley.com/doi/10.1002/14651858.CD012119.pub2/abstract.

37. Wood Heickman L, Davallow Ghajar L, Conaway M, Rogol A. Evaluation of Hypothalamic-Pituitary-Adrenal Axis Suppression following Cutaneous Use of Topical Corticosteroids in Children: A Meta-Analysis. *Horm Res Paediatr*. 2018;**89**(6):389-96.

38. Yan J, Chen S, Wang X, Zhou W, Wang F. Meta-analysis of tacrolimus ointment for atopic dermatitis in pediatric patients. *Pediatr Dermatol*. 2008;**25**(1):117-20.
