# Supplementary material for: Safety of topical corticosteroids in atopic eczema: an umbrella review
Source: BMJ Open. 2021 Jul 7;11(7):e046476. doi: 10.1136/bmjopen-2020-046476 (PMC8264889; doi:10.1136/bmjopen-2020-046476)
Supplement: Supplementary data [file bmjopen-2020-046476supp007.pdf]

Appendix 7: edits made to meta-analyses and data from Broeders *et al* 2016 [1]

1) Switched the forest plot labels around so topical corticosteroids are the intervention and topical calcineurin inhibitors are is the comparator.
2) Amended a data error given in the skin infection forest plot where the number of events and participants were given the wrong way round for topical corticosteroids and topical calcineurin inhibitors in Luger et al 2004 [2].
3) Added skin atrophy data from Sigurgeirsson et al 2015 [3] into the forest plot – this is not provided in the publication but is given in online correspondence on the journal website
4) Changed to random effects instead of fixed effects as the decision was based on whether the I^2 value which is not appropriate.
5) Bieber et al 2007 [4] was listed as "least potent" in table I of the publication – but according to the Australian potency classification it should be classified as potent.
6) In table I, the topical calcineurin inhibitors given for Mandelin et al 2010 [5] is tacrolimus 1% - this should be 0.1%.
7) In table I, the therapy given for Hofman et al 2006 [6] was hydrocortisone acetate 0.1%. However, patients used hydrocortisone ointment 1% (mild potency) twice daily for head/neck and hydrocortisone butyrate ointment 0.1% (potent) for trunk and limbs for 2 weeks then hydrocortisone 1% twice daily for flares.

References

1. Broeders J, Ahmed Ali U, Fischer G. Systematic review and meta-analysis of randomized clinical trials (RCTs) comparing topical calcineurin inhibitors with topical corticosteroids for atopic dermatitis: A 15-year experience. *J Am Acad Dermatol*. 2016;**75**(2):410-9.e3.
2. Luger T, Van Leent E, Graeber M, Hedgecock S, Thurston M, Kandra A, et al. SDZ ASM 981: an emerging safe and effective treatment for atopic dermatitis. *Br J Dermatol*. 2001;**144**(4):788-94.
3. Sigurgeirsson B, Boznanski A, Todd G, Vertruyen A, Schuttelaar M, Zhu X, et al. Safety and efficacy of pimecrolimus in atopic dermatitis: a 5-year randomized trial. *Pediatrics* 2015;**135**(4):597-606.
4. Bieber T, Vick K, Folster-Holst R, Belloni-Fortina A, Stadtler G, Worm M, et al. Efficacy and safety of methylprednisolone aceponate ointment 0.1% compared to tacrolimus 0.03% in children and adolescents with an acute flare of severe atopic dermatitis. *Allergy*. 2007;**62**(2):184-9.
5. Mandelin J, Remitz A, Virtanen H, Reitamo S. One-year treatment with 0.1% tacrolimus ointment versus a corticosteroid regimen in adults with moderate to severe atopic dermatitis: A randomized, double-blind, comparative trial. *Acta Derm Venereol*. 2010;**90**(2):170-4.
6. Hofman T, Cranswick N, Kuna P, Boznanski A, Latos T, Gold M, et al. Tacrolimus ointment does not affect the immediate response to vaccination, the generation of immune memory, or humoral and cell-mediated immunity in children. *Arch Dis Child*. 2006;**91**(11):905-10.
