# Supplementary material for: Safety of topical corticosteroids in atopic eczema: an umbrella review
Source: BMJ Open. 2021 Jul 7;11(7):e046476. doi: 10.1136/bmjopen-2020-046476 (PMC8264889; doi:10.1136/bmjopen-2020-046476)
Supplement: Supplementary data [file bmjopen-2020-046476supp008.pdf]

Appendix 8 – meta-analysis of TCS versus TCI – cutaneous adverse events

1) Skin thinning

Study or Subgroup	Corticosteroid (TCS) Events	Corticosteroid (TCS) Total	Calcineurin inhibitor (TCI) Events	Calcineurin inhibitor (TCI) Total	Weight	Risk Ratio M-H, Random, 95% CI
Luger 2004 (1)	3	330	0	328	26.5%	6.96 [0.36, 134.17]
Mandelin 2010 (2)	2	40	0	40	25.7%	5.00 [0.25, 100.97]
Reitamo 2005 (3)	2	485	0	487	25.2%	5.02 [0.24, 104.30]
Sigurgeirsson 2015 (4)	1	1213	0	1205	22.7%	2.98 [0.12, 73.08]
**Total (95% CI)**		**2068**		**2060**	**100.0%**	**4.86 [1.06, 22.28]**
Total events	8		0			

Heterogeneity: Tau² = 0.00; Chi² = 0.15, df = 3 (P = 0.99); I² = 0%
Test for overall effect: Z = 2.03 (P = 0.04)

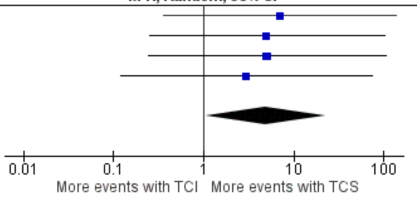

2) Skin burning

Study or Subgroup	Corticosteroid (TCS) Events	Corticosteroid (TCS) Total	Calcineurin inhibitor (TCI) Events	Calcineurin inhibitor (TCI) Total	Weight	Risk Ratio M-H, Random, 95% CI
Doss 2009 (5)	9	279	47	287	9.1%	0.20 [0.10, 0.39]
Doss 2010 (6)	6	239	18	237	6.4%	0.33 [0.13, 0.82]
Hofman 2006 (7)	2	124	10	133	2.9%	0.21 [0.05, 0.96]
Luger 2001 (8)	4	42	22	45	5.8%	0.19 [0.07, 0.52]
Luger 2004 (1)	36	330	85	328	15.6%	0.42 [0.29, 0.60]
Reitamo 2002(I) (9)	24	186	113	191	14.9%	0.22 [0.15, 0.32]
Reitamo 2002(II) (10)	13	185	38	186	10.7%	0.34 [0.19, 0.62]
Reitamo 2004 (11)	30	207	50	210	14.5%	0.61 [0.40, 0.92]
Reitamo 2005 (3)	67	485	255	487	18.4%	0.26 [0.21, 0.33]
Sikder 2005 (12)	1	15	7	15	1.8%	0.14 [0.02, 1.02]
**Total (95% CI)**		**2092**		**2119**	**100.0%**	**0.31 [0.23, 0.40]**
Total events	192		645			

Heterogeneity: Tau² = 0.09; Chi² = 21.66, df = 9 (P = 0.01); I² = 58%
Test for overall effect: Z = 8.44 (P < 0.00001)

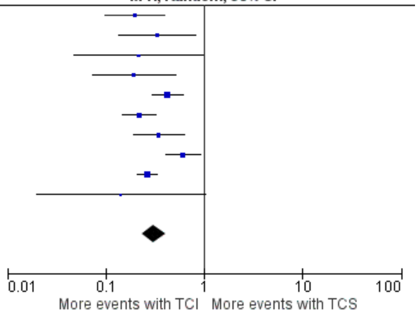

3) Pruritus

Study or Subgroup	Corticosteroid (TCS) Events	Corticosteroid (TCS) Total	Calcineurin inhibitor (TCI) Events	Calcineurin inhibitor (TCI) Total	Weight	Risk Ratio M-H, Random, 95% CI
Doss 2009 (5)	9	279	12	287	4.7%	0.77 [0.33, 1.80]
Doss 2010 (6)	8	239	10	237	4.1%	0.79 [0.32, 1.98]
Hofman 2006 (7)	4	124	8	133	2.5%	0.54 [0.17, 1.74]
Luger 2001 (8)	5	42	14	45	3.9%	0.38 [0.15, 0.97]
Luger 2004 (1)	6	330	18	328	4.1%	0.33 [0.13, 0.82]
Reitamo 2002(I) (9)	18	186	29	191	11.2%	0.64 [0.37, 1.11]
Reitamo 2002(II) (10)	14	185	21	186	8.2%	0.67 [0.35, 1.28]
Reitamo 2004 (11)	33	207	45	210	20.7%	0.74 [0.50, 1.12]
Reitamo 2005 (3)	65	485	88	487	39.3%	0.74 [0.55, 1.00]
Sikder 2005 (12)	2	15	3	15	1.3%	0.67 [0.13, 3.44]
**Total (95% CI)**		**2092**		**2119**	**100.0%**	**0.68 [0.56, 0.82]**
Total events	164		248			

Heterogeneity: Tau² = 0.00; Chi² = 4.80, df = 9 (P = 0.85); I² = 0%
Test for overall effect: Z = 4.11 (P < 0.0001)

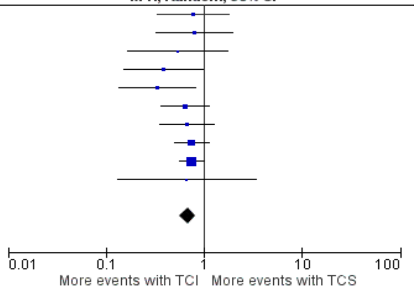

4) Skin infections

Study or Subgroup	Corticosteroid (TCS) Events	Corticosteroid (TCS) Total	Calcineurin inhibitor (TCI) Events	Calcineurin inhibitor (TCI) Total	Weight	Risk Ratio M-H, Random, 95% CI
Doss 2010 (6)	49	239	44	239	14.6%	1.11 [0.77, 1.60]
Hofman 2006 (7)	4	124	2	133	0.7%	2.15 [0.40, 11.51]
Luger 2004 (1)	80	330	69	328	24.2%	1.15 [0.87, 1.53]
Mandelin 2010 (2)	17	40	26	40	10.7%	0.65 [0.43, 1.00]
Reitamo 2002(II) (10)	4	185	4	186	1.0%	1.01 [0.26, 3.96]
Reitamo 2004 (11)	6	207	6	210	1.6%	1.01 [0.33, 3.09]
Reitamo 2005 (3)	9	485	13	487	2.8%	0.70 [0.30, 1.61]
Sigurgeirsson 2015 (4)	150	1213	157	1205	44.5%	0.95 [0.77, 1.17]
**Total (95% CI)**		**2823**		**2828**	**100.0%**	**0.98 [0.85, 1.12]**
Total events	319		321			

Heterogeneity: Tau² = 0.00; Chi² = 6.80, df = 7 (P = 0.45); I² = 0%
Test for overall effect: Z = 0.33 (P = 0.74)

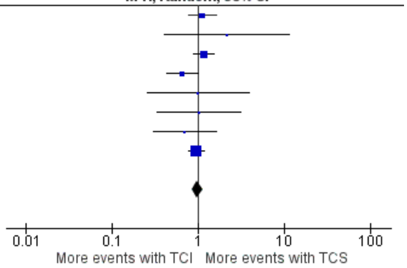

References:

1. Luger T, Lahfa M, Folster-Holst R, Gulliver W, Allen R, Molloy S, et al. Long-term safety and tolerability of pimecrolimus cream 1% and topical corticosteroids in adults with moderate to severe atopic dermatitis. *J Dermatolog Treat*. 2004;**15**(3):169-78
2. Mandelin J, Remitz A, Virtanen H, Reitamo S. One-year treatment with 0.1% tacrolimus ointment versus a corticosteroid regimen in adults with moderate to severe atopic dermatitis: A randomized, double-blind, comparative trial. *Acta Derm Venereol*. 2010;**90**(2):170-4
3. Reitamo S, Ortonne J, Sand C, Cambazard F, Bieber T, Folster-Holst R, et al. A multicentre, randomized, double-blind, controlled study of long-term treatment with 0.1% tacrolimus ointment in adults with moderate to severe atopic dermatitis. *Br J Dermatol*. 2005;**152**(6):1282-9
4. Sigurgeirsson B, Boznanski A, Todd G, Vertruyen A, Schuttelaar M, Zhu X, et al. Safety and efficacy of pimecrolimus in atopic dermatitis: a 5-year randomized trial. *Pediatrics* 2015;**135**(4):597-606
5. Doss N, Reitamo S, Dubertret L, Fekete G, Kamoun M, Lahfa M, et al. Superiority of tacrolimus 0.1% ointment compared with fluticasone 0.005% in adults with moderate to severe atopic dermatitis of the face: results from a randomized, double-blind trial. *Br J Dermatol*. 2009;**161**(2):427-34
6. Doss N, Kamoun M, Dubertret L, Cambazard F, Remitz A, Lahfa M, et al. Efficacy of tacrolimus 0.03% ointment as second-line treatment for children with moderate-to-severe atopic dermatitis: evidence from a randomized, double-blind non-inferiority trial vs. fluticasone 0.005% ointment. *Pediatr Allergy Immunol*. 2010;**21**(2 Pt 1):321-9.
7. Hofman T, Cranswick N, Kuna P, Boznanski A, Latos T, Gold M, et al. Tacrolimus ointment does not affect the immediate response to vaccination, the generation of immune memory, or humoral and cell-mediated immunity in children. *Arch Dis Child*. 2006;**91**(11):905-10
8. Luger T, Van Leent E, Graeber M, Hedgecock S, Thurston M, Kandra A, et al. SDZ ASM 981: an emerging safe and effective treatment for atopic dermatitis. *Br J Dermatol*. 2001;**144**(4):788-94
9. Reitamo S, Rustin M, Ruzicka T, Cambazard F, Kalimo K, Friedmann P, et al. Efficacy and safety of tacrolimus ointment compared with that of hydrocortisone butyrate ointment in adult patients with atopic dermatitis. *J Allergy Clin Immunol*. 2002;**109(**3):547-55
10. Reitamo S, Van Leent E, Ho V, Harper J, Ruzicka T, Kalimo K, et al. Efficacy and safety of tacrolimus ointment compared with that of hydrocortisone acetate ointment in children with atopic dermatitis. *J Allergy Clin Immunol*. 2002;**109**(3):539-46
11. Reitamo S, Harper J, Bos J, Cambazard F, Bruijnzeel-Koomen C, Valk P, et al. 0.03% Tacrolimus ointment applied once or twice daily is more efficacious than 1% hydrocortisone acetate in children with moderate to severe atopic dermatitis: results of a randomized double-blind controlled trial. *Br J Dermatol*. 2004;**150**(3):554-62
12. Sikder M, Al Mamun S, Khan R, Chowdhury A, Khan H, Hoque M. Topical 0.03% tacrolimus ointment, 0.05% clobetasone butyrate cream alone and their combination in older children with atopic dermatitis - An open randomized comparative study. *J Pak Assoc Dermatol*. 2005;**15**(4):304-12
