# Supplementary material for: Safety of topical corticosteroids in atopic eczema: an umbrella review
Source: BMJ Open. 2021 Jul 7;11(7):e046476. doi: 10.1136/bmjopen-2020-046476 (PMC8264889; doi:10.1136/bmjopen-2020-046476)
Supplement: Supplementary data [file bmjopen-2020-046476supp009.pdf]

Appendix 9: subgroup analyses of TCS versus TCI – cutaneous adverse events

By different topical corticosteroid potencies

Skin thinning

Study or Subgroup	Corticosteroids (TCS) Events	Total	Calcineurin inhibitor (TCI) Events	Total	Weight	Risk Ratio M-H, Random, 95% CI
**2.6.1 Potent**						
Luger 2004 (1)	3	330	0	328	26.5%	6.96 [0.36, 134.17]
Mandelin 2010 (2)	2	40	0	40	25.7%	5.00 [0.25, 100.97]
Reitamo 2005 (3)	2	485	0	487	25.2%	5.02 [0.24, 104.30]
**Subtotal (95% CI)**		**855**		**855**	**77.3%**	**5.61 [0.99, 31.67]**
Total events	7		0			
Heterogeneity: Tau² = 0.00; Chi² = 0.03, df = 2 (P = 0.98); I² = 0%						
Test for overall effect: Z = 1.95 (P = 0.05)						
**2.6.2 Moderate or mild potency**						
Sigurgeirsson 2015 (4)	1	1213	0	1205	22.7%	2.98 [0.12, 73.08]
**Subtotal (95% CI)**		**1213**		**1205**	**22.7%**	**2.98 [0.12, 73.08]**
Total events	1		0			
Heterogeneity: Not applicable						
Test for overall effect: Z = 0.67 (P = 0.50)						
**Total (95% CI)**		**2068**		**2060**	**100.0%**	**4.86 [1.06, 22.28]**
Total events	8		0			
Heterogeneity: Tau² = 0.00; Chi² = 0.15, df = 3 (P = 0.99); I² = 0%						
Test for overall effect: Z = 2.03 (P = 0.04)						
Test for subgroup differences: Chi² = 0.12, df = 1 (P = 0.73), I² = 0%						

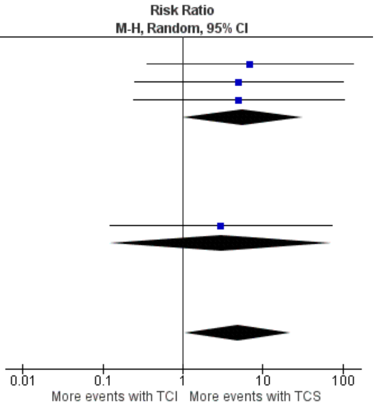

Skin burning

Study or Subgroup	Corticosteroids (TCS) Events	Total	Calcineurin inhibitor (TCI) Events	Total	Weight	Risk Ratio M-H, Random, 95% CI
**2.2.1 Potent**						
Doss 2009 (5)	9	279	47	287	9.1%	0.20 [0.10, 0.39]
Doss 2010 (6)	6	239	18	237	6.4%	0.33 [0.13, 0.82]
Luger 2001 (7)	4	42	22	45	5.8%	0.19 [0.07, 0.52]
Luger 2004 (1)	36	330	85	328	15.6%	0.42 [0.29, 0.60]
Reitamo 2002(I) (8)	24	186	113	191	14.9%	0.22 [0.15, 0.32]
Reitamo 2005 (3)	67	485	255	487	18.4%	0.26 [0.21, 0.33]
**Subtotal (95% CI)**		**1561**		**1575**	**70.2%**	**0.27 [0.21, 0.35]**
Total events	146		540			
Heterogeneity: Tau² = 0.04; Chi² = 8.42, df = 5 (P = 0.13); I² = 41%						
Test for overall effect: Z = 10.11 (P < 0.00001)						
**2.2.2 Potent or mild potency**						
Hofman 2006 (9)	2	124	10	133	2.9%	0.21 [0.05, 0.96]
**Subtotal (95% CI)**		**124**		**133**	**2.9%**	**0.21 [0.05, 0.96]**
Total events	2		10			
Heterogeneity: Not applicable						
Test for overall effect: Z = 2.01 (P = 0.04)						
**2.2.3 Moderate potency**						
Sikder 2005 (10)	1	15	7	15	1.8%	0.14 [0.02, 1.02]
**Subtotal (95% CI)**		**15**		**15**	**1.8%**	**0.14 [0.02, 1.02]**
Total events	1		7			
Heterogeneity: Not applicable						
Test for overall effect: Z = 1.94 (P = 0.05)						
**2.2.4 Mild potency**						
Reitamo 2002(II) (11)	13	185	38	186	10.7%	0.34 [0.19, 0.62]
Reitamo 2004 (12)	30	207	50	210	14.5%	0.61 [0.40, 0.92]
**Subtotal (95% CI)**		**392**		**396**	**25.1%**	**0.48 [0.27, 0.83]**
Total events	43		88			
Heterogeneity: Tau² = 0.10; Chi² = 2.41, df = 1 (P = 0.12); I² = 59%						
Test for overall effect: Z = 2.61 (P = 0.009)						
**Total (95% CI)**		**2092**		**2119**	**100.0%**	**0.31 [0.23, 0.40]**
Total events	192		645			
Heterogeneity: Tau² = 0.09; Chi² = 21.66, df = 9 (P = 0.01); I² = 58%						
Test for overall effect: Z = 8.44 (P < 0.00001)						
Test for subgroup differences: Chi² = 3.92, df = 3 (P = 0.27), I² = 23.4%						

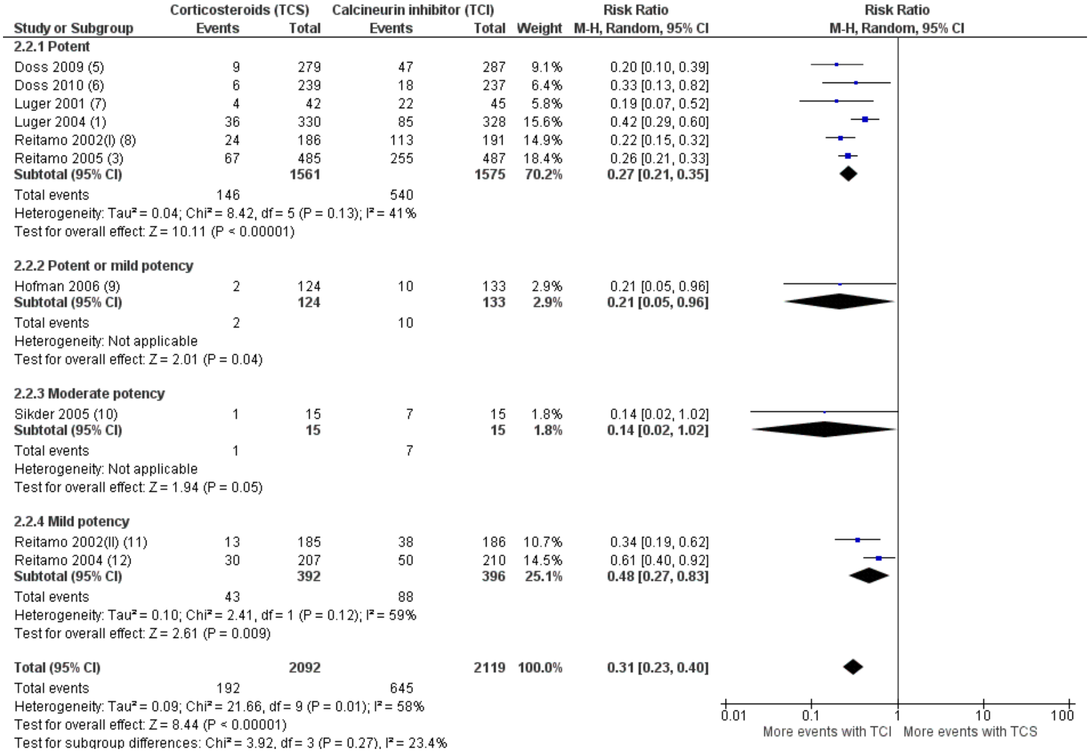

Pruritus

	Corticosteroids (TCS)		Calcineurin inhibitor (TCI)			Risk Ratio
**Study or Subgroup**	**Events**	**Total**	**Events**	**Total**	**Weight**	**M-H, Random, 95% CI**
**2.3.1 Potent**						
Doss 2009 (5)	9	279	12	287	4.7%	0.77 [0.33, 1.80]
Doss 2010 (6)	8	239	10	237	4.1%	0.79 [0.32, 1.98]
Luger 2001 (7)	5	42	14	45	3.9%	0.38 [0.15, 0.97]
Luger 2004 (1)	6	330	18	328	4.1%	0.33 [0.13, 0.82]
Reitamo 2002(I) (8)	18	186	29	191	11.2%	0.64 [0.37, 1.11]
Reitamo 2005 (3)	65	485	88	487	39.3%	0.74 [0.55, 1.00]
**Subtotal (95% CI)**		**1561**		**1575**	**67.4%**	**0.67 [0.53, 0.84]**
Total events	111		171			
Heterogeneity: Tau² = 0.00; Chi² = 4.43, df = 5 (P = 0.49); I² = 0%						
Test for overall effect: Z = 3.53 (P = 0.0004)						
**2.3.2 Potent or mild potency**						
Hofman 2006 (9)	4	124	8	133	2.5%	0.54 [0.17, 1.74]
**Subtotal (95% CI)**		**124**		**133**	**2.5%**	**0.54 [0.17, 1.74]**
Total events	4		8			
Heterogeneity: Not applicable						
Test for overall effect: Z = 1.04 (P = 0.30)						
**2.3.3 Moderate potency**						
Sikder 2005 (10)	2	15	3	15	1.3%	0.67 [0.13, 3.44]
**Subtotal (95% CI)**		**15**		**15**	**1.3%**	**0.67 [0.13, 3.44]**
Total events	2		3			
Heterogeneity: Not applicable						
Test for overall effect: Z = 0.48 (P = 0.63)						
**2.3.4 Mild potency**						
Reitamo 2002(II) (11)	14	185	21	186	8.2%	0.67 [0.35, 1.28]
Reitamo 2004 (12)	33	207	45	210	20.7%	0.74 [0.50, 1.12]
**Subtotal (95% CI)**		**392**		**396**	**28.9%**	**0.72 [0.51, 1.02]**
Total events	47		66			
Heterogeneity: Tau² = 0.00; Chi² = 0.07, df = 1 (P = 0.79); I² = 0%						
Test for overall effect: Z = 1.86 (P = 0.06)						
**Total (95% CI)**		**2092**		**2119**	**100.0%**	**0.68 [0.56, 0.82]**
Total events	164		248			
Heterogeneity: Tau² = 0.00; Chi² = 4.80, df = 9 (P = 0.85); I² = 0%						
Test for overall effect: Z = 4.11 (P < 0.0001)						
Test for subgroup differences: Chi² = 0.30, df = 3 (P = 0.96), I² = 0%						

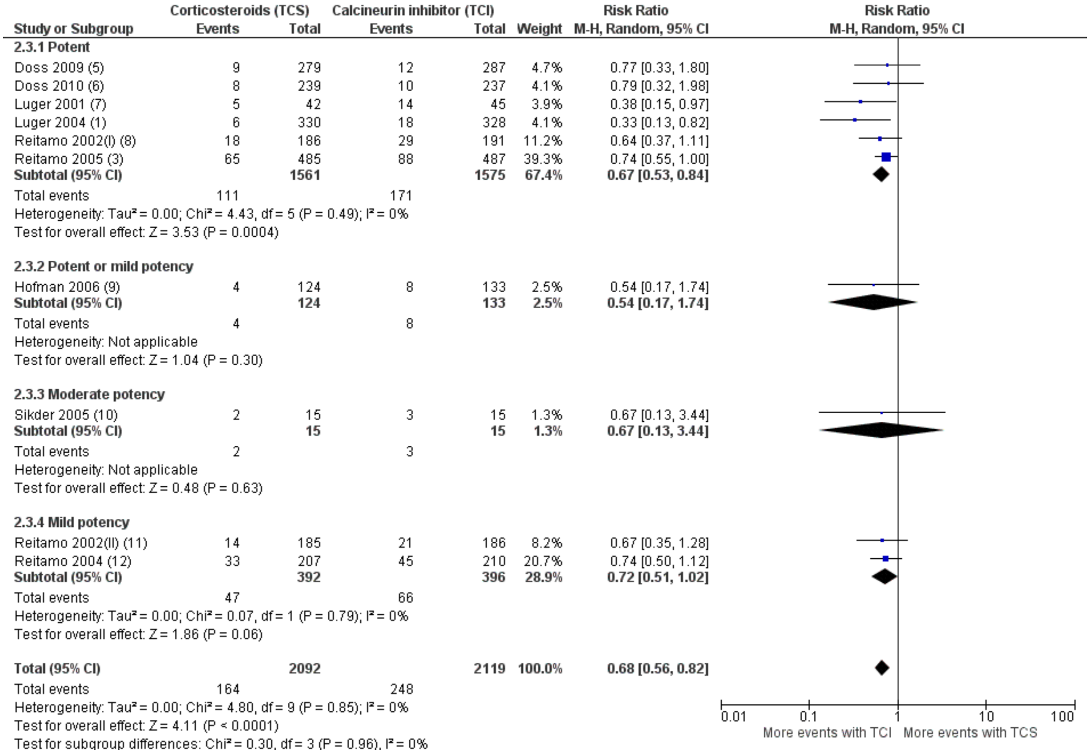

Skin infections

	Corticosteroids (TCS)		Calcineurin inhibitor (TCI)			Risk Ratio
**Study or Subgroup**	**Events**	**Total**	**Events**	**Total**	**Weight**	**M-H, Random, 95% CI**
**2.7.1 Potent**						
Doss 2010 (6)	49	239	44	239	14.6%	1.11 [0.77, 1.60]
Luger 2004 (1)	80	330	69	328	24.2%	1.15 [0.87, 1.53]
Mandelin 2010 (2)	17	40	26	40	10.7%	0.65 [0.43, 1.00]
Reitamo 2005 (3)	9	485	13	487	2.8%	0.70 [0.30, 1.61]
**Subtotal (95% CI)**		**1094**		**1094**	**52.2%**	**0.94 [0.70, 1.26]**
Total events	155		152			
Heterogeneity: Tau² = 0.04; Chi² = 5.90, df = 3 (P = 0.12); I² = 49%						
Test for overall effect: Z = 0.41 (P = 0.68)						
**2.7.2 Potent or mild potency**						
Hofman 2006 (9)	4	124	2	133	0.7%	2.15 [0.40, 11.51]
**Subtotal (95% CI)**		**124**		**133**	**0.7%**	**2.15 [0.40, 11.51]**
Total events	4		2			
Heterogeneity: Not applicable						
Test for overall effect: Z = 0.89 (P = 0.37)						
**2.7.3 Moderate or mild potency**						
Sigurgeirsson 2015 (4)	150	1213	157	1205	44.5%	0.95 [0.77, 1.17]
**Subtotal (95% CI)**		**1213**		**1205**	**44.5%**	**0.95 [0.77, 1.17]**
Total events	150		157			
Heterogeneity: Not applicable						
Test for overall effect: Z = 0.49 (P = 0.62)						
**2.7.4 Mild potency**						
Reitamo 2002(II) (11)	4	185	4	186	1.0%	1.01 [0.26, 3.96]
Reitamo 2004 (12)	6	207	6	210	1.6%	1.01 [0.33, 3.09]
**Subtotal (95% CI)**		**392**		**396**	**2.6%**	**1.01 [0.43, 2.40]**
Total events	10		10			
Heterogeneity: Tau² = 0.00; Chi² = 0.00, df = 1 (P = 0.99); I² = 0%						
Test for overall effect: Z = 0.02 (P = 0.98)						
**Total (95% CI)**		**2823**		**2828**	**100.0%**	**0.98 [0.85, 1.12]**
Total events	319		321			
Heterogeneity: Tau² = 0.00; Chi² = 6.80, df = 7 (P = 0.45); I² = 0%						
Test for overall effect: Z = 0.33 (P = 0.74)						
Test for subgroup differences: Chi² = 0.92, df = 3 (P = 0.82), I² = 0%						

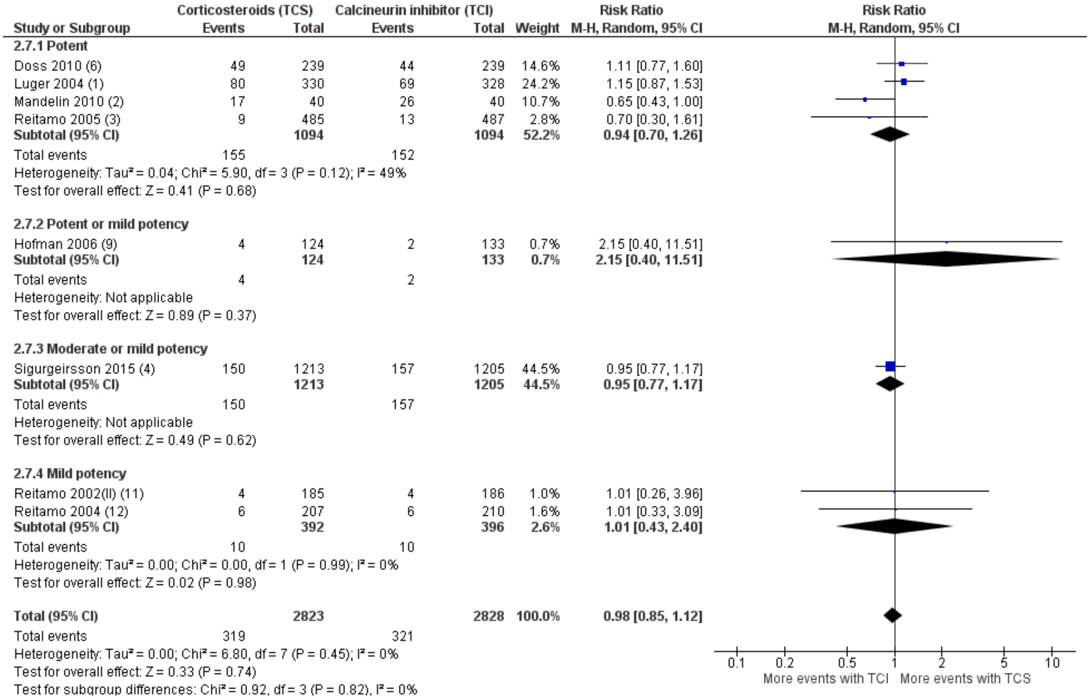

By age of participants (children or adults)

Skin thinning

Study or Subgroup	Corticosteroids (TCS) Events	Corticosteroids (TCS) Total	Calcineurin inhibitor (TCI) Events	Calcineurin inhibitor (TCI) Total	Weight	Risk Ratio M-H, Random, 95% CI
**3.6.1 Adults**						
Luger 2004 (1)	3	330	0	328	26.5%	6.96 [0.36, 134.17]
Mandelin 2010 (2)	2	40	0	40	25.7%	5.00 [0.25, 100.97]
Reitamo 2005 (3)	2	485	0	487	25.2%	5.02 [0.24, 104.30]
**Subtotal (95% CI)**		**855**		**855**	**77.3%**	**5.61 [0.99, 31.67]**
Total events	7		0			
Heterogeneity: Tau² = 0.00; Chi² = 0.03, df = 2 (P = 0.98); I² = 0%						
Test for overall effect: Z = 1.95 (P = 0.05)						
**3.6.2 Children**						
Sigurgeirsson 2015 (4)	1	1213	0	1205	22.7%	2.98 [0.12, 73.08]
**Subtotal (95% CI)**		**1213**		**1205**	**22.7%**	**2.98 [0.12, 73.08]**
Total events	1		0			
Heterogeneity: Not applicable						
Test for overall effect: Z = 0.67 (P = 0.50)						
**Total (95% CI)**		**2068**		**2060**	**100.0%**	**4.86 [1.06, 22.28]**
Total events	8		0			
Heterogeneity: Tau² = 0.00; Chi² = 0.15, df = 3 (P = 0.99); I² = 0%						
Test for overall effect: Z = 2.03 (P = 0.04)						
Test for subgroup differences: Chi² = 0.12, df = 1 (P = 0.73), I² = 0%						

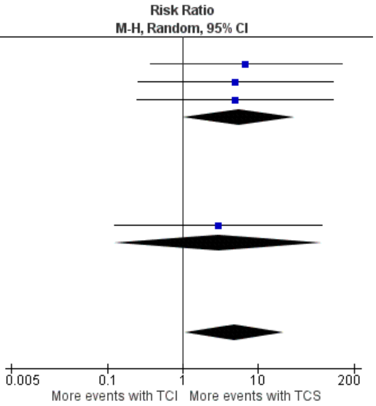

Skin burning

Study or Subgroup	Corticosteroids (TCS) Events	Corticosteroids (TCS) Total	Calcineurin inhibitor (TCI) Events	Calcineurin inhibitor (TCI) Total	Weight	Risk Ratio M-H, Random, 95% CI
**3.2.1 Adults**						
Doss 2009 (5)	9	279	47	287	9.1%	0.20 [0.10, 0.39]
Luger 2001 (7)	4	42	22	45	5.8%	0.19 [0.07, 0.52]
Luger 2004 (1)	36	330	85	328	15.6%	0.42 [0.29, 0.60]
Reitamo 2002(I) (8)	24	186	113	191	14.9%	0.22 [0.15, 0.32]
Reitamo 2005 (3)	67	485	255	487	18.4%	0.26 [0.21, 0.33]
**Subtotal (95% CI)**		**1322**		**1338**	**63.7%**	**0.27 [0.20, 0.36]**
Total events	140		522			
Heterogeneity: Tau² = 0.05; Chi² = 8.28, df = 4 (P = 0.08); I² = 52%						
Test for overall effect: Z = 9.17 (P < 0.00001)						
**3.2.2 Children**						
Doss 2010 (6)	6	239	18	237	6.4%	0.33 [0.13, 0.82]
Hofman 2006 (9)	2	124	10	133	2.9%	0.21 [0.05, 0.96]
Reitamo 2002(II) (11)	13	185	38	186	10.7%	0.34 [0.19, 0.62]
Reitamo 2004 (12)	30	207	50	210	14.5%	0.61 [0.40, 0.92]
Sikder 2005 (10)	1	15	7	15	1.8%	0.14 [0.02, 1.02]
**Subtotal (95% CI)**		**770**		**781**	**36.3%**	**0.40 [0.26, 0.62]**
Total events	52		123			
Heterogeneity: Tau² = 0.07; Chi² = 5.68, df = 4 (P = 0.22); I² = 30%						
Test for overall effect: Z = 4.17 (P < 0.0001)						
**Total (95% CI)**		**2092**		**2119**	**100.0%**	**0.31 [0.23, 0.40]**
Total events	192		645			
Heterogeneity: Tau² = 0.09; Chi² = 21.66, df = 9 (P = 0.01); I² = 58%						
Test for overall effect: Z = 8.44 (P < 0.00001)						
Test for subgroup differences: Chi² = 2.48, df = 1 (P = 0.11), I² = 59.8%						

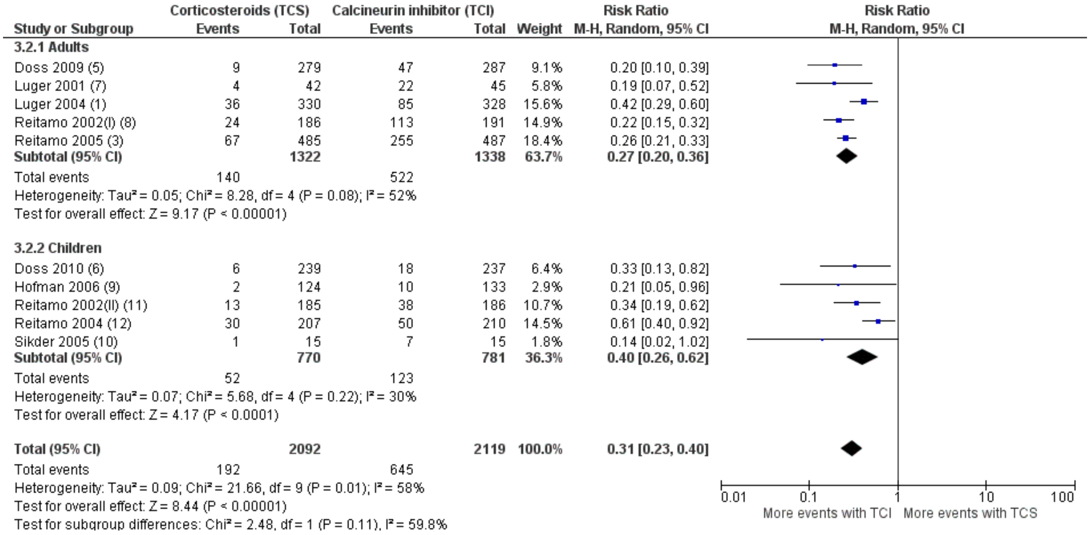

Pruritus

Study or Subgroup	Corticosteroids (TCS) Events	Total	Calcineurin inhibitor (TCI) Events	Total	Weight	Risk Ratio M-H, Random, 95% CI
**3.3.1 Adults**						
Doss 2009 (5)	9	279	12	287	4.7%	0.77 [0.33, 1.80]
Luger 2001 (7)	5	42	14	45	3.9%	0.38 [0.15, 0.97]
Luger 2004 (1)	6	330	18	328	4.1%	0.33 [0.13, 0.82]
Reitamo 2002(I) (8)	18	186	29	191	11.2%	0.64 [0.37, 1.11]
Reitamo 2005 (3)	65	485	88	487	39.3%	0.74 [0.55, 1.00]
**Subtotal (95% CI)**		**1322**		**1338**	**63.2%**	**0.65 [0.50, 0.84]**
Total events	103		161			
Heterogeneity: Tau² = 0.01; Chi² = 4.28, df = 4 (P = 0.37); I² = 7%						
Test for overall effect: Z = 3.34 (P = 0.0008)						
**3.3.2 Children**						
Doss 2010 (6)	8	239	10	237	4.1%	0.79 [0.32, 1.98]
Hofman 2006 (9)	4	124	8	133	2.5%	0.54 [0.17, 1.74]
Reitamo 2002(II) (11)	14	185	21	186	8.2%	0.67 [0.35, 1.28]
Reitamo 2004 (12)	33	207	45	210	20.7%	0.74 [0.50, 1.12]
Sikder 2005 (10)	2	15	3	15	1.3%	0.67 [0.13, 3.44]
**Subtotal (95% CI)**		**770**		**781**	**36.8%**	**0.71 [0.53, 0.97]**
Total events	61		87			
Heterogeneity: Tau² = 0.00; Chi² = 0.36, df = 4 (P = 0.99); I² = 0%						
Test for overall effect: Z = 2.17 (P = 0.03)						
**Total (95% CI)**		**2092**		**2119**	**100.0%**	**0.68 [0.56, 0.82]**
Total events	164		248			
Heterogeneity: Tau² = 0.00; Chi² = 4.80, df = 9 (P = 0.85); I² = 0%						
Test for overall effect: Z = 4.11 (P < 0.0001)						
Test for subgroup differences: Chi² = 0.23, df = 1 (P = 0.63), I² = 0%						

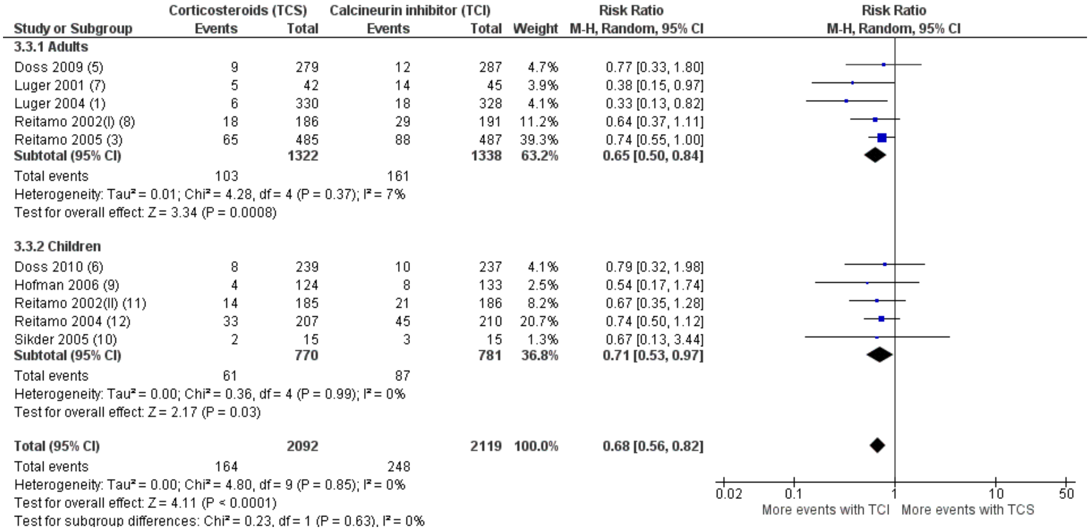

Skin infections

Study or Subgroup	Corticosteroids (TCS) Events	Total	Calcineurin inhibitor (TCI) Events	Total	Weight	Risk Ratio M-H, Random, 95% CI
**3.7.1 Adults**						
Luger 2004 (1)	80	330	69	328	24.2%	1.15 [0.87, 1.53]
Mandelin 2010 (2)	17	40	26	40	10.7%	0.65 [0.43, 1.00]
Reitamo 2005 (3)	9	485	13	487	2.8%	0.70 [0.30, 1.61]
**Subtotal (95% CI)**		**855**		**855**	**37.7%**	**0.85 [0.55, 1.32]**
Total events	106		108			
Heterogeneity: Tau² = 0.09; Chi² = 5.35, df = 2 (P = 0.07); I² = 63%						
Test for overall effect: Z = 0.70 (P = 0.48)						
**3.7.2 Children**						
Doss 2010 (6)	49	239	44	239	14.6%	1.11 [0.77, 1.60]
Hofman 2006 (9)	4	124	2	133	0.7%	2.15 [0.40, 11.51]
Reitamo 2002(II) (11)	4	185	4	186	1.0%	1.01 [0.26, 3.96]
Reitamo 2004 (12)	6	207	6	210	1.6%	1.01 [0.33, 3.09]
Sigurgeirsson 2015 (4)	150	1213	157	1205	44.5%	0.95 [0.77, 1.17]
**Subtotal (95% CI)**		**1968**		**1973**	**62.3%**	**1.00 [0.84, 1.19]**
Total events	213		213			
Heterogeneity: Tau² = 0.00; Chi² = 1.37, df = 4 (P = 0.85); I² = 0%						
Test for overall effect: Z = 0.04 (P = 0.97)						
**Total (95% CI)**		**2823**		**2828**	**100.0%**	**0.98 [0.85, 1.12]**
Total events	319		321			
Heterogeneity: Tau² = 0.00; Chi² = 6.80, df = 7 (P = 0.45); I² = 0%						
Test for overall effect: Z = 0.33 (P = 0.74)						
Test for subgroup differences: Chi² = 0.41, df = 1 (P = 0.52), I² = 0%						

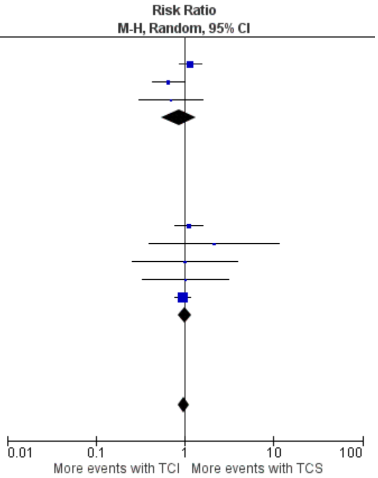

By individual topical calcineurin inhibitor (TCI)

Skin thinning

Study or Subgroup	Corticosteroids (TCS) Events	Corticosteroids (TCS) Total	Calcineurin inhibitor (TCI) Events	Calcineurin inhibitor (TCI) Total	Weight	Risk Ratio M-H, Random, 95% CI
**4.6.1 Tacrolimus 0.1%**						
Mandelin 2010 (2)	2	40	0	40	25.7%	5.00 [0.25, 100.97]
Reitamo 2005 (3)	2	485	0	487	25.2%	5.02 [0.24, 104.30]
**Subtotal (95% CI)**		**525**		**527**	**50.9%**	**5.01 [0.59, 42.37]**
Total events	4		0			
Heterogeneity: Tau² = 0.00; Chi² = 0.00, df = 1 (P = 1.00); I² = 0%						
Test for overall effect: Z = 1.48 (P = 0.14)						
**4.6.2 Pimecrolimus 1%**						
Luger 2004 (1)	3	330	0	328	26.5%	6.96 [0.36, 134.17]
Sigurgeirsson 2015 (4)	1	1213	0	1205	22.7%	2.98 [0.12, 73.08]
**Subtotal (95% CI)**		**1543**		**1533**	**49.1%**	**4.71 [0.54, 41.33]**
Total events	4		0			
Heterogeneity: Tau² = 0.00; Chi² = 0.15, df = 1 (P = 0.70); I² = 0%						
Test for overall effect: Z = 1.40 (P = 0.16)						
**Total (95% CI)**		**2068**		**2060**	**100.0%**	**4.86 [1.06, 22.28]**
Total events	8		0			
Heterogeneity: Tau² = 0.00; Chi² = 0.15, df = 3 (P = 0.99); I² = 0%						
Test for overall effect: Z = 2.03 (P = 0.04)						
Test for subgroup differences: Chi² = 0.00, df = 1 (P = 0.97), I² = 0%						

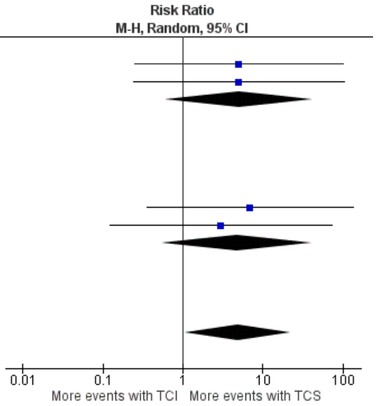

Skin burning

Study or Subgroup	Corticosteroids (TCS) Events	Corticosteroids (TCS) Total	Calcineurin inhibitor (TCI) Events	Calcineurin inhibitor (TCI) Total	Weight	Risk Ratio M-H, Random, 95% CI
**4.2.1 Tacrolimus 0.1%**						
Doss 2009 (5)	9	279	47	287	9.1%	0.20 [0.10, 0.39]
Reitamo 2002(I) (8)	24	186	113	191	14.9%	0.22 [0.15, 0.32]
Reitamo 2002(II) (11)	13	185	38	186	10.7%	0.34 [0.19, 0.62]
Reitamo 2005 (3)	67	485	255	487	18.4%	0.26 [0.21, 0.33]
**Subtotal (95% CI)**		**1135**		**1151**	**53.0%**	**0.25 [0.21, 0.31]**
Total events	113		453			
Heterogeneity: Tau² = 0.00; Chi² = 2.19, df = 3 (P = 0.53); I² = 0%						
Test for overall effect: Z = 14.49 (P < 0.00001)						
**4.2.2 Tacrolimus 0.03%**						
Doss 2010 (6)	6	239	18	237	6.4%	0.33 [0.13, 0.82]
Hofman 2006 (9)	2	124	10	133	2.9%	0.21 [0.05, 0.96]
Reitamo 2004 (12)	30	207	50	210	14.5%	0.61 [0.40, 0.92]
Sikder 2005 (10)	1	15	7	15	1.8%	0.14 [0.02, 1.02]
**Subtotal (95% CI)**		**585**		**595**	**25.6%**	**0.40 [0.22, 0.73]**
Total events	39		85			
Heterogeneity: Tau² = 0.13; Chi² = 4.60, df = 3 (P = 0.20); I² = 35%						
Test for overall effect: Z = 3.00 (P = 0.003)						
**4.2.3 Pimecrolimus 1%**						
Luger 2001 (7)	4	42	22	45	5.8%	0.19 [0.07, 0.52]
Luger 2004 (1)	36	330	85	328	15.6%	0.42 [0.29, 0.60]
**Subtotal (95% CI)**		**372**		**373**	**21.4%**	**0.33 [0.16, 0.67]**
Total events	40		107			
Heterogeneity: Tau² = 0.16; Chi² = 2.12, df = 1 (P = 0.15); I² = 53%						
Test for overall effect: Z = 3.08 (P = 0.002)						
**Total (95% CI)**		**2092**		**2119**	**100.0%**	**0.31 [0.23, 0.40]**
Total events	192		645			
Heterogeneity: Tau² = 0.09; Chi² = 21.66, df = 9 (P = 0.01); I² = 58%						
Test for overall effect: Z = 8.44 (P < 0.00001)						
Test for subgroup differences: Chi² = 2.43, df = 2 (P = 0.30), I² = 17.8%						

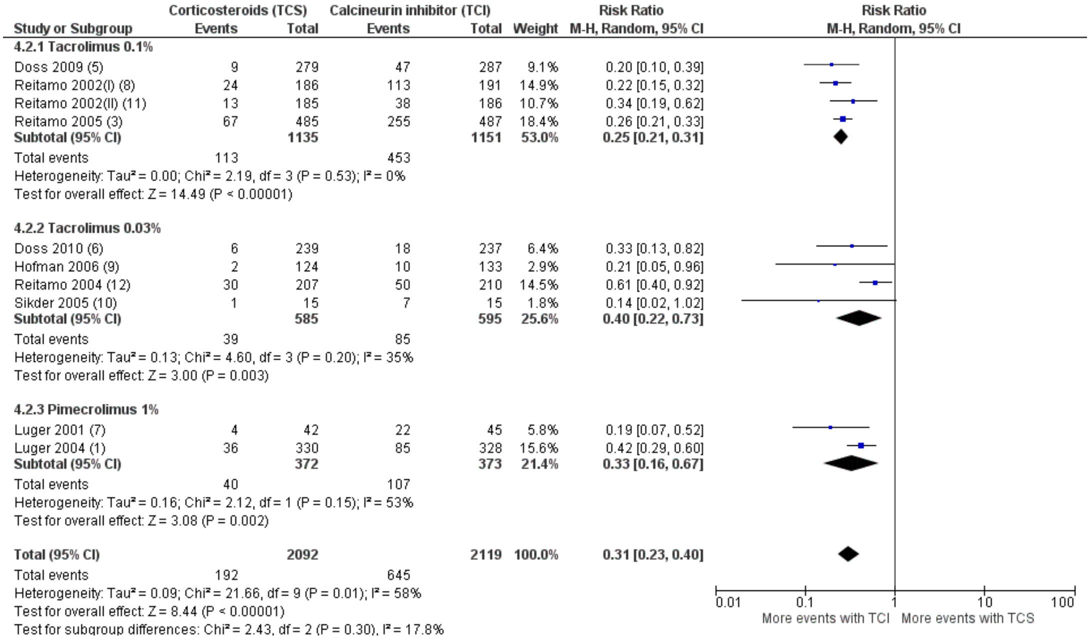

Pruritus

Study or Subgroup	Corticosteroids (TCS) Events	Total	Calcineurin inhibitor (TCI) Events	Total	Weight	Risk Ratio M-H, Random, 95% CI
**4.3.1 Tacrolimus 0.1%**						
Doss 2009 (5)	9	279	12	287	4.7%	0.77 [0.33, 1.80]
Reitamo 2002(I) (8)	18	186	29	191	11.2%	0.64 [0.37, 1.11]
Reitamo 2002(II) (11)	14	185	21	186	8.2%	0.67 [0.35, 1.28]
Reitamo 2005 (3)	65	485	88	487	39.3%	0.74 [0.55, 1.00]
**Subtotal (95% CI)**		**1135**		**1151**	**63.4%**	**0.71 [0.57, 0.90]**
Total events	106		150			
Heterogeneity: Tau² = 0.00; Chi² = 0.30, df = 3 (P = 0.96); I² = 0%						
Test for overall effect: Z = 2.84 (P = 0.005)						
**4.3.2 Tacrolimus 0.03%**						
Doss 2010 (6)	8	239	10	237	4.1%	0.79 [0.32, 1.98]
Hofman 2006 (9)	4	124	8	133	2.5%	0.54 [0.17, 1.74]
Reitamo 2004 (12)	33	207	45	210	20.7%	0.74 [0.50, 1.12]
Sikder 2005 (10)	2	15	3	15	1.3%	0.67 [0.13, 3.44]
**Subtotal (95% CI)**		**585**		**595**	**28.5%**	**0.73 [0.51, 1.03]**
Total events	47		66			
Heterogeneity: Tau² = 0.00; Chi² = 0.32, df = 3 (P = 0.96); I² = 0%						
Test for overall effect: Z = 1.81 (P = 0.07)						
**4.3.3 Pimecrolimus 1%**						
Luger 2001 (7)	5	42	14	45	3.9%	0.38 [0.15, 0.97]
Luger 2004 (1)	6	330	18	328	4.1%	0.33 [0.13, 0.82]
**Subtotal (95% CI)**		**372**		**373**	**8.1%**	**0.36 [0.19, 0.68]**
Total events	11		32			
Heterogeneity: Tau² = 0.00; Chi² = 0.05, df = 1 (P = 0.83); I² = 0%						
Test for overall effect: Z = 3.11 (P = 0.002)						
**Total (95% CI)**		**2092**		**2119**	**100.0%**	**0.68 [0.56, 0.82]**
Total events	164		248			
Heterogeneity: Tau² = 0.00; Chi² = 4.80, df = 9 (P = 0.85); I² = 0%						
Test for overall effect: Z = 4.11 (P < 0.0001)						
Test for subgroup differences: Chi² = 4.13, df = 2 (P = 0.13), I² = 51.6%						

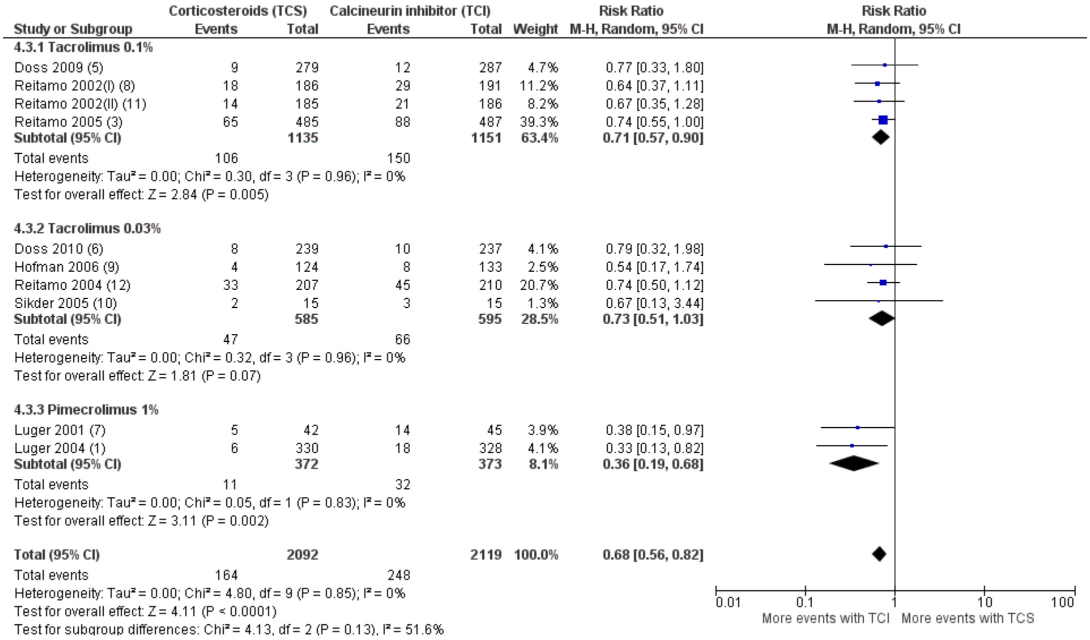

Skin infections

Study or Subgroup	Corticosteroids (TCS) Events	Total	Calcineurin inhibitor (TCI) Events	Total	Weight	Risk Ratio M-H, Random, 95% CI
**4.7.1 Tacrolimus 0.1%**						
Mandelin 2010 (2)	17	40	26	40	10.7%	0.65 [0.43, 1.00]
Reitamo 2002(II) (11)	4	185	4	186	1.0%	1.01 [0.26, 3.96]
Reitamo 2005 (3)	9	485	13	487	2.8%	0.70 [0.30, 1.61]
**Subtotal (95% CI)**		**710**		**713**	**14.5%**	**0.68 [0.47, 0.98]**
Total events	30		43			
Heterogeneity: Tau² = 0.00; Chi² = 0.36, df = 2 (P = 0.83); I² = 0%						
Test for overall effect: Z = 2.05 (P = 0.04)						
**4.7.2 Tacrolimus 0.03%**						
Doss 2010 (6)	49	239	44	239	14.6%	1.11 [0.77, 1.60]
Hofman 2006 (9)	4	124	2	133	0.7%	2.15 [0.40, 11.51]
Reitamo 2004 (12)	6	207	6	210	1.6%	1.01 [0.33, 3.09]
**Subtotal (95% CI)**		**570**		**582**	**16.8%**	**1.13 [0.81, 1.59]**
Total events	59		52			
Heterogeneity: Tau² = 0.00; Chi² = 0.60, df = 2 (P = 0.74); I² = 0%						
Test for overall effect: Z = 0.73 (P = 0.47)						
**4.7.3 Pimecrolimus 1%**						
Luger 2004 (1)	80	330	69	328	24.2%	1.15 [0.87, 1.53]
Sigurgeirsson 2015 (4)	150	1213	157	1205	44.5%	0.95 [0.77, 1.17]
**Subtotal (95% CI)**		**1543**		**1533**	**68.7%**	**1.02 [0.85, 1.23]**
Total events	230		226			
Heterogeneity: Tau² = 0.00; Chi² = 1.17, df = 1 (P = 0.28); I² = 15%						
Test for overall effect: Z = 0.22 (P = 0.83)						
**Total (95% CI)**		**2823**		**2828**	**100.0%**	**0.98 [0.85, 1.12]**
Total events	319		321			
Heterogeneity: Tau² = 0.00; Chi² = 6.80, df = 7 (P = 0.45); I² = 0%						
Test for overall effect: Z = 0.33 (P = 0.74)						
Test for subgroup differences: Chi² = 4.65, df = 2 (P = 0.10), I² = 57.0%						

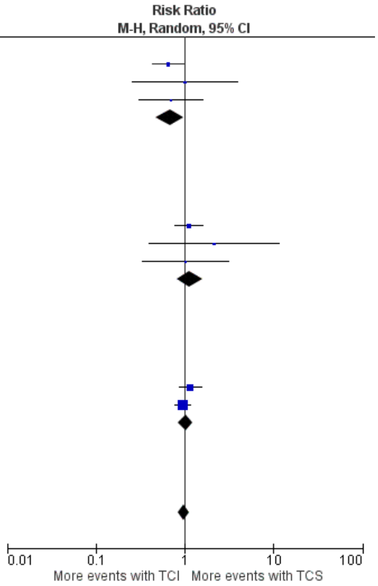

References:

1. Luger T, Lahfa M, Folster-Holst R, Gulliver W, Allen R, Molloy S, et al. Long-term safety and tolerability of pimecrolimus cream 1% and topical corticosteroids in adults with moderate to severe atopic dermatitis. *J Dermatolog Treat*. 2004;**15**(3):169-78
2. Mandelin J, Remitz A, Virtanen H, Reitamo S. One-year treatment with 0.1% tacrolimus ointment versus a corticosteroid regimen in adults with moderate to severe atopic dermatitis: A randomized, double-blind, comparative trial. *Acta Derm Venereol*. 2010;**90**(2):170-4
3. Reitamo S, Ortonne J, Sand C, Cambazard F, Bieber T, Folster-Holst R, et al. A multicentre, randomized, double-blind, controlled study of long-term treatment with 0.1% tacrolimus ointment in adults with moderate to severe atopic dermatitis. *Br J Dermatol*. 2005;**152**(6):1282-9
4. Sigurgeirsson B, Boznanski A, Todd G, Vertruyen A, Schuttelaar M, Zhu X, et al. Safety and efficacy of pimecrolimus in atopic dermatitis: a 5-year randomized trial. *Pediatrics* 2015;**135**(4):597-606
5. Doss N, Reitamo S, Dubertret L, Fekete G, Kamoun M, Lahfa M, et al. Superiority of tacrolimus 0.1% ointment compared with fluticasone 0.005% in adults with moderate to severe atopic dermatitis of the face: results from a randomized, double-blind trial. *Br J Dermatol*. 2009;**161**(2):427-34
6. Doss N, Kamoun M, Dubertret L, Cambazard F, Remitz A, Lahfa M, et al. Efficacy of tacrolimus 0.03% ointment as second-line treatment for children with moderate-to-severe atopic dermatitis: evidence from a randomized, double-blind non-inferiority trial vs. fluticasone 0.005% ointment. *Pediatr Allergy Immunol*. 2010;**21**(2 Pt 1):321-9.
7. Luger T, Van Leent E, Graeber M, Hedgecock S, Thurston M, Kandra A, et al. SDZ ASM 981: an emerging safe and effective treatment for atopic dermatitis. *Br J Dermatol*. 2001;**144**(4):788-94
8. Reitamo S, Rustin M, Ruzicka T, Cambazard F, Kalimo K, Friedmann P, et al. Efficacy and safety of tacrolimus ointment compared with that of hydrocortisone butyrate ointment in adult patients with atopic dermatitis. *J Allergy Clin Immunol*. 2002;**109(**3):547-55
9. Hofman T, Cranswick N, Kuna P, Boznanski A, Latos T, Gold M, et al. Tacrolimus ointment does not affect the immediate response to vaccination, the generation of immune memory, or humoral and cell-mediated immunity in children. *Arch Dis Child*. 2006;**91**(11):905-10
10. Sikder M, Al Mamun S, Khan R, Chowdhury A, Khan H, Hoque M. Topical 0.03% tacrolimus ointment, 0.05% clobetasone butyrate cream alone and their combination in older children with atopic dermatitis - An open randomized comparative study. *J Pak Assoc Dermatol*. 2005;**15**(4):304-12
11. Reitamo S, Van Leent E, Ho V, Harper J, Ruzicka T, Kalimo K, et al. Efficacy and safety of tacrolimus ointment compared with that of hydrocortisone acetate ointment in children with atopic dermatitis. *J Allergy Clin Immunol*. 2002;**109**(3):539-46
12. Reitamo S, Harper J, Bos J, Cambazard F, Bruijnzeel-Koomen C, Valk P, et al. 0.03% Tacrolimus ointment applied once or twice daily is more efficacious than 1% hydrocortisone acetate in children with moderate to severe atopic dermatitis: results of a randomized double-blind controlled trial. *Br J Dermatol*. 2004;**150**(3):554-62

Axon E, *et al*. *BMJ Open* 2021; 11:e046476. doi: 10.1136/bmjopen-2020-046476
