# Supplementary material for: Safety of topical corticosteroids in atopic eczema: an umbrella review
Source: BMJ Open. 2021 Jul 7;11(7):e046476. doi: 10.1136/bmjopen-2020-046476 (PMC8264889; doi:10.1136/bmjopen-2020-046476)
Supplement: Author's manuscript [file bmjopen-2020-046476.draft_revisions.pdf]

BMJ Open is committed to open peer review. As part of this commitment we make the peer review history of every article we publish publicly available.

When an article is published we post the peer reviewers' comments and the authors' responses online. We also post the versions of the paper that were used during peer review. These are the versions that the peer review comments apply to.

The versions of the paper that follow are the versions that were submitted during the peer review process. They are not the versions of record or the final published versions. They should not be cited or distributed as the published version of this manuscript.

BMJ Open is an open access journal and the full, final, typeset and author-corrected version of record of the manuscript is available on our site with no access controls, subscription charges or pay-per-view fees (http://bmjopen.bmj.com).

If you have any questions on BMJ Open's open peer review process please email info.bmjopen@bmj.com

Safety of topical corticosteroids in atopic eczema; an umbrella review

Journal:	*BMJ Open*
Manuscript ID	bmjopen-2020-046476
Article Type:	Original research
Date Submitted by the Author:	30-Oct-2020

Complete List of Authors:	Axon, Emma; University of Nottingham, Centre of Evidence Based Dermatology<br>Chalmers, Joanne; University of Nottingham, Centre of Evidence Based Dermatology<br>Santer, Miriam; University of Southampton, Primary Care & Population Sciences<br>Ridd, Matthew; University of Bristol Faculty of Health Sciences, Population Health Sciences;<br>Lawton, Sandra; Rotherham NHS Foundation Trust, Dermatology Department<br>Langan, Sinead; London School of Hygiene & Tropical Medicine,<br>Grindlay, Douglas; University of Nottingham School of Health Sciences<br>Muller, Ingrid; University of Southampton, School of Primary Care, Population Health and Medical Education<br>Roberts, Amanda; University of Nottingham, Centre of Evidence-Based Dermatology<br>Ahmed, Amina; University of Nottingham, Centre of Evidence Based Dermatology<br>Williams, HC; University of Nottingham,<br>Thomas, Kim; University of Nottingham, Dermatology

Keywords:	Eczema < DERMATOLOGY, Paediatric dermatology < PAEDIATRICS, Adult dermatology < DERMATOLOGY

SCHOLARONE™
Manuscripts

BMJ

I, the Submitting Author has the right to grant and does grant on behalf of all authors of the Work (as defined in the below author licence), an exclusive licence and/or a non-exclusive licence for contributions from authors who are: i) UK Crown employees; ii) where BMJ has agreed a CC-BY licence shall apply, and/or iii) in accordance with the terms applicable for US Federal Government officers or employees acting as part of their official duties; on a worldwide, perpetual, irrevocable, royalty-free basis to BMJ Publishing Group Ltd ("BMJ") its licensees and where the relevant Journal is co-owned by BMJ to the co-owners of the Journal, to publish the Work in this journal and any other BMJ products and to exploit all rights, as set out in our licence.

The Submitting Author accepts and understands that any supply made under these terms is made by BMJ to the Submitting Author unless you are acting as an employee on behalf of your employer or a postgraduate student of an affiliated institution which is paying any applicable article publishing charge ("APC") for Open Access articles. Where the Submitting Author wishes to make the Work available on an Open Access basis (and intends to pay the relevant APC), the terms of reuse of such Open Access shall be governed by a Creative Commons licence – details of these licences and which Creative Commons licence will apply to this Work are set out in our licence referred to above.

Other than as permitted in any relevant BMJ Author's Self Archiving Policies, I confirm this Work has not been accepted for publication elsewhere, is not being considered for publication elsewhere and does not duplicate material already published. I confirm all authors consent to publication of this Work and authorise the granting of this licence.

For peer review only

Safety of topical corticosteroids in atopic eczema; an umbrella review

Dr Emma Axon (ORCID ID: 0000-0002-3246-9968)
Systematic Review Methodologist and Research Associate
Centre of Evidence Based Dermatology, School of Medicine, University of Nottingham, Kings Meadow Campus, Nottingham, UK, NG7 2NR,
Dr Joanne R Chalmers (ORCID ID: 0000-0002-2281-7367)
Senior Research Fellow, Centre of Evidence Based Dermatology, School of Medicine, University of Nottingham, Kings Meadow Campus, Nottingham, UK, NG7 2NR.
Dr Miriam Santer (ORCID ID: 0000-0001-7264-5260)
Associate Professor in Primary Care Research, Primary Care, Population Sciences & Medical Education, University of Southampton, Southampton, SO16 5ST, UK,
Dr Matthew J Ridd (ORCID ID: 0000-0002-7954-8823)
GP and Reader in Primary Healthcare, Population Health Sciences, Bristol Medical School, University of Bristol, Canynge Hall, 39 Whatley Road, Bristol, BS8 2PS, UK.
Mrs Sandra Lawton (ORCID ID: 0000-0002-6163-5822)
Nurse Consultant and Clinical Lead Dermatology, Rotherham NHS Foundation Trust, Moorgate Road, Rotherham, S60 2UD, UK
Professor Sinéad M Langan (ORCID ID: 0000-0002-7022-7441)
Wellcome Senior Clinical Fellow and Professor**,** London School of Hygiene and Tropical Medicine, Faculty of Epidemiology and Population Health, Room 250, Keppel Street, London, UK WC1E 7HT.
Dr Douglas J Grindlay (ORCID ID: 0000-0002-0992-7182)
Information Specialist, Centre of Evidence Based Dermatology, School of Medicine, University of Nottingham, Kings Meadow Campus, Nottingham, UK, NG7 2NR.
Dr Ingrid Muller (ORCID ID: 0000-0001-9341-6133)
Health Psychologist, Primary Care, Population Sciences & Medical Education, University of Southampton, Southampton, SO16 5ST, UK.
Mrs Amanda Roberts (ORCID ID: 0000-0003-0370-3695)
Patient and public involvement representative, Centre of Evidence Based Dermatology, School of Medicine, University of Nottingham, Kings Meadow Campus, Nottingham, UK, NG7 2NR.
Mrs Amina Ahmed (ORCID ID: 0000-0001-9494-742X)
Patient and public involvement representative, Centre of Evidence Based Dermatology, School of Medicine, University of Nottingham, Kings Meadow Campus, Nottingham, UK, NG7 2NR.
Professor Hywel C Williams (ORCID ID: 0000-0002-5646-3093)
Professor of Dermato-Epidemiology, Centre of Evidence Based Dermatology, School of Medicine, University of Nottingham, Kings Meadow Campus, Nottingham, UK, NG7 2NR.
Professor Kim S Thomas (ORCID ID: 0000-0001-7785-7465)
Professor of Applied Dermatology Research, Centre of Evidence Based Dermatology, School of Medicine, University of Nottingham, Kings Meadow Campus, Nottingham, UK, NG7 2NR.

Corresponding author: Dr Joanne R Chalmers. Centre of Evidence Based Dermatology, University of Nottingham, King's Meadow Campus, Nottingham, NG7 2NR. Joanne.chalmers@nottingham.ac.uk. +44 (0) 115 82 32435

Funding: This report presents independent research funded by the National Institute for Health Research (NIHR) under its Programme Grants for Applied Research programme (grant ref No. RP-PG-0216-20007). The views expressed are those of the author(s) and not necessarily those of the NHS, the NIHR or the department of Health and Social Care.

Competing interests: Authors are co-applicants on a NIHR Programme Grants for Applied Research (P-PG-0216-20007) which funded this overview. The aim of the Programme Grant is to develop an

For peer review only - http://bmjopen.bmj.com/site/about/guidelines.xhtml

intervention to support eczema self-care and the results of this overview will contribute to this intervention. MJR is funded by an NIHR Post-Doctoral Research Fellowship (PDF-2014-07-013). SML is supported by a Wellcome Senior Clinical fellowship in Science (205039/Z/16/Z).

Word count: abstract 300 words, main text 3566 words

For peer review only

Abstract

Objective: To summarise all safety data from systematic reviews of TCS in adults and children with atopic eczema in an umbrella review.

Methods: Embase, MEDLINE, PubMed, Cochrane Database of Systematic Reviews, Epistemonikos and the Centre of Evidence Based Dermatology map of eczema systematic reviews were searched until 7th November 2018. Reviews were included if they assessed safety of TCS in atopic eczema and searched ≥1 database using a reproducible search strategy. Review quality assessed using AMSTAR-2. PROSPERO registration: CRD42018079409.

Results: 36 systematic reviews included, 32 low/critically low quality. Treatment and follow-up usually short (2-4 weeks). Key findings:

TCS versus emollient/vehicle: No meta-analyses identified for skin-thinning. Two 2-week randomised controlled trials (RCTs) found no significant increased risk with very potent TCS (0/196 TCS vs 0/33 vehicle in children and 6/109 TCS vs 2/50 vehicle, age unknown). Biochemical adrenal suppression (cortisol) was 3.8% (95% CI 2.4%-5.8%) in a meta-analysis of 11 uncontrolled observational studies (any potency TCS, 522 children). Effects were reversed when treatment ceased.

TCS versus topical calcineurin inhibitors (TCIs): Meta-analysis showed higher relative risk of skin-thinning with TCS (RR 4.86, 95% CI 1.06-22.28, n=4128, four RCTs, including one 5-year RCT). Eight cases in 2068 participants, 7 using potent TCS. No evidence of growth suppression.

Once daily versus more frequent TCS: No meta-analyses identified. No skin-thinning in one RCT (3 weeks potent TCS, n=94) or biochemical adrenal suppression in two RCTs (up to 2 weeks very potent/moderate TCS, n=129).

For peer review only - http://bmjopen.bmj.com/site/about/guidelines.xhtml

TCS twice/week to prevent flares ('weekend therapy') versus vehicle: No meta-analyses identified. No evidence of skin-thinning in five RCTs. One RCT found biochemical adrenal suppression (2/44 children, potent TCS).

Conclusions: We found no evidence of harm when TCS are used intermittently "as required" to treat flares or "weekend therapy" to prevent flares. However on long-term safety evidence was limited.

Strengths and limitations of this overview

- This is a comprehensive overview of systematic reviews which, for the first time, brings together all safety data from systematic reviews on topical corticosteroids (TCS) used in eczema, a topic that was identified as a priority in a James Lind Alliance priority setting partnership on eczema.
- Robust Cochrane methodology was followed and included a thorough and inclusive literature search.
- People with eczema and parents of children with eczema have inputted throughout this overview to ensure it meets the needs of patients as well as healthcare professionals.
- By extracting data from existing reviews, results are limited to data reported in those reviews possibly missing other data included in the original trials and are limited to topics for which there is an eligible systematic review.

For peer review only

Keywords:

Atopic eczema, topical corticosteroids, adverse events

Abbreviations:

AMSTAR 2 = A MeaSurement Tool to Assess systematic Reviews 2

CI = Confidence interval

GRADE = Grading of Recommendations, Assessment, Development and Evaluation

NHS = National Health Service

For peer review only - http://bmjopen.bmj.com/site/about/guidelines.xhtml

RCT = Randomised controlled trial

RR = Relative risk

TCI = Topical calcineurin inhibitors

TCS = Topical corticosteroids

UK = United Kingdom

USA = United States of America

Introduction

Atopic eczema (also known as atopic dermatitis, or eczema) is an itchy inflammatory skin condition. It is most common in children with 1 in 5 affected worldwide [1, 2], but often persists into adulthood. [3]

Topical corticosteroids (TCS) are first line therapy for treating inflammatory eczema flares but widespread concerns regarding their safety amongst patients and healthcare professionals contribute to poor adherence, and subsequent worsening of disease control and quality of life. [4, 5] Safety concerns include skin thinning and retardation of growth and development. These concerns are thought to mainly originate from what is now considered to be inappropriate use, such as using potent TCS on the face or continual long-term use. [6] Strategies recommended to minimise exposure to TCS, and hence the risk of adverse events, include reducing frequency of application to once daily during treatment of an inflammatory episode, or TCS used for two consecutive days a week (sometimes referred to as “weekend therapy”) as a strategy to prevent flares. [7-9] This umbrella review aims to assess TCS safety in atopic eczema, and addressed two areas of research prioritised in the James Lind Alliance priority setting partnership for atopic eczema. [10]

Methods

Protocol, registration, and study design

This umbrella review includes published systematic reviews of randomised controlled trials (RCTs) and/or observational studies reporting adverse event data in people with eczema using TCS. The aim of this overview was to summarise data from existing reviews, therefore, meta-analyses and data from individual studies were extracted and presented in this overview in the format and

For peer review only - http://bmjopen.bmj.com/site/about/guidelines.xhtml

completeness that they were presented in the original systematic reviews. The only exception was for missing p values which were calculated where appropriate. The checklist 'Preferred Reporting Items for overview of systematic reviews' was followed [11], and the protocol was registered on PROSPERO (CRD42018079409). [12]

Search strategy

Embase, MEDLINE, PubMed, Cochrane Database of Systematic Reviews and Epistemonikos were searched from inception to 7th November 2018 by DG (Information Specialist), with no restrictions on language or publication date. The search strategy is in Appendix 1. We also checked the Centre of Evidence Based Dermatology eczema map of systematic reviews, [13] and searched PROSPERO on 13th July 2018 for any relevant ongoing systematic reviews using the terms "eczema" and "dermatitis".

Eligibility criteria

We included systematic reviews that presented data on the safety of TCS used to treat participants of any age and gender with atopic eczema, searched at least one database and provided a reproducible search strategy. Multiple reviews on the same topic were included, except for "abridged" versions of the same review where no additional data were reported. For each comparison, the review that included the highest number of studies on that comparison and therefore appeared the most comprehensive was taken as the primary review and other included reviews were checked for additional studies and data. Conference abstracts were excluded. Reviews that covered multiple skin conditions were only included if they reported data on atopic eczema patients separately. Studies comparing TCS with non-topical treatments were excluded.

Outcomes

Safety outcomes reported during treatment and follow-up were extracted where reported in the reviews on cutaneous adverse events (e.g. skin thinning, telangiectasia, skin infections, folliculitis), systemic adverse events (e.g. effects on endocrine system, impact on growth) and rebound symptoms/steroid withdrawal.

For peer review only - http://bmjopen.bmj.com/site/about/guidelines.xhtml

Selection of studies and data extraction

Records identified from the database searches were uploaded into Covidence© (Veritas Health Innovation, Australia). [14] Two authors (EA and JRC) independently assessed the eligibility of each record, and where unclear the full text was obtained. The number of included and excluded records along with reasons for exclusion were reported in a Preferred Reporting Items for Systematic Reviews and Meta-Analyses (PRISMA) flow diagram.

Two authors (EA and JRC) independently extracted all safety data presented in the included reviews along with other information such as review/participant characteristics, and funding sources). Any disagreements regarding eligibility or data extraction were resolved via discussion or input from a third reviewer (HCW or KST).

Assessment of quality of included systematic reviews

As this was an overview of reviews, the methodological quality of the evidence was assessed at the systematic review level using the AMSTAR 2 tool. [15] Reviews were considered critically low quality if there was more than one critical flaw. Data on the quality of individual studies (e.g. risk of bias) and the quality of evidence (e.g. GRADE[16]) were also extracted where presented in the review, but undertaking these quality assessments for individual studies was not within the remit of this overview.

Measures of treatment effect and data synthesis

Where relevant meta-analyses were presented in the systematic review, *the forest plots*, relative risk (RR) and 95% confidence intervals (CI) were extracted. *In the absence of any meta-analysis, adverse event data* from individual studies *were included in this overview based on the data presented in the published systematic review*. P values were calculated using Review Manager software, [17] with <0.05 indicating statistically significant results. Where meta-analyses were presented, we assessed the following subgroups where possible: age, TCS potency, anatomical site, treatment duration, and genetic predisposition to a disrupted skin barrier (filaggrin status). TCS potency was determined

For peer review only - http://bmjopen.bmj.com/site/about/guidelines.xhtml

using a hierarchy of sources: UK 'British National Formulary', World Health Organization, and USA classifications. [18-20] An NHS classification ranging from very common (>1 in 10 people affected) to very rare (< 1 in 10,000) was used to narratively describe the absolute risk of each adverse event. [21]

Patient and public involvement

People with eczema and parents of children with eczema have been involved the decision to conduct this overview and in the design. The James Lind Alliance priority setting partnership for atopic eczema involved people with eczema and parents of children with eczema in which two of the identified priority areas were around research into the safety of TCS. [10] Two of the overview authors are patient representatives (AR, AA) and both have been involved in the design of this overview and interpretation of the findings.

Wider patient and parent involvement has been particularly important in identifying important safety outcomes for this overview. We held a workshop involving five patient representatives in which the proposed overview was discussed which highlighted the need to seek out data on long-term TCS use, reversibility of any side effects and TCS withdrawal symptoms. We supplemented this with a survey about safety concerns with TCS at a National Eczema Society meeting of thirty-one people with eczema or parents of children with eczema and a published qualitative study of patient concerns relating to TCS safety. [6]

Dissemination of the results are underway as part of the wider programme of research of which this overview is a part and our patient representatives are a key stakeholder in this activity.

Results

Search results

After de-duplication, 598 records were screened; 122 records underwent full text screening and 36 systematic reviews met the inclusion criteria (**Error! Reference source not found.**). [7, 8, 22-55] The list of

For peer review only - http://bmjopen.bmj.com/site/about/guidelines.xhtml

excluded reviews is in Appendix 2. The search of PROSPERO identified four ongoing systematic reviews (Appendix 3). [56-59]

Characteristics and quality of the included systematic reviews

All but three reviews were published in English. Two Chinese reviews and one German review were translated into English. [32, 36, 45] Twenty-eight of the included reviews were rated critically low quality according to AMSTAR-2; with four low, two moderate, and two high quality (Table 1). The most common reasons for downgrading were no protocol, no list of full text exclusions or a literature search restricted to the English language.

The included reviews identified 73 RCTs and 30 observational studies that included relevant safety data. Risk of bias assessments were available from the reviews for 63 RCTs, most reporting at least one domain rated as high or unclear risk. The trials included in the reviews usually evaluated use of short bursts of TCS (2-4 weeks) to treat the flare but varied greatly in length of follow-up. Around two thirds of trials included no post-treatment follow-up, whilst the remainder included several weeks/months of follow-up generally using TCS intermittently "as required". One notable trial (the 'PETITE' study) had 5 years follow-up with TCS used "as required". [60]

Characteristics and quality assessments of each systematic review are in **Error! Reference source not found.**, with further detail in Appendix 4 and 5. Individual study data and quality assessments are in Appendix 6.

Safety of TCS compared to other topical treatments or corticosteroids

How safe are TCS compared to emollient or vehicle, or no comparison?

Twelve reviews provided data on this comparison: one high [53], two low, [41, 46] and nine critically low quality. [24, 27, 30, 34-36, 49, 50, 54] Key results can be found in Table 2 and additional data in Appendix 6.

For peer review only - http://bmjopen.bmj.com/site/about/guidelines.xhtml

Reported rates of skin thinning in RCTs were generally very low, with no significant increases seen with TCS compared to emollient/vehicle. No skin thinning or telangiectasia was reported in an RCT, 196 participants aged ≥12 years old using very potent TCS twice a day for 2 weeks compared to 50 using vehicle. [61] Another RCT reported skin thinning in 6/109 participants using very potent TCS for 2 weeks compared to 2/50 using vehicle, p=0.69. [62]

No significant differences in other cutaneous adverse events, such as hypopigmentation, were observed between treatments in five RCTs, and event rates were low. [63-67]

A meta-analysis[54] of 11 uncontrolled observational studies (up to 4 weeks of treatment) reported biochemical adrenal suppression (cortisol levels) in 20/522 children (3.8%, 95% CI 2.4% to 5.8%) with any potency TCS. [68-78] This was 2% (3/148 children) when only mild potency TCS were analysed. [69, 71, 74, 76] No clinical symptoms or signs of adrenal suppression were observed[68-78], and the biochemical effects were transient, with cortisol levels returning to normal after TCS were discontinued. [68, 72, 74, 75, 78]

Two included reviews assessed TCS withdrawal symptoms, mostly from case reports, but no incidence data were reported. [40, 43]

How safe are TCS compared to topical calcineurin inhibitors (TCI)?

Seven systematic reviews were identified: one moderate [22], one low [47], and five critically low quality. [26, 29, 42, 49, 51] Most RCTs used twice daily TCS to treat the current flare (up to 3 weeks), and where longer-term follow-up was included, TCS were used “as required” to treat flares. Key results can be found in Table 2 and additional data in Appendix 6.

Meta-analyses of cutaneous adverse events were presented in one review [26] which was slightly modified for this overview**Error! Reference source not found.** (modifications shown in Appendix 7). A meta-analysis of four RCTs (26 weeks to 5 years duration, twice a day or “as directed”) showed a significant increase in the relative risk of skin thinning with TCS compared with TCIs (0.1% tacrolimus

For peer review only - http://bmjopen.bmj.com/site/about/guidelines.xhtml

or 1% pimecrolimus) (RR 4.86, 95% CI 1.06 to 22.28, p=0.04, n=4128). However, skin thinning was uncommon: 8/2068 participants (0.4%) with TCS versus 0/2060 (0%) with TCIs. Of the eight cases of skin thinning, seven were reported when using potent TCS and one using mild/moderate TCS. [60, 79-81]

The relative risk of skin burning and pruritus (itching) was significantly lower with TCS compared to TCIs (1% pimecrolimus or 0.1% / 0.03% tacrolimus) in meta-analyses of 10 RCTs in 4211 participants (skin burning: RR 0.31, 95% CI 0.23 to 0.40, p<0.00001; pruritus: RR 0.68, 95% CI 0.56 to 0.82, p <0.0001*)***.* [79, 81-89] There was no significant difference in skin infections with potent, moderate or mild potency TCS compared to TCIs (1% pimecrolimus or 0.1% / 0.03% tacrolimus) [60, 79-82, 84, 86, 88] or erythema compared with 0.1% tacrolimus (Appendix 8). [87, 88]

Subgroup analyses of age, TCS potency and specific TCI showed no significant differences for any comparison (Appendix 9). We were unable to undertake any further subgroup analyses.

No differences in growth were observed in one 5-year RCT (‘PETITE’ study) in 2418 young children using moderate/mild potency TCS compared to those using TCI (1% pimecrolimus) (rates not given) and no cases of lymphoma were reported. [60] A large cohort study (n=1,438,333) showed a small non-significant increased risk of lymphoma with TCI and TCS compared to the general population, with a similar risk between treatments. [90] In addition, one case-control study (294 cases/293,000 controls) found no increased risk of lymphoma with TCS or TCI compared to controls. [91]

Is there any difference in safety of TCS of different potencies?

Six reviews compared the safety of different potency TCS: two low,[41, 46] and four critically low quality. [27, 33, 49, 52] RCTs were mainly short-term use of TCS (2-3 weeks), used once or twice daily. Results can be found in Appendix 6.

One RCT reported mild skin thinning in 4/13 children using potent TCS for up to 6 weeks compared with 2/12 using mild TCS (p=0.42), [92] whilst another RCT in 37 children found no evidence of skin thinning with mild or moderate potency TCS for 3 weeks. [93] One study compared 3 weeks of potent

For peer review only - http://bmjopen.bmj.com/site/about/guidelines.xhtml

and moderate TCS in 40 children and reported “some” biochemical adrenal suppression (cortisol levels) but no numerical data were provided. [94]

How safe are TCS compared to topically applied Chinese herbal medicine?

Two systematic reviews provided data on TCS compared with topical Chinese herbal medicine: one high quality [39] and one critically low. [38] Results can be found in Appendix 6.

A meta-analysis of two RCTs [95, 96] was presented in two systematic reviews. [38, 39] More cutaneous adverse events, including application site burning, were observed with 2 weeks of very potent/potent TCS compared to topical Chinese herbal medicine (RR 12.03, 95% CI 1.59 to 91.26, p=0.02; 11/147 versus 0/148 participants). One additional RCT, including 95 young children, reported minor adverse events such as burning with 2 weeks of potent TCS but no numerical data were presented. [97]

Safety of different strategies for using TCS

How safe are once daily TCS compared with more frequent application?

Two low quality reviews provided safety data relating to different frequency of application. [7, 46] Key results can be found in Table 2 and additional data in Appendix 6.

No skin thinning was reported with once or twice daily application of potent TCS for 3 weeks in one RCT (94 adults). [98] Four RCTs in 740 older children/adults showed no significant difference between once and twice daily application of moderate/potent TCS in other cutaneous adverse events including telangiectasia, [99, 100], folliculitis, [101] and burning, itching or stinging. [101, 102] Two RCTs showed no significant differences in biochemical adrenal suppression (cortisol levels) between once and twice daily very potent/moderate TCS used for up to 2 weeks in 129 children. [78, 103]

For peer review only - http://bmjopen.bmj.com/site/about/guidelines.xhtml

How safe are TCS when used proactively to prevent flares ("weekend therapy")?

Two reviews included data on the safety of TCS used proactively 2 days a week ("weekend therapy") to prevent flares, both critically low quality. [8, 52] Key results can be found in Table 2 and additional data in Appendix 6.

There was no evidence of skin thinning in five RCTs comparing 16-20 weeks of weekend therapy with potent TCS versus vehicle in 993 participants. [99, 104-107] Furthermore, two RCTs (n=423) reported no significant differences in other cutaneous adverse events, including folliculitis and transient telangiectasia, with potent TCS compared to vehicle. [104, 105] Events were uncommon in both groups.

There was no evidence of biochemical adrenal suppression (cortisol levels) in two RCTs (n=129) between potent TCS and vehicle used for 16 weeks. [104, 107] In a 20-week RCT, 2/44 children had possible adrenal suppression with potent TCS compared to zero with vehicle. [105]

How safe are TCS used under occlusion?

Four reviews included data on the safety of TCS used under occlusion: one high [53], and three critically low quality. [25, 31, 37] Results can be found in Appendix 6.

There were no cases of skin thinning and one case of striae in two uncontrolled observational studies of a diluted potent TCS used under wet-wrap for 1-2 weeks in 44 young children. [108, 109] A significant difference in the rate of folliculitis (mostly mild) was observed in one RCT of TCS under wet-wrap for 4 weeks, with more folliculitis in the diluted potent TCS group (10/19 children) compared to emollient (2/20 children) (p=0.02). [110] A meta-analysis from one review [37] of two RCTs in young children showed no significant difference in the number of participants with cutaneous adverse events between mild potency TCS under wet wrap (7/38 participants) versus not under wet-wrap (0/31 participants) (p=0.08)[111, 112]; this evidence was rated low quality by the systematic review authors using GRADE. [16]

For peer review only - http://bmjopen.bmj.com/site/about/guidelines.xhtml

Biochemical adrenal suppression (cortisol levels) was reported in three uncontrolled observational studies of 2-14 days of diluted potent TCS under wet-wraps in 74 children. [108, 109, 113] Actual rates were not specified in the review, but increases were described as transient in two studies. [108, 113] One short-term uncontrolled observational study of diluted potent TCS under wet-wrap in eight children showed no effect on growth or bone turnover. [114]

Discussion

This umbrella review brings together all safety data for TCS used in treating eczema included from 36 systematic reviews. Although skin thinning and other side effects of TCS concern many people with eczema and parents of children with eczema, we found no evidence of harm when TCS were used intermittently "as required" to treat flares or as "weekend therapy" to prevent flares. [5] Similarly, no evidence of growth retardation or clinically significant adrenal suppression was found. Adherence to TCS treatment is known to be poor and these findings may encourage appropriate use of TCS and therefore improve treatment effectiveness and patient benefit. [115]

A thorough literature search was conducted and Cochrane methodology was used. Conclusions were limited by the content of the included reviews because safety was frequently reported in less detail than effectiveness, reviews reported on different adverse events and some adverse events were not described in the reviews. It is not clear whether this is because the trials did not report adverse events in sufficient detail or whether the review authors didn't include all the available safety data, perhaps only focussing on a restricted group of adverse events. Furthermore, most of the included reviews were rated low or critically low quality using AMSTAR 2. The lack of comprehensive search strategies and duplicate screening/data extraction in the included reviews may have resulted in missing studies and safety data, which could have impacted on this overview particularly where there was limited data. In addition, where the quality of evidence assessments (e.g. GRADE) was reported in the reviews, most individual studies included in the reviews indicated a high or unclear risk in at least one domain.

For peer review only

Many RCTs did not include follow-up beyond 2-4 weeks of treatment and therefore data on long-term safety are limited. Although short-term TCS use reflects appropriate treatment duration for treating an individual flare, it does not reflect the chronic nature of eczema and the need for TCS use over the long-term. The 'PETITE study' was the notable exception and data published in the correspondence showed there was only one episode of skin thinning in 1213 children using mild/moderate TCS "as required" with 5-year follow up. [60] Trials using intermittent TCS as "weekend therapy" to prevent flares also provide reassurance for the safety of longer-term use of TCS, as these trials generally included 16-20 weeks of follow-up to assess the prevention of flares. The inclusion of systematic reviews that included observational studies as well as reviews of RCTs also increased the amount of safety data available to report in this overview.

Although this review focussed on the safety of TCS as the key issue for patients, treatment decisions are a balance of benefits and harms. For example, although the safety profile of Chinese herbal medicine was better than TCS, in practice this would be considered alongside the relative effectiveness of these treatments. Likewise, although there was no difference in the safety of once versus twice daily TCS, effectiveness of these regimens is also important to consider. A Cochrane review is underway comparing the effectiveness and safety of different ways of using TCS. [116]

In summary, we found no evidence that TCS cause harm when used intermittently "as required" to treatment eczema flares or as "weekend therapy" to prevent flares and this should support the use of TCS in the management of eczema. However, high-quality evidence was limited, particularly for long-term use. Future RCTs should include lengthier follow-up to enable assessment of longer-term effects of treatments, and more data on whether adverse events resolved when treatment stopped. The adverse events of greatest concern to patients and clinicians, such as skin thinning, are uncommon.

For peer review only - http://bmjopen.bmj.com/site/about/guidelines.xhtml

Authors contributions and acknowledgements

All authors helped conceive of and design this overview, draft or critique the output for important intellectual content and interpret the results. EA and JRC screened the titles, abstracts and full texts, and extracted and collated the data. HCW and KST acted as 3rd reviewers to resolve disagreements. EA performed the statistical analysis and JRC is the study guarantor. All authors commented on and approved the final manuscript. The corresponding author attests that all listed authors meet authorship criteria and that no others meeting the criteria have been omitted.

We would like to thank Faye Shelton for her assistance with the screening of the search results and to Jane Harvey for her input into classifying the TCS potencies. We would also like to thank Chiau Ming Long for translating two of the included reviews published in Chinese, and to Jonathan Batchelor for confirming exclusion of two reviews published in Japanese.

References

1. Odhiambo JA, Williams HC, Clayton TO, Robertson CF, Asher MI, ISAAC Phase Three Study Group. Global variations in prevalence of eczema symptoms in children from ISAAC Phase Three. *J Allergy Clin Immunol*. 2009;**124**(6):1251-8. e23.
2. Weidinger S, Novak N. Atopic dermatitis. *Lancet*. 2016;**387**(10023):1109-22.
3. Abuabara K, Yu A, Okhovat J, Allen I, Langan S. The prevalence of atopic dermatitis beyond childhood: A systematic review and meta-analysis of longitudinal studies. *Allergy*. 2018;**73**(3):696-704.
4. Aubert-Wastiaux H, Moret L, Le Rhun A, Fontenoy A, Nguyen J, Leux C, et al. Topical corticosteroid phobia in atopic dermatitis: a study of its nature, origins and frequency. *Br J Dermatol*. 2011;**165**(4):808-14.
5. Charman C, Morris A, Williams H. Topical corticosteroid phobia in patients with atopic eczema. *Br J Dermatol*. 2000;**142**(5):931-6.
6. Li AW, Yin ES, Antaya RJ. Topical Corticosteroid Phobia in Atopic Dermatitis: A Systematic Review. *JAMA Dermatol*. 2017;**153**(10):1036-42.
7. Green C, Colquitt J, Kirby J, Davidson P, Payne E. Clinical and cost-effectiveness of once-daily versus more frequent use of same potency topical corticosteroids for atopic eczema: a systematic review and economic evaluation. *Health Technol Assess (Winchester, England)*. 2004;**8**(47):iii,iv, 1-120.
8. Schmitt J, Von Kobyletzki L, Svensson A, Apfelbacher C. Efficacy and tolerability of proactive treatment with topical corticosteroids and calcineurin inhibitors for atopic eczema: Systematic review and meta-analysis of randomized controlled trials. *Br J Dermatol*. 2011;**164**(2):415-28.

For peer review only - http://bmjopen.bmj.com/site/about/guidelines.xhtml

9. Williams H. Established corticosteroid creams should be applied only once daily in patients with atopic eczema. *BMJ* 2007;**334**(7606):1272.
10. Batchelor J, Ridd M, Clarke T, Ahmed A, Cox M, Crowe S, et al. The Eczema Priority Setting Partnership: a collaboration between patients, carers, clinicians and researchers to identify and prioritize important research questions for the treatment of eczema. *Br J Dermatol*. 2013;**168**(3):577-82.
11. Bougioukas KI LA, Tsapas A, Ntzani E, Haidich AB. Preferred Reporting Items for Overviews of systematic reviews including harms checklist: A pilot tool to be used for balanced reporting of benefits and harms. *J Clin Epidemiol*. 2018;**93**:9-24.
12. Mead E, Chalmers J, Thomas K, Santer M, Williams H, Grindlay D, et al. The safety of topical corticosteroids in atopic eczema: an overview of systematic reviews [CRD42018079409]2017 (accessed: 13/07/18). Available from: https://www.crd.york.ac.uk/prospero/display_record.php?RecordID=79409.
13. Centre of Evidence Based Dermatology. Systematic reviews on eczema: University of Nottingham; 2018 [07/11/2018]. Available from: https://www.nottingham.ac.uk/research/groups/cebd/resources/eczema-systematic-reviews.aspx.
14. Covidence systematic review software. Melbourne, Australia: Veritas Health Innovation. Available at www.covidence.org; 2017.
15. Shea BJ, Reeves BC, Wells G, Thuku M, Hamel C, Moran J, et al. AMSTAR 2: a critical appraisal tool for systematic reviews that include randomised or non-randomised studies of healthcare interventions, or both. *BMJ* 2017;**358**:j4008.
16. Schünemann H BJ, Oxman A, editor(s). Handbook for grading the quality of evidence and the strength of recommendations using the GRADE approach (updated October 2013). GRADE Working Group, 2013: The GRADE Working Group.; 2013. Available from: www.guidelinedevelopment.org/handbook.
17. Review Manager 5 (RevMan 5). Copenhagen: Nordic Cochrane Centre, The Cochrane Collaboration; 2014.
18. Resource clinical. Potency of Topical Corticosteroids (UK Classification). 2015 [Available from: http://www.resourceclinical.com/clinical-tables/topical-corticosteroids-potency-UK-classification.html.
19. Resource clinical. Potency of Topical Corticosteroids (USA Classification) 2015 [Available from: http://www.resourceclinical.com/clinical-tables/topical-corticosteroids-potency-USA-classification.pdf.
20. World Health Organization (WHO). WHO Model Prescribing Information: Drugs Used in Skin Diseases. Classification of topical corticosteroids. 2017 [Available from: http://apps.who.int/medicinedocs/en/d/Jh2918e/32.html.
21. NHS. What are side effects? 2018 [Available from: https://www.nhs.uk/common-health-questions/medicines/what-are-side-effects/.
22. Ashcroft DM, Chen L-C, Garside R, Stein K, Williams HC. Topical pimecrolimus for eczema Cochrane Database Syst Rev [Internet]. 2007; (4). Available from: http://onlinelibrary.wiley.com/doi/10.1002/14651858.CD005500.pub2/abstract.
23. Ashcroft D, Dimmock P, Garside R, Stein K, Williams H. Efficacy and tolerability of topical pimecrolimus and tacrolimus in the treatment of atopic dermatitis: Meta-analysis of randomised controlled trials. *BMJ* 2005;**330**(7490):516-22.
24. Barnes L, Kaya G, Rollason V. Topical Corticosteroid-Induced Skin Atrophy: A Comprehensive Review. *Drug Saf*. 2015;**38**(5):493-509.
25. Braham S, Pugashetti R, Koo J, Maibach H. Occlusive therapy in atopic dermatitis: overview. *J Dermatolog Treat*. 2010;**21**(2):62-72.
26. Broeders J, Ahmed Ali U, Fischer G. Systematic review and meta-analysis of randomized clinical trials (RCTs) comparing topical calcineurin inhibitors with topical corticosteroids for atopic dermatitis: A 15-year experience. *J Am Acad Dermatol*. 2016;**75**(2):410-9.e3.

For peer review only - http://bmjopen.bmj.com/site/about/guidelines.xhtml

27. Callen J, Chamlin S, Eichenfield L, Ellis C, Girardi M, Goldfarb M, et al. A systematic review of the safety of topical therapies for atopic dermatitis. *Br J Dermatol*. 2007;**156**(2):203-21.
28. Chen S, Yan J, Wang F. Two topical calcineurin inhibitors for the treatment of atopic dermatitis in pediatric patients: A meta-analysis of randomized clinical trials. *J Dermatolog Treat*. 2010;**21**(3):144-56.
29. Cury Martins J, Martins C, Aoki V, Gois AF, Ishii HA, da SEM. Topical tacrolimus for atopic dermatitis. Cochrane Database Syst Rev [Internet]. 2015; (7). Available from: http://onlinelibrary.wiley.com/doi/10.1002/14651858.CD009864.pub2/abstract.
30. de Tiedra A, Mercadal J, Lozano R. Prednicarbate versus fluocortin for inflammatory dermatoses: A cost-effectiveness study. *PharmacoEconomics*. 1997;**12**(2 Pt 1):193-208.
31. Devillers A, Oranje A. Efficacy and safety of 'wet-wrap' dressings as an intervention treatment in children with severe and/or refractory atopic dermatitis: a critical review of the literature. *Br J Dermatol*. 2006;**154**(4):579-85.
32. Dong Y, Zeng W, Li W, Ma H, Zheng W. Efficacy and safety of topical tacrolimus for childhood atopic dermatitis; a meta-analysis. [Chinese]. *J Clin Dermatol*. 2017;**46**(4):239-42.
33. Eichenfield L, Tom W, Berger T, Krol A, Paller A, Schwarzenberger K, et al. Guidelines of care for the management of atopic dermatitis: Section 2. Management and treatment of atopic dermatitis with topical therapies. *J Am Acad Dermatol*. 2014;**71**(1):116-32.
34. Feldman S. Relative efficacy and interchangeability ofvarious clobetasol propionate vehicles in the management of steroid-responsive dermatoses. *Curr Ther Res Clin Exp*. 2005;**66**(3):154-71.
35. Frangos J, Kimball A. Clobetasol propionate emollient formulation foam in the treatment of corticosteroid-responsive dermatoses. *Expert Opin Pharmacother*. 2008;**9**(11):2001-7.
36. Froeschl B, Arts D, Leopold C. Corticosteroid therapy in the treatment of pediatric patients with atopic dermatitis (Structured abstract). Health Technol Assess [Internet]. 2007; (4). Available from: http://onlinelibrary.wiley.com/o/cochrane/clhta/articles/HTA-32008100208/frame.html.
37. Gonzalez-Lopez G, Ceballos-Rodriguez R, Gonzalez-Lopez J, Feito Rodriguez M, Herranz-Pinto P. Efficacy and safety of wet wrap therapy for patients with atopic dermatitis: a systematic review and meta-analysis. *Br J Dermatol*. 2017;**177**(3):688-95.
38. Gu S, Yang A, Li C, Lu C, Xue C. Topical application of Chinese herbal medicine for atopic eczema: A systematic review with a meta-analysis. *Dermatology*. 2014;**228**(4):294-302.
39. Gu S, Yang AW, Xue CC, Li CG, Pang C, Zhang W, et al. Chinese herbal medicine for atopic eczema. Cochrane Database Syst Rev [Internet]. 2013; (9). Available from: http://onlinelibrary.wiley.com/doi/10.1002/14651858.CD008642.pub2/abstract.
40. Hajar T, Leshem Y, Hanifin J, Nedorost S, Lio P, Paller A, et al. A systematic review of topical corticosteroid withdrawal ("steroid addiction") in patients with atopic dermatitis and other dermatoses. *J Am Acad Dermatol*. 2015;**72**(3):541-9.e2.
41. Hoare C, Li Wan Po A, Williams H. Systematic review of treatments for atopic eczema. *Health Technol Assess*. 2000;**4**(37):1-191.
42. Iskedjian M, Piwko C, Shear N, Langley R, Einarson T. Topical calcineurin inhibitors in the treatment of atopic dermatitis: A meta-analysis of current evidence. *Am J Clin Dermatol*. 2004;**5**(4):267-79.
43. Juhász ML, Curley RA, Rasmussen A, Malakouti M, Silverberg N, Jacob SE. Systematic Review of the Topical Steroid Addiction and Topical Steroid Withdrawal Phenomenon in Children Diagnosed With Atopic Dermatitis and Treated With Topical Corticosteroids. *J Dermatol Nurses Assoc*. 2017;**9**(5):233-40.
44. Legendre L, Barnetche T, Mazereeuw-Hautier J, Meyer N, Murrell D, Paul C. Risk of lymphoma in patients with atopic dermatitis and the role of topical treatment: A systematic review and meta-analysis. *J Am Acad Dermatol*. 2015;**72**(6):992-1002.
45. Dong R, Zhu H, Fan L, Ni S, Feng C, Wu Z. Efficacy and tolerability of topical tacrolimus in the treatment of atopic dermatitis: A systematic review of randomized controlled trials. [Chinese]. *J Clin Dermatol*. 2007;**36**(12):757-60.

For peer review only - http://bmjopen.bmj.com/site/about/guidelines.xhtml

46. Nankervis H, Thomas K, Delamere F, Barbarot S, Rogers N, Williams H. Scoping systematic review of treatments for eczema2016 2016/05/None.
47. Penaloza Hidalgo B, Knight T, Burls A. A systematic review of effectiveness and cost effectiveness of tacrolimus ointment for topical treatment of atopic dermatitis in adults and children Health Technol Assess [Internet]. 2004; (4):[81 p.].
48. Sidbury R, Tom W, Bergman J, Cooper K, Silverman R, Berger T, et al. Guidelines of care for the management of atopic dermatitis: Section 4. Prevention of disease flares and use of adjunctive therapies and approaches. *J Am Acad Dermatol*. 2014;**71**(6):1218-33.
49. Siegfried E, Jaworski J, Kaiser J, Hebert A. Systematic review of published trials: Long-term safety of topical corticosteroids and topical calcineurin inhibitors in pediatric patients with atopic dermatitis. *BMC Pediatr*. 2016;**16** (75).
50. Singh S, Mann B. Clinical utility of clocortolone pivalate for the treatment of corticosteroid-responsive skin disorders: A systematic review. *Clin Cosmet Investig Dermatol*. 2012;**5**:61-8.
51. Svensson A, Chambers C, Gånemo A, Mitchell S. A systematic review of tacrolimus ointment compared with corticosteroids in the treatment of atopic dermatitis. *Curr Med Res Opin*. 2011;**27**(7):1395-406.
52. Tang T, Bieber T, Williams H. Are the concepts of induction of remission and treatment of subclinical inflammation in atopic dermatitis clinically useful? *J Allergy Clin Immunol*. 2014;**133**(6):1615-25.e1.
53. van Zuuren EJ, Fedorowicz Z, Christensen R, Lavrijsen AP, Arents BW. Emollients and moisturisers for eczema. Cochrane Database Syst Rev [Internet]. 2017; (2). Available from: http://onlinelibrary.wiley.com/doi/10.1002/14651858.CD012119.pub2/abstract.
54. Wood Heickman L, Davallow Ghajar L, Conaway M, Rogol A. Evaluation of Hypothalamic-Pituitary-Adrenal Axis Suppression following Cutaneous Use of Topical Corticosteroids in Children: A Meta-Analysis. *Horm Res Paediatr*. 2018;**89**(6):389-96.
55. Yan J, Chen S, Wang X, Zhou W, Wang F. Meta-analysis of tacrolimus ointment for atopic dermatitis in pediatric patients. *Pediatr Dermatol*. 2008;**25**(1):117-20.
56. Chan A-W. Cancer risk in patients with atopic dermatitis: protocol for a systematic review [CRD42018092929] 2018 (accessed: 13/07/18). Available from: https://www.crd.york.ac.uk/prospero/display_record.php?RecordID=92929.
57. Fishbein A, Kaat A, Smith P. Systematic review and meta-analysis of randomized clinical trials (RCTs) comparing topical corticosteroids with moisturizers for children (< 2 years old) with atopic dermatitis [CRD42017056060] 2017 (accessed: 13/07/18). Available from: http://www.crd.york.ac.uk/PROSPERO/display_record.php?ID=CRD42017056060.
58. Tan Q, Tan C, Peng W, Shi Y, Xia L. Effects of emollients in the management of atopic dermatitis in pediatric patients a systemic review and meta-analysis [CRD42015016525] 2015 (accessed: 13/07/18). Available from: http://www.crd.york.ac.uk/PROSPERO/display_record.php?ID=CRD42015016525.
59. Yu A, Hong J, Lee M, Hong B. Interventions to improve quality of life in paediatric atopic dermatitis: a systematic review [CRD42015027873] 2015 (accessed: 13/07/18). Available from: http://www.crd.york.ac.uk/PROSPERO/display_record.php?ID=CRD42015027873.
60. Sigurgeirsson B, Boznanski A, Todd G, Vertruyen A, Schuttelaar M, Zhu X, et al. Safety and efficacy of pimecrolimus in atopic dermatitis: a 5-year randomized trial. *Pediatrics* 2015;**135**(4):597-606.
61. Breneman D, Fleischer A, Foley V ea, editors. Clobetasol propionate 0.05% is equivalent as lotion or emollient cream in atopic dermatitis. Presented at the World Congress of Dermatology, 2002; and the European Academy of Dermatology and Venereology, 2003; 2003.
62. Rosso J, Bhambri S. Daily application of fluocinonide 0.1% cream for the treatment of atopic dermatitis. *J Clin Aesthet Dermatol*. 2009;**2**(9):24-32.
63. Binder R. Clinical study of clocortolone pivalate in the treatment of eczema/atopic dermatitis. *Curr Ther Res Clin Exp*. 1977;**21**(6):796-801.

For peer review only - http://bmjopen.bmj.com/site/about/guidelines.xhtml

64. Griffiths C, Van Leent E, Gilbert M, Traulsen J, Cipamyflline Study Group. Randomized comparison of the type 4 phosphodiesterase inhibitor cipamfylline cream, cream vehicle and hydrocortisone 17-butyrate cream for the treatment of atopic dermatitis. *Br J Dermatol*. 2002;**147**(2):299-307.
65. Paller A, Nimmagadda S, Schachner L, Mallory S, Kahn T, Willis I, et al. Fluocinolone acetonide 0.01% in peanut oil: therapy for childhood atopic dermatitis, even in patients who are peanut sensitive. *J Am Acad Dermatol*. 2003;**48**(4):569-77.
66. Pellanda C, Weber M, Bircher A, Surber C. Low-dose triamcinolone acetonide in the phytocosmetic lichtena reduces inflammation in mild to moderate atopic dermatitis. *Dermatology*. 2005;**211**(4):338-40.
67. Dolle S, Hoser D, Rasche C, Loddenkemper C, Maurer M, Zuberbier T, et al. Long-term reduction in local inflammation by a lipid raft molecule in atopic dermatitis. *Allergy*. 2010;**65**(9):1158-65.
68. Abramovits W, Oquendo M. Hydrocortisone butyrate 0.1% cream (proprietary lipid rich cream vehicle) does not significantly suppress hypothalamic-pituitary-adrenal axis and is effective in pediatric patients 3 months and older with extensive atopic dermatitis. *Skinmed*. 2010;**8**(3):150-4.
69. Dohil MA, Alvarez ‐ Connelly E, Eichenfield LF. Fluocinolone acetonide 0.01% in peanut oil: safety and efficacy data in the treatment of childhood atopic dermatitis in infants as young as 3 months of age. *Pediatr Dermatol*. 2009;**26**(3):262-8.
70. Eichenfield L, Ellis CN, Fivenson D, Hebert AA, Dromgoole S, Piacquadio D. Evaluation of adrenal suppression of a lipid enhanced, topical emollient cream formulation of hydrocortisone butyrate 0.1% in treating children with atopic dermatitis. *Pediatr Dermatol*. 2007;**24**(1):81-4.
71. Eichenfield LF, Basu S, Calvarese B, Trancik RJ. Effect of desonide hydrogel 0.05% on the hypothalamic ‐ pituitary ‐ adrenal axis in pediatric subjects with moderate to severe atopic dermatitis. *Pediatr Dermatol*. 2007;**24**(3):289-95.
72. Friedlander S, Hebert A, Allen D. Safety of fluticasone propionate cream 0.05% for the treatment of severe and extensive atopic dermatitis in children as young as 3 months. *J Am Acad Dermatol*. 2002;**46**(3):387-93.
73. Hebert AA, Friedlander SF, Allen DB. Topical fluticasone propionate lotion does not cause HPA axis suppression. *J Pediatr*. 2006;**149**(3):378-82.
74. Hebert AA, Desonide Foam Phase III Clinical Study Group. Desonide foam 0.05%: safety in children as young as 3 months. *J Am Acad Dermatol*. 2008;**59**(2):334-40.
75. Kimball AB, Gold MH, Zib B, Davis MW, Phase CPEFF, Group ICS. Clobetasol propionate emulsion formulation foam 0.05%: review of phase II open-label and phase III randomized controlled trials in steroid-responsive dermatoses in adults and adolescents. *J Am Acad Dermatol*. 2008;**59**(3):448-54. e1.
76. Lucky A, Grote G, Williams J, Tuley M, Czernielewski J, Dolak T, et al. Effect of desonide ointment, 0.05%, on the hypothalamic-pituitary-adrenal axis of children with atopic dermatitis. *Cutis*. 1997;**59**(3):151-3.
77. Moshang T. Prednicarbate emollient cream 0.1% in pediatric patients with atopic dermatitis. *Cutis*. 2001;**68**(1):63-9.
78. Schlessinger J, Miller B, Gilbert R, Plott R, Vanos Study Group. An open-label adrenal suppression study of 0.1% fluocinonide cream in pediatric patients with atopic dermatitis. *Arch Dermatol*. 2006;**142**(12):1568-72.
79. Luger T, Lahfa M, Folster-Holst R, Gulliver W, Allen R, Molloy S, et al. Long-term safety and tolerability of pimecrolimus cream 1% and topical corticosteroids in adults with moderate to severe atopic dermatitis. *J Dermatolog Treat*. 2004;**15**(3):169-78.
80. Mandelin J, Remitz A, Virtanen H, Reitamo S. One-year treatment with 0.1% tacrolimus ointment versus a corticosteroid regimen in adults with moderate to severe atopic dermatitis: A randomized, double-blind, comparative trial. *Acta Derm Venereol*. 2010;**90**(2):170-4.

For peer review only - http://bmjopen.bmj.com/site/about/guidelines.xhtml

81. Reitamo S, Ortonne J, Sand C, Cambazard F, Bieber T, Folster-Holst R, et al. A multicentre, randomized, double-blind, controlled study of long-term treatment with 0.1% tacrolimus ointment in adults with moderate to severe atopic dermatitis. *Br J Dermatol*. 2005;**152**(6):1282-9.
82. Doss N, Kamoun M, Dubertret L, Cambazard F, Remitz A, Lahfa M, et al. Efficacy of tacrolimus 0.03% ointment as second-line treatment for children with moderate-to-severe atopic dermatitis: evidence from a randomized, double-blind non-inferiority trial vs. fluticasone 0.005% ointment. *Pediatr Allergy Immunol*. 2010;**21**(2 Pt 1):321-9.
83. Doss N, Reitamo S, Dubertret L, Fekete G, Kamoun M, Lahfa M, et al. Superiority of tacrolimus 0.1% ointment compared with fluticasone 0.005% in adults with moderate to severe atopic dermatitis of the face: results from a randomized, double-blind trial. *Br J Dermatol*. 2009;**161**(2):427-34.
84. Hofman T, Cranswick N, Kuna P, Boznanski A, Latos T, Gold M, et al. Tacrolimus ointment does not affect the immediate response to vaccination, the generation of immune memory, or humoral and cell-mediated immunity in children. *Arch Dis Child*. 2006;**91**(11):905-10.
85. Luger T, Van Leent E, Graeber M, Hedgecock S, Thurston M, Kandra A, et al. SDZ ASM 981: an emerging safe and effective treatment for atopic dermatitis. *Br J Dermatol*. 2001;**144**(4):788-94.
86. Reitamo S, Harper J, Bos J, Cambazard F, Bruijnzeel-Koomen C, Valk P, et al. 0.03% Tacrolimus ointment applied once or twice daily is more efficacious than 1% hydrocortisone acetate in children with moderate to severe atopic dermatitis: results of a randomized double-blind controlled trial. *Br J Dermatol*. 2004;**150**(3):554-62.
87. Reitamo S, Rustin M, Ruzicka T, Cambazard F, Kalimo K, Friedmann P, et al. Efficacy and safety of tacrolimus ointment compared with that of hydrocortisone butyrate ointment in adult patients with atopic dermatitis. *J Allergy Clin Immunol*. 2002;**109**(3):547-55.
88. Reitamo S, Van Leent E, Ho V, Harper J, Ruzicka T, Kalimo K, et al. Efficacy and safety of tacrolimus ointment compared with that of hydrocortisone acetate ointment in children with atopic dermatitis. *J Allergy Clin Immunol*. 2002;**109**(3):539-46.
89. Sikder M, Al Mamun S, Khan R, Chowdhury A, Khan H, Hoque M. Topical 0.03% tacrolimus ointment, 0.05% clobetasone butyrate cream alone and their combination in older children with atopic dermatitis - An open randomized comparative study. *J Pak Assoc Dermatol*. 2005;**15**(4):304-12.
90. Schneeweiss S, Doherty M, Zhu S, Funch D, Schlienger R, Fernandez-Vidaurre C, et al. Topical treatments with pimecrolimus, tacrolimus and medium- to high-potency corticosteroids, and risk of lymphoma. *Dermatology*. 2009;**219**(1):7-21.
91. Arellano F, Wentworth C, Arana A, Fernandez C, Paul C. Risk of lymphoma following exposure to calcineurin inhibitors and topical steroids in patients with atopic dermatitis. *J Invest Dermatol*. 2007;**127**(4):808-16.
92. Prado de Oliveira Z, Cuce L, Arnone M. Comparative evaluation of efficacy, tolerability and safety of 0.1% topical momethasone furoate and 0.05% desonide in the treatment of childhood atopic dermatitis. *An Bras Dermatol*. 2002;**77**:25-33.
93. Kuokkanen K, Sillantaka I. Alclometasone dipropionate 0.05% vs hydrocortisone 1.0%: potential to induce cutaneous atrophy in children. *Clin Ther*. 1987;**9**(2):223-31.
94. Smitt J, Winterberg D, Oosting J. Treatment of atopic dermatitis with topical corticosteroids in children. Efficacy and systemic effects of triamcinolone acetonide and alclometasone dipropionate. *Eur J Dermatol*. 1993;**3**(7):549-52.
95. Chen H. Clinical observation of Huanglian Qingdai cream for infantile eczema

J Sichuan Trad Chin Med 2011;**29**:88–9.
96. Huang Z, Chen P, Wei W. Observation of efficacy of Chushi Zhiyang cream for atopic dermatitis. *Mod J Integr Trad Chin West Med*. 2010;**19**:2647–8.
97. Dong M. Efficacy of external use of Jingfang mixture in the treatment of children with eczema. *Med J Qilu*. 2012;**27**:75–8.

For peer review only - http://bmjopen.bmj.com/site/about/guidelines.xhtml

98. Hoybye S, Balk Moller S, De Cunha Bang F, Ottevanger V, Veien N. Continuous and intermittent treatment of atopic dermatitis in adults with mometasone furoate versus hydrocortisone 17-butyrate. *Curr Ther Res Clin Exp*. 1991;**50**(1):67-72.
99. Berth-Jones J, Damstra R, Golsch S, Livden J, Van Hooteghem O, Allegra F, et al. Twice weekly fluticasone propionate added to emollient maintenance treatment to reduce risk of relapse in atopic dermatitis: randomised, double blind, parallel group study. *BMJ* 2003;**326**(7403):1367-9.
100. Marchesi E, Rozzoni M, Pini P, Cainelli T. Comparative study of mometasone furoate and betamethasone dipropionate in the treatment of atopic dermatitis. *G Ital Dermatol Venereol*. 1994;**129**(1-2):IX-XII.
101. Koopmans B, Lasthein Andersen B, Mork N, Austad J, Suhonen R, Roders G. Multicentre randomized double-blind study of Locoid Lipocream fatty cream twice daily versus Locoid Lipocream once daily and Locobase once daily. *J Dermatolog Treat*. 1995;**6**(2):103-6.
102. Tharp M. A comparison of twice-daily and once-daily administration of fluticasone propionate cream, 0.05%, in the treatment of eczema. *Cutis*. 1996;**57**(Suppl 2):19-26.
103. Richelli C, Piacentini G, Sette L, Bonizzato M, Andreoli A, Boner A. Clinical efficacy and tolerability of clobetasone 17-butyrate 0.5% lotion in children with atopic dermatitis. *Curr Ther Res Clin Exp*. 1990;**47**(3):413-7.
104. Glazenburg E, Wolkerstorfer A, Gerretsen A, Mulder P, Oranje A. Efficacy and safety of fluticasone propionate 0.005% ointment in the long-term maintenance treatment of children with atopic dermatitis: differences between boys and girls? *Pediatr Allergy Immunol*. 2009;**20**(1):59-66.
105. Hanifin J, Gupta A, Rajagopalan R. Intermittent dosing of fluticasone propionate cream for reducing the risk of relapse in atopic dermatitis patients. *Br J Dermatol*. 2002;**147**(3):528-37.
106. Peserico A, Stadtler G, Sebastian M, Fernandez R, Vick K, Bieber T. Reduction of relapses of atopic dermatitis with methylprednisolone aceponate cream twice weekly in addition to maintenance treatment with emollient: a multicentre, randomized, double-blind, controlled study. *Br J Dermatol*. 2008;**158**(4):801-7.
107. Van Der Meer J, Glazenburg E, Mulder P, Eggink H, Coenraads P. The management of moderate to severe atopic dermatitis in adults with topical fluticasone propionate. The Netherlands Adult Atopic DermatitisStudy Group. *Br J Dermatol*. 1999;**140**(6):1114-21.
108. Devillers A, de Waard-van der Spek F, Mulder P, Oranje A. Treatment of refractory atopic dermatitis using 'wet-wrap'dressings and diluted corticosteroids: results of standardized treatment in both children and adults. *Dermatology*. 2002;**204**(1):50-5.
109. Wolkerstorfer A, Visser R, De Waard van der Spek F, Mulder P, Oranje A. Efficacy and safety of wet - wrap dressings in children with severe atopic dermatitis: influence of corticosteroid dilution. *Br J Dermatol*. 2000;**143**(5):999-1004.
110. Janmohamed S, Oranje A, Devillers A, Rizopoulos D, van Praag M, Van Gysel D, et al. The proactive wet-wrap method with diluted corticosteroids versus emollients in children with atopic dermatitis: a prospective, randomized, double-blind, placebo-controlled trial. *J Am Acad Dermatol*. 2014;**70**(6):1076-82.
111. Beattie P, Lewis-Jones M. A pilot study on the use of wet wraps in infants with moderate atopic eczema. *Clin Exp Dermatol*. 2004;**29**(4):348-53.
112. Hindley D, Galloway G, Murray J, Gardener L. A randomised study of "wet wraps" versus conventional treatment for atopic eczema. *Arch Dis Child*. 2006;**91**(2):164-8.
113. Goodyear H, Spowart K, Harper J. 'Wet-wrap'dressings for the treatment of atopic eczema in children. *Br J Dermatol*. 1991;**125**(6):604.
114. McGowan R, Tucker P, Joseph D, Wallace A, Hughes I, Burrows N, et al. Short-term growth and bone turnover in children undergoing occlusive steroid ('wet-wrap') dressings for treatment of atopic eczema. *J Dermatolog Treat*. 2003;**14**(3):149-52.
115. NICE. Atopic eczema in under 12s: diagnosis and management. Clinical guideline [CG57]. 2007 [Available from: https://www.nice.org.uk/guidance/cg57.

For peer review only - http://bmjopen.bmj.com/site/about/guidelines.xhtml

116. Chalmers JR, Axon E, Harvey J, Santer M, Ridd MJ, Lawton S, et al. Different strategies for using topical corticosteroids in people with eczema. *Cochrane Database Syst Rev*. 2019(6).

For peer review only

Tables

Table 1: Characteristics of included systematic reviews

First author, publication year	Type of review	Review contained safety data from RCTs for comparisons of interest?	Review contained safety data from observational studies?	AMSTAR 2 rating
Ashcroft 2005 [23]	Non-Cochrane	Yes (TCS versus TCI)	No	Critically low[1 3 6 7]
Ashcroft 2007 [22]	Cochrane	Yes (TCS versus TCI)	Yes (TCS versus TCI)	Moderate [8]
Barnes 2015 [24]	Non-Cochrane	Yes (TCS versus vehicle, TCS versus TCI, TCS versus another TCS)	Yes (single arm TCS studies)	Critically low [1 2 3 4 6]
Braham 2010 [25]	Non-Cochrane	Yes (occluded TCS versus non-occluded TCS)	Yes (occluded TCS)	Critically low [1 2 3 4 6]
Broeders 2016 [26]	Non-Cochrane	Yes (TCS versus TCI)	No	Critically low [1 3 5 6]
Callen 2007 [27]	Non-Cochrane	Yes (TCS versus vehicle, TCS versus another TCS)	Yes (single arm studies or comparing TCS potencies)	Critically low [1 2 3 4 6]
Chen 2010 [28]	Non-Cochrane	Yes (TCS versus TCI)	No	Critically low [1 3 6]
Cury Martins 2015 [29]	Cochrane	Yes (TCS versus TCI)	Yes (TCS versus TCI)	Moderate [8]
De Tiedra 1997 [30]	Non-Cochrane	Yes (TCS versus another TCS)	Yes (usually only reported data from one arm of RCTs)	Critically low [1 2 3 4 6]
Devillers 2006 [31]	Non-Cochrane	Yes (occluded TCS versus non-occluded TCS)	Yes (occluded TCS)	Critically low [1 2 3 4 6]
Dong 2017 [32]	Non-Cochrane	Yes (TCS versus TCI)	No	Critically low [1 2 3 4 6]

For peer review only - http://bmjopen.bmj.com/site/about/guidelines.xhtml

Eichenfield 2014 [33]	Non-Cochrane	No	Yes (different TCS potencies)	Critically low [1,2,3,4,6]
Feldman 2005 [34]	Non-Cochrane	Yes (TCS versus vehicle)	No	Critically low [1,2,3,4,6]
Frangos 2008 [35]	Non-Cochrane	Yes (TCS versus vehicle)	Yes (single arm studies)	Critically low [1,2,3,4,6]
Froeschl 2007 [36]	GMS HTA report	Yes (TCS versus vehicle, TCS versus TCI, TCS versus another TCS)	No	Critically low [1,2,4,6]
Gonzalez-Lopez 2017 [37]	Non-Cochrane	Yes (occluded TCS versus non-occluded TCS)	No	Critically low [1,3]
Green 2004 [7]	HTA report	Yes (once daily versus twice daily TCS use)	No	Low [2]
Gu 2013 [39]	Cochrane	Yes (TCS versus topical CHM)	No	High
Gu 2014 [38]	Non-Cochrane	Yes (TCS versus topical CHM)	No	Critically low [1,2,3,7]
Hajar 2015 [40]	Non-Cochrane	No	Yes (case series or case reports)	Critically low [2,3,6]
Hoare 2000 [41]	NIHR HTA report	Yes (TCS versus vehicle, TCS versus another TCS)	No	Low [1]
Iskedjian 2004 [42]	Non-Cochrane	Yes (TCS versus vehicle, TCS versus TCI)	No	Critically low [1,3,6]
Juhasz 2017 [43]	Non-Cochrane	No	Yes (social media analysis)	Critically low [1,2,3,4,6]
Legendre 2015 [44]	Non-Cochrane	No	Yes (TCS versus TCI)	Critically low [1,2,3,6]
Li 2007 [45]	Non-Cochrane	Yes (TCS versus TCI)	No	Critically low [1,3,6]
Nankervis 2016 [46]	NIHR HTA report	Yes (TCS versus vehicle, TCS versus emollients, TCS versus TCI, TCS versus another TCS, once a day versus twice a day use, proactive TCS to prevent flares ("weekend therapy") versus vehicle, occluded TCS versus non-occluded TCS)	No	Low [3]
Penaloza Hidalgo 2004 [47]	West Midlands HTA report	Yes (TCS versus TCI)	No	Low [6]
Schmitt 2011 [8]	Non-Cochrane	Yes (proactive TCS to prevent flares ("weekend therapy") versus vehicle)	No	Critically low [3,6]
Sidbury 2014 [48]	Non-Cochrane	Yes (proactive TCS to prevent flares ("weekend therapy") versus vehicle)	No	Critically low [1,2,3,4,6]
Siegfried 2016 [49]	Non-Cochrane	Yes (TCS versus vehicle, TCS versus TCI, TCS versus another TCS)	No	Critically low [1,2,3,4,6]
Singh 2012 [50]	Non-Cochrane	Yes (TCS versus vehicle, TCS versus TCI, TCS versus another TCS)	Yes (single arm study)	Critically low [1,2,6]
Svensson 2011 [51]	Non-Cochrane	Yes (TCS versus TCI)	No	Critically low [1,3,6,7]
Tang 2014 [52]	Non-Cochrane	Yes (proactive TCS to prevent flares ("weekend therapy") versus vehicle)	No	Critically low [1,3,4,6]
van Zuuren 2017 [53]	Cochrane	Yes (TCS versus emollient)	No	High
Wood Heickman 2018 [54]	Non-Cochrane	No	Yes (single arm cohort studies)	Critically low [1,2,3,4,6,7]
Yan 2008 [55]	Non-Cochrane	Yes (TCS versus TCI)	No	Critically low [1,3,6,7]

Footnotes: Additional data on TCS including potency can be found in the Appendix 6. Key: TCS=Topical corticosteroid; TCI=Topical calcineurin inhibitors; CHM=Chinese herbal medicine; HTA=Health Technology Assessment; GMS=German Medical Science; NIHR=National Institute for Health Research.

AMSTAR 2 ratings – reasons for downgrading the quality of the review: [1]No protocol; [2]Search strategy not comprehensive; [3]No list of excluded studies with reasons; [4]Risk of bias not assessed; [5]inappropriate meta-analysis methods; [6]Risk of bias assessments not included in the interpretation of the results; [7]publication bias not explored in the meta-analysis.

For peer review only

For peer review only - http://bmjopen.bmj.com/site/about/guidelines.xhtml

Table 2: summary of main findings for key safety outcomes

	Cutaneous adverse events	Systemic adverse events
**How safe are TCS compared to emollient or vehicle, or no comparison?**<br><br>12 reviews:<br><br>1 moderate quality<br>2 low quality<br>9 critically low quality	• **Skin thinning:** No significant differences in 2 RCTs of 2-4 weeks compared to emollient/vehicle: (i) 0/196 children with very potent TCS and 0/33 vehicle, (ii) 6/109 very potent TCS vs 2/50 vehicle, p=0.69. Very low rates.<br><br>• **Other cutaneous adverse events:** No significant differences in 5 RCTs (2-4 weeks) between TCS (various potencies) and emollient/vehicle (n=172, plus one study, n not specified). Low event rates.	• **Biochemical evidence of adrenal suppression:** Meta-analysis (11 *observational* studies, max 4 weeks) - 20/522 children with any potency TCS (3.8%, 95% CI 2.4% to 5.8%), 3/148 children (2%) with mild potency TCS. Effects were transient.<br><br>• **Clinical symptoms or signs of adrenal suppression:** none observed in same as above observational studies.
**How safe are TCS compared to topical calcineurin inhibitors (TCI)?**<br><br>7 reviews:<br><br>1 moderate quality<br>1 low quality<br>5 critically low quality	• **Skin thinning:** Higher with TCS than TCI (meta-analysis of 4 RCTs: RR 4.86, 95% 1.06 to 22.28, n=4128) but very low rate (8/2068, 7 of which were using potent TCS).<br><br>• **Other cutaneous adverse events:** No difference in **skin infections** between TCS and TCI (8 RCTs). **Skin burning** and **pruritus** lower with TCS than TCI: meta-analysis of 10 RCTs: **burning** - RR 0.31, 95% CI 0.23 to 0.40 (n=4211), **pruritus** - RR 0.68, 95% CI 0.56 to 0.82(n=4211).	• **Growth rate:** no differences in growth rates tween TCS and TCI (1 RCT of 2418 children with 5 years follow up).<br><br>• **Lymphoma:** no cases reported in 1 same large RCT as above. One cohort study (n=1,438,333, approx. 4years follow up) - very small *non-significant* increase with TCI *and* TCS compared to general population. One case-control study - no increased risk with TCS or TCIs (294 cases/293,000 controls).

For peer review only - http://bmjopen.bmj.com/site/about/guidelines.xhtml

**How safe are once daily TCS compared with twice daily application?**<br><br>2 reviews:<br><br>2 low quality	• **Skin thinning:** no cases using once daily vs twice daily potent TCS for 3-weeks (1 RCT, 94 adults).<br><br>• **Other cutaneous adverse events:** no significant difference between groups in **telangiectasia**, **folliculitis**, or **burning/itching/stinging** (4 RCTs, 4-16 weeks follow up740 older children/adults).	• **Biochemical evidence of adrenal suppression:** no significant differences between once and twice daily moderate/potent TCS up to 2 weeks in children (2 RCTs, n=129).
**How safe are TCS used proactively to prevent flares ("weekend therapy")?**<br><br>3 reviews:<br><br>3 critically low quality	• **Skin thinning:** no cases with 16-20 weeks of 2 days/week of potent TCS versus vehicle (5 RCTs, n=993).<br><br>• **Other cutaneous adverse events:** no significant differences between groups, including **folliculitis** and transient **telangiectasia**, with potent TCS (16-20 weeks) compared to either vehicle or another TCS (2 RCTs, n=423). Events were uncommon in both groups.	• **Biochemical evidence of adrenal suppression:** no cases with 16 weeks of 2 days/week of potent TCS (2 RCTs, n=129). Possible adrenal suppression in 2/44 children with potent TCS compared to zero using vehicle (1 RCT, 20 weeks).
**How safe are TCS used under occlusion?**<br><br>4 reviews:<br><br>1 high quality<br>3 critically low quality	• **Skin thinning:** no cases in two *observational* studies (potent TCS + wet wrap, 1-2 weeks, n=44).<br><br>• **Other cutaneous adverse events:** One case of **striae** in two *observational* studies, n-44. More **folliculitis** with diluted potent TCS (10/19 children) compared to emollient (2/20), both under wet	• **Biochemical evidence of adrenal suppression:** reported in 3 *observational* studies (2-14 days of diluted potent TCS under wet-wraps in 74 children) but rates not specified in review. Described as transient in 2 studies.

For peer review only - http://bmjopen.bmj.com/site/about/guidelines.xhtml

	wrap (1 RCT). A meta-analysis (2 RCTs, n=69) of wet wrap vs no wet wrap (mild potency) - no significant difference in **cutaneous adverse events**.	• **Growth or bone turnover:** no effect seen in 1 small short-term *observational* study (potent TCS wet-wrap in 8 children, (median follow-up 12 weeks).

For peer review only

For peer review only - http://bmjopen.bmj.com/site/about/guidelines.xhtml

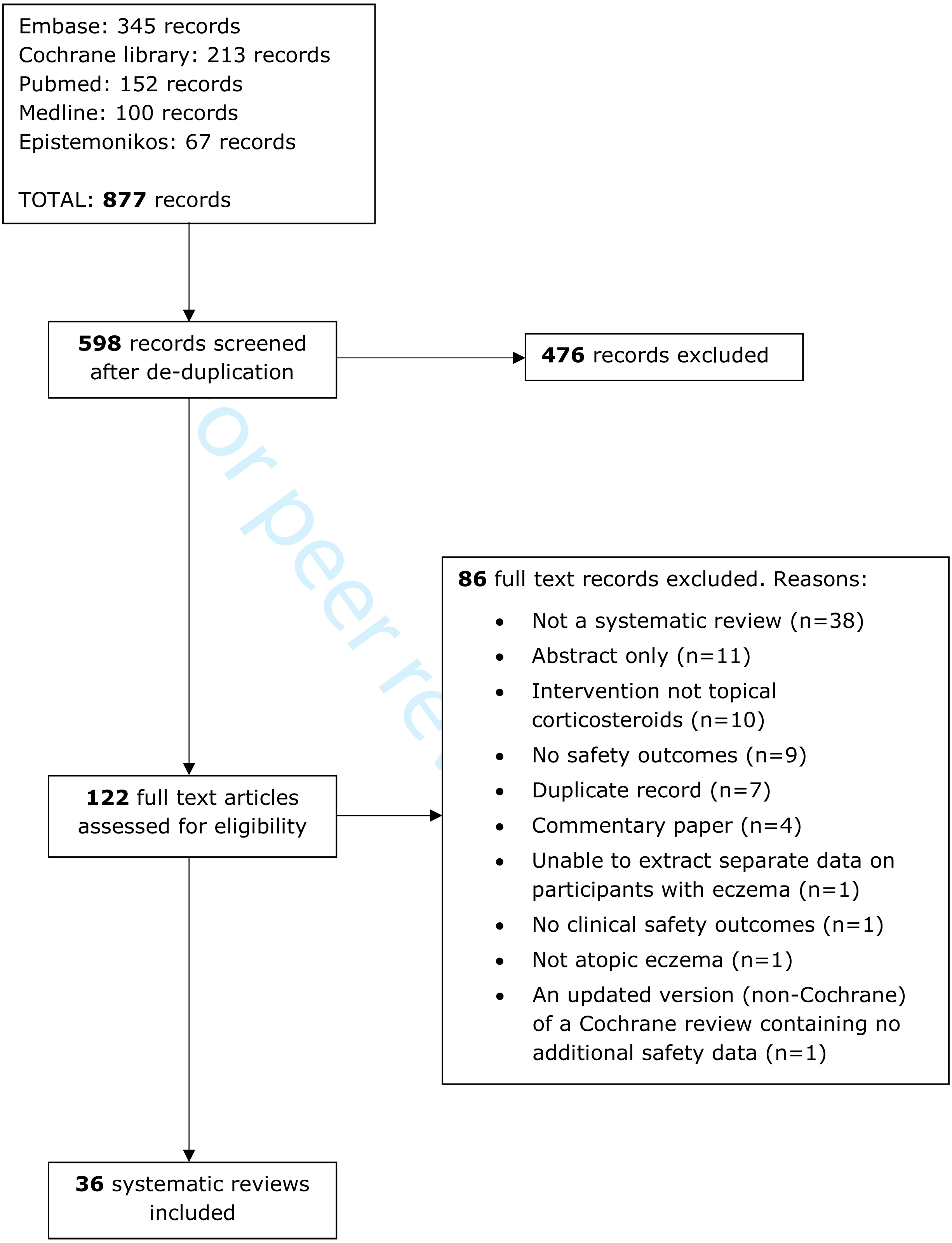
Embase: 345 records
Cochrane library: 213 records
Pubmed: 152 records
Medline: 100 records
Epistemonikos: 67 records
TOTAL: **877** records
598 records screened after de-duplication
476 records excluded
86 full text records excluded. Reasons:
Not a systematic review (n=38)
Abstract only (n=11)
Intervention not topical corticosteroids (n=10)
No safety outcomes (n=9)
Duplicate record (n=7)
Commentary paper (n=4)
Unable to extract separate data on participants with eczema (n=1)
No clinical safety outcomes (n=1)
Not atopic eczema (n=1)
An updated version (non-Cochrane) of a Cochrane review containing no additional safety data (n=1)
122 full text articles assessed for eligibility
36 systematic reviews included

Appendix 1: Search strategies

Search facets for searches:

Topical steroids
Eczema
Systematic reviews

PubMed search

Uses PubMed Clinical Queries systematic review filter (command systematic[sb]):
https://www.nlm.nih.gov/bsd/pubmed_subsets/sysreviews_strategy.html
(steroid* OR corticosteroid* OR glucocorticosteroid* OR glucocorticoid* OR glucocorticoids[MeSH Terms] OR alclometasone OR alclomethasone OR amcinonide OR beclometasone OR beclomethasone OR beclomethasone[MeSH Terms] OR betametasone OR betamethasone OR betamethasone[MeSH Terms] OR clobetasol OR clobetasol[MeSH Terms] OwR clobetasone OR desonide OR desonide[MeSH Terms] OR desoximetasone OR desoximetasone[MeSH Terms] OR diflorasone OR diflucortolone OR diflucortolone[MeSH Terms] OR fludroxycortide OR flumetasone OR flumethasone OR flumethasone[MeSH Terms] OR fluocinolone OR fluocinolone acetonide[MeSH Terms] OR fluocinonide OR fluocinonide[MeSH Terms] OR fluocortolone OR fluocortolone[MeSH Terms] OR flurandrenolide OR flurandrenolone OR flurandrenolone[MeSH Terms] OR fluticasone OR halcinonide OR halcinonide[MeSH Terms] OR halobetasol OR halometasone OR hydrocortisone OR hydrocortisone[MeSH Terms] OR methylprednisolone OR methylprednisolone[MeSH Terms] OR mometasone OR triamcinolone OR triamcinolone[MeSH Terms]) AND ("dermatitis, atopic"[MeSH Terms] OR "eczema"[MeSH Terms] OR "neurodermatitis"[MeSH Terms] OR eczema OR "atopic dermatitis" OR neurodermatitis) AND (systematic[sb] OR "systematic review")

Ovid MEDLINE search

Uses SIGN MEDLINE systematic review filter:
http://www.sign.ac.uk/search-filters.html
Ovid MEDLINE(R) Epub Ahead of Print, In-Process & Other Non-Indexed Citations, Ovid MEDLINE(R) Daily, Ovid MEDLINE and Versions(R)
MEDLINE eczema steroid systematic reviews

1. Meta-Analysis as Topic/
2. meta analy$.tw.
3. metaanaly$.tw.
4. Meta-Analysis/
5. (systematic adj (review$1 or overview$1)).tw.
6. exp Review Literature as Topic/
7. or/1-6
8. cochrane.ab.
9. embase.ab.
10. (psychlit or psyclit).ab.
11. (psychinfo or psycinfo).ab.
12. (cinahl or cinhal).ab.
13. science citation index.ab.
14. bids.ab.
15. cancerlit.ab.
16. or/8-15
17. reference list$.ab.
18. bibliograph$.ab.

For peer review only - http://bmjopen.bmj.com/site/about/guidelines.xhtml

19. hand-search$.ab.
20. relevant journals.ab.
21. manual search$.ab.
22. or/17-21
23. selection criteria.ab.
24. data extraction.ab.
25. 23 or 24
26. Review/
27. 25 and 26
28. Comment/
29. Letter/
30. Editorial/
31. animal/
32. human/
33. 31 not (31 and 32)
34. or/28-30,33
35. 7 or 16 or 22 or 27
36. 35 not 34
37. steroid$.mp.
38. corticosteroid$.mp.
39. glucocorticosteroid$.mp.
40. glucocorticoid$.mp.
41. exp Glucocorticoids/
42. alclometasone.mp.
43. alclomethasone.mp.
44. amcinonide.mp.
45. beclometasone.mp.
46. beclomethasone.mp.
47. exp Beclomethasone/
48. betametasone.mp.
49. betamethasone.mp.
50. exp Betamethasone/
51. clobetasol.mp.
52. exp Clobetasol/
53. clobetasone.mp.
54. desonide.mp.
55. exp Desonide/
56. desoximetasone.mp.
57. exp Desoximetasone/
58. diflorasone.mp.
59. diflucortolone.mp.
60. exp Diflucortolone/
61. fludroxycortide.mp.
62. flumetasone.mp.
63. flumethasone.mp.
64. exp Flumethasone/
65. fluocinolone.mp.
66. exp Fluocinolone Acetonide/
67. fluocinonide.mp.

For peer review only

68. exp Fluocinonide/
69. fluocortolone.mp.
70. exp Fluocortolone/
71. flurandrenolide.mp.
72. flurandrenolone.mp.
73. exp Flurandrenolone/
74. fluticasone.mp.
75. halcinonide.mp.
76. exp Halcinonide/
77. halobetasol.mp.
78. halometasone.mp.
79. hydrocortisone.mp.
80. exp Hydrocortisone/
81. methylprednisolone.mp.
82. exp methylprednisolone/
83. mometasone.mp.
84. triamcinolone.mp.
85. exp Triamcinolone/
86. or/37-85
87. exp dermatitis, atopic/
88. exp eczema/
89. exp neurodermatitis/
90. eczema.mp.
91. atopic dermatitis.mp.
92. neurodermatitis.mp.
93. or/87-92
94. 36 and 86 and 93

Ovid Embase search

Uses SIGN Embase systematic review filter:
http://www.sign.ac.uk/search-filters.html
Embase 1974 to 2017 October 23
Embase eczema steroid systematic reviews

1. exp Meta Analysis/
2. ((meta adj analy$) or metaanalys$).tw.
3. (systematic adj (review$1 or overview$1)).tw.
4. or/1-3
5. cancerlit.ab.
6. cochrane.ab.
7. embase.ab.
8. (psychlit or psyclit).ab.
9. (psychinfo or psycinfo).ab.
10. (cinahl or cinhal).ab.
11. science citation index.ab.
12. bids.ab.
13. or/5-12
14. reference lists.ab.
15. bibliograph$.ab.
16. hand-search$.ab.

For peer review only - http://bmjopen.bmj.com/site/about/guidelines.xhtml
For peer review only

17. manual search$.ab.
18. relevant journals.ab.
19. or/14-18
20. data extraction.ab.
21. selection criteria.ab.
22. 20 or 21
23. review.pt.
24. 22 and 23
25. letter.pt.
26. editorial.pt.
27. animal/
28. human/
29. 27 not (27 and 28)
30. or/25-26,29
31. 4 or 13 or 19 or 24
32. 31 not 30
33. steroid$.mp.
34. corticosteroid$.mp.
35. exp corticosteroid/
36. glucocorticosteroid$.mp.
37. glucocorticoid$.mp.
38. exp glucocorticoid/
39. alclometasone.mp.
40. alclomethasone.mp.
41. amcinonide.mp.
42. beclometasone.mp.
43. beclomethasone.mp.
44. betametasone.mp.
45. betamethasone.mp.
46. clobetasol.mp.
47. clobetasone.mp.
48. desonide.mp.
49. desoximetasone.mp.
50. diflorasone.mp.
51. diflucortolone.mp.
52. fludroxycortide.mp.
53. flumetasone.mp.
54. flumethasone.mp.
55. fluocinolone.mp.
56. fluocinonide.mp.
57. fluocortolone.mp.
58. flurandrenolide.mp.
59. flurandrenolone.mp.
60. fluticasone.mp.
61. halcinonide.mp.
62. halobetasol.mp.
63. halometasone.mp.
64. hydrocortisone.mp.
65. methylprednisolone.mp.

For peer review only

66. mometasone.mp.
67. triamcinolone.mp.
68. or/33-67
69. exp atopic dermatitis/
70. exp eczema/
71. exp neurodermatitis/
72. eczema.mp.
73. atopic dermatitis.mp.
74. neurodermatitis.mp.
75. or/69-74
76. 32 and 68 and 75

Epistemonikos

(steroid* OR corticosteroid* OR glucocorticosteroid* OR glucocorticoid* OR alclometasone OR alclomethasone OR amcinonide OR beclometasone OR beclomethasone OR betametasone OR betamethasone OR clobetasol OR clobetasone OR desonide OR desoximetasone OR diflorasone OR diflucortolone OR fludroxycortide OR flumetasone OR flumethasone OR fluocinolone OR fluocinonide OR fluocortolone OR flurandrenolide OR flurandrenolone OR fluticasone OR halcinonide OR halobetasol OR halometasone OR hydrocortisone OR methylprednisolone OR mometasone OR triamcinolone) AND (eczema OR "atopic dermatitis" OR neurodermatitis)

Enter search into advanced search and choose "**systematic review**" from drop-down box for "Publication type":

https://www.epistemonikos.org/advanced_search?q=(steroid*%20OR%20corticosteroid*%20OR%20glucocorticosteroid*%20OR%20glucocorticoid*%20OR%20alclometasone%20OR%20alclomethasone%20OR%20amcinonide%20OR%20beclometasone%20OR%20beclomethasone%20OR%20betametasone%20OR%20betamethasone%20OR%20clobetasol%20OR%20clobetasone%20OR%20desonide%20OR%20desoximetasone%20OR%20diflorasone%20OR%20diflucortolone%20OR%20fludroxycortide%20OR%20flumetasone%20OR%20flumethasone%20OR%20fluocinolone%20OR%20fluocinonide%20OR%20fluocortolone%20OR%20flurandrenolide%20OR%20flurandrenolone%20OR%20fluticasone%20OR%20halcinonide%20OR%20halobetasol%20OR%20halometasone%20OR%20hydrocortisone%20OR%20methylprednisolone%20OR%20mometasone%20OR%20triamcinolone)%20AND%20(eczema%20OR%20%22atopic%20dermatitis%22%20OR%20neurodermatitis)&protocol=no&classification=systematic-review

Cochrane Library

(steroid* OR corticosteroid* OR glucocorticosteroid* OR glucocorticoid* OR [mh "glucocorticoids"] OR alclometasone OR alclomethasone OR amcinonide OR beclometasone OR beclomethasone OR betametasone OR betamethasone OR clobetasol OR clobetasone OR desonide OR desoximetasone OR diflorasone OR diflucortolone OR fludroxycortide OR flumetasone OR flumethasone OR fluocinolone OR fluocinonide OR fluocortolone OR flurandrenolide OR flurandrenolone OR fluticasone OR halcinonide OR halobetasol OR halometasone OR hydrocortisone OR methylprednisolone OR mometasone OR triamcinolone) AND ([mh "eczema"] OR [mh "dermatitis, atopic"] OR [mh "neurodermatitis"] OR eczema OR "atopic dermatitis" OR neurodermatitis)

"Search all text" option chosen.

Cochrane Reviews, **Other Reviews** (i.e. DARE), and **Technology Assessments** (i.e. HTA) chosen.

For peer review only - http://bmjopen.bmj.com/site/about/guidelines.xhtml

Appendix 2 - list of excluded studies with reasons

Excluded study	Reason for exclusion
Abramovits 2005 [1]	Not a systematic review
Abramovits 2006 [2]	Not a systematic review
Anonymous 1995 [3]	Not a systematic review
Anonymous 1999 [4]	Not a systematic review
Anonymous 2004 [5]	Not a systematic review
Anonymous 2005 [6]	Abstract
Anonymous 2007 [7]	Not a systematic review
Anonymous 2015 [8]	Abstract
Anonymous 2015 [9]	Abstract
Aslam 2014 [10]	Not a systematic review
Barfield 2017 [11]	Wrong intervention (not topical corticosteroids)
Batchelor 2010 [12]	Not a systematic review
Bath-Hextall 2010 [13]	Updated version of a Cochrane review (non-Cochrane) but no additional safety data.
Bigby 2001 [14]	Commentary paper
Bonchak 2017 [15]	Wrong intervention (not topical corticosteroids)
Birnie 2008 [16]	Wrong intervention (not topical corticosteroids)
Boucher 2001 [17]	Not a systematic review
Broersen 2015 [18]	Unable to extract separate data for atopic eczema patients
Cameron 2000 [19]	Commentary paper
Carbone 2010 [20]	Not a systematic review
Chavigny 2005 [21]	Not a systematic review
Chi 2009 [22]	Unable to extract separate data for atopic eczema patients
Chi 2015 [23]	Unable to extract separate data for atopic eczema patients
Chia 2015 [24]	Not a systematic review
Chu 1995 [25]	Not a systematic review
Conroy 2004 [26]	Not a systematic review
Das 2017 [27]	Not a systematic review
El-Batawy 2009 [28]	No safety outcome
Fleischer Jr 2010 [29]	Wrong intervention (not topical corticosteroids)
Frohna 2005 [30]	Commentary paper
Froschl 2007 [31]	Duplicate record of an included systematic review
Furue 2006 [32]	Not a systematic review
Furue 2006 [33]	Not a systematic review
Garside 2005 [34]	No safety outcome
Goustas 2003 [35]	Not a systematic review
Green 2005 [36]	Duplicate record of an included systematic review
Green 2004 [37]	Duplicate record of an included systematic review
Halling-Overgaard 2017 [38]	Skin atrophy is not assessed clinically in this review
Health Technology Assessment Database 2004 [39]	Not a systematic review
Health Technology Assessment Database 2004 [40]	Abstract – unable to find the full publication
Health Technology Assessment Database 2001 [41]	Abstract – unable to find the full publication
Health Technology Assessment Database 2004 [42]	Abstract – unable to find the full publication
Hannuksela 2000 [43]	Wrong intervention (not topical corticosteroids)
Hebert 2006 [44]	Wrong intervention (not topical corticosteroids)
Hoare 2000 [45]	Duplicate record of an included systematic review

For peer review only

Hon 2011 [46]	Wrong intervention (not topical corticosteroids)
Hulshof 2017 [47]	Wrong intervention (not topical corticosteroids)
Hussain 2016 [48]	Not a systematic review
Kaufman 2016 [49]	Abstract
Legendre 2015 [50]	Abstract
Li 2017 [51]	Abstract
Li 2017 [52]	No safety outcome
Meffert 1999 [53]	Not a systematic review
Mooney 2015 [54]	Not a systematic review
Murashkin 2016 [55]	Not a systematic review
Nankervis 2013 [56]	Abstract
Nankervis 2016 [57]	Duplicate record of an included systematic review
Nankervis 2017 [58]	Duplicate record of an included systematic review
Nowak 2017 [59]	No safety outcome
Orlow 2007 [60]	Not a systematic review
Pan 2013 [61]	No safety outcome
Park-Wyllie 2000 [62]	Unable to extract separate data for atopic eczema patients
Phipatanakul 2006 [63]	Commentary paper
Radovic 2017 [64]	Wrong intervention (not topical corticosteroids)
Ricci 2007 [65]	Not a systematic review
Ruzicka 1999 [66]	Wrong intervention (not topical corticosteroids)
Sanchez 2014 [67]	Not a systematic review
Schiffner 2003 [68]	No safety outcome
Schmitt 2011 [69]	Not a systematic review
Schmitt 2011 [70]	Duplicate record of an included systematic review
Sher 2012 [71]	Abstract
Sher 2012 [72]	No safety outcome
Siegfried 2013 [73]	Not a systematic review
Siegfried 2018 [74]	Not a systematic review
Silverberg 2014 [75]	Not a systematic review
Simpson 2010 [76]	Not a systematic review
Spada 2018 [77]	Not a systematic review
Torii 2003 [78]	No safety outcome
Torley 2013 [79]	Not a systematic review
Van Zuuren 2017 [80]	No safety outcome (abridged Cochrane review)
Wat 2014 [81]	Wrong patient population (not atopic eczema)
Wellington 2004 [82]	Not a systematic review
Williams 2007 [83]	Not a systematic review
Williams 2008 [84]	Not a systematic review
Williams 2010 [85]	Not a systematic review
Wollenberg 2018 [86]	Not a systematic review

References

1. Abramovits W, Boguniewicz M, Paller A, Whitaker-Worth D, Prendergast M, Tokar M, et al. The economics of topical immunomodulators for the treatment of atopic dermatitis. *Pharmacoeconomics*. 2005;**23**(6):543-66.

2. Abramovits W, Hung P, Tong K. Efficacy and economics of topical calcineurin inhibitors for the treatment of atopic dermatitis. *Am J Clin Dermatol*. 2006;**7**(4):213-22.
3. Anonymous. Once-a-day topical corticosteroids. *Drug Ther Bull*. 1995;**33**(3):21-2.
4. Anonymous. Using topical corticosteroids in general practice. *MeReC Bulletin*. 1999;**10**(6):1-5.
5. Anonymous. Pimecrolimus: Me-too: Too many risks, not beneficial enough in atopic dermatitis. *Prescrire International*. 2004;**13**(74):209+12.
6. Anonymous. Once-daily topical steroid dosing effective for atopic eczema. *J Fam Pract*. 2005;**54**(6):499-500.
7. Anonymous. Atopic dermatitis in the infant and child. [French, English]. *Nouvelles Dermatologiques*. 2007;**26**(SUPPL. 1):3-9.
8. Anonymous. Abstracts of the Australasian College of Dermatologists 48th Annual Scientific Meeting. *Australasian Journal of Dermatology Conference: 48th Annual Scientific Meeting of the Australasian College of Dermatologists Adelaide, SA Australia Conference Publication:*. 2015;**56**(no pagination).
9. Anonymous. 14th International Kidney Cancer Symposium. *BJU International Conference: 14th International Kidney Cancer Symposium Miami, FL United States Conference Publication:*. 2015;**116**(no pagination).
10. Aslam I, Sandoval L, Feldman S. What's new in the topical treatment of allergic skin diseases. *Curr Opin Allergy Clin Immunol*. 2014;**14**(5):436-50.
11. Barfield A, Brown H, Pernell P, Woodard J. Effectiveness of emollient therapy in pediatric patients with atopic dermatitis. *J Dermatol Nurses Assoc*. 2017;**9**(3):123-8.
12. Batchelor J, Grindlay D, Williams H. What's new in atopic eczema? An analysis of systematic reviews published in 2008 and 2009. *Clin Exp Dermatol*. 2010;**35**(8):823-7; quiz 7.
13. Bath-Hextall F, Birnie A, Ravenscroft J, Williams H. Interventions to reduce Staphylococcus aureus in the management of atopic eczema: An updated Cochrane review. *Br J Dermatol*. 2010;**163**(1):12-26.
14. Bigby M. A thorough systematic review of treatments for atopic eczema. *Arch Dermatol*. 2001;**137**(12):1635-6.
15. Bonchak J, Thareja S, Chen S, Quave C. Botanical Complementary and Alternative Medicine for Pruritus: a Systematic Review. *Curr Dermatol Rep*. 2017;**6**(4):248-55.
16. AJ B, FJ BH, JC R, HC W. Interventions to reduce Staphylococcus aureus in the management of atopic eczema. *Cochrane Database of Systematic Reviews*. 2008(3):CD003871.
17. Boucher M. Tacrolimus ointment for the treatment of atopic dermatitis (Structured abstract)2001; (4):[4 p.]. Available from: http://onlinelibrary.wiley.com/o/cochrane/clhta/articles/HTA-32001000957/frame.html.
18. Broersen L, Pereira A, Jorgensen J, Dekkers O. Adrenal insufficiency in corticosteroids use: Systematic review and meta-analysis. *J Clin Endocrinol Metab* 2015;**100**(6):2171-80.
19. Cameron F. 'Down to skin and bone'. *Australas J Dermatol*. 2000;**41**(3):146-8.
20. Carbone A, Siu A, Patel R. Pediatric atopic dermatitis: A review of the medical management. *Ann Pharmacother*. 2010;**44**(9):1448-58.
21. Chavigny J. The place of therapeutic education in the treatment of atopic dermatitis in children. [French]. *Ann Dermatol Venereol*. 2005;**132**(SPEC. ISS. 1):1S116-1S20.
22. Chi C, Lee C, Wojnarowska F, Kirtschig G. Safety of topical corticosteroids in pregnancy. *Cochrane Database of Systematic Reviews*. 2009;**(3) (no pagination)**(CD007346).
23. Chi C-C, Wang S-H, Wojnarowska F, Kirtschig G, Davies E, Bennett C. Safety of topical corticosteroids in pregnancy2015; (10). Available from: http://onlinelibrary.wiley.com/doi/10.1002/14651858.CD007346.pub3/abstract.
24. Chia B, Tey H. Systematic review on the efficacy, safety, and cost-effectiveness of topical calcineurin inhibitors in atopic dermatitis. *Dermatitis*. 2015;**26**(3):122-32.
25. Chu A, Munn S. Fluticasone propionate in the treatment of inflammatory dermatoses. *Br J Clin Pract*. 1995;**49**(3):131-3.
26. Conroy S. New products for eczema. *Arch Dis Child Educ Pract Ed*. 2004;**89**(1):ep23-ep6.
27. Das A, Panda S. Use of Topical Corticosteroids in Dermatology: An Evidence-based Approach. *Indian J Dermatol*. 2017;**62**(3):237-50.
28. El-Batawy M, Bosseila M, Mashaly H, Hafez V. Topical calcineurin inhibitors in atopic dermatitis: a systematic review and meta-analysis. *J Dermatol Sci*. 2009;**54**(2):76-87.
29. Fleischer Jr A, Boguniewicz M. An approach to pruritus in atopic dermatitis: A critical systematic review of the tacrolimus ointment literature. *J Drugs Dermatol*. 2010;**9**(5):488-98.

For peer review only

30. Frohna J. Efficacy and tolerability of topical pimecrolimus and tacrolimus in the treatment of atopic dermatitis: Meta-analysis of randomised controlled trials. *J Pediatr*. 2005;**147**(1):126.
31. Froschl B, Arts D, Leopold C. Corticosteroid therapy in the treatment of pediatric patients with atopic dermatitis. *GMS Health Technol Assess*. 2007;**3**:Doc09.
32. Furue M. [Topical steroids and tacrolimus for the treatment of atopic dermatitis]. *Fukuoka Igaku Zasshi*. 2006;**97**(10):285-92.
33. Furue M, Uchi H, Moroi Y, Ogawa S, Nakahara T, Urabe K. Topical tacrolimus in the management of atopic dermatitis in Japan. *Dermatol Ther*. 2006;**19**(2):118-26.
34. Garside R, Stein K, Castelnuovo E, Pitt M, Ashcroft D, Dimmock P, et al. The effectiveness and cost-effectiveness of pimecrolimus and tacrolimus for atopic eczema: A systematic review and economic evaluation. *Health Technol Assess*. 2005;**9**(29):iii-122.
35. Goustas P, Cork M, Higson D. EumovateTM (clobetasone butyrate 0.05%) cream: A review of clinical efficacy and safety. *J Dermatolog Treat*. 2003;**14**(2):71-85.
36. Green C, Colquitt J, Kirby J, Davidson P. Topical corticosteroids for atopic eczema: Clinical and cost effectiveness of once-daily vs. more frequent use. *Br J Dermatol*. 2005;**152**(1):130-41.
37. Green C, Colquitt J, Kirby J, Davidson P, Payne E. Clinical and cost-effectiveness of once-daily versus more frequent use of same potency topical corticosteroids for atopic eczema: a systematic review and economic evaluation (Structured abstract)2004; (4):[1 p.]. Available from: http://onlinelibrary.wiley.com/o/cochrane/clhta/articles/HTA-32004000840/frame.html.
38. Halling-Overgaard A, Kezic S, Jakasa I, Engebretsen K, Maibach H, Thyssen J. Skin absorption through atopic dermatitis skin: a systematic review. *Br J Dermatol*. 2017;**177**(1):84-106.
39. Tacrolimus and pimecrolimus for atopic eczema (Structured abstract)2004; (4):[45 p.]. Available from: http://onlinelibrary.wiley.com/o/cochrane/clhta/articles/HTA-32004000795/frame.html.
40. Pimecrolimus (Elidel (R)) for atopic dermatitis (Structured abstract)2004; (4). Available from: http://onlinelibrary.wiley.com/o/cochrane/clhta/articles/HTA-32004000175/frame.html.
41. New products for atopic dermatitis - horizon scanning review (Structured abstract)2001; (4):[5 p.]. Available from: http://onlinelibrary.wiley.com/o/cochrane/clhta/articles/HTA-32002000841/frame.html.
42. Frequency of application of topical corticosteroids for atopic eczema (Structured abstract)2004; (4):[34 p.]. Available from: http://onlinelibrary.wiley.com/o/cochrane/clhta/articles/HTA-32004000794/frame.html.
43. Hannuksela M. [What is the best treatment for atopic dermatitis?]. *Duodecim*. 2000;**116**(21):2321-2.
44. Hebert A. Review of pimecrolimus cream 1% for the treatment of mild to moderate atopic dermatitis. *Clin Ther*. 2006;**28**(12):1972-82.
45. Hoare C, Li WPA, Williams H. Systematic review of treatments for atopic eczema (Structured abstract)2000; 4(37):[1-191 pp.]. Available from: http://onlinelibrary.wiley.com/o/cochrane/cldare/articles/DARE-12001008089/frame.html.
46. Hon KL, Chan BC, Leung PC. Chinese herbal medicine research in eczema treatment. *Chin Med*. 2011;**6 (no pagination)**(17).
47. Hulshof L, van't Land B, Sprikkelman A, Garssen J. Role of microbial modulation in management of atopic dermatitis in children. *Nutrients*. 2017;**9** (8):pii: E854.
48. Hussain Z, Sahudin S, Thu H, Shuid A, Bukhari S, Kumolosasi E. Recent Advances in Pharmacotherapeutic Paradigm of Mild to Recalcitrant Atopic Dermatitis. *Crit Rev Ther Drug Carrier Syst*. 2016;**33**(3):213-63.
49. Kaufman B, Alexis A. Safety and efficacy of topical calcineurin inhibitors and topical steroids in atopic dermatitis in skin of color: A systematic review. *Exp Dermatol* 2016;**25**:50.
50. Legendre L, Barnetche T, Juliette M, Meyer N, Paul C. Risk of lymphoma in atopic dermatitis: A systematic review and metaanalysis of epidemiologic studies. *J Am Acad Dermatol*. 2015;**1**:AB75.
51. Li A, Yin E, Antaya R. Topical corticosteroid phobia and fear in atopic dermatitis: A systematic review. *J Invest Dermatol*. 2017;**137 (5 Supplement 1)**:S26.
52. Li A, Yin E, Antaya R. Topical corticosteroid phobia in atopic dermatitis: A systematic review. *JAMA Dermatol*. 2017;**153**(10):1036-42.
53. Meffert H, Schuppler J. Methylprednisolone aceponate lotion for the treatment of acute eczema. [German]. *H+G Zeitschrift fur Hautkrankheiten*. 1999;**74**(2):89-94.
54. Mooney E, Rademaker M, Dailey R, Daniel B, Drummond C, Fischer G, et al. Adverse effects of topical corticosteroids in paediatric eczema: Australasian consensus statement. *Australas J Dermatol*. 2015;**56**(4):241-51.

For peer review only

55. Murashkin N, Materikin A, Ambarchyan E, Epishev R. Current views on the pathogenesis and principles of external treatment of atopic dermatitis in children. [Russian]. *Voprosy Sovremennoi Pediatrii - Current Pediatrics*. 2016;**15**(6):584-9.
56. Nankervis H, Delamere F, Williams H. Systematic review of treatments for eczema informs clinical practice and highlights research gaps. *J Invest Dermatol*. 2013;**133**:S176.
57. Nankervis H, Thomas K, Delamere F, Barbarot S, Rogers N, Williams H. Programme Grants for Applied Research. 2016.
58. Nankervis H, Thomas K, Delamere F, Barbarot S, Smith S, Rogers N, et al. What is the evidence base for atopic eczema treatments? A summary of published randomized controlled trials. *Br J Dermatol*. 2017;**176**(4):910-27.
59. Novak N. Allergen specific immunotherapy for atopic dermatitis. *Curr Opin Allergy Clin Immunol*. 2007;**7**(6):542-6.
60. Orlow S. Topical calcineurin inhibitors in pediatric atopic dermatitis: a critical analysis of current issues. *Paediatr Drugs*. 2007;**9**(5):289-99.
61. Pan M, Heinecke G, Bernardo S, Tsui C, Levitt J. Urea: A comprehensive review of the clinical literature. *Dermatol Online J*. 2013;**19**(11).
62. Park-Wyllie L, Mazzotta P, Pastuszak A, Moretti M, Beique L, Hunnisett L, et al. Birth defects after maternal exposure to corticosteroids: prospective cohort study and meta-analysis of epidemiological studies (Structured abstract)2000; 62(6):[385-92 pp.]. Available from: http://onlinelibrary.wiley.com/o/cochrane/cldare/articles/DARE-12001003037/frame.html.
63. Phipatanakul W. Efficacy and tolerability of pimecrolimus and tacrolimus in the treatment of atopic dermatitis: Meta-analysis of randomised controlled trials. Commentary. *Pediatrics*. 2006;**118**(SUPPL. 1):S16-S7.
64. Radovic T, Kostovic K, Ceovic R, Mokos Z. Topical calcineurin inhibitors and malignancy risk. *Int J Cancer Manag*. 2017;**10 (4) (no pagination)**(e6173).
65. Ricci G, Dondi A, Patrizi A. Role of topical calcineurin inhibitors on atopic dermatitis of children. *Curr Med Chem*. 2007;**14**(14):1579-91.
66. Ruzicka T, Assmann T, Homey B. Tacrolimus: The drug for the turn of the millennium? *Arch Dermatol*. 1999;**135**(5):574-80.
67. Sanchez J, Paez B, Macias A, Olmos C, De Falco A. Atopic dermatitis guideline. Position paper from the Latin American Society of allergy, asthma and immunology. *Revista Alergia Mexico*. 2014;**61**(3):178-211.
68. Schiffner R, Schiffner-Rohe J, Landthaler M, Stolz W. Treatment of atopic dermatitis and impact on quality of life: a review with emphasis on topical non-corticosteroids. *Pharmacoeconomics*. 2003;**21**(3):159-79.
69. Schmitt J, Apfelbacher C, Flohr C. Eczema. *BMJ Clin Evid*. 2011(pagination).
70. Schmitt J, Kobyletzki L, Svensson A, Apfelbacher C. Efficacy and tolerability of proactive treatment with topical corticosteroids and calcineurin inhibitors for atopic eczema: systematic review and meta-analysis of randomized controlled trials (Provisional abstract)2011; 164(2):[415-28 pp.]. Available from: http://onlinelibrary.wiley.com/o/cochrane/cldare/articles/DARE-12011001466/frame.html.
71. Sher L, Chang J, Patel I, Balkrishnan R, Fleischer A. Relieving the pruritus of atopic dermatitis: A meta-analysis. *Value in Health*. 2012;**15 (4)**:A249-A50.
72. Sher LG, Chang J, Patel I, Balkrishnan R, Fleischer Jr A. Relieving the pruritus of atopic dermatitis: A meta-analysis. *Acta Derm Venereol*. 2012;**92**(5):455-61.
73. Siegfried E, Jaworski J, Hebert A. Topical calcineurin inhibitors and lymphoma risk: evidence update with implications for daily practice. *Am J Clin Dermatol*. 2013;**14**(3):163-78.
74. Siegfried E, Jaworski J, Mina-Osorio P. A Systematic Scoping Literature Review of Publications Supporting Treatment Guidelines for Pediatric Atopic Dermatitis in Contrast to Clinical Practice Patterns. *Dermatol Ther (Heidelb)*. 2018;**8**(3):349-77.
75. Silverberg J. Atopic dermatitis: An evidence-based treatment update. *Am J Clin Dermatol*. 2014;**15**(3):149-64.
76. Simpson E. Atopic dermatitis: A review of topical treatment options. *Curr Med Res Opin*. 2010;**26**(3):633-40.
77. Spada F, Barnes T, Greive K. Comparative safety and efficacy of topical mometasone furoate with other topical corticosteroids. *Australas J Dermatol*. 2018;**59**(3):e168-e74.
78. Torii H. The Evaluation of Tacrolimus on Evidence-based Medicine (EBM). [Japanese]. *Skin Research*. 2003;**2**(SUPPL. 3):27-30.
79. Torley D, Futamura M, Williams H, Thomas K. What's new in atopic eczema? An analysis of systematic reviews published in 2010-11. *Clin Exp Dermatol*. 2013;**38**(5):449-56.

For peer review only

80. van Zuuren E, Fedorowicz Z, Arents B. Emollients and moisturisers for eczema: abridged Cochrane systematic review including GRADE assessments. *Br J Dermatol*. 2017.
81. Wat H, Dytoc M. Off-label uses of topical vitamin D in dermatology: A systematic review. *J Cutan Med Surg*. 2014;**18**(2):91-108.
82. Wellington K, Noble S. Pimecrolimus: A review of its use in atopic dermatitis. *Am J Clin Dermatol*. 2004;**5**(6):479-95.
83. Williams H. Established corticosteroid creams should be applied only once daily in patients with atopic eczema. *BMJ*. 2007;**334**(7606):1272.
84. Williams H, Grindlay D. What's new in atopic eczema? An analysis of the clinical significance of systematic reviews on atopic eczema published in 2006 and 2007. *Clin Exp Dermatol*. 2008;**33**(6):685-8.
85. Williams H, Grindlay D. What's new in atopic eczema? An analysis of systematic reviews published in 2007 and 2008. Part 2. Disease prevention and treatment. *Clin Exp Dermatol*. 2010;**35**(3):223-7.
86. Wollenberg A, Barbarot S, Bieber T, Christen-Zaech S, Deleuran M, Fink-Wagner A, et al. Consensus-based European guidelines for treatment of atopic eczema (atopic dermatitis) in adults and children: part I. *J Eur Acad Dermatol Venereol*. 2018;**32**(5):657-82.

For peer review only

Appendix 3: Prospero search

Prospero – searched 13/07/18 – search results from 'Eczema OR dermatitis'

Potentially relevant ongoing systematic reviews:

Prospero ID	Date registered	Title	Status	Anticipated completion date
CRD42018092929 [1]	16/04/2018	Cancer risk in patients with atopic dermatitis: protocol for a systematic review	Review ongoing	11 August 2018
CRD42015016525 [2]	11/02/2015	Effects of emollients in the management of atopic dermatitis in pediatric patients a systemic review and meta-analysis	Review ongoing	25 February 2016
CRD42015027873 [3]	04/11/2015	Interventions to improve quality of life in paediatric atopic dermatitis: a systematic review	Review ongoing	01 January 2016
CRD42017056060 [4]	01/02/2017	Systematic review and meta-analysis of randomized clinical trials (RCTs) comparing topical corticosteroids with moisturizers for children (< 2 years old) with atopic dermatitis	Review ongoing	01 April 2017

References

1. Chan A-W. Cancer risk in patients with atopic dermatitis: protocol for a systematic review [CRD42018092929] 2018 (accessed: 13/07/18). Available from: https://www.crd.york.ac.uk/prospero/display_record.php?RecordID=92929.
2. Tan Q, Tan C, Peng W, Shi Y, Xia L. Effects of emollients in the management of atopic dermatitis in pediatric patients a systemic review and meta-analysis [CRD42015016525] 2015 (accessed: 13/07/18). Available from: http://www.crd.york.ac.uk/PROSPERO/display_record.php?ID=CRD42015016525.
3. Yu A, Hong J, Lee M, Hong B. Interventions to improve quality of life in paediatric atopic dermatitis: a systematic review [CRD42015027873] 2015 (accessed: 13/07/18). Available from: http://www.crd.york.ac.uk/PROSPERO/display_record.php?ID=CRD42015027873.
4. Fishbein A, Kaat A, Smith P. Systematic review and meta-analysis of randomized clinical trials (RCTs) comparing topical corticosteroids with moisturizers for children (< 2 years old) with atopic dermatitis [CRD42017056060] 2017 (accessed: 13/07/18). Available from: http://www.crd.york.ac.uk/PROSPERO/display_record.php?ID=CRD42017056060.

For peer review only

Appendix 4: AMSTAR 2 ratings [1]:

Review ID	1 PICO	2 Protocol*	3 Study designs	4 Search strategy*	5 Duplicate screening	6 Duplicate data extraction	7 Excluded studies*	8 Included studies	9 Risk of bias assessed*	10 Funding of studies	11 Appropriate meta-analysis*	12 Risk of bias in meta-analysis	13 Risk of bias in discussion *	14 Hetero-geneity	15 Publication bias in meta-analysis*	16 Reviewers' conflict of interest	Overall rating
Ashcroft 2005 [2]	Yes	No	No	Yes	Yes	Yes	No	Partial yes	Partial yes	No	Yes	No	No	No	No	Yes	Critically low
Ashcroft 2007 [3]	Yes	Partial yes	No	Yes	Yes	Yes	Yes	Partial yes	Yes	No	N/A	N/A	Yes	Yes	N/A	Yes	Moderate
Barnes 2015 [4]	No	No	No	No	No	No	No	No	No	No	N/A	N/A	No	No	N/A	Yes	Critically low
Braham 2010 [5]	No	No	Yes	No	No	No	No	Partial yes	No	No	N/A	N/A	No	No	N/A	Yes	Critically low
Broeders 2016 [6]	Yes	No	No	Partial yes	No	No	No	Partial yes	Partial yes	Yes	No	No	No	No	Yes	Yes	Critically low
Callen 2007 [7]	Yes	No	No	No	No	No	No	No	No	No	N/A	N/A	No	No	N/A	Yes	Critically low
Chen 2010 [8]	Yes	No	No	Partial yes	No	No	No	Partial yes	Yes	Yes	N/A	N/A	No	No	N/A	Yes	Critically low
Cury Martins 2015 [9]	Yes	Yes	No	Yes	Yes	Yes	Yes	Partial yes	Yes	No	Yes	Yes	Yes	No	Yes	Yes	Moderate
De Tiedra 1997 [10]	No	No	No	No	No	No	No	Partial yes	No	No	N/A	N/A	No	No	N/A	No	Critically low
Devillers 2006 [11]	No	No	Yes	No	No	No	No	Partial yes	No	No	N/A	N/A	No	No	N/A	Yes	Critically low
Dong 2017 [12]	Yes	No	No	No	Yes	Yes	No	No	Yes	No	No	No	No	No	No	No	Critically low
Eichenfield 2014 [13]	No	No	Yes	No	No	No	No	No	No	No	N/A	N/A	No	No	N/A	Yes	Critically low
Feldman 2005 [14]	No	No	Yes	No	No	No	No	Partial yes	No	No	N/A	N/A	No	No	N/A	No	Critically low
Frangos 2008 [15]	No	No	No	No	No	No	No	No	No	No	N/A	N/A	No	No	N/A	No	Critically low
Froeschl 2007 [16]	No	No	No	No	No	No	Yes	Partial yes	No	Yes	N/A	N/A	No	No	N/A	No	Critically low
Gonzalez-Lopez 2017 [17]	Yes	No	No	Yes	Yes	Yes	No	Yes	Yes	Yes	Yes	Yes	Yes	Yes	Yes	Yes	Critically low

Green 2004 [18]	Yes	Partial Yes	No	No	Partial yes	Yes	Partial yes	Yes	Partial yes	Yes	N/A	N/A	Yes	Yes	N/A	Yes	Low
Gu 2013 [19]	Yes	Yes	No	Yes	Yes	Yes	Yes	Yes	Yes	Yes	Yes	Yes	Yes	Yes	Yes	Yes	High
Gu 2014 [20]	Yes	No	No	No	No	No	No	No	Yes	No	Yes	No	Yes	Yes	No	Yes	Critically low
Hajar 2015 [21]	Yes	Yes	Yes	No	Yes	Yes	No	No	Partial yes	No	N/A	N/A	No	No	N/A	Yes	Critically low
Hoare 2000 [22]	Yes	No	Yes	Yes	No	No	Yes	Partial yes	Partial yes	No	N/A	N/A	Yes	No	N/A	Yes	Low
Iskedjian 2004 [23]	Yes	No	No	Partial yes	Yes	Yes	No	Partial yes	Partial yes	No	N/A	N/A	No	No	N/A	No	Critically low
Juhasz 2017 [24]	No	No	No	No	No	No	No	No	No	N/A	N/A	N/A	No	No	N/A	Yes	Critically low
Legendre 2015 [25]	Yes	No	No	No	Yes	Yes	No	Partial yes	Yes	No	Yes	No	No	Yes	Yes	Yes	Critically low
Li 2007 [26]	Yes	No	No	Partial yes	No	Yes	No	No	Partial yes	No	N/A	N/A	No	No	N/A	No	Critically low
Nankervis 2016 [27]	Yes	Partial yes	Yes	Partial yes	No	Yes	No	Partial yes	Partial yes	Yes	N/A	N/A	Yes	No	N/A	Yes	Low
Penaloza Hidalgo 2004 [28]	Yes	Partial yes	No	Yes	No	No	Yes	Partial yes	Partial yes	Yes	N/A	N/A	No	Yes	N/A	Yes	Low
Schmitt 2011 [29]	Yes	Partial yes	No	Partial yes	Yes	Yes	No	Yes	Yes	No	N/A	N/A	No	Yes	N/A	Yes	Critically low
Sidbury 2011 [30]	No	No	Yes	No	No	No	No	No	No	No	N/A	N/A	No	No	N/A	Yes	Critically low
Siegfried 2016 [31]	No	No	No	No	No	No	No	No	No	No	N/A	N/A	No	No	N/A	Yes	Critically low
Singh 2012 [32]	No	No	No	No	No	No	Partial yes	No	Partial yes	No	N/A	N/A	Yes	No	N/A	Yes	Critically low
Svensson 2011 [33]	Yes	No	No	Partial yes	Yes	No	No	Partial yes	Partial yes	No	Yes	No	No	Yes	No	Yes	Critically low
Tang 2014 [34]	Yes	No	No	Partial yes	No	No	No	No	No	No	N/A	N/A	No	No	N/A	Yes	Critically low
van Zuuren 2017 [35]	Yes	Yes	No	Partial yes	Yes	Yes	Yes	Yes	Yes	Yes	Yes	Yes	Yes	Yes	Yes	Yes	Moderate
Wood Heickman 2017 [36]	Yes	No	Yes	No	Yes	No	No	No	No	No	Yes	No	No	Yes	No	Yes	Critically low
Yan 2008 [37]	Yes	No	No	Partial yes	No	No	No	No	Partial yes	Yes	Yes	No	No	No	No	No	Critically low

Footnotes: AMSTAR 2 domains

1 Did the research questions and inclusion criteria for the review include the components of PICO?

**2 Did the report of the review contain an explicit statement that the review methods were established prior to the conduct of the review and did the report justify any significant deviations from the protocol?*

3 Did the review authors explain their selection of the study designs for inclusion in the review?

**4 Did the review authors use a comprehensive literature search strategy?*

5 Did the review authors perform study selection in duplicate?

6 Did the review authors perform data extraction in duplicate?

**7 Did the review authors provide a list of excluded studies and justify the exclusions?*

8 Did the review authors describe the included studies in adequate detail?

**9 Did the review authors use a satisfactory technique for assessing the risk of bias (RoB) in individual studies that were included in the review?*

10 Did the review authors report on the sources of funding for the studies included in the review?

**11 If meta-analysis was performed, did the review authors use appropriate methods for statistical combination of results?*

12 If meta-analysis was performed, did the review authors assess the potential impact of RoB in individual studies on the results of the meta-analysis or other evidence synthesis?

**13 Did the review authors account for RoB in primary studies when interpreting/discussing the results of the review?*

14 Did the review authors provide a satisfactory explanation for, and discussion of, any heterogeneity observed in the results of the review?

**15 If they performed quantitative synthesis did the review authors carry out an adequate investigation of publication bias (small study bias) and discuss its likely impact on the results of the review?*

16 Did the review authors report any potential sources of conflict of interest, including any funding they received for conducting the review?

NB domains marked * in the table and footnotes are critical domains.

For peer review only

References

1. Shea BJ, Reeves BC, Wells G, Thuku M, Hamel C, Moran J, et al. AMSTAR 2: a critical appraisal tool for systematic reviews that include randomised or non-randomised studies of healthcare interventions, or both. *BMJ* 2017;**358**:j4008.
2. Ashcroft D, Dimmock P, Garside R, Stein K, Williams H. Efficacy and tolerability of topical pimecrolimus and tacrolimus in the treatment of atopic dermatitis: Meta-analysis of randomised controlled trials. *BMJ* 2005;**330**(7490):516-22.
3. Ashcroft DM, Chen L-C, Garside R, Stein K, Williams HC. Topical pimecrolimus for eczema Cochrane Database Syst Rev [Internet]. 2007; (4). Available from: http://onlinelibrary.wiley.com/doi/10.1002/14651858.CD005500.pub2/abstract.
4. Barnes L, Kaya G, Rollason V. Topical Corticosteroid-Induced Skin Atrophy: A Comprehensive Review. *Drug Saf*. 2015;**38**(5):493-509.
5. Braham S, Pugashetti R, Koo J, Maibach H. Occlusive therapy in atopic dermatitis: overview. *J Dermatolog Treat*. 2010;**21**(2):62-72.
6. Broeders J, Ahmed Ali U, Fischer G. Systematic review and meta-analysis of randomized clinical trials (RCTs) comparing topical calcineurin inhibitors with topical corticosteroids for atopic dermatitis: A 15-year experience. *J Am Acad Dermatol*. 2016;**75**(2):410-9.e3.
7. Callen J, Chamlin S, Eichenfield L, Ellis C, Girardi M, Goldfarb M, et al. A systematic review of the safety of topical therapies for atopic dermatitis. *Br J Dermatol*. 2007;**156**(2):203-21.
8. Chen S, Yan J, Wang F. Two topical calcineurin inhibitors for the treatment of atopic dermatitis in pediatric patients: A meta-analysis of randomized clinical trials. *J Dermatolog Treat*. 2010;**21**(3):144-56.
9. Cury Martins J, Martins C, Aoki V, Gois AF, Ishii HA, da SEM. Topical tacrolimus for atopic dermatitis. Cochrane Database Syst Rev [Internet]. 2015; (7). Available from: http://onlinelibrary.wiley.com/doi/10.1002/14651858.CD009864.pub2/abstract.
10. de Tiedra A, Mercadal J, Lozano R. Prednicarbate versus fluocortin for inflammatory dermatoses: A cost-effectiveness study. *PharmacoEconomics*. 1997;**12**(2 Pt 1):193-208.
11. Devillers A, Oranje A. Efficacy and safety of 'wet-wrap' dressings as an intervention treatment in children with severe and/or refractory atopic dermatitis: a critical review of the literature. *Br J Dermatol*. 2006;**154**(4):579-85.
12. Dong Y, Zeng W, Li W, Ma H, Zheng W. Efficacy and safety of topical tacrolimus for childhood atopic dermatitis; a meta-analysis. [Chinese]. *J Clin Dermatol*. 2017;**46**(4):239-42.
13. Eichenfield L, Tom W, Berger T, Krol A, Paller A, Schwarzenberger K, et al. Guidelines of care for the management of atopic dermatitis: Section 2. Management and treatment of atopic dermatitis with topical therapies. *J Am Acad Dermatol*. 2014;**71**(1):116-32.
14. Feldman S. Relative efficacy and interchangeability ofvarious clobetasol propionate vehicles in the management of steroid-responsive dermatoses. *Curr Ther Res Clin Exp*. 2005;**66**(3):154-71.
15. Frangos J, Kimball A. Clobetasol propionate emollient formulation foam in the treatment of corticosteroid-responsive dermatoses. *Expert Opin Pharmacother*. 2008;**9**(11):2001-7.
16. Froeschl B, Arts D, Leopold C. Corticosteroid therapy in the treatment of pediatric patients with atopic dermatitis (Structured abstract). Health Technol Assess [Internet]. 2007; (4). Available from: http://onlinelibrary.wiley.com/o/cochrane/clhta/articles/HTA-32008100208/frame.html.
17. Gonzalez-Lopez G, Ceballos-Rodriguez R, Gonzalez-Lopez J, Feito Rodriguez M, Herranz-Pinto P. Efficacy and safety of wet wrap therapy for patients with atopic dermatitis: a systematic review and meta-analysis. *Br J Dermatol*. 2017;**177**(3):688-95.
18. Green C, Colquitt J, Kirby J, Davidson P, Payne E. Clinical and cost-effectiveness of once-daily versus more frequent use of same potency topical corticosteroids for atopic eczema: a systematic review and economic evaluation. *Health Technol Assess (Winchester, England)*. 2004;**8**(47):iii,iv, 1-120.

For peer review only - http://bmjopen.bmj.com/site/about/guidelines.xhtml

19. Gu S, Yang AW, Xue CC, Li CG, Pang C, Zhang W, et al. Chinese herbal medicine for atopic eczema. Cochrane Database Syst Rev [Internet]. 2013; (9). Available from: http://onlinelibrary.wiley.com/doi/10.1002/14651858.CD008642.pub2/abstract.
20. Gu S, Yang A, Li C, Lu C, Xue C. Topical application of Chinese herbal medicine for atopic eczema: A systematic review with a meta-analysis. *Dermatology*. 2014;**228**(4):294-302.
21. Hajar T, Leshem Y, Hanifin J, Nedorost S, Lio P, Paller A, et al. A systematic review of topical corticosteroid withdrawal ("steroid addiction") in patients with atopic dermatitis and other dermatoses. *J Am Acad Dermatol*. 2015;**72**(3):541-9.e2.
22. Hoare C, Li Wan Po A, Williams H. Systematic review of treatments for atopic eczema. *Health Technol Assess*. 2000;**4**(37):1-191.
23. Iskedjian M, Piwko C, Shear N, Langley R, Einarson T. Topical calcineurin inhibitors in the treatment of atopic dermatitis: A meta-analysis of current evidence. *Am J Clin Dermatol*. 2004;**5**(4):267-79.
24. Juhász ML, Curley RA, Rasmussen A, Malakouti M, Silverberg N, Jacob SE. Systematic Review of the Topical Steroid Addiction and Topical Steroid Withdrawal Phenomenon in Children Diagnosed With Atopic Dermatitis and Treated With Topical Corticosteroids. *J Dermatol Nurses Assoc*. 2017;**9**(5):233-40.
25. Legendre L, Barnetche T, Mazereeuw-Hautier J, Meyer N, Murrell D, Paul C. Risk of lymphoma in patients with atopic dermatitis and the role of topical treatment: A systematic review and meta-analysis. *J Am Acad Dermatol*. 2015;**72**(6):992-1002.
26. Li R, Zhu H, Fan L, Ni S, Feng C, Wu Z. Efficacy and tolerability of topical tacrolimus in the treatment of atopic dermatitis: A systematic review of randomized controlled trials. [Chinese]. *J Clin Dermatol*. 2007;**36**(12):757-60.
27. Nankervis H, Thomas K, Delamere F, Barbarot S, Rogers N, Williams H. Scoping systematic review of treatments for eczema2016 2016/05/None.
28. Penaloza Hidalgo B, Knight T, Burls A. A systematic review of effectiveness and cost effectiveness of tacrolimus ointment for topical treatment of atopic dermatitis in adults and children Health Technol Assess [Internet]. 2004; (4):[81 p.].
29. Schmitt J, Von Kobyletzki L, Svensson A, Apfelbacher C. Efficacy and tolerability of proactive treatment with topical corticosteroids and calcineurin inhibitors for atopic eczema: Systematic review and meta-analysis of randomized controlled trials. *Br J Dermatol*. 2011;**164**(2):415-28.
30. Sidbury R, Tom W, Bergman J, Cooper K, Silverman R, Berger T, et al. Guidelines of care for the management of atopic dermatitis: Section 4. Prevention of disease flares and use of adjunctive therapies and approaches. *J Am Acad Dermatol*. 2014;**71**(6):1218-33.
31. Siegfried E, Jaworski J, Kaiser J, Hebert A. Systematic review of published trials: Long-term safety of topical corticosteroids and topical calcineurin inhibitors in pediatric patients with atopic dermatitis. *BMC Pediatr*. 2016;**16** (75).
32. Singh S, Mann B. Clinical utility of clocortolone pivalate for the treatment of corticosteroid-responsive skin disorders: A systematic review. *Clin Cosmet Investig Dermatol*. 2012;**5**:61-8.
33. Svensson A, Chambers C, Gånemo A, Mitchell S. A systematic review of tacrolimus ointment compared with corticosteroids in the treatment of atopic dermatitis. *Curr Med Res Opin*. 2011;**27**(7):1395-406.
34. Tang T, Bieber T, Williams H. Are the concepts of induction of remission and treatment of subclinical inflammation in atopic dermatitis clinically useful? *J Allergy Clin Immunol*. 2014;**133**(6):1615-25.e1.
35. van Zuuren EJ, Fedorowicz Z, Christensen R, Lavrijsen AP, Arents BW. Emollients and moisturisers for eczema. Cochrane Database Syst Rev [Internet]. 2017; (2). Available from: http://onlinelibrary.wiley.com/doi/10.1002/14651858.CD012119.pub2/abstract.
36. Wood Heickman L, Davallow Ghajar L, Conaway M, Rogol A. Evaluation of Hypothalamic-Pituitary-Adrenal Axis Suppression following Cutaneous Use of Topical Corticosteroids in Children: A Meta-Analysis. *Horm Res Paediatr*. 2018;**89**(6):389-96.
37. Yan J, Chen S, Wang X, Zhou W, Wang F. Meta-analysis of tacrolimus ointment for atopic dermatitis in pediatric patients. *Pediatr Dermatol*. 2008;**25**(1):117-20.

For peer review only

Appendix 5: Characteristics of the included systematic reviews

First author and year of publication	Type of review	Language of publication	Funding source	Conflicts of interest	RCT comparisons	Observational data included	AMSTAR-2 rating
Ashcroft 2005 [1]	Non-Cochrane	English	NHS health technology assessment programme.	None known	TCS versus TCI	No	Critically low
Ashcroft 2007 [2]	Cochrane	English	School of Pharmacy and Pharmaceutical Sciences, University of Manchester, UK.	None known	TCS versus TCI	Yes – TCI compared with TCS	Moderate
Barnes 2015 [3]	Non-Cochrane	English	No funding	None known	TCS versus vehicle<br>TCS versus TCI<br>TCS versus TCS	Yes (single arm TCS studies)	Critically low
Braham 2010 [4]	Non-Cochrane	English	No funding	One author was a speaker for a number of pharmaceutical companies	Occlusion therapy versus non-occlusion therapy	Yes (occlusive therapy, no comparison)	Critically low
Broeders 2016 [5]	Non-Cochrane	English	No funding	None known	TCS versus TCI	No	Critically low
Callen 2007 [6]	Non-Cochrane	English	Funding from EBMed. One author received funding from Novartis Corporation for the project. They declared that "Novartis Corporation played no role in the design and conduct of the study or in data collection, data management, data analysis, interpretation of the data, manuscript preparation, manuscript review or manuscript approval"	Most authors had consultancy fees and/or research support from pharmaceutical companies	TCS versus vehicle<br>TCS versus TCS	Yes (single arm TCS studies or comparing various TCS potencies)	Critically low
Chen 2010 [7]	Non-Cochrane	English	Not stated	None known	TCS versus TCI	No	Critically low
Cury Martins 2015 [8]	Cochrane	English	NIHR	None known	TCS versus TCI	Yes – TCI compared to TCS	Moderate
De Tiedra 1997 [9]	Non-Cochrane	English	Supported by Laboratorios Novag, S.A, Grupo Ferrer.	Not clear	TCS versus TCS	Yes – in most cases they only report data from one arm of an RCT	Critically low
Devillers 2006 [10]	Non-Cochrane	English	Not stated	None known	Occlusive therapy versus non-occlusive therapy	Yes – occlusive therapy (no comparison)	Critically low
Dong 2017 [11]	Non-Cochrane	Chinese	Not stated	Not clear	TCS versus TCI	No	Critically low
Eichenfield 2014 [12]	Non-Cochrane	English	No funding	Most authors served as consultants, speakers, members of the advisory	None	Yes (comparing different TCS potencies)	Critically low

For peer review only

				board and/or investigators for pharmaceutical companies.			
Feldman 2005 [13]	Non-Cochrane	English	Grant from Galderma Laboratories, LP, Fort Worth, Texas.	Not clear	TCS versus vehicle	No	Critically low
Frangos 2008 [14]	Non-Cochrane	English	Not stated	One author is an investigator for Steifel and was an investigator on two of the studies reviewed.	TCS versus vehicle	Yes (single arm studies)	Critically low
Froschl 2007 [15]	GMS HTA report	German (executive summary in English)	Not stated	Not stated	TCS versus placebo/vehicle<br>TCS versus TCS<br>TCS versus TCI	No	Critically low
Gonzalez-Lopez 2017 [16]	Non-Cochrane	English	No funding	None known	Occlusive therapy versus non-occlusive therapy	No	Critically low
Green 2004 [17]	HTA report	English	Funded by the HTA Programme on behalf of NICE	None known	Once daily versus twice daily TCS use	No	Low
Gu 2013 [18]	Cochrane	English	RMIT University<br>Nottingham University, UK.<br>NIHR	One author was a principal investigator on one included study (but this study was not relevant for this overview)	Chinese herbal medicine versus TCS	No	High
Gu 2014 [19]	Non-Cochrane	English	Not stated	None known	Chinese herbal medicine versus TCS	No	Critically low
Hajar 2015 [20]	Non-Cochrane	English	No funding	None known	No RCTs found	Yes (case series or case reports on steroid withdrawal)	Critically low
Hoare 2000 [21]	NIHR HTA report	English	HTA programme	One author received payment from Novartis for lectures on the epidemiology of atopic eczema in 1999.<br>Another author has acted as occasional lecturer or consultant for pharmaceutical companies.	TCS versus TCS<br>TCS versus vehicle	No	Low
Iskedjian 2004 [22]	Non-Cochrane	English	Funded by Fujisawa Canada Inc.	Not clear	TCS versus TCI<br>TCS versus placebo	No	Critically low
Juhasz 2017 [23]	Non-Cochrane	English	Not stated	One author had primary contact with the 2nd case and has a blog on the subject matter in this systematic review	No RCTs found	Yes (case reports on steroid withdrawal)	Critically low
Legendre 2015 [24]	Non-Cochrane	English	No funding	One author is a consultant and investigator for two pharmaceutical companies. One author is a speaker	Only searched for cohort or case control studies	Yes (comparing TCS and TCI)	Critically low

For peer review only

				and/or on the advisory board for five pharmaceutical companies.			
Li 2007 [25]	Non-Cochrane	Chinese	Not stated	Not stated	TCS versus TCI	No	Critically low
Nankervis 2016 [26]	NIHR HTA report	English	NIHR	One author reports grants and fees from a number of pharmaceutical companies.	TCS versus placebo/vehicle<br>Proactive treatment versus vehicle<br>TCS versus TCI<br>TCS versus TCS<br>Once a day versus twice a day use of TCS<br>Occlusive therapy versus non-occlusive therapy<br>TCS versus emollients	No	Low
Penzaloza Hidalgo 2004 [27]	West Midlands HTA report	English	Not stated	None known	TCS versus TCI	No	Low
Schmitt 2011 [28]	Non-Cochrane	English	No funding	One author has served as paid lecturer for a pharmaceutical company.	Proactive treatment versus vehicle	No	Critically low
Sidbury 2014 [29]	Non-Cochrane	English	Not stated	Some authors have served as investigators, consultants, speakers, and on advisory boards for pharmaceutical companies.	Proactive treatment versus vehicle	No	Critically low
Siegfried 2016 [30]	Non-Cochrane	English	Financial support for writing by Valent Pharmaceutical North America LLC. They declared that “Valeant Pharmaceuticals had no role in the design of the literature searches, or analysis and presentation of results.”	Authors have either participated in paid contract research, received travel expenses for presentations, consulting fees, speakers, on advisory boards, or on data safety monitoring boards with pharmaceutical companies.	TCS versus TCS<br>TCS versus TCI<br>TCS versus vehicle	No	Critically low
Singh 2012 [31]	Non-Cochrane	English	Not stated	None known	TCS versus TCS<br>TCI versus TCS<br>TCS versus placebo/vehicle	Yes (single arm TCS study)	Critically low

For peer review only

Svensson 2011 [32]	Non-Cochrane	English	Funded by Astellas Pharma Europe Ltd.	One author was a paid employee of Astellas Pharma Europe Ltd and one author undertook paid consultancy work for Astellas Pharma Europe Ltd.	TCI versus TCS	No	Critically low
Tang 2014 [33]	Non-Cochrane	English	Not stated	One author has received lecture fees from Astellas.	Proactive treatment versus vehicle	No	Critically low
van Zuuren 2017 [34]	Cochrane	English	Oak Foundation, Denmark NIHR	None known	TCS versus emollient	No	Moderate
Wood Heickman 2018 [35]	Non-Cochrane	English	No grants, honoraria or royalties were received supporting the writing of the paper.	One author was a consultant with Perrigo, Inc. with regard to topical corticosteroid treatment. All authors have no financial or other potential conflicts of interest.	Two RCTs included but analysed as observational data	Yes – single arm cohort studies	Critically low
Yan 2008 [36]	Non-Cochrane	English	Not stated	Not stated	TCI versus TCS	No	Critically low

Key: TCI=topical calcinueirin inhibitor; TCS=topical corticosteroids; RCT=Randomised Controlled Trial; NIHR= National Institute for Health Research

References

1. Ashcroft D, Dimmock P, Garside R, Stein K, Williams H. Efficacy and tolerability of topical pimecrolimus and tacrolimus in the treatment of atopic dermatitis: Meta-analysis of randomised controlled trials. *BMJ* 2005;**330**(7490):516-22.

2. Ashcroft DM, Chen L-C, Garside R, Stein K, Williams HC. Topical pimecrolimus for eczema Cochrane Database Syst Rev [Internet]. 2007; (4). Available from: http://onlinelibrary.wiley.com/doi/10.1002/14651858.CD005500.pub2/abstract.

3. Barnes L, Kaya G, Rollason V. Topical Corticosteroid-Induced Skin Atrophy: A Comprehensive Review. *Drug Saf*. 2015;**38**(5):493-509.

4. Braham S, Pugashetti R, Koo J, Maibach H. Occlusive therapy in atopic dermatitis: overview. *J Dermatolog Treat*. 2010;**21**(2):62-72.

5. Broeders J, Ahmed Ali U, Fischer G. Systematic review and meta-analysis of randomized clinical trials (RCTs) comparing topical calcineurin inhibitors with topical corticosteroids for atopic dermatitis: A 15-year experience. *J Am Acad Dermatol*. 2016;**75**(2):410-9.e3.

6. Callen J, Chamlin S, Eichenfield L, Ellis C, Girardi M, Goldfarb M, et al. A systematic review of the safety of topical therapies for atopic dermatitis. *Br J Dermatol*. 2007;**156**(2):203-21.

For peer review only

7. Chen S, Yan J, Wang F. Two topical calcineurin inhibitors for the treatment of atopic dermatitis in pediatric patients: A meta-analysis of randomized clinical trials. *J Dermatolog Treat*. 2010;**21**(3):144-56.
8. Cury Martins J, Martins C, Aoki V, Gois AF, Ishii HA, da SEM. Topical tacrolimus for atopic dermatitis. Cochrane Database Syst Rev [Internet]. 2015; (7). Available from: http://onlinelibrary.wiley.com/doi/10.1002/14651858.CD009864.pub2/abstract.
9. de Tiedra A, Mercadal J, Lozano R. Prednicarbate versus fluocortin for inflammatory dermatoses: A cost-effectiveness study. *PharmacoEconomics*. 1997;**12**(2 Pt 1):193-208.
10. Devillers A, Oranje A. Efficacy and safety of 'wet-wrap' dressings as an intervention treatment in children with severe and/or refractory atopic dermatitis: a critical review of the literature. *Br J Dermatol*. 2006;**154**(4):579-85.
11. Dong Y, Zeng W, Li W, Ma H, Zheng W. Efficacy and safety of topical tacrolimus for childhood atopic dermatitis; a meta-analysis. [Chinese]. *J Clin Dermatol*. 2017;**46**(4):239-42.
12. Eichenfield L, Tom W, Berger T, Krol A, Paller A, Schwarzenberger K, et al. Guidelines of care for the management of atopic dermatitis: Section 2. Management and treatment of atopic dermatitis with topical therapies. *J Am Acad Dermatol*. 2014;**71**(1):116-32.
13. Feldman S. Relative efficacy and interchangeability ofvarious clobetasol propionate vehicles in the management of steroid-responsive dermatoses. *Curr Ther Res Clin Exp*. 2005;**66**(3):154-71.
14. Frangos J, Kimball A. Clobetasol propionate emollient formulation foam in the treatment of corticosteroid-responsive dermatoses. *Expert Opin Pharmacother*. 2008;**9**(11):2001-7.
15. Froeschl B, Arts D, Leopold C. Corticosteroid therapy in the treatment of pediatric patients with atopic dermatitis (Structured abstract). Health Technol Assess [Internet]. 2007; (4). Available from: http://onlinelibrary.wiley.com/o/cochrane/clhta/articles/HTA-32008100208/frame.html.
16. Gonzalez-Lopez G, Ceballos-Rodriguez R, Gonzalez-Lopez J, Feito Rodriguez M, Herranz-Pinto P. Efficacy and safety of wet wrap therapy for patients with atopic dermatitis: a systematic review and meta-analysis. *Br J Dermatol*. 2017;**177**(3):688-95.
17. Green C, Colquitt J, Kirby J, Davidson P, Payne E. Clinical and cost-effectiveness of once-daily versus more frequent use of same potency topical corticosteroids for atopic eczema: a systematic review and economic evaluation. *Health Technol Assess (Winchester, England)*. 2004;**8**(47):iii,iv, 1-120.
18. Gu S, Yang A, Li C, Lu C, Xue C. Topical application of Chinese herbal medicine for atopic eczema: A systematic review with a meta-analysis. *Dermatology*. 2014;**228**(4):294-302.
19. Gu S, Yang AW, Xue CC, Li CG, Pang C, Zhang W, et al. Chinese herbal medicine for atopic eczema. Cochrane Database Syst Rev [Internet]. 2013; (9). Available from: http://onlinelibrary.wiley.com/doi/10.1002/14651858.CD008642.pub2/abstract.
20. Hajar T, Leshem Y, Hanifin J, Nedorost S, Lio P, Paller A, et al. A systematic review of topical corticosteroid withdrawal ("steroid addiction") in patients with atopic dermatitis and other dermatoses. *J Am Acad Dermatol*. 2015;**72**(3):541-9.e2.
21. Hoare C, Li Wan Po A, Williams H. Systematic review of treatments for atopic eczema. *Health Technol Assess*. 2000;**4**(37):1-191.
22. Iskedjian M, Piwko C, Shear N, Langley R, Einarson T. Topical calcineurin inhibitors in the treatment of atopic dermatitis: A meta-analysis of current evidence. *Am J Clin Dermatol*. 2004;**5**(4):267-79.

For peer review only

23. Juhász ML, Curley RA, Rasmussen A, Malakouti M, Silverberg N, Jacob SE. Systematic Review of the Topical Steroid Addiction and Topical Steroid Withdrawal Phenomenon in Children Diagnosed With Atopic Dermatitis and Treated With Topical Corticosteroids. *J Dermatol Nurses Assoc*. 2017;**9**(5):233-40.
24. Legendre L, Barnetche T, Mazereeuw-Hautier J, Meyer N, Murrell D, Paul C. Risk of lymphoma in patients with atopic dermatitis and the role of topical treatment: A systematic review and meta-analysis. *J Am Acad Dermatol*. 2015;**72**(6):992-1002.
25. Li R, Zhu H, Fan L, Ni S, Feng C, Wu Z. Efficacy and tolerability of topical tacrolimus in the treatment of atopic dermatitis: A systematic review of randomized controlled trials. [Chinese]. *J Clin Dermatol*. 2007;**36**(12):757-60.
26. Nankervis H, Thomas K, Delamere F, Barbarot S, Rogers N, Williams H. Scoping systematic review of treatments for eczema2016 2016/05/None.
27. Penaloza Hidalgo B, Knight T, Burls A. A systematic review of effectiveness and cost effectiveness of tacrolimus ointment for topical treatment of atopic dermatitis in adults and children Health Technol Assess [Internet]. 2004; (4):[81 p.].
28. Schmitt J, Von Kobyletzki L, Svensson A, Apfelbacher C. Efficacy and tolerability of proactive treatment with topical corticosteroids and calcineurin inhibitors for atopic eczema: Systematic review and meta-analysis of randomized controlled trials. *Br J Dermatol*. 2011;**164**(2):415-28.
29. Sidbury R, Tom W, Bergman J, Cooper K, Silverman R, Berger T, et al. Guidelines of care for the management of atopic dermatitis: Section 4. Prevention of disease flares and use of adjunctive therapies and approaches. *J Am Acad Dermatol*. 2014;**71**(6):1218-33.
30. Siegfried E, Jaworski J, Kaiser J, Hebert A. Systematic review of published trials: Long-term safety of topical corticosteroids and topical calcineurin inhibitors in pediatric patients with atopic dermatitis. *BMC Pediatr*. 2016;**16** (75).
31. Singh S, Mann B. Clinical utility of clocortolone pivalate for the treatment of corticosteroid-responsive skin disorders: A systematic review. *Clin Cosmet Investig Dermatol*. 2012;**5**:61-8.
32. Svensson A, Chambers C, Gånemo A, Mitchell S. A systematic review of tacrolimus ointment compared with corticosteroids in the treatment of atopic dermatitis. *Curr Med Res Opin*. 2011;**27**(7):1395-406.
33. Tang T, Bieber T, Williams H. Are the concepts of induction of remission and treatment of subclinical inflammation in atopic dermatitis clinically useful? *J Allergy Clin Immunol*. 2014;**133**(6):1615-25.e1.
34. van Zuuren EJ, Fedorowicz Z, Christensen R, Lavrijsen AP, Arents BW. Emollients and moisturisers for eczema. Cochrane Database Syst Rev [Internet]. 2017; (2). Available from: http://onlinelibrary.wiley.com/doi/10.1002/14651858.CD012119.pub2/abstract.
35. Wood Heickman L, Davallow Ghajar L, Conaway M, Rogol A. Evaluation of Hypothalamic-Pituitary-Adrenal Axis Suppression following Cutaneous Use of Topical Corticosteroids in Children: A Meta-Analysis. *Horm Res Paediatr*. 2018;**89**(6):389-96.
36. Yan J, Chen S, Wang X, Zhou W, Wang F. Meta-analysis of tacrolimus ointment for atopic dermatitis in pediatric patients. *Pediatr Dermatol*. 2008;**25**(1):117-20.

For peer review only

Appendix table 6: Characteristics and safety data for each included study

How safe are topical corticosteroids compared to emollient or vehicle?

Study ID *(Systematic review*)*	Study design and study duration *(Quality assessment)*	Intervention and comparator	Participants	Cutaneous adverse events	Systemic adverse events	Unspecified adverse events
**Very potent topical corticosteroids**						
Breneman 2003 [1]<br>(unpublished)<br>***(Feldman 2005 [2] Nankervis [3])***	RCT<br>2 weeks treatment, then followed up for additional 2 weeks<br>*Cochrane risk of bias tool: randomisation described, allocation concealment unclear, intention-to-treat unclear.*	**Intervention:** Clobetasol propionate 0.05% lotion (twice a day) **(n=96)**<br>**Intervention:** Clobetasol propionate 0.05% emollient cream (twice a day) (n=100)<br>**Comparator:** Vehicle (n=33)	**Severity:** moderate to severe<br>**Age:** $\geq$ 12 years<br>**Sample size:** 229 participants	**<u>Local application site skin reactions</u>**<br>No clinically significant telangiectasia or skin thinning		**<u>Unspecified adverse events</u>**<br>Incidence comparable between groups.<br>**<u>Treatment-related adverse events</u>**<br>**Clobetasol lotion** = 4/96 patients (4.2%); **Clobetasol cream** = 1/100 patients (1%)<br>**Vehicle** = 6/33 patients (18.2%)<br>*(Difference between groups: p= 0.0006[a])*
Kimball 2008 [4] (trial a)<br>***(Frangos 2008 [5])***	RCT<br>Duration not specified in review<br>*Risk of bias not assessed in any of the included systematic reviews.*	**Intervention:** Clobetasol propionate emulsion formulation foam 0.05%<br>**Comparator:** Vehicle	**Severity:** not specified in the review<br>**Age:** not specified in the review<br>**Sample size:** not specified in the review			**<u>Incidence of adverse events or treatment related adverse events</u>**<br>**Clobetasol foam** = 8%<br>**Vehicle foam** = 10%<br>*(no significant differences between groups)*
Rosso 2009 [6]<br>***(Barnes 2009 [7])***	RCT<br>2 weeks treatment<br>*Risk of bias not assessed in any of the included systematic reviews.*	**Intervention:** Fluocinonide 0.1% cream (n=109)<br>**Comparator:** Vehicle (n=50)	**Severity:** not specified in the review<br>**Age:** not specified in the review<br>**Sample size:** 159 participants	**<u>Skin thinning</u>**<br>**Fluocinonide:** 6/109 participants (5.6%)<br>**Vehicle:** 2/50 participants (4.3%)<br>*(Difference between groups: p=0.69[a])*		
www.olux-e.com (online data) [8]<br>***(Frangos 2008 [5])***	Single arm study (observational)<br>2 weeks treatment<br>*Risk of bias not assessed in any of the included systematic reviews.*	**Intervention:** Clobetasol propionate emollient foam (twice daily) (n=37)<br>**Comparator:** No comparator	**Severity:** $\geq$30% BSA<br>**Age:** $\geq$12 years old<br>**Sample size:** 37 participants		**<u>HPA axis suppression</u>**<br>6/37 patients (16%)<br>(not specified in the review *how it was measured)*	

For peer review only

Kimball 2008 [4] (trial b)<br>***(Frangos 2008 [5]; Wood Heickman 2018 [9])***	Open label Phase II safety study<br>2 weeks treatment<br>*Risk of bias not assessed in any of the included systematic reviews.*	**Intervention:** Clobetasol propionate emollient foam 0.05% (twice daily) (n=52)<br>**Comparator:** No comparator	**Severity:** mild to severe<br>**Age:** children (from 6 years old) and adults<br>**Sample size:** 52 participants		<u>**HPA axis suppression**</u><br>7/30 (23.3%) had adrenal insufficiency (ACTH stimulation testing, measuring serum cortisol levels).<br>• 47% of children (aged 6-11)<br>• 0% of adolescents (aged 12-17)<br>• 27% of adults (≥18 years)<br>Was reported as transient and reversible. After TCS discontinuation, children with biochemical adrenal insufficiency had complete resolution at retesting.		
Herz 1991 [10]<br>***(Barnes 2015 [7])***	Single arm study (observational)<br>*(2 weeks treatment)*<br>*Risk of bias not assessed in any of the included systematic reviews.*	**Intervention:** Clobetasol propionate (n=59)<br>**Comparator:** No comparator	**Severity:** not specified in the review<br>**Age:** not specified in the review<br>**Sample size:** 59 participants	<u>**Skin thinning**</u> 1 case of skin thinning reported (not clear if in a psoriasis or eczema patient – but assume its eczema as this is the topic of the systematic review).			
**Potent topical corticosteroids**							
Sugarman 2009 [11]<br>***(Van Zuuren 2017 [12])***	RCT<br>*(4 weeks treatment)*<br>*(Cochrane risk of bias tool: low risk of selection, attrition and other biases. Unclear risk of reporting and performance bias, High risk of detection bias. [12])*<br>*(Cochrane risk of bias tool: unclear risk of selection bias, high risk from no blinding. [3])*	**Intervention:** Fluticasone 0.05% cream twice daily (hydrocortisone 2.5% for the face and body folds) (n=62)<br>**Comparator:** Ceramide-dominant barrier repair formulation (EpiCeram) twice daily (emollient) (n=59)	**Severity:** moderate to severe<br>**Age:** children 6 months to 18 years (mean age 7.1 years)<br>**Sample size:** 121 participants			<u>**Serious adverse events**</u><br>The participants did not report any in either group.<br>No further details regarding other possible treatment related adverse events were reported.	
Griffiths 2002 [13]<br>***(Nankervis 2017 [3])***	RCT<br>*(up to 14 days treatment)*<br>*(Cochrane risk of bias tool: low risk of selection*	**Intervention:** Hydrocortisone 17-butyrate cream (0.1%) maximum application of 2g (four fingertip units) per day (n=49)	**Severity:** not specified<br>**Age:** adults ≥18 years old	No difference in cutaneous adverse events which were possibly or probably related to treatment in either group (p = 0.13)		<u>**Unspecified adverse events**</u><br>**Hydrocortisone group:** 20/49 (40.8%) participants reported 41 adverse events in total.	

For peer review only

	bias from sequence generation, unclear risk of selection bias from allocation concealment, low risk from blinding. [3])	**Comparator:** Cipamfylline cream (1.5 mg of cipamfylline per gram of cream) used up to a maximum of 2 g (four fingertip units) of cream per day (emollient) (n=54)	**Sample size:** 103 participants	The adverse events were mostly application site reactions, including itching, stinging or burning, and drug reactions.		**Emollient:** 29/52 (55.8%) participants reported 63 adverse events in total.<br>*(Difference between groups: p= 0.14 [a])*
Wu 2013 [14]<br>***(Nankervis 2017 [3])***	RCT<br>*(10 days treatment)*<br>*(Cochrane risk of bias tool: low risk of selection bias from sequence generation. Unclear risk of selection bias from allocation concealment, unclear risk from blinding and other biases: Two out of 60 participants were excluded from the analyses as they used concomitant medication [3])*	**Intervention:** Mometasone furoate 0.1% cream, twice a day (n=20)**Comparator:** placebo of distilled water in 1% dimethyl sulfoxide mixed with the identical cream base as used for the 15(R/S)-methyl-lipoxin A4 (n=20)<br>**Comparator:** 15(R/S)-methyl-lipoxin A4 0.1% cream (n=20)	**Severity:** not specified<br>**Age:** not specified<br>**Sample size:** 60 participants		None of the safety tests (e.g. full blood count, kidney and liver function test, and electrocardiogram) showed any significant differences compared with baseline for all three treatment groups.	No clinical adverse events were reported.
Eichenfield 2006 [15]<br>***(Nankervis 2017 [3])***	RCT<br>*(Duration not specified in the review)*<br>*Risk of bias not assessed*	**Intervention:** Fluticasone propionate<br>**Comparator:** Vehicle	**Severity:** not specified in the review<br>**Age:** children from 3 months old<br>**Sample size:** not specified in the review			**<u>Withdrawal due to adverse events</u>**<br>**Topical corticosteroids:** 4 participants in total from this study and from Hebert 2007
Pellanda 2005 [16]<br>***(Nankervis 2017 [3])***	RCT<br>*(Duration not specified in the review)*<br>*Risk of bias not assessed*	**Intervention:** Triamcinolone acetonide<br>**Comparator:** Vehicle	**Severity:** mild to moderate<br>**Age:** not specified in the review<br>**Sample size:** not specified in the review	**<u>Skin changes</u>**<br>One report by a participant using placebo (no further details)		
Lebwohl 1996 [17]<br>***(Hoare 2000 [18])***	RCT<br>*(29 days treatment)*<br>*(Moher 1995 quality checklist: method and*	**Intervention:** Fluticasone propionate ointment 0.005%<br>**Comparator:** Vehicle	**Severity:** not specified in the review			The review authors only reported that *"Drug related adverse effects were rare"*

For peer review only

	concealment of randomisation unclear, double blinded, large number of withdrawals and dropouts, no ITT analysis [18])		**Age:** not specified in the review<br>**Sample size:** 203 participants			
Lebwohl 1999 [19]<br>***(Hoare 2000 [18])***	RCT<br>*(29 days treatment)*<br>*(Moher 1995 quality checklist: method and concealment of randomisation unclear, double blinded, large number of withdrawals and dropouts, no ITT analysis [18])*	**Intervention:** Fluticasone propionate ointment 0.005%<br>**Comparator:** Vehicle	**Severity:** not specified in the review<br>**Age:** not specified in the review<br>**Sample size:** 169 participants			The review authors only reported that *"Drug related adverse effects were rare"*
Friedlander 2002 [20]<br>***(Callen 2007 [21]; Wood Heickman 2018 [9])***	Single arm study (observational)<br>*(3 to 4 weeks)*<br>*Risk of bias not assessed in any of the included systematic reviews.*	**Intervention:** Fluticasone propionate cream 0.05% (n=43)<br>**Comparator:** No comparator	**Severity:** not specified in the review<br>**Age:** children 3 months to 6 years<br>**Sample size:** 43 participants		**HPA axis suppression**<br>2/43 (4.7%) children (measured using ACTH stimulation testing, measuring serum cortisol levels)<br>After TCS discontinuation, children with biochemical adrenal insufficiency had complete resolution at retesting.	
Eichenfield 2007 [22]<br>***(Wood Heickman 2018 [9])***	Single arm study (observational)<br>(4 weeks treatment)<br>*Risk of bias not assessed in any of the included systematic reviews.*	**Intervention:** Hydrocortisone butyrate 0.1% (n=20)<br>**Comparator:** No comparator	**Severity:** not specified in the review<br>**Age:** children (median or mean = 9 years)<br>**Sample size**: 20 children		**HPA axis suppression**<br>0/20 (0%) children (measured using ACTH stimulation testing, measuring serum cortisol levels)	
Hebert 2006 [23]<br>***(Wood Heickman 2018 [9])***	Single arm study (observational)<br>(3 to 4 weeks treatment)<br>*Risk of bias not assessed in any of the included systematic reviews.*	**Intervention:** Fluticasone propionate 0.05% lotion (n=42)<br>**Comparator:** No comparator	**Severity:** not specified in the review<br>**Age:** children (median or mean 2.6 years)<br>**Sample size**: 42 children		**HPA axis suppression**<br>0/42 (0%) children (measured using ACTH stimulation testing, measuring serum cortisol levels)	

For peer review only

Abramovitis 2010 [24]<br>***(Wood Heickman 2018 [9])***	Single arm study (observational)<br>(29 days treatment)<br>*Risk of bias not assessed in any of the included systematic reviews.*	**Intervention:** Hydrocortisone butyrate 0.1% cream (n=63)<br>**Comparator:** No comparator	**Severity:** not specified in the review<br>**Age:** children (median or mean 6.3 years)<br>**Sample size**: 63 children		**HPA axis suppression**<br>5/63 (7.9%) children (measured using ACTH stimulation testing, measuring serum cortisol levels)<br>After TCS discontinuation, children with biochemical adrenal insufficiency had complete resolution at retesting.	
**Moderate potency topical corticosteroids**						
De Belilovsky 2011 [25]<br>***(Van Zuuren 2017 [12])***	RCT<br>*(3 weeks treatment)*<br>*(Cochrane risk of bias tool: low risk of selection, attrition, reporting and other biases. Unclear risk of performance bias. High risk of detection bias. [12])*<br>*(Cochrane risk of bias tool: unclear risk of selection bias and risk from blinding. [3])*	**Intervention:** Hydrocortisone butyric propionate 0.1% twice daily (n=40)<br>**Comparator:** Stelatopia (2% sunflower oil, fatty acids, ceramides) twice daily (n=40)	**Severity:** mild to moderate<br>**Age:** children 4 months to 4 years (mean age 2.3 years)<br>**Sample size:** 80 participants			No participants reported adverse events
Rosenthal 1980 [26]<br>***(Singh 2012 [27])***	RCT<br>(*14 days treatment)*<br>*(Delphi list: method of randomisation not described, allocation not concealed, blinded, no ITT analysis [27])*	**Intervention:** Clocortolone pivalate 0.1% cream (applied thrice daily)<br>**Comparator:** Vehicle (applied thrice daily)	**Severity:** not specified in the review<br>**Age:** not specified in the review<br>**Sample size:** 100 participants			No adverse events
Binder 1977 [28]<br>***(Singh 2012 [27])***	RCT<br>*(14 days treatment)*<br>*(Delphi list: method of randomisation not described, allocation not concealed, blinded, no ITT analysis [27])*	**Intervention:** Clocortolone pivalate (applied thrice daily) (n=17)<br>**Comparator:** Vehicle (n=12)	**Severity:** not specified in the review<br>**Age:** mean age 30 years<br>**Sample size:** 29 participants	**Irritation and dryness**<br>Clinically significant in one patient in each group – did not result in discontinuation.		
Nolting 1991 [29]	RCT (but safety data only presented for one arm)	**Intervention:** Prednicarbate cream 0.25% (2 applications per day) (n=34)	**Severity:** Disease duration = mean 4.1 years ± 2.7			**Adverse reactions**<br>2/34 patients (5.9%)

For peer review only

For peer review only - http://bmjopen.bmj.com/site/about/guidelines.xhtml

(De Tiedra 1997 [30])	*(21 days treatment)*<br>*Risk of bias not assessed in any of the included systematic reviews.*	**Comparator:** mometasone cream 0.1% twice daily (no safety data given)	**Age:** children 2-12 years (mean 6.6 ± 3.6).<br>**Sample size:** 34 participants (with safety data)			
Rampini 1992 [31]<br>***(De Tiedra 1997 [30])***	RCT (but safety data only presented for one arm)<br>*(21 days treatment)*<br>*(Moher 1995 quality checklist: method and concealment of randomisation unclear, double blinded, two dropouts, no ITT analysis, [18])*	Intervention: Prednicarbate cream/unguent 0.25% (2 applications per day) (n=93)<br>Comparator:methylprednisolone aceponate 0.1% once daily (no safety data given)	**Severity:** not specified in the review<br>**Age:** children 0.3 to 14 years (mean 6.6).<br>**Sample size:** 93 participants (with safety data)			**<u>Adverse reactions</u>**<br>3/93 patients (3.2%)
Camacho 1996 [32]<br>***(De Tiedra 1997 [30])***	RCT (but safety data only presented for one arm)<br>*(21 days treatment)*<br>*(Moher 1995 quality checklist: method and concealment of randomisation unclear, double blinded, no ITT analysis, 14/49 dropouts, [18])*	**Intervention:** Prednicarbate cream 0.25% (2 applications per day) (n=49)<br>**Comparator:** fluocortolone pivalate cream 0.2% (no safety data given)	**Severity:** Disease duration= mean 6.2 years ± 8.2 (range 0.25 to 39 years).<br>**Age:** adults 19 to 65 years (mean 34.1 ± 12).<br>**Sample size:** 49 participants (with safety data)			**<u>Adverse reactions</u>**<br>4/49 patients (8.1%)
Gimenez Camarasa 1994 [33]<br>***(De Tiedra 1997 [30])***	RCT (but safety data only presented for one arm)<br>*(21 days treatment)*<br>*Risk of bias not assessed in any of the included systematic reviews.*	**Intervention:** Prednicarbate cream 0.25% (2 applications per day) (n=41)<br>**Comparator**: fluocinolone cream 0.025% twice daily (no safety data given)	**Severity:** Disease duration = mean 6.4 years ± 8.6 (range 0-40).<br>**Age:** adults 18 to 77 years (mean 37.6 ± 15.9).<br>**Sample size:** 41 participants (with safety data)			**<u>Adverse reactions</u>**<br>0/41 patients (0%)
Moshang 2001 [34]	Single arm study (observational)<br>*(3 weeks treatment)*	**Intervention:** Prednicarbate emollient cream 0.1%, twice daily (n=55)<br>**Comparator:** No comparator	**Severity:** not specified in the review		**<u>HPA axis suppression</u>**<br>All normal (measured using ACTH stimulation testing, measuring serum cortisol levels)	

For peer review only

For peer review only - http://bmjopen.bmj.com/site/about/guidelines.xhtml

(Callen 2007 [21]; Wood Heickman 2018 [9])	*Risk of bias not assessed in any of the included systematic reviews.*		**Age:** children 4 months to 12 years<br>**Sample size:** 55 participants			
Conde 2008 [35]<br>***(Singh 2012 [27])***	Single arm study (observational)<br>*(4 weeks treatment)*<br>*Risk of bias not assessed in any of the included systematic reviews.*	**Intervention:** Clocortolone pivalate cream 0.1% twice daily (n=10)<br>**Comparator:** No comparator	**Severity:** mild to moderate<br>**Age:** children, mean age 7.9 years<br>**Sample size:** 10 participants			No adverse events reported
Crespi 1986 [36]<br>***(Callen 2007 [21])***	Single arm study (observational)<br>*(4 weeks treatment)*<br>*Risk of bias not assessed in any of the included systematic reviews.*	**Intervention:** Alclometasone cream, twice daily (n=39)<br>**Comparator:** No comparator	**Severity:** not specified in the review<br>**Age:** children<br>**Sample size:** 39 participants		**HPA axis suppression**<br>All normal (measured via morning cortisol)	
**Mild potency topical corticosteroids**						
Udompataikul 2011 [37]<br>***(Van Zuuren 2017 [12])***	Within-participant RCT<br>*(6 weeks treatment)*<br>*(Cochrane risk of bias tool: unclear risk of selection, performance and attrition bias. Low risk of reporting and other biases. High risk of detection bias. [12])*<br>*(Cochrane risk of bias tool: unclear risk of selection bias from sequence generation and risk from blinding. Low risk of selection bias from allocation concealment, [3])*	**Intervention:** Hydrocortisone acetate 1% cream twice daily, was applied one side of the body for 4 weeks followed by the cream base for 2 weeks.<br>**Comparator:** Licochalcone (containing *Glycyrrhiza inflata* root extract, decanediol, menthoxypropanediol and 6-fatty acids) applied twice daily on one side of the body for 6 weeks	**Severity:** mild to moderate<br>**Age:** children 2 months to 10 years (mean age 5.8 years)<br>**Sample size:** 30 participants			No adverse events on either side during the study.
Wananukul 2013 [38]<br>***(Van Zuuren 2017 [12])***	Within-participant RCT<br>*(4 weeks treatment)*<br>*(Cochrane risk of bias tool: low risk of selection bias from sequence*	**Intervention:** Hydrocortisone acetate 1% cream twice daily on one side of the body<br>**Comparator:** Licochalcone (containing *Glycyrrhiza inflata*	**Severity:** mild to moderate<br>**Age:** children, mean age 3.1 years			No adverse events on either side during the study

For peer review only - http://bmjopen.bmj.com/site/about/guidelines.xhtml

	generation, performance, detection, attrition, reporting and other biases. Unclear risk of selection bias from allocation concealment. [12]*)*	root extract, decanediol, menthoxypropanediol and 6-fatty acids) twice daily on one side of the body	**Sample size:** 55 participants				
Jirabundansuk 2014 [39] ***(Van Zuuren 2017*** [12]***)***	Within-participant RCT *(4 weeks treatment)* *(Cochrane risk of bias tool: Unclear risk of selection and performance bias. High risk of detection bias. Low risk of attrition, reporting and other biases.* [12]*)*	**Intervention:** Hydrocortisone acetate 1% cream twice daily on one side of the body **Comparator:** Moisturiser containing spent grain, Vitellaria paradoxa (formerly Butyrospermum parkii) extract plus Argania spinosa kernel oil twice daily on one side of the body	**Severity:** Mild or moderate **Age:** children 2-15 years (mean age 4.3 years) **Sample size:** 31 participants				The investigators stated that "no specific adverse events were reported".
Dolle 2010 [40] ***(Nankervis 2017*** [3]***)***	Within-participant RCT *(3 weeks treatment)* *(Cochrane risk of bias tool: unclear risk of selection bias and risk from blinding.* [3]*)*	**Intervention:** 1% hydrocortisone solution once daily for 1st week then twice daily up to 3 weeks **Comparator:** 6% miltefosine solution once daily for 1st week then twice daily up to 3 weeks	**Severity:** moderate to severe **Age:** adults (≥18 years old) **Sample size:** 16 participants	**Local topical adverse events related to the treatment** **Hydrocortisone:** 7/16 participants (44%) **Emollient:** 10/16 participants (63%) These adverse events included pruritus, burning, tingling and dry skin. Dry skin was seen only with emollient treatment.	No systemic adverse events		No withdrawals because of adverse events
Patzelt-Wenczler 2000 [41] ***(Nankervis 2017*** [3]***)***	Within-participant RCT *(2 weeks treatment)* *(Cochrane risk of bias tool: unclear risk of selection bias and high risk from no blinding.* [3]*)*	**Intervention:** Hydrocortisone 0.5% twice daily **Comparator:** Kamillosan® cream, containing 2% ethanolic extract of chamomile flowers, twice daily (emollient) Comparator: Vehicle cream applied twice daily	**Severity:** at least moderate **Age:** not specified in the review **Sample size:** 72 participants				Three participants in the emollient group withdrew early because of intolerability.
Hebert 2007 [42] ***(Nankervis 2017*** [3]***)***	RCT *(Duration not specified in the review)* *Risk of bias not assessed in any of the included systematic reviews.*	**Intervention:** Desonide **Comparator:** Vehicle	**Severity:** not specified in the review **Age:** children				**Serious adverse events** One event reported in TCS group but not thought to be related to treatment **Withdrawal due to adverse events**

For peer review only

			**Sample size:** not specified in the review			TCS group: 4 in total from this study and from Eichenfield 2006
Paller 2003 [43]<br>***(Nankervis 2017 [3])***	RCT<br>*(2 weeks treatment)*<br>*Risk of bias not assessed in any of the included systematic reviews.*	**Intervention:** Fluocinolone acetonide 0.01% twice daily (n=45)<br>**Comparator:** Vehicle twice daily (n=49)	**Severity:** not specified in the review<br>**Age:** children from 2 to 12 years old<br>**Sample size:** 94 participants	**<u>Mild hypopigmentation</u>**<br>Two participants out of 45 reported this event with fluocinolone (4.4%)		
Patel 1995 [44]<br>***(Callen 2007 [21])***	Single arm study (observational)<br>*(3-10 years follow up)*<br>*Risk of bias not assessed in any of the included systematic reviews.*	**Intervention:** 1% Hydrocortisone ointment (n=14; 9/14 intermittently used moderate to high potency)<br>**Comparator:** No comparator	**Severity:** not specified in the review<br>**Age:** children 3.1 to 10.7 years<br>**Sample size:** 14 participants		**<u>HPA axis suppression</u>**<br>Plasma cortisol levels - no change in basal/peak levels but peaked earlier	
Dohil 2009 [45]<br>(Wood Heickman 2018 [9])	Single arm study (observational)<br>(4 weeks duration)<br>*Risk of bias not assessed in any of the included systematic reviews.*	**Intervention:** fluocinolone acetonide 0.01%<br>**Comparator:** No comparator	**Severity:** not specified in the review<br>**Age:** children (median or mean age 1.1 years)<br>**Sample size:** 24 participants		**<u>HPA axis suppression</u>**<br>No cases of adrenal insufficiency *(measured using ACTH stimulation testing, measuring serum cortisol levels)*	
Eichenfield 2007 [46]<br>(Wood Heickman 2018 [9])	Single arm study (observational)<br>(4 weeks duration)<br>*Risk of bias not assessed in any of the included systematic reviews.*	**Intervention:** Desonide hydrogel 0.05%<br>**Comparator:** No comparator	**Severity:** not specified in the review<br>**Age:** children (median or mean age 3.3 years)<br>**Sample size:** 34 participants		**<u>HPA axis suppression</u>**<br>No cases of adrenal insufficiency *<u>(measured using ACTH stimulation testing, measuring serum cortisol levels)</u>*	
Hebert 2008 [47]<br>(Wood Heickman 2018 [9])	Single arm study (observational)<br>(4 weeks duration)	**Intervention:** Desonide 0.05% foam<br>**Comparator:** No comparator	**Severity:** not specified in the review		**<u>HPA axis suppression</u>**<br>Three out of 75 participants had adrenal insufficiency *(measured using ACTH stimulation testing, measuring serum cortisol levels)*	

For peer review only

	Risk of bias not assessed in any of the included systematic reviews.		**Age:** children (median or mean age 6.7 years)<br>**Sample size:** 75 participants			
**How safe are topical corticosteroids compared to topical calcineurin inhibitors?**						
**Study ID *(Systematic review*)***	**Study design and study duration *(Quality assessment)***	**Intervention and comparator**	**Participants**	**Cutaneous adverse events**	**Systemic adverse events**	**Unspecified adverse events**
**Potent topical corticosteroids**						
Bieber 2007 [48]<br>***(Broeders 2016 [49])***	RCT<br>*(up to 3 weeks treatment)*<br>*(Jadad score 4/5 – risk from sequence generation and allocation concealment [49])*<br>*(Cochrane risk of bias tool: unclear risk of selection bias and from blinding. [3])*<br>*(Cochrane risk of bias tool: unclear risk of selection bias. Low risk of performance, detection, attrition, reporting and other biases. [50])*	**Intervention:** Methyl-prednisolone 0.1% (n=129) once daily in the evening to all affected body surface areas for a minimum of 2 weeks and a maximum of 3 weeks and cleared areas treated for an additional 7 days post clearance. Also applied a vehicle ointment in the morning to maintain blinding.<br>**Comparator:** Tacrolimus 0.03% (n=136), applied twice daily, morning and evening, to all affected body surface areas for a minimum of 2 weeks and a maximum of 3 weeks and cleared areas treated for an additional 7 days post clearance.	**Severity:** severe to very severe<br>**Age:** children 2 to 15 years old<br>**Sample size:** 265 participants	**Adverse events related to treatment**<br>**Methyl-prednisolone:** 0/129 participants (0%)<br>**Tacrolimus:** 6/136 participants (4.4%)<br>*(Difference between groups: p=0.09 [a, b])*		**Severe adverse events**<br>**Methyl-prednisolone:** 0/129 participants (0%)<br>**Tacrolimus:** 6/136 participants (4.4%)<br>*(Difference between groups: p=0.09 [a, c])*<br>**Adverse events requiring discontinuation**<br>**Methyl-prednisolone:** 0/129 (0%)<br>**Tacrolimus:** 4/136 (3%)<br>*(Difference between groups: p=0.15 [a])*
Doss 2010 [51]<br>***(Broeders 2016 [49])***	RCT<br>*(3 weeks treatment twice daily, plus 3 weeks follow up with once daily treatment)*<br>*(Jadad score 5/5 – risk from allocation concealment [49])*<br>*(Cochrane risk of bias tool: unclear risk of*	**Intervention:** Fluticasone 0.005% ointment applied twice daily to all affected areas except eyelids until clearance, up to 3 weeks. All participants who responded to treatment could apply treatment once a day to the remaining lesions for another 3 weeks (n=239)<br>**Comparator:** Tacrolimus 0.03% ointment applied twice daily to all affected areas except eyelids until clearance, up to 3 weeks.	**Severity:** moderate to severe<br>**Age:** children 2 to 15 years old<br>**Sample size:** 478 participants	**Adverse events related to treatment**<br>**Fluticasone:** 45/239 participants (19%)<br>**Tacrolimus:** 55/239 participants (23%)<br>*(Difference between groups: p=0.26 [a])*<br>**Skin burning**<br>**Fluticasone:** 6/239 (2.5%)<br>**Tacrolimus:** 18/237 (7.6%)		**Severe adverse events**<br>**Fluticasone:** 2/239 participants (0.8%)<br>**Tacrolimus:** 1/239 participants (0.4%)<br>*(Difference between groups: p=0.57 [a])*<br>**Adverse events requiring discontinuation**<br>**Fluticasone:** 6/239 participants (2.5%)<br>**Tacrolimus:** 4/239 participants (1.7%)

	selection bias and from blinding. [3])<br>*(Cochrane risk of bias tool: Low risk of selection, performance, detection, attrition, reporting and other biases. [50])*<br>*Cochrane risk of bias tool: low risk of selection, performance, attrition, reporting and other biases. Unclear risk of performance bias [52]).*	All participants who responded to treatment could apply treatment once a day to the remaining lesions for another 3 weeks (n=239)		*(Difference between groups: p=0.02 [a])*<br>**Pruritus**<br>**Fluticasone:** 8/239 participants (3.3%)<br>**Tacrolimus:** 10/237 participants (4.2%)<br>*(Difference between groups: p=0.62 [a])*<br>**Skin infection**<br>**Fluticasone:** 49/239 participants (21%)<br>**Tacrolimus:** 44/239 participants (18%)<br>*(Difference between groups: p=0.56 [a])*		*(Difference between groups: p=0.53 [a])*
Doss 2009 [53]<br>***(Broeders 2016 [49])***	RCT<br>*(3 weeks of treatment – then for a further 3 weeks either stop treatment, once daily treatment or switch to other treatment twice daily)*<br>*(Jadad score 5/5 – risk from allocation concealment [49])*<br>*(Cochrane risk of bias tool: unclear risk of selection bias and low risk from blinding. [3])*	**Intervention:** Fluticasone 0.005% ointment twice daily on facial eczema lesions for 3 weeks or until clearance (n=279)<br>**Comparator:** Tacrolimus 0.1% twice daily on facial eczema lesions for 3 weeks or until clearance (n=287)<br>For 21 days after the initial 3 weeks, the participants could stop treatments if the facial lesions had cleared; stay on the same treatment once a day; or swap treatment using it twice daily (still blinded)	**Severity:** moderate to severe<br>**Age:** adults<br>**Sample size:** 566 participants	**Adverse events related to treatment**<br>**Fluticasone:** 42/279 participants (15%)<br>**Tacrolimus:** 75/287 participants (26%)<br>*(Difference between groups: p=0.001 [a])*<br>**Skin burning**<br>**Fluticasone:** 9/279 participants (3.2%)<br>**Tacrolimus:** 47/287 participants (16.4%)<br>*(Difference between groups: p<0.00001 [a])*<br>**Pruritus**<br>**Fluticasone:** 9/279 participants (3.2%)<br>**Tacrolimus:** 12/287 participants (4.2%)<br>*(Difference between groups: p=0.55 [a])*		**Severe adverse events**<br>**Fluticasone:** 0/279 participants (0%)<br>**Tacrolimus:** 1/287 participants (0.3%)<br>*(Difference between groups: p=0.51 [a]).*<br>**Adverse events requiring discontinuation**<br>**Fluticasone:** 8/279 participants (2.9%)<br>**Tacrolimus:** 7/287 participants (2.4%)<br>*(Difference between groups: p=0.75 [a])*
Luger 2001 [54]<br>***(Broeders 2016 [49])***	RCT<br>*(up to 3 weeks treatment)*	**Intervention:** Betamethasone valerate 0.1% applied twice daily on all affected areas except for the head and neck for up to 3 weeks or until complete	**Severity:** moderate<br>**Age:** adults ≥ 18 years old	**Pruritus**<br>**Betamethasone:** 5/42 participants (12%)<br>**Pimecrolimus:** 14/45 participants (31%)		**Adverse events requiring discontinuation**<br>**Betamethasone:** 1/42 participants (2.4%)

For peer review only

	(Jadad score 3/5 – risk from sequence generation and allocation concealment [49])<br>*(Cochrane risk of bias tool: unclear risk of selection bias and unclear risk from blinding. [3])*<br>*(Jadad scale: 3/5 [55])*<br>*(Cochrane risk of bias tool: unclear risk of selection bias, adequate blinding, inadequate loss to follow up. [56])*	clearance if this was sooner (n=42)<br>**Comparator:** Pimecrolimus 1% applied twice daily on all affected areas except for the head and neck for up to 3 weeks or until complete clearance if this was sooner (n=45)	**Sample size:** 87 participants	*(Difference between groups: p=0.04 [a])*<br>**Skin burning**<br>**Betamethasone:** 4/42 participants (9.5%)<br>**Pimecrolimus:** 22/45 participants (49%)<br>*(Difference between groups: p=0.001 [a])*		**Pimecrolimus:** 3/45 participants (6.7%)<br>*(Difference between groups: p=0.36 [a])*
Luger 2004 [57]<br>***(Broeders 2016 [49])***	*RCT*<br>*(52 weeks. Twice daily until clearance, restarted with flares)*<br>*(Jadad score 3/5 – risk from sequence generation and allocation concealment [49])*<br>*(Cochrane risk of bias tool: unclear risk of selection bias, low risk from blinding. [3])*<br>*(Jadad scale: 3/5 [55])*<br>*(Cochrane risk of bias tool: adequate allocation generation, unclear allocation concealment, adequate blinding, inadequate loss to follow up. [56])*	**Intervention:** Triamcinolone 0.1% (potent) and Hydrocortisone acetate 1% (face) (Mild potency) twice daily until complete clearance and itching had stopped, then treatment restarted if inflammation recurred (n=330)<br>**Comparator:** Pimecrolimus 1% twice daily until complete clearance and itching had stopped, then treatment restarted if inflammation recurred (n=328)	**Severity:** moderate to severe<br>**Age:** adults (age 18 to 79 years)<br>**Sample size:** 658 participants	**Skin burning**<br>**Triamcinolone + hydrocortisone:** 36/330 participants (11%)<br>**Pimecrolimus:** 85/328 participants (26%)<br>*(Difference between groups: p<0.00001 [a])*<br>**Pruritus**<br>**Triamcinolone + hydrocortisone:** 6/330 participants (1.8%)<br>**Pimecrolimus:** 18/328 participants (5.5%)<br>*(Difference between groups: p=0.02 [a])*<br>**Skin thinning**<br>**Triamcinolone + hydrocortisone:** 3/330 participants (0.9%)<br>**Pimecrolimus:** 0/328 participants (0%)<br>*(Difference between groups: p=20 [a])*<br>**Skin infection**<br>**Triamcinolone + hydrocortisone:** 80/330 participants (24%)<br>**Pimecrolimus:** 69/328 participants (21%)<br>*(Difference between groups: p=0.33 [a])*		**Severe adverse events**<br>**Triamcinolone + hydrocortisone:** 21/330 participants (6.4%)<br>**Pimecrolimus:** 16/328 participants (4.9%)<br>*(Difference between groups: p=0.41 [a])*

For peer review only

Mandelin 2010 [58]<br><br>***(Broeders 2016 [49])***	RCT<br><br>*(52 weeks, as prescribed until 7 days after clearance, then restarted with flares)*<br><br>*(Jadad score 3/5 – risk from sequence generation and allocation concealment, [49])*<br><br>*(Cochrane risk of bias tool: unclear risk of selection bias, risk from no blinding. [3])*	**Intervention:** Hydrocortisone butyrate 0.1% ointment (potent) and Hydrocortisone acetate 1% ointment (face) (Mild potency) twice daily, as prescribed, for a flare until 7 days after clearance, as many times as required in 1 year (n=40)<br><br>**Comparator:** Tacrolimus 0.1% ointment twice daily, as prescribed, for a flare until 7 days after clearance, as many times as required in 1 year (n=40)	**Severity:** moderate to severe<br><br>**Age:** adults<br><br>**Sample size:** 80 participants	**<u>Skin thinning</u>**<br>**Hydrocortisone:** 2/40 participants (5%)<br>**Tacrolimus:** 0/40 participants (0%)<br>*(Difference between groups: p=0.29 [a])*<br><br>**<u>Skin infection</u>**<br>**Hydrocortisone:** 17/40 participants (43%)<br>**Tacrolimus:** 26/40 participants (65%)<br>*(Difference between groups: p=0.05 [a])*		**Severe adverse events**<br>None in either group

For peer review only

Reitamo 2002 (I) [59]<br><br>***(Broeders 2016*** [49]***; Iskedjian 2004*** [60]***)***	RCT<br><br>*(3 weeks treatment)*<br><br>*(Jadad score 4/5 – risk from allocation concealment* [49]*)*<br><br>*(Cochrane risk of bias tool: unclear risk of selection bias, unclear risk from blinding.* [3]*)*<br><br>*(Jadad scale: 5/5,* [55]*)*<br><br>*(Cochrane risk of bias tool: Low risk of selection, performance, detection, attrition, reporting and other biases.* [50]*)*<br><br>*(Cochrane risk of bias tool: adequate randomisation and allocation concealment, blinding and ITT analysis done.* [61]*)*	**Intervention:** Hydrocortisone butyrate 0.1% twice daily for 3 weeks (n=186)<br><br>**Comparator:** Tacrolimus 0.1% twice daily for 3 weeks (n=191)<br><br>**Comparator:** Tacrolimus 0.03% twice daily for 3 weeks (arm not included in Broeders 2016 review) (n=193)	**Severity:** moderate to severe<br><br>**Age:** adults (age 16 to 70 years)<br><br>**Sample size: 571** participants	**Skin burning**<br>**Hydrocortisone:** 24/186 participants (13%)<br>**Tacrolimus 0.1%:** 113/191 participants (59%)<br>*(Difference between groups: p<0.00001* [a]*)*<br><br>**Pruritus**<br>**Hydrocortisone:** 18/186 participants (9.7%)<br>**Tacrolimus 0.1%:** 29/191 participants (15%)<br>*(Difference between groups: p=0.11* [a]*)*<br><br>**Erythema at application site**<br>**Hydrocortisone:** 1/186 participants (0.5%)<br>**Tacrolimus 0.1%:** 7/191 participants (3.7%)<br>**Tacrolimus 0.03%:** 4/193 participants (2.1%)<br>*(Difference between groups: tacrolimus 0.1% versus hydrocortisone: p=0.07* [a]*)*<br>*(Difference between groups: tacrolimus 0.03% versus hydrocortisone: p=0.23* [a]*)*		**Severe adverse events**<br>**Hydrocortisone:** 0/186 participants (0%)<br>**Tacrolimus 0.1%:** 1/191 participants (0.5%)<br>*(Difference between groups: p=0.51* [a]*)*<br><br>**Adverse events requiring discontinuation**<br>**Hydrocortisone:** 3/186 participants (1.6%)<br>**Tacrolimus 0.1%:** 8/191 participants (4.2%)<br>*(Difference between groups: p=0.15* [a]*)*

For peer review only

Reitamo 2005 [62]<br><br>***(Broeders 2016 [49])***	RCT<br><br>*(26 weeks)* twice daily treatment until 7 days after clearance, then whenever a flare occurs)<br><br>*(Jadad score 5/5; [49])*<br><br>*(Cochrane risk of bias tool: unclear risk of selection bias,, low risk from blinding. [3])*<br><br>*(Jadad scale: 5/5, [55])*<br><br>*(Cochrane risk of bias tool: High risk of attrition bias. Low risk of selection, performance, detection, reporting and other biases. [50])*<br><br>*(Cochrane risk of bias tool: unclear risk of selection bias, unclear if blinded, and unclear if ITT analysis used. [61])*	**Intervention:** Hydrocortisone butyrate 0.1%(potent) **and** Hydrocortisone acetate 1% (face) (Mild potency) twice daily until 7 days after clearance of eczema each time a flare of eczema occurred for 6 months (n=485)<br><br>**Comparator:** Tacrolimus 0.1% twice daily until 7 days after clearance of eczema each time a flare of eczema occurred for 6 months (n=487)	**Severity:** moderate to severe<br><br>**Age:** adults (≥18 years old)<br><br>**Sample size:** 972 participants	**<u>Adverse events related to treatment</u>**<br>**Hydrocortisone:** 11/485 participants (2.3%)<br>**Tacrolimus:** 7/487 participants (1.4%)<br>*(Difference between groups: p=0.34 [a])*<br><br>**<u>Skin burning</u>**<br>**Hydrocortisone:** 67/485 participants (14%)<br>**Tacrolimus:** 255/487 participants (52%)<br>*(Difference between groups: p<0.00001 [a])*<br><br>**<u>Pruritus</u>**<br>**Hydrocortisone:** 65/485 participants (13%)<br>**Tacrolimus:** 88/487 participants (18%)<br>*(Difference between groups: p=0.05 [a])*<br><br>**<u>Adverse events requiring discontinuation</u>**<br>**Hydrocortisone:** 16/485 participants (3.3%)<br>**Tacrolimus:** 10/487 participants (2%)<br>*(Difference between groups: p=0.23 [a])*<br><br>**<u>Skin thinning</u>**<br>**Hydrocortisone:** 2/485 participants (0.4%)<br>**Tacrolimus:** 0/487 participants (0%)<br>*(Difference between groups: p=0.30 [a])*<br><br>**<u>Skin infection</u>**<br>**Hydrocortisone:** 9/485 participants (1.9%)<br>**Tacrolimus:** 13/487 participants (2.7%)<br>*(Difference between groups: p=0.40 [a])*		**<u>Severe adverse events</u>**<br>**Hydrocortisone:** 9/485 participants (1.9%)<br>**Tacrolimus:** 5/487 participants (1%)<br>*(Difference between groups: p=0.29 [a])*<br><br>**<u>Adverse events requiring discontinuation</u>**<br>**Hydrocortisone:** 16/485 participants (3.3%)<br>**Tacrolimus:** 10/487 participants (2.1%)<br>*(Difference between groups: p=0.23 [a])*

For peer review only

For peer review only - http://bmjopen.bmj.com/site/about/guidelines.xhtml

Gradman 2007 [63]<br><br>***(Svensson 201 [64]1)***	Crossover RCT<br><br>*(2 weeks treatment)*<br><br>*(Cochrane risk of bias tool: low risk of selection bias from sequence generation, but unclear for allocation concealment. Low risk from blinding. [3])*	**Intervention:** Mometasone furoate 0.1% once daily<br><br>**Comparator:** Tacrolimus 0.1% twice daily	**Severity:** mild to moderate<br><br>**Age:** children 5 to 12 years<br><br>**Sample size:** 20 participants			**<u>Withdrawal from study</u>**<br>**Mometasone:** 1 patient<br>**Tacrolimus**: 1 patient
Kawashima 1997 [65]<br><br>(Ashcroft 2005 [55])	RCT<br><br>*(3 weeks treatment)*<br><br>*(Jadad scale: 5/5, [55])*	**Intervention:** Betamethasone valerate 0.12% twice daily for three weeks (n=89)<br><br>**Comparator:** tacrolimus 0.1% twice daily for three weeks (n=92)	**Severity:** mild to moderate<br><br>**Age:** adults<br><br>**Sample size:** 181 participants	**Skin infections**<br>**Betamethasone:** 5/89 participants<br>**Tacrolimus:** 6/92 participants<br>(*Difference between groups: p=0.80* [a])<br><br>**Skin burning**<br>**Betamethasone:** 3/89 participants<br>**Tacrolimus:** 25/92 participants<br>(*Difference between groups: p=0.0004*[a])		
**Potent or mild potency topical corticosteroids**						
Hofman 2006 [66]<br><br>***(Broeders 2016 [49]; Siegfried 2016 [67])***	RCT<br><br>*(2 weeks treatment, 28 weeks follow up)*<br><br>*(Jadad score 5/5 – risk from sequence generation and allocation concealment [49])*	**Intervention:** Hydrocortisone ointment 1% (mild potency) twice daily for head/neck and hydrocortisone butyrate ointment 0.1% (potent) for trunk and limbs for 2 weeks then hydrocortisone 1% (mild potency) twice daily for flares. (n=124)<br><br>**Comparator:** Tacrolimus 0.03% twice daily for 3 weeks then tacrolimus once daily and vehicle once daily for flares (n=133)	**Severity:** moderate to severe<br><br>**Age:** children 2 to 11 years old (mean 6 years old)<br><br>**Sample size:** 257 participants	**<u>Adverse events related to treatment</u>**<br>**Hydrocortisone:** 2/124 participants (1.6%)<br>**Tacrolimus:** 10/133 participants (7.5%)<br>*(Difference between groups: p=0.04* [a]*)*<br><br>**<u>Skin burning</u>**<br>**Hydrocortisone:** 0/124 participants (0%)<br>**Tacrolimus:** 2/133 participants (1.5%)<br>*(Difference between groups: p=0.32* [a]*)*<br><br>**<u>Pruritus</u>**<br>**Hydrocortisone:** 4/124 participants (3%)<br>**Tacrolimus:** 8/133 participants (6%)		**<u>Severe adverse events</u>**<br>**Hydrocortisone:** 0/124 participants (0%)<br>**Tacrolimus:** 2/133 participants (1.5%)<br>(*Difference between groups: p=0.32* [a])

For peer review only

				(Difference between groups: p=0.30 [a])<br>**Skin infection**<br>**Hydrocortisone:** 4/124 participants (3.2%)<br>**Tacrolimus:** 2/133 participants (1.5%)<br>*(Difference between groups: p=0.37 [a])*<br>**Bacterial infection**<br>**Hydrocortisone:** 3/124 participants (2%)<br>**Tacrolimus:** 33/133 participants (2%)<br>*(Difference between groups: p<0.0001)*<br>**Viral infection**<br>**Hydrocortisone:** incidence not reported<br>**Tacrolimus:** 1/133 participants (0.8%)		
**Moderate potency topical corticosteroids**						
Sikder 2005 [68]<br>***(Broeders 2016 [49])***	RCT<br>*(4 weeks treatment)*<br>*(Jadad score 2/5 – risk from sequence generation and allocation concealment, no blinding of observer or patients, [49])*<br>*(Cochrane risk of bias tool: Unclear risk of selection and detection bias. Low risk of performance, attrition, reporting and other biases.[50])*<br>*(Cochrane risk of bias tool: low risk of selection bias, from blinding of participants and missing data. Unclear risk from*	**Intervention:** Clobetasone 0.05% twice daily (n=15)<br>Tacrolimus 0.03% twice daily (n=15)	**Severity:** moderate to severe<br>**Age:** children 7 to 15 years old<br>**Sample size:** 30 participants	**Skin burning**<br>**Clobetasone:** 1/15 participants (6.7%)<br>**Tacrolimus**: 7/15 participants (47%)<br>*(Difference between groups: p=0.05 [a, d])*<br>**Pruritus**<br>**Clobetasone:** 2/15 participants (13%)<br>**Tacrolimus**: 3/15 participants (20%)<br>*(Difference between groups: p=0.63 [a])*		

For peer review only

	blinding outcome assessors, reporting and other biases [52]).					
Torok 2003 [69]<br><br>***(Svensson 2011 [64])***	RCT<br><br>*(3 weeks treatment)*<br><br>*(Delphi list: method of randomisation not described, allocation not concealed, blinded assessors but not participants, ITT analysis, [27])*	**Intervention:** Clocortolone pivalate 0.1% twice daily (n=19)<br><br>**Intervention:** Clocortolone 0.1% + Tacrolimus 0.1% twice daily (n=19)<br><br>**Comparator:** Tacrolimus 0.1% twice daily (n=19)	**Severity:** not specified in the review<br><br>**Age:** adults 16 to 65 years<br><br>**Sample size:** 57 participants	**Skin irritation**<br>Most commonly reported adverse event<br><br>**Skin burning**<br>More frequent in those treated with Tacrolimus 0.1%.<br><br>**Pruritus**<br>Commonly reported in both arms.<br>*(No numerical data provided in the review)*		
**Moderate or mild potency topical corticosteroids**						
Sigurgeirsson 2015 [70]<br><br>***(Broeders 2016 [49]; Siegfried 2016 [67])***	RCT<br><br>*(260 weeks used until clearance or according to country's label. Medication reinstated when a flare occurred))*<br><br>*(Jadad score 3/5 – risk from allocation concealment, no blinding of observer or patients, [49])*	**Intervention:** A moderate potency or mild potency TCS used according to the country's label with potency selected by the investigator (n=1213)<br><br>**Comparator:** Pimecrolimus 1% twice daily (n=1205)	**Severity:** mild to moderate<br><br>**Age:** children age 3 to 12 months old (mean 7 months)<br><br>**Sample size:** 2418 participants	**Skin thinning*(from online correspondence)***<br>**Topical corticosteroid:** 1/1213 participants (0.08%)<br>**Pimecrolimus:** 0/1205 (0%)<br>*(Difference between groups: p=0.50 [a])*<br><br>**Skin infection**<br>**Topical corticosteroid:** 150/1213 participants (12%)<br>**Pimecrolimus:** 157/1205 participants (13%)<br>*(Difference between groups: p=0.62 [a])*<br><br>**Cutaneous bacterial infection**<br>**Topical corticosteroid:** 121/1213 participants (10%)<br>**Pimecrolimus:** 145/1205 participants (12%)<br>*(Difference between groups: p=0.11 [a])*<br><br>**Cutaneous viral infection**<br>**Topical corticosteroid:** 279/1213 participants (23%)<br>**Pimecrolimus:** 301/12 05 participants (25%)	**Systemic bacterial infection**<br>**Topical corticosteroid:** 206/1213 participants (17%)<br>**Pimecrolimus:** 205/1205 participants (17%)<br>*(Difference between groups: p=0.98 [a])*<br><br>**Systemic viral infection**<br>**Topical corticosteroid:** 206/1213 participants (17%)<br>**Pimecrolimus:** 205/1205 participants (17%)<br>*(Difference between groups: p=0.98 [a])*<br><br>**Systemic RTI**<br>**Topical corticosteroid:** 388/1213 participants (32%)<br>**Pimecrolimus:** 422/1205 participants (35%)<br>*(Difference between groups: p=0.11 [a])*<br><br>**Systemic GI**<br>**Topical corticosteroid:** 376/1213 participants (31%)<br>**Pimecrolimus:** 386/1205 participants (32%)	**Severe adverse events**<br>**Topical corticosteroid:** 210/1213 participants (17%)<br>**Pimecrolimus:** 247/1205 participants (20%)<br>*(Difference between groups: p=0.05 [a])*<br><br>**Adverse events requiring discontinuation**<br>**Topical corticosteroid:** 12/1213 participants (1.0%)<br>**Pimecrolimus:** 7/1205 participants (0.6%)<br>*(Difference between groups: p=0.26 [a])*

For peer review only

				(Difference between groups: p=0.25 [a]*)*	*(Difference between groups: p=0.58*[a]*)*<br>**Lymphoma**<br>Zero cases in either group<br>**Growth rate and immune system**<br>No difference between groups	
**Mild potency topical corticosteroids**						
Reitamo 2002 (II) [71] ***(Broeders 2016*** [49]***; Iskedjian 2004*** [60]***)***	RCT<br>*(3 weeks treatment)*<br>*(Jadad score 5/5 – risk from allocation concealment* [49]*)*<br>*(Cochrane risk of bias tool: unclear risk of selection bias, unclear risk of blinding* [3]*)*<br>*(Jadad scale: 5/5,* [55]*)*<br>*(Cochrane risk of bias tool: Low risk of selection, performance, detection, attrition, reporting and other biases.* [50]*)*<br>*Cochrane risk of bias tool: low risk of selection, performance, attrition, reporting and other bias. Unclear risk from blinding outcome assessors* [52]*).*<br>*(Cochrane risk of bias tool: adequate method of randomisation and allocation concealment, blinding done, ITT used.* [61]*)*	**Intervention:** Hydrocortisone acetate 1% twice daily (n=185)<br>**Comparator:** Tacrolimus 0.1% ointment twice daily (n=186)<br>**Comparator:** Tacrolimus 0.03% ointment twice daily (arm not included in Broeders 2016 review) (n=189)	**Severity:** moderate to severe<br>**Age:** children 2 to 15 years old<br>**Sample size:** 560participants	**Skin burning**<br>**Hydrocortisone:** 13/185 participants (7%)<br>**Tacrolimus 0.1%:** 38/186 participants (20%)<br>*(Difference between groups: p=0.004* [a]*)*<br>**Pruritus**<br>**Hydrocortisone:** 14/185 participants (7.6%)<br>**Tacrolimus 0.1%:** 21/186 participants (11%)<br>*(Difference between groups: p=0.22* [a]*)*<br>**Skin infection**<br>**Hydrocortisone:** 4/185 participants (2.2%)<br>**Tacrolimus 0.1%:** 4/186 participants (2.2%)<br>*(Difference between groups: p=0.99* [a]*)*<br>**Erythema at application site**<br>**Hydrocortisone:** 3/185 participants (1.6%)<br>**Tacrolimus 0.1%:** 1/186 participants (0.5%)<br>**Tacrolimus 0.03%:** 4/189 participants (2.1%)<br>*(Difference between groups, hydrocortisone vs tacrolimus 0.1%: P=0.34* [a]*)*<br>*(Difference between groups, hydrocortisone vs tacrolimus 0.03%: P=0.72* [a]*)*		**Severe adverse events**<br>**Hydrocortisone:** 2/185 participants (1.1%)<br>**Tacrolimus 0.1%:** 1/186 participants (0.5%)<br>*(Difference between groups: (p=0.57* [a]*)*<br>**Adverse events requiring discontinuation**<br>**Hydrocortisone:** 4/185 participants (2.2%)<br>**Tacrolimus 0.1%:** 3/186 participants (1.6%)<br>*(Difference between groups: p=0.70* [a]*)*

For peer review only

Reitamo 2004 [72]<br><br>***(Broeders 2016 [49])***	RCT<br><br>*(3 weeks treatment)*<br><br>*(Jadad score 3/5 – risk from sequence generation and allocation concealment [49])*<br><br>*(Cochrane risk of bias tool: unclear risk of selection bias. Unclear risk from blinding. [3])*<br><br>*(Jadad scale: 4/5, [55])*<br><br>*(Cochrane risk of bias tool: Unclear risk of selection bias (allocation concealment). Low risk of selection bias (random sequence generation), performance, detection, attrition, reporting and other biases. [50])*<br><br>*Cochrane risk of bias tool: low risk of selection, performance, attrition, reporting and other bias. Unclear risk from blinding outcome assessors [52]).*	**Intervention:** Hydrocortisone acetate 1% twice daily (n=207)<br><br>**Comparator:** Tacrolimus 0.03% twice daily (n=210)	**Severity:** moderate to severe<br><br>**Age:** children 2 to 15 years old<br><br>**Sample size:** 417 participants	**<u>Skin burning</u>**<br>**Hydrocortisone:** 30/207 participants (15%)<br>**Tacrolimus:** 50/210 participants (24%)<br>*(Difference between groups: p=0.02 [a])*<br><br>**<u>Pruritus</u>**<br>**Hydrocortisone:** 33/207 participants (16%)<br>**Tacrolimus:** 45/210 participants (21%)<br>*(Difference between groups: p=0.15 [a])*<br><br>**<u>Skin infection</u>**<br>**Hydrocortisone:** 6/207 participants (2.9%)<br>**Tacrolimus:** 6/210 participants (2.9%)<br>*(Difference between groups: p=0.98 [a])*		**<u>Severe adverse events</u>**<br>**Hydrocortisone:** 3/207 participants (1.4%)<br>**Tacrolimus:** 3/210 participants (1.4%)<br>*(Difference between groups: p=0.99 [a])*<br><br>**<u>Adverse events requiring discontinuation</u>**<br>**Hydrocortisone:** 6/207 participants (2.9%)<br>**Tacrolimus:** 8/210 participants (3.8%)<br>*(Difference between groups: p=0.61 [a])*
**Potency of topical corticosteroids unknown**						
Gutgesell 1998 [73]<br><br>(abstract only)<br><br>***(Penaloza Hidalgo 2004 [61])***	Within-participant RCT<br><br>*(3 weeks treatment)*<br><br>*(Cochrane risk of bias tool: randomisation and allocation concealment method inadequate, unclear if blinded ,ITT analysis used [61])*	**Intervention:** Topical corticosteroids on one side of the body, twice daily<br><br>**Comparator:** Tacrolimus 0.1% on one side of the body, twice daily	**Severity:** severe<br><br>**Age:** adults (22 to 36 years)<br><br>**Sample size:** 7 participants	**<u>Skin burning</u>**<br>**Topical corticosteroids:** 0/7 (0%)<br>**Tacrolimus:** 2/7 participants (29%)		
Arellano 2007 [74]<br><br>***(Ashcroft 2007 [56]****; Cury Martins 2015 [50]*	Nested case-control<br><br>*(Duration not specified in the review)*	**Intervention:** Topical corticosteroids at different potencies<br><br>**Comparator:** pimecrolimus or tacrolimus	**Severity:** not specified in the review<br><br>**Age:** not specified in the review		**<u>Lymphoma</u>**<br>No increased risk of lymphoma with TCI or TCS when compared against controls.<br>***Super potent TCS:*** OR 1.2, 95% CI 0.8 to 1.8	

For peer review only

	Risk of bias not assessed in any of the included systematic reviews.	**Comparator:** controls (not specified in the review)	**Sample size:** 294 cases/293,000 controls		***Low potency TCS:*** *OR* 1.1, 95%CI 0.7 to 1.6<br>***Pimecrolimus:*** OR 0.8, 95%CI 0.4 to 1.6<br>***Tacrolimus*** OR 0.8, 95% CI 0.4 to 1.7	
Arellano 2009 [75]<br>(Cury Martins 2015 [50])	Cohort<br>*(followed up between 1992 to 2006)*<br>*Risk of bias not assessed in any of the included systematic reviews.*	**Intervention:** Topical corticosteroids at different potencies<br>**Comparator:** pimecrolimus or tacrolimus	**Severity:** not specified in the review<br>**Age:** not specified in the review<br>**Sample size:** > 3,000,000		**Lymphoma**<br>Increased risk with topical corticosteroids (related to potency) but no numerical data given. Insufficient data to assess TCI-related risks.	
Schneeweiss 2009 [76]<br>***(Cury Martins 2015 [50])***	Cohort<br>*(followed up between the years of 2002 to 2006)*<br>*Risk of bias not assessed in any of the included systematic reviews.*	**Intervention: mid to potent** topical corticosteroids (n=1,043,025)<br>**Comparator:** pimecrolimus (n=118,863) or tacrolimus (n=38,757)<br>(also a comparison with untreated dermatitis (n=118,825) and general population (n=118,863) .)	**Severity:** not specified<br>**Age:** median 1.3 years<br>**Sample size:** 1,438,333 participants		**Lymphoma**<br>Very small non-significant increased risk in TCI and TCS patients when compared with the general population, but with similar risks between the treatment groups	
Reitamo 2000 [77]<br>***(Cury Martins 2015 [50])***	Open label, single group<br>*(6 to 12 months of treatment)*<br>*Risk of bias not assessed in any of the included systematic reviews.*	**Intervention:** No steroids (except prior to treatment)<br>**Comparator:** Tacrolimus 0.1%	**Severity:** not specified in the review<br>**Age:** adults<br>**Sample size:** 316 participants	**Skin thinning**<br>One participant had skin thinning when using TCS prior to treatment with tacrolimus – but this ameliorated after 6 months of treatment with tacrolimus.		
**Is there any difference in safety of topical corticosteroids of different potencies?**						
**Study ID *(Systematic review*)***	**Study design and study duration *(Quality assessment)***	**Intervention and comparator**	**Participants**	**Cutaneous adverse events**	**Systemic adverse events**	**Unspecified adverse events**
**Potent topical corticosteroid versus moderate potency topical corticosteroid**						
Ulrich 1991 [78]<br>***(Hoare 2000 [18])***	RCT<br>*(2 weeks treatment)*<br>*(Moher 1995 quality checklist: method and*	**Intervention:** 0.05% halomethasone cream, twice daily (Assume potent)	**Severity:** not specified in the review			No adverse events

For peer review only

	concealment of randomisation unclear, concerns over subgroup analysis [18])	**Comparator:** 0.25% prednicarbate cream, twice daily (moderate potency)	**Age:** not specified in the review<br>**Sample size:** 165 participants			
Smitt 1993 [79]<br>***(Callen 2007 [21])***	RCT<br>*(3 weeks treatment)*<br>*Risk of bias not assessed in any of the included systematic reviews.*	**Intervention:** Trimaconiolone acetonide 0.1%, twice daily (potent)<br>**Comparator:** Alclomethasone cream, twice daily (moderate)	**Severity:** not specified in the review<br>**Age:** children 1 to 15 years<br>**Sample size:** 40 participants		<u>**HPA axis suppression**</u><br>There was suppression after 2 weeks, but no further after 3 (no further details).	
**Potent topical corticosteroid versus mild potency topical corticosteroid**						
Lebrun-Vignes 2000 [80]<br>***(Nankervis 2017 [3])***	RCT<br>*(15 days treatment, 30 days follow up)*<br>*(Cochrane risk of bias tool: unclear risk of selection bias and unclear risk from blinding. [3])*	**Intervention:** Micronized desonide cream 0.1% (mild potency) 1 to 5 days twice daily (in hospital), days 6 and 7 once daily, then alternate days until day 15 (n=15)<br>**Comparator:** Betamethasone dipropionate cream 0.05% (potent) 1 to 5 days twice daily (in hospital), days 6 and 7 once daily, then alternate days until day 15 (n=14)	**Severity:** severe<br>**Age:** children ≤ 8 years<br>**Sample size:** 29 participants			There were no adverse events in either group
Prado de Oliveira 2002 [81]<br>***(Nankervis 2017 [3])***	RCT<br>*(Up to 42 days treatment)*<br>*(Cochrane risk of bias tool: unclear risk of selection bias and unclear risk from blinding. [3])*	**Intervention:** Mometasone furoate 0.1% once daily after a bath (N=13) (potent)<br>**Comparator:** Desonide cream 0.05% once a day after a bath (N=12) (mild potency)	**Severity:** not specified in the review<br>**Age:** children 2 to 12 years<br>**Sample size:** 25 participants	<u>**Signs of mild thinning**</u><br>**Mometasone furoate:** 4/13 participants (31%)<br>**Desonide:** 2/12 participants (17%)<br>*(Difference between groups: p=0.42 [a])*		
Hanifin 1996 [82]<br>***(Callen 2007 [21])***	Matched case control<br>*(3 weeks treatment)*<br>*Risk of bias not assessed in any of the included systematic reviews.*	**Intervention:** Mometasone cream (potent)<br>**Comparator:** Hydrocortisone cream (mild potency)	**Severity:** not specified in the review<br>**Age:** children 6 months to 2 years<br>**Sample size:** 62 participants		<u>**HPA axis suppression**</u><br>**Mometasone:** 1 abnormal cotrosyn simulation test	

For peer review only

Kirkup 2003 [83] (trial a)<br>***(Tang 2014 [84]; Siegfried 2016 [67])***<br>Most safety data presented was combined with Kirkup 2003 (trial b) (see same potency section below)	RCT<br>*(16 weeks: twice daily for 2-4 weeks until stabilised then 'as required' for 12 weeks)*<br>*(Cochrane risk of bias tool: unclear risk of selection bias, unclear risk from blinding. [3])*	**Intervention:** Fluticasone propionate 0.005% ointment (potent), twice daily for 2-4 weeks until stabilised then 'as required' for 12 weeks (n=70)<br>**Comparator:** Hydrocortisone 1% cream (mild potency), twice daily for 2-4 weeks until stabilised then 'as required' for 12 weeks (n=67)	**Severity: moderate**<br>**Age:** children (age 2-14 years old)<br>**Sample size (maintenance phase):** 137 participants	**Ringworm and folliculitis**<br>1 participant but not clear which group<br>**Kirkup 2003a and b: Bacterial infection**<br>**Fluticasone:** 1/136 participants (0.7%)<br>**Hydrocortisone:** 3/129 participants (2%)<br>*(Difference between groups: p = 0.32 [a])*<br>**Kirkup 2003a and b: Fungal infection**<br>**Fluticasone:** 1/136 participants (0.7%)<br>**Hydrocortisone:** 0/129 (0%)<br>*(Difference between groups: p = 0.52 [a])*	**Kirkup 2003a and b viral infection**<br>**Fluticasone:** 5/136 participants (4%)<br>**Hydrocortisone:** 5/129 participants (4%)<br>*(Difference between groups: p = 0.93 [a])*<br>**Kirkup 2003a and b: Respiratory tract infection**<br>**Fluticasone:** 8/136 participants (6%)<br>**Hydrocortisone:** 5/129 participants (4%)<br>*(Difference between groups: p = 0.45 [a])*	**Kirkup 2003a and b: Discontinuation due to adverse events**<br>**Fluticasone:** 1/136 participants (0.7%)<br>**Hydrocortisone:** 1/129 participants (0.7%)<br>*(Difference between groups: p = 0.97 [a])*
**Moderate potency topical corticosteroid versus mild potency topical corticosteroid**						
Kuokkanen 1987 [85]<br>***(Hoare 2000 [18])***	RCT, within participant<br>*(3 weeks treatment)*<br>*(Moher 1995 quality checklist: method and concealment of randomisation unclear, double blinded study, three dropouts/ withdrawals, no ITT, [18])*	**Intervention:** Alclometasone dipropionate 0.05% twice daily (moderate potency)<br>**Comparator:** Hydrocortisone 1% twice daily (mild potency)	**Severity:** not specified in the review<br>**Age:** children<br>**Sample size:** 37 participants	No evidence of skin thinning		
**Various potencies**						
Ellison 2000 [86]<br>***(Callen 2007 [21]; Eichenfield 2014 [87])***	Observational study<br>*(Duration 0.7 to 18.7 years)*<br>*Risk of bias not assessed in any of the included systematic reviews.*	**Intervention:** Mild, moderate, potent topical corticosteroids	**Severity:** Disease severity score 5-8<br>**Age:** children and adolescents (0.7 to 18.7 years)<br>**Sample size:** 35 participants		**HPA axis suppression**<br>**Mild potency topical corticosteroids:** no change in plasma cortisol levels<br>**Potent topical corticosteroids:** suppression in 4/4 (100%) patients	

For peer review only

For peer review only - http://bmjopen.bmj.com/site/about/guidelines.xhtml

Kristmundsdottir 1987 [88]<br>***(Eichenfield 2014 [87])***	Observational study<br>*(Duration not specified in the review)*<br>*Risk of bias not assessed in any of the included systematic reviews.*	**Intervention:** Different potencies of topical corticosteroids	**Severity:** not specified in the review<br>**Age:** not specified in the review<br>**Sample size:** not specified in the review		Review authors reported "Also concerns for negative effects on linear growth, although reports have given mixed conclusions"	
Patel 1997 [89]<br>***(Eichenfield 2014 [87])***	Observational study<br>*(Duration not specified in the review)*<br>*Risk of bias not assessed in any of the included systematic reviews.*	**Intervention:** Four different potency topical corticosteroids	**Severity:** not specified in the review<br>**Age:** not specified in the review<br>**Sample size:** not specified in the review		Review authors reported "Also concerns for negative effects on linear growth, although reports have given mixed conclusions"	
Patel 1998 [90]<br>***(Eichenfield 2014 [87])***	Observational study<br>*(Duration not specified in the review)*<br>*Risk of bias not assessed in any of the included systematic reviews.*	**Intervention:** Different potencies of topical corticosteroids	**Severity:** not specified in the review<br>**Age:** not specified in the review<br>**Sample size:** not specified in the review		Review authors reported "Also concerns for negative effects on linear growth, although reports have given mixed conclusions"	
**Is there any difference in safety between topical corticosteroids of the same potency?**						
**Study ID *(Systematic review*)***	**Study design and study duration *(Quality assessment)***	**Intervention and comparator**	**Participants**	**Cutaneous adverse events**	**Systemic adverse events**	**Unspecified adverse events**
**Potent topical corticosteroid versus another potent topical corticosteroid**						
Kirkup 2003 [83] (trial b)<br>***(Tang 2014 [84]; Siegfried 2016 [67])***<br>Most safety data presented was combined with Kirkup 2003	RCT<br>*(16 weeks: twice daily for 2-4 weeks until stabilised then intermittently for 12 weeks)*<br>*(Cochrane risk of bias tool: unclear risk of selection bias, unclear risk from blinding. [3])*	**Intervention:** Fluticasone propionate 0.005% ointment, twice daily for 2-4 weeks until stabilised then intermittently for 12 weeks (n=66)<br>**Comparator:** Hydrocortisone butyrate 0.1% cream (potent), twice daily for 2-4 weeks until	**Severity:** moderate<br>**Age:** children (age 2-14 years old)<br>**Sample size:** n=128	**Ringworm and folliculitis**<br>None reported<br>**Kirkup 2003a and b: Bacterial infection**<br>**Fluticasone:** 1/136 participants (0.7%)<br>**Hydrocortisone:** 3/129 participants (2%)	**Kirkup 2003a and b viral infection**<br>**Fluticasone:** 5/136 participants (4%)<br>**Hydrocortisone:** 5/129 participants (4%)<br>*(Difference between groups: p = 0.93 [a])*<br>**Kirkup 2003a and b: Respiratory tract infection**	**Kirkup 2003a and b: Discontinuation due to adverse events**<br>**Fluticasone:** 1/136 participants (0.7%)<br>**Hydrocortisone:** 1/129 participants (0.7%)<br>*(Difference between groups: p = 0.97 [a])*

For peer review only

(trial a) (see different potency section above)		stabilised then intermittently for 12 weeks (n=62)		*(Difference between groups: p = 0.32 [a])*<br>**Kirkup 2003a and b: Fungal infection**<br>**Fluticasone:** 1/136 participants (0.7%)<br>**Hydrocortisone:** 0/129 (0%)<br>*(Difference between groups: p = 0.52 [a])*	**Fluticasone:** 8/136 participants (6%)<br>**Hydrocortisone**: 5/129 participants (4%)<br>*(Difference between groups: p = 0.45 [a])*	
**Moderate potency topical corticosteroid versus another moderate potency topical corticosteroid**						
Aliaga 1994 [91]<br>***(De Tiedra 1997 [30])***	RCT<br>*(21 days treatment)*<br>*Risk of bias not assessed in any of the included systematic reviews.*	**Intervention:** Prednicarbate ointment 0.25%, twice daily (moderate potency) (n=36)<br>**Comparator:** Flucortin ointment 0.75%, twice daily (assumed moderate potency) (n=31)	**Severity:** Disease duration – mean 7.7 years (range 0.1 to 31).<br>**Age:** adults 18-74 years (mean 33.6)<br>**Sample size:** 67 participants			**Adverse reactions**<br>**Prednicarbate:** 0/36 patients (0%)<br>**Flucortin:** 2/31 patients (6.5%)<br>*(Difference between groups: p = 0.16 [a])*
**Mild potency topical corticosteroid versus another mild potency topical corticosteroid**						
Lucky 1997 [92]<br>***(Callen 2007 [21]; Hoare 2000 [18]; Wood Heickman 2018 [9])***	RCT<br>*(4 weeks treatment)*<br>*(Moher 1995 quality checklist: method and concealment of randomisation unclear, open label, five dropouts, no ITT [18])*	**Intervention:** Desonide 0.05% ointment, twice per day (mild potency)<br>**Comparator:** Hydrocortisone 2.5% ointment, twice per day (mild potency)	**Severity:** not specified in the review<br>**Age:** children (mean or median is 4.7 years)<br>**Sample size:** 20 participants		**HPA axis suppression**<br>Normal in both groups (measured using ACTH stimulation testing, measuring serum cortisol levels)	
Jorizzo 1995 [93]<br>***(Siegfried 2016 [67]; Froeschl 2007 [94])***	RCT<br>*(25 weeks: 5 weeks of treatment, 20 weeks follow up)*<br>*(Moher 1995 quality checklist: method and concealment of randomisation unclear, investigator blind, two dropouts/withdrawals, no ITT [18])*	**Intervention:** 0.05% desonide twice daily (n=16) (mild potency)<br>**Comparator:** 1% hydrocortisone ointment twice daily (n=20) (mild potency)	**Severity:** mild to moderate<br>**Age:** children 5 years and under<br>**Sample size:** 36 participants	**Skin thinning**<br>No cases - measured by a magnifying lamp		

For peer review only - http://bmjopen.bmj.com/site/about/guidelines.xhtml

How safe are topical corticosteroids compared to Chinese herbal medicine?						
**Study ID *(Systematic review*)***	**Study design and study duration *(Quality assessment)***	**Intervention and comparator**	**Participants**	**Cutaneous adverse events**	**Systemic adverse events**	**Unspecified adverse events**
**Very potent topical corticosteroids**						
Huang 2010 [95] ***(Gu 2013 [96]; Gu 2014 [97])***	RCT *(2 weeks treatment, followed up for 12 weeks after)* *(Cochrane risk of bias tool: low risk of selection bias (random sequence generation), and other biases. Unclear risk of selection (allocation concealment), detection and attrition bias. High risk of performance and reporting bias. [96, 97]*	**Intervention:** Clobetasol propionate ointment, 3 times daily (n=97) **Comparator:** Chushi Zhiyang ointment, 3 times daily (n=98)	**Severity:** not specified in the review **Age:** children and adults, 3 months to 22 years **Sample size:** 195 participants	<u>**Cutaneous adverse events**</u> **Clobetasol:** 5/97 participants (5%) **Chinese herbal medicine:** 0/98 participants (0%) *(Difference between groups: p=0.10 [a, e])* The five events were pigmentation (unclear if hyper- or hypo-)		
**Potent topical corticosteroids**						
Chen 2011 [98] ***(Gu 2013 [96]; Gu 2014 [97])***	RCT *(2 weeks treatment)* *(Cochrane risk of bias tool: unclear risk of selection, detection, attrition, reporting and other bias. High risk of performance bias [96, 97])*	**Intervention:** Mometasone furoate cream, once daily (n=50) **Comparator:** Huanglian Qingdai ointment, 2 to 3 times daily (n=50)	**Severity:** not specified in the review **Age:** children 58 days to 2 years **Sample size:** 100 participants	<u>**Cutaneous adverse events**</u> **Mometasone:** 6/50 participants (12%) **Chinese herbal medicine:** 0/50 participants (0%) *(Difference between groups: p=0.08 [a, f])* Minor adverse events such as burning, dryness and scaling of the skin were reported in the TCS groups		
Dong 2012 [99] ***(Gu 2014 [97])***	RCT *(2 weeks treatment)* *(Cochrane risk of bias tool: unclear risk of selection, detection, attrition, and reporting bias. High risk of*	**Intervention:** Hydrocortisone butyrate cream, twice daily (n=47) **Comparator:** Jingfang mixture solution, twice daily (n=48)	**Severity:** not specified in the review **Age:** children 0.5 to 5.5 years **Sample size:** 95 participants	Minor adverse events such as burning, dryness and scaling of the skin were reported in the TCS groups. *(No numerical data provided in the review)*		

	performance bias. Low risk of other biases. [97]*)*					
Xu 2012 [100]<br>***(Gu 2014*** [97]***)***	RCT<br>*(2 weeks treatment)*<br>*(Cochrane risk of bias tool: unclear risk of selection, detection, attrition, and reporting bias. High risk of performance and other biases.* [97]*)*	**Intervention:** Triamcinolone acetonide acetate cream, twice daily (n=51)<br>**Comparator:** Kouqiang Xiaoyan powder, twice daily (n=53)	**Severity:** not specified in the review<br>**Age:** children 35 days to 2 years<br>**Sample size:** 104 participants			No adverse events in either group
**How safe is more frequent topical corticosteroid application compared with once daily application?**						
**Study ID *(Systematic review*)***	**Study design and study duration *(Quality assessment)***	**Intervention and comparator**	**Participants**	**Cutaneous adverse events**	**Systemic adverse events**	**Unspecified adverse events**
**Very potent topical corticosteroid**						
Schlessinger 2006 [101]<br>***(Nankervis 2017*** [3]***; Wood Heickman 2018*** [9]***)***	Open label RCT<br>*(2 weeks treatment)*<br>*(Cochrane risk of bias tool: unclear risk of selection bias, high risk from no blinding.* [3]*)*	**Intervention:** fluocinonide cream 0.1% applied once daily (n=63)<br>**Comparator:** fluocinonide cream 0.1% applied twice daily (n=63)	**Severity:** not specified in the review<br>**Age:** children, aged 12 to <18 years (cohort 1); 6 to <12 years (cohort 2); 2 to <6 years (cohort 3); and 3 months to <2 years (cohort 4).<br>**Sample size:** 126 participants		<u>**HPA axis suppression**</u><br>**Once daily**: 0/63 (0%)<br>**Twice daily**: 3/63 (4.8%)<br>*(Difference between groups: P=0.19*[a]*)*<br>(measured using ACTH stimulation testing, measuring serum cortisol levels)<br>After TCS discontinuation, children with biochemical adrenal insufficiency had complete resolution at retesting.	
**Potent topical corticosteroids**						
Bleehen 1995 [102]<br>***(Green 2004*** [103]***)***	RCT<br>*(4 weeks treatment))*<br>*(Quality using NHS CRD criteria: method for randomisation/allocation concealment unknown, adequate blinding, and ITT used.* [103]*)*<br>*(Moher 1995 quality checklist: method and*	**Intervention:** Fluticasone propionate 0.05% cream once daily (plus vehicle once daily for blinding) (n=137)<br>**Comparator:** Fluticasone propionate 0.05% cream twice daily (n=133)	**Severity:** at least moderate severity<br>**Age:** children and adults<br>**Sample size:** 270 participants			<u>**Number of events possibility, probably or almost certainly related to study medication**</u><br>**Once daily:** 26 events<br>**Twice daily**: 24 events<br>(most were skin disorders)

For review only

	concealment of randomisation unclear, Probably investigator blinded but unclear, ITT analysis, [18])					
GSK report 1995 [104]<br><br>***(Green 2004 [103])***	RCT<br><br>*(4 weeks treatment)*<br><br>*(Quality using NHS CRD criteria: adequate method of randomisation /allocation concealment, adequate blinding, and ITT used. [103])*	**Intervention:** Fluticasone propionate 0.005% ointment once daily and placebo only daily (n=123)<br><br>**Comparator:** Fluticasone propionate 0.005% ointment twice daily (n=122)	**Severity:** at least moderate severity<br><br>**Age:** children and adults<br><br>**Sample size:** 245 participants			**Number of adverse events possibly related to medication**<br>**Once daily:** 6 events<br>**Twice daily**: 8 events<br><br>**Number of adverse events probably related to medication**<br>**Once daily:** 9 events<br>**Twice daily:** 3 events<br><br>**Number of adverse events almost certain related to medication**<br>**Once daily:** 6 events<br>**Twice daily:** 3 events<br><br>(Mainly included skin related disorders including exacerbation of eczema, pruritus and redness of skin)
Koopmans 1995 [105]<br><br>***(Green 2004 [103])***	RCT<br><br>*(4 weeks treatment))*<br><br>*(Quality using NHS CRD criteria: method for randomisation /allocation concealment unknown, partial blinding, and no ITT used. [103])*<br><br>*(Moher 1995 quality checklist: method and concealment of randomisation unclear, double blinded, one dropout, no ITT analysis, [18])*	**Intervention:** Locoid lipocream (0.1% hydrocortisone 17-butyrate) once daily and locobase once daily (n=75)<br><br>**Comparator:** Locoid lipocream twice daily (n=75)	**Severity:** not specified in the review<br><br>**Age:** children aged over 12 years and adults<br><br>**Sample size:** 150 participants	**Folliculitis in all skin areas after 1 week of treatment – treatment stopped**<br>**Once daily:** 1/75 participants (1.3%)<br>**Twice daily:** 0/75 participants (0%)<br>*(Difference between groups: p = 0.50 [a])*<br><br>**Folliculitis - treatment continued**<br>**Once daily:** 0/75 participants (0%)<br>**Twice daily:** 4/75 participants (5.3%)<br>*(Difference between groups: p = 0.14 [a])*<br><br>**Burning, itching and stinging sensations – treatment continued**<br>**Once daily:** 3/75 participants (4%)		

For peer review only

				**Twice daily:** 0/75 participants (0%)<br>*(Difference between groups: p = 0.20 [a])*		
Tharp 1996 [106]<br><br>***(Green 2004 [103])***	RCT<br><br>*(4 weeks treatment)*<br><br>*(Quality using NHS CRD criteria: method for randomisation /allocation concealment unknown, adequate blinding, and no ITT used. [103])*	**Intervention:** Fluticasone propionate cream 0.05% once daily and vehicle once daily (n=77)<br><br>**Comparator:** Fluticasone propionate cream 0.05% twice daily (n=77)	**Severity:** moderate to severe<br><br>**Age:** children over 12 years and adults<br><br>**Sample size:** 154 participants	**Burning**<br>**Once daily**: 2/77 participants (3%)<br>**Twice daily:** 0/77 participants (0%)<br>*(Difference between groups: p = 0.30 [a])*<br><br>**Dryness**<br>**Once daily:** 2/77 participants (3%)<br>**Twice daily:** 0/77 participants (0%)<br>*(Difference between groups: p = 0.30 [a])*<br><br>**Pruritus**<br>**Once daily:** 0/77 participants (0%)<br>**Twice daily**: 1/77 participants (1%)<br>*(Difference between groups: p = 0.50 [a])*<br><br>**Erythema**<br>**Once daily:** 0/77 participants (0%)<br>**Twice daily:** 0/77 participants(0%)<br><br>**Stinging**<br>**Once daily:** 0/77 participants (0%)<br>**Twice daily:** 1/77 participants (1%)<br>*(Difference between groups: p = 0.50 [a])*<br><br>**Irritation**<br>**Once daily:** 0/77 participants (0%)<br>**Twice daily:** 1/77 participants (1%)		None of adverse events were serious or unexpected

For peer review only

				(Difference between groups: p = 0.50 [a])		
Hoybye 1991 [107]<br>***(Green 2004 [103])***	RCT<br>*((3 weeks treatment)*<br>*(Quality using NHS CRD criteria: method for randomisation /allocation concealment unknown, partial or inadequate blinding, and no ITT used. [103])*<br>*(Moher 1995 quality checklist: method and concealment of randomisation unclear, single blind, ten dropouts/withdrawals, no ITT analysis, [18])*	**Intervention:** Mometasone furoate in fatty cream base (Elocon) once daily (n=49)<br>**Comparator:** Hydrocortisone 17-butyrate in fatty cream base (Locoid) twice daily (n=45)	**Severity:** severity score at least 4.5/9<br>**Age:** adults (age 18 to 70)<br>**Sample size:** 94 participants	**<u>Treatment related side effects</u>**<br>Were only a few and similar in both groups. They included stinging, burning, itching, dryness, acne, folliculitis, and hair growth.<br>**<u>Skin thinning</u>**<br>No evidence		
Berth-Jones 2003 [108]<br>***(Green 2004 [103])***<br>(This study is also included in the "Topical corticosteroids used proactively to prevent flares", as there is a second phase of the study when participants who have gained control of eczema are randomised to proactive treatment with topical corticosteroid or vehicle. This section only includes safety	RCT (four arms)<br>*(4 weeks treatment)*<br>*(Quality using NHS CRD criteria: adequate randomisation /allocation concealment, partial blinding, and ITT used. [103])*	**Intervention:** Fluticasone propionate cream 0.05% once daily<br>N=95<br>**Intervention:** Fluticasone propionate ointment 0.005% once daily<br>N=100<br>**Comparator:** Fluticasone propionate cream 0.05% twice daily<br>N=91<br>**Comparator:** Fluticasone propionate ointment 0.005% twice daily<br>N=90	**Severity:** moderate to severe<br>**Age:** children and adults (12-65 years)<br>**Sample size:** 376 participants	**<u>Telangiectasia</u>**<br>**Once daily cream**: 0/95 participants (0%)<br>**Twice daily cream:** 1/91 participants (1%)<br>*(Difference between groups: p = 0.48 [a])*<br>**Once daily ointment:** 1/100 participants (1%)<br>**Twice daily ointment:** 0/90 participants (0%)<br>*(Difference between groups: p = 0.54 [a])*<br>**<u>Striae</u>**<br>**Once daily cream:** 0/95 participants (0%)<br>**Twice daily cream:** 0/91 participants (0%)<br>*(Difference between groups: n/a)*<br>**Once daily ointment:** 1/100 participants (1%)<br>**Twice daily ointment**: 0/90 participants (0%)		

For peer review only

data from the induction of remission phase).				*(Difference between groups: p = 0.54 [a])*<br>For the three events listed above: two of these patients had a previous history of skin changes, and therefore only one report was newly observed (group not specified in the review).		
Marchesi 1994 [109]<br>***(Green 2004 [103])***	RCT<br>*(3 weeks treatment))*<br>*(Quality using NHS CRD criteria: method for randomisation /allocation concealment unknown, partial blinding, and no ITT used. [103])*<br>*(Moher 1995 quality checklist: method and concealment of randomisation unclear, third-party blind evaluator, no dropouts/withdrawals [18])*	**Intervention:** Mometasone furoate ointment 0.1% once daily (n=30)<br>**Comparator:** Betamethasone dipropionate ointment 0.05% twice daily (n=30)	**Severity:** at least moderate severity<br>**Age:** adults<br>**Sample size:** 60 participants	**Telangiectasia of mild severity in last 2 weeks**<br>**Once daily:** 4/30 participants (13.3%)<br>**Twice daily:** 5/30 participants (16.7%)<br>*(Difference between groups: p = 0.72 [a])*<br>**Possible skin thinning (*"Loss of skin marks and reduced elasticity"*)**<br>**Once daily:** 0/30 participants (0%)<br>**Twice daily:** 1/30 participants (3.3%)<br>*(Difference between groups: p = 0.50 [a])*<br>**Local application site reactions**<br>Did not occur	**Systemic reactions**<br>None – all patients checked for blood test and value varied within a very narrow range.	
**Moderate potency topical corticosteroids**						
Richelli 1990 [110]<br>***(Green 2004 [103])***	RCT<br>(one week treatment<br>*(Quality using NHS CRD criteria: method for randomisation /allocation concealment unknown, inadequate blinding, and no ITT used. [103])*<br>*(Moher 1995 quality checklist: method and concealment of*	**Intervention:** Clobetasone 17-butyrate 0.05% lotion once daily at 9pm (n=9)<br>**Comparator:** Clobetasone 17-butyrate 0.05% lotion twice daily at 8am and 3pm (n=13)<br>**Comparator:** Clobetasone 17-butyrate 0.05% lotion twice daily at 3pm and 8pm (n=8)	**Severity:** not specified in the review<br>**Age:** children<br>**Sample size:** 30 participants		**HPA axis suppression**<br>No significant difference in serum cortisol and ACTH levels before and after TCS administration in any of the three groups, or any differences between groups	Adverse effects not reported

For peer review only - http://bmjopen.bmj.com/site/about/guidelines.xhtml

	randomisation unclear, blinding unclear, ITT unclear [18])					
**How safe are topical corticosteroids when used proactively to prevent flares ("weekend therapy")?**						
**Study ID *(Systematic review*)***	**Study design and study duration *(Quality assessment)***	**Intervention and comparator**	**Participants**	**Cutaneous adverse events**	**Systemic adverse events**	**Unspecified adverse events**
**Potent topical corticosteroids versus vehicle**						
Berth-Jones 2003 [108]<br>***(Schmitt 2011 [111]; Tang 2014 [84])***<br>(This study is also included under the comparison "Topical corticosteroids applied once a day compared with more frequent application" – where the induction of remission part of the study is included).	RCT<br>*(16 weeks maintenance)*<br>*(Cochrane risk of bias tool: low risk of selection (sequence generation), attrition and other biases, Unclear risk of selection (allocation concealment), bias from blinding and reporting bias. [111])*	**Intervention:** Fluticasone propionate 0.005% ointment on two consecutive days per week, once daily (n=68)<br>**Intervention:** Fluticasone propionate 0.05% cream on two consecutive days per week, once daily (n=70)<br>**Comparator:** Vehicle cream or ointment (n=84)<br>**Comparator:** Vehicle ointment (n=73)	**Severity:** moderate to severe<br>**Age:** 12 to 65 years<br>**Sample size (maintenance phase):** 295 participants	**Skin thinning**<br>No new visual signs observed in either group during maintenance phase		
Glazenburg 2009 [112]<br>***(Schmitt 2011 [111]; Tang 2014 [84])***	RCT<br>*(16 weeks maintenance)*<br>*(Cochrane risk of bias tool: low risk of selection (sequence generation), and attrition bias. Unclear risk of selection (allocation concealment), bias from blinding, reporting and other biases. [111])*	**Intervention:** Fluticasone propionate 0.005% ointment (two consecutive days per week, once daily) (n=39)<br>**Comparator:** Vehicle (n=36)	**Severity:** moderate to severe<br>**Age:** children 4-10 years<br>**Sample size (maintenance phase):** 75 participants	**Skin thinning**<br>No evidence in either group<br>**Adverse events related to treatment (cutaneous)**<br>**Fluticasone:** 2 events (flexural hyperpigmentation, folliculitis, transient telangiectasia) (n=39)<br>**Vehicle:** 1 event (no further details reported) (n=36)<br>*(Difference between groups: p=0.61[a])*	**Adrenal suppression**<br>No evidence in either group (measured by assessment of urinary overnight cortisol/creatinine ratios)<br>**Cancer**<br>No cases in either group	

Hanifin 2002 [113]<br><br>***(Schmitt 2011 [111])***	RCT<br><br>*(20 weeks maintenance)*<br><br>*(Cochrane risk of bias tool: low risk of attrition bias. Unclear risk of selection, bias from blinding and reporting bias. High risk of other biases (noncompliance) [111])*	**Intervention:** Fluticasone propionate 0.05% cream (once daily 4 days per week for 4 weeks, then once daily 2 days per week for 16 weeks) (n=229)<br><br>**Comparator:** Vehicle (n=119)	**Severity:** moderate to severe<br><br>**Age:** children and adults, 3 months to65 years<br><br>**Sample size (maintenance phase):** 348 participants	**Adverse events related to treatment**<br>**Fluticasone:** 1/229 (one case of acne) (0.4%)<br>**Vehicle:** 0/119 (0%)<br>*(Difference between groups: p=0.78[a])*<br><br>**Skin thinning**<br>No evidence (by visual skin assessment)	**Possible adrenal suppression**<br>**Fluticasone:** 2/44 children (4.5%)<br>**Vehicle:** no evidence of adrenal suppression<br>*(measured by cosynthropin stimulation test)*<br><br>**Cancer**<br>No cases	
Van der Meer 1999 [114]<br><br>***(Schmitt 2011 [111])***	RCT<br><br>*(16 weeks maintenance)*<br><br>*(Cochrane risk of bias tool: low risk of attrition, and other biases. Unclear risk of selection, bias from blinding and reporting bias. [111])*<br><br>*((Moher 1995 quality checklist: method and concealment of randomisation unclear, double blinded, 17 withdrawals/dropouts, no ITT, only data up to first relapse analysed, [18])*	**Intervention:** Fluticasone propionate 0.005% ointment (2 consecutive days per week, once daily) (n=23)<br><br>**Comparator:** Vehicle (n=31)	**Severity:** moderate to severe<br><br>**Age:** children and adults, aged 15-50 years<br><br>**Sample size (maintenance phase):** 54 participants	**Skin thinning**<br>No evidence	**Adrenal suppression**<br>No change in geometric mean cortisol levels at baseline and end of maintenance<br><br>**Cancer**<br>No cases	
Peserico 2008 [115]<br><br>***(Schmitt 2011 [111])***	RCT<br><br>*(16 weeks maintenance)*<br><br>*(Cochrane risk of bias tool: high risk of selection bias (sequence generation). Low risk of attrition bias and bias from blinding. Unclear risk of selection (allocation concealment) reporting and other biases. [111])*	**Intervention:** Prednisolone aceponate 0.1% cream (two consecutive days per week, once daily) (n=112)<br><br>**Comparator:** Vehicle (n=108)	**Severity:** IGA≥ moderate<br><br>**Age:** children ≥12 years and adults<br><br>**Sample size (maintenance phase):** 221 participants	**Skin thinning**<br>No evidence	**Cancer**<br>No cases.	**Adverse events related to treatment**<br>Zero in both groups
**How safe are topical corticosteroids used under occlusion?**						

For peer review only

Study ID *(Systematic review*)*	Study design and study duration *(Quality assessment)*	Intervention and comparator	Participants	Cutaneous adverse events	Systemic adverse events	Unspecified adverse events
**Very potent topical corticosteroid**						
Volden 1992 [116] ***(Braham 2010 [117])***	Prospective (observational) *(8-18 days treatment)* *Risk of bias not assessed in any of the included systematic reviews.*	**Intervention:** Dry occlusion with clobetasol propionate lotion under dry occlusion (weekly) (n=48) **Comparator:** No comparator	**Severity:** therapy resistant atopic eczema **Age:** adults **Sample size:** 48 participants	**Mild folliculitis** 2/48 participants (4%) **Skin thinning** None		
**Potent topical corticosteroids**						
Janmohamed 2014 [118] ***(Van Zuuren 2017 [12])***	RCT *(4 weeks treatment)* *(Cochrane risk of bias tool: low risk of selection (sequence generation), attrition, reporting and other biases. Unclear risk of selection (allocation concealment), performance and detection bias. [12])*	**Intervention:** wet wrap therapy with diluted mometasone furoate 0.1% ointment (n=19) **Comparator:** 20% petrolatum in cetomacrogol combined with wet wrap (n=20)	**Severity:** severe **Age:** children 6 months to 10 years (mean age 3.4 years) **Sample size:** 39 participants	**Folliculitis** **Mometasone under wet wrap:** 9/19 (47%) **Emollient under wet wrap:** 2/20 (10%) *(Difference between groups: p = 0.03 [a])* **Severe folliculitis** **Mometasone under wet wrap:** 1/19 (5.2%) **Emollient under wet wrap:** 0/20 (0%) *(Difference between groups: p = 0.47*)* **Secondary infected eczema** **Mometasone under wet wrap:** 0/19 (0%) **Emollient under wet wrap**: 2/20 (10%) *(Difference between groups: p= 0.30 [a])* **Beginning of decubitus** **Mometasone under wet wrap:** 0/19 (%) **Emollient under wet wrap:** 2/20 (10%) *(Difference between groups: p = 0.30[a])* **Decubitus**		

				**Mometasone under wet wrap:** 2/19 (11%)<br>**Emollient under wet wrap:** 1/20 (5%)<br>*(Difference between groups: p = 0.53 [a])*		
Schnopp 2002 [119]<br>***(Braham 2010 [117])***	RCT, within-participant<br>*(5 days treatment)*<br>*(Cochrane risk of bias tool: unclear risk of selection bias, unclear risk from blinding. [3])*	**Intervention:** wet wrap therapy with mometasone furoate 0.1%, twice daily<br>**Comparator:** wet wrap therapy with vehicle	**Severity:** exacerbated atopic eczema<br>**Age:** children aged 2 to 17 years (mean 7.2 years)<br>**Sample size:** 20 participants	**Clinical skin infections**<br>None in either group		
McGowan 2003 [120]<br>***(Devillers 2006 [121])***	Prospective (observational)<br>*(Up to 14 days treatment)*<br>*Risk of bias not assessed in any of the included systematic reviews.*	**Intervention:** wet wrap therapy with diluted beclomethasone dipropionate, once daily (n=8)<br>**Comparator:** No comparator	**Severity:** not specified in the review<br>**Age:** children age 3.3 to 8.8 years<br>**Sample size:** 8 participants		**Short term growth and bone turnover**<br>No significant differences found between outcomes before and during a median treatment period of 12 weeks (range 2-18).<br>*(assessed safety with knemometry and urinary deoxypyridinoline crosslink excretion and early morning serum cortisol).*	
Wolkerstorfer 2000 [122]<br>***(Braham 2010 [117])***	Prospective, side to side (observational)<br>*(1 week treatment)*<br>*Risk of bias not assessed in any of the included systematic reviews.*	**Intervention:** wet wrap therapy with 10-50% dilution fluticasone propionate 0.05% cream (daily)<br>**Comparator:** emollient (only 2 participants) or no comparator	**Severity:** severe<br>**Age:** children 5 months to 13 years<br>**Sample size:** 18 participants	**URI and/or folliculitis**<br>**Fluticasone:** one third of participants<br>**Furunculosis**<br>**Fluticasone:** one case<br>**Generalized folliculitis**<br>One case in both emollient controls<br>**Skin thinning**<br>No cases	**HPA axis suppression**<br>"Nearly all" had decreased cortisol, 3 children were HPA suppressed	
Tang 2000 [123]<br>***(Braham 2010 [117])***	Prospective (observational)<br>*("Few days" treatment)*	**Intervention:** wet wrap therapy with 10% dilution mometasone furoate 0.1% (daily for 2 to 3 hours) (n=10)<br>**Comparator:** No comparator	**Severity:** review only reports 'facial eczema flare'<br>**Age:** children (mean 8.4 years)	**Skin thinning**<br>None<br>**Infections**<br>None		

For peer review only

	Risk of bias not assessed in any of the included systematic reviews.		**Sample size:** 10 participants			
Goodyear 1991 [124]<br>***(Braham 2010 [117])***	Prospective (observational)<br>*(2 to 5 days treatment)*<br>*Risk of bias not assessed in any of the included systematic reviews.*	**Intervention:** wet wrap therapy with 25% dilution betamethasone or hydrocortisone 1%, twice daily (potent or mild potency) (n=30)<br>**Comparator:** No comparator	**Severity:** acute erythrodermic eczema<br>**Age:** children aged 9 months to 2 years (mean 5.5 years)<br>**Sample size:** 30 participants	**Bacterial infections**<br>Some during follow up at home	**HPA axis suppression**<br>Transient low morning cortisol. During the follow up at home some adrenal suppression	
Mallon 1994 [125]<br>***(Braham 2010 [117])***	Prospective (observational)<br>*(up to 5 days treatment)*<br>*Risk of bias not assessed in any of the included systematic reviews.*	**Intervention:** wet wrap therapy with 10% dilution betamethasone 0.1% cream or hydrocortisone 0.5% cream(daily) (potent or mild potency) (n=21)<br>**Comparator:** No comparator	**Severity:** chronic severe eczema<br>**Age:** children aged 4 months to 10 years (5.1 years)<br>**Sample size:** 21 participants	No infections.		
Devillers 2002 [126]<br>***(Braham 2010 [117])***	Retrospective side to side (observational)<br>*(1 week treatment)*<br>*Risk of bias not assessed in any of the included systematic reviews.*	**Intervention:** wet wrap therapy with **d**iluted fluticasone propionate 0.05% (daily re-wet every 2 to3 hours) (n=26)<br>**Comparator:** No comparator	**Severity:** refractory atopic eczema<br>**Age:** children (mean 3 years), adults (mean 30 years)<br>**Sample size:** 26 participants (14 children, 12 adults)	**Infections**<br>38% (n=10) had localized folliculitis, impetigo, pseudomonas, cellulitis, or purulent conjunctitivitis<br>**Skin thinning**<br>One case of striae in a patient taking inhaled steroids.	**HPA axis suppression**<br>Transient low morning cortisol, 12.5% with HPA suppression	
**Moderate potency topical corticosteroids**						
Foelster-Holst 2006 [127]<br>***(Gonzalez-Lopez 2017 [128])***	Within-participant RCT<br>*(48 to 72 hours treatment)*<br>*(Cochrane risk of bias tool: unclear risk of selection and performance bias. High risk of performance bias. Unclear risk of attrition, reporting and other biases. [128])*	**Intervention:** wet wrap therapy with prednicarbate ointment<br>**Comparator:** Prednicarbate ointment	**Severity:** local SCORAD >10, severe<br>**Age:** children and adults, aged 6-63 years<br>**Sample size: 24** participants	Zero adverse events in either group. Did not observe severe cutaneous events.	Did not observe systemic events such as growth retardation or HPA suppression – but these events were not actively investigated.	

For peer review only

	(Cochrane risk of bias tool: unclear risk of selection bias, high risk from no blinding. [3])					
**Mild potency topical corticosteroid**						
Beattie 2004 [129]<br><br>***(Gonzalez-Lopez 2017 [128])***	RCT<br><br>*(2 weeks treatment)*<br><br>*(Cochrane risk of bias tool: low risk of selection, reporting and other biases. High risk of performance and attrition bias. Unclear risk of detection bias. Gonzalez-Lopez 2017)*<br><br>*(Cochrane risk of bias tool: low risk of selection bias (sequence generation), unclear risk of selection bias (allocation concealment), unclear risk from blinding. [3])*	**Intervention:** wet wrap therapy with hydrocortisone 1% twice daily then overnight the second week(n=10)<br><br>**Comparator:** Hydrocortisone 1% twice daily then daily (n=9)	**Severity:** moderate<br><br>**Age:** children < 5 years<br><br>**Sample size:** 19 participants	<u>**Cutaneous adverse events**</u><br>**Wet wrap therapy with hydrocortisone:** 2/10 participants (20%) (2 events were folliculitis, one child withdrew)<br>**Hydrocortisone only:** 0/9 participants (0%)<br>*(Difference between groups: (p=0.31 [a])*<br><br>Did not observe severe cutaneous events.	Did not observe systemic such as growth retardation or HPA suppression – but these events were not actively investigated.	
Hindley 2006 [130]<br><br>***(Gonzalez-Lopez 2017 [128])***	RCT<br><br>*(4 weeks – not clear if treatment given for whole 4 weeks)*<br><br>*(Cochrane risk of bias tool: low risk of selection (random sequence generation) and reporting bias. Unclear risk of selection (allocation concealment), detection and other biases. High risk of performance and attrition bias. [128])*<br><br>*(Cochrane risk of bias tool: unclear risk of*	**Intervention:** wet wrap therapy with hydrocortisone 1% for 24 hours – could be reduced to 12 hours per day after first week (n=28)<br><br>**Comparator:** Hydrocortisone 1% twice day (n=22)	**Severity:** SCORAD >15, moderate to severe<br><br>**Age:** children 3 months to 5 years<br><br>**Sample size:** 50 participants	<u>**Cutaneous adverse events**</u><br>**Wet wrap therapy with hydrocortisone:** 5/28 participants (18%) (five cases of infected eczema)<br>**Hydrocortisone only:** 0/22 participants (0%)<br><br>*(Difference between groups: p = 0.14 [a])*<br><br>Did not observe severe cutaneous events.	Did not observe systemic events such as growth retardation or HPA suppression – but these events were not actively investigated.	

For peer review only

	selection bias, low risk from blinding. [3]*)*					

Footnotes:
*This column refers to the systematic review in which the safety data was extract from. The trial may have also been included in other systematic reviews, but no additional safety data was reported.
Abbreviations: RCT = randomised controlled trial; TCS = topical corticosteroids; TCI = topical calcineurin inhibitors; HPA = hypothalamic pituitary adrenal, WWT = wet wrap therapy; RR = risk ratio; OR: odds ratios; 95% CI = 95% confidence interval; CHM = Chinese herbal medicine; IPA = Investigator's Global Assessment; BSA = Body Surface Area

[a] P value calculated by review authors using RevMan software.
[b] The P value calculated from Fisher's Exact Test was significant: 0.0298 (but in the overview, this study is included in a meta-analysis)
[c] The P value calculated from Fisher's Exact Test was significant: 0.0298 (but in the overview, this study is included in a meta-analysis)
[d] The P value calculated from Fisher's Exact Test was significant: 0.0352 (but in the overview, this study is included in a meta-analysis)
[e] The P value calculated from Fisher's Exact Test was significant: 0.0289 (but in the overview, this study is included in a meta-analysis)
[f] The P value calculated from Fisher's Exact Test was significant: 0.0267 (but in the overview, this study is included in a meta-analysis)

Where studies include "diluted" topical corticosteroids and we aren't sure how this affects the potency, we have put the topical corticosteroids in the potency classification based on the undiluted version.
The terms skin atrophy and skin thinning were both used in the included reviews – for consistently we have used skin thinning throughout.

References

1. Breneman D, Fleischer A, Foley V ea, editors. Clobetasol propionate 0.05% is equivalent as lotion or emollient cream in atopic dermatitis. Presented at the World Congress of Dermatology, 2002; and the European Academy of Dermatology and Venereology, 2003; 2003.
2. Feldman S. Relative efficacy and interchangeability ofvarious clobetasol propionate vehicles in the management of steroid-responsive dermatoses. *Curr Ther Res Clin Exp*. 2005;**66**(3):154-71.
3. Nankervis H, Thomas K, Delamere F, Barbarot S, Rogers N, Williams H. Scoping systematic review of treatments for eczema2016 2016/05/None.
4. Kimball AB, Gold MH, Zib B, Davis MW, Phase CPEFF, Group ICS. Clobetasol propionate emulsion formulation foam 0.05%: review of phase II open-label and phase III randomized controlled trials in steroid-responsive dermatoses in adults and adolescents. *J Am Acad Dermatol*. 2008;**59**(3):448-54. e1.
5. Frangos J, Kimball A. Clobetasol propionate emollient formulation foam in the treatment of corticosteroid-responsive dermatoses. *Expert Opin Pharmacother*. 2008;**9**(11):2001-7.
6. Rosso J, Bhambri S. Daily application of fluocinonide 0.1% cream for the treatment of atopic dermatitis. *J Clin Aesthet Dermatol*. 2009;**2**(9):24-32.
7. Barnes L, Kaya G, Rollason V. Topical Corticosteroid-Induced Skin Atrophy: A Comprehensive Review. *Drug Saf*. 2015;**38**(5):493-509.
8. Olux-e (dobetasol propionate) foam pw. Steiffel Corporation, Palo Alto, CA; [Available from: www.olux-e.com.
9. Wood Heickman L, Davallow Ghajar L, Conaway M, Rogol A. Evaluation of Hypothalamic-Pituitary-Adrenal Axis Suppression following Cutaneous Use of Topical Corticosteroids in Children: A Meta-Analysis. *Horm Res Paediatr*. 2018;**89**(6):389-96.
10. Herz G, Yawalkar S, Weirich E. Evaluation of halometasone ointment in the treatment of paediatric patients with chronic eczematous dermatoses. *J Int Med Res*. 1983;**11 Suppl 1**:21-5.
11. Sugarman J, Parish L. Efficacy of a lipid-based barrier repair formulation in moderate-to-severe pediatric atopic dermatitis. *J Drugs Dermatol*. 2009;**8**(12):1106-11.
12. van Zuuren EJ, Fedorowicz Z, Christensen R, Lavrijsen AP, Arents BW. Emollients and moisturisers for eczema. Cochrane Database Syst Rev [Internet]. 2017; (2). Available from: http://onlinelibrary.wiley.com/doi/10.1002/14651858.CD012119.pub2/abstract.

For peer review only - http://bmjopen.bmj.com/site/about/guidelines.xhtml

13. Griffiths C, Van Leent E, Gilbert M, Traulsen J, Cipamyflline Study Group. Randomized comparison of the type 4 phosphodiesterase inhibitor cipamfylline cream, cream vehicle and hydrocortisone 17-butyrate cream for the treatment of atopic dermatitis. *Br J Dermatol*. 2002;**147**(2):299-307.
14. Wu S, Chen X, Liu B, Wu H, Dong L. Efficacy and safety of 15(R/S)-methyl-lipoxin A4 in topical treatment of infantile eczema. *Br J Dermatol*. 2013;**168**(1):172-8.
15. Eichenfield L, Miller B, Cutivate Lotion Study Group. Two randomized, double-blind, placebo-controlled studies of fluticasone propionate lotion 0.05% for the treatment of atopic dermatitis in subjects from 3 months of age. *J Am Acad Dermatol*. 2006;**54**(4):715-7.
16. Pellanda C, Weber M, Bircher A, Surber C. Low-dose triamcinolone acetonide in the phytocosmetic lichtena reduces inflammation in mild to moderate atopic dermatitis. *Dermatology*. 2005;**211**(4):338-40.
17. Lebwohl M. Efficacy and safety of fluticasone propionate ointment, 0.005%, in the treatment of eczema. *Cutis*. 1996;**57**(Suppl 2):62-8.
18. Hoare C, Li Wan Po A, Williams H. Systematic review of treatments for atopic eczema. *Health Technol Assess*. 2000;**4**(37):1-191.
19. Lebwohl M. A comparison of once-daily application of mometasone furoate 0.1% cream compared with twice-daily hydrocortisone valerate 0.2% cream in pediatric atopic dermatitis patients who failed to respond to hydrocortisone. *Int J Dermatol*. 1999;**38**(8):604-6.
20. Friedlander S, Hebert A, Allen D. Safety of fluticasone propionate cream 0.05% for the treatment of severe and extensive atopic dermatitis in children as young as 3 months. *J Am Acad Dermatol*. 2002;**46**(3):387-93.
21. Callen J, Chamlin S, Eichenfield L, Ellis C, Girardi M, Goldfarb M, et al. A systematic review of the safety of topical therapies for atopic dermatitis. *Br J Dermatol*. 2007;**156**(2):203-21.
22. Eichenfield L, Ellis CN, Fivenson D, Hebert AA, Dromgoole S, Piacquadio D. Evaluation of adrenal suppression of a lipid enhanced, topical emollient cream formulation of hydrocortisone butyrate 0.1% in treating children with atopic dermatitis. *Pediatr Dermatol*. 2007;**24**(1):81-4.
23. Hebert AA, Friedlander SF, Allen DB. Topical fluticasone propionate lotion does not cause HPA axis suppression. *J Pediatr*. 2006;**149**(3):378-82.
24. Abramovits W, Oquendo M. Hydrocortisone butyrate 0.1% cream (proprietary lipid rich cream vehicle) does not significantly suppress hypothalamic-pituitary-adrenal axis and is effective in pediatric patients 3 months and older with extensive atopic dermatitis. *Skinmed*. 2010;**8**(3):150-4.
25. De Belilovsky C, Roo-Rodriguez E, Baudouin C, Menu F, Chadoutaud B, Msika P. Natural peroxisome proliferator-activated receptor-alpha agonist cream demonstrates similar therapeutic response to topical steroids in atopic dermatitis. *J Dermatolog Treat*. 2011;**22**(6):359-65.
26. Rosenthal A. Clocortolone pivalate: a paired comparison clinical trial of a new topical steroid in eczema/atopic dermatitis. *Cutis*. 1980;**25**(1):96-8.
27. Singh S, Mann B. Clinical utility of clocortolone pivalate for the treatment of corticosteroid-responsive skin disorders: A systematic review. *Clin Cosmet Investig Dermatol*. 2012;**5**:61-8.
28. Binder R. Clinical study of clocortolone pivalate in the treatment of eczema/atopic dermatitis. *Curr Ther Res Clin Exp*. 1977;**21**(6):796-801.
29. Nolting S GH, Griinder K. Therapie des atopischen ekzems im kindesalter: ergebnisse einer kontrollierten studie mit den topischen kortikosteroidzubereitungen mometason und prednicarbaL *Der Kinderarzt*. 1991;**22**(10):1690-3.
30. de Tiedra A, Mercadal J, Lozano R. Prednicarbate versus fluocortin for inflammatory dermatoses: A cost-effectiveness study. *PharmacoEconomics*. 1997;**12**(2 Pt 1):193-208.
31. Rampini E. Methylprenisolone aceponate (MPA)* - Use and clinical experience in children. *J Dermatolog Treat*. 1992;**3 (SUPPL. 2)**(Suppl 2 CNO - CN-00421335):27-9.
32. Camacho F, García B, Diaz J, Aguirre A, Arnau C, Garcia J, et al. Double-blind intraindividual comparative trial between prednicarbate versus fluocortolone for the treatment of atopic dermatitis [Ensayo comparativo intraindividual y doble ciego entre prednicarbato y f1uocortolona en el tratamiento de la dermatitis atapica]. Actas Dermo-Sifiliograficas [Internet]. 1996; 87:[59-63 pp.]. Available from: http://cochranelibrary-wiley.com/o/cochrane/clcentral/articles/811/CN-00662811/frame.html.
33. Gimenez Camarasa JM SEFC, editor Prednicarbato, un nuevo corticoide tapico, en comparacian con f1uocinolona en el tratamiento de la dermatitis atapica. XXIII Congreso Nacional de la Academia Espanola de Dermatologia y Venereologia; 1994 Jun 16-18; Madrid.
34. Moshang T. Prednicarbate emollient cream 0.1% in pediatric patients with atopic dermatitis. *Cutis*. 2001;**68**(1):63-9.

For peer review only - http://bmjopen.bmj.com/site/about/guidelines.xhtml

35. Conde J, Kaur M, Fleischer Jr A, Tusa M, Camacho F, Feldman S. Adherence to clocortolone pivalate cream 0.1% in a pediatric population with atopic dermatitis. *Cutis*. 2008;**81**(5):435-41.
36. Crespi H. Topical corticosteroid therapy for children: alclometasone dipropionate cream 0.05%. *Clin Ther*. 1986;**8**(2):203-10.
37. Udompataikul M, Srisatwaja W. Comparative trial of moisturizer containing licochalcone A vs. hydrocortisone lotion in the treatment of childhood atopic dermatitis: a pilot study. *J Eur Acad Dermatol Venereol*. 2011;**25**(6):660-5.
38. Wananukul S, Chatproedprai S, Chunharas A, Limpongsanuruk W, Singalavanija S, Nitiyarom R, et al. Randomized, double-blind, split-side, comparison study of moisturizer containing licochalcone A and 1% hydrocortisone in the treatment of childhood atopic dermatitis. *J Med Assoc Thai [Chotmaihet thangphaet]*. 2013;**96**(9):1135-42.
39. Jirabundansuk P, Ophaswongse S, Udompataikul M. Comparative trial of moisturizer containing spent grain wax, Butyrospermum parkii extract, Argania spinosa kernel oil vs. 1% hydrocortisone cream in the treatment of childhood atopic dermatitis. *J Med Assoc Thai [Chotmaihet thangphaet]*. 2014;**97**(8):820-6.
40. Dolle S, Hoser D, Rasche C, Loddenkemper C, Maurer M, Zuberbier T, et al. Long-term reduction in local inflammation by a lipid raft molecule in atopic dermatitis. *Allergy*. 2010;**65**(9):1158-65.
41. Patzelt-Wenczler R, Ponce-Poschl E. Proof of efficacy of Kamillosan(R) cream in atopic eczema. *Eur J Med Res*. 2000;**5**(4):171-5.
42. Hebert A, Cook-Bolden F, Basu S, Calvarese B, Trancik R, Desonide Hydrogel Study Group. Safety and efficacy of desonide hydrogel 0.05% in pediatric subjects with atopic dermatitis. *J Drugs Dermatol*. 2007;**6**(2):175-81.
43. Paller A, Nimmagadda S, Schachner L, Mallory S, Kahn T, Willis I, et al. Fluocinolone acetonide 0.01% in peanut oil: therapy for childhood atopic dermatitis, even in patients who are peanut sensitive. *J Am Acad Dermatol*. 2003;**48**(4):569-77.
44. Patel L, Clayton P, Addison G, Price D, David T. Adrenal function following topical steroid treatment in children with atopic dermatitis. *Br J Dermatol*. 1995;**132**(6):950-5.
45. Dohil MA, Alvarez-Connelly E, Eichenfield LF. Fluocinolone acetonide 0.01% in peanut oil: safety and efficacy data in the treatment of childhood atopic dermatitis in infants as young as 3 months of age. *Pediatr Dermatol*. 2009;**26**(3):262-8.
46. Eichenfield LF, Basu S, Calvarese B, Trancik RJ. Effect of desonide hydrogel 0.05% on the hypothalamic-pituitary-adrenal axis in pediatric subjects with moderate to severe atopic dermatitis. *Pediatr Dermatol*. 2007;**24**(3):289-95.
47. Hebert AA, Desonide Foam Phase III Clinical Study Group. Desonide foam 0.05%: safety in children as young as 3 months. *J Am Acad Dermatol*. 2008;**59**(2):334-40.
48. Bieber T, Vick K, Folster-Holst R, Belloni-Fortina A, Stadtler G, Worm M, et al. Efficacy and safety of methylprednisolone aceponate ointment 0.1% compared to tacrolimus 0.03% in children and adolescents with an acute flare of severe atopic dermatitis. *Allergy*. 2007;**62**(2):184-9.
49. Broeders J, Ahmed Ali U, Fischer G. Systematic review and meta-analysis of randomized clinical trials (RCTs) comparing topical calcineurin inhibitors with topical corticosteroids for atopic dermatitis: A 15-year experience. *J Am Acad Dermatol*. 2016;**75**(2):410-9.e3.
50. Cury Martins J, Martins C, Aoki V, Gois AF, Ishii HA, da SEM. Topical tacrolimus for atopic dermatitis. Cochrane Database Syst Rev [Internet]. 2015; (7). Available from: http://onlinelibrary.wiley.com/doi/10.1002/14651858.CD009864.pub2/abstract.
51. Doss N, Kamoun M, Dubertret L, Cambazard F, Remitz A, Lahfa M, et al. Efficacy of tacrolimus 0.03% ointment as second-line treatment for children with moderate-to-severe atopic dermatitis: evidence from a randomized, double-blind non-inferiority trial vs. fluticasone 0.005% ointment. *Pediatr Allergy Immunol*. 2010;**21**(2 Pt 1):321-9.
52. Dong Y, Zeng W, Li W, Ma H, Zheng W. Efficacy and safety of topical tacrolimus for childhood atopic dermatitis; a meta-analysis. [Chinese]. *J Clin Dermatol*. 2017;**46**(4):239-42.
53. Doss N, Reitamo S, Dubertret L, Fekete G, Kamoun M, Lahfa M, et al. Superiority of tacrolimus 0.1% ointment compared with fluticasone 0.005% in adults with moderate to severe atopic dermatitis of the face: results from a randomized, double-blind trial. *Br J Dermatol*. 2009;**161**(2):427-34.
54. Luger T, Van Leent E, Graeber M, Hedgecock S, Thurston M, Kandra A, et al. SDZ ASM 981: an emerging safe and effective treatment for atopic dermatitis. *Br J Dermatol*. 2001;**144**(4):788-94.

For peer review only

55. Ashcroft D, Dimmock P, Garside R, Stein K, Williams H. Efficacy and tolerability of topical pimecrolimus and tacrolimus in the treatment of atopic dermatitis: Meta-analysis of randomised controlled trials. *BMJ* 2005;**330**(7490):516-22.
56. Ashcroft DM, Chen L-C, Garside R, Stein K, Williams HC. Topical pimecrolimus for eczema Cochrane Database Syst Rev [Internet]. 2007; (4). Available from: http://onlinelibrary.wiley.com/doi/10.1002/14651858.CD005500.pub2/abstract.
57. Luger T, Lahfa M, Folster-Holst R, Gulliver W, Allen R, Molloy S, et al. Long-term safety and tolerability of pimecrolimus cream 1% and topical corticosteroids in adults with moderate to severe atopic dermatitis. *J Dermatolog Treat*. 2004;**15**(3):169-78.
58. Mandelin J, Remitz A, Virtanen H, Reitamo S. One-year treatment with 0.1% tacrolimus ointment versus a corticosteroid regimen in adults with moderate to severe atopic dermatitis: A randomized, double-blind, comparative trial. *Acta Derm Venereol*. 2010;**90**(2):170-4.
59. Reitamo S, Rustin M, Ruzicka T, Cambazard F, Kalimo K, Friedmann P, et al. Efficacy and safety of tacrolimus ointment compared with that of hydrocortisone butyrate ointment in adult patients with atopic dermatitis. *J Allergy Clin Immunol*. 2002;**109**(3):547-55.
60. Iskedjian M, Piwko C, Shear N, Langley R, Einarson T. Topical calcineurin inhibitors in the treatment of atopic dermatitis: A meta-analysis of current evidence. *Am J Clin Dermatol*. 2004;**5**(4):267-79.
61. Penaloza Hidalgo B, Knight T, Burls A. A systematic review of effectiveness and cost effectiveness of tacrolimus ointment for topical treatment of atopic dermatitis in adults and children Health Technol Assess [Internet]. 2004; (4):[81 p.].
62. Reitamo S, Ortonne J, Sand C, Cambazard F, Bieber T, Folster-Holst R, et al. A multicentre, randomized, double-blind, controlled study of long-term treatment with 0.1% tacrolimus ointment in adults with moderate to severe atopic dermatitis. *Br J Dermatol*. 2005;**152**(6):1282-9.
63. Gradman J, Wolthers O. Short-term growth in children with eczema during treatment with topical mometasone furoate and tacrolimus. *Acta paediatrica*. 2007;**96**(8):1233-7.
64. Svensson A, Chambers C, Gånemo A, Mitchell S. A systematic review of tacrolimus ointment compared with corticosteroids in the treatment of atopic dermatitis. *Curr Med Res Opin*. 2011;**27**(7):1395-406.
65. FK506 Ointment Study Group. Phase III comparative study of FK506 ointment vs betamethasone valerate ointment in atopic dermatitis (trunk/extremities) [in Japanese]. *Nishinihon J Dermatol*. 1997;**59**:870-9.
66. Hofman T, Cranswick N, Kuna P, Boznanski A, Latos T, Gold M, et al. Tacrolimus ointment does not affect the immediate response to vaccination, the generation of immune memory, or humoral and cell-mediated immunity in children. *Arch Dis Child*. 2006;**91**(11):905-10.
67. Siegfried E, Jaworski J, Kaiser J, Hebert A. Systematic review of published trials: Long-term safety of topical corticosteroids and topical calcineurin inhibitors in pediatric patients with atopic dermatitis. *BMC Pediatr*. 2016;**16** (75).
68. Sikder M, Al Mamun S, Khan R, Chowdhury A, Khan H, Hoque M. Topical 0.03% tacrolimus ointment, 0.05% clobetasone butyrate cream alone and their combination in older children with atopic dermatitis - An open randomized comparative study. *J Pak Assoc Dermatol*. 2005;**15**(4):304-12.
69. Torok H, Maas-Irslinger R, Slayton R. Clocortolone pivalate cream 0.1% used concomitantly with tacrolimus ointment 0.1% in atopic dermatitis. *Cutis*. 2003;**72**(2):161-6.
70. Sigurgeirsson B, Boznanski A, Todd G, Vertruyen A, Schuttelaar M, Zhu X, et al. Safety and efficacy of pimecrolimus in atopic dermatitis: a 5-year randomized trial. *Pediatrics* 2015;**135**(4):597-606.
71. Reitamo S, Van Leent E, Ho V, Harper J, Ruzicka T, Kalimo K, et al. Efficacy and safety of tacrolimus ointment compared with that of hydrocortisone acetate ointment in children with atopic dermatitis. *J Allergy Clin Immunol*. 2002;**109**(3):539-46.
72. Reitamo S, Harper J, Bos J, Cambazard F, Bruijnzeel-Koomen C, Valk P, et al. 0.03% Tacrolimus ointment applied once or twice daily is more efficacious than 1% hydrocortisone acetate in children with moderate to severe atopic dermatitis: results of a randomized double-blind controlled trial. *Br J Dermatol*. 2004;**150**(3):554-62.
73. Gutgesell C, Jung T, Reich K, Junghans V, Bohn M, Neumann C. Double-blind hydrocortisone-controlled tacrolimus ointment for atopic dermatitis.(Abstract 1255). *J Invest Dermatol*. 1998;**110**(4):681.

For peer review only

74. Arellano F, Wentworth C, Arana A, Fernandez C, Paul C. Risk of lymphoma following exposure to calcineurin inhibitors and topical steroids in patients with atopic dermatitis. *J Invest Dermatol*. 2007;**127**(4):808-16.
75. Arellano F, Arana A, Wentworth C, Fernandez-Vidaurre C, Schlienger R, Conde E. Lymphoma among patients with atopic dermatitis and/or treated with topical immunosuppressants in the United Kingdom. *J Allergy Clin Immunol*. 2009;**123**(5):1111–6.
76. Schneeweiss S, Doherty M, Zhu S, Funch D, Schlienger R, Fernandez-Vidaurre C, et al. Topical treatments with pimecrolimus, tacrolimus and medium- to high-potency corticosteroids, and risk of lymphoma. *Dermatology*. 2009;**219**(1):7-21.
77. Reitamo S WA, Schopf E, Perrot JL, Marks R, Ruzicka T, et al. Safety and efficacy of 1 year of tacrolimus ointment monotherapy in adults with atopic dermatitis.The European Tacrolimus Ointment Study Group. *Arch Dermatol*. 2000;**136**(8):999-1006.
78. Ulrich R, Andresen I. Double-blind comparative trial involving 0.5% halomethasone (Sicorten (R)) cream versus 0.25% prednicarbate cream in patients with acute episodes of atopic dermatitis [Behandlung akuter schube der atopischen dermatitis. Doppelblinde vergleichsstudie mit 0,05%-halometason-creme versus 0,25%-prednicarbat-creme]. *MMW Fortschr Med*. 1991;**109**(36):49-50+3-4.
79. Smitt J, Winterberg D, Oosting J. Treatment of atopic dermatitis with topical corticosteroids in children. Efficacy and systemic effects of triamcinolone acetonide and alclometasone dipropionate. *Eur J Dermatol*. 1993;**3**(7):549-52.
80. Lebrun-Vignes B, Legrain V, Amoric J, Taieb A. [Comparative study of efficacy and effect on plasma cortisol levels of micronised desonide cream 0.1 p. 100 versus betamethasone dipropionate cream 0.05 p. 100 In the treatment of childhood atopic dermatitis] (French). *Ann Dermatol Venereol*. 2000;**127**(6-7):590-5.
81. Prado de Oliveira Z, Cuce L, Arnone M. Comparative evaluation of efficacy, tolerability and safety of 0.1% topical momethasone furoate and 0.05% desonide in the treatment of childhood atopic dermatitis. *An Bras Dermatol*. 2002;**77**:25-33.
82. Hanifin J. The role of topical steroids in the treatment of allergic skin disease. Towards Evolution of Allergic Skin Disease Management. Hong Kong: Excerpta Medica; 1996. p. 1-2.
83. Kirkup M, Birchall N, Weinberg E, Helm K, Kennedy C. Acute and maintenance treatment of atopic dermatitis in children - two comparative studies with fluticasone propionate (0.05%) cream. *J Dermatolog Treat*. 2003;**14**(3):141-8.
84. Tang T, Bieber T, Williams H. Are the concepts of induction of remission and treatment of subclinical inflammation in atopic dermatitis clinically useful? *J Allergy Clin Immunol*. 2014;**133**(6):1615-25.e1.
85. Kuokkanen K, Sillantaka I. Alclometasone dipropionate 0.05% vs hydrocortisone 1.0%: potential to induce cutaneous atrophy in children. *Clin Ther*. 1987;**9**(2):223-31.
86. Ellison JA, Patel L, Ray DW, David TJ, Clayton PE. Hypothalamic-pituitary-adrenal function and glucocorticoid sensitivity in atopic dermatitis. *Pediatrics*. 2000;**105**(4 Pt 1):794-9.
87. Eichenfield L, Tom W, Berger T, Krol A, Paller A, Schwarzenberger K, et al. Guidelines of care for the management of atopic dermatitis: Section 2. Management and treatment of atopic dermatitis with topical therapies. *J Am Acad Dermatol*. 2014;**71**(1):116-32.
88. Kristmundsdottir F, David T. Growth impairment in children with atopic eczema. *Journal of the Royal Society of Medicine*. 1987;**80**(1):9-12.
89. Patel L, Clayton P, Jenney M, Ferguson J, David T. Adult height in patients with childhood onset atopic dermatitis. *Arch Dis Child*. 1997;**76**(6):505-7.
90. Patel L, Clayton P, Addison G, Price D, David T. Linear growth in prepubertal children with atopic dermatitis. *Arch Dis Child*. 1998;**79**(2):169-72.
91. Aliaga A, Rodriguez M, Armijo M, Bravo J, Avila AL, Mascaro JM, et al. Double-blind study of prednicarbate versus fluocortin butyl ester in atopic dermatitis. *Int J Dermatol*. 1996;**35**(2):131-2.
92. Lucky A, Grote G, Williams J, Tuley M, Czernielewski J, Dolak T, et al. Effect of desonide ointment, 0.05%, on the hypothalamic-pituitary-adrenal axis of children with atopic dermatitis. *Cutis*. 1997;**59**(3):151-3.
93. Jorizzo J, Levy M, Lucky A, Shavin J, Goldberg G, Dunlap F, et al. Multicenter trial for long-term safety and efficacy comparison of 0.05% desonide and 1% hydrocortisone ointments in the treatment of atopic dermatitis in pediatric patients. *J Am Acad Dermatol*. 1995;**33**(1):74-7.

For peer review only

94. Froeschl B, Arts D, Leopold C. Corticosteroid therapy in the treatment of pediatric patients with atopic dermatitis (Structured abstract). Health Technol Assess [Internet]. 2007; (4). Available from: http://onlinelibrary.wiley.com/o/cochrane/clhta/articles/HTA-32008100208/frame.html.
95. Huang Z, Chen P, Wei W. Observation of efficacy of Chushi Zhiyang cream for atopic dermatitis. *Mod J Integr Trad Chin West Med*. 2010;**19**:2647–8.
96. Gu S, Yang AW, Xue CC, Li CG, Pang C, Zhang W, et al. Chinese herbal medicine for atopic eczema. Cochrane Database Syst Rev [Internet]. 2013; (9). Available from: http://onlinelibrary.wiley.com/doi/10.1002/14651858.CD008642.pub2/abstract.
97. Gu S, Yang A, Li C, Lu C, Xue C. Topical application of Chinese herbal medicine for atopic eczema: A systematic review with a meta-analysis. *Dermatology*. 2014;**228**(4):294-302.
98. Chen H. Clinical observation of Huanglian Qingdai cream for infantile eczema

J Sichuan Trad Chin Med 2011;**29**:88–9.
99. Dong M. Efficacy of external use of Jingfang mixture in the treatment of children with eczema. *Med J Qilu*. 2012;**27**:75–8.
100. Xu XC GY. Clinical observation of Kouqiang Xiaoyan powder for infantile eczema. *Strait Pharm J*. 2012;**24**:210.
101. Schlessinger J, Miller B, Gilbert R, Plott R, Vanos Study Group. An open-label adrenal suppression study of 0.1% fluocinonide cream in pediatric patients with atopic dermatitis. *Arch Dermatol*. 2006;**142**(12):1568-72.
102. Bleehen S, Chu A, Hamann I, Holden C, Hunter J, Marks R. Fluticasone propionate 0.05% cream in the treatment of atopic eczema: a multicentre study comparing once-daily treatment and once-daily vehicle cream application versus twice-daily treatment. *Br J Dermatol*. 1995;**133**(4):592-7.
103. Green C, Colquitt J, Kirby J, Davidson P, Payne E. Clinical and cost-effectiveness of once-daily versus more frequent use of same potency topical corticosteroids for atopic eczema: a systematic review and economic evaluation. *Health Technol Assess (Winchester, England)*. 2004;**8**(47):iii,iv, 1-120.
104. (GSK) G. A four week multicentre, double blind study to compare safety and efficacy with an OD and BD administration of fluticasone propionate 0.005% ointment in the treatment of atopic eczema. 1995. Contract No.: Report 135L,Protocol No. GL/FLT/002.
105. Koopmans B, Lasthein Andersen B, Mork N, Austad J, Suhonen R, Roders G. Multicentre randomized double-blind study of Locoid Lipocream fatty cream twice daily versus Locoid Lipocream once daily and Locobase once daily. *J Dermatolog Treat*. 1995;**6**(2):103-6.
106. Tharp M. A comparison of twice-daily and once-daily administration of fluticasone propionate cream, 0.05%, in the treatment of eczema. *Cutis*. 1996;**57**(Suppl 2):19-26.
107. Hoybye S, Balk Moller S, De Cunha Bang F, Ottevanger V, Veien N. Continuous and intermittent treatment of atopic dermatitis in adults with mometasone furoate versus hydrocortisone 17-butyrate. *Curr Ther Res Clin Exp*. 1991;**50**(1):67-72.
108. Berth-Jones J, Damstra R, Golsch S, Livden J, Van Hooteghem O, Allegra F, et al. Twice weekly fluticasone propionate added to emollient maintenance treatment to reduce risk of relapse in atopic dermatitis: randomised, double blind, parallel group study. *BMJ* 2003;**326**(7403):1367-9.
109. Marchesi E, Rozzoni M, Pini P, Cainelli T. Comparative study of mometasone furoate and betamethasone dipropionate in the treatment of atopic dermatitis. *G Ital Dermatol Venereol*. 1994;**129**(1-2):IX-XII.
110. Richelli C, Piacentini G, Sette L, Bonizzato M, Andreoli A, Boner A. Clinical efficacy and tolerability of clobetasone 17-butyrate 0.5% lotion in children with atopic dermatitis. *Curr Ther Res Clin Exp*. 1990;**47**(3):413-7.
111. Schmitt J, Von Kobyletzki L, Svensson A, Apfelbacher C. Efficacy and tolerability of proactive treatment with topical corticosteroids and calcineurin inhibitors for atopic eczema: Systematic review and meta-analysis of randomized controlled trials. *Br J Dermatol*. 2011;**164**(2):415-28.
112. Glazenburg E, Wolkerstorfer A, Gerretsen A, Mulder P, Oranje A. Efficacy and safety of fluticasone propionate 0.005% ointment in the long-term maintenance treatment of children with atopic dermatitis: differences between boys and girls? *Pediatr Allergy Immunol*. 2009;**20**(1):59-66.
113. Hanifin J, Gupta A, Rajagopalan R. Intermittent dosing of fluticasone propionate cream for reducing the risk of relapse in atopic dermatitis patients. *Br J Dermatol*. 2002;**147**(3):528-37.

For peer review only

114. Van Der Meer J, Glazenburg E, Mulder P, Eggink H, Coenraads P. The management of moderate to severe atopic dermatitis in adults with topical fluticasone propionate. The Netherlands Adult Atopic DermatitisStudy Group. *Br J Dermatol*. 1999;**140**(6):1114-21.
115. Peserico A, Stadtler G, Sebastian M, Fernandez R, Vick K, Bieber T. Reduction of relapses of atopic dermatitis with methylprednisolone aceponate cream twice weekly in addition to maintenance treatment with emollient: a multicentre, randomized, double-blind, controlled study. *Br J Dermatol*. 2008;**158**(4):801-7.
116. Volden G. Successful treatment of therapy-resistant atopic dermatitis with clobetasol propionate and a hydrocolloid occlusive dressing. *Acta Derm Venereol Suppl (Stockh)*. 1992;**176**:126-8.
117. Braham S, Pugashetti R, Koo J, Maibach H. Occlusive therapy in atopic dermatitis: overview. *J Dermatolog Treat*. 2010;**21**(2):62-72.
118. Janmohamed S, Oranje A, Devillers A, Rizopoulos D, van Praag M, Van Gysel D, et al. The proactive wet-wrap method with diluted corticosteroids versus emollients in children with atopic dermatitis: a prospective, randomized, double-blind, placebo-controlled trial. *J Am Acad Dermatol*. 2014;**70**(6):1076-82.
119. Schnopp C, Holtmann C, Stock S, Remling R, Folster-Holst R, Ring J, et al. Topical steroids under wet-wrap dressings in atopic dermatitis - A vehicle-controlled trial. *Dermatology*. 2002;**204**(1):56-9.
120. McGowan R, Tucker P, Joseph D, Wallace A, Hughes I, Burrows N, et al. Short-term growth and bone turnover in children undergoing occlusive steroid ('wet-wrap') dressings for treatment of atopic eczema. *J Dermatolog Treat*. 2003;**14**(3):149-52.
121. Devillers A, Oranje A. Efficacy and safety of 'wet-wrap' dressings as an intervention treatment in children with severe and/or refractory atopic dermatitis: a critical review of the literature. *Br J Dermatol*. 2006;**154**(4):579-85.
122. Wolkerstorfer A, Visser R, De Waard van der Spek F, Mulder P, Oranje A. Efficacy and safety of wet-wrap dressings in children with severe atopic dermatitis: influence of corticosteroid dilution. *Br J Dermatol*. 2000;**143**(5):999-1004.
123. Tang W. Diluted steroid facial wet wraps for childhood atopic eczema. *Dermatology*. 2000;**200**(4):338-9.
124. Goodyear H, Spowart K, Harper J. 'Wet-wrap'dressings for the treatment of atopic eczema in children. *Br J Dermatol*. 1991;**125**(6):604.
125. Mallon E, Powell S, Bridgman A. 'Wet-wrap'dressings for the treatment of atopic eczema in the community. *J Dermatolog Treat*. 1994;**5**(2):97-8.
126. Devillers A, de Waard-van der Spek F, Mulder P, Oranje A. Treatment of refractory atopic dermatitis using 'wet-wrap'dressings and diluted corticosteroids: results of standardized treatment in both children and adults. *Dermatology*. 2002;**204**(1):50-5.
127. Foelster-Holst R, Nagel F, Zoellner P, Spaeth D. Efficacy of crisis intervention treatment with topical corticosteroid prednicarbat with and without partial wet-wrap dressing in atopic dermatitis. *Dermatology*. 2006;**212**(1):66-9.
128. Gonzalez-Lopez G, Ceballos-Rodriguez R, Gonzalez-Lopez J, Feito Rodriguez M, Herranz-Pinto P. Efficacy and safety of wet wrap therapy for patients with atopic dermatitis: a systematic review and meta-analysis. *Br J Dermatol*. 2017;**177**(3):688-95.
129. Beattie P, Lewis-Jones M. A pilot study on the use of wet wraps in infants with moderate atopic eczema. *Clin Exp Dermatol*. 2004;**29**(4):348-53.
130. Hindley D, Galloway G, Murray J, Gardener L. A randomised study of "wet wraps" versus conventional treatment for atopic eczema. *Arch Dis Child*. 2006;**91**(2):164-8.

For peer review only

Appendix 7: edits made to meta-analyses and data from Broeders *et al* 2016 [1]

1) Switched the forest plot labels around so topical corticosteroids are the intervention and topical calcineurin inhibitors are is the comparator.
2) Amended a data error given in the skin infection forest plot where the number of events and participants were given the wrong way round for topical corticosteroids and topical calcineurin inhibitors in Luger et al 2004 [2].
3) Added skin atrophy data from Sigurgeirsson et al 2015 [3] into the forest plot – this is not provided in the publication but is given in online correspondence on the journal website
4) Changed to random effects instead of fixed effects as the decision was based on whether the I^2 value which is not appropriate.
5) Bieber et al 2007 [4] was listed as "least potent" in table I of the publication – but according to the Australian potency classification it should be classified as potent.
6) In table I, the topical calcineurin inhibitors given for Mandelin et al 2010 [5] is tacrolimus 1% - this should be 0.1%.
7) In table I, the therapy given for Hofman et al 2006 [6] was hydrocortisone acetate 0.1%. However, patients used hydrocortisone ointment 1% (mild potency) twice daily for head/neck and hydrocortisone butyrate ointment 0.1% (potent) for trunk and limbs for 2 weeks then hydrocortisone 1% twice daily for flares.

References

1. Broeders J, Ahmed Ali U, Fischer G. Systematic review and meta-analysis of randomized clinical trials (RCTs) comparing topical calcineurin inhibitors with topical corticosteroids for atopic dermatitis: A 15-year experience. *J Am Acad Dermatol*. 2016;**75**(2):410-9.e3.
2. Luger T, Van Leent E, Graeber M, Hedgecock S, Thurston M, Kandra A, et al. SDZ ASM 981: an emerging safe and effective treatment for atopic dermatitis. *Br J Dermatol*. 2001;**144**(4):788-94.
3. Sigurgeirsson B, Boznanski A, Todd G, Vertruyen A, Schuttelaar M, Zhu X, et al. Safety and efficacy of pimecrolimus in atopic dermatitis: a 5-year randomized trial. *Pediatrics* 2015;**135**(4):597-606.
4. Bieber T, Vick K, Folster-Holst R, Belloni-Fortina A, Stadtler G, Worm M, et al. Efficacy and safety of methylprednisolone aceponate ointment 0.1% compared to tacrolimus 0.03% in children and adolescents with an acute flare of severe atopic dermatitis. *Allergy*. 2007;**62**(2):184-9.
5. Mandelin J, Remitz A, Virtanen H, Reitamo S. One-year treatment with 0.1% tacrolimus ointment versus a corticosteroid regimen in adults with moderate to severe atopic dermatitis: A randomized, double-blind, comparative trial. *Acta Derm Venereol*. 2010;**90**(2):170-4.
6. Hofman T, Cranswick N, Kuna P, Boznanski A, Latos T, Gold M, et al. Tacrolimus ointment does not affect the immediate response to vaccination, the generation of immune memory, or humoral and cell-mediated immunity in children. *Arch Dis Child*. 2006;**91**(11):905-10.

For peer review only

Appendix 8 – meta-analysis of TCS versus TCI – cutaneous adverse events

1) Skin thinning

Study or Subgroup	Corticosteroid (TCS) Events	Total	Calcineurin inhibitor (TCI) Events	Total	Weight	Risk Ratio M-H, Random, 95% CI
Luger 2004 (1)	3	330	0	328	26.5%	6.96 [0.36, 134.17]
Mandelin 2010 (2)	2	40	0	40	25.7%	5.00 [0.25, 100.97]
Reitamo 2005 (3)	2	485	0	487	25.2%	5.02 [0.24, 104.30]
Sigurgeirsson 2015 (4)	1	1213	0	1205	22.7%	2.98 [0.12, 73.08]
**Total (95% CI)**		**2068**		**2060**	**100.0%**	**4.86 [1.06, 22.28]**
Total events	8		0			

Heterogeneity: $Tau^2 = 0.00$; $Chi^2 = 0.15$, df = 3 (P = 0.99); $I^2 = 0\%$
Test for overall effect: Z = 2.03 (P = 0.04)

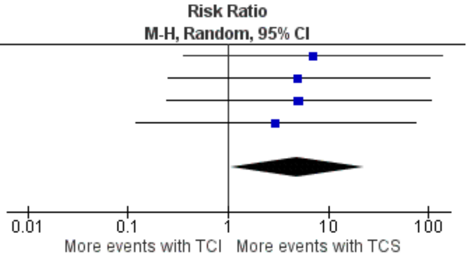

2) Skin burning

Study or Subgroup	Corticosteroid (TCS) Events	Total	Calcineurin inhibitor (TCI) Events	Total	Weight	Risk Ratio M-H, Random, 95% CI
Doss 2009 (5)	9	279	47	287	9.1%	0.20 [0.10, 0.39]
Doss 2010 (6)	6	239	18	237	6.4%	0.33 [0.13, 0.82]
Hofman 2006 (7)	2	124	10	133	2.9%	0.21 [0.05, 0.96]
Luger 2001 (8)	4	42	22	45	5.8%	0.19 [0.07, 0.52]
Luger 2004 (1)	36	330	85	328	15.6%	0.42 [0.29, 0.60]
Reitamo 2002(I) (9)	24	186	113	191	14.9%	0.22 [0.15, 0.32]
Reitamo 2002(II) (10)	13	185	38	186	10.7%	0.34 [0.19, 0.62]
Reitamo 2004 (11)	30	207	50	210	14.5%	0.61 [0.40, 0.92]
Reitamo 2005 (3)	67	485	255	487	18.4%	0.26 [0.21, 0.33]
Sikder 2005 (12)	1	15	7	15	1.8%	0.14 [0.02, 1.02]
**Total (95% CI)**		**2092**		**2119**	**100.0%**	**0.31 [0.23, 0.40]**
Total events	192		645			

Heterogeneity: $Tau^2 = 0.09$; $Chi^2 = 21.66$, df = 9 (P = 0.01); $I^2 = 58\%$
Test for overall effect: Z = 8.44 (P < 0.00001)

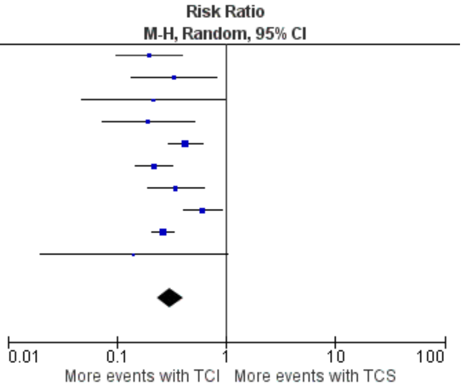

3) Pruritus

Study or Subgroup	Corticosteroid (TCS) Events	Total	Calcineurin inhibitor (TCI) Events	Total	Weight	Risk Ratio M-H, Random, 95% CI
Doss 2009 (5)	9	279	12	287	4.7%	0.77 [0.33, 1.80]
Doss 2010 (6)	8	239	10	237	4.1%	0.79 [0.32, 1.98]
Hofman 2006 (7)	4	124	8	133	2.5%	0.54 [0.17, 1.74]
Luger 2001 (8)	5	42	14	45	3.9%	0.38 [0.15, 0.97]
Luger 2004 (1)	6	330	18	328	4.1%	0.33 [0.13, 0.82]
Reitamo 2002(I) (9)	18	186	29	191	11.2%	0.64 [0.37, 1.11]
Reitamo 2002(II) (10)	14	185	21	186	8.2%	0.67 [0.35, 1.28]
Reitamo 2004 (11)	33	207	45	210	20.7%	0.74 [0.50, 1.12]
Reitamo 2005 (3)	65	485	88	487	39.3%	0.74 [0.55, 1.00]
Sikder 2005 (12)	2	15	3	15	1.3%	0.67 [0.13, 3.44]
**Total (95% CI)**		**2092**		**2119**	**100.0%**	**0.68 [0.56, 0.82]**
Total events	164		248			

Heterogeneity: $Tau^2 = 0.00$; $Chi^2 = 4.80$, df = 9 (P = 0.85); $I^2 = 0\%$
Test for overall effect: Z = 4.11 (P < 0.0001)

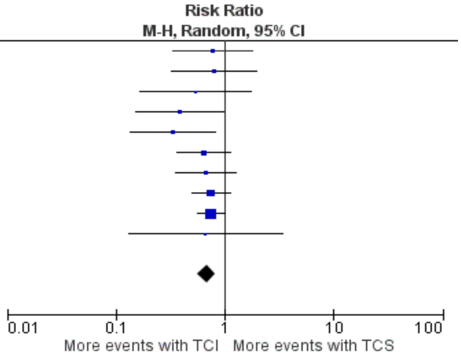

4) Skin infections

Study or Subgroup	Corticosteroid (TCS) Events	Total	Calcineurin inhibitor (TCI) Events	Total	Weight	Risk Ratio M-H, Random, 95% CI
Doss 2010 (6)	49	239	44	239	14.6%	1.11 [0.77, 1.60]
Hofman 2006 (7)	4	124	2	133	0.7%	2.15 [0.40, 11.51]
Luger 2004 (1)	80	330	69	328	24.2%	1.15 [0.87, 1.53]
Mandelin 2010 (2)	17	40	26	40	10.7%	0.65 [0.43, 1.00]
Reitamo 2002(II) (10)	4	185	4	186	1.0%	1.01 [0.26, 3.96]
Reitamo 2004 (11)	6	207	6	210	1.6%	1.01 [0.33, 3.09]
Reitamo 2005 (3)	9	485	13	487	2.8%	0.70 [0.30, 1.61]
Sigurgeirsson 2015 (4)	150	1213	157	1205	44.5%	0.95 [0.77, 1.17]
**Total (95% CI)**		**2823**		**2828**	**100.0%**	**0.98 [0.85, 1.12]**
Total events	319		321			

Heterogeneity: $Tau^2 = 0.00$; $Chi^2 = 6.80$, df = 7 (P = 0.45); $I^2 = 0\%$
Test for overall effect: Z = 0.33 (P = 0.74)

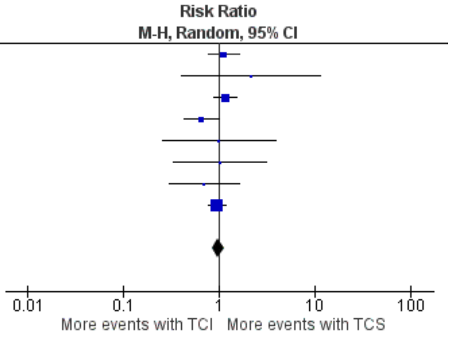

References:

1. Luger T, Lahfa M, Folster-Holst R, Gulliver W, Allen R, Molloy S, et al. Long-term safety and tolerability of pimecrolimus cream 1% and topical corticosteroids in adults with moderate to severe atopic dermatitis. *J Dermatolog Treat*. 2004;**15**(3):169-78
2. Mandelin J, Remitz A, Virtanen H, Reitamo S. One-year treatment with 0.1% tacrolimus ointment versus a corticosteroid regimen in adults with moderate to severe atopic dermatitis: A randomized, double-blind, comparative trial. *Acta Derm Venereol*. 2010;**90**(2):170-4
3. Reitamo S, Ortonne J, Sand C, Cambazard F, Bieber T, Folster-Holst R, et al. A multicentre, randomized, double-blind, controlled study of long-term treatment with 0.1% tacrolimus ointment in adults with moderate to severe atopic dermatitis. *Br J Dermatol*. 2005;**152**(6):1282-9
4. Sigurgeirsson B, Boznanski A, Todd G, Vertruyen A, Schuttelaar M, Zhu X, et al. Safety and efficacy of pimecrolimus in atopic dermatitis: a 5-year randomized trial. *Pediatrics* 2015;**135**(4):597-606
5. Doss N, Reitamo S, Dubertret L, Fekete G, Kamoun M, Lahfa M, et al. Superiority of tacrolimus 0.1% ointment compared with fluticasone 0.005% in adults with moderate to severe atopic dermatitis of the face: results from a randomized, double-blind trial. *Br J Dermatol*. 2009;**161**(2):427-34
6. Doss N, Kamoun M, Dubertret L, Cambazard F, Remitz A, Lahfa M, et al. Efficacy of tacrolimus 0.03% ointment as second-line treatment for children with moderate-to-severe atopic dermatitis: evidence from a randomized, double-blind non-inferiority trial vs. fluticasone 0.005% ointment. *Pediatr Allergy Immunol*. 2010;**21**(2 Pt 1):321-9.
7. Hofman T, Cranswick N, Kuna P, Boznanski A, Latos T, Gold M, et al. Tacrolimus ointment does not affect the immediate response to vaccination, the generation of immune memory, or humoral and cell-mediated immunity in children. *Arch Dis Child*. 2006;**91**(11):905-10
8. Luger T, Van Leent E, Graeber M, Hedgecock S, Thurston M, Kandra A, et al. SDZ ASM 981: an emerging safe and effective treatment for atopic dermatitis. *Br J Dermatol*. 2001;**144**(4):788-94
9. Reitamo S, Rustin M, Ruzicka T, Cambazard F, Kalimo K, Friedmann P, et al. Efficacy and safety of tacrolimus ointment compared with that of hydrocortisone butyrate ointment in adult patients with atopic dermatitis. *J Allergy Clin Immunol*. 2002;**109(**3):547-55
10. Reitamo S, Van Leent E, Ho V, Harper J, Ruzicka T, Kalimo K, et al. Efficacy and safety of tacrolimus ointment compared with that of hydrocortisone acetate ointment in children with atopic dermatitis. *J Allergy Clin Immunol*. 2002;**109**(3):539-46
11. Reitamo S, Harper J, Bos J, Cambazard F, Bruijnzeel-Koomen C, Valk P, et al. 0.03% Tacrolimus ointment applied once or twice daily is more efficacious than 1% hydrocortisone acetate in children with moderate to severe atopic dermatitis: results of a randomized double-blind controlled trial. *Br J Dermatol*. 2004;**150**(3):554-62
12. Sikder M, Al Mamun S, Khan R, Chowdhury A, Khan H, Hoque M. Topical 0.03% tacrolimus ointment, 0.05% clobetasone butyrate cream alone and their combination in older children with atopic dermatitis - An open randomized comparative study. *J Pak Assoc Dermatol*. 2005;**15**(4):304-12

For peer review only

Appendix 9: subgroup analyses of TCS versus TCI – cutaneous adverse events

By different topical corticosteroid potencies

Skin thinning

Study or Subgroup	Corticosteroids (TCS) Events	Corticosteroids (TCS) Total	Calcineurin inhibitor (TCI) Events	Calcineurin inhibitor (TCI) Total	Weight	Risk Ratio M-H, Random, 95% CI
**2.6.1 Potent**						
Luger 2004 (1)	3	330	0	328	26.5%	6.96 [0.36, 134.17]
Mandelin 2010 (2)	2	40	0	40	25.7%	5.00 [0.25, 100.97]
Reitamo 2005 (3)	2	485	0	487	25.2%	5.02 [0.24, 104.30]
**Subtotal (95% CI)**		**855**		**855**	**77.3%**	**5.61 [0.99, 31.67]**
Total events	7		0			
Heterogeneity: Tau² = 0.00; Chi² = 0.03, df = 2 (P = 0.98); I² = 0%						
Test for overall effect: Z = 1.95 (P = 0.05)						
**2.6.2 Moderate or mild potency**						
Sigurgeirsson 2015 (4)	1	1213	0	1205	22.7%	2.98 [0.12, 73.08]
**Subtotal (95% CI)**		**1213**		**1205**	**22.7%**	**2.98 [0.12, 73.08]**
Total events	1		0			
Heterogeneity: Not applicable						
Test for overall effect: Z = 0.67 (P = 0.50)						
**Total (95% CI)**		**2068**		**2060**	**100.0%**	**4.86 [1.06, 22.28]**
Total events	8		0			
Heterogeneity: Tau² = 0.00; Chi² = 0.15, df = 3 (P = 0.99); I² = 0%						
Test for overall effect: Z = 2.03 (P = 0.04)						
Test for subgroup differences: Chi² = 0.12, df = 1 (P = 0.73), I² = 0%						

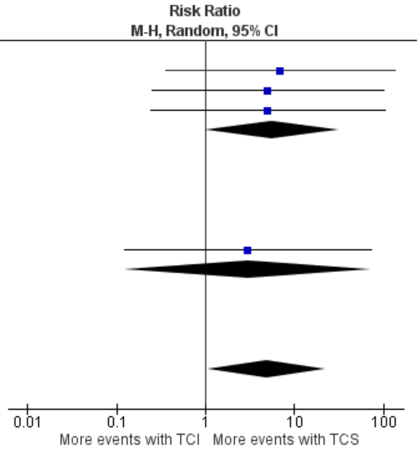

Skin burning

Study or Subgroup	Corticosteroids (TCS) Events	Corticosteroids (TCS) Total	Calcineurin inhibitor (TCI) Events	Calcineurin inhibitor (TCI) Total	Weight	Risk Ratio M-H, Random, 95% CI
**2.2.1 Potent**						
Doss 2009 (5)	9	279	47	287	9.1%	0.20 [0.10, 0.39]
Doss 2010 (6)	6	239	18	237	6.4%	0.33 [0.13, 0.82]
Luger 2001 (7)	4	42	22	45	5.8%	0.19 [0.07, 0.52]
Luger 2004 (1)	36	330	85	328	15.6%	0.42 [0.29, 0.60]
Reitamo 2002(I) (8)	24	186	113	191	14.9%	0.22 [0.15, 0.32]
Reitamo 2005 (3)	67	485	255	487	18.4%	0.26 [0.21, 0.33]
**Subtotal (95% CI)**		**1561**		**1575**	**70.2%**	**0.27 [0.21, 0.35]**
Total events	146		540			
Heterogeneity: Tau² = 0.04; Chi² = 8.42, df = 5 (P = 0.13); I² = 41%						
Test for overall effect: Z = 10.11 (P < 0.00001)						
**2.2.2 Potent or mild potency**						
Hofman 2006 (9)	2	124	10	133	2.9%	0.21 [0.05, 0.96]
**Subtotal (95% CI)**		**124**		**133**	**2.9%**	**0.21 [0.05, 0.96]**
Total events	2		10			
Heterogeneity: Not applicable						
Test for overall effect: Z = 2.01 (P = 0.04)						
**2.2.3 Moderate potency**						
Sikder 2005 (10)	1	15	7	15	1.8%	0.14 [0.02, 1.02]
**Subtotal (95% CI)**		**15**		**15**	**1.8%**	**0.14 [0.02, 1.02]**
Total events	1		7			
Heterogeneity: Not applicable						
Test for overall effect: Z = 1.94 (P = 0.05)						
**2.2.4 Mild potency**						
Reitamo 2002(II) (11)	13	185	38	186	10.7%	0.34 [0.19, 0.62]
Reitamo 2004 (12)	30	207	50	210	14.5%	0.61 [0.40, 0.92]
**Subtotal (95% CI)**		**392**		**396**	**25.1%**	**0.48 [0.27, 0.83]**
Total events	43		88			
Heterogeneity: Tau² = 0.10; Chi² = 2.41, df = 1 (P = 0.12); I² = 59%						
Test for overall effect: Z = 2.61 (P = 0.009)						
**Total (95% CI)**		**2092**		**2119**	**100.0%**	**0.31 [0.23, 0.40]**
Total events	192		645			
Heterogeneity: Tau² = 0.09; Chi² = 21.66, df = 9 (P = 0.01); I² = 58%						
Test for overall effect: Z = 8.44 (P < 0.00001)						
Test for subgroup differences: Chi² = 3.92, df = 3 (P = 0.27), I² = 23.4%						

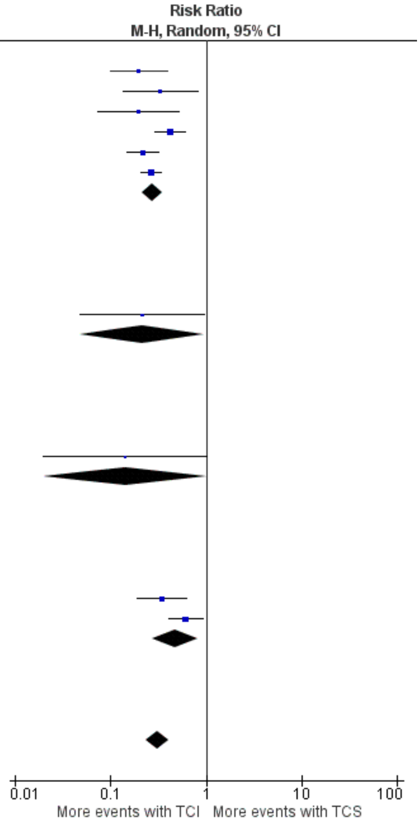

For peer review only - http://bmjopen.bmj.com/site/about/guidelines.xhtml

Pruritus

Study or Subgroup	Corticosteroids (TCS) Events	Total	Calcineurin inhibitor (TCI) Events	Total	Weight	Risk Ratio M-H, Random, 95% CI
**2.3.1 Potent**						
Doss 2009 (5)	9	279	12	287	4.7%	0.77 [0.33, 1.80]
Doss 2010 (6)	8	239	10	237	4.1%	0.79 [0.32, 1.98]
Luger 2001 (7)	5	42	14	45	3.9%	0.38 [0.15, 0.97]
Luger 2004 (1)	6	330	18	328	4.1%	0.33 [0.13, 0.82]
Reitamo 2002(I) (8)	18	186	29	191	11.2%	0.64 [0.37, 1.11]
Reitamo 2005 (3)	65	485	88	487	39.3%	0.74 [0.55, 1.00]
**Subtotal (95% CI)**		**1561**		**1575**	**67.4%**	**0.67 [0.53, 0.84]**
Total events	111		171			
Heterogeneity: Tau² = 0.00; Chi² = 4.43, df = 5 (P = 0.49); I² = 0%						
Test for overall effect: Z = 3.53 (P = 0.0004)						
**2.3.2 Potent or mild potency**						
Hofman 2006 (9)	4	124	8	133	2.5%	0.54 [0.17, 1.74]
**Subtotal (95% CI)**		**124**		**133**	**2.5%**	**0.54 [0.17, 1.74]**
Total events	4		8			
Heterogeneity: Not applicable						
Test for overall effect: Z = 1.04 (P = 0.30)						
**2.3.3 Moderate potency**						
Sikder 2005 (10)	2	15	3	15	1.3%	0.67 [0.13, 3.44]
**Subtotal (95% CI)**		**15**		**15**	**1.3%**	**0.67 [0.13, 3.44]**
Total events	2		3			
Heterogeneity: Not applicable						
Test for overall effect: Z = 0.48 (P = 0.63)						
**2.3.4 Mild potency**						
Reitamo 2002(II) (11)	14	185	21	186	8.2%	0.67 [0.35, 1.28]
Reitamo 2004 (12)	33	207	45	210	20.7%	0.74 [0.50, 1.12]
**Subtotal (95% CI)**		**392**		**396**	**28.9%**	**0.72 [0.51, 1.02]**
Total events	47		66			
Heterogeneity: Tau² = 0.00; Chi² = 0.07, df = 1 (P = 0.79); I² = 0%						
Test for overall effect: Z = 1.86 (P = 0.06)						
**Total (95% CI)**		**2092**		**2119**	**100.0%**	**0.68 [0.56, 0.82]**
Total events	164		248			
Heterogeneity: Tau² = 0.00; Chi² = 4.80, df = 9 (P = 0.85); I² = 0%						
Test for overall effect: Z = 4.11 (P < 0.0001)						
Test for subgroup differences: Chi² = 0.30, df = 3 (P = 0.96), I² = 0%						

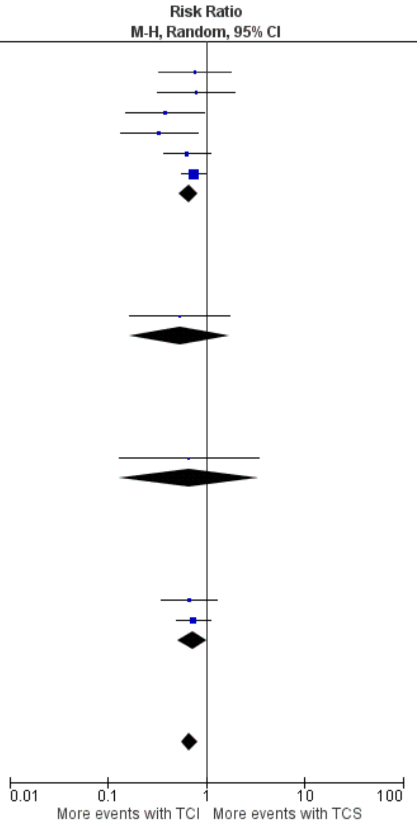

Skin infections

Study or Subgroup	Corticosteroids (TCS) Events	Total	Calcineurin inhibitor (TCI) Events	Total	Weight	Risk Ratio M-H, Random, 95% CI
**2.7.1 Potent**						
Doss 2010 (6)	49	239	44	239	14.6%	1.11 [0.77, 1.60]
Luger 2004 (1)	80	330	69	328	24.2%	1.15 [0.87, 1.53]
Mandelin 2010 (2)	17	40	26	40	10.7%	0.65 [0.43, 1.00]
Reitamo 2005 (3)	9	485	13	487	2.8%	0.70 [0.30, 1.61]
**Subtotal (95% CI)**		**1094**		**1094**	**52.2%**	**0.94 [0.70, 1.26]**
Total events	155		152			
Heterogeneity: Tau² = 0.04; Chi² = 5.90, df = 3 (P = 0.12); I² = 49%						
Test for overall effect: Z = 0.41 (P = 0.68)						
**2.7.2 Potent or mild potency**						
Hofman 2006 (9)	4	124	2	133	0.7%	2.15 [0.40, 11.51]
**Subtotal (95% CI)**		**124**		**133**	**0.7%**	**2.15 [0.40, 11.51]**
Total events	4		2			
Heterogeneity: Not applicable						
Test for overall effect: Z = 0.89 (P = 0.37)						
**2.7.3 Moderate or mild potency**						
Sigurgeirsson 2015 (4)	150	1213	157	1205	44.5%	0.95 [0.77, 1.17]
**Subtotal (95% CI)**		**1213**		**1205**	**44.5%**	**0.95 [0.77, 1.17]**
Total events	150		157			
Heterogeneity: Not applicable						
Test for overall effect: Z = 0.49 (P = 0.62)						
**2.7.4 Mild potency**						
Reitamo 2002(II) (11)	4	185	4	186	1.0%	1.01 [0.26, 3.96]
Reitamo 2004 (12)	6	207	6	210	1.6%	1.01 [0.33, 3.09]
**Subtotal (95% CI)**		**392**		**396**	**2.6%**	**1.01 [0.43, 2.40]**
Total events	10		10			
Heterogeneity: Tau² = 0.00; Chi² = 0.00, df = 1 (P = 0.99); I² = 0%						
Test for overall effect: Z = 0.02 (P = 0.98)						
**Total (95% CI)**		**2823**		**2828**	**100.0%**	**0.98 [0.85, 1.12]**
Total events	319		321			
Heterogeneity: Tau² = 0.00; Chi² = 6.80, df = 7 (P = 0.45); I² = 0%						
Test for overall effect: Z = 0.33 (P = 0.74)						
Test for subgroup differences: Chi² = 0.92, df = 3 (P = 0.82), I² = 0%						

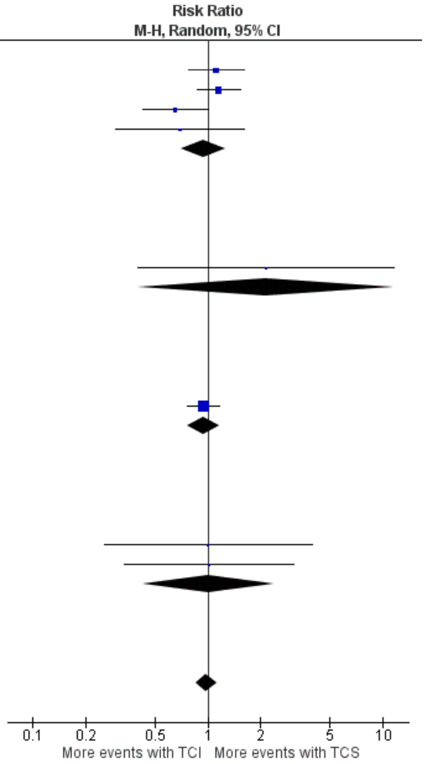

2

By age of participants (children or adults)

Skin thinning

Study or Subgroup	Corticosteroids (TCS) Events	Total	Calcineurin inhibitor (TCI) Events	Total	Weight	Risk Ratio M-H, Random, 95% CI
**3.6.1 Adults**						
Luger 2004 (1)	3	330	0	328	26.5%	6.96 [0.36, 134.17]
Mandelin 2010 (2)	2	40	0	40	25.7%	5.00 [0.25, 100.97]
Reitamo 2005 (3)	2	485	0	487	25.2%	5.02 [0.24, 104.30]
**Subtotal (95% CI)**		**855**		**855**	**77.3%**	**5.61 [0.99, 31.67]**
Total events	7		0			
Heterogeneity: Tau² = 0.00; Chi² = 0.03, df = 2 (P = 0.98); I² = 0%						
Test for overall effect: Z = 1.95 (P = 0.05)						
**3.6.2 Children**						
Sigurgeirsson 2015 (4)	1	1213	0	1205	22.7%	2.98 [0.12, 73.08]
**Subtotal (95% CI)**		**1213**		**1205**	**22.7%**	**2.98 [0.12, 73.08]**
Total events	1		0			
Heterogeneity: Not applicable						
Test for overall effect: Z = 0.67 (P = 0.50)						
**Total (95% CI)**		**2068**		**2060**	**100.0%**	**4.86 [1.06, 22.28]**
Total events	8		0			
Heterogeneity: Tau² = 0.00; Chi² = 0.15, df = 3 (P = 0.99); I² = 0%						
Test for overall effect: Z = 2.03 (P = 0.04)						
Test for subgroup differences: Chi² = 0.12, df = 1 (P = 0.73), I² = 0%						

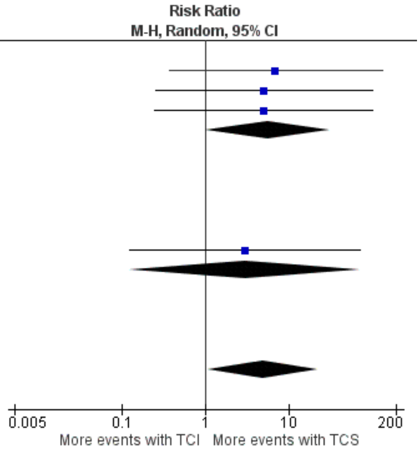

Skin burning

Study or Subgroup	Corticosteroids (TCS) Events	Total	Calcineurin inhibitor (TCI) Events	Total	Weight	Risk Ratio M-H, Random, 95% CI
**3.2.1 Adults**						
Doss 2009 (5)	9	279	47	287	9.1%	0.20 [0.10, 0.39]
Luger 2001 (7)	4	42	22	45	5.8%	0.19 [0.07, 0.52]
Luger 2004 (1)	36	330	85	328	15.6%	0.42 [0.29, 0.60]
Reitamo 2002(I) (8)	24	186	113	191	14.9%	0.22 [0.15, 0.32]
Reitamo 2005 (3)	67	485	255	487	18.4%	0.26 [0.21, 0.33]
**Subtotal (95% CI)**		**1322**		**1338**	**63.7%**	**0.27 [0.20, 0.36]**
Total events	140		522			
Heterogeneity: Tau² = 0.05; Chi² = 8.28, df = 4 (P = 0.08); I² = 52%						
Test for overall effect: Z = 9.17 (P < 0.00001)						
**3.2.2 Children**						
Doss 2010 (6)	6	239	18	237	6.4%	0.33 [0.13, 0.82]
Hofman 2006 (9)	2	124	10	133	2.9%	0.21 [0.05, 0.96]
Reitamo 2002(II) (11)	13	185	38	186	10.7%	0.34 [0.19, 0.62]
Reitamo 2004 (12)	30	207	50	210	14.5%	0.61 [0.40, 0.92]
Sikder 2005 (10)	1	15	7	15	1.8%	0.14 [0.02, 1.02]
**Subtotal (95% CI)**		**770**		**781**	**36.3%**	**0.40 [0.26, 0.62]**
Total events	52		123			
Heterogeneity: Tau² = 0.07; Chi² = 5.68, df = 4 (P = 0.22); I² = 30%						
Test for overall effect: Z = 4.17 (P < 0.0001)						
**Total (95% CI)**		**2092**		**2119**	**100.0%**	**0.31 [0.23, 0.40]**
Total events	192		645			
Heterogeneity: Tau² = 0.09; Chi² = 21.66, df = 9 (P = 0.01); I² = 58%						
Test for overall effect: Z = 8.44 (P < 0.00001)						
Test for subgroup differences: Chi² = 2.48, df = 1 (P = 0.11), I² = 59.8%						

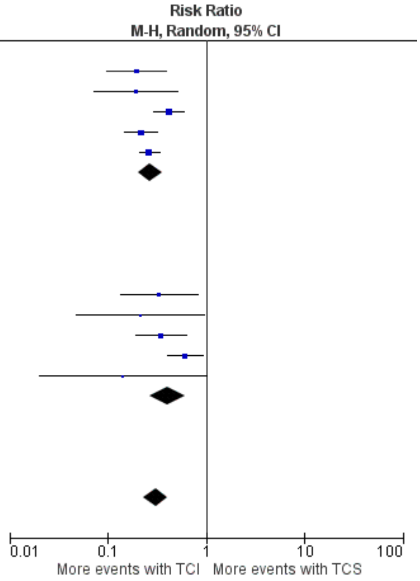

For peer review only - http://bmjopen.bmj.com/site/about/guidelines.xhtml

Pruritus

Study or Subgroup	Corticosteroids (TCS) Events	Total	Calcineurin inhibitor (TCI) Events	Total	Weight	Risk Ratio M-H, Random, 95% CI
**3.3.1 Adults**						
Doss 2009 (5)	9	279	12	287	4.7%	0.77 [0.33, 1.80]
Luger 2001 (7)	5	42	14	45	3.9%	0.38 [0.15, 0.97]
Luger 2004 (1)	6	330	18	328	4.1%	0.33 [0.13, 0.82]
Reitamo 2002(I) (8)	18	186	29	191	11.2%	0.64 [0.37, 1.11]
Reitamo 2005 (3)	65	485	88	487	39.3%	0.74 [0.55, 1.00]
**Subtotal (95% CI)**		**1322**		**1338**	**63.2%**	**0.65 [0.50, 0.84]**
Total events	103		161			
Heterogeneity: Tau² = 0.01; Chi² = 4.28, df = 4 (P = 0.37); I² = 7%						
Test for overall effect: Z = 3.34 (P = 0.0008)						
**3.3.2 Children**						
Doss 2010 (6)	8	239	10	237	4.1%	0.79 [0.32, 1.98]
Hofman 2006 (9)	4	124	8	133	2.5%	0.54 [0.17, 1.74]
Reitamo 2002(II) (11)	14	185	21	186	8.2%	0.67 [0.35, 1.28]
Reitamo 2004 (12)	33	207	45	210	20.7%	0.74 [0.50, 1.12]
Sikder 2005 (10)	2	15	3	15	1.3%	0.67 [0.13, 3.44]
**Subtotal (95% CI)**		**770**		**781**	**36.8%**	**0.71 [0.53, 0.97]**
Total events	61		87			
Heterogeneity: Tau² = 0.00; Chi² = 0.36, df = 4 (P = 0.99); I² = 0%						
Test for overall effect: Z = 2.17 (P = 0.03)						
**Total (95% CI)**		**2092**		**2119**	**100.0%**	**0.68 [0.56, 0.82]**
Total events	164		248			
Heterogeneity: Tau² = 0.00; Chi² = 4.80, df = 9 (P = 0.85); I² = 0%						
Test for overall effect: Z = 4.11 (P < 0.0001)						
Test for subgroup differences: Chi² = 0.23, df = 1 (P = 0.63), I² = 0%						

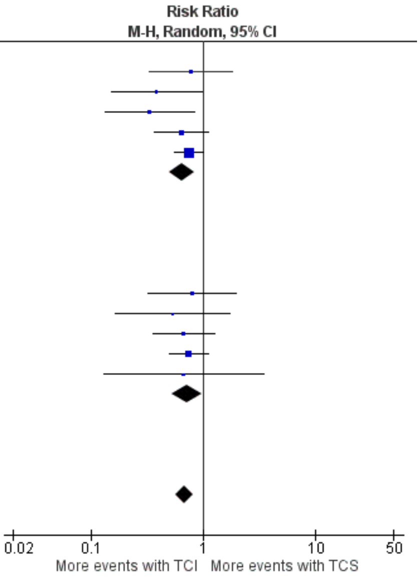

Skin infections

Study or Subgroup	Corticosteroids (TCS) Events	Total	Calcineurin inhibitor (TCI) Events	Total	Weight	Risk Ratio M-H, Random, 95% CI
**3.7.1 Adults**						
Luger 2004 (1)	80	330	69	328	24.2%	1.15 [0.87, 1.53]
Mandelin 2010 (2)	17	40	26	40	10.7%	0.65 [0.43, 1.00]
Reitamo 2005 (3)	9	485	13	487	2.8%	0.70 [0.30, 1.61]
**Subtotal (95% CI)**		**855**		**855**	**37.7%**	**0.85 [0.55, 1.32]**
Total events	106		108			
Heterogeneity: Tau² = 0.09; Chi² = 5.35, df = 2 (P = 0.07); I² = 63%						
Test for overall effect: Z = 0.70 (P = 0.48)						
**3.7.2 Children**						
Doss 2010 (6)	49	239	44	239	14.6%	1.11 [0.77, 1.60]
Hofman 2006 (9)	4	124	2	133	0.7%	2.15 [0.40, 11.51]
Reitamo 2002(II) (11)	4	185	4	186	1.0%	1.01 [0.26, 3.96]
Reitamo 2004 (12)	6	207	6	210	1.6%	1.01 [0.33, 3.09]
Sigurgeirsson 2015 (4)	150	1213	157	1205	44.5%	0.95 [0.77, 1.17]
**Subtotal (95% CI)**		**1968**		**1973**	**62.3%**	**1.00 [0.84, 1.19]**
Total events	213		213			
Heterogeneity: Tau² = 0.00; Chi² = 1.37, df = 4 (P = 0.85); I² = 0%						
Test for overall effect: Z = 0.04 (P = 0.97)						
**Total (95% CI)**		**2823**		**2828**	**100.0%**	**0.98 [0.85, 1.12]**
Total events	319		321			
Heterogeneity: Tau² = 0.00; Chi² = 6.80, df = 7 (P = 0.45); I² = 0%						
Test for overall effect: Z = 0.33 (P = 0.74)						
Test for subgroup differences: Chi² = 0.41, df = 1 (P = 0.52), I² = 0%						

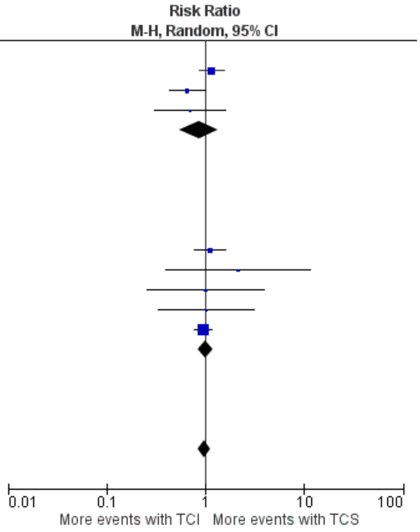

For peer review only - http://bmjopen.bmj.com/site/about/guidelines.xhtml

By individual topical calcineurin inhibitor (TCI)

Skin thinning

Study or Subgroup	Corticosteroids (TCS) Events	Corticosteroids (TCS) Total	Calcineurin inhibitor (TCI) Events	Calcineurin inhibitor (TCI) Total	Weight	Risk Ratio M-H, Random, 95% CI
**4.6.1 Tacrolimus 0.1%**						
Mandelin 2010 (2)	2	40	0	40	25.7%	5.00 [0.25, 100.97]
Reitamo 2005 (3)	2	485	0	487	25.2%	5.02 [0.24, 104.30]
**Subtotal (95% CI)**		**525**		**527**	**50.9%**	**5.01 [0.59, 42.37]**
Total events	4		0			
Heterogeneity: Tau² = 0.00; Chi² = 0.00, df = 1 (P = 1.00); I² = 0%						
Test for overall effect: Z = 1.48 (P = 0.14)						
**4.6.2 Pimecrolimus 1%**						
Luger 2004 (1)	3	330	0	328	26.5%	6.96 [0.36, 134.17]
Sigurgeirsson 2015 (4)	1	1213	0	1205	22.7%	2.98 [0.12, 73.08]
**Subtotal (95% CI)**		**1543**		**1533**	**49.1%**	**4.71 [0.54, 41.33]**
Total events	4		0			
Heterogeneity: Tau² = 0.00; Chi² = 0.15, df = 1 (P = 0.70); I² = 0%						
Test for overall effect: Z = 1.40 (P = 0.16)						
**Total (95% CI)**		**2068**		**2060**	**100.0%**	**4.86 [1.06, 22.28]**
Total events	8		0			
Heterogeneity: Tau² = 0.00; Chi² = 0.15, df = 3 (P = 0.99); I² = 0%						
Test for overall effect: Z = 2.03 (P = 0.04)						
Test for subgroup differences: Chi² = 0.00, df = 1 (P = 0.97), I² = 0%						

Risk Ratio
M-H, Random, 95% CI

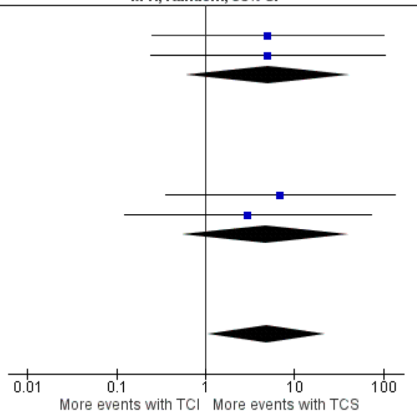

Skin burning

Study or Subgroup	Corticosteroids (TCS) Events	Corticosteroids (TCS) Total	Calcineurin inhibitor (TCI) Events	Calcineurin inhibitor (TCI) Total	Weight	Risk Ratio M-H, Random, 95% CI
**4.2.1 Tacrolimus 0.1%**						
Doss 2009 (5)	9	279	47	287	9.1%	0.20 [0.10, 0.39]
Reitamo 2002(I) (8)	24	186	113	191	14.9%	0.22 [0.15, 0.32]
Reitamo 2002(II) (11)	13	185	38	186	10.7%	0.34 [0.19, 0.62]
Reitamo 2005 (3)	67	485	255	487	18.4%	0.26 [0.21, 0.33]
**Subtotal (95% CI)**		**1135**		**1151**	**53.0%**	**0.25 [0.21, 0.31]**
Total events	113		453			
Heterogeneity: Tau² = 0.00; Chi² = 2.19, df = 3 (P = 0.53); I² = 0%						
Test for overall effect: Z = 14.49 (P < 0.00001)						
**4.2.2 Tacrolimus 0.03%**						
Doss 2010 (6)	6	239	18	237	6.4%	0.33 [0.13, 0.82]
Hofman 2006 (9)	2	124	10	133	2.9%	0.21 [0.05, 0.96]
Reitamo 2004 (12)	30	207	50	210	14.5%	0.61 [0.40, 0.92]
Sikder 2005 (10)	1	15	7	15	1.8%	0.14 [0.02, 1.02]
**Subtotal (95% CI)**		**585**		**595**	**25.6%**	**0.40 [0.22, 0.73]**
Total events	39		85			
Heterogeneity: Tau² = 0.13; Chi² = 4.60, df = 3 (P = 0.20); I² = 35%						
Test for overall effect: Z = 3.00 (P = 0.003)						
**4.2.3 Pimecrolimus 1%**						
Luger 2001 (7)	4	42	22	45	5.8%	0.19 [0.07, 0.52]
Luger 2004 (1)	36	330	85	328	15.6%	0.42 [0.29, 0.60]
**Subtotal (95% CI)**		**372**		**373**	**21.4%**	**0.33 [0.16, 0.67]**
Total events	40		107			
Heterogeneity: Tau² = 0.16; Chi² = 2.12, df = 1 (P = 0.15); I² = 53%						
Test for overall effect: Z = 3.08 (P = 0.002)						
**Total (95% CI)**		**2092**		**2119**	**100.0%**	**0.31 [0.23, 0.40]**
Total events	192		645			
Heterogeneity: Tau² = 0.09; Chi² = 21.66, df = 9 (P = 0.01); I² = 58%						
Test for overall effect: Z = 8.44 (P < 0.00001)						
Test for subgroup differences: Chi² = 2.43, df = 2 (P = 0.30), I² = 17.8%						

Risk Ratio
M-H, Random, 95% CI

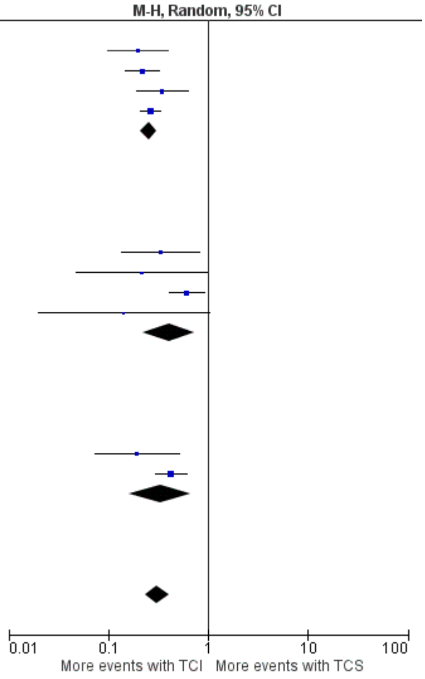

For peer review only - http://bmjopen.bmj.com/site/about/guidelines.xhtml

Pruritus

Study or Subgroup	Corticosteroids (TCS) Events	Total	Calcineurin inhibitor (TCI) Events	Total	Weight	Risk Ratio M-H, Random, 95% CI
**4.3.1 Tacrolimus 0.1%**						
Doss 2009 (5)	9	279	12	287	4.7%	0.77 [0.33, 1.80]
Reitamo 2002(I) (8)	18	186	29	191	11.2%	0.64 [0.37, 1.11]
Reitamo 2002(II) (11)	14	185	21	186	8.2%	0.67 [0.35, 1.28]
Reitamo 2005 (3)	65	485	88	487	39.3%	0.74 [0.55, 1.00]
**Subtotal (95% CI)**		**1135**		**1151**	**63.4%**	**0.71 [0.57, 0.90]**
Total events	106		150			
Heterogeneity: Tau² = 0.00; Chi² = 0.30, df = 3 (P = 0.96); I² = 0%						
Test for overall effect: Z = 2.84 (P = 0.005)						
**4.3.2 Tacrolimus 0.03%**						
Doss 2010 (6)	8	239	10	237	4.1%	0.79 [0.32, 1.98]
Hofman 2006 (9)	4	124	8	133	2.5%	0.54 [0.17, 1.74]
Reitamo 2004 (12)	33	207	45	210	20.7%	0.74 [0.50, 1.12]
Sikder 2005 (10)	2	15	3	15	1.3%	0.67 [0.13, 3.44]
**Subtotal (95% CI)**		**585**		**595**	**28.5%**	**0.73 [0.51, 1.03]**
Total events	47		66			
Heterogeneity: Tau² = 0.00; Chi² = 0.32, df = 3 (P = 0.96); I² = 0%						
Test for overall effect: Z = 1.81 (P = 0.07)						
**4.3.3 Pimecrolimus 1%**						
Luger 2001 (7)	5	42	14	45	3.9%	0.38 [0.15, 0.97]
Luger 2004 (1)	6	330	18	328	4.1%	0.33 [0.13, 0.82]
**Subtotal (95% CI)**		**372**		**373**	**8.1%**	**0.36 [0.19, 0.68]**
Total events	11		32			
Heterogeneity: Tau² = 0.00; Chi² = 0.05, df = 1 (P = 0.83); I² = 0%						
Test for overall effect: Z = 3.11 (P = 0.002)						
**Total (95% CI)**		**2092**		**2119**	**100.0%**	**0.68 [0.56, 0.82]**
Total events	164		248			
Heterogeneity: Tau² = 0.00; Chi² = 4.80, df = 9 (P = 0.85); I² = 0%						
Test for overall effect: Z = 4.11 (P < 0.0001)						
Test for subgroup differences: Chi² = 4.13, df = 2 (P = 0.13), I² = 51.6%						

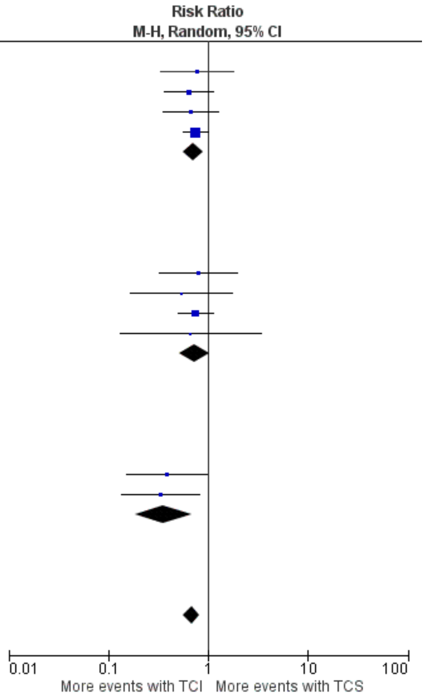

revi

Skin infections

Study or Subgroup	Corticosteroids (TCS) Events	Total	Calcineurin inhibitor (TCI) Events	Total	Weight	Risk Ratio M-H, Random, 95% CI
**4.7.1 Tacrolimus 0.1%**						
Mandelin 2010 (2)	17	40	26	40	10.7%	0.65 [0.43, 1.00]
Reitamo 2002(II) (11)	4	185	4	186	1.0%	1.01 [0.26, 3.96]
Reitamo 2005 (3)	9	485	13	487	2.8%	0.70 [0.30, 1.61]
**Subtotal (95% CI)**		**710**		**713**	**14.5%**	**0.68 [0.47, 0.98]**
Total events	30		43			
Heterogeneity: Tau² = 0.00; Chi² = 0.36, df = 2 (P = 0.83); I² = 0%						
Test for overall effect: Z = 2.05 (P = 0.04)						
**4.7.2 Tacrolimus 0.03%**						
Doss 2010 (6)	49	239	44	239	14.6%	1.11 [0.77, 1.60]
Hofman 2006 (9)	4	124	2	133	0.7%	2.15 [0.40, 11.51]
Reitamo 2004 (12)	6	207	6	210	1.6%	1.01 [0.33, 3.09]
**Subtotal (95% CI)**		**570**		**582**	**16.8%**	**1.13 [0.81, 1.59]**
Total events	59		52			
Heterogeneity: Tau² = 0.00; Chi² = 0.60, df = 2 (P = 0.74); I² = 0%						
Test for overall effect: Z = 0.73 (P = 0.47)						
**4.7.3 Pimecrolimus 1%**						
Luger 2004 (1)	80	330	69	328	24.2%	1.15 [0.87, 1.53]
Sigurgeirsson 2015 (4)	150	1213	157	1205	44.5%	0.95 [0.77, 1.17]
**Subtotal (95% CI)**		**1543**		**1533**	**68.7%**	**1.02 [0.85, 1.23]**
Total events	230		226			
Heterogeneity: Tau² = 0.00; Chi² = 1.17, df = 1 (P = 0.28); I² = 15%						
Test for overall effect: Z = 0.22 (P = 0.83)						
**Total (95% CI)**		**2823**		**2828**	**100.0%**	**0.98 [0.85, 1.12]**
Total events	319		321			
Heterogeneity: Tau² = 0.00; Chi² = 6.80, df = 7 (P = 0.45); I² = 0%						
Test for overall effect: Z = 0.33 (P = 0.74)						
Test for subgroup differences: Chi² = 4.65, df = 2 (P = 0.10), I² = 57.0%						

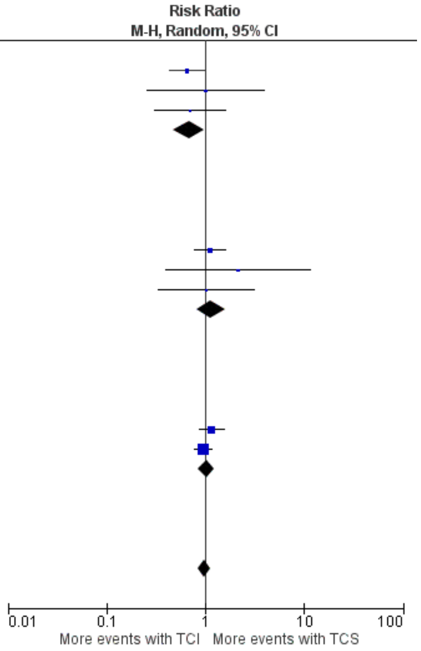

References:

1. Luger T, Lahfa M, Folster-Holst R, Gulliver W, Allen R, Molloy S, et al. Long-term safety and tolerability of pimecrolimus cream 1% and topical corticosteroids in adults with moderate to severe atopic dermatitis. *J Dermatolog Treat*. 2004;**15**(3):169-78
2. Mandelin J, Remitz A, Virtanen H, Reitamo S. One-year treatment with 0.1% tacrolimus ointment versus a corticosteroid regimen in adults with moderate to severe atopic dermatitis: A randomized, double-blind, comparative trial. *Acta Derm Venereol*. 2010;**90**(2):170-4
3. Reitamo S, Ortonne J, Sand C, Cambazard F, Bieber T, Folster-Holst R, et al. A multicentre, randomized, double-blind, controlled study of long-term treatment with 0.1% tacrolimus ointment in adults with moderate to severe atopic dermatitis. *Br J Dermatol*. 2005;**152**(6):1282-9
4. Sigurgeirsson B, Boznanski A, Todd G, Vertruyen A, Schuttelaar M, Zhu X, et al. Safety and efficacy of pimecrolimus in atopic dermatitis: a 5-year randomized trial. *Pediatrics* 2015;**135**(4):597-606
5. Doss N, Reitamo S, Dubertret L, Fekete G, Kamoun M, Lahfa M, et al. Superiority of tacrolimus 0.1% ointment compared with fluticasone 0.005% in adults with moderate to severe atopic dermatitis of the face: results from a randomized, double-blind trial. *Br J Dermatol*. 2009;**161**(2):427-34
6. Doss N, Kamoun M, Dubertret L, Cambazard F, Remitz A, Lahfa M, et al. Efficacy of tacrolimus 0.03% ointment as second-line treatment for children with moderate-to-severe atopic dermatitis: evidence from a randomized, double-blind non-inferiority trial vs. fluticasone 0.005% ointment. *Pediatr Allergy Immunol*. 2010;**21**(2 Pt 1):321-9.
7. Luger T, Van Leent E, Graeber M, Hedgecock S, Thurston M, Kandra A, et al. SDZ ASM 981: an emerging safe and effective treatment for atopic dermatitis. *Br J Dermatol*. 2001;**144**(4):788-94
8. Reitamo S, Rustin M, Ruzicka T, Cambazard F, Kalimo K, Friedmann P, et al. Efficacy and safety of tacrolimus ointment compared with that of hydrocortisone butyrate ointment in adult patients with atopic dermatitis. *J Allergy Clin Immunol*. 2002;**109(**3):547-55
9. Hofman T, Cranswick N, Kuna P, Boznanski A, Latos T, Gold M, et al. Tacrolimus ointment does not affect the immediate response to vaccination, the generation of immune memory, or humoral and cell-mediated immunity in children. *Arch Dis Child*. 2006;**91**(11):905-10
10. Sikder M, Al Mamun S, Khan R, Chowdhury A, Khan H, Hoque M. Topical 0.03% tacrolimus ointment, 0.05% clobetasone butyrate cream alone and their combination in older children with atopic dermatitis - An open randomized comparative study. *J Pak Assoc Dermatol*. 2005;**15**(4):304-12
11. Reitamo S, Van Leent E, Ho V, Harper J, Ruzicka T, Kalimo K, et al. Efficacy and safety of tacrolimus ointment compared with that of hydrocortisone acetate ointment in children with atopic dermatitis. *J Allergy Clin Immunol*. 2002;**109**(3):539-46
12. Reitamo S, Harper J, Bos J, Cambazard F, Bruijnzeel-Koomen C, Valk P, et al. 0.03% Tacrolimus ointment applied once or twice daily is more efficacious than 1% hydrocortisone acetate in children with moderate to severe atopic dermatitis: results of a randomized double-blind controlled trial. *Br J Dermatol*. 2004;**150**(3):554-62

For peer review only - http://bmjopen.bmj.com/site/about/guidelines.xhtml

PRISMA 2009 Checklist

Section/topic	#	Checklist item	Reported on page #
**TITLE**			
Title	1	Identify the report as a systematic review, meta-analysis, or both.	1,3
**ABSTRACT**			
Structured summary	2	Provide a structured summary including, as applicable: background; objectives; data sources; study eligibility criteria, participants, and interventions; study appraisal and synthesis methods; results; limitations; conclusions and implications of key findings; systematic review registration number.	3
**INTRODUCTION**			
Rationale	3	Describe the rationale for the review in the context of what is already known.	5
Objectives	4	Provide an explicit statement of questions being addressed with reference to participants, interventions, comparisons, outcomes, and study design (PICOS).	5,6
**METHODS**			
Protocol and registration	5	Indicate if a review protocol exists, if and where it can be accessed (e.g., Web address), and, if available, provide registration information including registration number.	6
Eligibility criteria	6	Specify study characteristics (e.g., PICOS, length of follow-up) and report characteristics (e.g., years considered, language, publication status) used as criteria for eligibility, giving rationale.	6
Information sources	7	Describe all information sources (e.g., databases with dates of coverage, contact with study authors to identify additional studies) in the search and date last searched.	6
Search	8	Present full electronic search strategy for at least one database, including any limits used, such that it could be repeated.	Appendix 1
Study selection	9	State the process for selecting studies (i.e., screening, eligibility, included in systematic review, and, if applicable, included in the meta-analysis).	6,7
Data collection process	10	Describe method of data extraction from reports (e.g., piloted forms, independently, in duplicate) and any processes for obtaining and confirming data from investigators.	7
Data items	11	List and define all variables for which data were sought (e.g., PICOS, funding sources) and any assumptions and simplifications made.	7
Risk of bias in individual studies	12	Describe methods used for assessing risk of bias of individual studies (including specification of whether this was done at the study or outcome level), and how this information is to be used in any data synthesis.	7
Summary measures	13	State the principal summary measures (e.g., risk ratio, difference in means).	7,8
Synthesis of results	14	Describe the methods of handling data and combining results of studies, if done, including measures of consistency (e.g., I^2) for each meta-analysis.	7

For peer review only - http://bmjopen.bmj.com/site/about/guidelines.xhtml

PRISMA 2009 Checklist

Section/topic	#	Checklist item	Reported on page #
Risk of bias across studies	15	Specify any assessment of risk of bias that may affect the cumulative evidence (e.g., publication bias, selective reporting within studies).	7
Additional analyses	16	Describe methods of additional analyses (e.g., sensitivity or subgroup analyses, meta-regression), if done, indicating which were pre-specified.	7
**RESULTS**			
Study selection	17	Give numbers of studies screened, assessed for eligibility, and included in the review, with reasons for exclusions at each stage, ideally with a flow diagram.	9 & fig 1
Study characteristics	18	For each study, present characteristics for which data were extracted (e.g., study size, PICOS, follow-up period) and provide the citations.	10-14 & appendix 6
Risk of bias within studies	19	Present data on risk of bias of each study and, if available, any outcome level assessment (see item 12).	10-14, 24-25 & appendix 4
Results of individual studies	20	For all outcomes considered (benefits or harms), present, for each study: (a) simple summary data for each intervention group (b) effect estimates and confidence intervals, ideally with a forest plot.	10-14 & appendix 7,8
Synthesis of results	21	Present results of each meta-analysis done, including confidence intervals and measures of consistency.	10-14
Risk of bias across studies	22	Present results of any assessment of risk of bias across studies (see Item 15).	10-14, 24-25 & appendix 4
Additional analysis	23	Give results of additional analyses, if done (e.g., sensitivity or subgroup analyses, meta-regression [see Item 16]).	10-14 & appendix 9
**DISCUSSION**			
Summary of evidence	24	Summarize the main findings including the strength of evidence for each main outcome; consider their relevance to key groups (e.g., healthcare providers, users, and policy makers).	14
Limitations	25	Discuss limitations at study and outcome level (e.g., risk of bias), and at review-level (e.g., incomplete retrieval of identified research, reporting bias).	14, 15

For peer review only

PRISMA 2009 Checklist

Conclusions	26	Provide a general interpretation of the results in the context of other evidence, and implications for future research.	15
**FUNDING**			
Funding	27	Describe sources of funding for the systematic review and other support (e.g., supply of data); role of funders for the systematic review.	1

From: Moher D, Liberati A, Tetzlaff J, Altman DG, The PRISMA Group (2009). Preferred Reporting Items for Systematic Reviews and Meta-Analyses: The PRISMA Statement. PLoS Med 6(7): e1000097. doi:10.1371/journal.pmed1000097

For more information, visit: **www.prisma-statement.org**.

For peer review only

For peer review only - http://bmjopen.bmj.com/site/about/guidelines.xhtml

BMJ Open

Safety of topical corticosteroids in atopic eczema; an umbrella review

Journal:	*BMJ Open*
Manuscript ID	bmjopen-2020-046476.R1
Article Type:	Original research
Date Submitted by the Author:	12-Apr-2021
Complete List of Authors:	Axon, Emma; University of Nottingham, Centre of Evidence Based Dermatology<br>Chalmers, Joanne; University of Nottingham, Centre of Evidence Based Dermatology<br>Santer, Miriam; University of Southampton, Primary Care & Population Sciences<br>Ridd, Matthew; University of Bristol Faculty of Health Sciences, Population Health Sciences<br>Lawton, Sandra; Rotherham NHS Foundation Trust, Dermatology Department<br>Langan, Sinead; London School of Hygiene & Tropical Medicine,<br>Grindlay, Douglas; University of Nottingham, Centre of Evidence Based Dermtology<br>Muller, Ingrid; University of Southampton, School of Primary Care, Population Health and Medical Education<br>Roberts, Amanda; University of Nottingham, Centre of Evidence-Based Dermatology<br>Ahmed, Amina; University of Nottingham, Centre of Evidence Based Dermatology<br>Williams, HC; University of Nottingham, Centre of Evidence Based Dermatology<br>Thomas, Kim; University of Nottingham, Centre of Evidence Based Dermatology
**Primary Subject Heading**:	Dermatology
Secondary Subject Heading:	Dermatology
Keywords:	Eczema < DERMATOLOGY, Paediatric dermatology < PAEDIATRICS, Adult dermatology < DERMATOLOGY

SCHOLARONE™
Manuscripts

I, the Submitting Author has the right to grant and does grant on behalf of all authors of the Work (as defined in the below author licence), an exclusive licence and/or a non-exclusive licence for contributions from authors who are: i) UK Crown employees; ii) where BMJ has agreed a CC-BY licence shall apply, and/or iii) in accordance with the terms applicable for US Federal Government officers or employees acting as part of their official duties; on a worldwide, perpetual, irrevocable, royalty-free basis to BMJ Publishing Group Ltd ("BMJ") its licensees and where the relevant Journal is co-owned by BMJ to the co-owners of the Journal, to publish the Work in this journal and any other BMJ products and to exploit all rights, as set out in our licence.

The Submitting Author accepts and understands that any supply made under these terms is made by BMJ to the Submitting Author unless you are acting as an employee on behalf of your employer or a postgraduate student of an affiliated institution which is paying any applicable article publishing charge ("APC") for Open Access articles. Where the Submitting Author wishes to make the Work available on an Open Access basis (and intends to pay the relevant APC), the terms of reuse of such Open Access shall be governed by a Creative Commons licence – details of these licences and which Creative Commons licence will apply to this Work are set out in our licence referred to above.

Other than as permitted in any relevant BMJ Author's Self Archiving Policies, I confirm this Work has not been accepted for publication elsewhere, is not being considered for publication elsewhere and does not duplicate material already published. I confirm all authors consent to publication of this Work and authorise the granting of this licence.

For peer review only

Safety of topical corticosteroids in atopic eczema; an umbrella review

Dr Emma Axon (ORCID ID: 0000-0002-3246-9968)
Systematic Review Methodologist and Research Associate
Centre of Evidence Based Dermatology, School of Medicine, University of Nottingham, Kings Meadow Campus, Nottingham, UK, NG7 2NR,
Dr Joanne R Chalmers (ORCID ID: 0000-0002-2281-7367)
Senior Research Fellow, Centre of Evidence Based Dermatology, School of Medicine, University of Nottingham, Kings Meadow Campus, Nottingham, UK, NG7 2NR.
Dr Miriam Santer (ORCID ID: 0000-0001-7264-5260)
Associate Professor in Primary Care Research, Primary Care, Population Sciences & Medical Education, University of Southampton, Southampton, SO16 5ST, UK,
Dr Matthew J Ridd (ORCID ID: 0000-0002-7954-8823)
GP and Reader in Primary Healthcare, Population Health Sciences, Bristol Medical School, University of Bristol, Canynge Hall, 39 Whatley Road, Bristol, BS8 2PS, UK.
Mrs Sandra Lawton (ORCID ID: 0000-0002-6163-5822)
Nurse Consultant and Clinical Lead Dermatology, Rotherham NHS Foundation Trust, Moorgate Road, Rotherham, S60 2UD, UK
Professor Sinéad M Langan (ORCID ID: 0000-0002-7022-7441)
Wellcome Senior Clinical Fellow and Professor**,** London School of Hygiene and Tropical Medicine, Faculty of Epidemiology and Population Health, Room 250, Keppel Street, London, UK WC1E 7HT.
Dr Douglas J Grindlay (ORCID ID: 0000-0002-0992-7182)
Information Specialist, Centre of Evidence Based Dermatology, School of Medicine, University of Nottingham, Kings Meadow Campus, Nottingham, UK, NG7 2NR.
Dr Ingrid Muller (ORCID ID: 0000-0001-9341-6133)
Health Psychologist, Primary Care, Population Sciences & Medical Education, University of Southampton, Southampton, SO16 5ST, UK.
Mrs Amanda Roberts (ORCID ID: 0000-0003-0370-3695)
Patient and public involvement representative, Centre of Evidence Based Dermatology, School of Medicine, University of Nottingham, Kings Meadow Campus, Nottingham, UK, NG7 2NR.
Mrs Amina Ahmed (ORCID ID: 0000-0001-9494-742X)
Patient and public involvement representative, Centre of Evidence Based Dermatology, School of Medicine, University of Nottingham, Kings Meadow Campus, Nottingham, UK, NG7 2NR.
Professor Hywel C Williams (ORCID ID: 0000-0002-5646-3093)
Professor of Dermato-Epidemiology, Centre of Evidence Based Dermatology, School of Medicine, University of Nottingham, Kings Meadow Campus, Nottingham, UK, NG7 2NR.
Professor Kim S Thomas (ORCID ID: 0000-0001-7785-7465)
Professor of Applied Dermatology Research, Centre of Evidence Based Dermatology, School of Medicine, University of Nottingham, Kings Meadow Campus, Nottingham, UK, NG7 2NR.

Corresponding author: Dr Emma Axon, Centre of Evidence Based Dermatology, University of Nottingham, King's Meadow Campus, Nottingham, NG7 2NR. emma.axon@nottingham.ac.uk. +44 (0) 115 82 32435

Funding: This report presents independent research funded by the National Institute for Health Research (NIHR) under its Programme Grants for Applied Research programme (grant ref No. RP-PG-0216-20007). The views expressed are those of the author(s) and not necessarily those of the NHS, the NIHR or the department of Health and Social Care.

Competing interests: Authors are co-applicants on a NIHR Programme Grants for Applied Research (P-PG-0216-20007) which funded this overview. The aim of the Programme Grant is to develop an

For peer review only - http://bmjopen.bmj.com/site/about/guidelines.xhtml

intervention to support eczema self-care and the results of this overview will contribute to this intervention. MJR is funded by an NIHR Post-Doctoral Research Fellowship (PDF-2014-07-013). SML is supported by a Wellcome Senior Clinical fellowship in Science (205039/Z/16/Z). HCW was an author on four included reviews, and KST was an author on one included review.

Data availability: All data relevant to the study are included in the article or uploaded as supplementary information. For any further details email cebd@nottingham.ac.uk.

Word count: abstract 300 words, main text 3903 words

For peer review only

For peer review only - http://bmjopen.bmj.com/site/about/guidelines.xhtml

Abstract

Objective: An umbrella review summarising all safety data from systematic reviews of topical corticosteroids (TCS) in adults and children with atopic eczema .

Methods: Embase, MEDLINE, PubMed, Cochrane Database of Systematic Reviews and the Centre of Evidence Based Dermatology map of eczema systematic reviews searched until 7th November 2018 and Epistemonikos until 2nd March 2021. Reviews were included if they assessed safety of TCS in atopic eczema and searched ≥1 database using a reproducible search strategy. Review quality assessed using AMSTAR-2. PROSPERO registration: CRD42018079409.

Results: 38 systematic reviews included, 34 low/critically low quality. Treatment and follow-up usually short (2-4 weeks). Key findings:

TCS versus emollient/vehicle: No meta-analyses identified for skin-thinning. Two 2-week randomised controlled trials (RCTs) found no significant increased risk with very potent TCS (0/196 TCS vs 0/33 vehicle in children and 6/109 TCS vs 2/50 vehicle, age unknown). Biochemical adrenal suppression (cortisol) was 3.8% (95% CI 2.4%-5.8%) in a meta-analysis of 11 uncontrolled observational studies (any potency TCS, 522 children). Effects reversed when treatment ceased.

TCS versus topical calcineurin inhibitors (TCIs): Meta-analysis showed higher relative risk of skin-thinning with TCS (RR 4.86, 95% CI 1.06-22.28, n=4128, four RCTs, including one 5-year RCT). Eight cases in 2068 participants, 7 using potent TCS. No evidence of growth suppression.

Once daily versus more frequent TCS: No meta-analyses identified. No skin-thinning in one RCT (3 weeks potent TCS, n=94) or biochemical adrenal suppression in two RCTs (up to 2 weeks very potent/moderate TCS, n=129).

For peer review only

TCS twice/week to prevent flares ('weekend therapy') versus vehicle: No meta-analyses identified. No evidence of skin-thinning in five RCTs. One RCT found biochemical adrenal suppression (2/44 children, potent TCS).

Conclusions: We found no evidence of harm when TCS used intermittently "as required" to treat flares or "weekend therapy" to prevent flares. However, long-term safety data was limited.

Strengths and limitations of this overview

- Robust Cochrane methodology was followed and a thorough and inclusive literature search was performed to ensure this was a comprehensive overview.
- By extracting data from existing reviews, results are limited to topics for which there is an eligible systematic review.
- Safety was usually reported in less detail than effectiveness in systematic reviews limiting the available data for extraction, therefore potentially missing data included in the original papers.
- Most included reviews were rated low or critically low-quality using AMSTAR 2, and where quality of evidence assessments was reported for individual studies most indicated a high or unclear risk in at least one domain.
- Many RCTs were only short duration (2-4 weeks) limiting our ability to assess side-effects that take longer to develop such as skin thinning.

Keywords:

Atopic eczema, topical corticosteroids, adverse events

Abbreviations:

AMSTAR 2 = A MeaSurement Tool to Assess systematic Reviews 2

CI = Confidence interval

GRADE = Grading of Recommendations, Assessment, Development and Evaluation

NHS = National Health Service

For peer review only - http://bmjopen.bmj.com/site/about/guidelines.xhtml

RCT = Randomised controlled trial

RR = Relative risk

TCI = Topical calcineurin inhibitors

TCS = Topical corticosteroids

UK = United Kingdom

USA = United States of America

Introduction

Atopic eczema (also known as atopic dermatitis, or eczema) is an itchy inflammatory skin condition. It is most common in children with 1 in 5 affected worldwide [1, 2], but often persists into adulthood. [3]

Topical corticosteroids (TCS) are first line therapy for treating inflammatory eczema flares but widespread concerns regarding their safety amongst patients and healthcare professionals contribute to poor adherence, and subsequent worsening of disease control and quality of life. [4, 5] Safety concerns include skin thinning and retardation of growth and development. These concerns are thought to mainly originate from what is now considered to be inappropriate use, such as using potent TCS on the face or continual long-term use. [6] Strategies recommended to minimise exposure to TCS, and hence the risk of adverse events, include reducing frequency of application to once daily during treatment of an inflammatory episode, or TCS used for two consecutive days a week (sometimes referred to as "weekend therapy") as a strategy to prevent flares. [7-9] This umbrella review aims to assess safety (local and systemic adverse events) of TCS compared to other topical treatments, placebo or no comparator in people of any age and gender with atopic eczema, and addressed two areas of research prioritised in the James Lind Alliance priority setting partnership for atopic eczema. [10]

For peer review only - http://bmjopen.bmj.com/site/about/guidelines.xhtml

Methods

Protocol, registration, and study design

This umbrella review includes published systematic reviews of randomised controlled trials (RCTs) and/or observational studies reporting adverse event data in people with eczema using TCS. The aim of this overview was to summarise data from existing reviews, therefore, meta-analyses and data from individual studies were extracted and presented in this overview in the format and completeness that they were presented in the original systematic reviews. The only exception was for missing p values which were calculated where appropriate. The checklist 'Preferred Reporting Items for overview of systematic reviews' was followed [11], and the protocol was registered on PROSPERO (CRD42018079409). [12]

Search strategy

Embase, MEDLINE, PubMed, Cochrane Database of Systematic Reviews and Epistemonikos were searched from inception to 7th November 2018 by DG (Information Specialist), with no restrictions on language or publication date. The search strategy is in Appendix 1. The Epistemonikos search was updated on 2nd March 2021, with a publication date restricted to 2018 to 2021. Epistemonikos is now well-established as a comprehensive database of reviews that regularly searches ten major databases including the Cochrane Library, PubMed and Embase [13] thus making the need to search these individual databases redundant. We also checked the Centre of Evidence Based Dermatology eczema map of systematic reviews, [14] and searched PROSPERO up to 23rd March 2021 for any relevant ongoing systematic reviews using the terms "eczema" and "dermatitis".

Eligibility criteria

We included systematic reviews that presented data on the safety of TCS used to treat people of any age and gender with atopic eczema, had clinical outcomes, searched at least one database and provided a reproducible search strategy. Systematic reviews of any types of clinical study design were included. Multiple reviews on the same topic were included, except for "abridged" versions of the same review where no additional data were reported. To avoid duplication of data, for each

For peer review only - http://bmjopen.bmj.com/site/about/guidelines.xhtml

comparison, the review that included the highest number of studies on that comparison and therefore appeared the most comprehensive was taken as the primary review and other included reviews were checked for additional studies and data. Conference abstracts were excluded. Reviews that covered multiple skin conditions were only included if they reported data on atopic eczema patients separately.

Interventions and Control

Our intervention of interest was any TCS of any preparation and potency used to treat atopic eczema. For RCTs, the comparisons of interest were any other TCS, the same TCS used in a different way, another topical anti-inflammatory treatment, vehicle, no treatment or a combination of any of these. Comparisons with non-topical treatments were excluded as we were interested in clinical practice decisions regarding alternatives to TCS.

Outcomes

Safety outcomes reported during treatment and follow-up were extracted where reported in the reviews on immediate cutaneous adverse events (e.g. burning sensation/stinging), other cutaneous adverse events (e.g. skin thinning, telangiectasia, skin infections, folliculitis), systemic adverse events (e.g. effects on endocrine system, impact on growth) and rebound symptoms/steroid withdrawal.

Selection of studies and data extraction

Records identified from the database searches were uploaded into Covidence© (Veritas Health Innovation, Australia). [15] Two authors (EA and JRC) independently assessed the eligibility of each record, and where unclear the full text was obtained. The number of included and excluded records along with reasons for exclusion were reported in a Preferred Reporting Items for Systematic Reviews and Meta-Analyses (PRISMA) flow diagram.

Two authors (EA and JRC) independently extracted all safety data presented in the included reviews along with other information such as review/participant characteristics, and funding sources). Any disagreements regarding eligibility or data extraction were resolved via discussion or input from a

For peer review only - http://bmjopen.bmj.com/site/about/guidelines.xhtml

For peer review only

third reviewer (HCW or KST). Where available, we will report results separately for age, filaggrin mutation status, TCS potency, site of application of the TCS, and duration of continuous treatment.

Assessment of quality of included systematic reviews

As this was an overview of reviews, the methodological quality of the evidence was assessed at the systematic review level using the AMSTAR 2 tool and this was conducted in duplicate by EA and JC.[16] Reviews were considered critically low quality if there was more than one critical flaw. Data on the quality of individual studies (e.g. risk of bias) and the quality of evidence (e.g. GRADE[17]) were also extracted where presented in the review, but undertaking these quality assessments for individual studies was not within the remit of this overview.

Measures of treatment effect and data synthesis

Where relevant meta-analyses were presented in the systematic review, the forest plots, relative risk (RR) and 95% confidence intervals (CI) were extracted. In the absence of any meta-analysis, adverse event data from individual studies were included in this overview based on the data presented in the published systematic review. P values were calculated using Review Manager software, [18] with <0.05 indicating statistically significant results. Where meta-analyses were presented, we assessed the following subgroups where possible: age, TCS potency, anatomical site, treatment duration, and genetic predisposition to a disrupted skin barrier (filaggrin status). TCS potency was determined using a hierarchy of sources: UK 'British National Formulary', World Health Organization, and USA classifications. [19-21] An NHS classification ranging from very common (>1 in 10 people affected) to very rare (< 1 in 10,000) was used to narratively describe the absolute risk of each adverse event. [22]

Patient and public involvement

People with eczema and parents of children with eczema have been involved the decision to conduct this overview and in the design. The James Lind Alliance priority setting partnership for atopic eczema involved people with eczema and parents of children with eczema in which two of the

For peer review only - http://bmjopen.bmj.com/site/about/guidelines.xhtml

identified priority areas were around research into the safety of TCS. [10] Two of the overview authors are patient representatives (AR, AA) and both have been involved in the design of this overview and interpretation of the findings.

Wider patient and parent involvement has been particularly important in identifying important safety outcomes for this overview. We held a workshop involving five patient representatives in which the proposed overview was discussed which highlighted the need to seek out data on long-term TCS use, reversibility of any side effects and TCS withdrawal symptoms. We supplemented this with a survey about safety concerns with TCS at a National Eczema Society meeting of thirty-one people with eczema or parents of children with eczema and a published qualitative study of patient concerns relating to TCS safety. [6]

Dissemination of the results are underway as part of the wider programme of research of which this overview is a part and our patient representatives are a key stakeholder in this activity.

Results

Search results

After de-duplication, 635 records were screened; 127 records underwent full text screening and 38 systematic reviews met the inclusion criteria (Figure 1). [(7, 8, 23-56)] The list of excluded reviews is in Appendix 2. The search of PROSPERO identified five ongoing systematic reviews (Appendix 3). [(57-61)]

Characteristics and quality of the included systematic reviews

All but three reviews were published in English. Two Chinese reviews and one German review were translated into English. [32, 36, 45] Thirty of the included reviews were rated critically low quality according to AMSTAR-2; with four low, two moderate, and two high quality (Table 1). The most common reasons for downgrading were no protocol, no list of full text exclusions or a literature search restricted to the English language.

For peer review only - http://bmjopen.bmj.com/site/about/guidelines.xhtml

The included reviews identified 106 studies (77 RCTs and 29 observational studies) that included relevant safety data. Risk of bias assessments were available from the reviews for 63 RCTs, of which 42 used the Cochrane risk of bias tool. Most of these assessments rated at least one domain as high or unclear risk, most noticeably selection bias from lack of allocation concealment, performance bias due to lack of blinding of participants and detection bias due to lack of blinding of outcome assessors. The trials included in the reviews usually evaluated use of short bursts of TCS (2-4 weeks) to treat the flare but varied greatly in length of follow-up. Around two thirds of trials included no post-treatment follow-up, whilst the remainder included several weeks/months of follow-up generally using TCS intermittently “as required”. A total of 14 RCTs (5,874 participants) and 5 cohort/observational studies (4,438,698 participants) out of a total of 106 studies included follow up of more than 3 months. One notable trial (the ‘PETITE’ study) had 5 years follow-up with TCS used “as required”. [62]

Characteristics and quality assessments of each systematic review are in Table 1, with further detail in Appendix 4 and 5. Individual study data and quality assessments are in Appendix 6.

Safety of TCS compared to other topical treatments or corticosteroids

How safe are TCS compared to emollient or vehicle, or no comparison?

Thirteen reviews provided data on this comparison: one high [54], two low, [42, 47] and ten critically low quality. [25, 28, 31, 35-37, 50, 51, 55, 63] Key results can be found in Table 2 and additional data in Appendix 6.

Reported rates of skin thinning in RCTs were generally very low, with no significant increases seen with TCS compared to emollient/vehicle. No skin thinning or telangiectasia was reported in an RCT, 196 participants aged ≥12 years old using very potent TCS twice a day for 2 weeks compared to 50 using vehicle. [64] Another RCT reported skin thinning in 6/109 participants using very potent TCS for 2 weeks compared to 2/50 using vehicle, p=0.69. [65]

For peer review only - http://bmjopen.bmj.com/site/about/guidelines.xhtml

No significant differences in other cutaneous adverse events, such as hypopigmentation, were observed between treatments in five RCTs, and event rates were low. [66-70]

A meta-analysis[55] of 11 uncontrolled observational studies (up to 4 weeks of treatment) reported biochemical adrenal suppression (cortisol levels) in 20/522 children (3.8%, 95% CI 2.4% to 5.8%) with any potency TCS. [71-81] This was 2% (3/148 children) when only mild potency TCS were analysed. [72, 74, 77, 79] No clinical symptoms or signs of adrenal suppression were observed[71-81], and the biochemical effects were transient, with cortisol levels returning to normal after TCS were discontinued. [71, 75, 77, 78, 81]

Two included reviews assessed TCS withdrawal symptoms, mostly from case reports, but no incidence data were reported. [41, 44]

How safe are TCS compared to topical calcineurin inhibitors (TCI)?

Eight systematic reviews were identified: one moderate [23], one low [48], and six critically low quality. [27, 30, 43, 50, 52] [82] Most RCTs used twice daily TCS to treat the current flare (up to 3 weeks), and where longer-term follow-up was included, TCS were used “as required” to treat flares. Key results can be found in Table 2 and additional data in Appendix 6.

Meta-analyses of cutaneous adverse events were presented in two reviews. [27] [82] so the more comprehensive review was used to extract the cutaneous adverse event data [27]Some minor modifications were made to the data for this overview shown in Appendix 7. A meta-analysis of four RCTs (26 weeks to 5 years duration, twice a day or “as directed”) showed a significant increase in the relative risk of skin thinning with TCS compared with TCIs (0.1% tacrolimus or 1% pimecrolimus) (RR 4.86, 95% CI 1.06 to 22.28, p=0.04, n=4128). However, skin thinning was uncommon: 8/2068 participants (0.4%) with TCS versus 0/2060 (0%) with TCIs. Of the eight cases of skin thinning, seven were reported when using potent TCS and one using mild/moderate TCS. [62, 83-85]

For peer review only - http://bmjopen.bmj.com/site/about/guidelines.xhtml

The relative risk of skin burning and pruritus (itching) was significantly lower with TCS compared to TCIs (1% pimecrolimus or 0.1% / 0.03% tacrolimus) in meta-analyses of 10 RCTs in 4211 participants (skin burning: RR 0.31, 95% CI 0.23 to 0.40, p<0.00001; pruritus: RR 0.68, 95% CI 0.56 to 0.82, p <0.0001***).*** [83, 85-93] The GRADE assessments for these two adverse events indicated these were of moderate quality. [82] There was no significant difference in skin infections with potent, moderate or mild potency TCS compared to TCIs (1% pimecrolimus or 0.1% / 0.03% tacrolimus) [62, 83-86, 88, 90, 92] or erythema compared with 0.1% tacrolimus (Appendix 8). [91, 92]

Subgroup analyses of age, TCS potency and specific TCI showed no significant differences for any comparison (Appendix 9). We were unable to undertake any further subgroup analyses.

No differences in growth were observed in one 5-year RCT ('PETITE' study) in 2418 young children using moderate/mild potency TCS compared to those using TCI (1% pimecrolimus) (rates not given) and no cases of lymphoma were reported. [62] A large cohort study (n=1,438,333) showed a small non-significant increased risk of lymphoma with TCI and TCS compared to the general population, with a similar risk between treatments. [94] In addition, one case-control study (294 cases/293,000 controls) found no increased risk of lymphoma with TCS or TCI compared to controls. [95]

Is there any difference in safety of TCS of different potencies?

Six reviews compared the safety of different potency TCS: two low,[42, 47] and four critically low quality. [28, 34, 50, 53] RCTs were mainly short-term use of TCS (2-3 weeks), used once or twice daily. Results can be found in Appendix 6.

One RCT reported mild skin thinning in 4/13 children using potent TCS for up to 6 weeks compared with 2/12 using mild TCS (p=0.42), [96] whilst another RCT in 37 children found no evidence of skin thinning with mild or moderate potency TCS for 3 weeks. [97] One study compared 3 weeks of potent and moderate TCS in 40 children and reported "some" biochemical adrenal suppression (cortisol levels) but no numerical data were provided. [98]

For peer review only - http://bmjopen.bmj.com/site/about/guidelines.xhtml

How safe are TCS compared to topically applied Chinese herbal medicine?

Two systematic reviews provided data on TCS compared with topical Chinese herbal medicine: one high quality [(40)] and one critically low. [(39)] Results can be found in Appendix 6.

A meta-analysis of two RCTs [(99, 100)] was presented in two systematic reviews. [(39, 40)] More cutaneous adverse events, including application site burning, were observed with 2 weeks of very potent/potent TCS compared to topical Chinese herbal medicine (RR 12.03, 95% CI 1.59 to 91.26, p=0.02; 11/147 versus 0/148 participants). One additional RCT, including 95 young children, reported minor adverse events such as burning with 2 weeks of potent TCS but no numerical data were presented. [(101)]

Safety of different strategies for using TCS

How safe are once daily TCS compared with more frequent application?

Two low quality reviews provided safety data relating to different frequency of application. [(7, 47)] Key results can be found in Table 2 and additional data in Appendix 6.

No skin thinning was reported with once or twice daily application of potent TCS for 3 weeks in one RCT (94 adults). [(102)] Four RCTs in 740 older children/adults showed no significant difference between once and twice daily application of moderate/potent TCS in other cutaneous adverse events including telangiectasia, [(103, 104)], folliculitis, [(105)] and burning, itching or stinging. [(105, 106)] Two RCTs showed no significant differences in biochemical adrenal suppression (cortisol levels) between once and twice daily very potent/moderate TCS used for up to 2 weeks in 129 children. [(81, 107)]

How safe are TCS when used proactively to prevent flares ("weekend therapy")?

Two reviews included data on the safety of TCS used proactively 2 days a week ("weekend therapy") to prevent flares, both critically low quality. [(8, 53)] Key results can be found in Table 2 and additional data in Appendix 6.

For peer review only - http://bmjopen.bmj.com/site/about/guidelines.xhtml

There was no evidence of skin thinning in five RCTs comparing 16-20 weeks of weekend therapy with potent TCS versus vehicle in 993 participants. [103, 108-111] Furthermore, two RCTs (n=423) reported no significant differences in other cutaneous adverse events, including folliculitis and transient telangiectasia, with potent TCS compared to vehicle. [108, 109] Events were uncommon in both groups.

There was no evidence of biochemical adrenal suppression (cortisol levels) in two RCTs (n=129) between potent TCS and vehicle used for 16 weeks. [108, 111] In a 20-week RCT, 2/44 children had possible adrenal suppression with potent TCS compared to zero with vehicle. [109]

How safe are TCS used under occlusion?

Four reviews included data on the safety of TCS used under occlusion: one high [54], and three critically low quality. [26, 32, 38] Results can be found in Appendix 6.

There were no cases of skin thinning and one case of striae in two uncontrolled observational studies of a diluted potent TCS used under wet-wrap for 1-2 weeks in 44 young children. [112, 113] A significant difference in the rate of folliculitis (mostly mild) was observed in one RCT of TCS under wet-wrap for 4 weeks, with more folliculitis in the diluted potent TCS group (10/19 children) compared to emollient (2/20 children) (p=0.02). [114] A meta-analysis from one review [38] of two RCTs in young children showed no significant difference in the number of participants with cutaneous adverse events between mild potency TCS under wet wrap (7/38 participants) versus not under wet-wrap (0/31 participants) (p=0.08)[115, 116]; this evidence was rated low quality by the systematic review authors using GRADE. [17]

Biochemical adrenal suppression (cortisol levels) was reported in three uncontrolled observational studies of 2-14 days of diluted potent TCS under wet-wraps in 74 children. [112, 113, 117] Actual rates were not specified in the review, but increases were described as transient in two studies. [112, 117] One short-term uncontrolled observational study of diluted potent TCS under wet-wrap in eight children showed no effect on growth or bone turnover. [118]

For peer review only - http://bmjopen.bmj.com/site/about/guidelines.xhtml

Discussion

This comprehensive overview of systematic reviews which, for the first time, brings together all safety data from systematic reviews on topical corticosteroids (TCS) used in eczema from 38 systematic reviews, a topic that was identified as a priority in a James Lind Alliance priority setting partnership on eczema. Skin thinning and effects on growth concern many people with eczema and parents of children with eczema when using TCS. However, we found no evidence of skin thinning when TCS were used intermittently “as required” to treat flares or as “weekend therapy” to prevent flares, although the majority of data was from short-term studies.[(5)] Similarly, we found no evidence of growth retardation or clinically significant adrenal suppression but the only data available was from one 5 year that included 1213 children using TCS. [(62)] Other studies only reported biochemical signs of adrenal suppression. Adherence to TCS treatment is known to be poor and these findings, particularly around skin thinning, may encourage appropriate use of TCS and therefore improve treatment effectiveness and patient benefit. [(119)]

A thorough literature search was conducted and Cochrane methodology was used. Conclusions were limited by the content of the included reviews because safety was frequently reported in less detail than effectiveness, reviews reported on different adverse events and some adverse events were not described in the reviews. It is not clear whether this is because the trials did not report adverse events in sufficient detail or whether the review authors didn’t include all the available safety data, perhaps only focussing on a restricted group of adverse events. None of the included systematic reviews presented data on our prespecified subgroup analyses. Furthermore, most of the included reviews were rated low or critically low quality using AMSTAR 2. The lack of comprehensive search strategies and duplicate screening/data extraction in the included reviews may have resulted in missing studies and safety data, which could have impacted on this overview particularly where there was limited data. In addition, where the quality of evidence assessments (e.g. GRADE) was

For peer review only - http://bmjopen.bmj.com/site/about/guidelines.xhtml

reported in the reviews, most individual studies included in the reviews indicated a high or unclear risk in at least one domain.

Many RCTs did not include follow-up beyond 2-4 weeks of treatment and therefore data on long-term safety are limited. Although short-term TCS use reflects appropriate treatment duration for treating an individual flare, it does not reflect the chronic nature of eczema and the need for TCS use over the long-term. The 'PETITE study' was the notable exception and data published in the correspondence showed there was only one episode of skin thinning in 1213 children using mild/moderate TCS "as required" with 5-year follow up. [62] Trials using intermittent TCS as "weekend therapy" to prevent flares also provide reassurance for the safety of longer-term use of TCS, as these trials generally included 16-20 weeks of follow-up to assess the prevention of flares. The inclusion of systematic reviews that included observational studies as well as reviews of RCTs also increased the amount of safety data available to report in this overview.

Although this review focussed on the safety of TCS as the key issue for patients, treatment decisions are a balance of benefits and harms. For example, although the safety profile of Chinese herbal medicine was better than TCS, in practice this would be considered alongside the relative effectiveness of these treatments. Likewise, although there was no difference in the safety of once versus twice daily TCS, effectiveness of these regimens is also important to consider. A Cochrane review is underway comparing the effectiveness and safety of different ways of using TCS. [120]

In summary, we found no evidence that TCS cause harm when used intermittently "as required" to treatment eczema flares or as "weekend therapy" to prevent flares and this should support the use of TCS in the management of eczema. We found that the adverse events of greatest concern to patients and clinicians, such as skin thinning, are uncommon with short-term use of TCS. However, high-quality evidence was limited, particularly for long-term use. Rather than follow-up of perhaps just a few weeks, future RCTs should include lengthier follow-up to enable better safety assessment. However, it should be noted that longer-term prospect observational studies are better placed to

For peer review only - http://bmjopen.bmj.com/site/about/guidelines.xhtml

explore longer-term safety of TCS and should be designed with years rather than months of follow up to add useful information to the field. Perhaps equally as important as duration of follow up in trials is resolution of adverse events which is often not reported. For adverse events such as biochemical signs of adrenal suppression, it is crucial to know if the effect is transient and levels return to normal once the TCS is stopped, particularly as it is not clear how to interpret the clinical relevance of these.

Authors contributions and acknowledgements

All authors (EA, JRC, MS, MJR, SL, SML, DJG, IM, AR, AA, HCW, KST) helped conceive of and design this overview DJG and EA conducted the searches. EA and JRC carried out the eligibility screening, data extraction and quality assessments. HCW and KST acted as 3rd reviewers to resolve disagreements. EA performed the statistical analysis and JRC is the study guarantor. EA and JRC collated and interpreted the data with input from all other authors. EA and JRC completed the initial drafts of the manuscript and all authors (EA, JRC, MS, MJR, SL, SML, DJG, IM, AR, AA, HCW, KST) commented on and approved the final manuscript. The corresponding author attests that all listed authors meet authorship criteria and that no others meeting the criteria have been omitted.

We would like to thank Faye Shelton for her assistance with the screening of the search results and to Jane Harvey for her input into classifying the TCS potencies. We would also like to thank Chiau Ming Long for translating two of the included reviews published in Chinese, and to Jonathan Batchelor for confirming exclusion of two reviews published in Japanese.

Figure 1: PRISMA Flow Diagram

References

1. Odhiambo JA, Williams HC, Clayton TO, Robertson CF, Asher MI, ISAAC Phase Three Study Group. Global variations in prevalence of eczema symptoms in children from ISAAC Phase Three. *J Allergy Clin Immunol*. 2009;**124**(6):1251-8. e23.
2. Weidinger S, Novak N. Atopic dermatitis. *Lancet*. 2016;**387**(10023):1109-22.

For peer review only - http://bmjopen.bmj.com/site/about/guidelines.xhtml

3. Abuabara K, Yu A, Okhovat J, Allen I, Langan S. The prevalence of atopic dermatitis beyond childhood: A systematic review and meta-analysis of longitudinal studies. *Allergy*. 2018;**73**(3):696-704.
4. Aubert-Wastiaux H, Moret L, Le Rhun A, Fontenoy A, Nguyen J, Leux C, et al. Topical corticosteroid phobia in atopic dermatitis: a study of its nature, origins and frequency. *Br J Dermatol*. 2011;**165**(4):808-14.
5. Charman C, Morris A, Williams H. Topical corticosteroid phobia in patients with atopic eczema. *Br J Dermatol*. 2000;**142**(5):931-6.
6. Li AW, Yin ES, Antaya RJ. Topical Corticosteroid Phobia in Atopic Dermatitis: A Systematic Review. *JAMA Dermatol*. 2017;**153**(10):1036-42.
7. Green C, Colquitt J, Kirby J, Davidson P, Payne E. Clinical and cost-effectiveness of once-daily versus more frequent use of same potency topical corticosteroids for atopic eczema: a systematic review and economic evaluation. *Health Technol Assess (Winchester, England)*. 2004;**8**(47):iii,iv, 1-120.
8. Schmitt J, Von Kobyletzki L, Svensson A, Apfelbacher C. Efficacy and tolerability of proactive treatment with topical corticosteroids and calcineurin inhibitors for atopic eczema: Systematic review and meta-analysis of randomized controlled trials. *Br J Dermatol*. 2011;**164**(2):415-28.
9. Williams H. Established corticosteroid creams should be applied only once daily in patients with atopic eczema. *BMJ*. 2007;**334**(7606):1272.
10. Batchelor J, Ridd M, Clarke T, Ahmed A, Cox M, Crowe S, et al. The Eczema Priority Setting Partnership: a collaboration between patients, carers, clinicians and researchers to identify and prioritize important research questions for the treatment of eczema. *Br J Dermatol*. 2013;**168**(3):577-82.
11. Bougioukas KI LA, Tsapas A, Ntzani E, Haidich AB. Preferred Reporting Items for Overviews of systematic reviews including harms checklist: A pilot tool to be used for balanced reporting of benefits and harms. *J Clin Epidemiol*. 2018;**93**:9-24.
12. Mead E, Chalmers J, Thomas K, Santer M, Williams H, Grindlay D, et al. The safety of topical corticosteroids in atopic eczema: an overview of systematic reviews [CRD42018079409]2017 (accessed: 13/07/18). Available from: https://www.crd.york.ac.uk/prospero/display_record.php?RecordID=79409.
13. Rada G, Pérez D, Araya-Quintanilla F, Ávila C, Bravo-Soto G, Bravo-Jeria R, et al. Epistemonikos: a comprehensive database of systematic reviews for health decision-making. *BMC Med Res Methodol*. 2020;**20**(1):286.
14. Centre of Evidence Based Dermatology. Systematic reviews on eczema: University of Nottingham; 2018 [07/11/2018]. Available from: https://www.nottingham.ac.uk/research/groups/cebd/resources/eczema-systematic-reviews.aspx.
15. Covidence systematic review software. Melbourne, Australia: Veritas Health Innovation. Available at www.covidence.org; 2017.
16. Shea BJ, Reeves BC, Wells G, Thuku M, Hamel C, Moran J, et al. AMSTAR 2: a critical appraisal tool for systematic reviews that include randomised or non-randomised studies of healthcare interventions, or both. *BMJ*. 2017;**358**:j4008.
17. Schünemann H BJ, Oxman A, editor(s). Handbook for grading the quality of evidence and the strength of recommendations using the GRADE approach (updated October 2013). GRADE Working Group, 2013: The GRADE Working Group.; 2013. Available from: www.guidelinedevelopment.org/handbook.
18. Review Manager 5 (RevMan 5). Copenhagen: Nordic Cochrane Centre, The Cochrane Collaboration; 2014.
19. Resource clinical. Potency of Topical Corticosteroids (UK Classification). 2015 [Available from: http://www.resourceclinical.com/clinical-tables/topical-corticosteroids-potency-UK-classification.html.

For peer review only - http://bmjopen.bmj.com/site/about/guidelines.xhtml

20. Resource clinical. Potency of Topical Corticosteroids (USA Classification) 2015 [Available from: http://www.resourceclinical.com/clinical-tables/topical-corticosteroids-potency-USA-classification.pdf.
21. World Health Organization (WHO). WHO Model Prescribing Information: Drugs Used in Skin Diseases. Classification of topical corticosteroids. 2017 [Available from: http://apps.who.int/medicinedocs/en/d/Jh2918e/32.html.
22. NHS. What are side effects? 2018 [Available from: https://www.nhs.uk/common-health-questions/medicines/what-are-side-effects/.
23. Ashcroft DM, Chen L-C, Garside R, Stein K, Williams HC. Topical pimecrolimus for eczema Cochrane Database Syst Rev [Internet]. 2007; (4). Available from: http://onlinelibrary.wiley.com/doi/10.1002/14651858.CD005500.pub2/abstract.
24. Ashcroft D, Dimmock P, Garside R, Stein K, Williams H. Efficacy and tolerability of topical pimecrolimus and tacrolimus in the treatment of atopic dermatitis: Meta-analysis of randomised controlled trials. *BMJ* 2005;**330**(7490):516-22.
25. Barnes L, Kaya G, Rollason V. Topical Corticosteroid-Induced Skin Atrophy: A Comprehensive Review. *Drug Saf*. 2015;**38**(5):493-509.
26. Braham S, Pugashetti R, Koo J, Maibach H. Occlusive therapy in atopic dermatitis: overview. *J Dermatolog Treat*. 2010;**21**(2):62-72.
27. Broeders J, Ahmed Ali U, Fischer G. Systematic review and meta-analysis of randomized clinical trials (RCTs) comparing topical calcineurin inhibitors with topical corticosteroids for atopic dermatitis: A 15-year experience. *J Am Acad Dermatol*. 2016;**75**(2):410-9.e3.
28. Callen J, Chamlin S, Eichenfield L, Ellis C, Girardi M, Goldfarb M, et al. A systematic review of the safety of topical therapies for atopic dermatitis. *Br J Dermatol*. 2007;**156**(2):203-21.
29. Chen S, Yan J, Wang F. Two topical calcineurin inhibitors for the treatment of atopic dermatitis in pediatric patients: A meta-analysis of randomized clinical trials. *J Dermatolog Treat*. 2010;**21**(3):144-56.
30. Cury Martins J, Martins C, Aoki V, Gois AF, Ishii HA, da SEM. Topical tacrolimus for atopic dermatitis. Cochrane Database Syst Rev [Internet]. 2015; (7). Available from: http://onlinelibrary.wiley.com/doi/10.1002/14651858.CD009864.pub2/abstract.
31. de Tiedra A, Mercadal J, Lozano R. Prednicarbate versus fluocortin for inflammatory dermatoses: A cost-effectiveness study. *PharmacoEconomics*. 1997;**12**(2 Pt 1):193-208.
32. Devillers A, Oranje A. Efficacy and safety of 'wet-wrap' dressings as an intervention treatment in children with severe and/or refractory atopic dermatitis: a critical review of the literature. *Br J Dermatol*. 2006;**154**(4):579-85.
33. Dong Y, Zeng W, Li W, Ma H, Zheng W. Efficacy and safety of topical tacrolimus for childhood atopic dermatitis; a meta-analysis. [Chinese]. *J Clin Dermatol*. 2017;**46**(4):239-42.
34. Eichenfield L, Tom W, Berger T, Krol A, Paller A, Schwarzenberger K, et al. Guidelines of care for the management of atopic dermatitis: Section 2. Management and treatment of atopic dermatitis with topical therapies. *J Am Acad Dermatol*. 2014;**71**(1):116-32.
35. Feldman S. Relative efficacy and interchangeability ofvarious clobetasol propionate vehicles in the management of steroid-responsive dermatoses. *Curr Ther Res Clin Exp*. 2005;**66**(3):154-71.
36. Frangos J, Kimball A. Clobetasol propionate emollient formulation foam in the treatment of corticosteroid-responsive dermatoses. *Expert Opin Pharmacother*. 2008;**9**(11):2001-7.
37. Froeschl B, Arts D, Leopold C. Corticosteroid therapy in the treatment of pediatric patients with atopic dermatitis (Structured abstract). Health Technol Assess [Internet]. 2007; (4). Available from: http://onlinelibrary.wiley.com/o/cochrane/clhta/articles/HTA-32008100208/frame.html.
38. Gonzalez-Lopez G, Ceballos-Rodriguez R, Gonzalez-Lopez J, Feito Rodriguez M, Herranz-Pinto P. Efficacy and safety of wet wrap therapy for patients with atopic dermatitis: a systematic review and meta-analysis. *Br J Dermatol*. 2017;**177**(3):688-95.
39. Gu S, Yang A, Li C, Lu C, Xue C. Topical application of Chinese herbal medicine for atopic eczema: A systematic review with a meta-analysis. *Dermatology*. 2014;**228**(4):294-302.

For peer review only - http://bmjopen.bmj.com/site/about/guidelines.xhtml

40. Gu S, Yang AW, Xue CC, Li CG, Pang C, Zhang W, et al. Chinese herbal medicine for atopic eczema. Cochrane Database Syst Rev [Internet]. 2013; (9). Available from: http://onlinelibrary.wiley.com/doi/10.1002/14651858.CD008642.pub2/abstract.
41. Hajar T, Leshem Y, Hanifin J, Nedorost S, Lio P, Paller A, et al. A systematic review of topical corticosteroid withdrawal ("steroid addiction") in patients with atopic dermatitis and other dermatoses. *J Am Acad Dermatol*. 2015;**72**(3):541-9.e2.
42. Hoare C, Li Wan Po A, Williams H. Systematic review of treatments for atopic eczema. *Health Technol Assess*. 2000;**4**(37):1-191.
43. Iskedjian M, Piwko C, Shear N, Langley R, Einarson T. Topical calcineurin inhibitors in the treatment of atopic dermatitis: A meta-analysis of current evidence. *Am J Clin Dermatol*. 2004;**5**(4):267-79.
44. Juhász ML, Curley RA, Rasmussen A, Malakouti M, Silverberg N, Jacob SE. Systematic Review of the Topical Steroid Addiction and Topical Steroid Withdrawal Phenomenon in Children Diagnosed With Atopic Dermatitis and Treated With Topical Corticosteroids. *J Dermatol Nurses Assoc*. 2017;**9**(5):233-40.
45. Legendre L, Barnetche T, Mazereeuw-Hautier J, Meyer N, Murrell D, Paul C. Risk of lymphoma in patients with atopic dermatitis and the role of topical treatment: A systematic review and meta-analysis. *J Am Acad Dermatol*. 2015;**72**(6):992-1002.
46. Li R, Zhu H, Fan L, Ni S, Feng C, Wu Z. Efficacy and tolerability of topical tacrolimus in the treatment of atopic dermatitis: A systematic review of randomized controlled trials. [Chinese]. *J Clin Dermatol*. 2007;**36**(12):757-60.
47. Nankervis H, Thomas K, Delamere F, Barbarot S, Rogers N, Williams H. Scoping systematic review of treatments for eczema2016 2016/05/None.
48. Penaloza Hidalgo B, Knight T, Burls A. A systematic review of effectiveness and cost effectiveness of tacrolimus ointment for topical treatment of atopic dermatitis in adults and children Health Technol Assess [Internet]. 2004; (4):[81 p.].
49. Sidbury R, Tom W, Bergman J, Cooper K, Silverman R, Berger T, et al. Guidelines of care for the management of atopic dermatitis: Section 4. Prevention of disease flares and use of adjunctive therapies and approaches. *J Am Acad Dermatol*. 2014;**71**(6):1218-33.
50. Siegfried E, Jaworski J, Kaiser J, Hebert A. Systematic review of published trials: Long-term safety of topical corticosteroids and topical calcineurin inhibitors in pediatric patients with atopic dermatitis. *BMC Pediatr*. 2016;**16** (75).
51. Singh S, Mann B. Clinical utility of clocortolone pivalate for the treatment of corticosteroid-responsive skin disorders: A systematic review. *Clin Cosmet Investig Dermatol*. 2012;**5**:61-8.
52. Svensson A, Chambers C, Gånemo A, Mitchell S. A systematic review of tacrolimus ointment compared with corticosteroids in the treatment of atopic dermatitis. *Curr Med Res Opin*. 2011;**27**(7):1395-406.
53. Tang T, Bieber T, Williams H. Are the concepts of induction of remission and treatment of subclinical inflammation in atopic dermatitis clinically useful? *J Allergy Clin Immunol*. 2014;**133**(6):1615-25.e1.
54. van Zuuren EJ, Fedorowicz Z, Christensen R, Lavrijsen AP, Arents BW. Emollients and moisturisers for eczema. Cochrane Database Syst Rev [Internet]. 2017; (2). Available from: http://onlinelibrary.wiley.com/doi/10.1002/14651858.CD012119.pub2/abstract.
55. Wood Heickman L, Davallow Ghajar L, Conaway M, Rogol A. Evaluation of Hypothalamic-Pituitary-Adrenal Axis Suppression following Cutaneous Use of Topical Corticosteroids in Children: A Meta-Analysis. *Horm Res Paediatr*. 2018;**89**(6):389-96.
56. Yan J, Chen S, Wang X, Zhou W, Wang F. Meta-analysis of tacrolimus ointment for atopic dermatitis in pediatric patients. *Pediatr Dermatol*. 2008;**25**(1):117-20.
57. Ruirui L, Jing G, Dingxi B, Qian Y, Lin Z, Zhi Y, et al. A network meta-analysis of five categories of external therapy of traditional Chinese for common diseases of dermatology

For peer review only - http://bmjopen.bmj.com/site/about/guidelines.xhtml

[CRD42021230047]2021 (assessed: 27/03/2021). Available from: www.crd.york.ac.uk/prospero/display_record.php?RecordID=230047.
58. Lee K. Efficacy of Non-Steroidal Topical Therapies for Atopic Dermatitis: A Systematic Review & Meta-Analysis [CRD42020161558] 2020 (accessed: 27/03/2021). Available from: www.crd.york.ac.uk/prospero/display_record.php?RecordID=161558.
59. Lam M, Zhu J, Tadrous M, Drucker A. The association between topical calcineurin inhibitor use and risk of cancer: a systematic review and meta-analysis [CRD42020190452]2020 (accessed: 27/03/2021). Available from: www.crd.york.ac.uk/prospero/display_record.php?RecordID=190452.
60. Tan Q, Tan C, Peng W, Shi Y, Xia L. Effects of emollients in the management of atopic dermatitis in pediatric patients a systemic review and meta-analysis [CRD42015016525] 2015 (accessed: 27/03/21). Available from: http://www.crd.york.ac.uk/PROSPERO/display_record.php?ID=CRD42015016525.
61. Yu A, Hong J, Lee M, Hong B. Interventions to improve quality of life in paediatric atopic dermatitis: a systematic review [CRD42015027873] 2015 (accessed: 27/03/21). Available from: http://www.crd.york.ac.uk/PROSPERO/display_record.php?ID=CRD42015027873.
62. Sigurgeirsson B, Boznanski A, Todd G, Vertruyen A, Schuttelaar M, Zhu X, et al. Safety and efficacy of pimecrolimus in atopic dermatitis: a 5-year randomized trial. *Pediatrics* 2015;**135**(4):597-606.
63. Fishbein AB, Mueller K, Lor J, Smith P, Paller AS, Kaat A. Systematic Review and Meta-analysis Comparing Topical Corticosteroids With Vehicle/Moisturizer in Childhood Atopic Dermatitis. *J Pediatr Nurs*. 2019;**47**:36-43.
64. Breneman D, Fleischer A, Foley V ea, editors. Clobetasol propionate 0.05% is equivalent as lotion or emollient cream in atopic dermatitis. Presented at the World Congress of Dermatology, 2002; and the European Academy of Dermatology and Venereology, 2003; 2003.
65. Rosso J, Bhambri S. Daily application of fluocinonide 0.1% cream for the treatment of atopic dermatitis. *J Clin Aesthet Dermatol*. 2009;**2**(9):24-32.
66. Binder R. Clinical study of clocortolone pivalate in the treatment of eczema/atopic dermatitis. *Curr Ther Res Clin Exp*. 1977;**21**(6):796-801.
67. Griffiths C, Van Leent E, Gilbert M, Traulsen J, Cipamyflline Study Group. Randomized comparison of the type 4 phosphodiesterase inhibitor cipamfylline cream, cream vehicle and hydrocortisone 17-butyrate cream for the treatment of atopic dermatitis. *Br J Dermatol*. 2002;**147**(2):299-307.
68. Paller A, Nimmagadda S, Schachner L, Mallory S, Kahn T, Willis I, et al. Fluocinolone acetonide 0.01% in peanut oil: therapy for childhood atopic dermatitis, even in patients who are peanut sensitive. *J Am Acad Dermatol*. 2003;**48**(4):569-77.
69. Pellanda C, Weber M, Bircher A, Surber C. Low-dose triamcinolone acetonide in the phytocosmetic lichtena reduces inflammation in mild to moderate atopic dermatitis. *Dermatology*. 2005;**211**(4):338-40.
70. Dolle S, Hoser D, Rasche C, Loddenkemper C, Maurer M, Zuberbier T, et al. Long-term reduction in local inflammation by a lipid raft molecule in atopic dermatitis. *Allergy*. 2010;**65**(9):1158-65.
71. Abramovits W, Oquendo M. Hydrocortisone butyrate 0.1% cream (proprietary lipid rich cream vehicle) does not significantly suppress hypothalamic-pituitary-adrenal axis and is effective in pediatric patients 3 months and older with extensive atopic dermatitis. *Skinmed*. 2010;**8**(3):150-4.
72. Dohil MA, Alvarez ‐ Connelly E, Eichenfield LF. Fluocinolone acetonide 0.01% in peanut oil: safety and efficacy data in the treatment of childhood atopic dermatitis in infants as young as 3 months of age. *Pediatr Dermatol*. 2009;**26**(3):262-8.
73. Eichenfield L, Ellis CN, Fivenson D, Hebert AA, Dromgoole S, Piacquadio D. Evaluation of adrenal suppression of a lipid enhanced, topical emollient cream formulation of hydrocortisone butyrate 0.1% in treating children with atopic dermatitis. *Pediatr Dermatol*. 2007;**24**(1):81-4.

For peer review only - http://bmjopen.bmj.com/site/about/guidelines.xhtml

74. Eichenfield LF, Basu S, Calvarese B, Trancik RJ. Effect of desonide hydrogel 0.05% on the hypothalamic - pituitary - adrenal axis in pediatric subjects with moderate to severe atopic dermatitis. *Pediatr Dermatol*. 2007;**24**(3):289-95.
75. Friedlander S, Hebert A, Allen D. Safety of fluticasone propionate cream 0.05% for the treatment of severe and extensive atopic dermatitis in children as young as 3 months. *J Am Acad Dermatol*. 2002;**46**(3):387-93.
76. Hebert AA, Friedlander SF, Allen DB. Topical fluticasone propionate lotion does not cause HPA axis suppression. *J Pediatr*. 2006;**149**(3):378-82.
77. Hebert AA, Desonide Foam Phase III Clinical Study Group. Desonide foam 0.05%: safety in children as young as 3 months. *J Am Acad Dermatol*. 2008;**59**(2):334-40.
78. Kimball AB, Gold MH, Zib B, Davis MW, Phase CPEFF, Group ICS. Clobetasol propionate emulsion formulation foam 0.05%: review of phase II open-label and phase III randomized controlled trials in steroid-responsive dermatoses in adults and adolescents. *J Am Acad Dermatol*. 2008;**59**(3):448-54. e1.
79. Lucky A, Grote G, Williams J, Tuley M, Czernielewski J, Dolak T, et al. Effect of desonide ointment, 0.05%, on the hypothalamic-pituitary-adrenal axis of children with atopic dermatitis. *Cutis*. 1997;**59**(3):151-3.
80. Moshang T. Prednicarbate emollient cream 0.1% in pediatric patients with atopic dermatitis. *Cutis*. 2001;**68**(1):63-9.
81. Schlessinger J, Miller B, Gilbert R, Plott R, Vanos Study Group. An open-label adrenal suppression study of 0.1% fluocinonide cream in pediatric patients with atopic dermatitis. *Arch Dermatol*. 2006;**142**(12):1568-72.
82. Łabędź N, Pawliczak R. Efficacy and safety of topical calcineurin inhibitors for the treatment of atopic dermatitis: meta-analysis of randomized clinical trials. *Postepy Dermatol Alergol*. 2019;**36**(6):752-9.
83. Luger T, Lahfa M, Folster-Holst R, Gulliver W, Allen R, Molloy S, et al. Long-term safety and tolerability of pimecrolimus cream 1% and topical corticosteroids in adults with moderate to severe atopic dermatitis. *J Dermatolog Treat*. 2004;**15**(3):169-78.
84. Mandelin J, Remitz A, Virtanen H, Reitamo S. One-year treatment with 0.1% tacrolimus ointment versus a corticosteroid regimen in adults with moderate to severe atopic dermatitis: A randomized, double-blind, comparative trial. *Acta Derm Venereol*. 2010;**90**(2):170-4.
85. Reitamo S, Ortonne J, Sand C, Cambazard F, Bieber T, Folster-Holst R, et al. A multicentre, randomized, double-blind, controlled study of long-term treatment with 0.1% tacrolimus ointment in adults with moderate to severe atopic dermatitis. *Br J Dermatol*. 2005;**152**(6):1282-9.
86. Doss N, Kamoun M, Dubertret L, Cambazard F, Remitz A, Lahfa M, et al. Efficacy of tacrolimus 0.03% ointment as second-line treatment for children with moderate-to-severe atopic dermatitis: evidence from a randomized, double-blind non-inferiority trial vs. fluticasone 0.005% ointment. *Pediatr Allergy Immunol*. 2010;**21**(2 Pt 1):321-9.
87. Doss N, Reitamo S, Dubertret L, Fekete G, Kamoun M, Lahfa M, et al. Superiority of tacrolimus 0.1% ointment compared with fluticasone 0.005% in adults with moderate to severe atopic dermatitis of the face: results from a randomized, double-blind trial. *Br J Dermatol*. 2009;**161**(2):427-34.
88. Hofman T, Cranswick N, Kuna P, Boznanski A, Latos T, Gold M, et al. Tacrolimus ointment does not affect the immediate response to vaccination, the generation of immune memory, or humoral and cell-mediated immunity in children. *Arch Dis Child*. 2006;**91**(11):905-10.
89. Luger T, Van Leent E, Graeber M, Hedgecock S, Thurston M, Kandra A, et al. SDZ ASM 981: an emerging safe and effective treatment for atopic dermatitis. *Br J Dermatol*. 2001;**144**(4):788-94.
90. Reitamo S, Harper J, Bos J, Cambazard F, Bruijnzeel-Koomen C, Valk P, et al. 0.03% Tacrolimus ointment applied once or twice daily is more efficacious than 1% hydrocortisone acetate in children with moderate to severe atopic dermatitis: results of a randomized double-blind controlled trial. *Br J Dermatol*. 2004;**150**(3):554-62.

For peer review only

For peer review only - http://bmjopen.bmj.com/site/about/guidelines.xhtml

91. Reitamo S, Rustin M, Ruzicka T, Cambazard F, Kalimo K, Friedmann P, et al. Efficacy and safety of tacrolimus ointment compared with that of hydrocortisone butyrate ointment in adult patients with atopic dermatitis. *J Allergy Clin Immunol*. 2002;**109**(3):547-55.
92. Reitamo S, Van Leent E, Ho V, Harper J, Ruzicka T, Kalimo K, et al. Efficacy and safety of tacrolimus ointment compared with that of hydrocortisone acetate ointment in children with atopic dermatitis. *J Allergy Clin Immunol*. 2002;**109**(3):539-46.
93. Sikder M, Al Mamun S, Khan R, Chowdhury A, Khan H, Hoque M. Topical 0.03% tacrolimus ointment, 0.05% clobetasone butyrate cream alone and their combination in older children with atopic dermatitis - An open randomized comparative study. *J Pak Assoc Dermatol*. 2005;**15**(4):304-12.
94. Schneeweiss S, Doherty M, Zhu S, Funch D, Schlienger R, Fernandez-Vidaurre C, et al. Topical treatments with pimecrolimus, tacrolimus and medium- to high-potency corticosteroids, and risk of lymphoma. *Dermatology*. 2009;**219**(1):7-21.
95. Arellano F, Wentworth C, Arana A, Fernandez C, Paul C. Risk of lymphoma following exposure to calcineurin inhibitors and topical steroids in patients with atopic dermatitis. *J Invest Dermatol*. 2007;**127**(4):808-16.
96. Prado de Oliveira Z, Cuce L, Arnone M. Comparative evaluation of efficacy, tolerability and safety of 0.1% topical momethasone furoate and 0.05% desonide in the treatment of childhood atopic dermatitis. *An Bras Dermatol*. 2002;**77**:25-33.
97. Kuokkanen K, Sillantaka I. Alclometasone dipropionate 0.05% vs hydrocortisone 1.0%: potential to induce cutaneous atrophy in children. *Clin Ther*. 1987;**9**(2):223-31.
98. Smitt J, Winterberg D, Oosting J. Treatment of atopic dermatitis with topical corticosteroids in children. Efficacy and systemic effects of triamcinolone acetonide and alclometasone dipropionate. *Eur J Dermatol*. 1993;**3**(7):549-52.
99. Chen H. Clinical observation of Huanglian Qingdai cream for infantile eczema

J Sichuan Trad Chin Med. 2011;**29**:88–9.
100. Huang Z, Chen P, Wei W. Observation of efficacy of Chushi Zhiyang cream for atopic dermatitis. *Mod J Integr Trad Chin West Med*. 2010;**19**:2647–8.
101. Dong M. Efficacy of external use of Jingfang mixture in the treatment of children with eczema. *Med J Qilu*. 2012;**27**:75–8.
102. Hoybye S, Balk Moller S, De Cunha Bang F, Ottevanger V, Veien N. Continuous and intermittent treatment of atopic dermatitis in adults with mometasone furoate versus hydrocortisone 17-butyrate. *Curr Ther Res Clin Exp*. 1991;**50**(1):67-72.
103. Berth-Jones J, Damstra R, Golsch S, Livden J, Van Hooteghem O, Allegra F, et al. Twice weekly fluticasone propionate added to emollient maintenance treatment to reduce risk of relapse in atopic dermatitis: randomised, double blind, parallel group study. *BMJ* 2003;**326**(7403):1367-9.
104. Marchesi E, Rozzoni M, Pini P, Cainelli T. Comparative study of mometasone furoate and betamethasone dipropionate in the treatment of atopic dermatitis. *G Ital Dermatol Venereol*. 1994;**129**(1-2):IX-XII.
105. Koopmans B, Lasthein Andersen B, Mork N, Austad J, Suhonen R, Roders G. Multicentre randomized double-blind study of Locoid Lipocream fatty cream twice daily versus Locoid Lipocream once daily and Locobase once daily. *J Dermatolog Treat*. 1995;**6**(2):103-6.
106. Tharp M. A comparison of twice-daily and once-daily administration of fluticasone propionate cream, 0.05%, in the treatment of eczema. *Cutis*. 1996;**57**(Suppl 2):19-26.
107. Richelli C, Piacentini G, Sette L, Bonizzato M, Andreoli A, Boner A. Clinical efficacy and tolerability of clobetasone 17-butyrate 0.5% lotion in children with atopic dermatitis. *Curr Ther Res Clin Exp*. 1990;**47**(3):413-7.
108. Glazenburg E, Wolkerstorfer A, Gerretsen A, Mulder P, Oranje A. Efficacy and safety of fluticasone propionate 0.005% ointment in the long-term maintenance treatment of children with atopic dermatitis: differences between boys and girls? *Pediatr Allergy Immunol*. 2009;**20**(1):59-66.

For peer review only - http://bmjopen.bmj.com/site/about/guidelines.xhtml

109. Hanifin J, Gupta A, Rajagopalan R. Intermittent dosing of fluticasone propionate cream for reducing the risk of relapse in atopic dermatitis patients. *Br J Dermatol*. 2002;**147**(3):528-37.
110. Peserico A, Stadtler G, Sebastian M, Fernandez R, Vick K, Bieber T. Reduction of relapses of atopic dermatitis with methylprednisolone aceponate cream twice weekly in addition to maintenance treatment with emollient: a multicentre, randomized, double-blind, controlled study. *Br J Dermatol*. 2008;**158**(4):801-7.
111. Van Der Meer J, Glazenburg E, Mulder P, Eggink H, Coenraads P. The management of moderate to severe atopic dermatitis in adults with topical fluticasone propionate. The Netherlands Adult Atopic DermatitisStudy Group. *Br J Dermatol*. 1999;**140**(6):1114-21.
112. Devillers A, de Waard-van der Spek F, Mulder P, Oranje A. Treatment of refractory atopic dermatitis using 'wet-wrap'dressings and diluted corticosteroids: results of standardized treatment in both children and adults. *Dermatology*. 2002;**204**(1):50-5.
113. Wolkerstorfer A, Visser R, De Waard van der Spek F, Mulder P, Oranje A. Efficacy and safety of wet - wrap dressings in children with severe atopic dermatitis: influence of corticosteroid dilution. *Br J Dermatol*. 2000;**143**(5):999-1004.
114. Janmohamed S, Oranje A, Devillers A, Rizopoulos D, van Praag M, Van Gysel D, et al. The proactive wet-wrap method with diluted corticosteroids versus emollients in children with atopic dermatitis: a prospective, randomized, double-blind, placebo-controlled trial. *J Am Acad Dermatol*. 2014;**70**(6):1076-82.
115. Beattie P, Lewis-Jones M. A pilot study on the use of wet wraps in infants with moderate atopic eczema. *Clin Exp Dermatol*. 2004;**29**(4):348-53.
116. Hindley D, Galloway G, Murray J, Gardener L. A randomised study of "wet wraps" versus conventional treatment for atopic eczema. *Arch Dis Child*. 2006;**91**(2):164-8.
117. Goodyear H, Spowart K, Harper J. 'Wet-wrap'dressings for the treatment of atopic eczema in children. *Br J Dermatol*. 1991;**125**(6):604.
118. McGowan R, Tucker P, Joseph D, Wallace A, Hughes I, Burrows N, et al. Short-term growth and bone turnover in children undergoing occlusive steroid ('wet-wrap') dressings for treatment of atopic eczema. *J Dermatolog Treat*. 2003;**14**(3):149-52.
119. NICE. Atopic eczema in under 12s: diagnosis and management. Clinical guideline [CG57]. 2007 [Available from: https://www.nice.org.uk/guidance/cg57.
120. Chalmers JR, Axon E, Harvey J, Santer M, Ridd MJ, Lawton S, et al. Different strategies for using topical corticosteroids in people with eczema. *Cochrane Database Syst Rev*. 2019(6).

For peer review only - http://bmjopen.bmj.com/site/about/guidelines.xhtml

Tables

Table 1: Characteristics of included systematic reviews

First author, publication year	Type of review	Review contained safety data from RCTs for comparisons of interest?	Review contained safety data from observational studies?	AMSTAR 2 rating
Ashcroft 2005 [24]	Non-Cochrane	Yes (TCS versus TCI)	No	Critically low [1] 3 6 7
Ashcroft 2007 [23]	Cochrane	Yes (TCS versus TCI)	Yes (TCS versus TCI)	Moderate [8]
Barnes 2015 [25]	Non-Cochrane	Yes (TCS versus vehicle, TCS versus TCI, TCS versus another TCS)	Yes (single arm TCS studies)	Critically low [1] 2 3 4 6
Braham 2010 [26]	Non-Cochrane	Yes (occluded TCS versus non-occluded TCS)	Yes (occluded TCS)	Critically low [1] 2 3 4 6
Broeders 2016 [27]	Non-Cochrane	Yes (TCS versus TCI)	No	Critically low [1] 3 5 6
Callen 2007 [28]	Non-Cochrane	Yes (TCS versus vehicle, TCS versus another TCS)	Yes (single arm studies or comparing TCS potencies)	Critically low [1] 2 3 4 6
Chen 2010 [29]	Non-Cochrane	Yes (TCS versus TCI)	No	Critically low [1] 3 6
Cury Martins 2015 [30]	Cochrane	Yes (TCS versus TCI)	Yes (TCS versus TCI)	Moderate [8]
De Tiedra 1997 [31]	Non-Cochrane	Yes (TCS versus another TCS)	Yes (usually only reported data from one arm of RCTs)	Critically low [1] 2 3 4 6
Devillers 2006 [32]	Non-Cochrane	Yes (occluded TCS versus non-occluded TCS)	Yes (occluded TCS)	Critically low [1] 2 3 4 6
Dong 2017 [33]	Non-Cochrane	Yes (TCS versus TCI)	No	Critically low [1] 2 3 4 6
Eichenfield 2014 [34]	Non-Cochrane	No	Yes (different TCS potencies)	Critically low [1] 2 3 4 6
Feldman 2005 [35]	Non-Cochrane	Yes (TCS versus vehicle)	No	Critically low [1] 2 3 4 6
Fishbein 2019 [63]	Non-Cochrane	Yes (TCS versus vehicle/moisturiser)	No	Critically low [3] 4 5 6 7
Frangos 2008 [36]	Non-Cochrane	Yes (TCS versus vehicle)	Yes (single arm studies)	Critically low [1] 2 3 4 6
Froeschl 2007 [37]	GMS HTA report	Yes (TCS versus vehicle, TCS versus TCI, TCS versus another TCS)	No	Critically low [1] 2 4 6
Gonzalez-Lopez 2017 [38]	Non-Cochrane	Yes (occluded TCS versus non-occluded TCS)	No	Critically low [1] 3
Green 2004 [7]	HTA report	Yes (once daily versus twice daily TCS use)	No	Low [2]
Gu 2013 [40]	Cochrane	Yes (TCS versus topical CHM)	No	High
Gu 2014 [39]	Non-Cochrane	Yes (TCS versus topical CHM)	No	Critically low [1] 2 3 7
Hajar 2015 [41]	Non-Cochrane	No	Yes (case series or case reports)	Critically low [2] 3 6
Hoare 2000 [42]	NIHR HTA report	Yes (TCS versus vehicle, TCS versus another TCS)	No	Low [1]
Iskedjian 2004 [43]	Non-Cochrane	Yes (TCS versus vehicle, TCS versus TCI)	No	Critically low [1] 3 6

For peer review only - http://bmjopen.bmj.com/site/about/guidelines.xhtml

Juhasz 2017 [44]	Non-Cochrane	No	Yes (social media analysis)	Critically low [1 2 3 4 6]
Labedz 2019 [82]	Non-Cochrane	Yes (TCS versus TCI)	No	Critically low[1 3 6 7]
Legendre 2015 [45]	Non-Cochrane	No	Yes (TCS versus TCI)	Critically low [1 2 3 6]
Li 2007 [46]	Non-Cochrane	Yes (TCS versus TCI)	No	Critically low [1 3 6]
Nankervis 2016 [47]	NIHR HTA report	Yes (TCS versus vehicle, TCS versus emollients, TCS versus TCI, TCS versus another TCS, once a day versus twice a day use, proactive TCS to prevent flares (“weekend therapy”) versus vehicle, occluded TCS versus non-occluded TCS)	No	Low [3]
Penaloza Hidalgo 2004 [48]	West Midlands HTA report	Yes (TCS versus TCI)	No	Low [6]
Schmitt 2011 [8]	Non-Cochrane	Yes (proactive TCS to prevent flares (“weekend therapy”) versus vehicle)	No	Critically low [3 6]
Sidbury 2014 [49]	Non-Cochrane	Yes (proactive TCS to prevent flares (“weekend therapy”) versus vehicle)	No	Critically low [1 2 3 4 6]
Siegfried 2016 [50]	Non-Cochrane	Yes (TCS versus vehicle, TCS versus TCI, TCS versus another TCS)	No	Critically low [1 2 3 4 6]
Singh 2012 [51]	Non-Cochrane	Yes (TCS versus vehicle, TCS versus TCI, TCS versus another TCS)	Yes (single arm study)	Critically low [1 2 6]
Svensson 2011 [52]	Non-Cochrane	Yes (TCS versus TCI)	No	Critically low [1 3 6 7]
Tang 2014 [53]	Non-Cochrane	Yes (proactive TCS to prevent flares (“weekend therapy”) versus vehicle)	No	Critically low [1 3 4 6]
van Zuuren 2017 [54]	Cochrane	Yes (TCS versus emollient)	No	High
Wood Heickman 2018 [55]	Non-Cochrane	No	Yes (single arm cohort studies)	Critically low [1 2 3 4 6 7]
Yan 2008 [56]	Non-Cochrane	Yes (TCS versus TCI)	No	Critically low [1 3 6 7]

Footnotes: Additional data on TCS including potency can be found in the Appendix 6. Key: TCS=Topical corticosteroid; TCI=Topical calcineurin inhibitors; CHM=Chinese herbal medicine; HTA=Health Technology Assessment; GMS=German Medical Science; NIHR=National Institute for Health Research.

AMSTAR 2 ratings – reasons for downgrading the quality of the review: [1]No protocol; [2]Search strategy not comprehensive; [3]No list of excluded studies with reasons; [4]Risk of bias not assessed; [5]inappropriate meta-analysis methods; [6]Risk of bias assessments not included in the interpretation of the results; [7]publication bias not explored in the meta-analysis.

For peer review only - http://bmjopen.bmj.com/site/about/guidelines.xhtml

Table 2: summary of main findings for key safety outcomes

	Cutaneous adverse events	Systemic adverse events
**How safe are TCS compared to emollient or vehicle, or no comparison?**<br><br>13 reviews:<br><br>1 moderate quality<br>2 low quality<br>10 critically low quality	• **Skin thinning:** No significant differences in 2 RCTs of 2-4 weeks compared to emollient/vehicle: (i) 0/196 children with very potent TCS and 0/33 vehicle, (ii) 6/109 very potent TCS vs 2/50 vehicle, p=0.69. Very low rates.<br><br>• **Other cutaneous adverse events:** No significant differences in 5 RCTs (2-4 weeks) between TCS (various potencies) and emollient/vehicle (n=172, plus one study, n not specified). Low event rates.	• **Biochemical evidence of adrenal suppression:** Meta-analysis (11 *observational* studies, max 4 weeks) - 20/522 children with any potency TCS (3.8%, 95% CI 2.4% to 5.8%), 3/148 children (2%) with mild potency TCS. Effects were transient.<br><br>• **Clinical symptoms or signs of adrenal suppression:** none observed in same as above observational studies.
**How safe are TCS compared to topical calcineurin inhibitors (TCI)?**<br><br>8 reviews:<br><br>1 moderate quality<br>1 low quality<br>6 critically low quality	• **Skin thinning:** Higher with TCS than TCI (meta-analysis of 4 RCTs: RR 4.86, 95% 1.06 to 22.28, n=4128) but very low rate (8/2068, 7 of which were using potent TCS).<br><br>• **Other cutaneous adverse events:** No difference in **skin infections** between TCS and TCI (8 RCTs). **Skin burning** and **pruritus** lower with TCS than TCI: meta-analysis of 10 RCTs: **burning** - RR 0.31, 95% CI 0.23 to 0.40 (n=4211), **pruritus** - RR 0.68, 95% CI 0.56 to 0.82(n=4211).	• **Growth rate:** no differences in growth rates tween TCS and TCI (1 RCT of 2418 children with 5 years follow up).<br><br>• **Lymphoma:** no cases reported in 1 same large RCT as above. One cohort study (n=1,438,333, approx. 4years follow up) - very small *non-significant* increase with TCI *and* TCS compared to general population. One case-control study - no increased risk with TCS or TCIs (294 cases/293,000 controls).

For peer review only - http://bmjopen.bmj.com/site/about/guidelines.xhtml

**How safe are once daily TCS compared with twice daily application?**<br><br>2 reviews:<br><br>2 low quality	• **Skin thinning:** no cases using once daily vs twice daily potent TCS for 3-weeks (1 RCT, 94 adults).<br><br>• **Other cutaneous adverse events:** no significant difference between groups in **telangiectasia**, **folliculitis**, or **burning/itching/stinging** (4 RCTs, 4-16 weeks follow up740 older children/adults).	• **Biochemical evidence of adrenal suppression:** no significant differences between once and twice daily moderate/potent TCS up to 2 weeks in children (2 RCTs, n=129).
**How safe are TCS used proactively to prevent flares ("weekend therapy")?**<br><br>3 reviews:<br><br>3 critically low quality	• **Skin thinning:** no cases with 16-20 weeks of 2 days/week of potent TCS versus vehicle (5 RCTs, n=993).<br><br>• **Other cutaneous adverse events:** no significant differences between groups, including **folliculitis** and transient **telangiectasia**, with potent TCS (16-20 weeks) compared to either vehicle or another TCS (2 RCTs, n=423). Events were uncommon in both groups.	• **Biochemical evidence of adrenal suppression:** no cases with 16 weeks of 2 days/week of potent TCS (2 RCTs, n=129). Possible adrenal suppression in 2/44 children with potent TCS compared to zero using vehicle (1 RCT, 20 weeks).
**How safe are TCS used under occlusion?**<br><br>4 reviews:<br><br>1 high quality<br>3 critically low quality	• **Skin thinning:** no cases in two *observational* studies (potent TCS + wet wrap, 1-2 weeks, n=44).<br><br>• **Other cutaneous adverse events:** One case of **striae** in two *observational* studies, n-44. More **folliculitis** with diluted potent TCS (10/19 children) compared to emollient (2/20), both under wet	• **Biochemical evidence of adrenal suppression:** reported in 3 *observational* studies (2-14 days of diluted potent TCS under wet-wraps in 74 children) but rates not specified in review. Described as transient in 2 studies.

For peer review only - http://bmjopen.bmj.com/site/about/guidelines.xhtml

	wrap (1 RCT). A meta-analysis (2 RCTs, n=69) of wet wrap vs no wet wrap (mild potency) - no significant difference in **cutaneous adverse events**.	• **Growth or bone turnover:** no effect seen in 1 small short-term *observational* study (potent TCS wet-wrap in 8 children, (median follow-up 12 weeks).

For peer review only

For peer review only - http://bmjopen.bmj.com/site/about/guidelines.xhtml

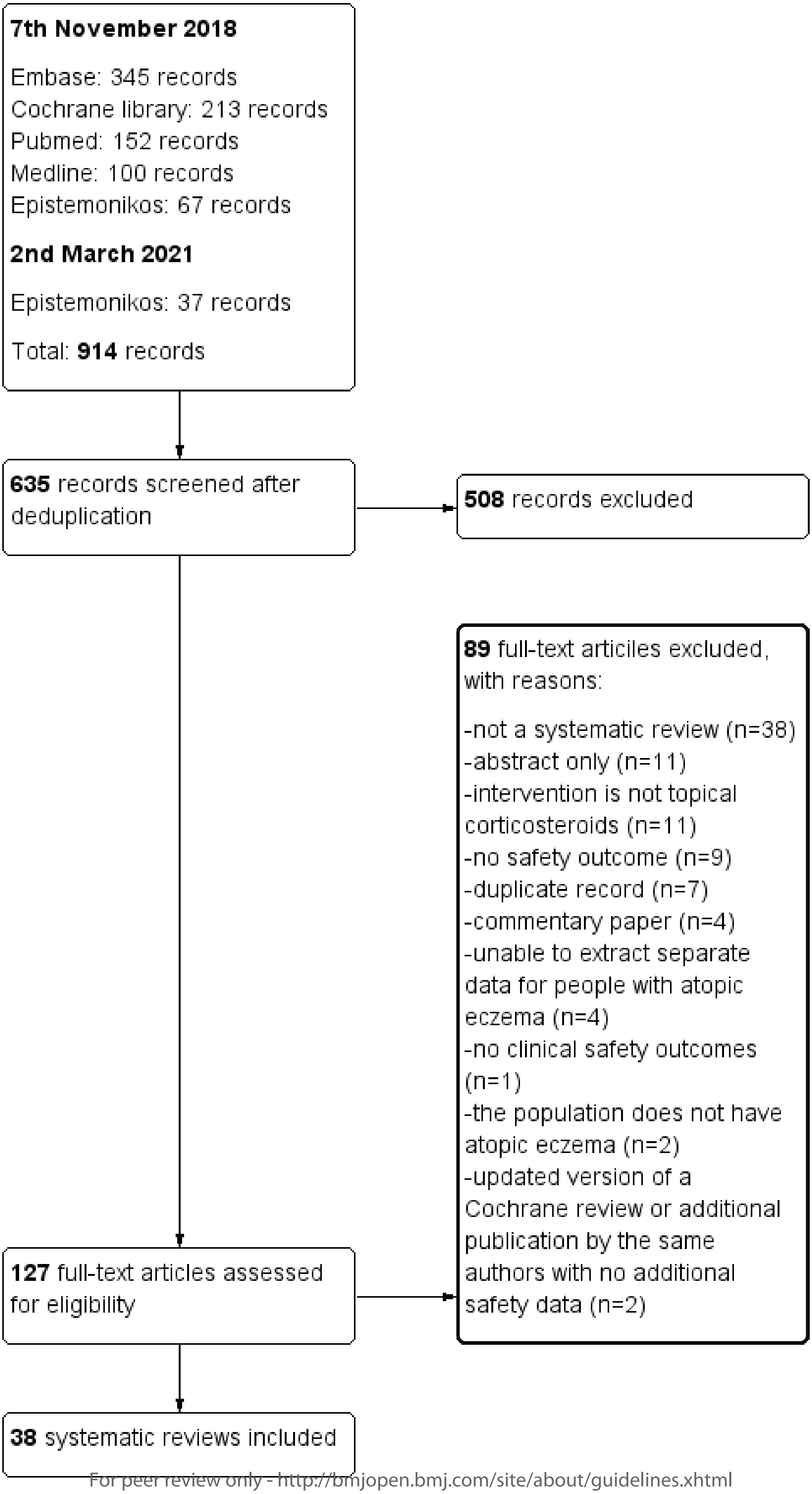
7th November 2018
Embase: 345 records
Cochrane library: 213 records
Pubmed: 152 records
Medline: 100 records
Epistemonikos: 67 records
2nd March 2021
Epistemonikos: 37 records
Total: **914** records
635 records screened after deduplication
508 records excluded
89 full-text articiles excluded, with reasons:
-not a systematic review (n=38)
-abstract only (n=11)
-intervention is not topical corticosteroids (n=11)
-no safety outcome (n=9)
-duplicate record (n=7)
-commentary paper (n=4)
-unable to extract separate data for people with atopic eczema (n=4)
-no clinical safety outcomes (n=1)
-the population does not have atopic eczema (n=2)
-updated version of a Cochrane review or additional publication by the same authors with no additional safety data (n=2)
127 full-text articles assessed for eligibility
38 systematic reviews included

Appendix 1: Search strategies

Search facets for searches:
Topical steroids
Eczema
Systematic reviews

PubMed search

Uses PubMed Clinical Queries systematic review filter (command systematic[sb]):
https://www.nlm.nih.gov/bsd/pubmed_subsets/sysreviews_strategy.html
(steroid* OR corticosteroid* OR glucocorticosteroid* OR glucocorticoid* OR glucocorticoids[MeSH Terms] OR alclometasone OR alclomethasone OR amcinonide OR beclometasone OR beclomethasone OR beclomethasone[MeSH Terms] OR betametasone OR betamethasone OR betamethasone[MeSH Terms] OR clobetasol OR clobetasol[MeSH Terms] OwR clobetasone OR desonide OR desonide[MeSH Terms] OR desoximetasone OR desoximetasone[MeSH Terms] OR diflorasone OR diflucortolone OR diflucortolone[MeSH Terms] OR fludroxycortide OR flumetasone OR flumethasone OR flumethasone[MeSH Terms] OR fluocinolone OR fluocinolone acetonide[MeSH Terms] OR fluocinonide OR fluocinonide[MeSH Terms] OR fluocortolone OR fluocortolone[MeSH Terms] OR flurandrenolide OR flurandrenolone OR flurandrenolone[MeSH Terms] OR fluticasone OR halcinonide OR halcinonide[MeSH Terms] OR halobetasol OR halometasone OR hydrocortisone OR hydrocortisone[MeSH Terms] OR methylprednisolone OR methylprednisolone[MeSH Terms] OR mometasone OR triamcinolone OR triamcinolone[MeSH Terms]) AND ("dermatitis, atopic"[MeSH Terms] OR "eczema"[MeSH Terms] OR "neurodermatitis"[MeSH Terms] OR eczema OR "atopic dermatitis" OR neurodermatitis) AND (systematic[sb] OR "systematic review")

Ovid MEDLINE search

Uses SIGN MEDLINE systematic review filter:
http://www.sign.ac.uk/search-filters.html
Ovid MEDLINE(R) Epub Ahead of Print, In-Process & Other Non-Indexed Citations, Ovid MEDLINE(R) Daily, Ovid MEDLINE and Versions(R)
MEDLINE eczema steroid systematic reviews
1. Meta-Analysis as Topic/
2. meta analy$.tw.
3. metaanaly$.tw.
4. Meta-Analysis/
5. (systematic adj (review$1 or overview$1)).tw.
6. exp Review Literature as Topic/
7. or/1-6
8. cochrane.ab.
9. embase.ab.
10. (psychlit or psyclit).ab.
11. (psychinfo or psycinfo).ab.
12. (cinahl or cinhal).ab.
13. science citation index.ab.
14. bids.ab.
15. cancerlit.ab.
16. or/8-15
17. reference list$.ab.
18. bibliograph$.ab.

For peer review only - http://bmjopen.bmj.com/site/about/guidelines.xhtml

19. hand-search$.ab.
20. relevant journals.ab.
21. manual search$.ab.
22. or/17-21
23. selection criteria.ab.
24. data extraction.ab.
25. 23 or 24
26. Review/
27. 25 and 26
28. Comment/
29. Letter/
30. Editorial/
31. animal/
32. human/
33. 31 not (31 and 32)
34. or/28-30,33
35. 7 or 16 or 22 or 27
36. 35 not 34
37. steroid$.mp.
38. corticosteroid$.mp.
39. glucocorticosteroid$.mp.
40. glucocorticoid$.mp.
41. exp Glucocorticoids/
42. alclometasone.mp.
43. alclomethasone.mp.
44. amcinonide.mp.
45. beclometasone.mp.
46. beclomethasone.mp.
47. exp Beclomethasone/
48. betametasone.mp.
49. betamethasone.mp.
50. exp Betamethasone/
51. clobetasol.mp.
52. exp Clobetasol/
53. clobetasone.mp.
54. desonide.mp.
55. exp Desonide/
56. desoximetasone.mp.
57. exp Desoximetasone/
58. diflorasone.mp.
59. diflucortolone.mp.
60. exp Diflucortolone/
61. fludroxycortide.mp.
62. flumetasone.mp.
63. flumethasone.mp.
64. exp Flumethasone/
65. fluocinolone.mp.
66. exp Fluocinolone Acetonide/
67. fluocinonide.mp.

For peer review only

68. exp Fluocinonide/
69. fluocortolone.mp.
70. exp Fluocortolone/
71. flurandrenolide.mp.
72. flurandrenolone.mp.
73. exp Flurandrenolone/
74. fluticasone.mp.
75. halcinonide.mp.
76. exp Halcinonide/
77. halobetasol.mp.
78. halometasone.mp.
79. hydrocortisone.mp.
80. exp Hydrocortisone/
81. methylprednisolone.mp.
82. exp methylprednisolone/
83. mometasone.mp.
84. triamcinolone.mp.
85. exp Triamcinolone/
86. or/37-85
87. exp dermatitis, atopic/
88. exp eczema/
89. exp neurodermatitis/
90. eczema.mp.
91. atopic dermatitis.mp.
92. neurodermatitis.mp.
93. or/87-92
94. 36 and 86 and 93

Ovid Embase search

Uses SIGN Embase systematic review filter:
http://www.sign.ac.uk/search-filters.html
Embase 1974 to 2017 October 23
Embase eczema steroid systematic reviews

1. exp Meta Analysis/
2. ((meta adj analy$) or metaanalys$).tw.
3. (systematic adj (review$1 or overview$1)).tw.
4. or/1-3
5. cancerlit.ab.
6. cochrane.ab.
7. embase.ab.
8. (psychlit or psyclit).ab.
9. (psychinfo or psycinfo).ab.
10. (cinahl or cinhal).ab.
11. science citation index.ab.
12. bids.ab.
13. or/5-12
14. reference lists.ab.
15. bibliograph$.ab.
16. hand-search$.ab.

For peer review only - http://bmjopen.bmj.com/site/about/guidelines.xhtml

For peer review only

17. manual search$.ab.
18. relevant journals.ab.
19. or/14-18
20. data extraction.ab.
21. selection criteria.ab.
22. 20 or 21
23. review.pt.
24. 22 and 23
25. letter.pt.
26. editorial.pt.
27. animal/
28. human/
29. 27 not (27 and 28)
30. or/25-26,29
31. 4 or 13 or 19 or 24
32. 31 not 30
33. steroid$.mp.
34. corticosteroid$.mp.
35. exp corticosteroid/
36. glucocorticosteroid$.mp.
37. glucocorticoid$.mp.
38. exp glucocorticoid/
39. alclometasone.mp.
40. alclomethasone.mp.
41. amcinonide.mp.
42. beclometasone.mp.
43. beclomethasone.mp.
44. betametasone.mp.
45. betamethasone.mp.
46. clobetasol.mp.
47. clobetasone.mp.
48. desonide.mp.
49. desoximetasone.mp.
50. diflorasone.mp.
51. diflucortolone.mp.
52. fludroxycortide.mp.
53. flumetasone.mp.
54. flumethasone.mp.
55. fluocinolone.mp.
56. fluocinonide.mp.
57. fluocortolone.mp.
58. flurandrenolide.mp.
59. flurandrenolone.mp.
60. fluticasone.mp.
61. halcinonide.mp.
62. halobetasol.mp.
63. halometasone.mp.
64. hydrocortisone.mp.
65. methylprednisolone.mp.

For peer review only - http://bmjopen.bmj.com/site/about/guidelines.xhtml

66. mometasone.mp.
67. triamcinolone.mp.
68. or/33-67
69. exp atopic dermatitis/
70. exp eczema/
71. exp neurodermatitis/
72. eczema.mp.
73. atopic dermatitis.mp.
74. neurodermatitis.mp.
75. or/69-74
76. 32 and 68 and 75

Epistemonikos

(steroid* OR corticosteroid* OR glucocorticosteroid* OR glucocorticoid* OR alclometasone OR alclomethasone OR amcinonide OR beclometasone OR beclomethasone OR betametasone OR betamethasone OR clobetasol OR clobetasone OR desonide OR desoximetasone OR diflorasone OR diflucortolone OR fludroxycortide OR flumetasone OR flumethasone OR fluocinolone OR fluocinonide OR fluocortolone OR flurandrenolide OR flurandrenolone OR fluticasone OR halcinonide OR halobetasol OR halometasone OR hydrocortisone OR methylprednisolone OR mometasone OR triamcinolone) AND (eczema OR "atopic dermatitis" OR neurodermatitis)

Enter search into advanced search and choose "**systematic review**" from drop-down box for "Publication type":

https://www.epistemonikos.org/advanced_search?q=(steroid*%20OR%20corticosteroid*%20OR%20glucocorticosteroid*%20OR%20glucocorticoid*%20OR%20alclometasone%20OR%20alclomethasone%20OR%20amcinonide%20OR%20beclometasone%20OR%20beclomethasone%20OR%20betametasone%20OR%20betamethasone%20OR%20clobetasol%20OR%20clobetasone%20OR%20desonide%20OR%20desoximetasone%20OR%20diflorasone%20OR%20diflucortolone%20OR%20fludroxycortide%20OR%20flumetasone%20OR%20flumethasone%20OR%20fluocinolone%20OR%20fluocinonide%20OR%20fluocortolone%20OR%20flurandrenolide%20OR%20flurandrenolone%20OR%20fluticasone%20OR%20halcinonide%20OR%20halobetasol%20OR%20halometasone%20OR%20hydrocortisone%20OR%20methylprednisolone%20OR%20mometasone%20OR%20triamcinolone)%20AND%20(eczema%20OR%20%22atopic%20dermatitis%22%20OR%20neurodermatitis)&protocol=no&classification=systematic-review

Cochrane Library

(steroid* OR corticosteroid* OR glucocorticosteroid* OR glucocorticoid* OR [mh "glucocorticoids"] OR alclometasone OR alclomethasone OR amcinonide OR beclometasone OR beclomethasone OR betametasone OR betamethasone OR clobetasol OR clobetasone OR desonide OR desoximetasone OR diflorasone OR diflucortolone OR fludroxycortide OR flumetasone OR flumethasone OR fluocinolone OR fluocinonide OR fluocortolone OR flurandrenolide OR flurandrenolone OR fluticasone OR halcinonide OR halobetasol OR halometasone OR hydrocortisone OR methylprednisolone OR mometasone OR triamcinolone) AND ([mh "eczema"] OR [mh "dermatitis, atopic"] OR [mh "neurodermatitis"] OR eczema OR "atopic dermatitis" OR neurodermatitis)

"Search all text" option chosen.

Cochrane Reviews, **Other Reviews** (i.e. DARE), and **Technology Assessments** (i.e. HTA) chosen.

For peer review only - http://bmjopen.bmj.com/site/about/guidelines.xhtml

Appendix 2 - list of excluded studies with reasons

Excluded study	Reason for exclusion
Abramovits 2005 [1]	Not a systematic review
Abramovits 2006 [2]	Not a systematic review
Anonymous 1995 [3]	Not a systematic review
Anonymous 1999 [4]	Not a systematic review
Anonymous 2004 [5]	Not a systematic review
Anonymous 2005 [6]	Abstract
Anonymous 2007 [7]	Not a systematic review
Anonymous 2015 [8]	Abstract
Anonymous 2015 [9]	Abstract
Aslam 2014 [10]	Not a systematic review
Barfield 2017 [11]	Wrong intervention (not topical corticosteroids)
Batchelor 2010 [12]	Not a systematic review
Bath-Hextall 2010 [13]	Updated version of a Cochrane review (non-Cochrane) but no additional safety data
Bigby 2001 [14]	Commentary paper
Bonchak 2017 [15]	Wrong intervention (not topical corticosteroids)
Birnie 2008 [16]	Wrong intervention (not topical corticosteroids)
Boucher 2001 [17]	Not a systematic review
Broersen 2015 [18]	Unable to extract separate data for atopic eczema patients
Cameron 2000 [19]	Commentary paper
Carbone 2010 [20]	Not a systematic review
Chavigny 2005 [21]	Not a systematic review
Chi 2009 [22]	Unable to extract separate data for atopic eczema patients
Chi 2015 [23]	Unable to extract separate data for atopic eczema patients
Chia 2015 [24]	Not a systematic review
Chu 1995 [25]	Not a systematic review
Conroy 2004 [26]	Not a systematic review
Das 2017 [27]	Not a systematic review
El-Batawy 2009 [28]	No safety outcome
Fleischer Jr 2010 [29]	Wrong intervention (not topical corticosteroids)
Frohna 2005 [30]	Commentary paper
Froschl 2007 [31]	Duplicate record of an included systematic review
Furue 2006 [32]	Not a systematic review
Furue 2006 [33]	Not a systematic review
Garside 2005 [34]	No safety outcome
Ghajar 2019 [35]	'Subgroup analysis' of an included review (Wood Heickman 2018) – no additional safety data
Goustas 2003 [36]	Not a systematic review
Green 2005 [37]	Duplicate record of an included systematic review
Green 2004 [38]	Duplicate record of an included systematic review
Halling-Overgaard 2017 [39]	Skin atrophy is not assessed clinically in this review
Health Technology Assessment Database 2004 [40]	Not a systematic review
Health Technology Assessment Database 2004 [41]	Abstract – unable to find the full publication
Health Technology Assessment Database 2001 [42]	Abstract – unable to find the full publication
Health Technology Assessment Database 2004 [43]	Abstract – unable to find the full publication
Hannuksela 2000 [44]	Wrong intervention (not topical corticosteroids)
Hebert 2006 [45]	Wrong intervention (not topical corticosteroids)

For peer review only

Hoare 2000 [46]	Duplicate record of an included systematic review
Hon 2011 [47]	Wrong intervention (not topical corticosteroids)
Hulshof 2017 [48]	Wrong intervention (not topical corticosteroids)
Hussain 2016 [49]	Not a systematic review
Kaufman 2016 [50]	Abstract
Legendre 2015 [51]	Abstract
Li 2017 [52]	Abstract
Li 2017 [53]	No safety outcome
Meffert 1999 [54]	Not a systematic review
Mooney 2015 [55]	Not a systematic review
Murashkin 2016 [56]	Not a systematic review
Nankervis 2013 [57]	Abstract
Nankervis 2016 [58]	Duplicate record of an included systematic review
Nankervis 2017 [59]	Duplicate record of an included systematic review
Nowak 2017 [60]	No safety outcome
Orlow 2007 [61]	Not a systematic review
Pan 2013 [62]	No safety outcome
Park-Wyllie 2000 [63]	Unable to extract separate data for atopic eczema patients
Payne 2019 [64]	Wrong intervention (not topical corticosteroids)
Phipatanakul 2006 [65]	Commentary paper
Radovic 2017 [66]	Wrong intervention (not topical corticosteroids)
Ricci 2007 [67]	Not a systematic review
Ruzicka 1999 [68]	Wrong intervention (not topical corticosteroids)
Sanchez 2014 [69]	Not a systematic review
Schiffner 2003 [70]	No safety outcome
Schmitt 2011 [71]	Not a systematic review
Schmitt 2011 [72]	Duplicate record of an included systematic review
Sher 2012 [73]	Abstract
Sher 2012 [74]	No safety outcome
Siegfried 2013 [75]	Not a systematic review
Siegfried 2018 [76]	Not a systematic review
Silverberg 2014 [77]	Not a systematic review
Simpson 2010 [78]	Not a systematic review
Spada 2018 [79]	Not a systematic review
Torii 2003 [80]	No safety outcome
Torley 2013 [81]	Not a systematic review
Uppal 2020 [82]	Wrong intervention (not topical corticosteroids)
Van Zuuren 2017 [83]	No safety outcome (abridged Cochrane review)
Wat 2014 [84]	Wrong patient population (not atopic eczema)
Wellington 2004 [85]	Not a systematic review
Williams 2007 [86]	Not a systematic review
Williams 2008 [87]	Not a systematic review
Williams 2010 [88]	Not a systematic review
Wollenberg 2018 [89]	Not a systematic review

For peer review only

1. Abramovits W, Boguniewicz M, Paller A, Whitaker-Worth D, Prendergast M, Tokar M, et al. The economics of topical immunomodulators for the treatment of atopic dermatitis. *Pharmacoeconomics*. 2005;**23**(6):543-66.
2. Abramovits W, Hung P, Tong K. Efficacy and economics of topical calcineurin inhibitors for the treatment of atopic dermatitis. *Am J Clin Dermatol*. 2006;**7**(4):213-22.
3. Anonymous. Once-a-day topical corticosteroids. *Drug Ther Bull*. 1995;**33**(3):21-2.
4. Anonymous. Using topical corticosteroids in general practice. *MeReC Bulletin*. 1999;**10**(6):1-5.
5. Anonymous. Pimecrolimus: Me-too: Too many risks, not beneficial enough in atopic dermatitis. *Prescrire International*. 2004;**13**(74):209+12.
6. Anonymous. Once-daily topical steroid dosing effective for atopic eczema. *J Fam Pract*. 2005;**54**(6):499-500.
7. Anonymous. Atopic dermatitis in the infant and child. [French, English]. *Nouvelles Dermatologiques*. 2007;**26**(SUPPL. 1):3-9.
8. Anonymous. Abstracts of the Australasian College of Dermatologists 48th Annual Scientific Meeting. *Australasian Journal of Dermatology Conference: 48th Annual Scientific Meeting of the Australasian College of Dermatologists Adelaide, SA Australia Conference Publication:*. 2015;**56**(no pagination).
9. Anonymous. 14th International Kidney Cancer Symposium. *BJU International Conference: 14th International Kidney Cancer Symposium Miami, FL United States Conference Publication:*. 2015;**116**(no pagination).
10. Aslam I, Sandoval L, Feldman S. What's new in the topical treatment of allergic skin diseases. *Curr Opin Allergy Clin Immunol*. 2014;**14**(5):436-50.
11. Barfield A, Brown H, Pernell P, Woodard J. Effectiveness of emollient therapy in pediatric patients with atopic dermatitis. *J Dermatol Nurses Assoc*. 2017;**9**(3):123-8.
12. Batchelor J, Grindlay D, Williams H. What's new in atopic eczema? An analysis of systematic reviews published in 2008 and 2009. *Clin Exp Dermatol*. 2010;**35**(8):823-7; quiz 7.
13. Bath-Hextall F, Birnie A, Ravenscroft J, Williams H. Interventions to reduce Staphylococcus aureus in the management of atopic eczema: An updated Cochrane review. *Br J Dermatol*. 2010;**163**(1):12-26.
14. Bigby M. A thorough systematic review of treatments for atopic eczema. *Arch Dermatol*. 2001;**137**(12):1635-6.
15. Bonchak J, Thareja S, Chen S, Quave C. Botanical Complementary and Alternative Medicine for Pruritus: a Systematic Review. *Curr Dermatol Rep*. 2017;**6**(4):248-55.
16. AJ B, FJ BH, JC R, HC W. Interventions to reduce Staphylococcus aureus in the management of atopic eczema. *Cochrane Database of Systematic Reviews*. 2008(3):CD003871.
17. Boucher M. Tacrolimus ointment for the treatment of atopic dermatitis (Structured abstract)2001; (4):[4 p.]. Available from: http://onlinelibrary.wiley.com/o/cochrane/clhta/articles/HTA-32001000957/frame.html.
18. Broersen L, Pereira A, Jorgensen J, Dekkers O. Adrenal insufficiency in corticosteroids use: Systematic review and meta-analysis. *J Clin Endocrinol Metab* 2015;**100**(6):2171-80.
19. Cameron F. 'Down to skin and bone'. *Australas J Dermatol*. 2000;**41**(3):146-8.
20. Carbone A, Siu A, Patel R. Pediatric atopic dermatitis: A review of the medical management. *Ann Pharmacother*. 2010;**44**(9):1448-58.
21. Chavigny J. The place of therapeutic education in the treatment of atopic dermatitis in children. [French]. *Ann Dermatol Venereol*. 2005;**132**(SPEC. ISS. 1):1S116-1S20.
22. Chi C, Lee C, Wojnarowska F, Kirtschig G. Safety of topical corticosteroids in pregnancy. *Cochrane Database of Systematic Reviews*. 2009;**(3) (no pagination)**(CD007346).

For peer review only

23. Chi C-C, Wang S-H, Wojnarowska F, Kirtschig G, Davies E, Bennett C. Safety of topical corticosteroids in pregnancy2015; (10). Available from: http://onlinelibrary.wiley.com/doi/10.1002/14651858.CD007346.pub3/abstract.
24. Chia B, Tey H. Systematic review on the efficacy, safety, and cost-effectiveness of topical calcineurin inhibitors in atopic dermatitis. *Dermatitis*. 2015;**26**(3):122-32.
25. Chu A, Munn S. Fluticasone propionate in the treatment of inflammatory dermatoses. *Br J Clin Pract*. 1995;**49**(3):131-3.
26. Conroy S. New products for eczema. *Arch Dis Child Educ Pract Ed*. 2004;**89**(1):ep23-ep6.
27. Das A, Panda S. Use of Topical Corticosteroids in Dermatology: An Evidence-based Approach. *Indian J Dermatol*. 2017;**62**(3):237-50.
28. El-Batawy M, Bosseila M, Mashaly H, Hafez V. Topical calcineurin inhibitors in atopic dermatitis: a systematic review and meta-analysis. *J Dermatol Sci*. 2009;**54**(2):76-87.
29. Fleischer Jr A, Boguniewicz M. An approach to pruritus in atopic dermatitis: A critical systematic review of the tacrolimus ointment literature. *J Drugs Dermatol*. 2010;**9**(5):488-98.
30. Frohna J. Efficacy and tolerability of topical pimecrolimus and tacrolimus in the treatment of atopic dermatitis: Meta-analysis of randomised controlled trials. *J Pediatr*. 2005;**147**(1):126.
31. Froschl B, Arts D, Leopold C. Corticosteroid therapy in the treatment of pediatric patients with atopic dermatitis. *GMS Health Technol Assess*. 2007;**3**:Doc09.
32. Furue M. [Topical steroids and tacrolimus for the treatment of atopic dermatitis]. *Fukuoka Igaku Zasshi*. 2006;**97**(10):285-92.
33. Furue M, Uchi H, Moroi Y, Ogawa S, Nakahara T, Urabe K. Topical tacrolimus in the management of atopic dermatitis in Japan. *Dermatol Ther*. 2006;**19**(2):118-26.
34. Garside R, Stein K, Castelnuovo E, Pitt M, Ashcroft D, Dimmock P, et al. The effectiveness and cost-effectiveness of pimecrolimus and tacrolimus for atopic eczema: A systematic review and economic evaluation. *Health Technol Assess*. 2005;**9**(29):iii-122.
35. Ghajar DL, Wood Heickman LK, Conaway M, Rogol AD. Low Risk of Adrenal Insufficiency After Use of Low- to Moderate-Potency Topical Corticosteroids for Children With Atopic Dermatitis. *Clin Pediatr (Phila)*. 2019;**58**(4):406-12.
36. Goustas P, Cork M, Higson D. EumovateTM (clobetasone butyrate 0.05%) cream: A review of clinical efficacy and safety. *J Dermatolog Treat*. 2003;**14**(2):71-85.
37. Green C, Colquitt J, Kirby J, Davidson P. Topical corticosteroids for atopic eczema: Clinical and cost effectiveness of once-daily vs. more frequent use. *Br J Dermatol*. 2005;**152**(1):130-41.
38. Green C, Colquitt J, Kirby J, Davidson P, Payne E. Clinical and cost-effectiveness of once-daily versus more frequent use of same potency topical corticosteroids for atopic eczema: a systematic review and economic evaluation (Structured abstract)2004; (4):[1 p.]. Available from: http://onlinelibrary.wiley.com/o/cochrane/clhta/articles/HTA-32004000840/frame.html.
39. Halling-Overgaard A, Kezic S, Jakasa I, Engebretsen K, Maibach H, Thyssen J. Skin absorption through atopic dermatitis skin: a systematic review. *Br J Dermatol*. 2017;**177**(1):84-106.
40. Tacrolimus and pimecrolimus for atopic eczema (Structured abstract)2004; (4):[45 p.]. Available from: http://onlinelibrary.wiley.com/o/cochrane/clhta/articles/HTA-32004000795/frame.html.
41. Pimecrolimus (Elidel (R)) for atopic dermatitis (Structured abstract)2004; (4). Available from: http://onlinelibrary.wiley.com/o/cochrane/clhta/articles/HTA-32004000175/frame.html.
42. New products for atopic dermatitis - horizon scanning review (Structured abstract)2001; (4):[5 p.]. Available from: http://onlinelibrary.wiley.com/o/cochrane/clhta/articles/HTA-32002000841/frame.html.
43. Frequency of application of topical corticosteroids for atopic eczema (Structured abstract)2004; (4):[34 p.]. Available from: http://onlinelibrary.wiley.com/o/cochrane/clhta/articles/HTA-32004000794/frame.html.

For peer review only

44. Hannuksela M. [What is the best treatment for atopic dermatitis?]. *Duodecim*. 2000;**116**(21):2321-2.
45. Hebert A. Review of pimecrolimus cream 1% for the treatment of mild to moderate atopic dermatitis. *Clin Ther*. 2006;**28**(12):1972-82.
46. Hoare C, Li WPA, Williams H. Systematic review of treatments for atopic eczema (Structured abstract)2000; 4(37):[1-191 pp.]. Available from: http://onlinelibrary.wiley.com/o/cochrane/cldare/articles/DARE-12001008089/frame.html.
47. Hon KL, Chan BC, Leung PC. Chinese herbal medicine research in eczema treatment. *Chin Med*. 2011;**6 (no pagination)**(17).
48. Hulshof L, van't Land B, Sprikkelman A, Garssen J. Role of microbial modulation in management of atopic dermatitis in children. *Nutrients*. 2017;**9** (8):pii: E854.
49. Hussain Z, Sahudin S, Thu H, Shuid A, Bukhari S, Kumolosasi E. Recent Advances in Pharmacotherapeutic Paradigm of Mild to Recalcitrant Atopic Dermatitis. *Crit Rev Ther Drug Carrier Syst*. 2016;**33**(3):213-63.
50. Kaufman B, Alexis A. Safety and efficacy of topical calcineurin inhibitors and topical steroids in atopic dermatitis in skin of color: A systematic review. *Exp Dermatol* 2016;**25**:50.
51. Legendre L, Barnetche T, Juliette M, Meyer N, Paul C. Risk of lymphoma in atopic dermatitis: A systematic review and metaanalysis of epidemiologic studies. *J Am Acad Dermatol*. 2015;**1**:AB75.
52. Li A, Yin E, Antaya R. Topical corticosteroid phobia and fear in atopic dermatitis: A systematic review. *J Invest Dermatol*. 2017;**137 (5 Supplement 1)**:S26.
53. Li A, Yin E, Antaya R. Topical corticosteroid phobia in atopic dermatitis: A systematic review. *JAMA Dermatol*. 2017;**153**(10):1036-42.
54. Meffert H, Schuppler J. Methylprednisolone aceponate lotion for the treatment of acute eczema. [German]. *H+G Zeitschrift fur Hautkrankheiten*. 1999;**74**(2):89-94.
55. Mooney E, Rademaker M, Dailey R, Daniel B, Drummond C, Fischer G, et al. Adverse effects of topical corticosteroids in paediatric eczema: Australasian consensus statement. *Australas J Dermatol*. 2015;**56**(4):241-51.
56. Murashkin N, Materikin A, Ambarchyan E, Epishev R. Current views on the pathogenesis and principles of external treatment of atopic dermatitis in children. [Russian]. *Voprosy Sovremennoi Pediatrii - Current Pediatrics*. 2016;**15**(6):584-9.
57. Nankervis H, Delamere F, Williams H. Systematic review of treatments for eczema informs clinical practice and highlights research gaps. *J Invest Dermatol*. 2013;**133**:S176.
58. Nankervis H, Thomas K, Delamere F, Barbarot S, Rogers N, Williams H. Programme Grants for Applied Research. 2016.
59. Nankervis H, Thomas K, Delamere F, Barbarot S, Smith S, Rogers N, et al. What is the evidence base for atopic eczema treatments? A summary of published randomized controlled trials. *Br J Dermatol*. 2017;**176**(4):910-27.
60. Novak N. Allergen specific immunotherapy for atopic dermatitis. *Curr Opin Allergy Clin Immunol*. 2007;**7**(6):542-6.
61. Orlow S. Topical calcineurin inhibitors in pediatric atopic dermatitis: a critical analysis of current issues. *Paediatr Drugs*. 2007;**9**(5):289-99.
62. Pan M, Heinecke G, Bernardo S, Tsui C, Levitt J. Urea: A comprehensive review of the clinical literature. *Dermatol Online J*. 2013;**19**(11).
63. Park-Wyllie L, Mazzotta P, Pastuszak A, Moretti M, Beique L, Hunnisett L, et al. Birth defects after maternal exposure to corticosteroids: prospective cohort study and meta-analysis of epidemiological studies (Structured abstract)2000; 62(6):[385-92 pp.]. Available from: http://onlinelibrary.wiley.com/o/cochrane/cldare/articles/DARE-12001003037/frame.html.
64. Payne J, Habet KA, Pona A, Feldman SR. A Review of Topical Corticosteroid Foams. *J Drugs Dermatol*. 2019;**18**(8):756-70.
65. Phipatanakul W. Efficacy and tolerability of pimecrolimus and tacrolimus in the treatment of atopic dermatitis: Meta-analysis of randomised controlled trials. Commentary. *Pediatrics*. 2006;**118**(SUPPL. 1):S16-S7.

For peer review only

66. Radovic T, Kostovic K, Ceovic R, Mokos Z. Topical calcineurin inhibitors and malignancy risk. *Int J Cancer Manag*. 2017;**10 (4) (no pagination)**(e6173).
67. Ricci G, Dondi A, Patrizi A. Role of topical calcineurin inhibitors on atopic dermatitis of children. *Curr Med Chem*. 2007;**14**(14):1579-91.
68. Ruzicka T, Assmann T, Homey B. Tacrolimus: The drug for the turn of the millennium? *Arch Dermatol*. 1999;**135**(5):574-80.
69. Sanchez J, Paez B, Macias A, Olmos C, De Falco A. Atopic dermatitis guideline. Position paper from the Latin American Society of allergy, asthma and immunology. *Revista Alergia Mexico*. 2014;**61**(3):178-211.
70. Schiffner R, Schiffner-Rohe J, Landthaler M, Stolz W. Treatment of atopic dermatitis and impact on quality of life: a review with emphasis on topical non-corticosteroids. *Pharmacoeconomics*. 2003;**21**(3):159-79.
71. Schmitt J, Apfelbacher C, Flohr C. Eczema. *BMJ Clin Evid*. 2011(pagination).
72. Schmitt J, Kobyletzki L, Svensson A, Apfelbacher C. Efficacy and tolerability of proactive treatment with topical corticosteroids and calcineurin inhibitors for atopic eczema: systematic review and meta-analysis of randomized controlled trials (Provisional abstract)2011; 164(2):[415-28 pp.]. Available from: http://onlinelibrary.wiley.com/o/cochrane/cldare/articles/DARE-12011001466/frame.html.
73. Sher L, Chang J, Patel I, Balkrishnan R, Fleischer A. Relieving the pruritus of atopic dermatitis: A meta-analysis. *Value in Health*. 2012;**15 (4)**:A249-A50.
74. Sher LG, Chang J, Patel I, Balkrishnan R, Fleischer Jr A. Relieving the pruritus of atopic dermatitis: A meta-analysis. *Acta Derm Venereol*. 2012;**92**(5):455-61.
75. Siegfried E, Jaworski J, Hebert A. Topical calcineurin inhibitors and lymphoma risk: evidence update with implications for daily practice. *Am J Clin Dermatol*. 2013;**14**(3):163-78.
76. Siegfried E, Jaworski J, Mina-Osorio P. A Systematic Scoping Literature Review of Publications Supporting Treatment Guidelines for Pediatric Atopic Dermatitis in Contrast to Clinical Practice Patterns. *Dermatol Ther (Heidelb)*. 2018;**8**(3):349-77.
77. Silverberg J. Atopic dermatitis: An evidence-based treatment update. *Am J Clin Dermatol*. 2014;**15**(3):149-64.
78. Simpson E. Atopic dermatitis: A review of topical treatment options. *Curr Med Res Opin*. 2010;**26**(3):633-40.
79. Spada F, Barnes T, Greive K. Comparative safety and efficacy of topical mometasone furoate with other topical corticosteroids. *Australas J Dermatol*. 2018;**59**(3):e168-e74.
80. Torii H. The Evaluation of Tacrolimus on Evidence-based Medicine (EBM). [Japanese]. *Skin Research*. 2003;**2**(SUPPL. 3):27-30.
81. Torley D, Futamura M, Williams H, Thomas K. What's new in atopic eczema? An analysis of systematic reviews published in 2010-11. *Clin Exp Dermatol*. 2013;**38**(5):449-56.
82. Uppal SK, Chat VS, Kearns DG, Wu JJ. Topical Agents Currently in Phase II or Phase III Trials for Atopic Dermatitis. *J Drugs Dermatol*. 2020;**19**(10):956-9.
83. van Zuuren E, Fedorowicz Z, Arents B. Emollients and moisturisers for eczema: abridged Cochrane systematic review including GRADE assessments. *Br J Dermatol*. 2017.
84. Wat H, Dytoc M. Off-label uses of topical vitamin D in dermatology: A systematic review. *J Cutan Med Surg*. 2014;**18**(2):91-108.
85. Wellington K, Noble S. Pimecrolimus: A review of its use in atopic dermatitis. *Am J Clin Dermatol*. 2004;**5**(6):479-95.
86. Williams H. Established corticosteroid creams should be applied only once daily in patients with atopic eczema. *BMJ*. 2007;**334**(7606):1272.
87. Williams H, Grindlay D. What's new in atopic eczema? An analysis of the clinical significance of systematic reviews on atopic eczema published in 2006 and 2007. *Clin Exp Dermatol*. 2008;**33**(6):685-8.

For peer review only

88. Williams H, Grindlay D. What's new in atopic eczema? An analysis of systematic reviews published in 2007 and 2008. Part 2. Disease prevention and treatment. *Clin Exp Dermatol*. 2010;**35**(3):223-7.
89. Wollenberg A, Barbarot S, Bieber T, Christen-Zaech S, Deleuran M, Fink-Wagner A, et al. Consensus-based European guidelines for treatment of atopic eczema (atopic dermatitis) in adults and children: part I. *J Eur Acad Dermatol Venereol*. 2018;**32**(5):657-82.

For peer review only

Appendix 3: Prospero search

Prospero – searched up to 23rd March 2021 – search results from 'Eczema OR dermatitis'

Potentially relevant ongoing systematic reviews:

Prospero ID	Date registered	Title	Status	Anticipated completion date
CRD42015016525 (1)	11 February 2015	Effects of emollients in the management of atopic dermatitis in pediatric patients a systemic review and meta-analysis	Review ongoing	25 February 2016
CRD42015027873 (2)	04 November 2015	Interventions to improve quality of life in paediatric atopic dermatitis: a systematic review	Review ongoing	01 January 2016
CRD42020190452 (3)	14 July 2020	The association between topical calcineurin inhibitor use and risk of cancer: a systematic review and meta-analysis	Review ongoing	31 August 2020
CRD42020161558 (4)	28 April 2020	Efficacy of Non-Steroidal Topical Therapies for Atopic Dermatitis: A Systematic Review & Meta-Analysis	Review ongoing	31 May 2020
CRD42021230047 (5)	31 October 2021	A network meta-analysis of five categories of external therapy of traditional Chinese for common diseases of dermatology	Review ongoing	31 October 2021

1. Tan Q, Tan C, Peng W, Shi Y, Xia L. Effects of emollients in the management of atopic dermatitis in pediatric patients a systemic review and meta-analysis [CRD42015016525] 2015 (accessed: 27/03/21). Available from: http://www.crd.york.ac.uk/PROSPERO/display_record.php?ID=CRD42015016525.
2. Yu A, Hong J, Lee M, Hong B. Interventions to improve quality of life in paediatric atopic dermatitis: a systematic review [CRD42015027873] 2015 (accessed: 27/03/21). Available from: http://www.crd.york.ac.uk/PROSPERO/display_record.php?ID=CRD42015027873.
3. Lam M, Zhu J, Tadrous M, Drucker A. The association between topical calcineurin inhibitor use and risk of cancer: a systematic review and meta-analysis [CRD42020190452]2020 (accessed: 27/03/2021). Available from: www.crd.york.ac.uk/prospero/display_record.php?RecordID=190452.
4. Lee K. Efficacy of Non-Steroidal Topical Therapies for Atopic Dermatitis: A Systematic Review & Meta-Analysis [CRD42020161558] 2020 (accessed: 27/03/2021). Available from: www.crd.york.ac.uk/prospero/display_record.php?RecordID=161558.
5. Ruirui L, Jing G, Dingxi B, Qian Y, Lin Z, Zhi Y, et al. A network meta-analysis of five categories of external therapy of traditional Chinese for common diseases of dermatology [CRD42021230047]2021 (assessed: 27/03/2021). Available from: www.crd.york.ac.uk/prospero/display_record.php?RecordID=230047.

Appendix 4: AMSTAR 2 ratings [1]:

Review ID	1 PICO	2 Protocol*	3 Study designs	4 Search strategy*	5 Duplicate screening	6 Duplicate data extraction	7 Excluded studies*	8 Included studies	9 Risk of bias assessed*	10 Funding of studies	11 Appropriate meta-analysis*	12 Risk of bias in meta-analysis	13 Risk of bias in discussion *	14 Hetero-geneity	15 Publication bias in meta-analysis*	16 Reviewers' conflict of interest	Overall rating
Ashcroft 2005 [2]	Yes	No	No	Yes	Yes	Yes	No	Partial yes	Partial yes	No	Yes	No	No	No	No	Yes	Critically low
Ashcroft 2007 [3]	Yes	Partial yes	No	Yes	Yes	Yes	Yes	Partial yes	Yes	No	N/A	N/A	Yes	Yes	N/A	Yes	Moderate
Barnes 2015 [4]	No	No	No	No	No	No	No	No	No	No	N/A	N/A	No	No	N/A	Yes	Critically low
Braham 2010 [5]	No	No	Yes	No	No	No	No	Partial yes	No	No	N/A	N/A	No	No	N/A	Yes	Critically low
Broeders 2016 [6]	Yes	No	No	Partial yes	No	No	No	Partial yes	Partial yes	Yes	No	No	No	No	Yes	Yes	Critically low
Callen 2007 [7]	Yes	No	No	No	No	No	No	No	No	No	N/A	N/A	No	No	N/A	Yes	Critically low
Chen 2010 [8]	Yes	No	No	Partial yes	No	No	No	Partial yes	Yes	Yes	N/A	N/A	No	No	N/A	Yes	Critically low
Cury Martins 2015 [9]	Yes	Yes	No	Yes	Yes	Yes	Yes	Partial yes	Yes	No	Yes	Yes	Yes	No	Yes	Yes	Moderate
De Tiedra 1997 [10]	No	No	No	No	No	No	No	Partial yes	No	No	N/A	N/A	No	No	N/A	No	Critically low
Devillers 2006 [11]	No	No	Yes	No	No	No	No	Partial yes	No	No	N/A	N/A	No	No	N/A	Yes	Critically low
Dong 2017 [12]	Yes	No	No	No	Yes	Yes	No	No	Yes	No	No	No	No	No	No	No	Critically low
Eichenfield 2014 [13]	No	No	Yes	No	No	No	No	No	No	No	N/A	N/A	No	No	N/A	Yes	Critically low
Feldman 2005 [14]	No	No	Yes	No	No	No	No	Partial yes	No	No	N/A	N/A	No	No	N/A	No	Critically low
Fishbein 2019 [15]	Yes	Partial yes	Yes	Partial yes	Yes	Yes	No	Yes	No	Yes	No	No	No	No	No	Yes	Critically low
Frangos 2008 [16]	No	No	No	No	No	No	No	No	No	No	N/A	N/A	No	No	N/A	No	Critically low
Froeschl 2007 [17]	No	No	No	No	No	No	Yes	Partial yes	No	Yes	N/A	N/A	No	No	N/A	No	Critically low

For peer review only

Gonzalez-Lopez 2017 [18]	Yes	No	No	Yes	Yes	Yes	No	Yes	Yes	Yes	Yes	Yes	Yes	Yes	Yes	Yes	Critically low
Green 2004 [19]	Yes	Partial Yes	No	No	Partial yes	Yes	Partial yes	Yes	Partial yes	Yes	N/A	N/A	Yes	Yes	N/A	Yes	Low
Gu 2013 [20]	Yes	Yes	No	Yes	Yes	Yes	Yes	Yes	Yes	Yes	Yes	Yes	Yes	Yes	Yes	Yes	High
Gu 2014 [21]	Yes	No	No	No	No	No	No	No	Yes	No	Yes	No	Yes	Yes	No	Yes	Critically low
Hajar 2015 [22]	Yes	Yes	Yes	No	Yes	Yes	No	No	Partial yes	No	N/A	N/A	No	No	N/A	Yes	Critically low
Hoare 2000 [23]	Yes	No	Yes	Yes	No	No	Yes	Partial yes	Partial yes	No	N/A	N/A	Yes	No	N/A	Yes	Low
Iskedjian 2004 [24]	Yes	No	No	Partial yes	Yes	Yes	No	Partial yes	Partial yes	No	N/A	N/A	No	No	N/A	No	Critically low
Juhasz 2017 [25]	No	No	No	No	No	No	No	No	No	N/A	N/A	N/A	No	No	N/A	Yes	Critically low
Labedz 2019 [26]	Yes	No	Yes	Yes	No	No	No	Partial yes	Yes	No	Yes	No	No	No	No	Yes	Critically low
Legendre 2015 [27]	Yes	No	No	No	Yes	Yes	No	Partial yes	Yes	No	Yes	No	No	Yes	Yes	Yes	Critically low
Li 2007 [28]	Yes	No	No	Partial yes	No	Yes	No	No	Partial yes	No	N/A	N/A	No	No	N/A	No	Critically low
Nankervis 2016 [29]	Yes	Partial yes	Yes	Partial yes	No	Yes	No	Partial yes	Partial yes	Yes	N/A	N/A	Yes	No	N/A	Yes	Low
Penaloza Hidalgo 2004 [30]	Yes	Partial yes	No	Yes	No	No	Yes	Partial yes	Partial yes	Yes	N/A	N/A	No	Yes	N/A	Yes	Low
Schmitt 2011 [31]	Yes	Partial yes	No	Partial yes	Yes	Yes	No	Yes	Yes	No	N/A	N/A	No	Yes	N/A	Yes	Critically low
Sidbury 2011 [32]	No	No	Yes	No	No	No	No	No	No	No	N/A	N/A	No	No	N/A	Yes	Critically low
Siegfried 2016 [33]	No	No	No	No	No	No	No	No	No	No	N/A	N/A	No	No	N/A	Yes	Critically low
Singh 2012 [34]	No	No	No	No	No	No	Partial yes	No	Partial yes	No	N/A	N/A	Yes	No	N/A	Yes	Critically low
Svensson 2011 [35]	Yes	No	No	Partial yes	Yes	No	No	Partial yes	Partial yes	No	Yes	No	No	Yes	No	Yes	Critically low
Tang 2014 [36]	Yes	No	No	Partial yes	No	No	No	No	No	No	N/A	N/A	No	No	N/A	Yes	Critically low
van Zuuren 2017 [37]	Yes	Yes	No	Partial yes	Yes	Yes	Yes	Yes	Yes	Yes	Yes	Yes	Yes	Yes	Yes	Yes	Moderate
Wood Heickman 2017 [38]	Yes	No	Yes	No	Yes	No	No	No	No	No	Yes	No	No	Yes	No	Yes	Critically low

Yan 2008 [39]	Yes	No	No	Partial yes	No	No	No	No	Partial yes	Yes	Yes	No	No	No	No	No	Critically low

Footnotes: AMSTAR 2 domains

1 Did the research questions and inclusion criteria for the review include the components of PICO?

**2 Did the report of the review contain an explicit statement that the review methods were established prior to the conduct of the review and did the report justify any significant deviations from the protocol?*

3 Did the review authors explain their selection of the study designs for inclusion in the review?

**4 Did the review authors use a comprehensive literature search strategy?*

5 Did the review authors perform study selection in duplicate?

6 Did the review authors perform data extraction in duplicate?

**7 Did the review authors provide a list of excluded studies and justify the exclusions?*

8 Did the review authors describe the included studies in adequate detail?

**9 Did the review authors use a satisfactory technique for assessing the risk of bias (RoB) in individual studies that were included in the review?*

10 Did the review authors report on the sources of funding for the studies included in the review?

**11 If meta-analysis was performed, did the review authors use appropriate methods for statistical combination of results?*

12 If meta-analysis was performed, did the review authors assess the potential impact of RoB in individual studies on the results of the meta-analysis or other evidence synthesis?

**13 Did the review authors account for RoB in primary studies when interpreting/discussing the results of the review?*

14 Did the review authors provide a satisfactory explanation for, and discussion of, any heterogeneity observed in the results of the review?

**15 If they performed quantitative synthesis did the review authors carry out an adequate investigation of publication bias (small study bias) and discuss its likely impact on the results of the review?*

16 Did the review authors report any potential sources of conflict of interest, including any funding they received for conducting the review?

NB domains marked * in the table and footnotes are critical domains.

For peer review only

1. Shea BJ, Reeves BC, Wells G, Thuku M, Hamel C, Moran J, et al. AMSTAR 2: a critical appraisal tool for systematic reviews that include randomised or non-randomised studies of healthcare interventions, or both. *BMJ*. 2017;**358**:j4008.
2. Ashcroft D, Dimmock P, Garside R, Stein K, Williams H. Efficacy and tolerability of topical pimecrolimus and tacrolimus in the treatment of atopic dermatitis: Meta-analysis of randomised controlled trials. *BMJ* 2005;**330**(7490):516-22.
3. Ashcroft DM, Chen L-C, Garside R, Stein K, Williams HC. Topical pimecrolimus for eczema Cochrane Database Syst Rev [Internet]. 2007; (4). Available from: http://onlinelibrary.wiley.com/doi/10.1002/14651858.CD005500.pub2/abstract.
4. Barnes L, Kaya G, Rollason V. Topical Corticosteroid-Induced Skin Atrophy: A Comprehensive Review. *Drug Saf*. 2015;**38**(5):493-509.
5. Braham S, Pugashetti R, Koo J, Maibach H. Occlusive therapy in atopic dermatitis: overview. *J Dermatolog Treat*. 2010;**21**(2):62-72.
6. Broeders J, Ahmed Ali U, Fischer G. Systematic review and meta-analysis of randomized clinical trials (RCTs) comparing topical calcineurin inhibitors with topical corticosteroids for atopic dermatitis: A 15-year experience. *J Am Acad Dermatol*. 2016;**75**(2):410-9.e3.
7. Callen J, Chamlin S, Eichenfield L, Ellis C, Girardi M, Goldfarb M, et al. A systematic review of the safety of topical therapies for atopic dermatitis. *Br J Dermatol*. 2007;**156**(2):203-21.
8. Chen S, Yan J, Wang F. Two topical calcineurin inhibitors for the treatment of atopic dermatitis in pediatric patients: A meta-analysis of randomized clinical trials. *J Dermatolog Treat*. 2010;**21**(3):144-56.
9. Cury Martins J, Martins C, Aoki V, Gois AF, Ishii HA, da SEM. Topical tacrolimus for atopic dermatitis. Cochrane Database Syst Rev [Internet]. 2015; (7). Available from: http://onlinelibrary.wiley.com/doi/10.1002/14651858.CD009864.pub2/abstract.
10. de Tiedra A, Mercadal J, Lozano R. Prednicarbate versus fluocortin for inflammatory dermatoses: A cost-effectiveness study. *PharmacoEconomics*. 1997;**12**(2 Pt 1):193-208.
11. Devillers A, Oranje A. Efficacy and safety of 'wet-wrap' dressings as an intervention treatment in children with severe and/or refractory atopic dermatitis: a critical review of the literature. *Br J Dermatol*. 2006;**154**(4):579-85.
12. Dong Y, Zeng W, Li W, Ma H, Zheng W. Efficacy and safety of topical tacrolimus for childhood atopic dermatitis; a meta-analysis. [Chinese]. *J Clin Dermatol*. 2017;**46**(4):239-42.
13. Eichenfield L, Tom W, Berger T, Krol A, Paller A, Schwarzenberger K, et al. Guidelines of care for the management of atopic dermatitis: Section 2. Management and treatment of atopic dermatitis with topical therapies. *J Am Acad Dermatol*. 2014;**71**(1):116-32.
14. Feldman S. Relative efficacy and interchangeability ofvarious clobetasol propionate vehicles in the management of steroid-responsive dermatoses. *Curr Ther Res Clin Exp*. 2005;**66**(3):154-71.
15. Fishbein AB, Mueller K, Lor J, Smith P, Paller AS, Kaat A. Systematic Review and Meta-analysis Comparing Topical Corticosteroids With Vehicle/Moisturizer in Childhood Atopic Dermatitis. *J Pediatr Nurs*. 2019;**47**:36-43.

For peer review only

16. Frangos J, Kimball A. Clobetasol propionate emollient formulation foam in the treatment of corticosteroid-responsive dermatoses. *Expert Opin Pharmacother*. 2008;**9**(11):2001-7.
17. Froeschl B, Arts D, Leopold C. Corticosteroid therapy in the treatment of pediatric patients with atopic dermatitis (Structured abstract). Health Technol Assess [Internet]. 2007; (4). Available from: http://onlinelibrary.wiley.com/o/cochrane/clhta/articles/HTA-32008100208/frame.html.
18. Gonzalez-Lopez G, Ceballos-Rodriguez R, Gonzalez-Lopez J, Feito Rodriguez M, Herranz-Pinto P. Efficacy and safety of wet wrap therapy for patients with atopic dermatitis: a systematic review and meta-analysis. *Br J Dermatol*. 2017;**177**(3):688-95.
19. Green C, Colquitt J, Kirby J, Davidson P, Payne E. Clinical and cost-effectiveness of once-daily versus more frequent use of same potency topical corticosteroids for atopic eczema: a systematic review and economic evaluation. *Health Technol Assess (Winchester, England)*. 2004;**8**(47):iii,iv, 1-120.
20. Gu S, Yang AW, Xue CC, Li CG, Pang C, Zhang W, et al. Chinese herbal medicine for atopic eczema. Cochrane Database Syst Rev [Internet]. 2013; (9). Available from: http://onlinelibrary.wiley.com/doi/10.1002/14651858.CD008642.pub2/abstract.
21. Gu S, Yang A, Li C, Lu C, Xue C. Topical application of Chinese herbal medicine for atopic eczema: A systematic review with a meta-analysis. *Dermatology*. 2014;**228**(4):294-302.
22. Hajar T, Leshem Y, Hanifin J, Nedorost S, Lio P, Paller A, et al. A systematic review of topical corticosteroid withdrawal ("steroid addiction") in patients with atopic dermatitis and other dermatoses. *J Am Acad Dermatol*. 2015;**72**(3):541-9.e2.
23. Hoare C, Li Wan Po A, Williams H. Systematic review of treatments for atopic eczema. *Health Technol Assess*. 2000;**4**(37):1-191.
24. Iskedjian M, Piwko C, Shear N, Langley R, Einarson T. Topical calcineurin inhibitors in the treatment of atopic dermatitis: A meta-analysis of current evidence. *Am J Clin Dermatol*. 2004;**5**(4):267-79.
25. Juhász ML, Curley RA, Rasmussen A, Malakouti M, Silverberg N, Jacob SE. Systematic Review of the Topical Steroid Addiction and Topical Steroid Withdrawal Phenomenon in Children Diagnosed With Atopic Dermatitis and Treated With Topical Corticosteroids. *J Dermatol Nurses Assoc*. 2017;**9**(5):233-40.
26. Łabędź N, Pawliczak R. Efficacy and safety of topical calcineurin inhibitors for the treatment of atopic dermatitis: meta-analysis of randomized clinical trials. *Postepy Dermatol Alergol*. 2019;**36**(6):752-9.
27. Legendre L, Barnetche T, Mazereeuw-Hautier J, Meyer N, Murrell D, Paul C. Risk of lymphoma in patients with atopic dermatitis and the role of topical treatment: A systematic review and meta-analysis. *J Am Acad Dermatol*. 2015;**72**(6):992-1002.
28. Li R, Zhu H, Fan L, Ni S, Feng C, Wu Z. Efficacy and tolerability of topical tacrolimus in the treatment of atopic dermatitis: A systematic review of randomized controlled trials. [Chinese]. *J Clin Dermatol*. 2007;**36**(12):757-60.
29. Nankervis H, Thomas K, Delamere F, Barbarot S, Rogers N, Williams H. Scoping systematic review of treatments for eczema2016 2016/05/None.
30. Penaloza Hidalgo B, Knight T, Burls A. A systematic review of effectiveness and cost effectiveness of tacrolimus ointment for topical treatment of atopic dermatitis in adults and children Health Technol Assess [Internet]. 2004; (4):[81 p.].
31. Schmitt J, Von Kobyletzki L, Svensson A, Apfelbacher C. Efficacy and tolerability of proactive treatment with topical corticosteroids and calcineurin inhibitors for atopic eczema: Systematic review and meta-analysis of randomized controlled trials. *Br J Dermatol*. 2011;**164**(2):415-28.
32. Sidbury R, Tom W, Bergman J, Cooper K, Silverman R, Berger T, et al. Guidelines of care for the management of atopic dermatitis: Section 4. Prevention of disease flares and use of adjunctive therapies and approaches. *J Am Acad Dermatol*. 2014;**71**(6):1218-33.

For peer review only

33. Siegfried E, Jaworski J, Kaiser J, Hebert A. Systematic review of published trials: Long-term safety of topical corticosteroids and topical calcineurin inhibitors in pediatric patients with atopic dermatitis. *BMC Pediatr*. 2016;**16** (75).

34. Singh S, Mann B. Clinical utility of clocortolone pivalate for the treatment of corticosteroid-responsive skin disorders: A systematic review. *Clin Cosmet Investig Dermatol*. 2012;**5**:61-8.

35. Svensson A, Chambers C, Gånemo A, Mitchell S. A systematic review of tacrolimus ointment compared with corticosteroids in the treatment of atopic dermatitis. *Curr Med Res Opin*. 2011;**27**(7):1395-406.

36. Tang T, Bieber T, Williams H. Are the concepts of induction of remission and treatment of subclinical inflammation in atopic dermatitis clinically useful? *J Allergy Clin Immunol*. 2014;**133**(6):1615-25.e1.

37. van Zuuren EJ, Fedorowicz Z, Christensen R, Lavrijsen AP, Arents BW. Emollients and moisturisers for eczema. Cochrane Database Syst Rev [Internet]. 2017; (2). Available from: http://onlinelibrary.wiley.com/doi/10.1002/14651858.CD012119.pub2/abstract.

38. Wood Heickman L, Davallow Ghajar L, Conaway M, Rogol A. Evaluation of Hypothalamic-Pituitary-Adrenal Axis Suppression following Cutaneous Use of Topical Corticosteroids in Children: A Meta-Analysis. *Horm Res Paediatr*. 2018;**89**(6):389-96.

39. Yan J, Chen S, Wang X, Zhou W, Wang F. Meta-analysis of tacrolimus ointment for atopic dermatitis in pediatric patients. *Pediatr Dermatol*. 2008;**25**(1):117-20.

For peer review only

Appendix 5: Characteristics of the included systematic reviews

First author and year of publication	Type of review	Language of publication	Funding source	Conflicts of interest	RCT comparisons	Observational data included	AMSTAR-2 rating
Ashcroft 2005 (1)	Non-Cochrane	English	NHS health technology assessment programme.	None known	TCS versus TCI	No	Critically low
Ashcroft 2007 (2)	Cochrane	English	School of Pharmacy and Pharmaceutical Sciences, University of Manchester, UK.	None known	TCS versus TCI	Yes – TCI compared with TCS	Moderate
Barnes 2015 (3)	Non-Cochrane	English	No funding	None known	TCS versus vehicle<br>TCS versus TCI<br>TCS versus TCS	Yes (single arm TCS studies)	Critically low
Braham 2010 (4)	Non-Cochrane	English	No funding	One author was a speaker for a number of pharmaceutical companies	Occlusion therapy versus non-occlusion therapy	Yes (occlusive therapy, no comparison)	Critically low
Broeders 2016 (5)	Non-Cochrane	English	No funding	None known	TCS versus TCI	No	Critically low
Callen 2007 (6)	Non-Cochrane	English	Funding from EBMed. One author received funding from Novartis Corporation for the project. They declared that "Novartis Corporation played no role in the design and conduct of the study or in data collection, data management, data analysis, interpretation of the data, manuscript preparation, manuscript review or manuscript approval"	Most authors had consultancy fees and/or research support from pharmaceutical companies	TCS versus vehicle<br>TCS versus TCS	Yes (single arm TCS studies or comparing various TCS potencies)	Critically low
Chen 2010 (7)	Non-Cochrane	English	Not stated	None known	TCS versus TCI	No	Critically low
Cury Martins 2015 (8)	Cochrane	English	NIHR	None known	TCS versus TCI	Yes – TCI compared to TCS	Moderate
De Tiedra 1997 (9)	Non-Cochrane	English	Supported by Laboratorios Novag, S.A, Grupo Ferrer.	Not clear	TCS versus TCS	Yes – in most cases they only report data from one arm of an RCT	Critically low
Devillers 2006 (10)	Non-Cochrane	English	Not stated	None known	Occlusive therapy versus non-occlusive therapy	Yes – occlusive therapy (no comparison)	Critically low
Dong 2017 (11)	Non-Cochrane	Chinese	Not stated	Not clear	TCS versus TCI	No	Critically low
Eichenfield 2014 (12)	Non-Cochrane	English	No funding	Most authors served as consultants, speakers, members of the advisory	None	Yes (comparing different TCS potencies)	Critically low

				board and/or investigators for pharmaceutical companies.			
Feldman 2005 (13)	Non-Cochrane	English	Grant from Galderma Laboratories, LP, Fort Worth, Texas.	Not clear	TCS versus vehicle	No	Critically low
Fishbein 2019 (14)	Non-Cochrane	English	No funding	None known	TCS versus vehicle/moisturizer	No	Critically low
Frangos 2008 (15)	Non-Cochrane	English	Not stated	One author is an investigator for Steifel and was an investigator on two of the studies reviewed.	TCS versus vehicle	Yes (single arm studies)	Critically low
Froschl 2007 (16)	GMS HTA report	German (executive summary in English)	Not stated	Not stated	TCS versus placebo/vehicle<br>TCS versus TCS<br>TCS versus TCI	No	Critically low
Gonzalez-Lopez 2017 (17)	Non-Cochrane	English	No funding	None known	Occlusive therapy versus non-occlusive therapy	No	Critically low
Green 2004 (18)	HTA report	English	Funded by the HTA Programme on behalf of NICE	None known	Once daily versus twice daily TCS use	No	Low
Gu 2013 (19)	Cochrane	English	RMIT University<br>Nottingham University, UK.<br>NIHR	One author was a principal investigator on one included study (but this study was not relevant for this overview)	Chinese herbal medicine versus TCS	No	High
Gu 2014 (20)	Non-Cochrane	English	Not stated	None known	Chinese herbal medicine versus TCS	No	Critically low
Hajar 2015 (21)	Non-Cochrane	English	No funding	None known	No RCTs found	Yes (case series or case reports on steroid withdrawal)	Critically low
Hoare 2000 (22)	NIHR HTA report	English	HTA programme	One author received payment from Novartis for lectures on the epidemiology of atopic eczema in 1999.<br>Another author has acted as occasional lecturer or consultant for pharmaceutical companies.	TCS versus TCS<br>TCS versus vehicle	No	Low
Iskedjian 2004 (23)	Non-Cochrane	English	Funded by Fujisawa Canada Inc.	Not clear	TCS versus TCI<br>TCS versus placebo	No	Critically low
Juhasz 2017 (24)	Non-Cochrane	English	Not stated	One author had primary contact with the 2nd case and has a blog on the subject matter in this systematic review	No RCTs found	Yes (case reports on steroid withdrawal)	Critically low

For peer review only

Labedz 2019 [25]	Non-Cochrane	English	Not stated	None known	TCS versus TCI	No	Critically low
Legendre 2015 [26]	Non-Cochrane	English	No funding	One author is a consultant and investigator for two pharmaceutical companies. One author is a speaker and/or on the advisory board for five pharmaceutical companies.	Only searched for cohort or case control studies	Yes (comparing TCS and TCI)	Critically low
Li 2007 [27]	Non-Cochrane	Chinese	Not stated	Not stated	TCS versus TCI	No	Critically low
Nankervis 2016 [28]	NIHR HTA report	English	NIHR	One author reports grants and fees from a number of pharmaceutical companies.	TCS versus placebo/vehicle<br>Proactive treatment versus vehicle<br>TCS versus TCI<br>TCS versus TCS<br>Once a day versus twice a day use of TCS<br>Occlusive therapy versus non-occlusive therapy<br>TCS versus emollients	No	Low
Penzaloza Hidalgo 2004 [29]	West Midlands HTA report	English	Not stated	None known	TCS versus TCI	No	Low
Schmitt 2011 [30]	Non-Cochrane	English	No funding	One author has served as paid lecturer for a pharmaceutical company.	Proactive treatment versus vehicle	No	Critically low
Sidbury 2014 [31]	Non-Cochrane	English	Not stated	Some authors have served as investigators, consultants, speakers, and on advisory boards for pharmaceutical companies.	Proactive treatment versus vehicle	No	Critically low
Siegfried 2016 [32]	Non-Cochrane	English	Financial support for writing by Valent Pharmaceutical North America LLC. They declared that "Valeant Pharmaceuticals had no role in the design of the literature searches, or analysis and presentation of results."	Authors have either participated in paid contract research, received travel expenses for presentations, consulting fees, speakers, on advisory boards, or on data safety monitoring boards with pharmaceutical companies.	TCS versus TCS<br>TCS versus TCI<br>TCS versus vehicle	No	Critically low
Singh 2012 [33]	Non-Cochrane	English	Not stated	None known	TCS versus TCS<br>TCI versus TCS<br>TCS versus placebo/vehicle	Yes (single arm TCS study)	Critically low

For peer review only

Svensson 2011 (34)	Non-Cochrane	English	Funded by Astellas Pharma Europe Ltd.	One author was a paid employee of Astellas Pharma Europe Ltd and one author undertook paid consultancy work for Astellas Pharma Europe Ltd.	TCI versus TCS	No	Critically low
Tang 2014 (35)	Non-Cochrane	English	Not stated	One author has received lecture fees from Astellas.	Proactive treatment versus vehicle	No	Critically low
van Zuuren 2017 (36)	Cochrane	English	Oak Foundation, Denmark<br>NIHR	None known	TCS versus emollient	No	Moderate
Wood Heickman 2018 (37)	Non-Cochrane	English	No grants, honoraria or royalties were received supporting the writing of the paper.	One author was a consultant with Perrigo, Inc. with regard to topical corticosteroid treatment. All authors have no financial or other potential conflicts of interest.	Two RCTs included but analysed as observational data	Yes – single arm cohort studies	Critically low
Yan 2008 (38)	Non-Cochrane	English	Not stated	Not stated	TCI versus TCS	No	Critically low

Key: TCI=topical calcinueirin inhibitor; TCS=topical corticosteroids; RCT=Randomised Controlled Trial; NIHR= National Institute for Health Research

1. Ashcroft D, Dimmock P, Garside R, Stein K, Williams H. Efficacy and tolerability of topical pimecrolimus and tacrolimus in the treatment of atopic dermatitis: Meta-analysis of randomised controlled trials. *BMJ* 2005;**330**(7490):516-22.
2. Ashcroft DM, Chen L-C, Garside R, Stein K, Williams HC. Topical pimecrolimus for eczema Cochrane Database Syst Rev [Internet]. 2007; (4). Available from: http://onlinelibrary.wiley.com/doi/10.1002/14651858.CD005500.pub2/abstract.
3. Barnes L, Kaya G, Rollason V. Topical Corticosteroid-Induced Skin Atrophy: A Comprehensive Review. *Drug Saf*. 2015;**38**(5):493-509.
4. Braham S, Pugashetti R, Koo J, Maibach H. Occlusive therapy in atopic dermatitis: overview. *J Dermatolog Treat*. 2010;**21**(2):62-72.
5. Broeders J, Ahmed Ali U, Fischer G. Systematic review and meta-analysis of randomized clinical trials (RCTs) comparing topical calcineurin inhibitors with topical corticosteroids for atopic dermatitis: A 15-year experience. *J Am Acad Dermatol*. 2016;**75**(2):410-9.e3.
6. Callen J, Chamlin S, Eichenfield L, Ellis C, Girardi M, Goldfarb M, et al. A systematic review of the safety of topical therapies for atopic dermatitis. *Br J Dermatol*. 2007;**156**(2):203-21.
7. Chen S, Yan J, Wang F. Two topical calcineurin inhibitors for the treatment of atopic dermatitis in pediatric patients: A meta-analysis of randomized clinical trials. *J Dermatolog Treat*. 2010;**21**(3):144-56.

8. Cury Martins J, Martins C, Aoki V, Gois AF, Ishii HA, da SEM. Topical tacrolimus for atopic dermatitis. Cochrane Database Syst Rev [Internet]. 2015; (7). Available from: http://onlinelibrary.wiley.com/doi/10.1002/14651858.CD009864.pub2/abstract.
9. de Tiedra A, Mercadal J, Lozano R. Prednicarbate versus fluocortin for inflammatory dermatoses: A cost-effectiveness study. *PharmacoEconomics*. 1997;**12**(2 Pt 1):193-208.
10. Devillers A, Oranje A. Efficacy and safety of 'wet-wrap' dressings as an intervention treatment in children with severe and/or refractory atopic dermatitis: a critical review of the literature. *Br J Dermatol*. 2006;**154**(4):579-85.
11. Dong Y, Zeng W, Li W, Ma H, Zheng W. Efficacy and safety of topical tacrolimus for childhood atopic dermatitis; a meta-analysis. [Chinese]. *J Clin Dermatol*. 2017;**46**(4):239-42.
12. Eichenfield L, Tom W, Berger T, Krol A, Paller A, Schwarzenberger K, et al. Guidelines of care for the management of atopic dermatitis: Section 2. Management and treatment of atopic dermatitis with topical therapies. *J Am Acad Dermatol*. 2014;**71**(1):116-32.
13. Feldman S. Relative efficacy and interchangeability ofvarious clobetasol propionate vehicles in the management of steroid-responsive dermatoses. *Curr Ther Res Clin Exp*. 2005;**66**(3):154-71.
14. Fishbein AB, Mueller K, Lor J, Smith P, Paller AS, Kaat A. Systematic Review and Meta-analysis Comparing Topical Corticosteroids With Vehicle/Moisturizer in Childhood Atopic Dermatitis. *J Pediatr Nurs*. 2019;**47**:36-43.
15. Frangos J, Kimball A. Clobetasol propionate emollient formulation foam in the treatment of corticosteroid-responsive dermatoses. *Expert Opin Pharmacother*. 2008;**9**(11):2001-7.
16. Froeschl B, Arts D, Leopold C. Corticosteroid therapy in the treatment of pediatric patients with atopic dermatitis (Structured abstract). Health Technol Assess [Internet]. 2007; (4). Available from: http://onlinelibrary.wiley.com/o/cochrane/clhta/articles/HTA-32008100208/frame.html.
17. Gonzalez-Lopez G, Ceballos-Rodriguez R, Gonzalez-Lopez J, Feito Rodriguez M, Herranz-Pinto P. Efficacy and safety of wet wrap therapy for patients with atopic dermatitis: a systematic review and meta-analysis. *Br J Dermatol*. 2017;**177**(3):688-95.
18. Green C, Colquitt J, Kirby J, Davidson P, Payne E. Clinical and cost-effectiveness of once-daily versus more frequent use of same potency topical corticosteroids for atopic eczema: a systematic review and economic evaluation. *Health Technol Assess (Winchester, England)*. 2004;**8**(47):iii,iv, 1-120.
19. Gu S, Yang A, Li C, Lu C, Xue C. Topical application of Chinese herbal medicine for atopic eczema: A systematic review with a meta-analysis. *Dermatology*. 2014;**228**(4):294-302.
20. Gu S, Yang AW, Xue CC, Li CG, Pang C, Zhang W, et al. Chinese herbal medicine for atopic eczema. Cochrane Database Syst Rev [Internet]. 2013; (9). Available from: http://onlinelibrary.wiley.com/doi/10.1002/14651858.CD008642.pub2/abstract.
21. Hajar T, Leshem Y, Hanifin J, Nedorost S, Lio P, Paller A, et al. A systematic review of topical corticosteroid withdrawal ("steroid addiction") in patients with atopic dermatitis and other dermatoses. *J Am Acad Dermatol*. 2015;**72**(3):541-9.e2.
22. Hoare C, Li Wan Po A, Williams H. Systematic review of treatments for atopic eczema. *Health Technol Assess*. 2000;**4**(37):1-191.
23. Iskedjian M, Piwko C, Shear N, Langley R, Einarson T. Topical calcineurin inhibitors in the treatment of atopic dermatitis: A meta-analysis of current evidence. *Am J Clin Dermatol*. 2004;**5**(4):267-79.

For peer review only

24. Juhász ML, Curley RA, Rasmussen A, Malakouti M, Silverberg N, Jacob SE. Systematic Review of the Topical Steroid Addiction and Topical Steroid Withdrawal Phenomenon in Children Diagnosed With Atopic Dermatitis and Treated With Topical Corticosteroids. *J Dermatol Nurses Assoc*. 2017;**9**(5):233-40.
25. Łabędź N, Pawliczak R. Efficacy and safety of topical calcineurin inhibitors for the treatment of atopic dermatitis: meta-analysis of randomized clinical trials. *Postepy Dermatol Alergol*. 2019;**36**(6):752-9.
26. Legendre L, Barnetche T, Mazereeuw-Hautier J, Meyer N, Murrell D, Paul C. Risk of lymphoma in patients with atopic dermatitis and the role of topical treatment: A systematic review and meta-analysis. *J Am Acad Dermatol*. 2015;**72**(6):992-1002.
27. Li R, Zhu H, Fan L, Ni S, Feng C, Wu Z. Efficacy and tolerability of topical tacrolimus in the treatment of atopic dermatitis: A systematic review of randomized controlled trials. [Chinese]. *J Clin Dermatol*. 2007;**36**(12):757-60.
28. Nankervis H, Thomas K, Delamere F, Barbarot S, Rogers N, Williams H. Scoping systematic review of treatments for eczema2016 2016/05/None.
29. Penaloza Hidalgo B, Knight T, Burls A. A systematic review of effectiveness and cost effectiveness of tacrolimus ointment for topical treatment of atopic dermatitis in adults and children Health Technol Assess [Internet]. 2004; (4):[81 p.].
30. Schmitt J, Von Kobyletzki L, Svensson A, Apfelbacher C. Efficacy and tolerability of proactive treatment with topical corticosteroids and calcineurin inhibitors for atopic eczema: Systematic review and meta-analysis of randomized controlled trials. *Br J Dermatol*. 2011;**164**(2):415-28.
31. Sidbury R, Tom W, Bergman J, Cooper K, Silverman R, Berger T, et al. Guidelines of care for the management of atopic dermatitis: Section 4. Prevention of disease flares and use of adjunctive therapies and approaches. *J Am Acad Dermatol*. 2014;**71**(6):1218-33.
32. Siegfried E, Jaworski J, Kaiser J, Hebert A. Systematic review of published trials: Long-term safety of topical corticosteroids and topical calcineurin inhibitors in pediatric patients with atopic dermatitis. *BMC Pediatr*. 2016;**16** (75).
33. Singh S, Mann B. Clinical utility of clocortolone pivalate for the treatment of corticosteroid-responsive skin disorders: A systematic review. *Clin Cosmet Investig Dermatol*. 2012;**5**:61-8.
34. Svensson A, Chambers C, Gånemo A, Mitchell S. A systematic review of tacrolimus ointment compared with corticosteroids in the treatment of atopic dermatitis. *Curr Med Res Opin*. 2011;**27**(7):1395-406.
35. Tang T, Bieber T, Williams H. Are the concepts of induction of remission and treatment of subclinical inflammation in atopic dermatitis clinically useful? *J Allergy Clin Immunol*. 2014;**133**(6):1615-25.e1.
36. van Zuuren EJ, Fedorowicz Z, Christensen R, Lavrijsen AP, Arents BW. Emollients and moisturisers for eczema. Cochrane Database Syst Rev [Internet]. 2017; (2). Available from: http://onlinelibrary.wiley.com/doi/10.1002/14651858.CD012119.pub2/abstract.
37. Wood Heickman L, Davallow Ghajar L, Conaway M, Rogol A. Evaluation of Hypothalamic-Pituitary-Adrenal Axis Suppression following Cutaneous Use of Topical Corticosteroids in Children: A Meta-Analysis. *Horm Res Paediatr*. 2018;**89**(6):389-96.
38. Yan J, Chen S, Wang X, Zhou W, Wang F. Meta-analysis of tacrolimus ointment for atopic dermatitis in pediatric patients. *Pediatr Dermatol*. 2008;**25**(1):117-20.

For peer review only

Appendix 6 – Characteristics and safety data from the included studies

How safe are topical corticosteroids compared to emollient or vehicle?						
**Study ID** ***(Systematic review*)***	**Study design and study duration** ***(Quality assessment)***	**Intervention and comparator**	**Participants**	**Cutaneous adverse events**	**Systemic adverse events**	**Unspecified adverse events**
**Very potent topical corticosteroids**						
Breneman 2003 [(1)]<br>(unpublished)<br>***(Feldman 2005*** [(2)] ***Nankervis*** [(3)]***)***	RCT<br>2 weeks treatment, then followed up for additional 2 weeks<br>*Cochrane risk of bias tool: randomisation described, allocation concealment unclear, intention-to-treat unclear.*	**Intervention:** Clobetasol propionate 0.05% lotion (twice a day) **(n=96)**<br>**Intervention:** Clobetasol propionate 0.05% emollient cream (twice a day) (n=100)<br>**Comparator:** Vehicle (n=33)	**Severity:** moderate to severe<br>**Age:** ≥ 12 years<br>**Sample size:** 229 participants	**Local application site skin reactions**<br>No clinically significant telangiectasia or skin thinning		**Unspecified adverse events**<br>Incidence comparable between groups.<br>**Treatment-related adverse events**<br>**Clobetasol lotion** = 4/96 patients (4.2%); **Clobetasol cream** = 1/100 patients (1%)<br>**Vehicle** = 6/33 patients (18.2%)<br>*(Difference between groups: p= 0.0006[a])*
Kimball 2008 [(4)] (trial a)<br>***(Frangos 2008*** [(5)]***)***	RCT<br>Duration not specified in review<br>*Risk of bias not assessed in any of the included systematic reviews.*	**Intervention:** Clobetasol propionate emulsion formulation foam 0.05%<br>**Comparator:** Vehicle	**Severity:** not specified in the review<br>**Age:** not specified in the review<br>**Sample size:** not specified in the review			**Incidence of adverse events or treatment related adverse events**<br>**Clobetasol foam** = 8%<br>**Vehicle foam** = 10%<br>*(no significant differences between groups)*
Rosso 2009 [(6)]<br>***(Barnes 2009*** [(7)]***)***	RCT<br>2 weeks treatment<br>*Risk of bias not assessed in any of the included systematic reviews.*	**Intervention:** Fluocinonide 0.1% cream (n=109)<br>**Comparator:** Vehicle (n=50)	**Severity:** not specified in the review<br>**Age:** not specified in the review<br>**Sample size:** 159 participants	**Skin thinning**<br>**Fluocinonide:** 6/109 participants (5.6%)<br>**Vehicle:** 2/50 participants (4.3%)<br>*(Difference between groups: p=0.69[a])*		
www.olux-e.com (online data) [(8)]<br>***(Frangos 2008*** [(5)]***)***	Single arm study (observational)<br>2 weeks treatment	**Intervention:** Clobetasol propionate emollient foam (twice daily) (n=37)<br>**Comparator:** No comparator	**Severity:** ≥30% BSA<br>**Age:** ≥12 years old<br>**Sample size:** 37 participants		**HPA axis suppression**<br>6/37 patients (16%)<br>*(not specified in the review how it was measured)*	

For peer review only

	Risk of bias not assessed in any of the included systematic reviews.					
Kimball 2008 [(4)] (trial b)<br>***(Frangos 2008*** [(5)]***; Wood Heickman 2018*** [(9)]***)***	Open label Phase II safety study<br>2 weeks treatment<br>*Risk of bias not assessed in any of the included systematic reviews.*	**Intervention:** Clobetasol propionate emollient foam 0.05% (twice daily) (n=52)<br>**Comparator:** No comparator	**Severity:** mild to severe<br>**Age:** children (from 6 years old) and adults<br>**Sample size:** 52 participants		**<u>HPA axis suppression</u>**<br>7/30 (23.3%) had adrenal insufficiency (ACTH stimulation testing, measuring serum cortisol levels).<br>• 47% of children (aged 6-11)<br>• 0% of adolescents (aged 12-17)<br>• 27% of adults (≥18 years)<br>Was reported as transient and reversible. After TCS discontinuation, children with biochemical adrenal insufficiency had complete resolution at retesting.	
Herz 1991 [(10)]<br>***(Barnes 2015*** [(7)]***)***	Single arm study (observational)<br>*(2 weeks treatment)*<br>*Risk of bias not assessed in any of the included systematic reviews.*	**Intervention:** Clobetasol propionate (n=59)<br>**Comparator:** No comparator	**Severity:** not specified in the review<br>**Age:** not specified in the review<br>**Sample size:** 59 participants	**<u>Skin thinning</u>** 1 case of skin thinning reported (not clear if in a psoriasis or eczema patient – but assume its eczema as this is the topic of the systematic review).		
**Potent topical corticosteroids**						
Sugarman 2009 [(11)]<br>***(Van Zuuren 2017*** [(12)]***)***	RCT<br>*(4 weeks treatment)*<br>*(Cochrane risk of bias tool: low risk of selection, attrition and other biases. Unclear risk of reporting and performance bias, High risk of detection bias.* [(12)]*)*<br>*(Cochrane risk of bias tool: unclear risk of selection bias, high risk from no blinding.* [(3)]*)*	**Intervention:** Fluticasone 0.05% cream twice daily (hydrocortisone 2.5% for the face and body folds) (n=62)<br>**Comparator:** Ceramide-dominant barrier repair formulation (EpiCeram) twice daily (emollient) (n=59)	**Severity:** moderate to severe<br>**Age:** children 6 months to 18 years (mean age 7.1 years)<br>**Sample size:** 121 participants			**<u>Serious adverse events</u>**<br>The participants did not report any in either group.<br>No further details regarding other possible treatment related adverse events were reported.

Griffiths 2002 [13]<br>***(Nankervis 2017*** [3]***)***	RCT<br>*(up to 14 days treatment)*<br>*(Cochrane risk of bias tool: low risk of selection bias from sequence generation, unclear risk of selection bias from allocation concealment, low risk from blinding.* [3]*)*	**Intervention:** Hydrocortisone 17-butyrate cream (0.1%) maximum application of 2g (four fingertip units) per day (n=49)<br>**Comparator:** Cipamfylline cream (1.5 mg of cipamfylline per gram of cream) used up to a maximum of 2 g (four fingertip units) of cream per day (emollient) (n=54)	**Severity:** not specified<br>**Age:** adults ≥18 years old<br>**Sample size:** 103 participants	No difference in cutaneous adverse events which were possibly or probably related to treatment in either group (p = 0.13)<br>The adverse events were mostly application site reactions, including itching, stinging or burning, and drug reactions.		**Unspecified adverse events**<br>**Hydrocortisone group:** 20/49 (40.8%) participants reported 41 adverse events in total.<br>**Emollient:** 29/52 (55.8%) participants reported 63 adverse events in total.<br>*(Difference between groups: p= 0.14* [a]*)*
Eichenfield 2006 [14]<br>***(Nankervis 2017*** [3]***)***	RCT<br>*(4 weeks treatment)*<br>*Risk of bias not assessed*	**Intervention:** Fluticasone propionate four times daily (n=221)<br>**Comparator:** Vehicle four times daily (n=217)	**Severity:** moderate to severe<br>**Age:** children from 3 months old to 16 years old<br>**Sample size:** 438 children			**Withdrawal due to adverse events**<br>**Topical corticosteroids:** 4 participants in total from this study and from Hebert 2007<br>**The number of participants reporting at least 1 adverse event**<br>**Fluticasone:** 77/221 (34.8%) participants<br>**Vehicle:** 82/217 (37.8%) participants<br>*(Difference between groups: p=0.52* [a]*)*
Wu 2013 [15]<br>***(Nankervis 2017*** [3]***, Fishbein 2019*** [16]***)***	RCT<br>*(10 days treatment)*<br>*(Cochrane risk of bias tool: low risk of selection bias from sequence generation. Unclear risk of selection bias from allocation concealment, unclear risk from blinding and other biases: Two out of 60 participants were excluded from the analyses as they used concomitant medication* [3]*)*	**Intervention:** Mometasone furoate 0.1% cream, twice a day (n=20)<br>**Comparator:** placebo of distilled water in 1% dimethyl sulfoxide mixed with the identical cream base as used for the 15(R/S)-methyl-lipoxin A4 (n=20)<br>**Comparator:** 15(R/S)-methyl-lipoxin A4 0.1% cream (n=20)	**Severity:** all severities<br>**Age:** children from 1 month to 1 year old<br>**Sample size:** 60 participants		None of the safety tests (e.g. full blood count, kidney and liver function test, and electrocardiogram) showed any significant differences compared with baseline for all three treatment groups.	No clinical adverse events were reported.

Pellanda 2005 [17]<br><br>***(Nankervis 2017 [3])***	RCT<br><br>*(Duration not specified in the review)*<br><br>*Risk of bias not assessed*	**Intervention:** Triamcinolone acetonide<br><br>**Comparator:** Vehicle	**Severity:** mild to moderate<br><br>**Age:** not specified in the review<br><br>**Sample size:** not specified in the review	**Skin changes**<br>One report by a participant using placebo (no further details)		
Lebwohl 1996 [18]<br><br>***(Hoare 2000 [19])***	RCT<br><br>*(29 days treatment)*<br><br>*(Moher 1995 quality checklist: method and concealment of randomisation unclear, double blinded, large number of withdrawals and dropouts, no ITT analysis [19])*	**Intervention:** Fluticasone propionate ointment 0.005%<br><br>**Comparator:** Vehicle	**Severity:** not specified in the review<br><br>**Age:** not specified in the review<br><br>**Sample size:** 203 participants			The review authors only reported that *"Drug related adverse effects were rare"*
Lebwohl 1999 [20]<br><br>***(Hoare 2000 [19])***	RCT<br><br>*(29 days treatment)*<br><br>*(Moher 1995 quality checklist: method and concealment of randomisation unclear, double blinded, large number of withdrawals and dropouts, no ITT analysis [19])*	**Intervention:** Fluticasone propionate ointment 0.005%<br><br>**Comparator:** Vehicle	**Severity:** not specified in the review<br><br>**Age:** not specified in the review<br><br>**Sample size:** 169 participants			The review authors only reported that *"Drug related adverse effects were rare"*
Abramovitis 2010 [21]<br><br>***(Wood Heickman 2018 [9], Fishbein 2019 [16])***	RCT<br><br>(21 to 29 days treatment)<br><br>*Risk of bias not assessed in any of the included systematic reviews.*	**Intervention:** Hydrocortisone butyrate 0.1% cream, twice daily (n=131)<br><br>**Comparator:** Lipocream vehicle, twice daily (n=133)	**Severity:** Mild to moderate<br><br>**Age:** children 3 months to 18 years (mean 7.2 years)<br><br>**Sample size**: 264 children		**HPA axis suppression (no data for vehicle group)**<br>5/63 (7.9%) children in the hydrocortisone group (measured using ACTH stimulation testing, measuring serum cortisol levels)<br><br>After TCS discontinuation, children with biochemical adrenal insufficiency had complete resolution at retesting.	**The number of participants reporting at least 1 adverse event**<br>**Hydrocortisone:** 29/131 (22.1%) participants<br>**Vehicle:** 28/133 (21.1%) participants<br>*(Difference between groups: p=0.83 [a])*
Matheson 2008 [22]	RCT<br><br>(28 days treatment)	**Intervention:** Hydrocortisone butyrate 0.1% lotion, twice daily (n=139)	**Severity:** Mild to moderate			**The number of participants reporting at least 1 adverse event**<br>**Hydrocortisone:** 48/139 (34.5%)

For peer review only - http://bmjopen.bmj.com/site/about/guidelines.xhtml

(Fishbein 2019 ***[16])***	*Risk of bias not assessed in any of the included systematic reviews.*	**Comparator:** Vehicle, twice daily (n=145)	**Age:** children 3 months to 18 years<br>**Sample size**: 284 children			participants<br>**Vehicle:** 56/145 (38.6%) participants<br>*(Difference between groups: p=0.48* [a]*)*
Friedlander 2002 [23]<br>***(Callen 2007*** ***[24]; Wood Heickman 2018*** ***[9])***	Single arm study (observational)<br>*(3 to 4 weeks)*<br>*Risk of bias not assessed in any of the included systematic reviews.*	**Intervention:** Fluticasone propionate cream 0.05% (n=43)<br>**Comparator:** No comparator	**Severity:** not specified in the review<br>**Age:** children 3 months to 6 years<br>**Sample size:** 43 participants		**HPA axis suppression**<br>2/43 (4.7%) children (measured using ACTH stimulation testing, measuring serum cortisol levels)<br>After TCS discontinuation, children with biochemical adrenal insufficiency had complete resolution at retesting.	
Eichenfield 2007 [25]<br>***(Wood Heickman 2018*** ***[9])***	Single arm study (observational)<br>(4 weeks treatment)<br>*Risk of bias not assessed in any of the included systematic reviews.*	**Intervention:** Hydrocortisone butyrate 0.1% (n=20)<br>**Comparator:** No comparator	**Severity:** not specified in the review<br>**Age:** children (median or mean = 9 years)<br>**Sample size**: 20 children		**HPA axis suppression**<br>0/20 (0%) children (measured using ACTH stimulation testing, measuring serum cortisol levels)	
Hebert 2006 [26]<br>***(Wood Heickman 2018*** ***[9])***	Single arm study (observational)<br>(3 to 4 weeks treatment)<br>*Risk of bias not assessed in any of the included systematic reviews.*	**Intervention:** Fluticasone propionate 0.05% lotion (n=42)<br>**Comparator:** No comparator	**Severity:** not specified in the review<br>**Age:** children (median or mean 2.6 years)<br>**Sample size**: 42 children		**HPA axis suppression**<br>0/42 (0%) children (measured using ACTH stimulation testing, measuring serum cortisol levels)	
**Moderate potency topical corticosteroids**						
De Belilovsky 2011 [27]<br>***(Van Zuuren 2017*** ***[12])***	RCT<br>*(3 weeks treatment)*<br>*(Cochrane risk of bias tool: low risk of selection, attrition, reporting and other biases. Unclear risk of performance bias.*	**Intervention:** Hydrocortisone butyric propionate 0.1% twice daily (n=40)<br>**Comparator:** Stelatopia (2% sunflower oil, fatty acids, ceramides) twice daily (n=40)	**Severity:** mild to moderate<br>**Age:** children 4 months to 4 years (mean age 2.3 years)<br>**Sample size:** 80 participants			No participants reported adverse events

For peer review only

	High risk of detection bias. [(12)]*)*<br>*(Cochrane risk of bias tool: unclear risk of selection bias and risk from blinding.* [(3)]*)*					
Rosenthal 1980 [(28)]<br>***(Singh 2012*** [(29)]***)***	RCT<br>*(14 days treatment)*<br>*(Delphi list: method of randomisation not described, allocation not concealed, blinded, no ITT analysis* [(29)]*)*	**Intervention:** Clocortolone pivalate 0.1% cream (applied thrice daily)<br>**Comparator:** Vehicle (applied thrice daily)	**Severity:** not specified in the review<br>**Age:** not specified in the review<br>**Sample size:** 100 participants			No adverse events
Binder 1977 [(30)]<br>***(Singh 2012*** [(29)]***)***	RCT<br>*(14 days treatment)*<br>*(Delphi list: method of randomisation not described, allocation not concealed, blinded, no ITT analysis* [(29)]*)*	**Intervention:** Clocortolone pivalate (applied thrice daily) (n=17)<br>**Comparator:** Vehicle (n=12)	**Severity:** not specified in the review<br>**Age:** mean age 30 years<br>**Sample size:** 29 participants	**<u>Irritation and dryness</u>**<br>Clinically significant in one patient in each group – did not result in discontinuation.		
Rauschkol 1981 [(31)]<br>***(Fishbein 2019*** [(16)]***)***	Within-participant RCT<br>*(14 days treatment)*<br>*Risk of bias not assessed in any of the included systematic reviews.*	**Intervention:** Halcinonide 0.025% cream, twice daily, on one arm<br>**Comparator:** Placebo cream unspecified, twice daily on the other arm at the same time	**Severity:** not reported<br>**Age:** children 7 months to 15 years (mean age 8 years)<br>**Sample size:** 86 children			**<u>The number of participants reporting at least 1 adverse event</u>**<br>**Halcinonide:** 4/86 (4.7%) participants<br>**Placebo:** 5/86 (5.8%) participants
Nolting 1991 [(32)]<br>***(De Tiedra 1997*** [(33)]***)***	RCT (but safety data only presented for one arm)<br>*(21 days treatment)*<br>*Risk of bias not assessed in any of the included systematic reviews.*	**Intervention:** Prednicarbate cream 0.25% (2 applications per day) (n=34)<br>**Comparator:** mometasone cream 0.1% twice daily (no safety data given)	**Severity:** Disease duration = mean 4.1 years ± 2.7<br>**Age:** children 2-12 years (mean 6.6 ± 3.6).<br>**Sample size:** 34 participants (with safety data)			**<u>Adverse reactions</u>**<br>2/34 patients (5.9%)
Rampini 1992 [(34)]	RCT (but safety data only presented for one arm)	Intervention: Prednicarbate cream/unguent 0.25% (2 applications per day) (n=93)	**Severity:** not specified in the review			**<u>Adverse reactions</u>**<br>3/93 patients (3.2%)

For peer review only

For peer review only - http://bmjopen.bmj.com/site/about/guidelines.xhtml

(De Tiedra 1997 ***[33])***	*(21 days treatment)*<br>*(Moher 1995 quality checklist: method and concealment of randomisation unclear, double blinded, two dropouts, no ITT analysis,* *[19])*	Comparator:methylprednisolone aceponate 0.1% once daily (no safety data given)	**Age:** children 0.3 to 14 years (mean 6.6).<br>**Sample size:** 93 participants (with safety data)			
Camacho 1996 [35]<br>***(De Tiedra 1997*** ***[33])***	RCT (but safety data only presented for one arm)<br>*(21 days treatment)*<br>*(Moher 1995 quality checklist: method and concealment of randomisation unclear, double blinded, no ITT analysis, 14/49 dropouts,* *[19])*	**Intervention:** Prednicarbate cream 0.25% (2 applications per day) (n=49)<br>**Comparator:** fluocortolone pivalate cream 0.2% (no safety data given)	**Severity:** Disease duration= mean 6.2 years ± 8.2 (range 0.25 to 39 years).<br>**Age:** adults 19 to 65 years (mean 34.1 ± 12).<br>**Sample size:** 49 participants (with safety data)			**Adverse reactions**<br>4/49 patients (8.1%)
Gimenez Camarasa 1994 [36]<br>***(De Tiedra 1997*** ***[33])***	RCT (but safety data only presented for one arm)<br>*(21 days treatment)*<br>*Risk of bias not assessed in any of the included systematic reviews.*	**Intervention:** Prednicarbate cream 0.25% (2 applications per day) (n=41)<br>**Comparator**: fluocinolone cream 0.025% twice daily (no safety data given)	**Severity:** Disease duration = mean 6.4 years ± 8.6 (range 0-40).<br>**Age:** adults 18 to 77 years (mean 37.6 ± 15.9).<br>**Sample size:** 41 participants (with safety data)			**Adverse reactions**<br>0/41 patients (0%)
Moshang 2001 [37]<br>***(Callen 2007*** ***[24]; Wood Heickman 2018*** ***[9])***	Single arm study (observational)<br>*(3 weeks treatment)*<br>*Risk of bias not assessed in any of the included systematic reviews.*	**Intervention:** Prednicarbate emollient cream 0.1%, twice daily (n=55)<br>**Comparator:** No comparator	**Severity:** not specified in the review<br>**Age:** children 4 months to 12 years<br>**Sample size:** 55 participants		**HPA axis suppression**<br>All normal (measured using ACTH stimulation testing, measuring serum cortisol levels)	
Conde 2008 [38]<br>***(Singh 2012*** ***[29])***	Single arm study (observational)<br>*(4 weeks treatment)*	**Intervention:** Clocortolone pivalate cream 0.1% twice daily (n=10)<br>**Comparator:** No comparator	**Severity:** mild to moderate<br>**Age:** children, mean age 7.9 years			No adverse events reported

	Risk of bias not assessed in any of the included systematic reviews.		**Sample size:** 10 participants			
Crespi 1986 (39)<br><br>***(Callen 2007 (24))***	Single arm study (observational)<br><br>*(4 weeks treatment)*<br><br>*Risk of bias not assessed in any of the included systematic reviews.*	**Intervention:** Alclometasone cream, twice daily (n=39)<br><br>**Comparator:** No comparator	**Severity:** not specified in the review<br><br>**Age:** children<br><br>**Sample size:** 39 participants		**HPA axis suppression**<br>All normal (measured via morning cortisol)	
**Mild potency topical corticosteroids**						
Udompataikul 2011 (40)<br><br>***(Van Zuuren 2017 (12))***	Within-participant RCT<br><br>*(6 weeks treatment)*<br><br>*(Cochrane risk of bias tool: unclear risk of selection, performance and attrition bias. Low risk of reporting and other biases. High risk of detection bias. (12))*<br><br>*(Cochrane risk of bias tool: unclear risk of selection bias from sequence generation and risk from blinding. Low risk of selection bias from allocation concealment, (3))*	**Intervention:** Hydrocortisone acetate 1% cream twice daily, was applied one side of the body for 4 weeks followed by the cream base for 2 weeks.<br>**Comparator:** Licochalcone (containing *Glycyrrhiza inflata* root extract, decanediol, menthoxypropanediol and 6-fatty acids) applied twice daily on one side of the body for 6 weeks	**Severity:** mild to moderate<br><br>**Age:** children 2 months to 10 years (mean age 5.8 years)<br><br>**Sample size:** 30 participants			No adverse events on either side during the study.
Hebert 2007 (41)<br><br>***(Nankervis 2017 (3), Fishbein 2019 (16))***	RCT<br><br>*(28 days)*<br><br>*Risk of bias not assessed in any of the included systematic reviews.*	**Intervention:** Desonide 0.05% gel twice daily (n=425)<br><br>**Comparator:** Hydrogel vehicle twice daily (n=157)	**Severity:** mild to moderate<br><br>**Age:** children 3 months to 18 years<br><br>**Sample size:** 582 children			**Serious adverse events**<br>One event reported in TCS group but not thought to be related to treatment<br><br>**Withdrawal due to adverse events**<br>TCS group: 4 in total from this study and from Eichenfield 2006<br><br>**The number of participants reporting at least 1 adverse event**<br>**Desonide:** 85/425 (20 %) participants<br>**Vehicle:** 46/157 (29.3%)

For peer review only

For peer review only - http://bmjopen.bmj.com/site/about/guidelines.xhtml

						participants<br>*(Difference between groups: p=0.02 [a])*
Udompataikul 2012 [42]<br><br>***(Fishbein 2019 [16])***	Within-participant RCT<br><br>*(4 weeks treatment)*<br><br>*Risk of bias not assessed in any of the included systematic reviews.*	**Intervention:** Hydrocortisone 1% ointment twice daily, applied to one arm.<br>**Comparator:** 5% dexapanthenol ointment twice daily, applied to the other arm at the same time.	**Severity:** mild to moderate<br><br>**Age:** children 2 years to 15 years (mean age 7.2 years)<br><br>**Sample size:** 30 participants			No adverse events on either side during the study.
Wananukul 2013 [43]<br><br>***(Van Zuuren 2017 [12])***	Within-participant RCT<br><br>*(4 weeks treatment)*<br><br>*(Cochrane risk of bias tool: low risk of selection bias from sequence generation, performance, detection, attrition, reporting and other biases. Unclear risk of selection bias from allocation concealment. [12])*	**Intervention:** Hydrocortisone acetate 1% cream twice daily on one side of the body<br><br>**Comparator:** Licochalcone (containing *Glycyrrhiza inflata* root extract, decanediol, menthoxypropanediol and 6-fatty acids) twice daily on one side of the body	**Severity:** mild to moderate<br><br>**Age:** children, mean age 3.1 years<br><br>**Sample size:** 55 participants			No adverse events on either side during the study
Jirabundansuk 2014 [44]<br><br>***(Van Zuuren 2017 [12])***	Within-participant RCT<br><br>*(4 weeks treatment)*<br><br>*(Cochrane risk of bias tool: Unclear risk of selection and performance bias. High risk of detection bias. Low risk of attrition, reporting and other biases. [12])*	**Intervention:** Hydrocortisone acetate 1% cream twice daily on one side of the body<br><br>**Comparator:** Moisturiser containing spent grain, Vitellaria paradoxa (formerly Butyrospermum parkii) extract plus Argania spinosa kernel oil twice daily on one side of the body	**Severity:** Mild or moderate<br><br>**Age:** children 2-15 years (mean age 4.3 years)<br><br>**Sample size:** 31 participants			The investigators stated that "no specific adverse events were reported".
Dolle 2010 [45]<br><br>***(Nankervis 2017 [3])***	Within-participant RCT<br><br>*(3 weeks treatment)*<br><br>*(Cochrane risk of bias tool: unclear risk of selection bias and risk from blinding. [3])*	**Intervention:** 1% hydrocortisone solution once daily for 1st week then twice daily up to 3 weeks<br><br>**Comparator:** 6% miltefosine solution once daily for 1st week then twice daily up to 3 weeks	**Severity:** moderate to severe<br><br>**Age:** adults (≥18 years old)<br><br>**Sample size:** 16 participants	<u>**Local topical adverse events related to the treatment**</u><br>**Hydrocortisone:** 7/16 participants (44%)<br>**Emollient:** 10/16 participants (63%)<br>These adverse events included pruritus, burning, tingling and dry	No systemic adverse events	No withdrawals because of adverse events

For peer review only

				skin. Dry skin was seen only with emollient treatment.		
Patzelt-Wenczler 2000 [46]<br><br>***(Nankervis 2017 [3])***	Within-participant RCT<br><br>*(2 weeks treatment)*<br><br>*(Cochrane risk of bias tool: unclear risk of selection bias and high risk from no blinding. [3])*	**Intervention:** Hydrocortisone 0.5% twice daily<br><br>**Comparator:** Kamillosan® cream, containing 2% ethanolic extract of chamomile flowers, twice daily (emollient)<br><br>Comparator: Vehicle cream applied twice daily	**Severity:** at least moderate<br><br>**Age:** not specified in the review<br><br>**Sample size:** 72 participants			Three participants in the emollient group withdrew early because of intolerability.
Paller 2003 [47]<br><br>***(Nankervis 2017 [3])***	RCT<br><br>*(2 weeks treatment)*<br><br>*Risk of bias not assessed in any of the included systematic reviews.*	**Intervention:** Fluocinolone acetonide 0.01% twice daily (n=45)<br><br>**Comparator:** Vehicle twice daily (n=49)	**Severity:** not specified in the review<br><br>**Age:** children from 2 to 12 years old<br><br>**Sample size:** 94 participants	**Mild hypopigmentation**<br>Two participants out of 45 reported this event with fluocinolone (4.4%)		
Patel 1995 [48]<br><br>***(Callen 2007 [24])***	Single arm study (observational)<br><br>*(3-10 years follow up)*<br><br>*Risk of bias not assessed in any of the included systematic reviews.*	**Intervention:** 1% Hydrocortisone ointment (n=14; 9/14 intermittently used moderate to high potency)<br><br>**Comparator:** No comparator	**Severity:** not specified in the review<br><br>**Age:** children 3.1 to 10.7 years<br><br>**Sample size:** 14 participants		**HPA axis suppression**<br>Plasma cortisol levels - no change in basal/peak levels but peaked earlier	
Dohil 2009 [49]<br><br>(Wood Heickman 2018 [9])	Single arm study (observational)<br><br>(4 weeks duration)<br><br>*Risk of bias not assessed in any of the included systematic reviews.*	**Intervention:** fluocinolone acetonide 0.01%<br><br>**Comparator:** No comparator	**Severity:** not specified in the review<br><br>**Age:** children (median or mean age 1.1 years)<br><br>**Sample size:** 24 participants		**HPA axis suppression**<br>No cases of adrenal insufficiency *(measured using ACTH stimulation testing, measuring serum cortisol levels)*	
Eichenfield 2007 [50]<br><br>(Wood Heickman 2018 [9])	Single arm study (observational)<br><br>(4 weeks duration)<br><br>*Risk of bias not assessed in any of the included systematic reviews.*	**Intervention:** Desonide hydrogel 0.05%<br><br>**Comparator:** No comparator	**Severity:** not specified in the review<br><br>**Age:** children (median or mean age 3.3 years)		**HPA axis suppression**<br>No cases of adrenal insufficiency *(measured using ACTH stimulation testing, measuring serum cortisol levels)*	

For peer review only

			**Sample size:** 34 participants			
Hebert 2008 [(51)]<br><br>(Wood Heickman 2018 [(9)])	Single arm study (observational)<br><br>(4 weeks duration)<br><br>*Risk of bias not assessed in any of the included systematic reviews.*	**Intervention:** Desonide 0.05% foam<br><br>**Comparator:** No comparator	**Severity:** not specified in the review<br><br>**Age:** children (median or mean age 6.7 years)<br><br>**Sample size:** 75 participants		**<u>HPA axis suppression</u>**<br>Three out of 75 participants had adrenal insufficiency<br>*(measured using ACTH stimulation testing, measuring serum cortisol levels)*	
**How safe are topical corticosteroids compared to topical calcineurin inhibitors?**						
**Study ID *(Systematic review*)***	**Study design and study duration *(Quality assessment)***	**Intervention and comparator**	**Participants**	**Cutaneous adverse events**	**Systemic adverse events**	**Unspecified adverse events**
**Potent topical corticosteroids**						
Bieber 2007 [(52)]<br><br>***(Broeders 2016 [(53)])***	RCT<br><br>*(up to 3 weeks treatment)*<br><br>*(Jadad score 4/5 – risk from sequence generation and allocation concealment [(53)])*<br><br>*(Cochrane risk of bias tool: unclear risk of selection bias and from blinding. [(3)])*<br><br>*(Cochrane risk of bias tool: unclear risk of selection bias. Low risk of performance, detection, attrition, reporting and other biases. [(54)])*	**Intervention:** Methyl-prednisolone 0.1% (n=129) once daily in the evening to all affected body surface areas for a minimum of 2 weeks and a maximum of 3 weeks and cleared areas treated for an additional 7 days post clearance. Also applied a vehicle ointment in the morning to maintain blinding.<br><br>**Comparator:** Tacrolimus 0.03% (n=136), applied twice daily, morning and evening, to all affected body surface areas for a minimum of 2 weeks and a maximum of 3 weeks and cleared areas treated for an additional 7 days post clearance.	**Severity:** severe to very severe<br><br>**Age:** children 2 to 15 years old<br><br>**Sample size:** 265 participants	**<u>Adverse events related to treatment</u>**<br>**Methyl-prednisolone:** 0/129 participants (0%)<br>**Tacrolimus:** 6/136 participants (4.4%)<br>*(Difference between groups: p=0.09 [a, b])*		**<u>Severe adverse events</u>**<br>**Methyl-prednisolone:** 0/129 participants (0%)<br>**Tacrolimus:** 6/136 participants (4.4%)<br>*(Difference between groups: p=0.09 [a, c])*<br><br>**<u>Adverse events requiring discontinuation</u>**<br>**Methyl-prednisolone:** 0/129 (0%)<br>**Tacrolimus:** 4/136 (3%)<br>*(Difference between groups: p=0.15 [a])*
Doss 2010 [(55)]<br><br>***(Broeders 2016 [(53)])***	RCT<br><br>*(3 weeks treatment twice daily, plus 3 weeks follow up with once daily treatment)*	**Intervention:** Fluticasone 0.005% ointment applied twice daily to all affected areas except eyelids until clearance, up to 3 weeks. All participants who responded to treatment could apply treatment once a day to	**Severity:** moderate to severe<br><br>**Age:** children 2 to 15 years old	**<u>Adverse events related to treatment</u>**<br>**Fluticasone:** 45/239 participants (19%)<br>**Tacrolimus:** 55/239 participants (23%)		**<u>Severe adverse events</u>**<br>**Fluticasone:** 2/239 participants (0.8%)<br>**Tacrolimus:** 1/239 participants (0.4%)<br>*(Difference between groups: p=0.57 [a])*

For peer review only

	(Jadad score 5/5 – risk from allocation concealment [53])<br><br>*(Cochrane risk of bias tool: unclear risk of selection bias and from blinding. [3])*<br><br>*(Cochrane risk of bias tool: Low risk of selection, performance, detection, attrition, reporting and other biases. [54])*<br><br>*Cochrane risk of bias tool: low risk of selection, performance, attrition, reporting and other biases. Unclear risk of performance bias [56]).*	the remaining lesions for another 3 weeks (n=239)<br><br>**Comparator:** Tacrolimus 0.03% ointment applied twice daily to all affected areas except eyelids until clearance, up to 3 weeks. All participants who responded to treatment could apply treatment once a day to the remaining lesions for another 3 weeks (n=239)	**Sample size:** 478 participants	*(Difference between groups: p=0.26 [a])*<br><br>**Skin burning**<br>**Fluticasone:** 6/239 (2.5%)<br>**Tacrolimus:** 18/237 (7.6%)<br>*(Difference between groups: p=0.02 [a])*<br><br>**Pruritus**<br>**Fluticasone:** 8/239 participants (3.3%)<br>**Tacrolimus:** 10/237 participants (4.2%)<br>*(Difference between groups: p=0.62 [a])*<br><br>**Skin infection**<br><br>**Fluticasone:** 49/239 participants (21%)<br>**Tacrolimus:** 44/239 participants (18%)<br>*(Difference between groups: p=0.56 [a])*		**Adverse events requiring discontinuation**<br>**Fluticasone:** 6/239 participants (2.5%)<br>**Tacrolimus:** 4/239 participants (1.7%)<br>*(Difference between groups: p=0.53 [a])*
Doss 2009 [57]<br><br>***(Broeders 2016 [53])***	RCT<br><br>*(3 weeks of treatment – then for a further 3 weeks either stop treatment, once daily treatment or switch to other treatment twice daily)*<br><br>*(Jadad score 5/5 – risk from allocation concealment [53])*<br><br>*(Cochrane risk of bias tool: unclear risk of selection bias and low risk from blinding. [3])*	**Intervention:** Fluticasone 0.005% ointment twice daily on facial eczema lesions for 3 weeks or until clearance (n=279)<br><br>**Comparator:** Tacrolimus 0.1% twice daily on facial eczema lesions for 3 weeks or until clearance (n=287)<br><br>For 21 days after the initial 3 weeks, the participants could stop treatments if the facial lesions had cleared; stay on the same treatment once a day; or swap treatment using it twice daily (still blinded)	**Severity:** moderate to severe<br><br>**Age:** adults<br><br>**Sample size:** 566 participants	**Adverse events related to treatment**<br>**Fluticasone:** 42/279 participants (15%)<br>**Tacrolimus:** 75/287 participants (26%)<br>*(Difference between groups: p=0.001 [a])*<br><br>**Skin burning**<br>**Fluticasone:** 9/279 participants (3.2%)<br>**Tacrolimus:** 47/287 participants (16.4%)<br>*(Difference between groups: p<0.00001 [a])*<br><br>**Pruritus**<br>**Fluticasone:** 9/279 participants (3.2%)<br>**Tacrolimus:** 12/287 participants (4.2%)<br>*(Difference between groups: p=0.55 [a])*		**Severe adverse events**<br>**Fluticasone:** 0/279 participants (0%)<br>**Tacrolimus:** 1/287 participants (0.3%)<br>*(Difference between groups: p=0.51 [a]).*<br><br>**Adverse events requiring discontinuation**<br>**Fluticasone:** 8/279 participants (2.9%)<br>**Tacrolimus:** 7/287 participants (2.4%)<br>*(Difference between groups: p=0.75 [a])*

For peer review only

For peer review only - http://bmjopen.bmj.com/site/about/guidelines.xhtml

Luger 2001 [58]<br><br>***(Broeders 2016 [53])***	RCT<br><br>*(up to 3 weeks treatment)*<br><br>*(Jadad score 3/5 – risk from sequence generation and allocation concealment [53])*<br><br>*(Cochrane risk of bias tool: unclear risk of selection bias and unclear risk from blinding. [3])*<br><br>*(Jadad scale: 3/5 [59])*<br><br>*(Cochrane risk of bias tool: unclear risk of selection bias, adequate blinding, inadequate loss to follow up. [60])*	**Intervention:** Betamethasone valerate 0.1% applied twice daily on all affected areas except for the head and neck for up to 3 weeks or until complete clearance if this was sooner (n=42)<br><br>**Comparator:** Pimecrolimus 1% applied twice daily on all affected areas except for the head and neck for up to 3 weeks or until complete clearance if this was sooner (n=45)	**Severity:** moderate<br><br>**Age:** adults ≥ 18 years old<br><br>**Sample size:** 87 participants	**<u>Pruritus</u>**<br>**Betamethasone:** 5/42 participants (12%)<br>**Pimecrolimus:** 14/45 participants (31%)<br>*(Difference between groups: p=0.04 [a])*<br><br>**<u>Skin burning</u>**<br>**Betamethasone:** 4/42 participants (9.5%)<br>**Pimecrolimus:** 22/45 participants (49%)<br>*(Difference between groups: p=0.001 [a])*		**<u>Adverse events requiring discontinuation</u>**<br>**Betamethasone:** 1/42 participants (2.4%)<br>**Pimecrolimus:** 3/45 participants (6.7%)<br>*(Difference between groups: p=0.36 [a])*
Luger 2004 [61]<br><br>***(Broeders 2016 [53])***	*RCT*<br><br>*(52 weeks. Twice daily until clearance, restarted with flares)*<br><br>*(Jadad score 3/5 – risk from sequence generation and allocation concealment [53])*<br><br>*(Cochrane risk of bias tool: unclear risk of selection bias, low risk from blinding. [3])*<br><br>*(Jadad scale: 3/5 [59])*<br><br>*(Cochrane risk of bias tool: adequate allocation generation, unclear allocation concealment, adequate blinding, inadequate loss to follow up. [60])*	**Intervention:** Triamcinolone 0.1% (potent) and Hydrocortisone acetate 1% (face) (Mild potency) twice daily until complete clearance and itching had stopped, then treatment restarted if inflammation recurred (n=330)<br><br>**Comparator:** Pimecrolimus 1% twice daily until complete clearance and itching had stopped, then treatment restarted if inflammation recurred (n=328)	**Severity:** moderate to severe<br><br>**Age:** adults (age 18 to 79 years)<br><br>**Sample size:** 658 participants	**<u>Skin burning</u>**<br>**Triamcinolone + hydrocortisone:** 36/330 participants (11%)<br>**Pimecrolimus:** 85/328 participants (26%)<br>*(Difference between groups: p<0.00001 [a])*<br><br>**<u>Pruritus</u>**<br>**Triamcinolone + hydrocortisone:** 6/330 participants (1.8%)<br>**Pimecrolimus:** 18/328 participants (5.5%)<br>*(Difference between groups: p=0.02 [a])*<br><br>**<u>Skin thinning</u>**<br>**Triamcinolone + hydrocortisone:** 3/330 participants (0.9%)<br>**Pimecrolimus:** 0/328 participants (0%)<br>*(Difference between groups: p=20 [a])*<br><br>**<u>Skin infection</u>**<br>**Triamcinolone + hydrocortisone:** 80/330 participants (24%)		**<u>Severe adverse events</u>**<br>**Triamcinolone + hydrocortisone:** 21/330 participants (6.4%)<br>**Pimecrolimus:** 16/328 participants (4.9%)<br>*(Difference between groups: p=0.41 [a])*

For peer review only

				**Pimecrolimus:** 69/328 participants (21%)<br>*(Difference between groups: p=0.33 [a])*		
Mandelin 2010 [(62)]<br><br>***(Broeders 2016 [(53)])***	RCT<br><br>*(52 weeks, as prescribed until 7 days after clearance, then restarted with flares)*<br><br>*(Jadad score 3/5 – risk from sequence generation and allocation concealment, [(53)])*<br><br>*(Cochrane risk of bias tool: unclear risk of selection bias, risk from no blinding. [(3)])*	**Intervention:** Hydrocortisone butyrate 0.1% ointment (potent) and Hydrocortisone acetate 1% ointment (face) (Mild potency) twice daily, as prescribed, for a flare until 7 days after clearance, as many times as required in 1 year (n=40)<br><br>**Comparator:** Tacrolimus 0.1% ointment twice daily, as prescribed, for a flare until 7 days after clearance, as many times as required in 1 year (n=40)	**Severity:** moderate to severe<br><br>**Age:** adults<br><br>**Sample size:** 80 participants	**Skin thinning**<br>**Hydrocortisone:** 2/40 participants (5%)<br>**Tacrolimus:** 0/40 participants (0%)<br>*(Difference between groups: p=0.29 [a])*<br><br>**Skin infection**<br>**Hydrocortisone:** 17/40 participants (43%)<br>**Tacrolimus:** 26/40 participants (65%)<br>*(Difference between groups: p=0.05 [a])*		**Severe adverse events**<br>None in either group

For peer review only

Reitamo 2002 (I) [63]<br><br>***(Broeders 2016*** [53]***; Iskedjian 2004*** [64]***)***	RCT<br><br>*(3 weeks treatment)*<br><br>*(Jadad score 4/5 – risk from allocation concealment* [53]*)*<br><br>*(Cochrane risk of bias tool: unclear risk of selection bias, unclear risk from blinding.* [3]*)*<br><br>*(Jadad scale: 5/5,* [59]*)*<br><br>*(Cochrane risk of bias tool: Low risk of selection, performance, detection, attrition, reporting and other biases.* [54]*)*<br><br>*(Cochrane risk of bias tool: adequate randomisation and allocation concealment, blinding and ITT analysis done.* [65]*)*	**Intervention:** Hydrocortisone butyrate 0.1% twice daily for 3 weeks (n=186)<br><br>**Comparator:** Tacrolimus 0.1% twice daily for 3 weeks (n=191)<br><br>**Comparator:** Tacrolimus 0.03% twice daily for 3 weeks (arm not included in Broeders 2016 review) (n=193)	**Severity:** moderate to severe<br><br>**Age:** adults (age 16 to 70 years)<br><br>**Sample size: 571** participants	**Skin burning**<br>**Hydrocortisone:** 24/186 participants (13%)<br>**Tacrolimus 0.1%:** 113/191 participants (59%)<br>*(Difference between groups: p<0.00001* [a]*)*<br><br>**Pruritus**<br>**Hydrocortisone:** 18/186 participants (9.7%)<br>**Tacrolimus 0.1%:** 29/191 participants (15%)<br>*(Difference between groups: p=0.11* [a]*)*<br><br>**Erythema at application site**<br>**Hydrocortisone:** 1/186 participants (0.5%)<br>**Tacrolimus 0.1%:** 7/191 participants (3.7%)<br>**Tacrolimus 0.03%:** 4/193 participants (2.1%)<br>*(Difference between groups: tacrolimus 0.1% versus hydrocortisone: p=0.07* [a]*)*<br>*(Difference between groups: tacrolimus 0.03% versus hydrocortisone: p=0.23* [a]*)*		**Severe adverse events**<br>**Hydrocortisone:** 0/186 participants (0%)<br>**Tacrolimus 0.1%:** 1/191 participants (0.5%)<br>*(Difference between groups: p=0.51* [a]*)*<br><br>**Adverse events requiring discontinuation**<br>**Hydrocortisone:** 3/186 participants (1.6%)<br>**Tacrolimus 0.1%:** 8/191 participants (4.2%)<br>*(Difference between groups: p=0.15* [a]*)*

For peer review only

Reitamo 2005 [(66)]<br><br>***(Broeders 2016*** ***[(53)])***	RCT<br><br>*(26 weeks)* twice daily treatment until 7 days after clearance, then whenever a flare occurs)<br><br>*(Jadad score 5/5; [(53)])*<br><br>*(Cochrane risk of bias tool: unclear risk of selection bias,, low risk from blinding. [(3)])*<br><br>*(Jadad scale: 5/5, [(59)])*<br><br>*(Cochrane risk of bias tool: High risk of attrition bias. Low risk of selection, performance, detection, reporting and other biases. [(54)])*<br><br>*(Cochrane risk of bias tool: unclear risk of selection bias, unclear if blinded, and unclear if ITT analysis used. [(65)])*	**Intervention:** Hydrocortisone butyrate 0.1%(potent) **and** Hydrocortisone acetate 1% (face) (Mild potency) twice daily until 7 days after clearance of eczema each time a flare of eczema occurred for 6 months (n=485)<br><br>**Comparator:** Tacrolimus 0.1% twice daily until 7 days after clearance of eczema each time a flare of eczema occurred for 6 months (n=487)	**Severity:** moderate to severe<br><br>**Age:** adults (≥18 years old)<br><br>**Sample size:** 972 participants	**Adverse events related to treatment**<br>**Hydrocortisone:** 11/485 participants (2.3%)<br>**Tacrolimus:** 7/487 participants (1.4%)<br>*(Difference between groups: p=0.34 [a])*<br><br>**Skin burning**<br>**Hydrocortisone:** 67/485 participants (14%)<br>**Tacrolimus:** 255/487 participants (52%)<br>*(Difference between groups: p<0.00001 [a])*<br><br>**Pruritus**<br>**Hydrocortisone:** 65/485 participants (13%)<br>**Tacrolimus:** 88/487 participants (18%)<br>*(Difference between groups: p=0.05 [a])*<br><br>**Adverse events requiring discontinuation**<br>**Hydrocortisone:** 16/485 participants (3.3%)<br>**Tacrolimus:** 10/487 participants (2%)<br>*(Difference between groups: p=0.23 [a])*<br><br>**Skin thinning**<br>**Hydrocortisone:** 2/485 participants (0.4%)<br>**Tacrolimus:** 0/487 participants (0%)<br>*(Difference between groups: p=0.30 [a])*<br><br>**Skin infection**<br>**Hydrocortisone:** 9/485 participants (1.9%)<br>**Tacrolimus:** 13/487 participants (2.7%)<br>*(Difference between groups: p=0.40 [a])*		**Severe adverse events**<br>**Hydrocortisone:** 9/485 participants (1.9%)<br>**Tacrolimus:** 5/487 participants (1%)<br>*(Difference between groups: p=0.29 [a])*<br><br>**Adverse events requiring discontinuation**<br>**Hydrocortisone:** 16/485 participants (3.3%)<br>**Tacrolimus:** 10/487 participants (2.1%)<br>*(Difference between groups: p=0.23 [a])*

For peer review only

Gradman 2007 [67]<br>***(Svensson 201*** [68]***1)***	Crossover RCT<br>*(2 weeks treatment)*<br>*(Cochrane risk of bias tool: low risk of selection bias from sequence generation, but unclear for allocation concealment. Low risk from blinding.* [3]*)*	**Intervention:** Mometasone furoate 0.1% once daily<br>**Comparator:** Tacrolimus 0.1% twice daily	**Severity:** mild to moderate<br>**Age:** children 5 to 12 years<br>**Sample size:** 20 participants			**Withdrawal from study**<br>**Mometasone:** 1 patient<br>**Tacrolimus**: 1 patient
Kawashima 1997 [69]<br>(Ashcroft 2005 [59])	RCT<br>*(3 weeks treatment)*<br>*(Jadad scale: 5/5,* [59]*)*	**Intervention:** Betamethasone valerate 0.12% twice daily for three weeks (n=89)<br>**Comparator:** tacrolimus 0.1% twice daily for three weeks (n=92)	**Severity:** mild to moderate<br>**Age:** adults<br>**Sample size:** 181 participants	**Skin infections**<br>**Betamethasone:** 5/89 participants<br>**Tacrolimus:** 6/92 participants<br>(*Difference between groups: p=0.80* [a])<br>**Skin burning**<br>**Betamethasone:** 3/89 participants<br>**Tacrolimus:** 25/92 participants<br>(*Difference between groups: p=0.0004* [a])		
**Potent or mild potency topical corticosteroids**						
Hofman 2006 [70]<br>***(Broeders 2016*** [53]***; Siegfried 2016*** [71]***)***	RCT<br>*(2 weeks treatment, 28 weeks follow up)*<br>*(Jadad score 5/5 – risk from sequence generation and allocation concealment* [53]*)*	**Intervention:** Hydrocortisone ointment 1% (mild potency) twice daily for head/neck and hydrocortisone butyrate ointment 0.1% (potent) for trunk and limbs for 2 weeks then hydrocortisone 1% (mild potency) twice daily for flares. (n=124)<br>**Comparator:** Tacrolimus 0.03% twice daily for 3 weeks then tacrolimus once daily and vehicle once daily for flares (n=133)	**Severity:** moderate to severe<br>**Age:** children 2 to 11 years old (mean 6 years old)<br>**Sample size:** 257 participants	**Adverse events related to treatment**<br>**Hydrocortisone:** 2/124 participants (1.6%)<br>**Tacrolimus:** 10/133 participants (7.5%)<br>*(Difference between groups: p=0.04* [a]*)*<br>**Skin burning**<br>**Hydrocortisone:** 0/124 participants (0%)<br>**Tacrolimus:** 2/133 participants (1.5%)<br>*(Difference between groups: p=0.32* [a]*)*<br>**Pruritus**<br>**Hydrocortisone:** 4/124 participants (3%)<br>**Tacrolimus:** 8/133 participants (6%)		**Severe adverse events**<br>**Hydrocortisone:** 0/124 participants (0%)<br>**Tacrolimus:** 2/133 participants (1.5%)<br>(*Difference between groups: p=0.32* [a]*)*

For peer review only - http://bmjopen.bmj.com/site/about/guidelines.xhtml

				(Difference between groups: p=0.30 [a])<br><br>**<u>Skin infection</u>**<br>**Hydrocortisone:** 4/124 participants (3.2%)<br>**Tacrolimus:** 2/133 participants (1.5%)<br>*(Difference between groups: p=0.37 [a])*<br><br>**<u>Bacterial infection</u>**<br>**Hydrocortisone:** 3/124 participants (2%)<br>**Tacrolimus:** 33/133 participants (2%)<br>*(Difference between groups: p<0.0001)*<br><br>**<u>Viral infection</u>**<br>**Hydrocortisone:** incidence not reported<br>**Tacrolimus:** 1/133 participants (0.8%)		
**Moderate potency topical corticosteroids**						
Sikder 2005 [(72)]<br><br>***(Broeders 2016*** [(53)]***)***	RCT<br><br>*(4 weeks treatment)*<br><br>*(Jadad score 2/5 – risk from sequence generation and allocation concealment, no blinding of observer or patients,* [(53)]*)*<br><br>*(Cochrane risk of bias tool: Unclear risk of selection and detection bias. Low risk of performance, attrition, reporting and other biases.* [(54)]*)*<br><br>*(Cochrane risk of bias tool: low risk of selection bias, from blinding of participants and missing data. Unclear risk from*	**Intervention:** Clobetasone 0.05% twice daily (n=15)<br><br>Tacrolimus 0.03% twice daily (n=15)	**Severity:** moderate to severe<br><br>**Age:** children 7 to 15 years old<br><br>**Sample size:** 30 participants	**<u>Skin burning</u>**<br>**Clobetasone:** 1/15 participants (6.7%)<br>**Tacrolimus**: 7/15 participants (47%)<br>*(Difference between groups: p=0.05 [a, d])*<br><br>**<u>Pruritus</u>**<br>**Clobetasone:** 2/15 participants (13%)<br>**Tacrolimus**: 3/15 participants (20%)<br>*(Difference between groups: p=0.63 [a])*		

For peer review only

	blinding outcome assessors, reporting and other biases [56]).					
Torok 2003 [73]<br>***(Svensson 2011 [68])***	RCT<br>*(3 weeks treatment)*<br>*(Delphi list: method of randomisation not described, allocation not concealed, blinded assessors but not participants, ITT analysis, [29])*	**Intervention:** Clocortolone pivalate 0.1% twice daily (n=19)<br>**Intervention:** Clocortolone 0.1% + Tacrolimus 0.1% twice daily (n=19)<br>**Comparator:** Tacrolimus 0.1% twice daily (n=19)	**Severity:** not specified in the review<br>**Age:** adults 16 to 65 years<br>**Sample size:** 57 participants	**Skin irritation**<br>Most commonly reported adverse event<br>**Skin burning**<br>More frequent in those treated with Tacrolimus 0.1%.<br>**Pruritus**<br>Commonly reported in both arms.<br>*(No numerical data provided in the review)*		
**Moderate or mild potency topical corticosteroids**						
Sigurgeirsson 2015 [74]<br>***(Broeders 2016 [53]; Siegfried 2016 [71])***	RCT<br>*(260 weeks used until clearance or according to country's label. Medication reinstated when a flare occurred))*<br>*(Jadad score 3/5 – risk from allocation concealment, no blinding of observer or patients, [53])*	**Intervention:** A moderate potency or mild potency TCS used according to the country's label with potency selected by the investigator (n=1213)<br>**Comparator:** Pimecrolimus 1% twice daily (n=1205)	**Severity:** mild to moderate<br>**Age:** children age 3 to 12 months old (mean 7 months)<br>**Sample size:** 2418 participants	**Skin thinning*(from online correspondence)***<br>**Topical corticosteroid:** 1/1213 participants (0.08%)<br>**Pimecrolimus:** 0/1205 (0%)<br>*(Difference between groups: p=0.50 [a])*<br>**Skin infection**<br>**Topical corticosteroid:** 150/1213 participants (12%)<br>**Pimecrolimus:** 157/1205 participants (13%)<br>*(Difference between groups: p=0.62 [a])*<br>**Cutaneous bacterial infection**<br>**Topical corticosteroid:** 121/1213 participants (10%)<br>**Pimecrolimus:** 145/1205 participants (12%)<br>*(Difference between groups: p=0.11 [a])*<br>**Cutaneous viral infection**<br>**Topical corticosteroid:** 279/1213 participants (23%)<br>**Pimecrolimus:** 301/12 05 participants (25%)	**Systemic bacterial infection**<br>**Topical corticosteroid:** 206/1213 participants (17%)<br>**Pimecrolimus:** 205/1205 participants (17%)<br>*(Difference between groups: p=0.98 [a])*<br>**Systemic viral infection**<br>**Topical corticosteroid:** 206/1213 participants (17%)<br>**Pimecrolimus:** 205/1205 participants (17%)<br>*(Difference between groups: p=0.98 [a])*<br>**Systemic RTI**<br>**Topical corticosteroid:** 388/1213 participants (32%)<br>**Pimecrolimus:** 422/1205 participants (35%)<br>*(Difference between groups: p=0.11 [a])*<br>**Systemic GI**<br>**Topical corticosteroid:** 376/1213 participants (31%)<br>**Pimecrolimus:** 386/1205 participants (32%)	**Severe adverse events**<br>**Topical corticosteroid:** 210/1213 participants (17%)<br>**Pimecrolimus:** 247/1205 participants (20%)<br>*(Difference between groups: p=0.05 [a])*<br>**Adverse events requiring discontinuation**<br>**Topical corticosteroid:** 12/1213 participants (1.0%)<br>**Pimecrolimus:** 7/1205 participants (0.6%)<br>*(Difference between groups: p=0.26 [a])*

				(Difference between groups: p=0.25 [a]*)*	*(Difference between groups: p=0.58*[a]*)*<br><br>**<u>Lymphoma</u>**<br>Zero cases in either group<br><br>**<u>Growth rate and immune system</u>**<br>No difference between groups	
**Mild potency topical corticosteroids**						
Reitamo 2002 (II) [75] ***(Broeders 2016*** [53]***; Iskedjian 2004*** [64]***)***	RCT<br><br>*(3 weeks treatment)*<br><br>*(Jadad score 5/5 – risk from allocation concealment* [53]*)*<br><br>*(Cochrane risk of bias tool: unclear risk of selection bias, unclear risk of blinding* [3]*)*<br><br>*(Jadad scale: 5/5,* [59]*)*<br><br>*(Cochrane risk of bias tool: Low risk of selection, performance, detection, attrition, reporting and other biases.* [54]*)*<br><br>*Cochrane risk of bias tool: low risk of selection, performance, attrition, reporting and other bias. Unclear risk from blinding outcome assessors* [56]*).*<br><br>*(Cochrane risk of bias tool: adequate method of randomisation and allocation concealment, blinding done, ITT used.* [65]*)*	**Intervention:** Hydrocortisone acetate 1% twice daily (n=185)<br><br>**Comparator:** Tacrolimus 0.1% ointment twice daily (n=186)<br><br>**Comparator:** Tacrolimus 0.03% ointment twice daily (arm not included in Broeders 2016 review) (n=189)	**Severity:** moderate to severe<br><br>**Age:** children 2 to 15 years old<br><br>**Sample size:** 560participants	**<u>Skin burning</u>**<br>**Hydrocortisone:** 13/185 participants (7%)<br>**Tacrolimus 0.1%:** 38/186 participants (20%)<br>*(Difference between groups: p=0.004* [a]*)*<br><br>**<u>Pruritus</u>**<br>**Hydrocortisone:** 14/185 participants (7.6%)<br>**Tacrolimus 0.1%:** 21/186 participants (11%)<br>*(Difference between groups: p=0.22* [a]*)*<br><br>**<u>Skin infection</u>**<br>**Hydrocortisone:** 4/185 participants (2.2%)<br>**Tacrolimus 0.1%:** 4/186 participants (2.2%)<br>*(Difference between groups: p=0.99* [a]*)*<br><br>**<u>Erythema at application site</u>**<br>**Hydrocortisone:** 3/185 participants (1.6%)<br>**Tacrolimus 0.1%:** 1/186 participants (0.5%)<br>**Tacrolimus 0.03%:** 4/189 participants (2.1%)<br>*(Difference between groups, hydrocortisone vs tacrolimus 0.1%: P=0.34* [a]*)*<br>*(Difference between groups, hydrocortisone vs tacrolimus 0.03%: P=0.72* [a]*)*		**<u>Severe adverse events</u>**<br>**Hydrocortisone:** 2/185 participants (1.1%)<br>**Tacrolimus 0.1%:** 1/186 participants (0.5%)<br>*(Difference between groups: (p=0.57* [a]*)*<br><br>**<u>Adverse events requiring discontinuation</u>**<br>**Hydrocortisone:** 4/185 participants (2.2%)<br>**Tacrolimus 0.1%:** 3/186 participants (1.6%)<br>*(Difference between groups: p=0.70* [a]*)*

For peer review only

Reitamo 2004 [76]<br><br>***(Broeders 2016 [53])***	RCT<br><br>*(3 weeks treatment)*<br><br>*(Jadad score 3/5 – risk from sequence generation and allocation concealment [53])*<br><br>*(Cochrane risk of bias tool: unclear risk of selection bias. Unclear risk from blinding. [3])*<br><br>*(Jadad scale: 4/5, [59])*<br><br>*(Cochrane risk of bias tool: Unclear risk of selection bias (allocation concealment). Low risk of selection bias (random sequence generation), performance, detection, attrition, reporting and other biases. [54])*<br><br>*Cochrane risk of bias tool: low risk of selection, performance, attrition, reporting and other bias. Unclear risk from blinding outcome assessors [56]).*	**Intervention:** Hydrocortisone acetate 1% twice daily (n=207)<br><br>**Comparator:** Tacrolimus 0.03% twice daily (n=210)	**Severity:** moderate to severe<br><br>**Age:** children 2 to 15 years old<br><br>**Sample size:** 417 participants	**<u>Skin burning</u>**<br>**Hydrocortisone:** 30/207 participants (15%)<br>**Tacrolimus:** 50/210 participants (24%)<br>*(Difference between groups: p=0.02 [a])*<br><br>**<u>Pruritus</u>**<br>**Hydrocortisone:** 33/207 participants (16%)<br>**Tacrolimus:** 45/210 participants (21%)<br>*(Difference between groups: p=0.15 [a])*<br><br>**<u>Skin infection</u>**<br>**Hydrocortisone:** 6/207 participants (2.9%)<br>**Tacrolimus:** 6/210 participants (2.9%)<br>*(Difference between groups: p=0.98 [a])*		**<u>Severe adverse events</u>**<br>**Hydrocortisone:** 3/207 participants (1.4%)<br>**Tacrolimus:** 3/210 participants (1.4%)<br>*(Difference between groups: p=0.99 [a])*<br><br>**<u>Adverse events requiring discontinuation</u>**<br>**Hydrocortisone:** 6/207 participants (2.9%)<br>**Tacrolimus:** 8/210 participants (3.8%)<br>*(Difference between groups: p=0.61 [a])*
**Potency of topical corticosteroids unknown**						
Gutgesell 1998 [77]<br><br>(abstract only)<br><br>***(Penaloza Hidalgo 2004 [65])***	Within-participant RCT<br><br>*(3 weeks treatment)*<br><br>*(Cochrane risk of bias tool: randomisation and allocation concealment method inadequate, unclear if blinded ,ITT analysis used [65])*	**Intervention:** Topical corticosteroids on one side of the body, twice daily<br><br>**Comparator:** Tacrolimus 0.1% on one side of the body, twice daily	**Severity:** severe<br><br>**Age:** adults (22 to 36 years)<br><br>**Sample size:** 7 participants	**<u>Skin burning</u>**<br>**Topical corticosteroids:** 0/7 (0%)<br>**Tacrolimus:** 2/7 participants (29%)		
Arellano 2007 [78]<br><br>***(Ashcroft 2007 [60]**;* Cury Martins 2015 [54]	Nested case-control<br><br>*(Duration not specified in the review)*	**Intervention:** Topical corticosteroids at different potencies<br><br>**Comparator:** pimecrolimus or tacrolimus	**Severity:** not specified in the review<br><br>**Age:** not specified in the review		**<u>Lymphoma</u>**<br>No increased risk of lymphoma with TCI or TCS when compared against controls.<br>***Super potent TCS:*** OR 1.2, 95% CI 0.8 to 1.8	

For peer review only

	Risk of bias not assessed in any of the included systematic reviews.	**Comparator:** controls (not specified in the review)	**Sample size:** 294 cases/293,000 controls		***Low potency TCS:*** *OR* 1.1, 95%CI 0.7 to 1.6<br>***Pimecrolimus:*** OR 0.8, 95%CI 0.4 to 1.6<br>***Tacrolimus*** OR 0.8, 95% CI 0.4 to 1.7	
Arellano 2009 [79]<br><br>(Cury Martins 2015 [54])	Cohort<br><br>*(followed up between 1992 to 2006)*<br><br>*Risk of bias not assessed in any of the included systematic reviews.*	**Intervention:** Topical corticosteroids at different potencies<br><br>**Comparator:** pimecrolimus or tacrolimus	**Severity:** not specified in the review<br><br>**Age:** not specified in the review<br><br>**Sample size:** > 3,000,000		**Lymphoma**<br>Increased risk with topical corticosteroids (related to potency) but no numerical data given. Insufficient data to assess TCI-related risks.	
Schneeweiss 2009 [80]<br><br>***(Cury Martins 2015 [54])***	Cohort<br><br>*(followed up between the years of 2002 to 2006)*<br><br>*Risk of bias not assessed in any of the included systematic reviews.*	**Intervention: mid to potent** topical corticosteroids (n=1,043,025)<br><br>**Comparator:** pimecrolimus (n=118,863) or tacrolimus (n=38,757)<br><br>(also a comparison with untreated dermatitis (n=118,825) and general population (n=118,863) .)	**Severity:** not specified<br><br>**Age:** median 1.3 years<br><br>**Sample size:** 1,438,333 participants		**Lymphoma**<br>Very small non-significant increased risk in TCI and TCS patients when compared with the general population, but with similar risks between the treatment groups	
Reitamo 2000 [81]<br><br>***(Cury Martins 2015 [54])***	Open label, single group<br><br>*(6 to 12 months of treatment)*<br><br>*Risk of bias not assessed in any of the included systematic reviews.*	**Intervention:** No steroids (except prior to treatment)<br><br>**Comparator:** Tacrolimus 0.1%	**Severity:** not specified in the review<br><br>**Age:** adults<br><br>**Sample size:** 316 participants	**Skin thinning**<br>One participant had skin thinning when using TCS prior to treatment with tacrolimus – but this ameliorated after 6 months of treatment with tacrolimus.		
**Is there any difference in safety of topical corticosteroids of different potencies?**						
**Study ID *(Systematic review*)***	**Study design and study duration *(Quality assessment)***	**Intervention and comparator**	**Participants**	**Cutaneous adverse events**	**Systemic adverse events**	**Unspecified adverse events**
**Potent topical corticosteroid versus moderate potency topical corticosteroid**						
Ulrich 1991 [82]<br><br>***(Hoare 2000 [19])***	RCT<br><br>*(2 weeks treatment)*<br><br>*(Moher 1995 quality checklist: method and*	**Intervention:** 0.05% halomethasone cream, twice daily (Assume potent)	**Severity:** not specified in the review			No adverse events

For peer review only

	concealment of randomisation unclear, concerns over subgroup analysis [(19)])	**Comparator:** 0.25% prednicarbate cream, twice daily (moderate potency)	**Age:** not specified in the review<br>**Sample size:** 165 participants			
Smitt 1993 [(83)]<br>***(Callen 2007 [(24)])***	RCT<br>*(3 weeks treatment)*<br>*Risk of bias not assessed in any of the included systematic reviews.*	**Intervention:** Trimaconiolone acetonide 0.1%, twice daily (potent)<br>**Comparator:** Alclomethasone cream, twice daily (moderate)	**Severity:** not specified in the review<br>**Age:** children 1 to 15 years<br>**Sample size:** 40 participants		**<u>HPA axis suppression</u>**<br>There was suppression after 2 weeks, but no further after 3 (no further details).	
**Potent topical corticosteroid versus mild potency topical corticosteroid**						
Lebrun-Vignes 2000 [(84)]<br>***(Nankervis 2017 [(3)])***	RCT<br>*(15 days treatment, 30 days follow up)*<br>*(Cochrane risk of bias tool: unclear risk of selection bias and unclear risk from blinding. [(3)])*	**Intervention:** Micronized desonide cream 0.1% (mild potency) 1 to 5 days twice daily (in hospital), days 6 and 7 once daily, then alternate days until day 15 (n=15)<br>**Comparator:** Betamethasone dipropionate cream 0.05% (potent) 1 to 5 days twice daily (in hospital), days 6 and 7 once daily, then alternate days until day 15 (n=14)	**Severity:** severe<br>**Age:** children ≤ 8 years<br>**Sample size:** 29 participants			There were no adverse events in either group
Prado de Oliveira 2002 [(85)]<br>***(Nankervis 2017 [(3)])***	RCT<br>*(Up to 42 days treatment)*<br>*(Cochrane risk of bias tool: unclear risk of selection bias and unclear risk from blinding. [(3)])*	**Intervention:** Mometasone furoate 0.1% once daily after a bath (N=13) (potent)<br>**Comparator:** Desonide cream 0.05% once a day after a bath (N=12) (mild potency)	**Severity:** not specified in the review<br>**Age:** children 2 to 12 years<br>**Sample size:** 25 participants	**<u>Signs of mild thinning</u>**<br>**Mometasone furoate:** 4/13 participants (31%)<br>**Desonide:** 2/12 participants (17%)<br>*(Difference between groups: p=0.42 [a])*		
Hanifin 1996 [(86)]<br>***(Callen 2007 [(24)])***	Matched case control<br>*(3 weeks treatment)*<br>*Risk of bias not assessed in any of the included systematic reviews.*	**Intervention:** Mometasone cream (potent)<br>**Comparator:** Hydrocortisone cream (mild potency)	**Severity:** not specified in the review<br>**Age:** children 6 months to 2 years<br>**Sample size:** 62 participants		**<u>HPA axis suppression</u>**<br>**Mometasone:** 1 abnormal cotrosyn simulation test	

For peer review only

Kirkup 2003 [87] (trial a)<br>***(Tang 2014 [88]; Siegfried 2016 [71])***<br>Most safety data presented was combined with Kirkup 2003 (trial b) (see same potency section below)	RCT<br>*(16 weeks: twice daily for 2-4 weeks until stabilised then 'as required' for 12 weeks)*<br>*(Cochrane risk of bias tool: unclear risk of selection bias, unclear risk from blinding. [3])*	**Intervention:** Fluticasone propionate 0.005% ointment (potent), twice daily for 2-4 weeks until stabilised then 'as required' for 12 weeks (n=70)<br>**Comparator:** Hydrocortisone 1% cream (mild potency), twice daily for 2-4 weeks until stabilised then 'as required' for 12 weeks (n=67)	**Severity: moderate**<br>**Age:** children (age 2-14 years old)<br>**Sample size (maintenance phase):** 137 participants	**Ringworm and folliculitis**<br>1 participant but not clear which group<br>**Kirkup 2003a and b: Bacterial infection**<br>**Fluticasone:** 1/136 participants (0.7%)<br>**Hydrocortisone:** 3/129 participants (2%)<br>*(Difference between groups: p = 0.32 [a])*<br>**Kirkup 2003a and b: Fungal infection**<br>**Fluticasone:** 1/136 participants (0.7%)<br>**Hydrocortisone:** 0/129 (0%)<br>*(Difference between groups: p = 0.52 [a])*	**Kirkup 2003a and b viral infection**<br>**Fluticasone:** 5/136 participants (4%)<br>**Hydrocortisone:** 5/129 participants (4%)<br>*(Difference between groups: p = 0.93 [a])*<br>**Kirkup 2003a and b: Respiratory tract infection**<br>**Fluticasone:** 8/136 participants (6%)<br>**Hydrocortisone**: 5/129 participants (4%)<br>*(Difference between groups: p = 0.45 [a])*	**Kirkup 2003a and b: Discontinuation due to adverse events**<br>**Fluticasone:** 1/136 participants (0.7%)<br>**Hydrocortisone:** 1/129 participants (0.7%)<br>*(Difference between groups: p = 0.97 [a])*
**Moderate potency topical corticosteroid versus mild potency topical corticosteroid**						
Kuokkanen 1987 [89]<br>***(Hoare 2000 [19])***	RCT, within participant<br>*(3 weeks treatment)*<br>*(Moher 1995 quality checklist: method and concealment of randomisation unclear, double blinded study, three dropouts/ withdrawals, no ITT, [19])*	**Intervention:** Alclometasone dipropionate 0.05% twice daily (moderate potency)<br>**Comparator:** Hydrocortisone 1% twice daily (mild potency)	**Severity:** not specified in the review<br>**Age:** children<br>**Sample size:** 37 participants	No evidence of skin thinning		
**Various potencies**						
Ellison 2000 [90]<br>***(Callen 2007 [24]; Eichenfield 2014 [91])***	Observational study<br>*(Duration 0.7 to 18.7 years)*<br>*Risk of bias not assessed in any of the included systematic reviews.*	**Intervention:** Mild, moderate, potent topical corticosteroids	**Severity:** Disease severity score 5-8<br>**Age:** children and adolescents (0.7 to 18.7 years)<br>**Sample size:** 35 participants		**HPA axis suppression**<br>**Mild potency topical corticosteroids:** no change in plasma cortisol levels<br>**Potent topical corticosteroids:** suppression in 4/4 (100%) patients	

For peer review only

Kristmundsdottir 1987 [92]<br>***(Eichenfield 2014 [91])***	Observational study<br>*(Duration not specified in the review)*<br>*Risk of bias not assessed in any of the included systematic reviews.*	**Intervention:** Different potencies of topical corticosteroids	**Severity:** not specified in the review<br>**Age:** not specified in the review<br>**Sample size:** not specified in the review		Review authors reported "Also concerns for negative effects on linear growth, although reports have given mixed conclusions"	
Patel 1997 [93]<br>***(Eichenfield 2014 [91])***	Observational study<br>*(Duration not specified in the review)*<br>*Risk of bias not assessed in any of the included systematic reviews.*	**Intervention:** Four different potency topical corticosteroids	**Severity:** not specified in the review<br>**Age:** not specified in the review<br>**Sample size:** not specified in the review		Review authors reported "Also concerns for negative effects on linear growth, although reports have given mixed conclusions"	
Patel 1998 [94]<br>***(Eichenfield 2014 [91])***	Observational study<br>*(Duration not specified in the review)*<br>*Risk of bias not assessed in any of the included systematic reviews.*	**Intervention:** Different potencies of topical corticosteroids	**Severity:** not specified in the review<br>**Age:** not specified in the review<br>**Sample size:** not specified in the review		Review authors reported "Also concerns for negative effects on linear growth, although reports have given mixed conclusions"	
**Is there any difference in safety between topical corticosteroids of the same potency?**						
**Study ID *(Systematic review*)***	**Study design and study duration *(Quality assessment)***	**Intervention and comparator**	**Participants**	**Cutaneous adverse events**	**Systemic adverse events**	**Unspecified adverse events**
**Potent topical corticosteroid versus another potent topical corticosteroid**						
Kirkup 2003 [87] (trial b)<br>***(Tang 2014 [88]; Siegfried 2016 [71])***<br>Most safety data presented was combined with Kirkup 2003	RCT<br>*(16 weeks: twice daily for 2-4 weeks until stabilised then intermittently for 12 weeks)*<br>*(Cochrane risk of bias tool: unclear risk of selection bias, unclear risk from blinding. [3])*	**Intervention:** Fluticasone propionate 0.005% ointment, twice daily for 2-4 weeks until stabilised then intermittently for 12 weeks (n=66)<br>**Comparator:** Hydrocortisone butyrate 0.1% cream (potent), twice daily for 2-4 weeks until	**Severity:** moderate<br>**Age:** children (age 2-14 years old)<br>**Sample size:** n=128	**Ringworm and folliculitis**<br>None reported<br>**Kirkup 2003a and b: Bacterial infection**<br>**Fluticasone:** 1/136 participants (0.7%)<br>**Hydrocortisone:** 3/129 participants (2%)	**Kirkup 2003a and b viral infection**<br>**Fluticasone:** 5/136 participants (4%)<br>**Hydrocortisone:** 5/129 participants (4%)<br>*(Difference between groups: p = 0.93 [a])*<br>**Kirkup 2003a and b: Respiratory tract infection**	**Kirkup 2003a and b: Discontinuation due to adverse events**<br>**Fluticasone:** 1/136 participants (0.7%)<br>**Hydrocortisone:** 1/129 participants (0.7%)<br>*(Difference between groups: p = 0.97 [a])*

(trial a) (see different potency section above)		stabilised then intermittently for 12 weeks (n=62)		*(Difference between groups: p = 0.32 [a])*<br>**Kirkup 2003a and b: Fungal infection**<br>**Fluticasone:** 1/136 participants (0.7%)<br>**Hydrocortisone:** 0/129 (0%)<br>*(Difference between groups: p = 0.52 [a])*	**Fluticasone:** 8/136 participants (6%)<br>**Hydrocortisone**: 5/129 participants (4%)<br>*(Difference between groups: p = 0.45 [a])*	
**Moderate potency topical corticosteroid versus another moderate potency topical corticosteroid**						
Aliaga 1994 [95]<br>***(De Tiedra 1997 [33])***	RCT<br>*(21 days treatment)*<br>*Risk of bias not assessed in any of the included systematic reviews.*	**Intervention:** Prednicarbate ointment 0.25%, twice daily (moderate potency) (n=36)<br>**Comparator:** Flucortin ointment 0.75%, twice daily (assumed moderate potency) (n=31)	**Severity:** Disease duration – mean 7.7 years (range 0.1 to 31).<br>**Age:** adults 18-74 years (mean 33.6)<br>**Sample size:** 67 participants			**Adverse reactions**<br>**Prednicarbate:** 0/36 patients (0%)<br>**Flucortin:** 2/31 patients (6.5%)<br>*(Difference between groups: p = 0.16 [a])*
**Mild potency topical corticosteroid versus another mild potency topical corticosteroid**						
Lucky 1997 [96]<br>***(Callen 2007 [24]; Hoare 2000 [19]; Wood Heickman 2018 [9])***	RCT<br>*(4 weeks treatment)*<br>*(Moher 1995 quality checklist: method and concealment of randomisation unclear, open label, five dropouts, no ITT [19])*	**Intervention:** Desonide 0.05% ointment, twice per day (mild potency)<br>**Comparator:** Hydrocortisone 2.5% ointment, twice per day (mild potency)	**Severity:** not specified in the review<br>**Age:** children (mean or median is 4.7 years)<br>**Sample size:** 20 participants		**HPA axis suppression**<br>Normal in both groups (measured using ACTH stimulation testing, measuring serum cortisol levels)	
Jorizzo 1995 [97]<br>***(Siegfried 2016 [71]; Froeschl 2007 [98])***	RCT<br>*(25 weeks: 5 weeks of treatment, 20 weeks follow up)*<br>*(Moher 1995 quality checklist: method and concealment of randomisation unclear, investigator blind, two dropouts/withdrawals, no ITT [19])*	**Intervention:** 0.05% desonide twice daily (n=16) (mild potency)<br>**Comparator:** 1% hydrocortisone ointment twice daily (n=20) (mild potency)	**Severity:** mild to moderate<br>**Age:** children 5 years and under<br>**Sample size:** 36 participants	**Skin thinning**<br>No cases - measured by a magnifying lamp		

How safe are topical corticosteroids compared to Chinese herbal medicine?						
**Study ID** ***(Systematic review*)***	**Study design and study duration** ***(Quality assessment)***	**Intervention and comparator**	**Participants**	**Cutaneous adverse events**	**Systemic adverse events**	**Unspecified adverse events**
**Very potent topical corticosteroids**						
Huang 2010 [99]<br>***(Gu 2013 [100]; Gu 2014 [101])***	RCT<br>*(2 weeks treatment, followed up for 12 weeks after)*<br>*(Cochrane risk of bias tool: low risk of selection bias (random sequence generation), and other biases. Unclear risk of selection (allocation concealment), detection and attrition bias. High risk of performance and reporting bias. [100, 101]*	**Intervention:** Clobetasol propionate ointment, 3 times daily (n=97)<br>**Comparator:** Chushi Zhiyang ointment, 3 times daily (n=98)	**Severity:** not specified in the review<br>**Age:** children and adults, 3 months to 22 years<br>**Sample size:** 195 participants	**<u>Cutaneous adverse events</u>**<br>**Clobetasol:** 5/97 participants (5%)<br>**Chinese herbal medicine:** 0/98 participants (0%)<br>*(Difference between groups: p=0.10 [a, e])*<br>The five events were pigmentation (unclear if hyper- or hypo-)		
**Potent topical corticosteroids**						
Chen 2011 [102]<br>***(Gu 2013 [100]; Gu 2014 [101])***	RCT<br>*(2 weeks treatment)*<br>*(Cochrane risk of bias tool: unclear risk of selection, detection, attrition, reporting and other bias. High risk of performance bias [100, 101])*	**Intervention:** Mometasone furoate cream, once daily (n=50)<br>**Comparator:** Huanglian Qingdai ointment, 2 to 3 times daily (n=50)	**Severity:** not specified in the review<br>**Age:** children 58 days to 2 years<br>**Sample size:** 100 participants	**<u>Cutaneous adverse events</u>**<br>**Mometasone:** 6/50 participants (12%)<br>**Chinese herbal medicine:** 0/50 participants (0%)<br>*(Difference between groups: p=0.08 [a, f])*<br>Minor adverse events such as burning, dryness and scaling of the skin were reported in the TCS groups		
Dong 2012 [103]<br>***(Gu 2014 [101])***	RCT<br>*(2 weeks treatment)*<br>*(Cochrane risk of bias tool: unclear risk of selection, detection, attrition, and reporting bias. High risk of*	**Intervention:** Hydrocortisone butyrate cream, twice daily (n=47)<br>**Comparator:** Jingfang mixture solution, twice daily (n=48)	**Severity:** not specified in the review<br>**Age:** children 0.5 to 5.5 years<br>**Sample size:** 95 participants	Minor adverse events such as burning, dryness and scaling of the skin were reported in the TCS groups.<br>*(No numerical data provided in the review)*		

For peer review only

	performance bias. Low risk of other biases. [(101)])					
Xu 2012 [(104)]<br><br>***(Gu 2014 [(101)])***	RCT<br><br>*(2 weeks treatment)*<br><br>*(Cochrane risk of bias tool: unclear risk of selection, detection, attrition, and reporting bias. High risk of performance and other biases. [(101)])*	**Intervention:** Triamcinolone acetonide acetate cream, twice daily (n=51)<br><br>**Comparator:** Kouqiang Xiaoyan powder, twice daily (n=53)	**Severity:** not specified in the review<br><br>**Age:** children 35 days to 2 years<br><br>**Sample size:** 104 participants			No adverse events in either group
**How safe is more frequent topical corticosteroid application compared with once daily application?**						
**Study ID *(Systematic review*)***	**Study design and study duration *(Quality assessment)***	**Intervention and comparator**	**Participants**	**Cutaneous adverse events**	**Systemic adverse events**	**Unspecified adverse events**
**Very potent topical corticosteroid**						
Schlessinger 2006 [(105)]<br><br>***(Nankervis 2017 [(3)]; Wood Heickman 2018 [(9)])***	Open label RCT<br><br>*(2 weeks treatment)*<br><br>*(Cochrane risk of bias tool: unclear risk of selection bias, high risk from no blinding. [(3)])*	**Intervention:** fluocinonide cream 0.1% applied once daily (n=63)<br><br>**Comparator:** fluocinonide cream 0.1% applied twice daily (n=63)	**Severity:** not specified in the review<br><br>**Age:** children, aged 12 to <18 years (cohort 1); 6 to <12 years (cohort 2); 2 to <6 years (cohort 3); and 3 months to <2 years (cohort 4).<br><br>**Sample size:** 126 participants		**<u>HPA axis suppression</u>**<br>**Once daily**: 0/63 (0%)<br>**Twice daily**: 3/63 (4.8%)<br>*(Difference between groups: P=0.19[a])*<br><br>(measured using ACTH stimulation testing, measuring serum cortisol levels)<br><br>After TCS discontinuation, children with biochemical adrenal insufficiency had complete resolution at retesting.	
**Potent topical corticosteroids**						
Bleehen 1995 [(106)]<br><br>***(Green 2004 [(107)])***	RCT<br><br>*(4 weeks treatment))*<br><br>*(Quality using NHS CRD criteria: method for randomisation/allocation concealment unknown, adequate blinding, and ITT used. [(107)])*<br><br>*(Moher 1995 quality checklist: method and*	**Intervention:** Fluticasone propionate 0.05% cream once daily (plus vehicle once daily for blinding) (n=137)<br><br>**Comparator:** Fluticasone propionate 0.05% cream twice daily (n=133)	**Severity:** at least moderate severity<br><br>**Age:** children and adults<br><br>**Sample size:** 270 participants			**<u>Number of events possibility, probably or almost certainly related to study medication</u>**<br>**Once daily:** 26 events<br>**Twice daily**: 24 events<br>(most were skin disorders)

For peer review only - http://bmjopen.bmj.com/site/about/guidelines.xhtml

	concealment of randomisation unclear, Probably investigator blinded but unclear, ITT analysis, [19])					
GSK report 1995 [108]<br><br>***(Green 2004*** **[107])**	RCT<br><br>*(4 weeks treatment)*<br><br>*(Quality using NHS CRD criteria: adequate method of randomisation /allocation concealment, adequate blinding, and ITT used. [107])*	**Intervention:** Fluticasone propionate 0.005% ointment once daily and placebo only daily (n=123)<br><br>**Comparator:** Fluticasone propionate 0.005% ointment twice daily (n=122)	**Severity:** at least moderate severity<br><br>**Age:** children and adults<br><br>**Sample size:** 245 participants			**Number of adverse events possibly related to medication**<br>**Once daily:** 6 events<br>**Twice daily**: 8 events<br><br>**Number of adverse events probably related to medication**<br>**Once daily:** 9 events<br>**Twice daily:** 3 events<br><br>**Number of adverse events almost certain related to medication**<br>**Once daily:** 6 events<br>**Twice daily:** 3 events<br><br>(Mainly included skin related disorders including exacerbation of eczema, pruritus and redness of skin)
Koopmans 1995 [109]<br><br>***(Green 2004*** **[107])**	RCT<br><br>*(4 weeks treatment))*<br><br>*(Quality using NHS CRD criteria: method for randomisation /allocation concealment unknown, partial blinding, and no ITT used. [107])*<br><br>*(Moher 1995 quality checklist: method and concealment of randomisation unclear, double blinded, one dropout, no ITT analysis, [19])*	**Intervention:** Locoid lipocream (0.1% hydrocortisone 17-butyrate) once daily and locobase once daily (n=75)<br><br>**Comparator:** Locoid lipocream twice daily (n=75)	**Severity:** not specified in the review<br><br>**Age:** children aged over 12 years and adults<br><br>**Sample size:** 150 participants	**Folliculitis in all skin areas after 1 week of treatment – treatment stopped**<br>**Once daily:** 1/75 participants (1.3%)<br>**Twice daily:** 0/75 participants (0%)<br>*(Difference between groups: p = 0.50 [a])*<br><br>**Folliculitis - treatment continued**<br>**Once daily:** 0/75 participants (0%)<br>**Twice daily:** 4/75 participants (5.3%)<br>*(Difference between groups: p = 0.14 [a])*<br><br>**Burning, itching and stinging sensations – treatment continued**<br>**Once daily:** 3/75 participants (4%)		

				**Twice daily:** 0/75 participants (0%)<br>*(Difference between groups: p = 0.20[a])*		
Tharp 1996 (110)<br><br>***(Green 2004*** *(107)* ***)***	RCT<br><br>*(4 weeks treatment)*<br><br>*(Quality using NHS CRD criteria: method for randomisation /allocation concealment unknown, adequate blinding, and no ITT used. (107))*	**Intervention:** Fluticasone propionate cream 0.05% once daily and vehicle once daily (n=77)<br><br>**Comparator:** Fluticasone propionate cream 0.05% twice daily (n=77)	**Severity:** moderate to severe<br><br>**Age:** children over 12 years and adults<br><br>**Sample size:** 154 participants	**Burning**<br>**Once daily**: 2/77 participants (3%)<br>**Twice daily:** 0/77 participants (0%)<br>*(Difference between groups: p = 0.30[a])*<br><br>**Dryness**<br>**Once daily:** 2/77 participants (3%)<br>**Twice daily:** 0/77 participants (0%)<br>*(Difference between groups: p = 0.30[a])*<br><br>**Pruritus**<br>**Once daily:** 0/77 participants (0%)<br>**Twice daily**: 1/77 participants (1%)<br>*(Difference between groups: p = 0.50[a])*<br><br>**Erythema**<br>**Once daily:** 0/77 participants (0%)<br>**Twice daily:** 0/77 participants(0%)<br><br>**Stinging**<br>**Once daily:** 0/77 participants (0%)<br>**Twice daily:** 1/77 participants (1%)<br>*(Difference between groups: p = 0.50[a])*<br><br>**Irritation**<br>**Once daily:** 0/77 participants (0%)<br>**Twice daily:** 1/77 participants (1%)		None of adverse events were serious or unexpected

For peer review only

				(Difference between groups: p = 0.50 [a]*)*		
Hoybye 1991 [111]<br><br>***(Green 2004*** [107]***)***	RCT<br><br>*((3 weeks treatment)*<br><br>*(Quality using NHS CRD criteria: method for randomisation /allocation concealment unknown, partial or inadequate blinding, and no ITT used.* [107]*)*<br><br>*(Moher 1995 quality checklist: method and concealment of randomisation unclear, single blind, ten dropouts/withdrawals, no ITT analysis,* [19]*)*	**Intervention:** Mometasone furoate in fatty cream base (Elocon) once daily (n=49)<br><br>**Comparator:** Hydrocortisone 17-butyrate in fatty cream base (Locoid) twice daily (n=45)	**Severity:** severity score at least 4.5/9<br><br>**Age:** adults (age 18 to 70)<br><br>**Sample size:** 94 participants	**<u>Treatment related side effects</u>**<br>Were only a few and similar in both groups. They included stinging, burning, itching, dryness, acne, folliculitis, and hair growth.<br><br>**<u>Skin thinning</u>**<br>No evidence		
Berth-Jones 2003 [112]<br><br>***(Green 2004*** [107]***)***<br><br>(This study is also included in the "Topical corticosteroids used proactively to prevent flares", as there is a second phase of the study when participants who have gained control of eczema are randomised to proactive treatment with topical corticosteroid or vehicle. This section only	RCT (four arms)<br><br>*(4 weeks treatment)*<br><br>*(Quality using NHS CRD criteria: adequate randomisation /allocation concealment, partial blinding, and ITT used.* [107]*)*	**Intervention:** Fluticasone propionate cream 0.05% once daily<br><br>N=95<br><br>**Intervention:** Fluticasone propionate ointment 0.005% once daily<br><br>N=100<br><br>**Comparator:** Fluticasone propionate cream 0.05% twice daily<br><br>N=91<br><br>**Comparator:** Fluticasone propionate ointment 0.005% twice daily<br><br>N=90	**Severity:** moderate to severe<br><br>**Age:** children and adults (12-65 years)<br><br>**Sample size:** 376 participants	**<u>Telangiectasia</u>**<br>**Once daily cream**: 0/95 participants (0%)<br>**Twice daily cream:** 1/91 participants (1%)<br>*(Difference between groups: p = 0.48* [a]*)*<br><br>**Once daily ointment:** 1/100 participants (1%)<br>**Twice daily ointment:** 0/90 participants (0%)<br>*(Difference between groups: p = 0.54* [a]*)*<br><br>**<u>Striae</u>**<br>**Once daily cream:** 0/95 participants (0%)<br>**Twice daily cream:** 0/91 participants (0%)<br>*(Difference between groups: n/a)*<br><br>**Once daily ointment:** 1/100 participants (1%)<br>**Twice daily ointment**: 0/90 participants (0%)		

For peer review only

includes safety data from the induction of remission phase).				*(Difference between groups: p = 0.54*[a]*)*<br>For the three events listed above: two of these patients had a previous history of skin changes, and therefore only one report was newly observed (group not specified in the review).		
Marchesi 1994 [113]<br>***(Green 2004*** [107]***)***	RCT<br>*(3 weeks treatment))*<br>*(Quality using NHS CRD criteria: method for randomisation /allocation concealment unknown, partial blinding, and no ITT used.* [107]*)*<br>*(Moher 1995 quality checklist: method and concealment of randomisation unclear, third-party blind evaluator, no dropouts/withdrawals* [19]*)*	**Intervention:** Mometasone furoate ointment 0.1% once daily (n=30)<br>**Comparator:** Betamethasone dipropionate ointment 0.05% twice daily (n=30)	**Severity:** at least moderate severity<br>**Age:** adults<br>**Sample size:** 60 participants	**<u>Telangiectasia of mild severity in last 2 weeks</u>**<br>**Once daily:** 4/30 participants (13.3%)<br>**Twice daily:** 5/30 participants (16.7%)<br>*(Difference between groups: p = 0.72*[a]*)*<br>**<u>Possible skin thinning (*“Loss of skin marks and reduced elasticity”*)</u>**<br>**Once daily:** 0/30 participants (0%)<br>**Twice daily:** 1/30 participants (3.3%)<br>*(Difference between groups: p = 0.50*[a]*)*<br>**<u>Local application site reactions</u>**<br>Did not occur	**<u>Systemic reactions</u>**<br>None – all patients checked for blood test and value varied within a very narrow range.	
**Moderate potency topical corticosteroids**						
Richelli 1990 [114]<br>***(Green 2004*** [107]***)***	RCT<br>(one week treatment<br>*(Quality using NHS CRD criteria: method for randomisation /allocation concealment unknown, inadequate blinding, and no ITT used.* [107]*)*<br>*(Moher 1995 quality checklist: method and concealment of*	**Intervention:** Clobetasone 17-butyrate 0.05% lotion once daily at 9pm (n=9)<br>**Comparator:** Clobetasone 17-butyrate 0.05% lotion twice daily at 8am and 3pm (n=13)<br>**Comparator:** Clobetasone 17-butyrate 0.05% lotion twice daily at 3pm and 8pm (n=8)	**Severity:** not specified in the review<br>**Age:** children<br>**Sample size:** 30 participants		**<u>HPA axis suppression</u>**<br>No significant difference in serum cortisol and ACTH levels before and after TCS administration in any of the three groups, or any differences between groups	Adverse effects not reported

For peer review only

	randomisation unclear, blinding unclear, ITT unclear [(19)])					
**How safe are topical corticosteroids when used proactively to prevent flares ("weekend therapy")?**						
**Study ID *(Systematic review*)***	**Study design and study duration *(Quality assessment)***	**Intervention and comparator**	**Participants**	**Cutaneous adverse events**	**Systemic adverse events**	**Unspecified adverse events**
**Potent topical corticosteroids versus vehicle**						
Berth-Jones 2003 [(112)]<br>***(Schmitt 2011*** [(115)]***; Tang 2014*** [(88)]***)***<br>(This study is also included under the comparison "Topical corticosteroids applied once a day compared with more frequent application" – where the induction of remission part of the study is included).	RCT<br>*(16 weeks maintenance)*<br>*(Cochrane risk of bias tool: low risk of selection (sequence generation), attrition and other biases, Unclear risk of selection (allocation concealment), bias from blinding and reporting bias.* [(115)]*)*	**Intervention:** Fluticasone propionate 0.005% ointment on two consecutive days per week, once daily (n=68)<br>**Intervention:** Fluticasone propionate 0.05% cream on two consecutive days per week, once daily (n=70)<br>**Comparator:** Vehicle cream or ointment (n=84)<br>**Comparator:** Vehicle ointment (n=73)	**Severity:** moderate to severe<br>**Age:** 12 to 65 years<br>**Sample size (maintenance phase):** 295 participants	**Skin thinning**<br>No new visual signs observed in either group during maintenance phase		
Glazenburg 2009 [(116)]<br>***(Schmitt 2011*** [(115)]***; Tang 2014*** [(88)]***)***	RCT<br>*(16 weeks maintenance)*<br>*(Cochrane risk of bias tool: low risk of selection (sequence generation), and attrition bias. Unclear risk of selection (allocation concealment), bias from blinding, reporting and other biases.* [(115)]*)*	**Intervention:** Fluticasone propionate 0.005% ointment (two consecutive days per week, once daily) (n=39)<br>**Comparator:** Vehicle (n=36)	**Severity:** moderate to severe<br>**Age:** children 4-10 years<br>**Sample size (maintenance phase):** 75 participants	**Skin thinning**<br>No evidence in either group<br>**Adverse events related to treatment (cutaneous)**<br>**Fluticasone:** 2 events (flexural hyperpigmentation, folliculitis, transient telangiectasia) (n=39)<br>**Vehicle:** 1 event (no further details reported) (n=36)<br>*(Difference between groups: p=0.61*[a]*)*	**Adrenal suppression**<br>No evidence in either group (measured by assessment of urinary overnight cortisol/creatinine ratios)<br>**Cancer**<br>No cases in either group	

Hanifin 2002 (117)<br>***(Schmitt 2011 (115))***<br>***(Fishbein 2019 (16))***	RCT<br>*(20 weeks maintenance)*<br>*(Cochrane risk of bias tool: low risk of attrition bias. Unclear risk of selection, bias from blinding and reporting bias. High risk of other biases (noncompliance) (115))*	**Intervention:** Fluticasone propionate 0.05% cream (once daily 4 days per week for 4 weeks, then once daily 2 days per week for 16 weeks) (n=229)<br>**Comparator:** Vehicle (n=119)	**Severity:** moderate to severe<br>**Age:** children and adults, 3 months to 65 years<br>**Sample size (maintenance phase):** 348 participants	**Adverse events related to treatment**<br>**Fluticasone:** 1/229 (one case of acne) (0.4%)<br>**Vehicle:** 0/119 (0%)<br>*(Difference between groups: p=0.78[a])*<br>**Skin thinning**<br>No evidence (by visual skin assessment)	**Possible adrenal suppression**<br>**Fluticasone:** 2/44* children (4.5%)<br>**Vehicle:** no evidence of adrenal suppression<br>*(measured by cosynthropin stimulation test)*<br>*One participant received 345 days of treatment and had a cortisol stimulation level after treatment of 17 ug/dL (normal was >=18 ug/dL). The other participant was treated for 280 days and had a cortisol stimulation level of 9 ug/dL. No follow up testing.<br>**Cancer**<br>No cases	
Van der Meer 1999 (118)<br>***(Schmitt 2011 (115))***	RCT<br>*(16 weeks maintenance)*<br>*(Cochrane risk of bias tool: low risk of attrition, and other biases. Unclear risk of selection, bias from blinding and reporting bias. (115))*<br>*((Moher 1995 quality checklist: method and concealment of randomisation unclear, double blinded, 17 withdrawals/dropouts, no ITT, only data up to first relapse analysed, (19))*	**Intervention:** Fluticasone propionate 0.005% ointment (2 consecutive days per week, once daily) (n=23)<br>**Comparator:** Vehicle (n=31)	**Severity:** moderate to severe<br>**Age:** children and adults, aged 15-50 years<br>**Sample size (maintenance phase):** 54 participants	**Skin thinning**<br>No evidence	**Adrenal suppression**<br>No change in geometric mean cortisol levels at baseline and end of maintenance<br>**Cancer**<br>No cases	
Peserico 2008 (119)<br>***(Schmitt 2011 (115))***	RCT<br>*(16 weeks maintenance)*<br>*(Cochrane risk of bias tool: high risk of selection bias (sequence generation). Low risk of attrition bias and bias from blinding. Unclear*	**Intervention:** Prednisolone aceponate 0.1% cream (two consecutive days per week, once daily) (n=112)<br>**Comparator:** Vehicle (n=108)	**Severity:** IGA≥ moderate<br>**Age:** children ≥12 years and adults<br>**Sample size (maintenance**	**Skin thinning**<br>No evidence	**Cancer**<br>No cases.	**Adverse events related to treatment**<br>Zero in both groups

For peer review only

	risk of selection (allocation concealment) reporting and other biases. [115]*)*		**phase):** 221 participants			
**How safe are topical corticosteroids used under occlusion?**						
**Study ID *(Systematic review*)***	**Study design and study duration *(Quality assessment)***	**Intervention and comparator**	**Participants**	**Cutaneous adverse events**	**Systemic adverse events**	**Unspecified adverse events**
**Very potent topical corticosteroid**						
Volden 1992 [120]<br>***(Braham 2010*** [121]***)***	Prospective (observational)<br>*(8-18 days treatment)*<br>*Risk of bias not assessed in any of the included systematic reviews.*	**Intervention:** Dry occlusion with clobetasol propionate lotion under dry occlusion (weekly) (n=48)<br>**Comparator:** No comparator	**Severity:** therapy resistant atopic eczema<br>**Age:** adults<br>**Sample size:** 48 participants	**<u>Mild folliculitis</u>**<br>2/48 participants (4%)<br>**<u>Skin thinning</u>**<br>None		
**Potent topical corticosteroids**						
Janmohamed 2014 [122]<br>***(Van Zuuren 2017*** [12]***)***	RCT<br>*(4 weeks treatment)*<br>*(Cochrane risk of bias tool: low risk of selection (sequence generation), attrition, reporting and other biases. Unclear risk of selection (allocation concealment), performance and detection bias.* [12]*)*	**Intervention:** wet wrap therapy with diluted mometasone furoate 0.1% ointment (n=19)<br>**Comparator:** 20% petrolatum in cetomacrogol combined with wet wrap (n=20)	**Severity:** severe<br>**Age:** children 6 months to 10 years (mean age 3.4 years)<br>**Sample size:** 39 participants	**<u>Folliculitis</u>**<br>**Mometasone under wet wrap:** 9/19 (47%)<br>**Emollient under wet wrap:** 2/20 (10%)<br>*(Difference between groups: p = 0.03* [a]*)*<br>**<u>Severe folliculitis</u>**<br>**Mometasone under wet wrap:** 1/19 (5.2%)<br>**Emollient under wet wrap:** 0/20 (0%)<br>*(Difference between groups: p = 0.47*)*<br>**<u>Secondary infected eczema</u>**<br>**Mometasone under wet wrap:** 0/19 (0%)<br>**Emollient under wet wrap**: 2/20 (10%)<br>*(Difference between groups: p= 0.30* [a]*)*<br>**<u>Beginning of decubitus</u>**<br>**Mometasone under wet wrap:** 0/19 (%)		

				**Emollient under wet wrap:** 2/20 (10%)<br>*(Difference between groups: p = 0.30[a])*<br><br>**<u>Decubitus</u>**<br>**Mometasone under wet wrap:** 2/19 (11%)<br>**Emollient under wet wrap:** 1/20 (5%)<br>*(Difference between groups: p = 0.53[a])*		
Schnopp 2002 [123]<br><br>***(Braham 2010*** [121]***)***	RCT, within-participant<br><br>*(5 days treatment)*<br><br>*(Cochrane risk of bias tool: unclear risk of selection bias, unclear risk from blinding.* [3]*)*	**Intervention:** wet wrap therapy with mometasone furoate 0.1%, twice daily<br><br>**Comparator:** wet wrap therapy with vehicle	**Severity:** exacerbated atopic eczema<br><br>**Age:** children aged 2 to 17 years (mean 7.2 years)<br><br>**Sample size:** 20 participants	**<u>Clinical skin infections</u>**<br>None in either group		
McGowan 2003 [124]<br><br>***(Devillers 2006*** [125]***)***	Prospective (observational)<br><br>*(Up to 14 days treatment)*<br><br>*Risk of bias not assessed in any of the included systematic reviews.*	**Intervention:** wet wrap therapy with diluted beclomethasone dipropionate, once daily (n=8)<br><br>**Comparator:** No comparator	**Severity:** not specified in the review<br><br>**Age:** children age 3.3 to 8.8 years<br><br>**Sample size:** 8 participants		**<u>Short term growth and bone turnover</u>**<br>No significant differences found between outcomes before and during a median treatment period of 12 weeks (range 2-18).<br><br>*(assessed safety with knemometry and urinary deoxypyridinoline crosslink excretion and early morning serum cortisol).*	
Wolkerstorfer 2000 [126]<br><br>***(Braham 2010*** [121]***)***<br><br>***(Fishbein 2019*** [16]***)***	Prospective, side to side (observational)<br><br>*(1 week treatment)*<br><br>*Risk of bias not assessed in any of the included systematic reviews.*	**Intervention:** wet wrap therapy with 10-50% dilution fluticasone propionate 0.05% cream (daily)<br><br>**Comparator:** emollient (only 2 participants) or no comparator	**Severity:** severe<br><br>**Age:** children 5 months to 13 years<br><br>**Sample size:** 18 participants	**<u>URI and/or folliculitis</u>**<br>**Fluticasone:** one third of participants<br><br>**<u>Furunculosis</u>**<br>**Fluticasone:** one case<br><br>**<u>Generalized folliculitis</u>**<br>One case in both emollient controls<br><br>**<u>Skin thinning</u>**<br>No cases	**<u>HPA axis suppression</u>**<br>"Nearly all" had decreased cortisol, 3 children were HPA suppressed (from Braham 2010 review).<br><br>Two patients having a 9am serum cortisol < 0.2 umol/L (0.09 and 0.03) after treatment for 7 days. Those participants used 957 ug/m^2 and 1125 ug/m^2 of steroid cream. There was no follow up	

For peer review only

					testing (from Fishbein 2019 review).	
Tang 2000 (127)<br><br>***(Braham 2010*** ***(121))***	Prospective (observational)<br><br>*("Few days" treatment)*<br><br>*Risk of bias not assessed in any of the included systematic reviews.*	**Intervention:** wet wrap therapy with 10% dilution mometasone furoate 0.1% (daily for 2 to 3 hours) (n=10)<br><br>**Comparator:** No comparator	**Severity:** review only reports 'facial eczema flare'<br><br>**Age:** children (mean 8.4 years)<br><br>**Sample size:** 10 participants	**Skin thinning**<br>None<br><br>**Infections**<br><br>None		
Goodyear 1991 (128)<br><br>***(Braham 2010*** ***(121))***	Prospective (observational)<br><br>*(2 to 5 days treatment)*<br><br>*Risk of bias not assessed in any of the included systematic reviews.*	**Intervention:** wet wrap therapy with 25% dilution betamethasone or hydrocortisone 1%, twice daily (potent or mild potency) (n=30)<br><br>**Comparator:** No comparator	**Severity:** acute erythrodermic eczema<br><br>**Age:** children aged 9 months to 2 years (mean 5.5 years)<br><br>**Sample size:** 30 participants	**Bacterial infections**<br>Some during follow up at home	**HPA axis suppression**<br>Transient low morning cortisol. During the follow up at home some adrenal suppression.	
Mallon 1994 (129)<br><br>***(Braham 2010*** ***(121))***	Prospective (observational)<br><br>*(up to 5 days treatment)*<br><br>*Risk of bias not assessed in any of the included systematic reviews.*	**Intervention:** wet wrap therapy with 10% dilution betamethasone 0.1% cream or hydrocortisone 0.5% cream(daily) (potent or mild potency) (n=21)<br><br>**Comparator:** No comparator	**Severity:** chronic severe eczema<br><br>**Age:** children aged 4 months to 10 years (5.1 years)<br><br>**Sample size:** 21 participants	No infections.		
Devillers 2002 (130)<br><br>***(Braham 2010*** ***(121))***	Retrospective side to side (observational)<br><br>*(1 week treatment)*<br><br>*Risk of bias not assessed in any of the included systematic reviews.*	**Intervention:** wet wrap therapy with **d**iluted fluticasone propionate 0.05% (daily re-wet every 2 to3 hours) (n=26)<br><br>**Comparator:** No comparator	**Severity:** refractory atopic eczema<br><br>**Age:** children (mean 3 years), adults (mean 30 years)<br><br>**Sample size:** 26 participants (14 children, 12 adults)	**Infections**<br>38% (n=10) had localized folliculitis, impetigo, pseudomonas, cellulitis, or purulent conjunctitivitis<br><br>**Skin thinning**<br>One case of striae in a patient taking inhaled steroids.	**HPA axis suppression**<br>Transient low morning cortisol, 12.5% with HPA suppression	
**Moderate potency topical corticosteroids**						
Foelster-Holst 2006 (131)	Within-participant RCT<br><br>*(48 to 72 hours treatment)*	**Intervention:** wet wrap therapy with prednicarbate ointment	**Severity:** local SCORAD >10, severe	Zero adverse events in either group. Did not observe severe cutaneous events.	Did not observe systemic events such as growth retardation or HPA suppression – but these	

For peer review only

(Gonzalez-Lopez 2017 [132]***)***	*(Cochrane risk of bias tool: unclear risk of selection and performance bias. High risk of performance bias. Unclear risk of attrition, reporting and other biases.* [132]*)*<br>*(Cochrane risk of bias tool: unclear risk of selection bias, high risk from no blinding.* [3]*)*	**Comparator:** Prednicarbate ointment	**Age:** children and adults, aged 6-63 years<br>**Sample size: 24** participants		events were not actively investigated.	
**Mild potency topical corticosteroid**						
Beattie 2004 [133]<br>***(Gonzalez-Lopez 2017*** [132]***)***	RCT<br>*(2 weeks treatment)*<br>*(Cochrane risk of bias tool: low risk of selection, reporting and other biases. High risk of performance and attrition bias. Unclear risk of detection bias. Gonzalez-Lopez 2017)*<br>*(Cochrane risk of bias tool: low risk of selection bias (sequence generation), unclear risk of selection bias (allocation concealment), unclear risk from blinding.* [3]*)*	**Intervention:** wet wrap therapy with hydrocortisone 1% twice daily then overnight the second week(n=10)<br>**Comparator:** Hydrocortisone 1% twice daily then daily (n=9)	**Severity:** moderate<br>**Age:** children < 5 years<br>**Sample size:** 19 participants	<u>**Cutaneous adverse events**</u><br>**Wet wrap therapy with hydrocortisone:** 2/10 participants (20%) (2 events were folliculitis, one child withdrew)<br>**Hydrocortisone only:** 0/9 participants (0%)<br>*(Difference between groups: (p=0.31* [a]*)*<br>Did not observe severe cutaneous events.	Did not observe systemic such as growth retardation or HPA suppression – but these events were not actively investigated.	
Hindley 2006 [134]<br>***(Gonzalez-Lopez 2017*** [132]***)***	RCT<br>*(4 weeks – not clear if treatment given for whole 4 weeks)*<br>*(Cochrane risk of bias tool: low risk of selection (random sequence generation) and reporting bias. Unclear risk of selection (allocation concealment),*	**Intervention:** wet wrap therapy with hydrocortisone 1% for 24 hours – could be reduced to 12 hours per day after first week (n=28)<br>**Comparator:** Hydrocortisone 1% twice day (n=22)	**Severity:** SCORAD >15, moderate to severe<br>**Age:** children 3 months to 5 years<br>**Sample size:** 50 participants	<u>**Cutaneous adverse events**</u><br>**Wet wrap therapy with hydrocortisone:** 5/28 participants (18%) (five cases of infected eczema)<br>**Hydrocortisone only:** 0/22 participants (0%)<br>*(Difference between groups: p = 0.14* [a]*)*	Did not observe systemic events such as growth retardation or HPA suppression – but these events were not actively investigated.	

For peer review only

	detection and other biases. High risk of performance and attrition bias. [(132)]*)*<br>*(Cochrane risk of bias tool: unclear risk of selection bias, low risk from blinding.* [(3)]*)*			Did not observe severe cutaneous events.		

Footnotes:
*This column refers to the systematic review in which the safety data was extract from. The trial may have also been included in other systematic reviews, but no additional safety data was reported.
Abbreviations: RCT = randomised controlled trial; TCS = topical corticosteroids; TCI = topical calcineurin inhibitors; HPA = hypothalamic pituitary adrenal, WWT = wet wrap therapy; RR = risk ratio; OR: odds ratios; 95% CI = 95% confidence interval; CHM = Chinese herbal medicine; IPA = Investigator's Global Assessment; BSA = Body Surface Area

[a] P value calculated by review authors using RevMan software.
[b] The P value calculated from Fisher's Exact Test was significant: 0.0298 (but in the overview, this study is included in a meta-analysis)
[c] The P value calculated from Fisher's Exact Test was significant: 0.0298 (but in the overview, this study is included in a meta-analysis)
[d] The P value calculated from Fisher's Exact Test was significant: 0.0352 (but in the overview, this study is included in a meta-analysis)
[e] The P value calculated from Fisher's Exact Test was significant: 0.0289 (but in the overview, this study is included in a meta-analysis)
[f] The P value calculated from Fisher's Exact Test was significant: 0.0267 (but in the overview, this study is included in a meta-analysis)

Where studies include "diluted" topical corticosteroids and we aren't sure how this affects the potency, we have put the topical corticosteroids in the potency classification based on the undiluted version.
The terms skin atrophy and skin thinning were both used in the included reviews – for consistently we have used skin thinning throughout.

1. Breneman D, Fleischer A, Foley V ea, editors. Clobetasol propionate 0.05% is equivalent as lotion or emollient cream in atopic dermatitis. Presented at the World Congress of Dermatology, 2002; and the European Academy of Dermatology and Venereology, 2003; 2003.
2. Feldman S. Relative efficacy and interchangeability ofvarious clobetasol propionate vehicles in the management of steroid-responsive dermatoses. *Curr Ther Res Clin Exp*. 2005;**66**(3):154-71.
3. Nankervis H, Thomas K, Delamere F, Barbarot S, Rogers N, Williams H. Scoping systematic review of treatments for eczema2016 2016/05/None.
4. Kimball AB, Gold MH, Zib B, Davis MW, Phase CPEFF, Group ICS. Clobetasol propionate emulsion formulation foam 0.05%: review of phase II open-label and phase III randomized controlled trials in steroid-responsive dermatoses in adults and adolescents. *J Am Acad Dermatol*. 2008;**59**(3):448-54. e1.
5. Frangos J, Kimball A. Clobetasol propionate emollient formulation foam in the treatment of corticosteroid-responsive dermatoses. *Expert Opin Pharmacother*. 2008;**9**(11):2001-7.
6. Rosso J, Bhambri S. Daily application of fluocinonide 0.1% cream for the treatment of atopic dermatitis. *J Clin Aesthet Dermatol*. 2009;**2**(9):24-32.
7. Barnes L, Kaya G, Rollason V. Topical Corticosteroid-Induced Skin Atrophy: A Comprehensive Review. *Drug Saf*. 2015;**38**(5):493-509.
8. Olux-e (dobetasol propionate) foam pw. Steiffel Corporation, Palo Alto, CA; [Available from: www.olux-e.com.
9. Wood Heickman L, Davallow Ghajar L, Conaway M, Rogol A. Evaluation of Hypothalamic-Pituitary-Adrenal Axis Suppression following Cutaneous Use of Topical Corticosteroids in Children: A Meta-Analysis. *Horm Res Paediatr*. 2018;**89**(6):389-96.
10. Herz G, Yawalkar S, Weirich E. Evaluation of halometasone ointment in the treatment of paediatric patients with chronic eczematous dermatoses. *J Int Med Res*. 1983;**11 Suppl 1**:21-5.
11. Sugarman J, Parish L. Efficacy of a lipid-based barrier repair formulation in moderate-to-severe pediatric atopic dermatitis. *J Drugs Dermatol*. 2009;**8**(12):1106-11.

For peer review only - http://bmjopen.bmj.com/site/about/guidelines.xhtml

12. van Zuuren EJ, Fedorowicz Z, Christensen R, Lavrijsen AP, Arents BW. Emollients and moisturisers for eczema. Cochrane Database Syst Rev [Internet]. 2017; (2). Available from: http://onlinelibrary.wiley.com/doi/10.1002/14651858.CD012119.pub2/abstract.

13. Griffiths C, Van Leent E, Gilbert M, Traulsen J, Cipamyflline Study Group. Randomized comparison of the type 4 phosphodiesterase inhibitor cipamfylline cream, cream vehicle and hydrocortisone 17-butyrate cream for the treatment of atopic dermatitis. *Br J Dermatol*. 2002;**147**(2):299-307.

14. Eichenfield L, Miller B, Cutivate Lotion Study Group. Two randomized, double-blind, placebo-controlled studies of fluticasone propionate lotion 0.05% for the treatment of atopic dermatitis in subjects from 3 months of age. *J Am Acad Dermatol*. 2006;**54**(4):715-7.

15. Wu S, Chen X, Liu B, Wu H, Dong L. Efficacy and safety of 15(R/S)-methyl-lipoxin A4 in topical treatment of infantile eczema. *Br J Dermatol*. 2013;**168**(1):172-8.

16. Fishbein AB, Mueller K, Lor J, Smith P, Paller AS, Kaat A. Systematic Review and Meta-analysis Comparing Topical Corticosteroids With Vehicle/Moisturizer in Childhood Atopic Dermatitis. *J Pediatr Nurs*. 2019;**47**:36-43.

17. Pellanda C, Weber M, Bircher A, Surber C. Low-dose triamcinolone acetonide in the phytocosmetic lichtena reduces inflammation in mild to moderate atopic dermatitis. *Dermatology*. 2005;**211**(4):338-40.

18. Lebwohl M. Efficacy and safety of fluticasone propionate ointment, 0.005%, in the treatment of eczema. *Cutis*. 1996;**57**(Suppl 2):62-8.

19. Hoare C, Li Wan Po A, Williams H. Systematic review of treatments for atopic eczema. *Health Technol Assess*. 2000;**4**(37):1-191.

20. Lebwohl M. A comparison of once-daily application of mometasone furoate 0.1% cream compared with twice-daily hydrocortisone valerate 0.2% cream in pediatric atopic dermatitis patients who failed to respond to hydrocortisone. *Int J Dermatol*. 1999;**38**(8):604-6.

21. Abramovits W, Oquendo M. Hydrocortisone butyrate 0.1% cream (proprietary lipid rich cream vehicle) does not significantly suppress hypothalamic-pituitary-adrenal axis and is effective in pediatric patients 3 months and older with extensive atopic dermatitis. *Skinmed*. 2010;**8**(3):150-4.

22. Matheson R, Kempers S, Breneman D, Draelos Z, Johnson CE, Loss R, et al. Hydrocortisone butyrate 0.1% lotion in the treatment of atopic dermatitis in pediatric subjects. *J Drugs Dermatol*. 2008;**7**(3):266-71.

23. Friedlander S, Hebert A, Allen D. Safety of fluticasone propionate cream 0.05% for the treatment of severe and extensive atopic dermatitis in children as young as 3 months. *J Am Acad Dermatol*. 2002;**46**(3):387-93.

24. Callen J, Chamlin S, Eichenfield L, Ellis C, Girardi M, Goldfarb M, et al. A systematic review of the safety of topical therapies for atopic dermatitis. *Br J Dermatol*. 2007;**156**(2):203-21.

25. Eichenfield L, Ellis CN, Fivenson D, Hebert AA, Dromgoole S, Piacquadio D. Evaluation of adrenal suppression of a lipid enhanced, topical emollient cream formulation of hydrocortisone butyrate 0.1% in treating children with atopic dermatitis. *Pediatr Dermatol*. 2007;**24**(1):81-4.

26. Hebert AA, Friedlander SF, Allen DB. Topical fluticasone propionate lotion does not cause HPA axis suppression. *J Pediatr*. 2006;**149**(3):378-82.

27. De Belilovsky C, Roo-Rodriguez E, Baudouin C, Menu F, Chadoutaud B, Msika P. Natural peroxisome proliferator-activated receptor-alpha agonist cream demonstrates similar therapeutic response to topical steroids in atopic dermatitis. *J Dermatolog Treat*. 2011;**22**(6):359-65.

28. Rosenthal A. Clocortolone pivalate: a paired comparison clinical trial of a new topical steroid in eczema/atopic dermatitis. *Cutis*. 1980;**25**(1):96-8.

29. Singh S, Mann B. Clinical utility of clocortolone pivalate for the treatment of corticosteroid-responsive skin disorders: A systematic review. *Clin Cosmet Investig Dermatol*. 2012;**5**:61-8.

30. Binder R. Clinical study of clocortolone pivalate in the treatment of eczema/atopic dermatitis. *Curr Ther Res Clin Exp*. 1977;**21**(6):796-801.

31. Rauschkolb EW, Bender SH, Ebling JK, McCormick GE, Rosenthal AL. Low concentration halcinonide cream in the topical management of atopic dermatitis in pediatric patients. *Cutis*. 1981;**27**(1):105-7.

32. Nolting S GH, Griinder K. Therapie des atopischen ekzems im kindesalter: ergebnisse einer kontrollierten studie mit den topischen kortikosteroidzubereitungen mometason und prednicarbaL *Der Kinderarzt*. 1991;**22**(10):1690-3.

33. de Tiedra A, Mercadal J, Lozano R. Prednicarbate versus fluocortin for inflammatory dermatoses: A cost-effectiveness study. *PharmacoEconomics*. 1997;**12**(2 Pt 1):193-208.

34. Rampini E. Methylprenisolone aceponate (MPA)* - Use and clinical experience in children. *J Dermatolog Treat*. 1992;**3 (SUPPL. 2)**(Suppl 2 CNO - CN-00421335):27-9.
35. Camacho F, García B, Diaz J, Aguirre A, Arnau C, Garcia J, et al. Double-blind intraindividual comparative trial between prednicarbate versus fluocortolone for the treatment of atopic dermatitis [Ensayo comparativo intraindividual y doble ciego entre prednicarbato y f1uocortolona en el tratamiento de la dermatitis atapica]. Actas Dermo-Sifiliograficas [Internet]. 1996; 87:[59-63 pp.]. Available from: http://cochranelibrary-wiley.com/o/cochrane/clcentral/articles/811/CN-00662811/frame.html.
36. Gimenez Camarasa JM SEFC, editor Prednicarbato, un nuevo corticoide tapico, en comparacian con f1uocinolona en el tratamiento de la dermatitis atapica. XXIII Congreso Nacional de la Academia Espanola de Dermatologia y Venereologia; 1994 Jun 16-18; Madrid.
37. Moshang T. Prednicarbate emollient cream 0.1% in pediatric patients with atopic dermatitis. *Cutis*. 2001;**68**(1):63-9.
38. Conde J, Kaur M, Fleischer Jr A, Tusa M, Camacho F, Feldman S. Adherence to clocortolone pivalate cream 0.1% in a pediatric population with atopic dermatitis. *Cutis*. 2008;**81**(5):435-41.
39. Crespi H. Topical corticosteroid therapy for children: alclometasone dipropionate cream 0.05%. *Clin Ther*. 1986;**8**(2):203-10.
40. Udompataikul M, Srisatwaja W. Comparative trial of moisturizer containing licochalcone A vs. hydrocortisone lotion in the treatment of childhood atopic dermatitis: a pilot study. *J Eur Acad Dermatol Venereol*. 2011;**25**(6):660-5.
41. Hebert A, Cook-Bolden F, Basu S, Calvarese B, Trancik R, Desonide Hydrogel Study Group. Safety and efficacy of desonide hydrogel 0.05% in pediatric subjects with atopic dermatitis. *J Drugs Dermatol*. 2007;**6**(2):175-81.
42. Udompataikul M, Limpa-o-vart D. Comparative trial of 5% dexpanthenol in water-in-oil formulation with 1% hydrocortisone ointment in the treatment of childhood atopic dermatitis: a pilot study. *J Drugs Dermatol*. 2012;**11**(3):366-74.
43. Wananukul S, Chatproedprai S, Chunharas A, Limpongsanuruk W, Singalavanija S, Nitiyarom R, et al. Randomized, double-blind, split-side, comparison study of moisturizer containing licochalcone A and 1% hydrocortisone in the treatment of childhood atopic dermatitis. *J Med Assoc Thai [Chotmaihet thangphaet]*. 2013;**96**(9):1135-42.
44. Jirabundansuk P, Ophaswongse S, Udompataikul M. Comparative trial of moisturizer containing spent grain wax, Butyrospermum parkii extract, Argania spinosa kernel oil vs. 1% hydrocortisone cream in the treatment of childhood atopic dermatitis. *J Med Assoc Thai [Chotmaihet thangphaet]*. 2014;**97**(8):820-6.
45. Dolle S, Hoser D, Rasche C, Loddenkemper C, Maurer M, Zuberbier T, et al. Long-term reduction in local inflammation by a lipid raft molecule in atopic dermatitis. *Allergy*. 2010;**65**(9):1158-65.
46. Patzelt-Wenczler R, Ponce-Poschl E. Proof of efficacy of Kamillosan(R) cream in atopic eczema. *Eur J Med Res*. 2000;**5**(4):171-5.
47. Paller A, Nimmagadda S, Schachner L, Mallory S, Kahn T, Willis I, et al. Fluocinolone acetonide 0.01% in peanut oil: therapy for childhood atopic dermatitis, even in patients who are peanut sensitive. *J Am Acad Dermatol*. 2003;**48**(4):569-77.
48. Patel L, Clayton P, Addison G, Price D, David T. Adrenal function following topical steroid treatment in children with atopic dermatitis. *Br J Dermatol*. 1995;**132**(6):950-5.
49. Dohil MA, Alvarez-Connelly E, Eichenfield LF. Fluocinolone acetonide 0.01% in peanut oil: safety and efficacy data in the treatment of childhood atopic dermatitis in infants as young as 3 months of age. *Pediatr Dermatol*. 2009;**26**(3):262-8.
50. Eichenfield LF, Basu S, Calvarese B, Trancik RJ. Effect of desonide hydrogel 0.05% on the hypothalamic-pituitary-adrenal axis in pediatric subjects with moderate to severe atopic dermatitis. *Pediatr Dermatol*. 2007;**24**(3):289-95.
51. Hebert AA, Desonide Foam Phase III Clinical Study Group. Desonide foam 0.05%: safety in children as young as 3 months. *J Am Acad Dermatol*. 2008;**59**(2):334-40.
52. Bieber T, Vick K, Folster-Holst R, Belloni-Fortina A, Stadtler G, Worm M, et al. Efficacy and safety of methylprednisolone aceponate ointment 0.1% compared to tacrolimus 0.03% in children and adolescents with an acute flare of severe atopic dermatitis. *Allergy*. 2007;**62**(2):184-9.
53. Broeders J, Ahmed Ali U, Fischer G. Systematic review and meta-analysis of randomized clinical trials (RCTs) comparing topical calcineurin inhibitors with topical corticosteroids for atopic dermatitis: A 15-year experience. *J Am Acad Dermatol*. 2016;**75**(2):410-9.e3.

54. Cury Martins J, Martins C, Aoki V, Gois AF, Ishii HA, da SEM. Topical tacrolimus for atopic dermatitis. Cochrane Database Syst Rev [Internet]. 2015; (7). Available from: http://onlinelibrary.wiley.com/doi/10.1002/14651858.CD009864.pub2/abstract.
55. Doss N, Kamoun M, Dubertret L, Cambazard F, Remitz A, Lahfa M, et al. Efficacy of tacrolimus 0.03% ointment as second-line treatment for children with moderate-to-severe atopic dermatitis: evidence from a randomized, double-blind non-inferiority trial vs. fluticasone 0.005% ointment. *Pediatr Allergy Immunol*. 2010;**21**(2 Pt 1):321-9.
56. Dong Y, Zeng W, Li W, Ma H, Zheng W. Efficacy and safety of topical tacrolimus for childhood atopic dermatitis; a meta-analysis. [Chinese]. *J Clin Dermatol*. 2017;**46**(4):239-42.
57. Doss N, Reitamo S, Dubertret L, Fekete G, Kamoun M, Lahfa M, et al. Superiority of tacrolimus 0.1% ointment compared with fluticasone 0.005% in adults with moderate to severe atopic dermatitis of the face: results from a randomized, double-blind trial. *Br J Dermatol*. 2009;**161**(2):427-34.
58. Luger T, Van Leent E, Graeber M, Hedgecock S, Thurston M, Kandra A, et al. SDZ ASM 981: an emerging safe and effective treatment for atopic dermatitis. *Br J Dermatol*. 2001;**144**(4):788-94.
59. Ashcroft D, Dimmock P, Garside R, Stein K, Williams H. Efficacy and tolerability of topical pimecrolimus and tacrolimus in the treatment of atopic dermatitis: Meta-analysis of randomised controlled trials. *BMJ* 2005;**330**(7490):516-22.
60. Ashcroft DM, Chen L-C, Garside R, Stein K, Williams HC. Topical pimecrolimus for eczema Cochrane Database Syst Rev [Internet]. 2007; (4). Available from: http://onlinelibrary.wiley.com/doi/10.1002/14651858.CD005500.pub2/abstract.
61. Luger T, Lahfa M, Folster-Holst R, Gulliver W, Allen R, Molloy S, et al. Long-term safety and tolerability of pimecrolimus cream 1% and topical corticosteroids in adults with moderate to severe atopic dermatitis. *J Dermatolog Treat*. 2004;**15**(3):169-78.
62. Mandelin J, Remitz A, Virtanen H, Reitamo S. One-year treatment with 0.1% tacrolimus ointment versus a corticosteroid regimen in adults with moderate to severe atopic dermatitis: A randomized, double-blind, comparative trial. *Acta Derm Venereol*. 2010;**90**(2):170-4.
63. Reitamo S, Rustin M, Ruzicka T, Cambazard F, Kalimo K, Friedmann P, et al. Efficacy and safety of tacrolimus ointment compared with that of hydrocortisone butyrate ointment in adult patients with atopic dermatitis. *J Allergy Clin Immunol*. 2002;**109**(3):547-55.
64. Iskedjian M, Piwko C, Shear N, Langley R, Einarson T. Topical calcineurin inhibitors in the treatment of atopic dermatitis: A meta-analysis of current evidence. *Am J Clin Dermatol*. 2004;**5**(4):267-79.
65. Penaloza Hidalgo B, Knight T, Burls A. A systematic review of effectiveness and cost effectiveness of tacrolimus ointment for topical treatment of atopic dermatitis in adults and children Health Technol Assess [Internet]. 2004; (4):[81 p.].
66. Reitamo S, Ortonne J, Sand C, Cambazard F, Bieber T, Folster-Holst R, et al. A multicentre, randomized, double-blind, controlled study of long-term treatment with 0.1% tacrolimus ointment in adults with moderate to severe atopic dermatitis. *Br J Dermatol*. 2005;**152**(6):1282-9.
67. Gradman J, Wolthers O. Short-term growth in children with eczema during treatment with topical mometasone furoate and tacrolimus. *Acta paediatrica*. 2007;**96**(8):1233-7.
68. Svensson A, Chambers C, Gånemo A, Mitchell S. A systematic review of tacrolimus ointment compared with corticosteroids in the treatment of atopic dermatitis. *Curr Med Res Opin*. 2011;**27**(7):1395-406.
69. FK506 Ointment Study Group. Phase III comparative study of FK506 ointment vs betamethasone valerate ointment in atopic dermatitis (trunk/extremities) [in Japanese]. *Nishinihon J Dermatol*. 1997;**59**:870-9.
70. Hofman T, Cranswick N, Kuna P, Boznanski A, Latos T, Gold M, et al. Tacrolimus ointment does not affect the immediate response to vaccination, the generation of immune memory, or humoral and cell-mediated immunity in children. *Arch Dis Child*. 2006;**91**(11):905-10.
71. Siegfried E, Jaworski J, Kaiser J, Hebert A. Systematic review of published trials: Long-term safety of topical corticosteroids and topical calcineurin inhibitors in pediatric patients with atopic dermatitis. *BMC Pediatr*. 2016;**16** (75).
72. Sikder M, Al Mamun S, Khan R, Chowdhury A, Khan H, Hoque M. Topical 0.03% tacrolimus ointment, 0.05% clobetasone butyrate cream alone and their combination in older children with atopic dermatitis - An open randomized comparative study. *J Pak Assoc Dermatol*. 2005;**15**(4):304-12.

For peer review only

73. Torok H, Maas-Irslinger R, Slayton R. Clocortolone pivalate cream 0.1% used concomitantly with tacrolimus ointment 0.1% in atopic dermatitis. *Cutis*. 2003;**72**(2):161-6.
74. Sigurgeirsson B, Boznanski A, Todd G, Vertruyen A, Schuttelaar M, Zhu X, et al. Safety and efficacy of pimecrolimus in atopic dermatitis: a 5-year randomized trial. *Pediatrics* 2015;**135**(4):597-606.
75. Reitamo S, Van Leent E, Ho V, Harper J, Ruzicka T, Kalimo K, et al. Efficacy and safety of tacrolimus ointment compared with that of hydrocortisone acetate ointment in children with atopic dermatitis. *J Allergy Clin Immunol*. 2002;**109**(3):539-46.
76. Reitamo S, Harper J, Bos J, Cambazard F, Bruijnzeel-Koomen C, Valk P, et al. 0.03% Tacrolimus ointment applied once or twice daily is more efficacious than 1% hydrocortisone acetate in children with moderate to severe atopic dermatitis: results of a randomized double-blind controlled trial. *Br J Dermatol*. 2004;**150**(3):554-62.
77. Gutgesell C, Jung T, Reich K, Junghans V, Bohn M, Neumann C. Double-blind hydrocortisone-controlled tacrolimus ointment for atopic dermatitis.(Abstract 1255). *J Invest Dermatol*. 1998;**110**(4):681.
78. Arellano F, Wentworth C, Arana A, Fernandez C, Paul C. Risk of lymphoma following exposure to calcineurin inhibitors and topical steroids in patients with atopic dermatitis. *J Invest Dermatol*. 2007;**127**(4):808-16.
79. Arellano F, Arana A, Wentworth C, Fernandez-Vidaurre C, Schlienger R, Conde E. Lymphoma among patients with atopic dermatitis and/or treated with topical immunosuppressants in the United Kingdom. *J Allergy Clin Immunol*. 2009;**123**(5):1111–6.
80. Schneeweiss S, Doherty M, Zhu S, Funch D, Schlienger R, Fernandez-Vidaurre C, et al. Topical treatments with pimecrolimus, tacrolimus and medium- to high-potency corticosteroids, and risk of lymphoma. *Dermatology*. 2009;**219**(1):7-21.
81. Reitamo S WA, Schopf E, Perrot JL, Marks R, Ruzicka T, et al. Safety and efficacy of 1 year of tacrolimus ointment monotherapy in adults with atopic dermatitis.The European Tacrolimus Ointment Study Group. *Arch Dermatol*. 2000;**136**(8):999-1006.
82. Ulrich R, Andresen I. Double-blind comparative trial involving 0.5% halomethasone (Sicorten (R)) cream versus 0.25% prednicarbate cream in patients with acute episodes of atopic dermatitis [Behandlung akuter schube der atopischen dermatitis. Doppelblinde vergleichsstudie mit 0,05%-halometason-creme versus 0,25%-prednicarbat-creme]. *MMW Fortschr Med*. 1991;**109**(36):49-50+3-4.
83. Smitt J, Winterberg D, Oosting J. Treatment of atopic dermatitis with topical corticosteroids in children. Efficacy and systemic effects of triamcinolone acetonide and alclometasone dipropionate. *Eur J Dermatol*. 1993;**3**(7):549-52.
84. Lebrun-Vignes B, Legrain V, Amoric J, Taieb A. [Comparative study of efficacy and effect on plasma cortisol levels of micronised desonide cream 0.1 p. 100 versus betamethasone dipropionate cream 0.05 p. 100 In the treatment of childhood atopic dermatitis] (French). *Ann Dermatol Venereol*. 2000;**127**(6-7):590-5.
85. Prado de Oliveira Z, Cuce L, Arnone M. Comparative evaluation of efficacy, tolerability and safety of 0.1% topical momethasone furoate and 0.05% desonide in the treatment of childhood atopic dermatitis. *An Bras Dermatol*. 2002;**77**:25-33.
86. Hanifin J. The role of topical steroids in the treatment of allergic skin disease. Towards Evolution of Allergic Skin Disease Management. Hong Kong: Excerpta Medica; 1996. p. 1-2.
87. Kirkup M, Birchall N, Weinberg E, Helm K, Kennedy C. Acute and maintenance treatment of atopic dermatitis in children - two comparative studies with fluticasone propionate (0.05%) cream. *J Dermatolog Treat*. 2003;**14**(3):141-8.
88. Tang T, Bieber T, Williams H. Are the concepts of induction of remission and treatment of subclinical inflammation in atopic dermatitis clinically useful? *J Allergy Clin Immunol*. 2014;**133**(6):1615-25.e1.
89. Kuokkanen K, Sillantaka I. Alclometasone dipropionate 0.05% vs hydrocortisone 1.0%: potential to induce cutaneous atrophy in children. *Clin Ther*. 1987;**9**(2):223-31.
90. Ellison JA, Patel L, Ray DW, David TJ, Clayton PE. Hypothalamic-pituitary-adrenal function and glucocorticoid sensitivity in atopic dermatitis. *Pediatrics*. 2000;**105**(4 Pt 1):794-9.

For peer review only

91. Eichenfield L, Tom W, Berger T, Krol A, Paller A, Schwarzenberger K, et al. Guidelines of care for the management of atopic dermatitis: Section 2. Management and treatment of atopic dermatitis with topical therapies. *J Am Acad Dermatol*. 2014;**71**(1):116-32.
92. Kristmundsdottir F, David T. Growth impairment in children with atopic eczema. *Journal of the Royal Society of Medicine*. 1987;**80**(1):9-12.
93. Patel L, Clayton P, Jenney M, Ferguson J, David T. Adult height in patients with childhood onset atopic dermatitis. *Arch Dis Child*. 1997;**76**(6):505-7.
94. Patel L, Clayton P, Addison G, Price D, David T. Linear growth in prepubertal children with atopic dermatitis. *Arch Dis Child*. 1998;**79**(2):169-72.
95. Aliaga A, Rodriguez M, Armijo M, Bravo J, Avila AL, Mascaro JM, et al. Double-blind study of prednicarbate versus fluocortin butyl ester in atopic dermatitis. *Int J Dermatol*. 1996;**35**(2):131-2.
96. Lucky A, Grote G, Williams J, Tuley M, Czernielewski J, Dolak T, et al. Effect of desonide ointment, 0.05%, on the hypothalamic-pituitary-adrenal axis of children with atopic dermatitis. *Cutis*. 1997;**59**(3):151-3.
97. Jorizzo J, Levy M, Lucky A, Shavin J, Goldberg G, Dunlap F, et al. Multicenter trial for long-term safety and efficacy comparison of 0.05% desonide and 1% hydrocortisone ointments in the treatment of atopic dermatitis in pediatric patients. *J Am Acad Dermatol*. 1995;**33**(1):74-7.
98. Froeschl B, Arts D, Leopold C. Corticosteroid therapy in the treatment of pediatric patients with atopic dermatitis (Structured abstract). Health Technol Assess [Internet]. 2007; (4). Available from: http://onlinelibrary.wiley.com/o/cochrane/clhta/articles/HTA-32008100208/frame.html.
99. Huang Z, Chen P, Wei W. Observation of efficacy of Chushi Zhiyang cream for atopic dermatitis. *Mod J Integr Trad Chin West Med*. 2010;**19**:2647–8.
100. Gu S, Yang AW, Xue CC, Li CG, Pang C, Zhang W, et al. Chinese herbal medicine for atopic eczema. Cochrane Database Syst Rev [Internet]. 2013; (9). Available from: http://onlinelibrary.wiley.com/doi/10.1002/14651858.CD008642.pub2/abstract.
101. Gu S, Yang A, Li C, Lu C, Xue C. Topical application of Chinese herbal medicine for atopic eczema: A systematic review with a meta-analysis. *Dermatology*. 2014;**228**(4):294-302.
102. Chen H. Clinical observation of Huanglian Qingdai cream for infantile eczema

J Sichuan Trad Chin Med. 2011;**29**:88–9.
103. Dong M. Efficacy of external use of Jingfang mixture in the treatment of children with eczema. *Med J Qilu*. 2012;**27**:75–8.
104. Xu XC GY. Clinical observation of Kouqiang Xiaoyan powder for infantile eczema. *Strait Pharm J*. 2012;**24**:210.
105. Schlessinger J, Miller B, Gilbert R, Plott R, Vanos Study Group. An open-label adrenal suppression study of 0.1% fluocinonide cream in pediatric patients with atopic dermatitis. *Arch Dermatol*. 2006;**142**(12):1568-72.
106. Bleehen S, Chu A, Hamann I, Holden C, Hunter J, Marks R. Fluticasone propionate 0.05% cream in the treatment of atopic eczema: a multicentre study comparing once-daily treatment and once-daily vehicle cream application versus twice-daily treatment. *Br J Dermatol*. 1995;**133**(4):592-7.
107. Green C, Colquitt J, Kirby J, Davidson P, Payne E. Clinical and cost-effectiveness of once-daily versus more frequent use of same potency topical corticosteroids for atopic eczema: a systematic review and economic evaluation. *Health Technol Assess (Winchester, England)*. 2004;**8**(47):iii,iv, 1-120.
108. (GSK) G. A four week multicentre, double blind study to compare safety and efficacy with an OD and BD administration of fluticasone propionate 0.005% ointment in the treatment of atopic eczema. 1995. Contract No.: Report 135L,Protocol No. GL/FLT/002.
109. Koopmans B, Lasthein Andersen B, Mork N, Austad J, Suhonen R, Roders G. Multicentre randomized double-blind study of Locoid Lipocream fatty cream twice daily versus Locoid Lipocream once daily and Locobase once daily. *J Dermatolog Treat*. 1995;**6**(2):103-6.
110. Tharp M. A comparison of twice-daily and once-daily administration of fluticasone propionate cream, 0.05%, in the treatment of eczema. *Cutis*. 1996;**57**(Suppl 2):19-26.
111. Hoybye S, Balk Moller S, De Cunha Bang F, Ottevanger V, Veien N. Continuous and intermittent treatment of atopic dermatitis in adults with mometasone furoate versus hydrocortisone 17-butyrate. *Curr Ther Res Clin Exp*. 1991;**50**(1):67-72.
112. Berth-Jones J, Damstra R, Golsch S, Livden J, Van Hooteghem O, Allegra F, et al. Twice weekly fluticasone propionate added to emollient maintenance treatment to reduce risk of relapse in atopic dermatitis: randomised, double blind, parallel group study. *BMJ* 2003;**326**(7403):1367-9.

For peer review only - http://bmjopen.bmj.com/site/about/guidelines.xhtml

113. Marchesi E, Rozzoni M, Pini P, Cainelli T. Comparative study of mometasone furoate and betamethasone dipropionate in the treatment of atopic dermatitis. *G Ital Dermatol Venereol*. 1994;**129**(1-2):IX-XII.
114. Richelli C, Piacentini G, Sette L, Bonizzato M, Andreoli A, Boner A. Clinical efficacy and tolerability of clobetasone 17-butyrate 0.5% lotion in children with atopic dermatitis. *Curr Ther Res Clin Exp*. 1990;**47**(3):413-7.
115. Schmitt J, Von Kobyletzki L, Svensson A, Apfelbacher C. Efficacy and tolerability of proactive treatment with topical corticosteroids and calcineurin inhibitors for atopic eczema: Systematic review and meta-analysis of randomized controlled trials. *Br J Dermatol*. 2011;**164**(2):415-28.
116. Glazenburg E, Wolkerstorfer A, Gerretsen A, Mulder P, Oranje A. Efficacy and safety of fluticasone propionate 0.005% ointment in the long-term maintenance treatment of children with atopic dermatitis: differences between boys and girls? *Pediatr Allergy Immunol*. 2009;**20**(1):59-66.
117. Hanifin J, Gupta A, Rajagopalan R. Intermittent dosing of fluticasone propionate cream for reducing the risk of relapse in atopic dermatitis patients. *Br J Dermatol*. 2002;**147**(3):528-37.
118. Van Der Meer J, Glazenburg E, Mulder P, Eggink H, Coenraads P. The management of moderate to severe atopic dermatitis in adults with topical fluticasone propionate. The Netherlands Adult Atopic DermatitisStudy Group. *Br J Dermatol*. 1999;**140**(6):1114-21.
119. Peserico A, Stadtler G, Sebastian M, Fernandez R, Vick K, Bieber T. Reduction of relapses of atopic dermatitis with methylprednisolone aceponate cream twice weekly in addition to maintenance treatment with emollient: a multicentre, randomized, double-blind, controlled study. *Br J Dermatol*. 2008;**158**(4):801-7.
120. Volden G. Successful treatment of therapy-resistant atopic dermatitis with clobetasol propionate and a hydrocolloid occlusive dressing. *Acta Derm Venereol Suppl (Stockh)*. 1992;**176**:126-8.
121. Braham S, Pugashetti R, Koo J, Maibach H. Occlusive therapy in atopic dermatitis: overview. *J Dermatolog Treat*. 2010;**21**(2):62-72.
122. Janmohamed S, Oranje A, Devillers A, Rizopoulos D, van Praag M, Van Gysel D, et al. The proactive wet-wrap method with diluted corticosteroids versus emollients in children with atopic dermatitis: a prospective, randomized, double-blind, placebo-controlled trial. *J Am Acad Dermatol*. 2014;**70**(6):1076-82.
123. Schnopp C, Holtmann C, Stock S, Remling R, Folster-Holst R, Ring J, et al. Topical steroids under wet-wrap dressings in atopic dermatitis - A vehicle-controlled trial. *Dermatology*. 2002;**204**(1):56-9.
124. McGowan R, Tucker P, Joseph D, Wallace A, Hughes I, Burrows N, et al. Short-term growth and bone turnover in children undergoing occlusive steroid ('wet-wrap') dressings for treatment of atopic eczema. *J Dermatolog Treat*. 2003;**14**(3):149-52.
125. Devillers A, Oranje A. Efficacy and safety of 'wet-wrap' dressings as an intervention treatment in children with severe and/or refractory atopic dermatitis: a critical review of the literature. *Br J Dermatol*. 2006;**154**(4):579-85.
126. Wolkerstorfer A, Visser R, De Waard van der Spek F, Mulder P, Oranje A. Efficacy and safety of wet-wrap dressings in children with severe atopic dermatitis: influence of corticosteroid dilution. *Br J Dermatol*. 2000;**143**(5):999-1004.
127. Tang W. Diluted steroid facial wet wraps for childhood atopic eczema. *Dermatology*. 2000;**200**(4):338-9.
128. Goodyear H, Spowart K, Harper J. 'Wet-wrap'dressings for the treatment of atopic eczema in children. *Br J Dermatol*. 1991;**125**(6):604.
129. Mallon E, Powell S, Bridgman A. 'Wet-wrap'dressings for the treatment of atopic eczema in the community. *J Dermatolog Treat*. 1994;**5**(2):97-8.
130. Devillers A, de Waard-van der Spek F, Mulder P, Oranje A. Treatment of refractory atopic dermatitis using 'wet-wrap'dressings and diluted corticosteroids: results of standardized treatment in both children and adults. *Dermatology*. 2002;**204**(1):50-5.
131. Foelster-Holst R, Nagel F, Zoellner P, Spaeth D. Efficacy of crisis intervention treatment with topical corticosteroid prednicarbat with and without partial wet-wrap dressing in atopic dermatitis. *Dermatology*. 2006;**212**(1):66-9.
132. Gonzalez-Lopez G, Ceballos-Rodriguez R, Gonzalez-Lopez J, Feito Rodriguez M, Herranz-Pinto P. Efficacy and safety of wet wrap therapy for patients with atopic dermatitis: a systematic review and meta-analysis. *Br J Dermatol*. 2017;**177**(3):688-95.
133. Beattie P, Lewis-Jones M. A pilot study on the use of wet wraps in infants with moderate atopic eczema. *Clin Exp Dermatol*. 2004;**29**(4):348-53.

For peer review only

134. Hindley D, Galloway G, Murray J, Gardener L. A randomised study of "wet wraps" versus conventional treatment for atopic eczema. *Arch Dis Child*. 2006;**91**(2):164-8.

For peer review only

Appendix 7: edits made to meta-analyses and data from Broeders *et al* 2016 [1]

1) Switched the forest plot labels around so topical corticosteroids are the intervention and topical calcineurin inhibitors are is the comparator.
2) Amended a data error given in the skin infection forest plot where the number of events and participants were given the wrong way round for topical corticosteroids and topical calcineurin inhibitors in Luger et al 2004 [2].
3) Added skin atrophy data from Sigurgeirsson et al 2015 [3] into the forest plot – this is not provided in the publication but is given in online correspondence on the journal website
4) Changed to random effects instead of fixed effects as the decision was based on whether the I^2 value which is not appropriate.
5) Bieber et al 2007 [4] was listed as "least potent" in table I of the publication – but according to the Australian potency classification it should be classified as potent.
6) In table I, the topical calcineurin inhibitors given for Mandelin et al 2010 [5] is tacrolimus 1% - this should be 0.1%.
7) In table I, the therapy given for Hofman et al 2006 [6] was hydrocortisone acetate 0.1%. However, patients used hydrocortisone ointment 1% (mild potency) twice daily for head/neck and hydrocortisone butyrate ointment 0.1% (potent) for trunk and limbs for 2 weeks then hydrocortisone 1% twice daily for flares.

References

1. Broeders J, Ahmed Ali U, Fischer G. Systematic review and meta-analysis of randomized clinical trials (RCTs) comparing topical calcineurin inhibitors with topical corticosteroids for atopic dermatitis: A 15-year experience. *J Am Acad Dermatol*. 2016;**75**(2):410-9.e3.
2. Luger T, Van Leent E, Graeber M, Hedgecock S, Thurston M, Kandra A, et al. SDZ ASM 981: an emerging safe and effective treatment for atopic dermatitis. *Br J Dermatol*. 2001;**144**(4):788-94.
3. Sigurgeirsson B, Boznanski A, Todd G, Vertruyen A, Schuttelaar M, Zhu X, et al. Safety and efficacy of pimecrolimus in atopic dermatitis: a 5-year randomized trial. *Pediatrics* 2015;**135**(4):597-606.
4. Bieber T, Vick K, Folster-Holst R, Belloni-Fortina A, Stadtler G, Worm M, et al. Efficacy and safety of methylprednisolone aceponate ointment 0.1% compared to tacrolimus 0.03% in children and adolescents with an acute flare of severe atopic dermatitis. *Allergy*. 2007;**62**(2):184-9.
5. Mandelin J, Remitz A, Virtanen H, Reitamo S. One-year treatment with 0.1% tacrolimus ointment versus a corticosteroid regimen in adults with moderate to severe atopic dermatitis: A randomized, double-blind, comparative trial. *Acta Derm Venereol*. 2010;**90**(2):170-4.
6. Hofman T, Cranswick N, Kuna P, Boznanski A, Latos T, Gold M, et al. Tacrolimus ointment does not affect the immediate response to vaccination, the generation of immune memory, or humoral and cell-mediated immunity in children. *Arch Dis Child*. 2006;**91**(11):905-10.

For peer review only

Appendix 8 – meta-analysis of TCS versus TCI – cutaneous adverse events

1) Skin thinning

Study or Subgroup	Corticosteroid (TCS) Events	Corticosteroid (TCS) Total	Calcineurin inhibitor (TCI) Events	Calcineurin inhibitor (TCI) Total	Weight	Risk Ratio M-H, Random, 95% CI
Luger 2004 (1)	3	330	0	328	26.5%	6.96 [0.36, 134.17]
Mandelin 2010 (2)	2	40	0	40	25.7%	5.00 [0.25, 100.97]
Reitamo 2005 (3)	2	485	0	487	25.2%	5.02 [0.24, 104.30]
Sigurgeirsson 2015 (4)	1	1213	0	1205	22.7%	2.98 [0.12, 73.08]
**Total (95% CI)**		**2068**		**2060**	**100.0%**	**4.86 [1.06, 22.28]**
Total events	8		0			

Heterogeneity: Tau² = 0.00; Chi² = 0.15, df = 3 (P = 0.99); I² = 0%
Test for overall effect: Z = 2.03 (P = 0.04)

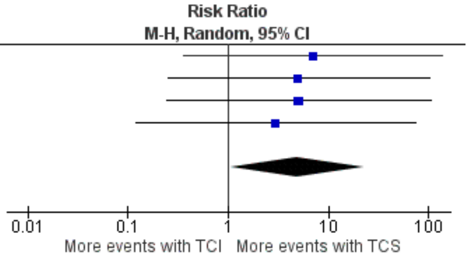

2) Skin burning

Study or Subgroup	Corticosteroid (TCS) Events	Corticosteroid (TCS) Total	Calcineurin inhibitor (TCI) Events	Calcineurin inhibitor (TCI) Total	Weight	Risk Ratio M-H, Random, 95% CI
Doss 2009 (5)	9	279	47	287	9.1%	0.20 [0.10, 0.39]
Doss 2010 (6)	6	239	18	237	6.4%	0.33 [0.13, 0.82]
Hofman 2006 (7)	2	124	10	133	2.9%	0.21 [0.05, 0.96]
Luger 2001 (8)	4	42	22	45	5.8%	0.19 [0.07, 0.52]
Luger 2004 (1)	36	330	85	328	15.6%	0.42 [0.29, 0.60]
Reitamo 2002(I) (9)	24	186	113	191	14.9%	0.22 [0.15, 0.32]
Reitamo 2002(II) (10)	13	185	38	186	10.7%	0.34 [0.19, 0.62]
Reitamo 2004 (11)	30	207	50	210	14.5%	0.61 [0.40, 0.92]
Reitamo 2005 (3)	67	485	255	487	18.4%	0.26 [0.21, 0.33]
Sikder 2005 (12)	1	15	7	15	1.8%	0.14 [0.02, 1.02]
**Total (95% CI)**		**2092**		**2119**	**100.0%**	**0.31 [0.23, 0.40]**
Total events	192		645			

Heterogeneity: Tau² = 0.09; Chi² = 21.66, df = 9 (P = 0.01); I² = 58%
Test for overall effect: Z = 8.44 (P < 0.00001)

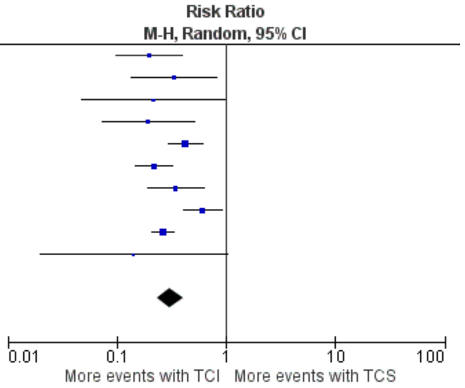

3) Pruritus

Study or Subgroup	Corticosteroid (TCS) Events	Corticosteroid (TCS) Total	Calcineurin inhibitor (TCI) Events	Calcineurin inhibitor (TCI) Total	Weight	Risk Ratio M-H, Random, 95% CI
Doss 2009 (5)	9	279	12	287	4.7%	0.77 [0.33, 1.80]
Doss 2010 (6)	8	239	10	237	4.1%	0.79 [0.32, 1.98]
Hofman 2006 (7)	4	124	8	133	2.5%	0.54 [0.17, 1.74]
Luger 2001 (8)	5	42	14	45	3.9%	0.38 [0.15, 0.97]
Luger 2004 (1)	6	330	18	328	4.1%	0.33 [0.13, 0.82]
Reitamo 2002(I) (9)	18	186	29	191	11.2%	0.64 [0.37, 1.11]
Reitamo 2002(II) (10)	14	185	21	186	8.2%	0.67 [0.35, 1.28]
Reitamo 2004 (11)	33	207	45	210	20.7%	0.74 [0.50, 1.12]
Reitamo 2005 (3)	65	485	88	487	39.3%	0.74 [0.55, 1.00]
Sikder 2005 (12)	2	15	3	15	1.3%	0.67 [0.13, 3.44]
**Total (95% CI)**		**2092**		**2119**	**100.0%**	**0.68 [0.56, 0.82]**
Total events	164		248			

Heterogeneity: Tau² = 0.00; Chi² = 4.80, df = 9 (P = 0.85); I² = 0%
Test for overall effect: Z = 4.11 (P < 0.0001)

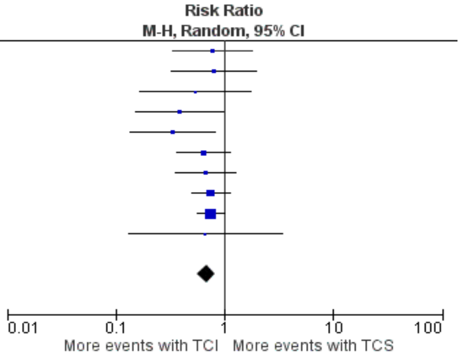

4) Skin infections

Study or Subgroup	Corticosteroid (TCS) Events	Corticosteroid (TCS) Total	Calcineurin inhibitor (TCI) Events	Calcineurin inhibitor (TCI) Total	Weight	Risk Ratio M-H, Random, 95% CI
Doss 2010 (6)	49	239	44	239	14.6%	1.11 [0.77, 1.60]
Hofman 2006 (7)	4	124	2	133	0.7%	2.15 [0.40, 11.51]
Luger 2004 (1)	80	330	69	328	24.2%	1.15 [0.87, 1.53]
Mandelin 2010 (2)	17	40	26	40	10.7%	0.65 [0.43, 1.00]
Reitamo 2002(II) (10)	4	185	4	186	1.0%	1.01 [0.26, 3.96]
Reitamo 2004 (11)	6	207	6	210	1.6%	1.01 [0.33, 3.09]
Reitamo 2005 (3)	9	485	13	487	2.8%	0.70 [0.30, 1.61]
Sigurgeirsson 2015 (4)	150	1213	157	1205	44.5%	0.95 [0.77, 1.17]
**Total (95% CI)**		**2823**		**2828**	**100.0%**	**0.98 [0.85, 1.12]**
Total events	319		321			

Heterogeneity: Tau² = 0.00; Chi² = 6.80, df = 7 (P = 0.45); I² = 0%
Test for overall effect: Z = 0.33 (P = 0.74)

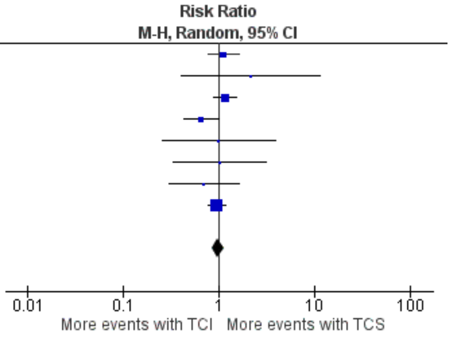

References:

1. Luger T, Lahfa M, Folster-Holst R, Gulliver W, Allen R, Molloy S, et al. Long-term safety and tolerability of pimecrolimus cream 1% and topical corticosteroids in adults with moderate to severe atopic dermatitis. *J Dermatolog Treat*. 2004;**15**(3):169-78
2. Mandelin J, Remitz A, Virtanen H, Reitamo S. One-year treatment with 0.1% tacrolimus ointment versus a corticosteroid regimen in adults with moderate to severe atopic dermatitis: A randomized, double-blind, comparative trial. *Acta Derm Venereol*. 2010;**90**(2):170-4
3. Reitamo S, Ortonne J, Sand C, Cambazard F, Bieber T, Folster-Holst R, et al. A multicentre, randomized, double-blind, controlled study of long-term treatment with 0.1% tacrolimus ointment in adults with moderate to severe atopic dermatitis. *Br J Dermatol*. 2005;**152**(6):1282-9
4. Sigurgeirsson B, Boznanski A, Todd G, Vertruyen A, Schuttelaar M, Zhu X, et al. Safety and efficacy of pimecrolimus in atopic dermatitis: a 5-year randomized trial. *Pediatrics* 2015;**135**(4):597-606
5. Doss N, Reitamo S, Dubertret L, Fekete G, Kamoun M, Lahfa M, et al. Superiority of tacrolimus 0.1% ointment compared with fluticasone 0.005% in adults with moderate to severe atopic dermatitis of the face: results from a randomized, double-blind trial. *Br J Dermatol*. 2009;**161**(2):427-34
6. Doss N, Kamoun M, Dubertret L, Cambazard F, Remitz A, Lahfa M, et al. Efficacy of tacrolimus 0.03% ointment as second-line treatment for children with moderate-to-severe atopic dermatitis: evidence from a randomized, double-blind non-inferiority trial vs. fluticasone 0.005% ointment. *Pediatr Allergy Immunol*. 2010;**21**(2 Pt 1):321-9.
7. Hofman T, Cranswick N, Kuna P, Boznanski A, Latos T, Gold M, et al. Tacrolimus ointment does not affect the immediate response to vaccination, the generation of immune memory, or humoral and cell-mediated immunity in children. *Arch Dis Child*. 2006;**91**(11):905-10
8. Luger T, Van Leent E, Graeber M, Hedgecock S, Thurston M, Kandra A, et al. SDZ ASM 981: an emerging safe and effective treatment for atopic dermatitis. *Br J Dermatol*. 2001;**144**(4):788-94
9. Reitamo S, Rustin M, Ruzicka T, Cambazard F, Kalimo K, Friedmann P, et al. Efficacy and safety of tacrolimus ointment compared with that of hydrocortisone butyrate ointment in adult patients with atopic dermatitis. *J Allergy Clin Immunol*. 2002;**109(**3):547-55
10. Reitamo S, Van Leent E, Ho V, Harper J, Ruzicka T, Kalimo K, et al. Efficacy and safety of tacrolimus ointment compared with that of hydrocortisone acetate ointment in children with atopic dermatitis. *J Allergy Clin Immunol*. 2002;**109**(3):539-46
11. Reitamo S, Harper J, Bos J, Cambazard F, Bruijnzeel-Koomen C, Valk P, et al. 0.03% Tacrolimus ointment applied once or twice daily is more efficacious than 1% hydrocortisone acetate in children with moderate to severe atopic dermatitis: results of a randomized double-blind controlled trial. *Br J Dermatol*. 2004;**150**(3):554-62
12. Sikder M, Al Mamun S, Khan R, Chowdhury A, Khan H, Hoque M. Topical 0.03% tacrolimus ointment, 0.05% clobetasone butyrate cream alone and their combination in older children with atopic dermatitis - An open randomized comparative study. *J Pak Assoc Dermatol*. 2005;**15**(4):304-12

For peer review only

Appendix 9: subgroup analyses of TCS versus TCI – cutaneous adverse events

By different topical corticosteroid potencies

Skin thinning

	Corticosteroids (TCS)		Calcineurin inhibitor (TCI)			Risk Ratio
**Study or Subgroup**	**Events**	**Total**	**Events**	**Total**	**Weight**	**M-H, Random, 95% CI**
**2.6.1 Potent**						
Luger 2004 (1)	3	330	0	328	26.5%	6.96 [0.36, 134.17]
Mandelin 2010 (2)	2	40	0	40	25.7%	5.00 [0.25, 100.97]
Reitamo 2005 (3)	2	485	0	487	25.2%	5.02 [0.24, 104.30]
**Subtotal (95% CI)**		**855**		**855**	**77.3%**	**5.61 [0.99, 31.67]**
Total events	7		0			
Heterogeneity: Tau^2 = 0.00; Chi^2 = 0.03, df = 2 (P = 0.98); I^2 = 0%						
Test for overall effect: Z = 1.95 (P = 0.05)						
**2.6.2 Moderate or mild potency**						
Sigurgeirsson 2015 (4)	1	1213	0	1205	22.7%	2.98 [0.12, 73.08]
**Subtotal (95% CI)**		**1213**		**1205**	**22.7%**	**2.98 [0.12, 73.08]**
Total events	1		0			
Heterogeneity: Not applicable						
Test for overall effect: Z = 0.67 (P = 0.50)						
**Total (95% CI)**		**2068**		**2060**	**100.0%**	**4.86 [1.06, 22.28]**
Total events	8		0			
Heterogeneity: Tau^2 = 0.00; Chi^2 = 0.15, df = 3 (P = 0.99); I^2 = 0%						
Test for overall effect: Z = 2.03 (P = 0.04)						
Test for subgroup differences: Chi^2 = 0.12, df = 1 (P = 0.73), I^2 = 0%						

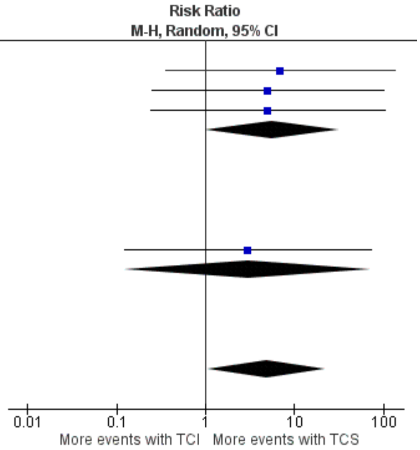

Skin burning

	Corticosteroids (TCS)		Calcineurin inhibitor (TCI)			Risk Ratio
**Study or Subgroup**	**Events**	**Total**	**Events**	**Total**	**Weight**	**M-H, Random, 95% CI**
**2.2.1 Potent**						
Doss 2009 (5)	9	279	47	287	9.1%	0.20 [0.10, 0.39]
Doss 2010 (6)	6	239	18	237	6.4%	0.33 [0.13, 0.82]
Luger 2001 (7)	4	42	22	45	5.8%	0.19 [0.07, 0.52]
Luger 2004 (1)	36	330	85	328	15.6%	0.42 [0.29, 0.60]
Reitamo 2002(I) (8)	24	186	113	191	14.9%	0.22 [0.15, 0.32]
Reitamo 2005 (3)	67	485	255	487	18.4%	0.26 [0.21, 0.33]
**Subtotal (95% CI)**		**1561**		**1575**	**70.2%**	**0.27 [0.21, 0.35]**
Total events	146		540			
Heterogeneity: Tau^2 = 0.04; Chi^2 = 8.42, df = 5 (P = 0.13); I^2 = 41%						
Test for overall effect: Z = 10.11 (P < 0.00001)						
**2.2.2 Potent or mild potency**						
Hofman 2006 (9)	2	124	10	133	2.9%	0.21 [0.05, 0.96]
**Subtotal (95% CI)**		**124**		**133**	**2.9%**	**0.21 [0.05, 0.96]**
Total events	2		10			
Heterogeneity: Not applicable						
Test for overall effect: Z = 2.01 (P = 0.04)						
**2.2.3 Moderate potency**						
Sikder 2005 (10)	1	15	7	15	1.8%	0.14 [0.02, 1.02]
**Subtotal (95% CI)**		**15**		**15**	**1.8%**	**0.14 [0.02, 1.02]**
Total events	1		7			
Heterogeneity: Not applicable						
Test for overall effect: Z = 1.94 (P = 0.05)						
**2.2.4 Mild potency**						
Reitamo 2002(II) (11)	13	185	38	186	10.7%	0.34 [0.19, 0.62]
Reitamo 2004 (12)	30	207	50	210	14.5%	0.61 [0.40, 0.92]
**Subtotal (95% CI)**		**392**		**396**	**25.1%**	**0.48 [0.27, 0.83]**
Total events	43		88			
Heterogeneity: Tau^2 = 0.10; Chi^2 = 2.41, df = 1 (P = 0.12); I^2 = 59%						
Test for overall effect: Z = 2.61 (P = 0.009)						
**Total (95% CI)**		**2092**		**2119**	**100.0%**	**0.31 [0.23, 0.40]**
Total events	192		645			
Heterogeneity: Tau^2 = 0.09; Chi^2 = 21.66, df = 9 (P = 0.01); I^2 = 58%						
Test for overall effect: Z = 8.44 (P < 0.00001)						
Test for subgroup differences: Chi^2 = 3.92, df = 3 (P = 0.27), I^2 = 23.4%						

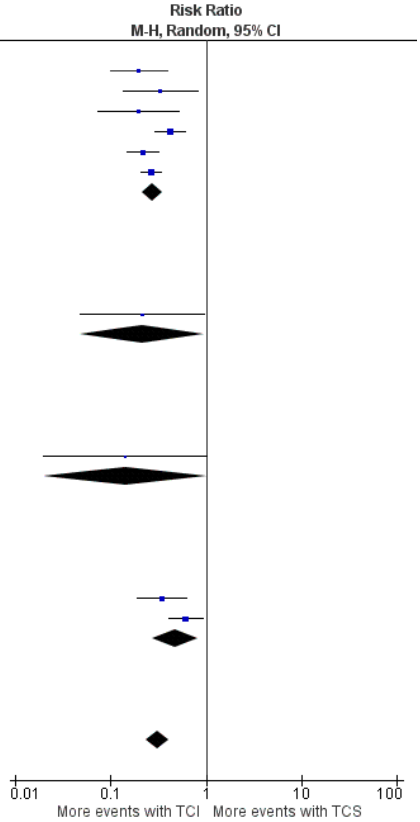

For peer review only - http://bmjopen.bmj.com/site/about/guidelines.xhtml

Pruritus

Study or Subgroup	Corticosteroids (TCS) Events	Corticosteroids (TCS) Total	Calcineurin inhibitor (TCI) Events	Calcineurin inhibitor (TCI) Total	Weight	Risk Ratio M-H, Random, 95% CI
**2.3.1 Potent**						
Doss 2009 (5)	9	279	12	287	4.7%	0.77 [0.33, 1.80]
Doss 2010 (6)	8	239	10	237	4.1%	0.79 [0.32, 1.98]
Luger 2001 (7)	5	42	14	45	3.9%	0.38 [0.15, 0.97]
Luger 2004 (1)	6	330	18	328	4.1%	0.33 [0.13, 0.82]
Reitamo 2002(I) (8)	18	186	29	191	11.2%	0.64 [0.37, 1.11]
Reitamo 2005 (3)	65	485	88	487	39.3%	0.74 [0.55, 1.00]
**Subtotal (95% CI)**		**1561**		**1575**	**67.4%**	**0.67 [0.53, 0.84]**
Total events	111		171			
Heterogeneity: Tau² = 0.00; Chi² = 4.43, df = 5 (P = 0.49); I² = 0%						
Test for overall effect: Z = 3.53 (P = 0.0004)						
**2.3.2 Potent or mild potency**						
Hofman 2006 (9)	4	124	8	133	2.5%	0.54 [0.17, 1.74]
**Subtotal (95% CI)**		**124**		**133**	**2.5%**	**0.54 [0.17, 1.74]**
Total events	4		8			
Heterogeneity: Not applicable						
Test for overall effect: Z = 1.04 (P = 0.30)						
**2.3.3 Moderate potency**						
Sikder 2005 (10)	2	15	3	15	1.3%	0.67 [0.13, 3.44]
**Subtotal (95% CI)**		**15**		**15**	**1.3%**	**0.67 [0.13, 3.44]**
Total events	2		3			
Heterogeneity: Not applicable						
Test for overall effect: Z = 0.48 (P = 0.63)						
**2.3.4 Mild potency**						
Reitamo 2002(II) (11)	14	185	21	186	8.2%	0.67 [0.35, 1.28]
Reitamo 2004 (12)	33	207	45	210	20.7%	0.74 [0.50, 1.12]
**Subtotal (95% CI)**		**392**		**396**	**28.9%**	**0.72 [0.51, 1.02]**
Total events	47		66			
Heterogeneity: Tau² = 0.00; Chi² = 0.07, df = 1 (P = 0.79); I² = 0%						
Test for overall effect: Z = 1.86 (P = 0.06)						
**Total (95% CI)**		**2092**		**2119**	**100.0%**	**0.68 [0.56, 0.82]**
Total events	164		248			
Heterogeneity: Tau² = 0.00; Chi² = 4.80, df = 9 (P = 0.85); I² = 0%						
Test for overall effect: Z = 4.11 (P < 0.0001)						
Test for subgroup differences: Chi² = 0.30, df = 3 (P = 0.96), I² = 0%						

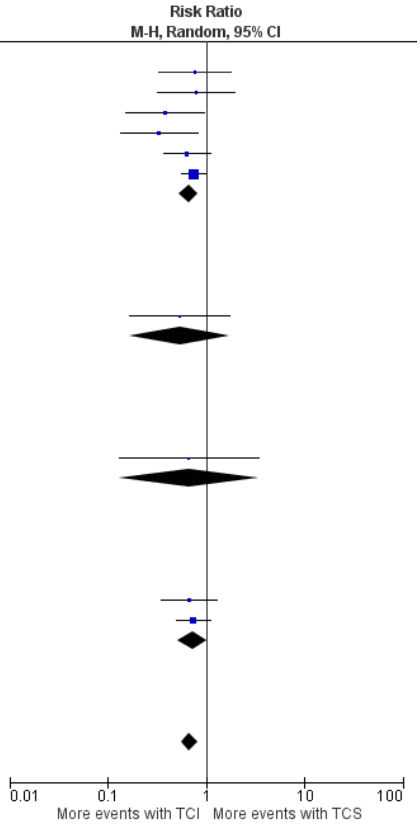

Skin infections

Study or Subgroup	Corticosteroids (TCS) Events	Corticosteroids (TCS) Total	Calcineurin inhibitor (TCI) Events	Calcineurin inhibitor (TCI) Total	Weight	Risk Ratio M-H, Random, 95% CI
**2.7.1 Potent**						
Doss 2010 (6)	49	239	44	239	14.6%	1.11 [0.77, 1.60]
Luger 2004 (1)	80	330	69	328	24.2%	1.15 [0.87, 1.53]
Mandelin 2010 (2)	17	40	26	40	10.7%	0.65 [0.43, 1.00]
Reitamo 2005 (3)	9	485	13	487	2.8%	0.70 [0.30, 1.61]
**Subtotal (95% CI)**		**1094**		**1094**	**52.2%**	**0.94 [0.70, 1.26]**
Total events	155		152			
Heterogeneity: Tau² = 0.04; Chi² = 5.90, df = 3 (P = 0.12); I² = 49%						
Test for overall effect: Z = 0.41 (P = 0.68)						
**2.7.2 Potent or mild potency**						
Hofman 2006 (9)	4	124	2	133	0.7%	2.15 [0.40, 11.51]
**Subtotal (95% CI)**		**124**		**133**	**0.7%**	**2.15 [0.40, 11.51]**
Total events	4		2			
Heterogeneity: Not applicable						
Test for overall effect: Z = 0.89 (P = 0.37)						
**2.7.3 Moderate or mild potency**						
Sigurgeirsson 2015 (4)	150	1213	157	1205	44.5%	0.95 [0.77, 1.17]
**Subtotal (95% CI)**		**1213**		**1205**	**44.5%**	**0.95 [0.77, 1.17]**
Total events	150		157			
Heterogeneity: Not applicable						
Test for overall effect: Z = 0.49 (P = 0.62)						
**2.7.4 Mild potency**						
Reitamo 2002(II) (11)	4	185	4	186	1.0%	1.01 [0.26, 3.96]
Reitamo 2004 (12)	6	207	6	210	1.6%	1.01 [0.33, 3.09]
**Subtotal (95% CI)**		**392**		**396**	**2.6%**	**1.01 [0.43, 2.40]**
Total events	10		10			
Heterogeneity: Tau² = 0.00; Chi² = 0.00, df = 1 (P = 0.99); I² = 0%						
Test for overall effect: Z = 0.02 (P = 0.98)						
**Total (95% CI)**		**2823**		**2828**	**100.0%**	**0.98 [0.85, 1.12]**
Total events	319		321			
Heterogeneity: Tau² = 0.00; Chi² = 6.80, df = 7 (P = 0.45); I² = 0%						
Test for overall effect: Z = 0.33 (P = 0.74)						
Test for subgroup differences: Chi² = 0.92, df = 3 (P = 0.82), I² = 0%						

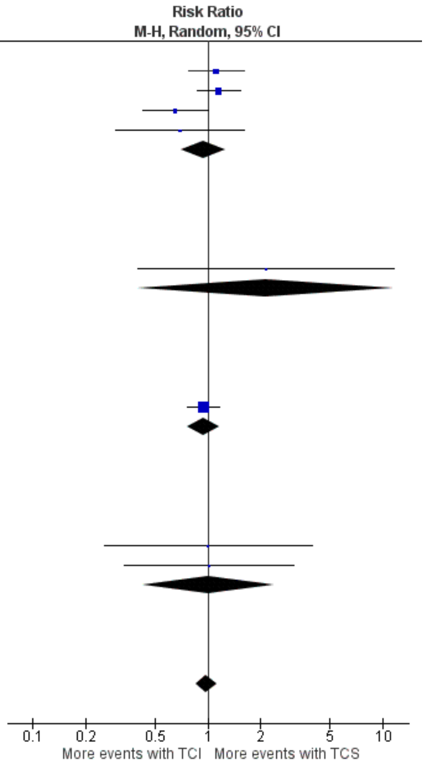

2

By age of participants (children or adults)

Skin thinning

Study or Subgroup	Corticosteroids (TCS) Events	Total	Calcineurin inhibitor (TCI) Events	Total	Weight	Risk Ratio M-H, Random, 95% CI
**3.6.1 Adults**						
Luger 2004 (1)	3	330	0	328	26.5%	6.96 [0.36, 134.17]
Mandelin 2010 (2)	2	40	0	40	25.7%	5.00 [0.25, 100.97]
Reitamo 2005 (3)	2	485	0	487	25.2%	5.02 [0.24, 104.30]
**Subtotal (95% CI)**		**855**		**855**	**77.3%**	**5.61 [0.99, 31.67]**
Total events	7		0			
Heterogeneity: Tau² = 0.00; Chi² = 0.03, df = 2 (P = 0.98); I² = 0%						
Test for overall effect: Z = 1.95 (P = 0.05)						
**3.6.2 Children**						
Sigurgeirsson 2015 (4)	1	1213	0	1205	22.7%	2.98 [0.12, 73.08]
**Subtotal (95% CI)**		**1213**		**1205**	**22.7%**	**2.98 [0.12, 73.08]**
Total events	1		0			
Heterogeneity: Not applicable						
Test for overall effect: Z = 0.67 (P = 0.50)						
**Total (95% CI)**		**2068**		**2060**	**100.0%**	**4.86 [1.06, 22.28]**
Total events	8		0			
Heterogeneity: Tau² = 0.00; Chi² = 0.15, df = 3 (P = 0.99); I² = 0%						
Test for overall effect: Z = 2.03 (P = 0.04)						
Test for subgroup differences: Chi² = 0.12, df = 1 (P = 0.73), I² = 0%						

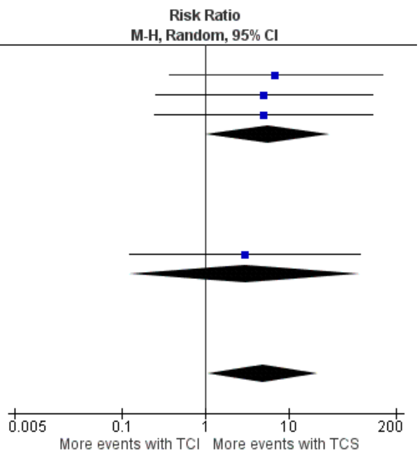

Skin burning

Study or Subgroup	Corticosteroids (TCS) Events	Total	Calcineurin inhibitor (TCI) Events	Total	Weight	Risk Ratio M-H, Random, 95% CI
**3.2.1 Adults**						
Doss 2009 (5)	9	279	47	287	9.1%	0.20 [0.10, 0.39]
Luger 2001 (7)	4	42	22	45	5.8%	0.19 [0.07, 0.52]
Luger 2004 (1)	36	330	85	328	15.6%	0.42 [0.29, 0.60]
Reitamo 2002(I) (8)	24	186	113	191	14.9%	0.22 [0.15, 0.32]
Reitamo 2005 (3)	67	485	255	487	18.4%	0.26 [0.21, 0.33]
**Subtotal (95% CI)**		**1322**		**1338**	**63.7%**	**0.27 [0.20, 0.36]**
Total events	140		522			
Heterogeneity: Tau² = 0.05; Chi² = 8.28, df = 4 (P = 0.08); I² = 52%						
Test for overall effect: Z = 9.17 (P < 0.00001)						
**3.2.2 Children**						
Doss 2010 (6)	6	239	18	237	6.4%	0.33 [0.13, 0.82]
Hofman 2006 (9)	2	124	10	133	2.9%	0.21 [0.05, 0.96]
Reitamo 2002(II) (11)	13	185	38	186	10.7%	0.34 [0.19, 0.62]
Reitamo 2004 (12)	30	207	50	210	14.5%	0.61 [0.40, 0.92]
Sikder 2005 (10)	1	15	7	15	1.8%	0.14 [0.02, 1.02]
**Subtotal (95% CI)**		**770**		**781**	**36.3%**	**0.40 [0.26, 0.62]**
Total events	52		123			
Heterogeneity: Tau² = 0.07; Chi² = 5.68, df = 4 (P = 0.22); I² = 30%						
Test for overall effect: Z = 4.17 (P < 0.0001)						
**Total (95% CI)**		**2092**		**2119**	**100.0%**	**0.31 [0.23, 0.40]**
Total events	192		645			
Heterogeneity: Tau² = 0.09; Chi² = 21.66, df = 9 (P = 0.01); I² = 58%						
Test for overall effect: Z = 8.44 (P < 0.00001)						
Test for subgroup differences: Chi² = 2.48, df = 1 (P = 0.11), I² = 59.8%						

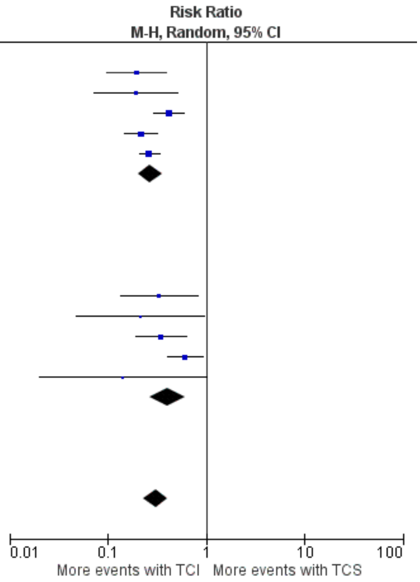

For peer review only - http://bmjopen.bmj.com/site/about/guidelines.xhtml

Pruritus

Study or Subgroup	Corticosteroids (TCS) Events	Total	Calcineurin inhibitor (TCI) Events	Total	Weight	Risk Ratio M-H, Random, 95% CI
**3.3.1 Adults**						
Doss 2009 (5)	9	279	12	287	4.7%	0.77 [0.33, 1.80]
Luger 2001 (7)	5	42	14	45	3.9%	0.38 [0.15, 0.97]
Luger 2004 (1)	6	330	18	328	4.1%	0.33 [0.13, 0.82]
Reitamo 2002(I) (8)	18	186	29	191	11.2%	0.64 [0.37, 1.11]
Reitamo 2005 (3)	65	485	88	487	39.3%	0.74 [0.55, 1.00]
**Subtotal (95% CI)**		**1322**		**1338**	**63.2%**	**0.65 [0.50, 0.84]**
Total events	103		161			
Heterogeneity: Tau^2 = 0.01; Chi^2 = 4.28, df = 4 (P = 0.37); I^2 = 7%						
Test for overall effect: Z = 3.34 (P = 0.0008)						
**3.3.2 Children**						
Doss 2010 (6)	8	239	10	237	4.1%	0.79 [0.32, 1.98]
Hofman 2006 (9)	4	124	8	133	2.5%	0.54 [0.17, 1.74]
Reitamo 2002(II) (11)	14	185	21	186	8.2%	0.67 [0.35, 1.28]
Reitamo 2004 (12)	33	207	45	210	20.7%	0.74 [0.50, 1.12]
Sikder 2005 (10)	2	15	3	15	1.3%	0.67 [0.13, 3.44]
**Subtotal (95% CI)**		**770**		**781**	**36.8%**	**0.71 [0.53, 0.97]**
Total events	61		87			
Heterogeneity: Tau^2 = 0.00; Chi^2 = 0.36, df = 4 (P = 0.99); I^2 = 0%						
Test for overall effect: Z = 2.17 (P = 0.03)						
**Total (95% CI)**		**2092**		**2119**	**100.0%**	**0.68 [0.56, 0.82]**
Total events	164		248			
Heterogeneity: Tau^2 = 0.00; Chi^2 = 4.80, df = 9 (P = 0.85); I^2 = 0%						
Test for overall effect: Z = 4.11 (P < 0.0001)						
Test for subgroup differences: Chi^2 = 0.23, df = 1 (P = 0.63), I^2 = 0%						

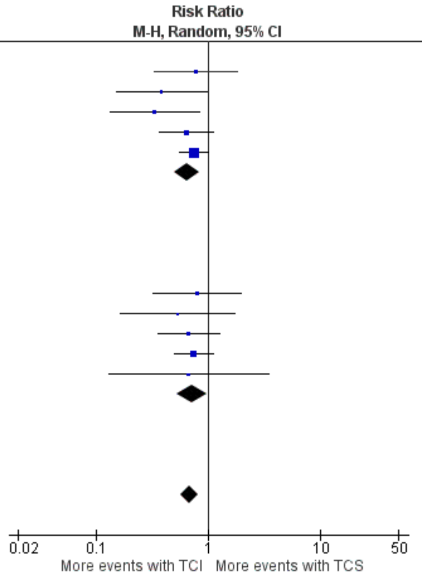

Skin infections

Study or Subgroup	Corticosteroids (TCS) Events	Total	Calcineurin inhibitor (TCI) Events	Total	Weight	Risk Ratio M-H, Random, 95% CI
**3.7.1 Adults**						
Luger 2004 (1)	80	330	69	328	24.2%	1.15 [0.87, 1.53]
Mandelin 2010 (2)	17	40	26	40	10.7%	0.65 [0.43, 1.00]
Reitamo 2005 (3)	9	485	13	487	2.8%	0.70 [0.30, 1.61]
**Subtotal (95% CI)**		**855**		**855**	**37.7%**	**0.85 [0.55, 1.32]**
Total events	106		108			
Heterogeneity: Tau^2 = 0.09; Chi^2 = 5.35, df = 2 (P = 0.07); I^2 = 63%						
Test for overall effect: Z = 0.70 (P = 0.48)						
**3.7.2 Children**						
Doss 2010 (6)	49	239	44	239	14.6%	1.11 [0.77, 1.60]
Hofman 2006 (9)	4	124	2	133	0.7%	2.15 [0.40, 11.51]
Reitamo 2002(II) (11)	4	185	4	186	1.0%	1.01 [0.26, 3.96]
Reitamo 2004 (12)	6	207	6	210	1.6%	1.01 [0.33, 3.09]
Sigurgeirsson 2015 (4)	150	1213	157	1205	44.5%	0.95 [0.77, 1.17]
**Subtotal (95% CI)**		**1968**		**1973**	**62.3%**	**1.00 [0.84, 1.19]**
Total events	213		213			
Heterogeneity: Tau^2 = 0.00; Chi^2 = 1.37, df = 4 (P = 0.85); I^2 = 0%						
Test for overall effect: Z = 0.04 (P = 0.97)						
**Total (95% CI)**		**2823**		**2828**	**100.0%**	**0.98 [0.85, 1.12]**
Total events	319		321			
Heterogeneity: Tau^2 = 0.00; Chi^2 = 6.80, df = 7 (P = 0.45); I^2 = 0%						
Test for overall effect: Z = 0.33 (P = 0.74)						
Test for subgroup differences: Chi^2 = 0.41, df = 1 (P = 0.52), I^2 = 0%						

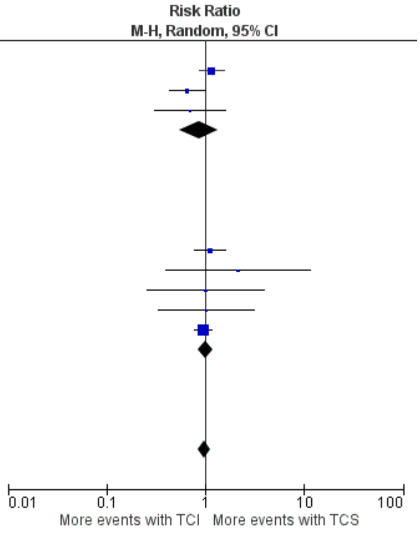

For peer review only - http://bmjopen.bmj.com/site/about/guidelines.xhtml

By individual topical calcineurin inhibitor (TCI)

Skin thinning

Study or Subgroup	Corticosteroids (TCS) Events	Corticosteroids (TCS) Total	Calcineurin inhibitor (TCI) Events	Calcineurin inhibitor (TCI) Total	Weight	Risk Ratio M-H, Random, 95% CI
**4.6.1 Tacrolimus 0.1%**						
Mandelin 2010 (2)	2	40	0	40	25.7%	5.00 [0.25, 100.97]
Reitamo 2005 (3)	2	485	0	487	25.2%	5.02 [0.24, 104.30]
**Subtotal (95% CI)**		**525**		**527**	**50.9%**	**5.01 [0.59, 42.37]**
Total events	4		0			
Heterogeneity: Tau² = 0.00; Chi² = 0.00, df = 1 (P = 1.00); I² = 0%						
Test for overall effect: Z = 1.48 (P = 0.14)						
**4.6.2 Pimecrolimus 1%**						
Luger 2004 (1)	3	330	0	328	26.5%	6.96 [0.36, 134.17]
Sigurgeirsson 2015 (4)	1	1213	0	1205	22.7%	2.98 [0.12, 73.08]
**Subtotal (95% CI)**		**1543**		**1533**	**49.1%**	**4.71 [0.54, 41.33]**
Total events	4		0			
Heterogeneity: Tau² = 0.00; Chi² = 0.15, df = 1 (P = 0.70); I² = 0%						
Test for overall effect: Z = 1.40 (P = 0.16)						
**Total (95% CI)**		**2068**		**2060**	**100.0%**	**4.86 [1.06, 22.28]**
Total events	8		0			
Heterogeneity: Tau² = 0.00; Chi² = 0.15, df = 3 (P = 0.99); I² = 0%						
Test for overall effect: Z = 2.03 (P = 0.04)						
Test for subgroup differences: Chi² = 0.00, df = 1 (P = 0.97), I² = 0%						

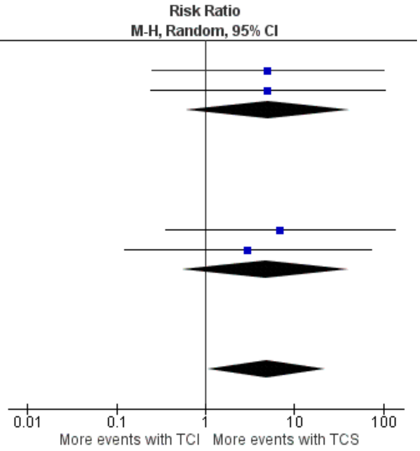

Skin burning

Study or Subgroup	Corticosteroids (TCS) Events	Corticosteroids (TCS) Total	Calcineurin inhibitor (TCI) Events	Calcineurin inhibitor (TCI) Total	Weight	Risk Ratio M-H, Random, 95% CI
**4.2.1 Tacrolimus 0.1%**						
Doss 2009 (5)	9	279	47	287	9.1%	0.20 [0.10, 0.39]
Reitamo 2002(I) (8)	24	186	113	191	14.9%	0.22 [0.15, 0.32]
Reitamo 2002(II) (11)	13	185	38	186	10.7%	0.34 [0.19, 0.62]
Reitamo 2005 (3)	67	485	255	487	18.4%	0.26 [0.21, 0.33]
**Subtotal (95% CI)**		**1135**		**1151**	**53.0%**	**0.25 [0.21, 0.31]**
Total events	113		453			
Heterogeneity: Tau² = 0.00; Chi² = 2.19, df = 3 (P = 0.53); I² = 0%						
Test for overall effect: Z = 14.49 (P < 0.00001)						
**4.2.2 Tacrolimus 0.03%**						
Doss 2010 (6)	6	239	18	237	6.4%	0.33 [0.13, 0.82]
Hofman 2006 (9)	2	124	10	133	2.9%	0.21 [0.05, 0.96]
Reitamo 2004 (12)	30	207	50	210	14.5%	0.61 [0.40, 0.92]
Sikder 2005 (10)	1	15	7	15	1.8%	0.14 [0.02, 1.02]
**Subtotal (95% CI)**		**585**		**595**	**25.6%**	**0.40 [0.22, 0.73]**
Total events	39		85			
Heterogeneity: Tau² = 0.13; Chi² = 4.60, df = 3 (P = 0.20); I² = 35%						
Test for overall effect: Z = 3.00 (P = 0.003)						
**4.2.3 Pimecrolimus 1%**						
Luger 2001 (7)	4	42	22	45	5.8%	0.19 [0.07, 0.52]
Luger 2004 (1)	36	330	85	328	15.6%	0.42 [0.29, 0.60]
**Subtotal (95% CI)**		**372**		**373**	**21.4%**	**0.33 [0.16, 0.67]**
Total events	40		107			
Heterogeneity: Tau² = 0.16; Chi² = 2.12, df = 1 (P = 0.15); I² = 53%						
Test for overall effect: Z = 3.08 (P = 0.002)						
**Total (95% CI)**		**2092**		**2119**	**100.0%**	**0.31 [0.23, 0.40]**
Total events	192		645			
Heterogeneity: Tau² = 0.09; Chi² = 21.66, df = 9 (P = 0.01); I² = 58%						
Test for overall effect: Z = 8.44 (P < 0.00001)						
Test for subgroup differences: Chi² = 2.43, df = 2 (P = 0.30), I² = 17.8%						

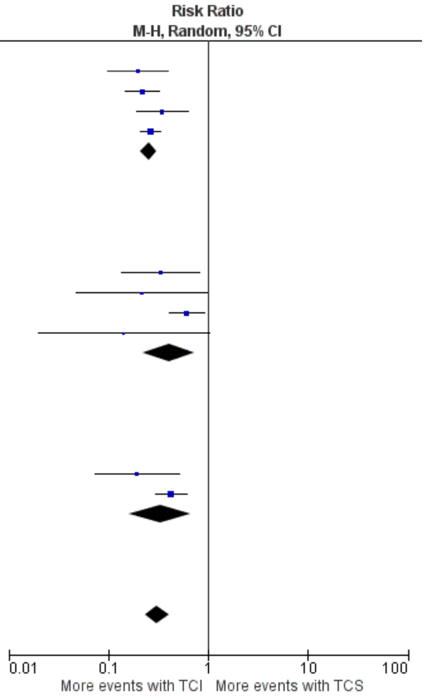

For peer review only - http://bmjopen.bmj.com/site/about/guidelines.xhtml

Pruritus

Study or Subgroup	Corticosteroids (TCS) Events	Total	Calcineurin inhibitor (TCI) Events	Total	Weight	Risk Ratio M-H, Random, 95% CI
**4.3.1 Tacrolimus 0.1%**						
Doss 2009 (5)	9	279	12	287	4.7%	0.77 [0.33, 1.80]
Reitamo 2002(I) (8)	18	186	29	191	11.2%	0.64 [0.37, 1.11]
Reitamo 2002(II) (11)	14	185	21	186	8.2%	0.67 [0.35, 1.28]
Reitamo 2005 (3)	65	485	88	487	39.3%	0.74 [0.55, 1.00]
**Subtotal (95% CI)**		**1135**		**1151**	**63.4%**	**0.71 [0.57, 0.90]**
Total events	106		150			
Heterogeneity: Tau² = 0.00; Chi² = 0.30, df = 3 (P = 0.96); I² = 0%						
Test for overall effect: Z = 2.84 (P = 0.005)						
**4.3.2 Tacrolimus 0.03%**						
Doss 2010 (6)	8	239	10	237	4.1%	0.79 [0.32, 1.98]
Hofman 2006 (9)	4	124	8	133	2.5%	0.54 [0.17, 1.74]
Reitamo 2004 (12)	33	207	45	210	20.7%	0.74 [0.50, 1.12]
Sikder 2005 (10)	2	15	3	15	1.3%	0.67 [0.13, 3.44]
**Subtotal (95% CI)**		**585**		**595**	**28.5%**	**0.73 [0.51, 1.03]**
Total events	47		66			
Heterogeneity: Tau² = 0.00; Chi² = 0.32, df = 3 (P = 0.96); I² = 0%						
Test for overall effect: Z = 1.81 (P = 0.07)						
**4.3.3 Pimecrolimus 1%**						
Luger 2001 (7)	5	42	14	45	3.9%	0.38 [0.15, 0.97]
Luger 2004 (1)	6	330	18	328	4.1%	0.33 [0.13, 0.82]
**Subtotal (95% CI)**		**372**		**373**	**8.1%**	**0.36 [0.19, 0.68]**
Total events	11		32			
Heterogeneity: Tau² = 0.00; Chi² = 0.05, df = 1 (P = 0.83); I² = 0%						
Test for overall effect: Z = 3.11 (P = 0.002)						
**Total (95% CI)**		**2092**		**2119**	**100.0%**	**0.68 [0.56, 0.82]**
Total events	164		248			
Heterogeneity: Tau² = 0.00; Chi² = 4.80, df = 9 (P = 0.85); I² = 0%						
Test for overall effect: Z = 4.11 (P < 0.0001)						
Test for subgroup differences: Chi² = 4.13, df = 2 (P = 0.13), I² = 51.6%						

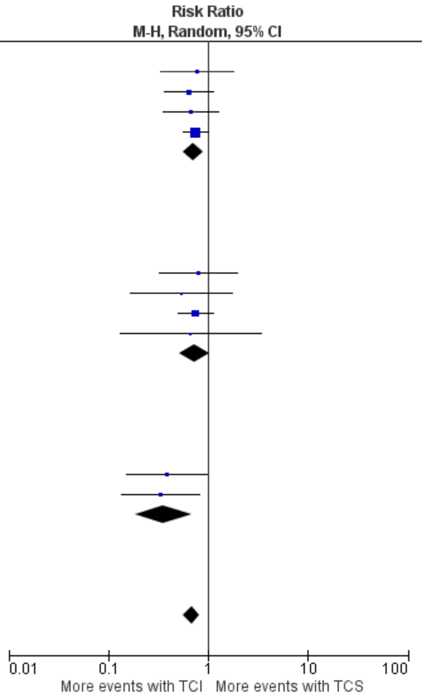

revi

Skin infections

Study or Subgroup	Corticosteroids (TCS) Events	Total	Calcineurin inhibitor (TCI) Events	Total	Weight	Risk Ratio M-H, Random, 95% CI
**4.7.1 Tacrolimus 0.1%**						
Mandelin 2010 (2)	17	40	26	40	10.7%	0.65 [0.43, 1.00]
Reitamo 2002(II) (11)	4	185	4	186	1.0%	1.01 [0.26, 3.96]
Reitamo 2005 (3)	9	485	13	487	2.8%	0.70 [0.30, 1.61]
**Subtotal (95% CI)**		**710**		**713**	**14.5%**	**0.68 [0.47, 0.98]**
Total events	30		43			
Heterogeneity: Tau² = 0.00; Chi² = 0.36, df = 2 (P = 0.83); I² = 0%						
Test for overall effect: Z = 2.05 (P = 0.04)						
**4.7.2 Tacrolimus 0.03%**						
Doss 2010 (6)	49	239	44	239	14.6%	1.11 [0.77, 1.60]
Hofman 2006 (9)	4	124	2	133	0.7%	2.15 [0.40, 11.51]
Reitamo 2004 (12)	6	207	6	210	1.6%	1.01 [0.33, 3.09]
**Subtotal (95% CI)**		**570**		**582**	**16.8%**	**1.13 [0.81, 1.59]**
Total events	59		52			
Heterogeneity: Tau² = 0.00; Chi² = 0.60, df = 2 (P = 0.74); I² = 0%						
Test for overall effect: Z = 0.73 (P = 0.47)						
**4.7.3 Pimecrolimus 1%**						
Luger 2004 (1)	80	330	69	328	24.2%	1.15 [0.87, 1.53]
Sigurgeirsson 2015 (4)	150	1213	157	1205	44.5%	0.95 [0.77, 1.17]
**Subtotal (95% CI)**		**1543**		**1533**	**68.7%**	**1.02 [0.85, 1.23]**
Total events	230		226			
Heterogeneity: Tau² = 0.00; Chi² = 1.17, df = 1 (P = 0.28); I² = 15%						
Test for overall effect: Z = 0.22 (P = 0.83)						
**Total (95% CI)**		**2823**		**2828**	**100.0%**	**0.98 [0.85, 1.12]**
Total events	319		321			
Heterogeneity: Tau² = 0.00; Chi² = 6.80, df = 7 (P = 0.45); I² = 0%						
Test for overall effect: Z = 0.33 (P = 0.74)						
Test for subgroup differences: Chi² = 4.65, df = 2 (P = 0.10), I² = 57.0%						

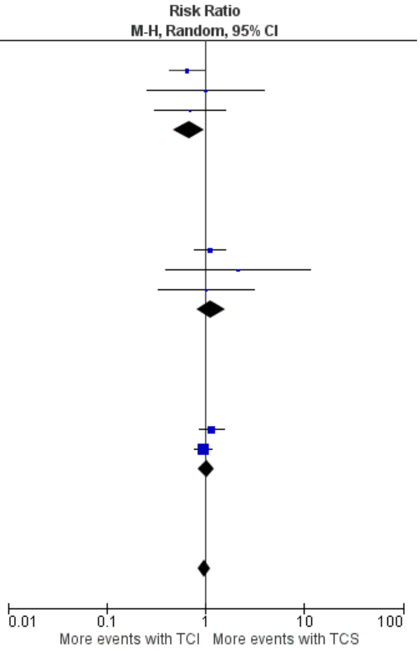

For peer review only - http://bmjopen.bmj.com/site/about/guidelines.xhtml

References:

1. Luger T, Lahfa M, Folster-Holst R, Gulliver W, Allen R, Molloy S, et al. Long-term safety and tolerability of pimecrolimus cream 1% and topical corticosteroids in adults with moderate to severe atopic dermatitis. *J Dermatolog Treat*. 2004;**15**(3):169-78
2. Mandelin J, Remitz A, Virtanen H, Reitamo S. One-year treatment with 0.1% tacrolimus ointment versus a corticosteroid regimen in adults with moderate to severe atopic dermatitis: A randomized, double-blind, comparative trial. *Acta Derm Venereol*. 2010;**90**(2):170-4
3. Reitamo S, Ortonne J, Sand C, Cambazard F, Bieber T, Folster-Holst R, et al. A multicentre, randomized, double-blind, controlled study of long-term treatment with 0.1% tacrolimus ointment in adults with moderate to severe atopic dermatitis. *Br J Dermatol*. 2005;**152**(6):1282-9
4. Sigurgeirsson B, Boznanski A, Todd G, Vertruyen A, Schuttelaar M, Zhu X, et al. Safety and efficacy of pimecrolimus in atopic dermatitis: a 5-year randomized trial. *Pediatrics* 2015;**135**(4):597-606
5. Doss N, Reitamo S, Dubertret L, Fekete G, Kamoun M, Lahfa M, et al. Superiority of tacrolimus 0.1% ointment compared with fluticasone 0.005% in adults with moderate to severe atopic dermatitis of the face: results from a randomized, double-blind trial. *Br J Dermatol*. 2009;**161**(2):427-34
6. Doss N, Kamoun M, Dubertret L, Cambazard F, Remitz A, Lahfa M, et al. Efficacy of tacrolimus 0.03% ointment as second-line treatment for children with moderate-to-severe atopic dermatitis: evidence from a randomized, double-blind non-inferiority trial vs. fluticasone 0.005% ointment. *Pediatr Allergy Immunol*. 2010;**21**(2 Pt 1):321-9.
7. Luger T, Van Leent E, Graeber M, Hedgecock S, Thurston M, Kandra A, et al. SDZ ASM 981: an emerging safe and effective treatment for atopic dermatitis. *Br J Dermatol*. 2001;**144**(4):788-94
8. Reitamo S, Rustin M, Ruzicka T, Cambazard F, Kalimo K, Friedmann P, et al. Efficacy and safety of tacrolimus ointment compared with that of hydrocortisone butyrate ointment in adult patients with atopic dermatitis. *J Allergy Clin Immunol*. 2002;**109(**3):547-55
9. Hofman T, Cranswick N, Kuna P, Boznanski A, Latos T, Gold M, et al. Tacrolimus ointment does not affect the immediate response to vaccination, the generation of immune memory, or humoral and cell-mediated immunity in children. *Arch Dis Child*. 2006;**91**(11):905-10
10. Sikder M, Al Mamun S, Khan R, Chowdhury A, Khan H, Hoque M. Topical 0.03% tacrolimus ointment, 0.05% clobetasone butyrate cream alone and their combination in older children with atopic dermatitis - An open randomized comparative study. *J Pak Assoc Dermatol*. 2005;**15**(4):304-12
11. Reitamo S, Van Leent E, Ho V, Harper J, Ruzicka T, Kalimo K, et al. Efficacy and safety of tacrolimus ointment compared with that of hydrocortisone acetate ointment in children with atopic dermatitis. *J Allergy Clin Immunol*. 2002;**109**(3):539-46
12. Reitamo S, Harper J, Bos J, Cambazard F, Bruijnzeel-Koomen C, Valk P, et al. 0.03% Tacrolimus ointment applied once or twice daily is more efficacious than 1% hydrocortisone acetate in children with moderate to severe atopic dermatitis: results of a randomized double-blind controlled trial. *Br J Dermatol*. 2004;**150**(3):554-62

For peer review only - http://bmjopen.bmj.com/site/about/guidelines.xhtml

PRISMA 2009 Checklist

Section/topic	#	Checklist item	Reported on page #
**TITLE**			
Title	1	Identify the report as a systematic review, meta-analysis, or both.	1,3
**ABSTRACT**			
Structured summary	2	Provide a structured summary including, as applicable: background; objectives; data sources; study eligibility criteria, participants, and interventions; study appraisal and synthesis methods; results; limitations; conclusions and implications of key findings; systematic review registration number.	3
**INTRODUCTION**			
Rationale	3	Describe the rationale for the review in the context of what is already known.	5
Objectives	4	Provide an explicit statement of questions being addressed with reference to participants, interventions, comparisons, outcomes, and study design (PICOS).	5,6
**METHODS**			
Protocol and registration	5	Indicate if a review protocol exists, if and where it can be accessed (e.g., Web address), and, if available, provide registration information including registration number.	6
Eligibility criteria	6	Specify study characteristics (e.g., PICOS, length of follow-up) and report characteristics (e.g., years considered, language, publication status) used as criteria for eligibility, giving rationale.	6
Information sources	7	Describe all information sources (e.g., databases with dates of coverage, contact with study authors to identify additional studies) in the search and date last searched.	6
Search	8	Present full electronic search strategy for at least one database, including any limits used, such that it could be repeated.	Appendix 1
Study selection	9	State the process for selecting studies (i.e., screening, eligibility, included in systematic review, and, if applicable, included in the meta-analysis).	6,7
Data collection process	10	Describe method of data extraction from reports (e.g., piloted forms, independently, in duplicate) and any processes for obtaining and confirming data from investigators.	7
Data items	11	List and define all variables for which data were sought (e.g., PICOS, funding sources) and any assumptions and simplifications made.	7
Risk of bias in individual studies	12	Describe methods used for assessing risk of bias of individual studies (including specification of whether this was done at the study or outcome level), and how this information is to be used in any data synthesis.	7
Summary measures	13	State the principal summary measures (e.g., risk ratio, difference in means).	7,8
Synthesis of results	14	Describe the methods of handling data and combining results of studies, if done, including measures of consistency (e.g., I^2) for each meta-analysis.	7

For peer review only - http://bmjopen.bmj.com/site/about/guidelines.xhtml

PRISMA 2009 Checklist

Section/topic	#	Checklist item	Reported on page #
Risk of bias across studies	15	Specify any assessment of risk of bias that may affect the cumulative evidence (e.g., publication bias, selective reporting within studies).	7
Additional analyses	16	Describe methods of additional analyses (e.g., sensitivity or subgroup analyses, meta-regression), if done, indicating which were pre-specified.	7
**RESULTS**			
Study selection	17	Give numbers of studies screened, assessed for eligibility, and included in the review, with reasons for exclusions at each stage, ideally with a flow diagram.	9 & fig 1
Study characteristics	18	For each study, present characteristics for which data were extracted (e.g., study size, PICOS, follow-up period) and provide the citations.	10-14 & appendix 6
Risk of bias within studies	19	Present data on risk of bias of each study and, if available, any outcome level assessment (see item 12).	10-14, 24-25 & appendix 4
Results of individual studies	20	For all outcomes considered (benefits or harms), present, for each study: (a) simple summary data for each intervention group (b) effect estimates and confidence intervals, ideally with a forest plot.	10-14 & appendix 7,8
Synthesis of results	21	Present results of each meta-analysis done, including confidence intervals and measures of consistency.	10-14
Risk of bias across studies	22	Present results of any assessment of risk of bias across studies (see Item 15).	10-14, 24-25 & appendix 4
Additional analysis	23	Give results of additional analyses, if done (e.g., sensitivity or subgroup analyses, meta-regression [see Item 16]).	10-14 & appendix 9
**DISCUSSION**			
Summary of evidence	24	Summarize the main findings including the strength of evidence for each main outcome; consider their relevance to key groups (e.g., healthcare providers, users, and policy makers).	14
Limitations	25	Discuss limitations at study and outcome level (e.g., risk of bias), and at review-level (e.g., incomplete retrieval of identified research, reporting bias).	14, 15

For peer review only

PRISMA 2009 Checklist

Conclusions	26	Provide a general interpretation of the results in the context of other evidence, and implications for future research.	15
**FUNDING**			
Funding	27	Describe sources of funding for the systematic review and other support (e.g., supply of data); role of funders for the systematic review.	1

From: Moher D, Liberati A, Tetzlaff J, Altman DG, The PRISMA Group (2009). Preferred Reporting Items for Systematic Reviews and Meta-Analyses: The PRISMA Statement. PLoS Med 6(7): e1000097. doi:10.1371/journal.pmed1000097

For more information, visit: **www.prisma-statement.org**.

For peer review only

For peer review only - http://bmjopen.bmj.com/site/about/guidelines.xhtml
